# A “Ligand First” Approach toward Selective, Covalent JNK2/3 Inhibitors

**DOI:** 10.1021/acs.jmedchem.5c00884

**Published:** 2025-05-22

**Authors:** Valentin R. Wydra, Nicole Plank, Stefan Zwirner, Roland Selig, Alexander Rasch, Benedikt Masberg, Michael Lämmerhofer, Lars Zender, Pierre Koch, Wolfgang Albrecht, Stefan Laufer

**Affiliations:** † Department of Pharmaceutical/Medicinal Chemistry, 9188 Eberhard Karls Universität Tübingen, Auf der Morgenstelle 8, 72076 Tübingen, DE, Germany; ‡ Department of Pharmaceutical/Medicinal Chemistry II, Institute of Pharmacy, 9147 University of Regensburg, Universitätsstraße 31, 93053 Regensburg, Germany; § Department of Medical Oncology and Pneumology (Internal Medicine VIII), University Hospital of Tübingen, Otfried-Müller-Straße 14, 72076 Tübingen, Germany; ∥ HepaRegenix GmbH, Eisenbahnstraße 63, 72072 Tübingen, Germany; ⊥ Pharmaceutical (Bio-) Analysis, Institute of Pharmaceutical Sciences, Department of Pharmaceutical/Medicinal Chemistry, Eberhard Karls Universität Tübingen, Tübingen 72076, Germany; # IFIT Cluster of Excellence EXC 2180 ‘Image Guided and Functionally Instructed Tumor Therapies’, Eberhard Karls University of Tübingen, 72076 Tübingen, Germany; ¶ German Cancer Research Consortium (DKTK), Partner Site Tübingen, German Cancer Research Center (DKFZ), 69120 Heidelberg, Germany; ∇ Tübingen Center for Academic Drug Discovery (TüCAD2), Auf der Morgenstelle 8, 72076 Tübingen, DE, Germany

## Abstract

All JNK isoforms play a specific role in various diseases. The role of the JNK2 isoform has so far received little attention compared to its JNK1 and JNK3 counterparts with JNK3 being a potential target for neurodegenerative diseases and an inhibitor with JNK1 bias being currently investigated in clinical trials. Using an iterative, structure-guided optimization approach starting from a reported reversible binding aminopyrazole-derived scaffold, novel highly potent JNK2/3 selective inhibitors were generated (“ligand-first approach”). These reversible inhibitors were further transformed to covalent inhibitors by attaching an electrophilic warhead moiety, able to address a conserved cysteine side chain present in JNKs. Reversible and covalent inhibitors presented in this study show high JNK2/3 isoform selectivity and activity in cells. The covalently acting lead compound **56d** shows good kinetic data with a *k*_inact_/*K*_I_ (JNK2) = 38,200 M^–1^ s^–1^ as well as cellular isoform selectivity and a clean kinome profile.

## Graphical abstract

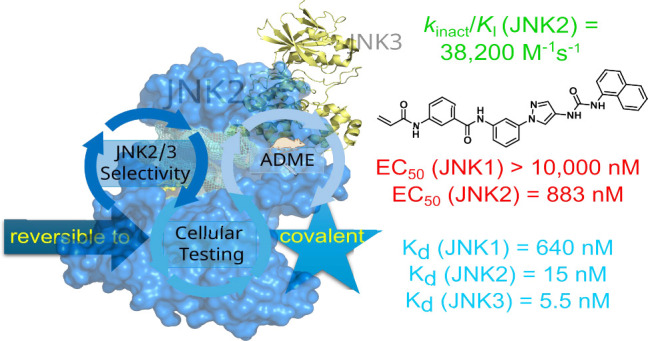

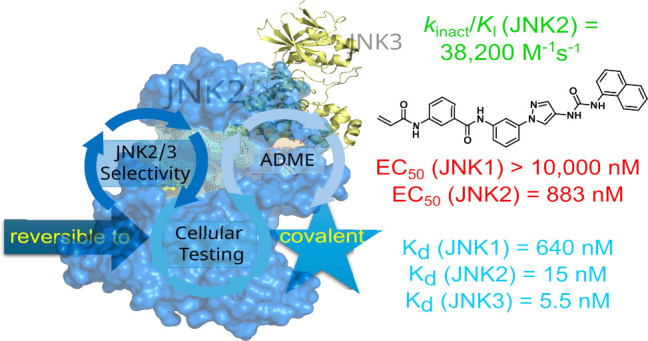

## Introduction

The c-Jun N-terminal kinases (JNKs) belong to the family of mitogen-activated protein kinases (MAPKs) and share a high structural similarity. The JNKs are activated in response to various stress stimuli and possess a wide variety of regulatory functions.^1^ Despite their structural similarity, each individual isoform of the c-Jun N-terminal kinases varies in nuances regarding its substrate interactions, affinities and specificities which in some cases even results in opposing or different regulatory functions among the isoforms within the cell.^2,3^ These differences are also associated with different pathophysiological outcomes, making each individual isoform a specific target linked to different diseases.^4–6^ Targeting of JNK1 might result in clinical benefits for the treatment of pulmonary fibrosis and hepatic diseases.^7^ JNK3 is strongly associated with neurodegenerative diseases like Parkinson’s, Alzheimer’s, and Huntington’s disease, and stroke.^8–10^ The clinical relevance of JNK2 is not as well established as for JNK1 and JNK3. One study showed that silencing of both JNK2 and the upstream mitogen-activated protein kinase kinase 4 (MKK4) via small interfering RNAs had a beneficial effect, improving hepatocyte repopulation and liver regeneration in a knockout mouse model, compared to silencing MKK4 alone. In contrast, JNK1 silencing in combination with MKK4 silencing counteracts the regenerative effects resulting from the knockdown of MKK4.^11^ JNK2 was also seen as a possible target against fibrosis.^12^ The high sequence homology, especially within the ATP binding site, between the three isoforms JNK1–3 (>95%) slowed down rapid development of isoform selective JNK inhibitors binding in the ATP binding site (orthosteric).^13,14^ Analysis of the sequence alignment of all the JNK isoforms (Figure 1A) reveals a difference in only three amino acids within the ATP binding pocket (Figure 1B, Val54, Leu77, Leu106 (highlighted in red); JNK2 numbering).

**Figure 1:**
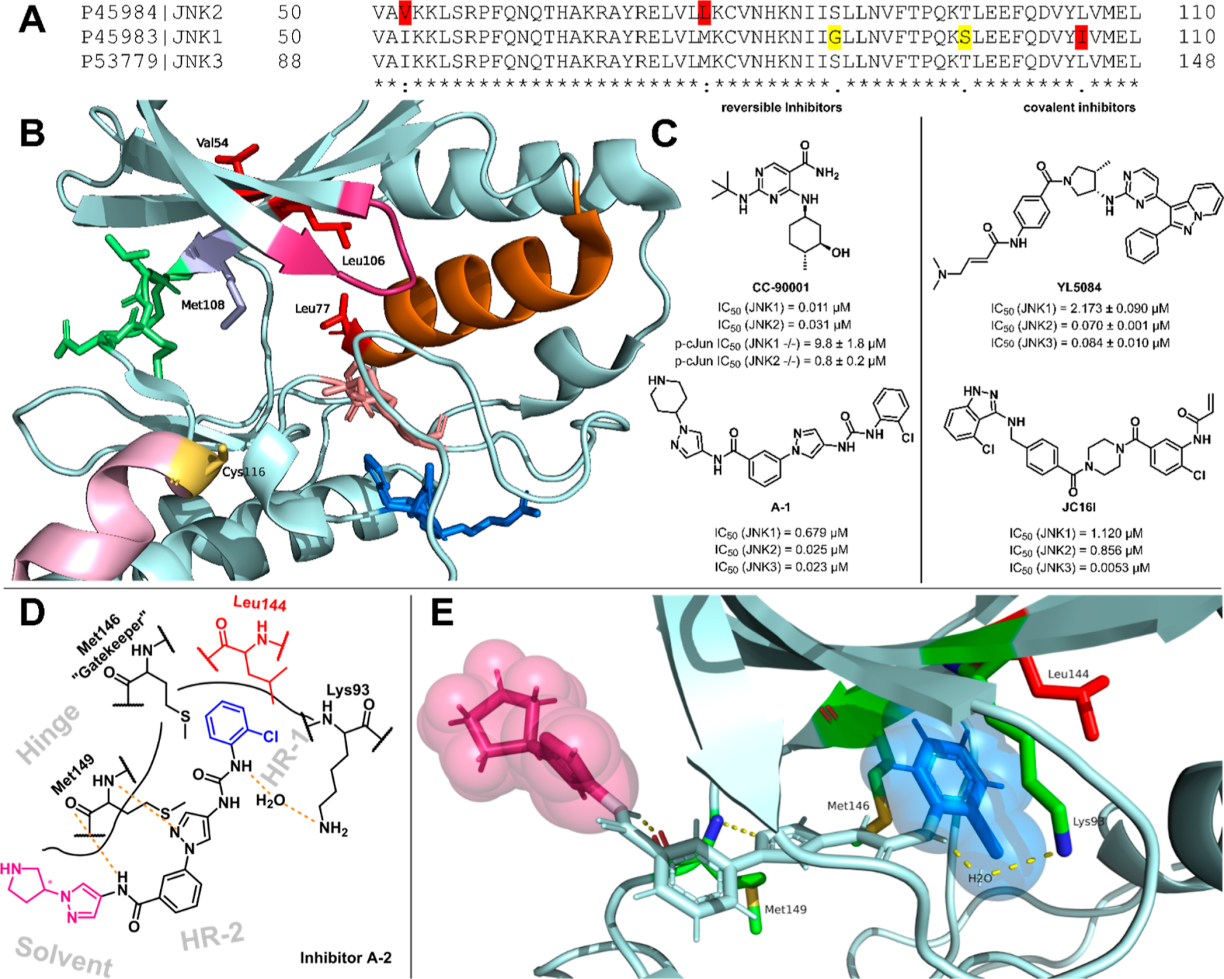
(A) Sequence alignment of human JNK1–3 created with Uniprot.^15^ Differences in the amino acid sequences are highlighted: red = part of the ATP binding pocket; yellow = not part of the binding site. (B) Illustration of JNK2 ATP binding pocket (PDB: 8ELC). Color coding: red = amino acids which differ between the isoforms and are simultaneously part of the ATP binding pocket; green = hinge motif; salmon = DFG motif; blue = HRD motif; pink = αD helix; magenta = P-loop; orange = αC helix; light blue = M108: gatekeeper amino acid; yellow = C116: cysteine in prominent αD+2 location only conserved in JNK isoforms within the cysteinome.^16^ (C) Examples of isoform selective JNK inhibitors CC-90001,^7^ inhibitor **A-1**,^17^ YL5084,^18^ JC16I.^19^ IC_50_ values taken from the respective publication. (D) Schematic depiction of inhibitor **A-2** binding to the ATP binding pocket of JNK3. (E) Illustration of inhibitor **A-2** Binding to JNK3 (PDB: 4WHZ).^17^ Color coding: blue = HR-I motif; magenta = solvent interacting moiety; red = Leu144 (JNK3) responsible for JNK2/3 selectivity of aminopyrazole derived inhibitors.^14^

Pyrimidine-carboxamide CC-90001 (BMS-986360) reported by Nagy et al. showed a slight preference for the JNK1-isoform in cellular assay settings. However, this isoform selectivity was less pronounced in the biochemical activity assay (Figure 1C).^7^ There have been ambitious efforts to bring this inhibitor into clinical trials. Two studies with CC-90001 have been reported in patients with pulmonary fibrosis (NCT03142191) and with nonalcoholic steatohepatitis and liver fibrosis (NCT04048876). Currently, patients are recruited for a phase-1 study of CC-90001 for its use in patients with advanced solid tumors (NCT05625412). In case of the JNK2 isoform, one well performing selective inhibitor was part of a covalent approach reported by the Gray group (Figure 1C, YL5084).^18^ This series could be seen as a further development of the pan-JNK probe JNK-IN-8, targeting a conserved cysteine at a prominent αD + 2 location, only occurring within the JNK isoforms (Figure 1B, Cys116 highlighted in yellow).^16,20^ Great progress has been made recently in the development of irreversible JNK3 isoform selective, orthosteric inhibitors. The covalent JNK3 inhibitor JC16I is a promising candidate to reach the status of a high quality chemical probe (Figure 1C).^19,21^

In 2014, Zheng et al. reported a series of aminopyrazole derivatives as reversible JNK3 inhibitors displaying selectivity versus JNK1.^17^ In a follow-up study Park et al. demonstrated, using X-ray crystallography and mutagenesis experiments, that the isoform selectivity toward JNK2/3 of the studied aminopyrazole-based inhibitors is mostly driven by a single amino acid difference in the hydrophobic region-I (HR-I) (Figure 1D + E, Leu106-JNK2/Leu144-JNK3 versus Ile106-JNK1 highlighted in red).^14^ Aminopyrazole **A-1** is a potent balanced dual JNK2/3 inhibitor showing modest selectivity versus JNK1 (Figure 1C).^17^ X-ray analysis of a derivative of a close analog of **A-1** (aminopyrazole **A-2**) in complex with JNK3 revealed the binding mode for this class of reversible JNK inhibitors (Figure 1E). The N2 of the central pyrazole ring as well as the NH of the amide group form hydrogen bond interactions to the backbone of Met149 of the hinge-region. The 2-chlorophenyl moiety is located in the HR-I. The pyrrolidinyl pyrazole moiety is located in the solvent exposed area.

The aim of the presented study was to identify novel selective JNK2/3 inhibitors. In a first step, the reversible ligands were optimized for JNK2/3 selectivity (“ligand first”). In a second step, increased potency as well as selectivity within the kinome should be achieved through the introduction of an electrophilic warhead. The compounds may serve as tool compounds to further evaluate the role of both enzymes in various disorders. As lead structure, we chose the ATP-competitive aminopyrazole **A-1**. This compound has already demonstrated 30-fold selectivity versus JNK1 and structure activity relationships (SAR) for this class of compounds have already been reported.^14,17,22,23^ We extended the SAR reported by Zheng et al. and evaluated our lead compounds employing cellular assays, a metabolic stability assay and a pharmacokinetic (PK) study. Finally, we evolved this scaffold in a covalent approach to an irreversible JNK2/3 inhibitor.

## Results and Discussion

With our first modifications of inhibitor **A-1** we tried to address the “selectivity pocket “ (HR-I), as designated by Kamenecka et al.^22^ This was only consequent to improve JNK2/3 selectivity, since it can be assumed that more space could be occupied within the HR-I in JNK2 and JNK3 compared to JNK1 due to the Leu­(JNK2/3)/Ile­(JNK1) difference as described above. Therefore, by replacing the 2-chlorophenyl moiety with different hydrophobic groups we were able to scan the three-dimensional space within the HR-I of each individual JNK-isoform (Table 1).

**Table 1:**
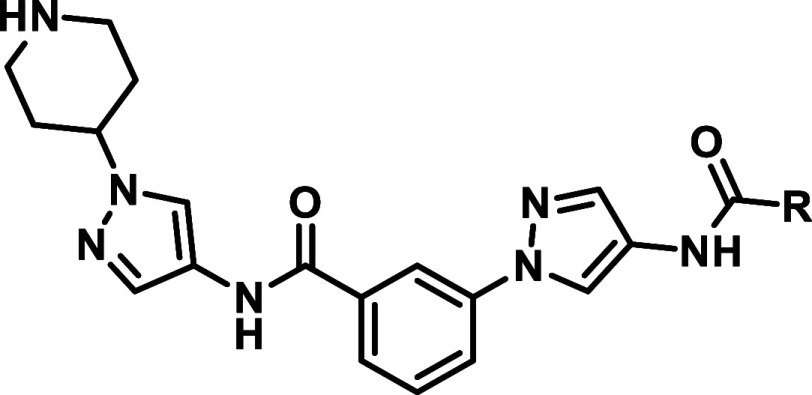

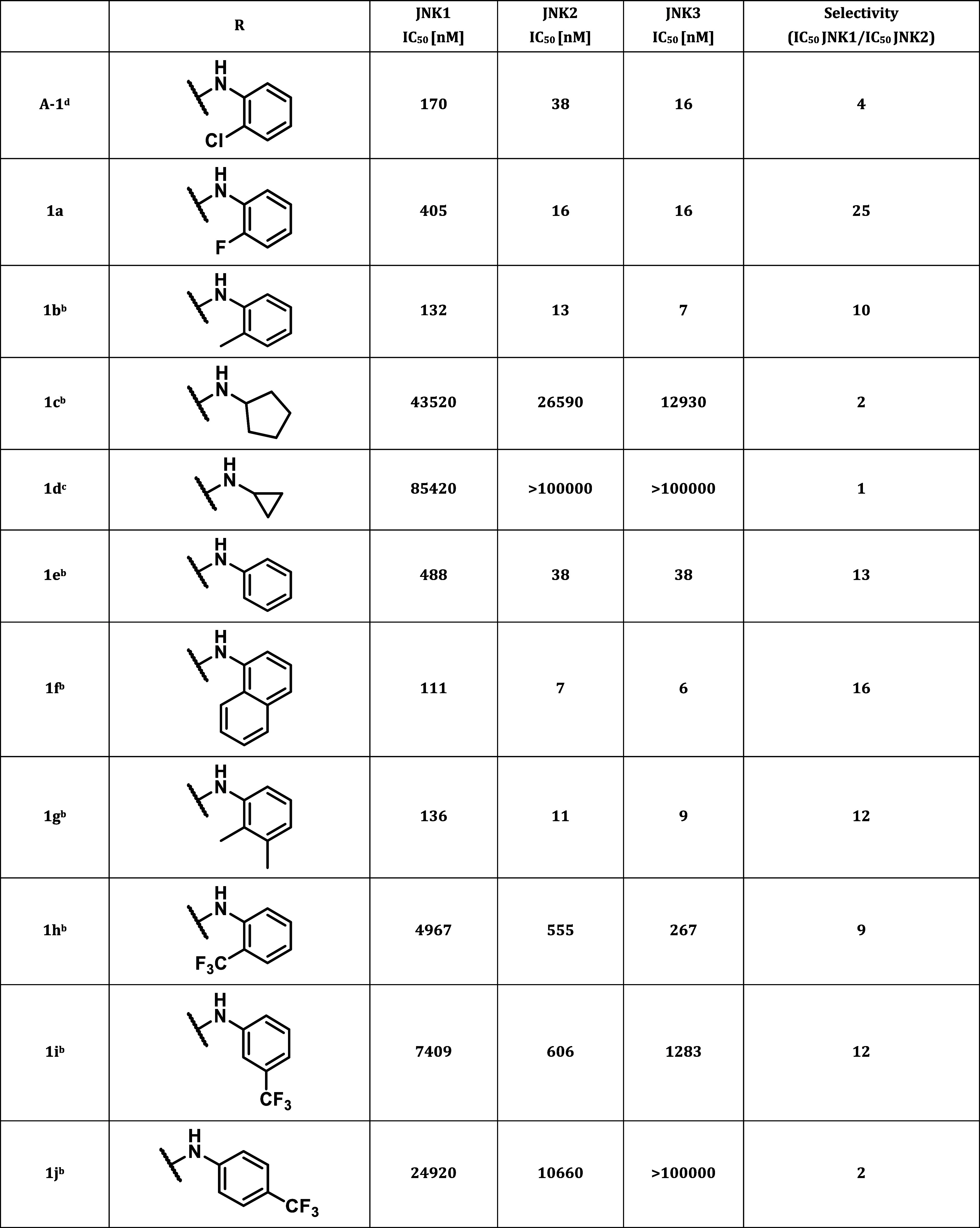
Compound Series Designed to Address the HR-I Moiety^a^

a IC_50_ determination employing the ^33^PanQuinase assay, measured in singlicates for all three JNK isoforms. Selectivity is represented by the ratio to JNK2.

b Isolated as HCl salt.

c Isolated as TFA salt.

d Isolated as HCl salt and corresponding free base, here free base was tested.

The published IC_50_-values for **A-1** could be partially reproduced by the commercial assay format performed by Reaction Biology (^33^PanQinase).^17,24^ However, with the applied conditions, the inhibitor was more active on JNK1, as reported in literature and therefore less JNK2/3 selective than reported. Replacement of the 2-chloro substituent with a fluorine atom slightly improved selectivity, while introducing more bulky alicyclic substituents resulted in compounds with no or low inhibitory activity on all isoforms (**1c**, **1d**). All CF_3_ substitutions (**1h**, **1i** and **1j**) resulted in decreased activity, with the para position being the least tolerated. In contrast, methylations were significantly better tolerated with **1b** and **1g** exhibiting a JNK1/JNK2 selectivity ratio comparable to the phenyl derivative **1e**. Further steric extension of **1g** led to the naphthyl derivative **1f** which demonstrated the highest inhibitory activity within this series and acceptable JNK2/3 selectivity (16-fold).

Building on the findings from our first series (Table 1), we retained the naphthyl moiety and applied a downsizing strategy in which the solvent interacting structure was reduced (Table 2). To achieve this, all structures west of the central aromatic core were removed and the aromatic core moiety was replaced by pyridines.

**Table 2:**
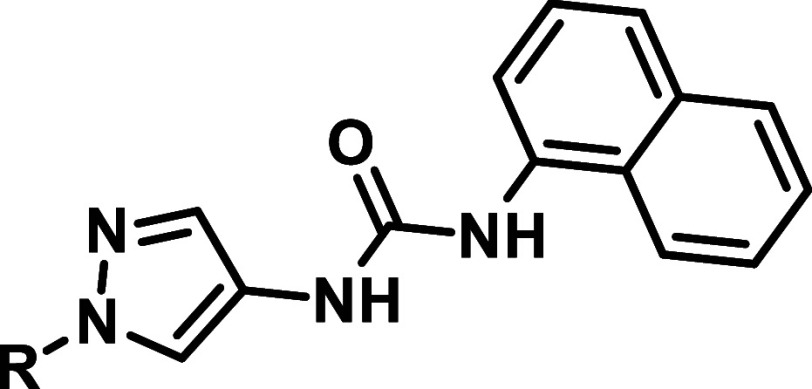

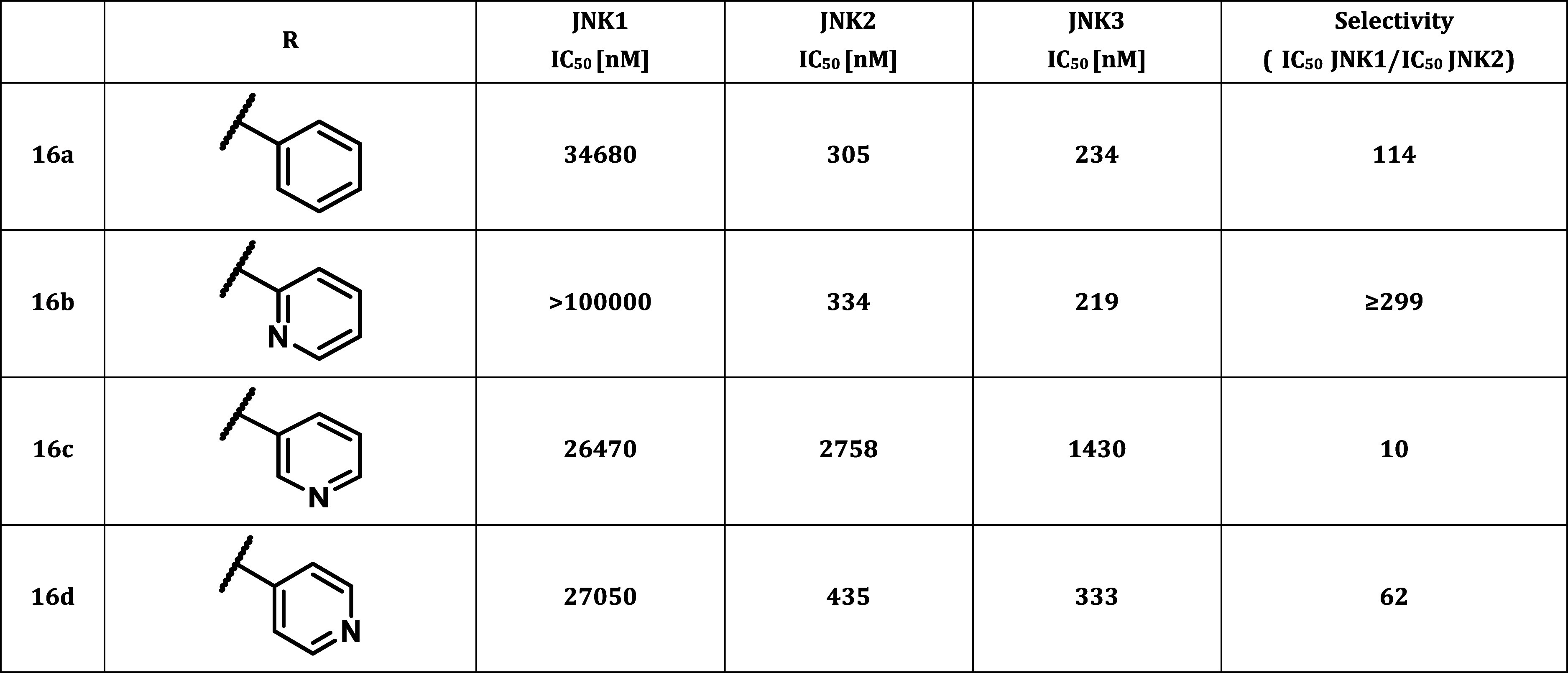
Compounds Lacking Moieties that Previously Interacted with the Solvent or HR-II^a^

a IC_50_ determination employing the ^33^PanQuinase assay, measured in singlicates for all three JNK isoforms. Selectivity is represented by the ratio to JNK2.

In general, inhibitory activity decreased on all isoforms, but to a different extent, leading to significantly more JNK2/3 selective inhibitors. The unsubstituted phenyl derivative **16a** demonstrated an excellent JNK1 to JNK2/3 selectivity ratio showing a 114-fold greater activity on JNK2 and a 148-fold higher activity on JNK3 compared to JNK1. Replacement of the phenyl ring of **16a** by a pyridine ring resulted in compounds **16b**–**d**. The 2-pyridyl derivative **16b** demonstrated even a higher selectivity ratio than compound **16a**. Regioisomeric effects appear to play an important role, as shown by the corresponding 3- and 4-pyridyl derivatives **16c** and **16d**. This series demonstrated that modifications targeting the HR-II lead to more favorable selectivity effects compared to those addressing HR-I. However, this might possibly only be the case if the interaction profile of the planar naphthalene moiety allowed JNK2/3 selectivity in the first place with its HR-I interaction.^14^ Consequently, we shifted our focus to the HR-II region.

In the resulting series, we retained the amide substitution from the initial starting compounds in Table 1, but omitted the second, solvent directed pyrazole moiety (Table 3). This strategy proved successful in the design of JNK3 selective inhibitors, as previously demonstrated by Feng et al.^23,25^

**Table 3:**
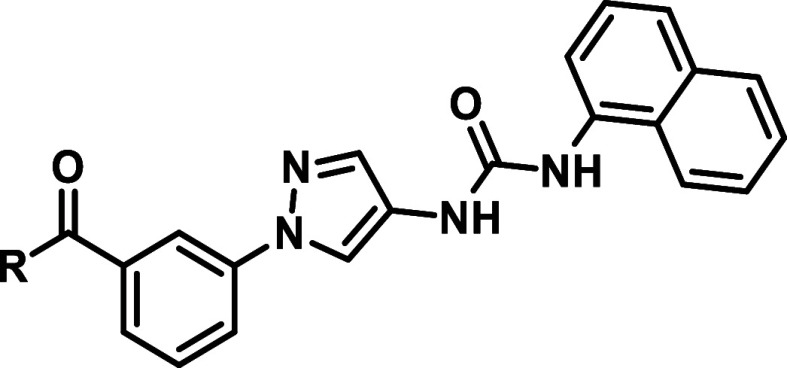

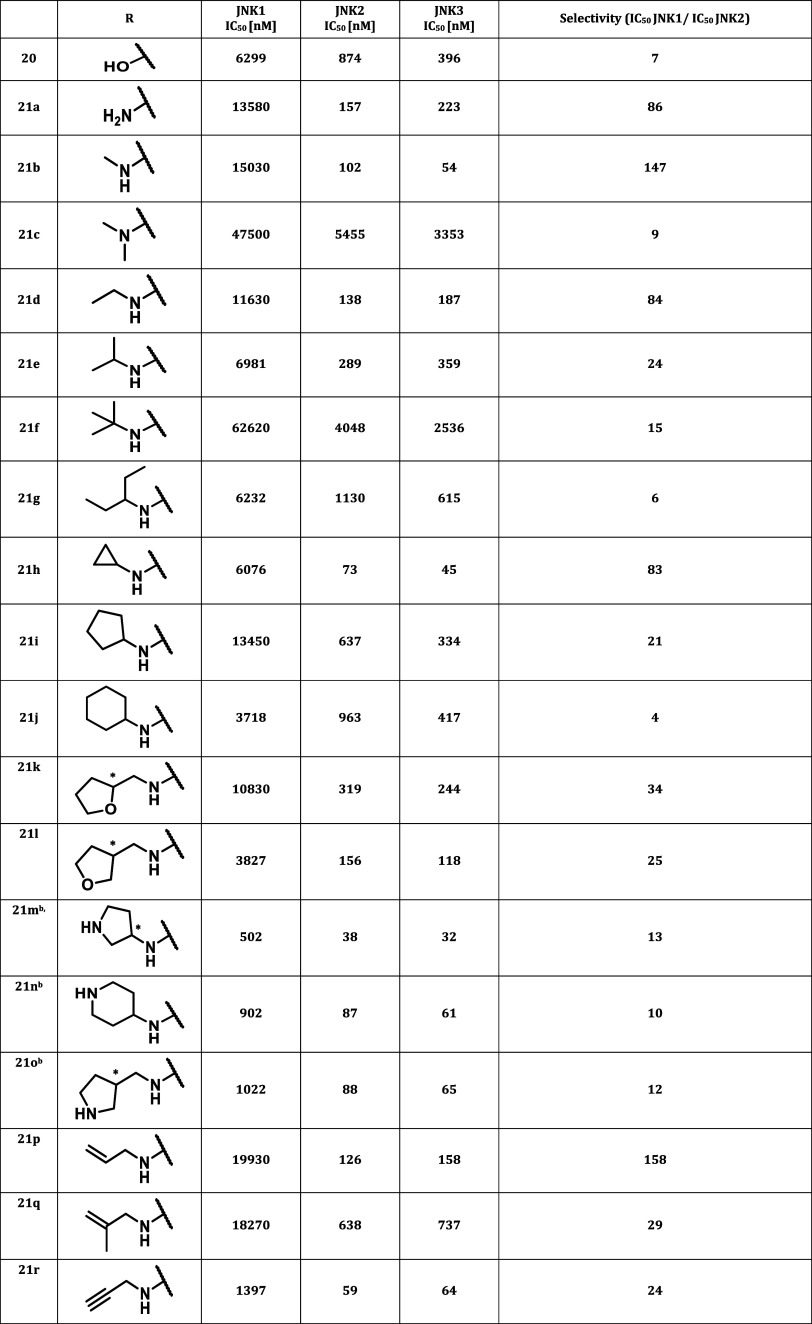
Addressing the Solvent Region/HR-II^a^

a IC_50_ determination employing the ^33^PanQuinase assay, measured in singlicates for all three JNK isoforms. Selectivity is represented by the ratio to JNK2.

b Isolated as HCl salt.

Comparing the benzoic acid (**20**) and benzamide derivative (**21a**), the benzamide was more potent than the corresponding benzoic acid and significantly more selective. Further derivatives were designed by a gradually increasing sterical demand, followed by branching in the aliphatic substitutions to form alicyclic residues, reducing molecular flexibility. As shown in Table 3 the SAR clearly indicates that sterically more demanding aliphatic (**21e**, **21f**) and alicyclic (**21i**, **21j**) residues led to reduced JNK2/3 activities and lower selectivity ratios. Additionally, the reduction in flexibility achieved through cyclization proved advantageous in terms of activity (e.g., comparing **21e** and **21h**). The presence of the hydrogen donor function was also found to be crucial, as demonstrated by compound **21c**. Along with **21h**, **21b** hereby emerged as one of the lead structures showing an excellent selectivity value (148-fold) while retaining activity. To further explore the benzamide series, we extended it to include heteroalicyclic and unsaturated aliphatic compounds. The pyrrolidine and tetrahydrofuryl derivatives (**21k**, **21l**, **21m** and **21o**) showed good JNK2/3 potencies but low selectivities toward JNK1. A similar trend was observed with the corresponding piperidine (**21n**). Further modifications incorporating allylic and propynylic components led to inhibitors **21p**–**21r**, with **21p** being a promising candidate demonstrating activity and selectivity scores comparable to **21b**. Further derivatives with different solvent addressing moieties, including morpholine and pegylated derivatives, were synthesized and tested but are listed in the Supporting Information due to their low relevance (Table S15).

Employing a quite different synthetic approach, para-substituted inhibitors shown in Table 4 were created. However, the generated compounds showed a significant drop in activity on all three isoforms. HR-II/solvent addressing residues were hereby adopted from potent meta-analogs, which were disclosed in Table 3.

**Table 4:**
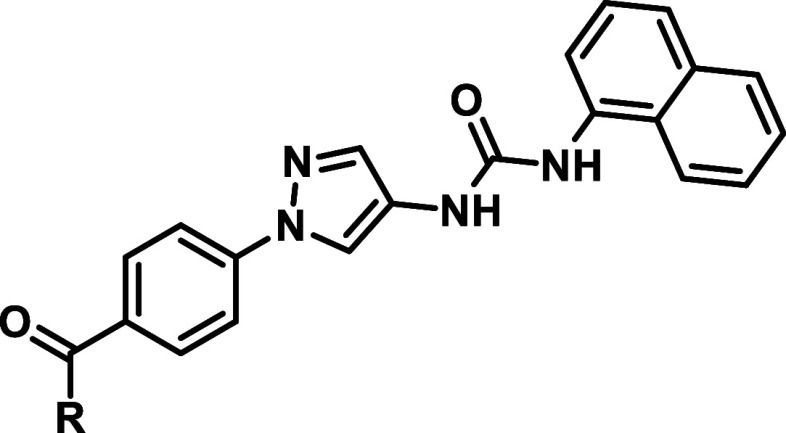

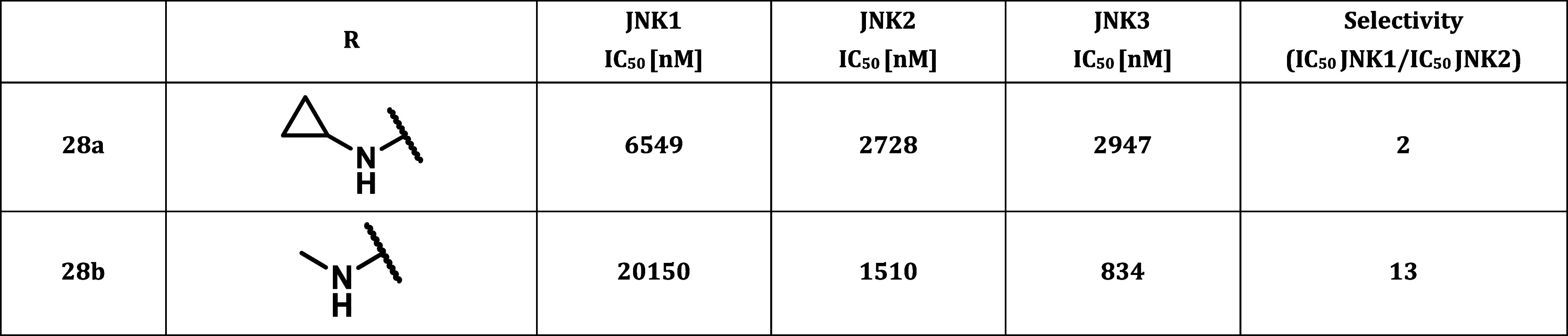
Para-Substituted Derivatives^a^

a IC_50_ determination employing the ^33^PanQuinase assay, measured in singlicates for all three JNK isoforms. Selectivity is represented by the ratio to JNK2.

So far, inhibitor **16b** has demonstrated the highest selectivity among all the derivatives tested (Table 2). To further enhance the series based on inhibitor **A-1**, modifications targeting the HR-I were made, leading to the identification of the naphthyl group as the most promising residue. This group exhibited the highest selectivity compared to other residues in the series (**1f**; Table 1). Building on this, compound **1f** was further developed into **21b**, **21h** and **21p**, incorporating optimized residues designed to target the HR-II (Table 3). Linearization of the scaffold did not prove advantageous and resulted in compounds with low activity (**28a**, **28b**; Table 4).

In an attempt to combine the beneficial features of the molecules presented so far, compounds **35a** and **36a** (Table 5) were synthesized. These compounds included modifications to HR-I and HR-II addressing moieties and the core scaffold. However, despite the strategic combination of the acquired SAR, the overall balance of the structural changes did not yield the expected improvements in either potency or selectivity. Neither the insertion of nitrogen at position 4 (**35a**) nor at position 6 (**36a**) resulted in acceptable activity values for JNK2 and unfortunately, both potency and selectivity decreased significantly compared to the initial compounds (**16b** and **21b**).

**Table 5:**
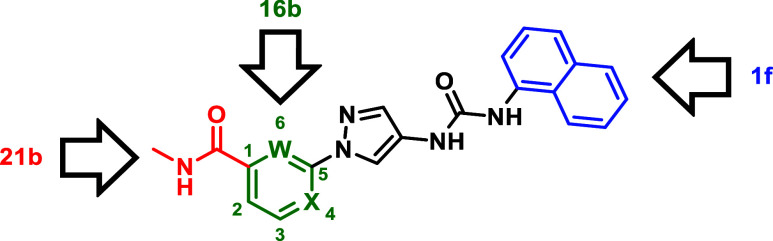
Pyridine as the Central Aromatic Core of Inhibitors with Optimized HR-I and HR-II/Solvent Addressing Moieties^a^

	W	X	JNK1 IC_50_ **[nM]**	JNK2 IC_50_ **[nM]**	JNK3 IC_50_ **[nM]**	selectivity (IC_50_ JNK1/IC_50_ JNK2)
**35a**	**CH**	**N**	44,840	**533**	**630**	**84**
**36a**	**N**	**CH**	20,550	400	**686**	**51**

a IC_50_ determination employing the ^33^PanQuinase assay, measured in singlicates for all three JNK isoforms. Selectivity is represented by the ratio to JNK2.

Further modifications were made to the aromatic core moiety (Table 6). In this series, methylations were primarily investigated using the scaffolds of inhibitors **21h** and **1f**.

**Table 6:**
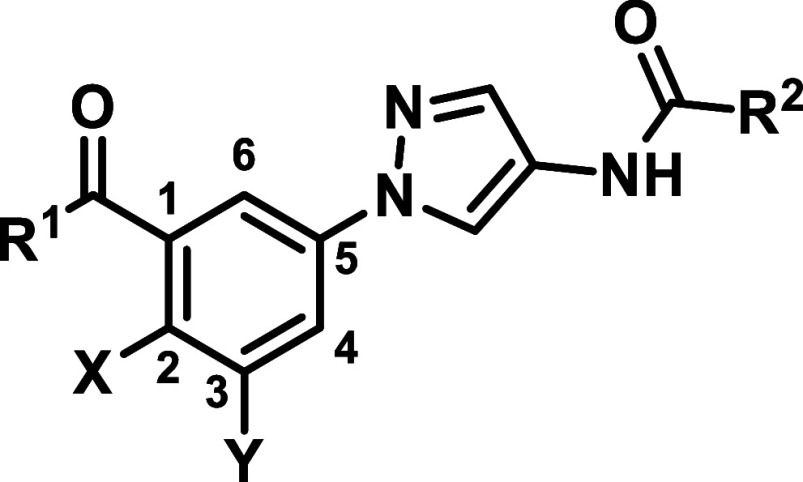

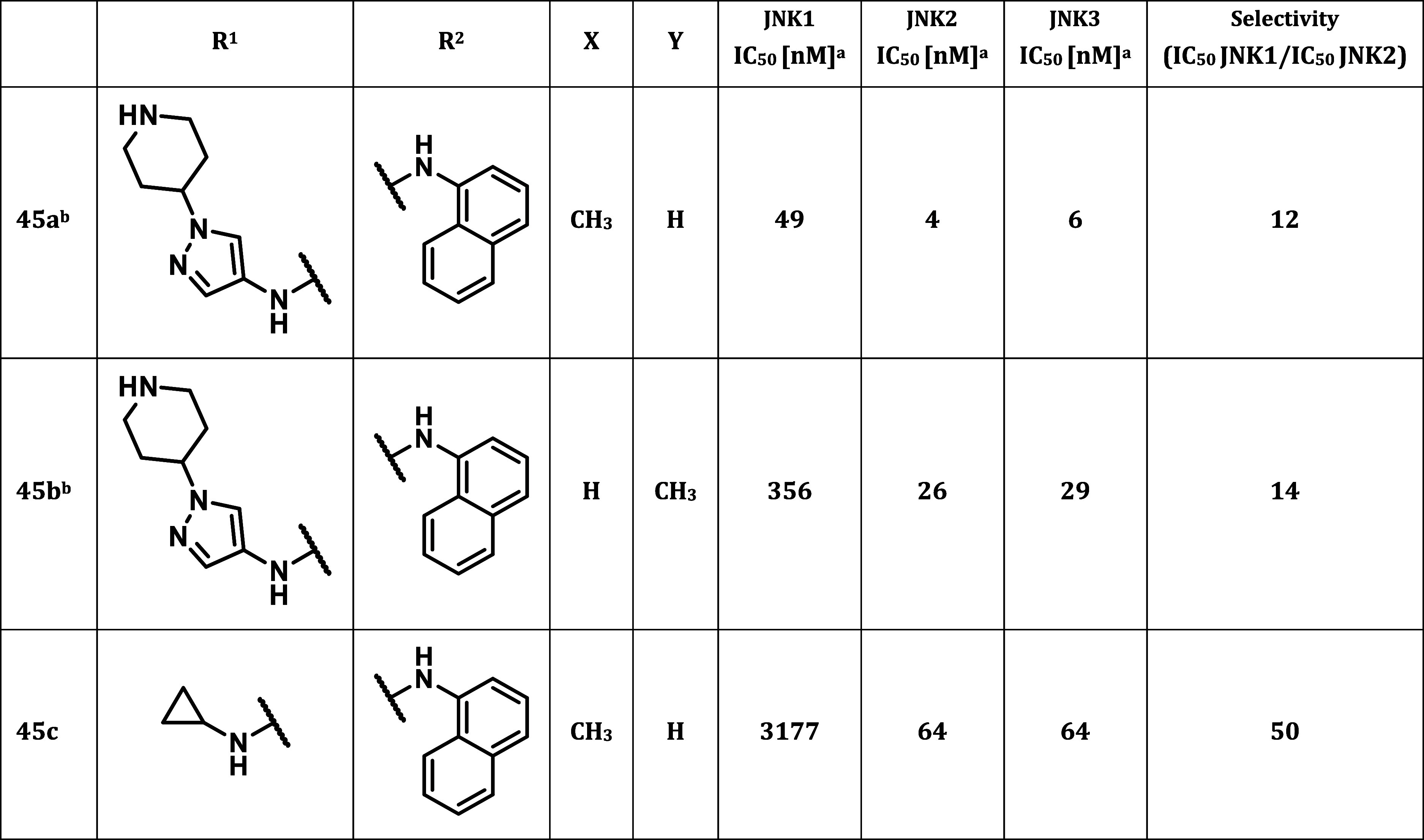
Derivatives Carrying a Methyl Group on the Aromatic Core^a^

a IC_50_ determination employing the ^33^PanQuinase assay, measured in singlicates for all three JNK isoforms. Selectivity is represented by the ratio to JNK2.

b Isolated as HCl salt.

Methylation of **1f** resulted in highly potent inhibitors **45a** and **45b**, with **45a** being the most potent inhibitor tested, showing activities in the single-digit nanomolar range. Furthermore, the selectivities of **45a** and **45b** did not differ greatly from the parent compound **1f**. Building on the observed small activity boost from methylation at the 2 position, compound **45c** was synthesized as a direct analogue of **21h**, which showed slightly better activity on JNK2 but also on JNK1.

As the last optimization attempt of our SAR studies on reversible inhibitors, we focused more on the western amide moiety (Table 7). Removing the amide’s carboxyl group, to leave only the amine function, resulted in a loss of activity toward JNK2/3 (**51a/b**). However, attaching a 4-pyridine-moiety via reductive amination also led to compound **51a** possessing good selectivity.

**Table 7:**
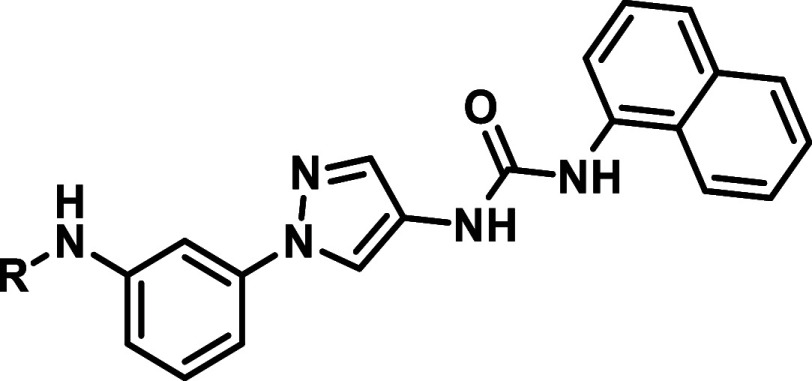

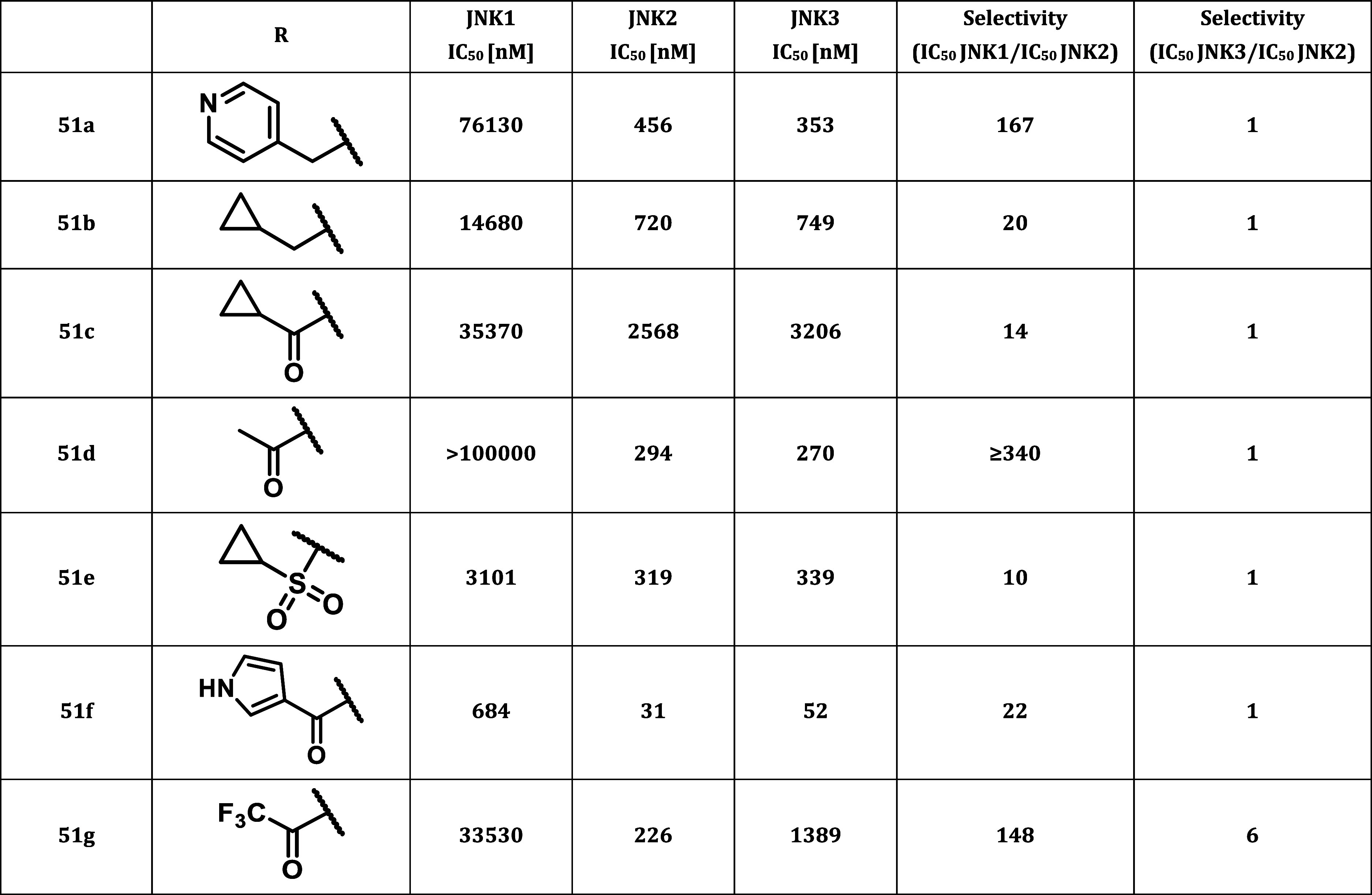
Inverting/Replacing the Western Amide Moiety^a^

a IC_50_ determination employing the ^33^PanQuinase assay, measured in singlicates for all three JNK isoforms. Selectivity is represented by the ratio to JNK2.

The introduction of inverted amides generated inhibitors displaying sharp SAR. Compounds with short aliphatic, noncyclic inverted amides attached, showed great improvements toward JNK1/JNK2 selectivity, as shown by inhibitor **51d**. Although in comparison to the analogous amide **21b**, **51d** was slightly less potent. However, the inverted amide analog to inhibitor **21h**, **51c** showed strongly diminished activity and selectivity values. If aromatic moieties were incorporated likewise, the inhibitors showed a lower selectivity, but a double-digit nanomolar activity (**51f**). An inverted sulfonamide displayed much less selectivity toward JNK2/3 than simple amides (**51e**) and a derivative carrying a more electron-withdrawing trifluoroacetamide showed not only JNK1/JNK2 selectivity, while retaining reasonable JNK2 potency, but seemed to be able to slightly discriminated between the JNK2 and JNK3 isoform, showing 6-fold higher activity on JNK2 (**51g**).

Encouraged by the high JNK1/JNK2 selectivity values of several promising compounds, we shifted our focus onto cellular assays and metabolic and PK studies. We then decided to investigate cellular activity employing inhibitors **21b** and **21h**, because of their high affinity and excellent selectivity profile. Because of their fast doubling time we used adherent murine hepatocellular carcinoma cells (*Nras* ^*G12V*^ *Cdkn2a* ^*ARF*–/–^ HCC cells) for the subsequent experiment. When these inhibitors were analyzed for their ability to inhibit the phosphorylation of the downstream substrate and transcription factor c-Jun in cells, the activity of **21b** was comparable to SP600125, a well-established pan-JNK inhibitor (Figure 2).^26^ The cells were exposed to 1 h of an osmotic stimulus with 133 mM sorbitol solution after 2 h of inhibitor preincubation. The inhibition data for the reference inhibitor nicely match the already published data with an activity of approximately 3.33 μM (5–10 μM published), even though the cell system was changed from humane Jurkat T lymphocytes to murine hepatocellular carcinoma cells.^26^ Although **21b** is less active in the biochemical assay than **A-1**, both inhibited c-Jun phosphorylation at about 1.11 μM. Presumably the piperidine moiety incorporated in **A-1** is positively charged at physiological pH and might therefore be less cell permeable than **21b**.

**Figure 2:**
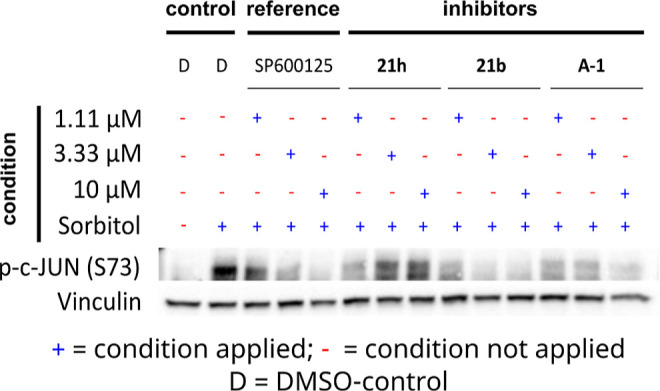
Western Blot analysis of whole cell lysates derived from adherend murine *Nras* ^*G12V*^; *Cdkn2a* ^*ARF*–/–^ hepatocellular carcinoma (HCC) cells. Cells were incubated with different JNK2/3 inhibitors (**21h**, **21b**, **A-1** (tested as HCl salt)) as well as the known reference pan-JNK inhibitor SP600125 each at three different concentrations (1.11 μM, 3.33 μM, 10 μM) for 3 h. As a control group DMSO (D) was used instead of compound. To induce the JNK pathway and phosphorylation of c-Jun an osmotic stimulus of 133 mM sorbitol was added to the cells after 2 h of preincubation. DMSO treated cells were either stimulated with 133 mM Sorbitol as an activator of high c-Jun phosphorylation (positive control) or not stimulated as a reference for basal c-Jun phosphorylation levels (negative control). Blots were incubated with p-c-Jun antibody (detecting p-c-Jun phosphorylated at Ser73). As loading control vinculin is shown. The blots were analyzed via a ChemiDoc MP imaging system. Experiment was performed in triplicates. For Vinculin (∼116 kDa) and p-c-Jun (S73) (∼48 kDa) only bands of interest are displayed. Applied conditions are marked with a + and unapplied conditions are marked with a -.

Interestingly at higher concentrations, **21h** showed an increased occurrence of p-c-Jun compared to a lower concentration (1.11 μM). This effect could be explained by **21h** also addressing side-targets at higher concentrations.

Since our compounds were able to penetrate cells, and with **21b** showing downstream target cellular activity, we conclusively subjected our lead compounds to metabolic studies. Initially we tested the metabolic stability in mouse liver microsomes. As shown in Figure 3A, all three JNK2/3 selective inhibitors (**21h**, **21b** and **51d**) remained >90% intact after 2 h of incubation, while the positive control Verapamil was converted by the microsomes to <60% quantity compared to the corresponding concentration at *t*_0_. The metabolites found by mass spectrometry for all three inhibitors had the mass [M+16] which indicates hydroxylation or oxidation. Interestingly, also an inversion of the western amide moiety did not result in a significant decrease in metabolic stability, concluding that this center is not susceptible to amidases in general.

**Figure 3:**
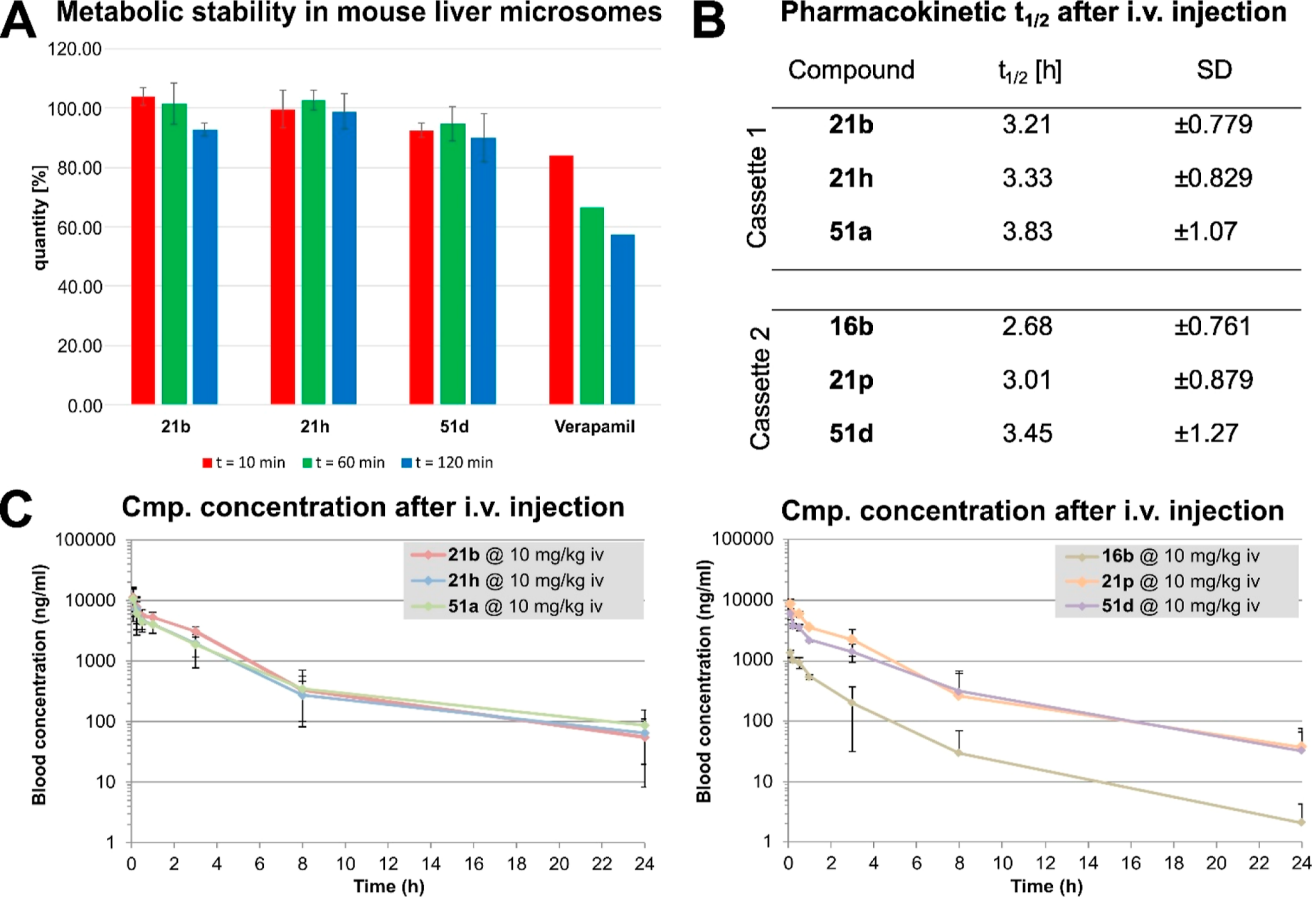
PK and metabolism studies. (A) Evaluation of metabolic stability in mouse liver microsomes of inhibitors **21b**, **21h** and **51d**. Only representative time spots are shown: *t* = 10/60 and 120 min. All values are depicted in the Supporting Information (Figure S3). Verapamil was chosen as reference/positive control. (B,C) Pharmacokinetic study via cassette dosing in mice. Cassettes were administered via i.v. injection into the lateral tail vein at a dosage of 10 mg/kg. Blood samples were taken after seven consecutive time spots: 5 min, 15 min, 30 min, 1 h, 3 h, 8 h, 24 h post dose. (B) Tabular representation of the elimination half-life of the PK study. (C) All three blots of each cassette summarized and linearized.

In a follow-up pharmacokinetic study, these compounds as well as three other candidates (**16b**, **21p** and **51a**) were administered in two cassettes via injection into the lateral tail vein of mice at a concentration of 10 mg/kg by bolus i.v. injection. At seven distinct time points blood samples were collected and subsequently analyzed and quantified via liquid chromatography mass spectrometry (LCMS). The plotted logarithmic summary-data (Figure 3C) states classical first order elimination kinetics, presupposing a standard open two-compartment model of each compound. Compound **16b** was eliminated more rapidly. **21h** showed a half-life of 3.33 h (±0.829) (Figure 3B) and persisted in high amounts in the mice for 24 h, with 2.2 times the IC_50_ value of the compound still remaining in the last blood sample (see Table S6). The highest *t*_1/2_ showed **51a** with 3.83 h (±1.07).

Since the inverted amide derivatives showed improvements with regard to their JNK2/3 selectivity (**51d**), but lost a little bit of potency, we searched for further modifications to achieve higher potency, while retaining the high JNK2/3 selectivity. In 2012, the Gray group published selective covalently acting pan-JNK inhibitors featuring acrylamide warheads, which target Cys116 in JNK1/2 respectively Cys154 in JNK3 at the prominent αD + 2 position only occurring within the JNKs.^16,20^ This led to the selective pan-JNK probe JNK-IN-8 (Figure 4B). This approach was also employed by some of us resulting in pyridinylimidazole-based covalent JNK3 inhibitors.^27^ We therefore assumed that by addressing the mentioned Cys116, the inhibitors might regain potency as well as achieve kinome-wide selectivity.^28^ After superimposing the covalent binding mode of the reference inhibitor JNK-IN-7 (PDB: 3V6S) on our aminopyrazole-based scaffold (PDB: 4WHZ; Figure 4A) we concluded, that addressing Cys116 might be possible by connecting an acrylamide warhead with an aminobenzamide linker to our inverted amide scaffold (Figure 4B). The reversible binding component (depicted in Figure 4B) hereby resembles inhibitor **16a**, which also showed high selectivity values toward JNK2/3.

**Figure 4:**
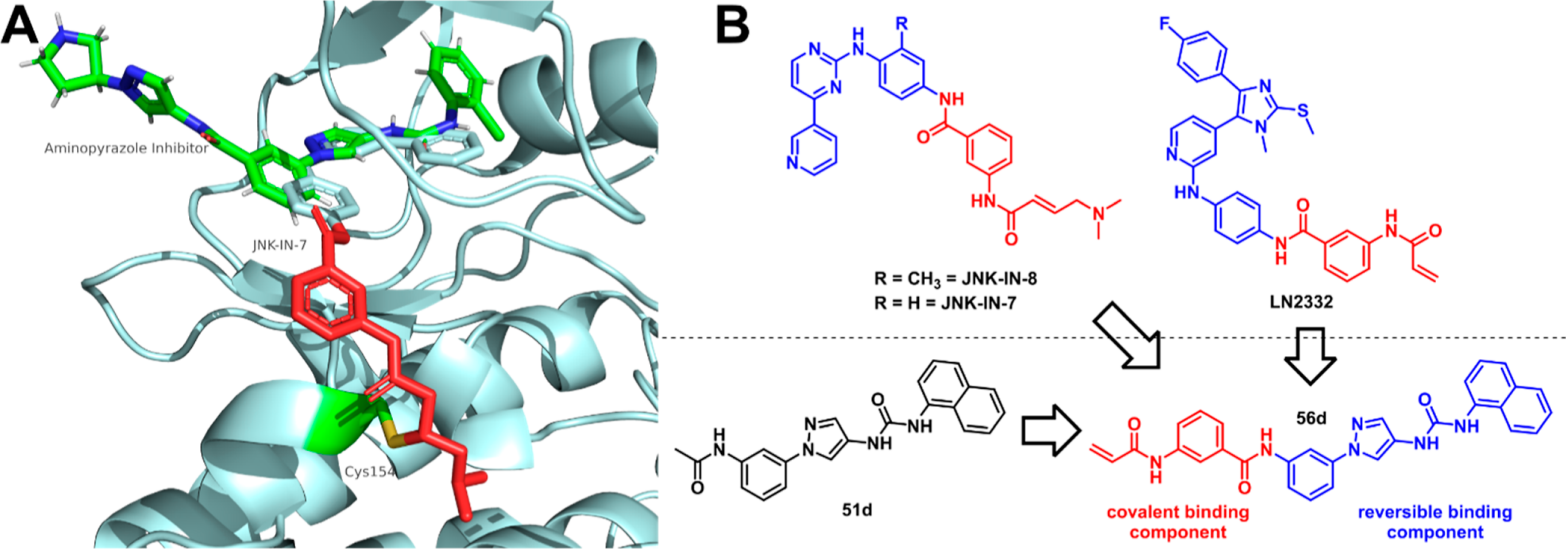
(A) Superimposing of two JNK3 crystal structures bearing a covalent and reversible JNK3 inhibitor, respectively. Reversible binding mode: Inhibitor **A-2** derived from compound **A-1** with the piperidine moiety exchanged with a pyrrolidine moiety (PDB: 4WHZ). Covalent binding mode: Inhibitor JNK-IN-7 covalently bound to Cys154 at the αD+2 position (PDB: 3V6S). (B) Design transfer of already published covalently acting JNK inhibitors onto the reversible aminopyrazole scaffold.

The inhibitors featuring para- and meta-substituted aminobenzamide linkers are presented in Table 8. The prepared acrylamide bearing compounds were investigated alongside their covalently inactive propyl amide analogs (**56a**, **56c**). All inhibitors showed modest IC_50_ values, but the propyl amide derivatives demonstrated lower activity compared to their acrylamide counterparts. Interestingly, the potential covalent candidate **56d** exhibited some potency against JNK2/3 but no activity on JNK1, particularly when compared to the corresponding para-substituted derivative **56b**. Inhibitor **56b** and **56d** were investigated in further assays.

**Table 8:**
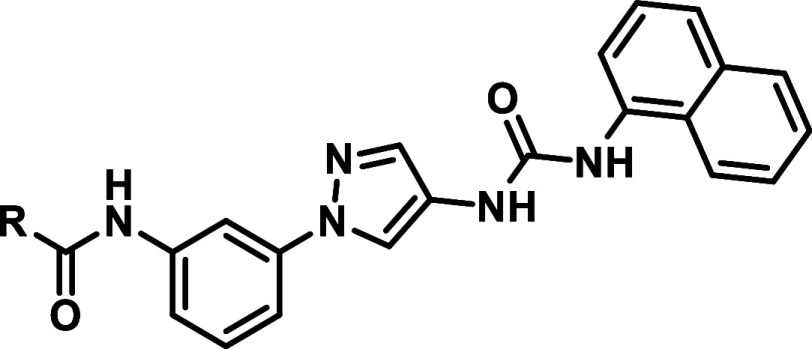

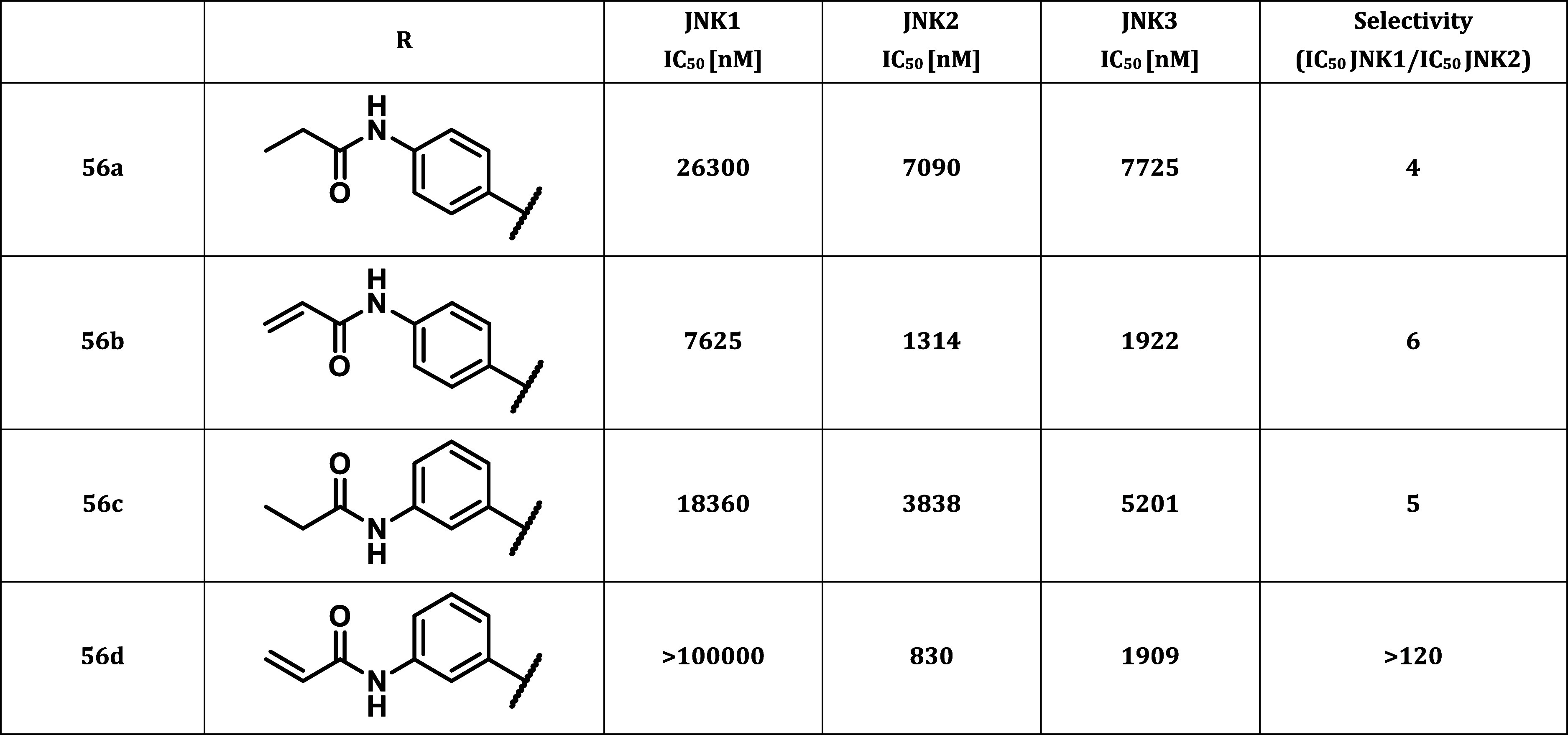
Covalent Approach^a^

a IC_50_ determination employing the ^33^PanQuinase assay, measured in singlicates for all three JNK isoforms. Selectivity is represented by the ratio to JNK2.

Since IC_50_ values should not be solemnly taken into account for the evaluation of potential covalent inhibitors, we employed kinetic studies, to prove the predicted covalent and irreversible mode of action of **56b + d**. Therefore, the time dependency of the inhibition, the binding mode via wash-out experiment and *k*_inact_/*K*_I_ values were determined. Furthermore, an intact protein mass spectrometry (IPMS) experiment was conducted after labeling with **56d**.

Initially, time-dependent inhibition (TDI) was confirmed, employing an IC_50_ determination with a different PhosphoSens testing platform provided by AssayQuant. As depicted in Table 9, the two inhibitors **56b** and **56d** were tested on all three JNK isoforms, with and without a 60 min preincubation period not competing with ATP.

**Table 9:** Determination of Time Dependency of Inhibition (TDI)^a^

	JNK1 IC_50_ [nM]		JNK2 IC_50_ [nM]		JNK3 IC_50_ [nM]	
	0 min	60 min	0 min	60 min	0 min	60 min
56b	>10,000	>10,000	>10,000	>10,000	>10,000	>10,000
56d	>10,000	>10,000	>10,000	25	>10,000	40

a IC_50_ determination employing the PhosphoSens assay from AssayQuant Technologies, Inc. measured in duplicates for all three isoforms, once with 60 min of preincubation of each inhibitor without ATP and once with direct ATP supply at respective Km.

Hereby **56b** showed no TDI and accordingly no activity in this assay format for any isoform, with every IC_50_-value being above 10,000 nM. In contrast **56d** showed a significant boost in activity when the inhibitor was preincubated without ATP, with the IC_50_ being in the low two-digit nM level for JNK2 and JNK3 respectively. Moreover, **56d** showed no affinity toward JNK1 under any of the applied conditions. It has to be noted that the potency curves for **56d** showed a recognizable “hook-“effect for JNK2/3 at higher compound concentrations (Figur S5), probably due to solubility issues. In summary the results clearly indicated the time dependency of activity of inhibitor **56d**.

Subsequently **56d** was investigated in a NanoBRET-based wash-out experiment to further elaborate its mode of binding to JNK2.^29^ As shown in Figure 5A, compound **56d** remained bound to the target enzyme whereas the reversible reference inhibitor CTX-0294885 was quickly displaced by the ATP competitive fluorescent tracer. Inhibitor **56b** however, showed significantly less retention compared to its meta derivative **56d**. In total this shows the irreversibility of the mode of action for **56d**.

**Figure 5:**
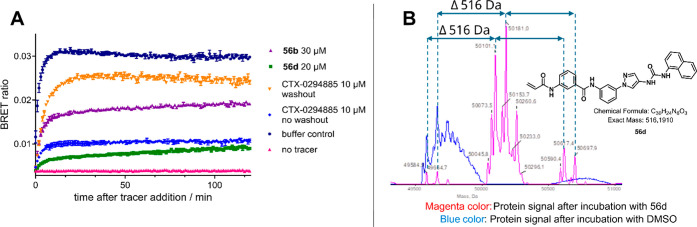
(A) NanoBRET platform (JNK2) used to verify the irreversible mode of action of **56d** (experiment performed in duplicate). After 2 h of incubation it was not possible to wash out **56d** (dark green squares) completely and to restore the BRET signal, compared to reversible inhibitor CTX0294885 (yellow triangles) and the sole vehicle (blue circles). Without BRET signal inducting tracer, no signal was detected (pink triangles). **56b** (purple triangles) did not show as high retention as **56d**. (B) Mass labeling experiment: active JNK2 was incubated once with **56d** for 4.25 h at 20 °C (magenta MS-lane) and once without (blue MS-lane). Mass shift shows almost complete monolabeling of active JNK2 by **56d** (*M*_r_ = 516).

The IPMS-experiment (Figure 5B) predominately showed single covalent labeling of JNK2 with **56d** in the observed mass shift, after a protein inhibitor incubation time of 4.25 h with a 5-fold excess of compound. These results have shown that **56d** was able to bind covalently and irreversibly to the target structure, preferably by monolabeling.

This matches nicely with our observations made in our glutathione (GSH)-stability assay (Figure S10), where we did not see relevant promiscuous inactivation of **56d**. Moreover **56d** showed a 10-fold higher stability toward thiol bearing nucleophiles compared to the FDA-approved EGFR inhibitor afatinib with an extrapolated *t*_1/2_ (**56d**) = 88.9 h compared to a *t*_1/2_ (afatinib) of 8.64 h (Figure S9).

With all the above data indicating selective, covalent and irreversible binding of **56d** to the target, we conclusively determined the *k*_inact_/*K*_I_ inactivation rate of **56d** for JNK2 and JNK3 at the respective Km ATP concentrations. With *k*_inact_/*K*_I_ values of 38,200 M^–1^·s^–1^ for JNK2 and 70,100 M^–1^·s^–1^ for JNK3 the inhibitor shows significant inactivation rates.

Reversible and covalent compounds were further investigated in NanoBRET TE assay. As depicted in Figure 6, the covalent binding entities showed not only cellular activity on JNK2, with **56d** delivering even similar activity values as in the biochemical ^33^PanQuinase assay format (EC_50_(JNK2) = 883 nM), but **56d** also demonstrated some selectivity over JNK1 (>11.3×). The para-derivative **56b** displayed activity, but was not able to differentiate activity wise between the isoforms. However, the reversible inhibitor **21h** did not show any binding affinity against JNK2 and JNK3 in this format, whereas optimized inhibitor **45c** was also able to show target engagement and some selectivity over JNK1 (>6.4×), despite being a reversible binder at JNK2 with an EC_50_ of 1555 nM.

**Figure 6:**
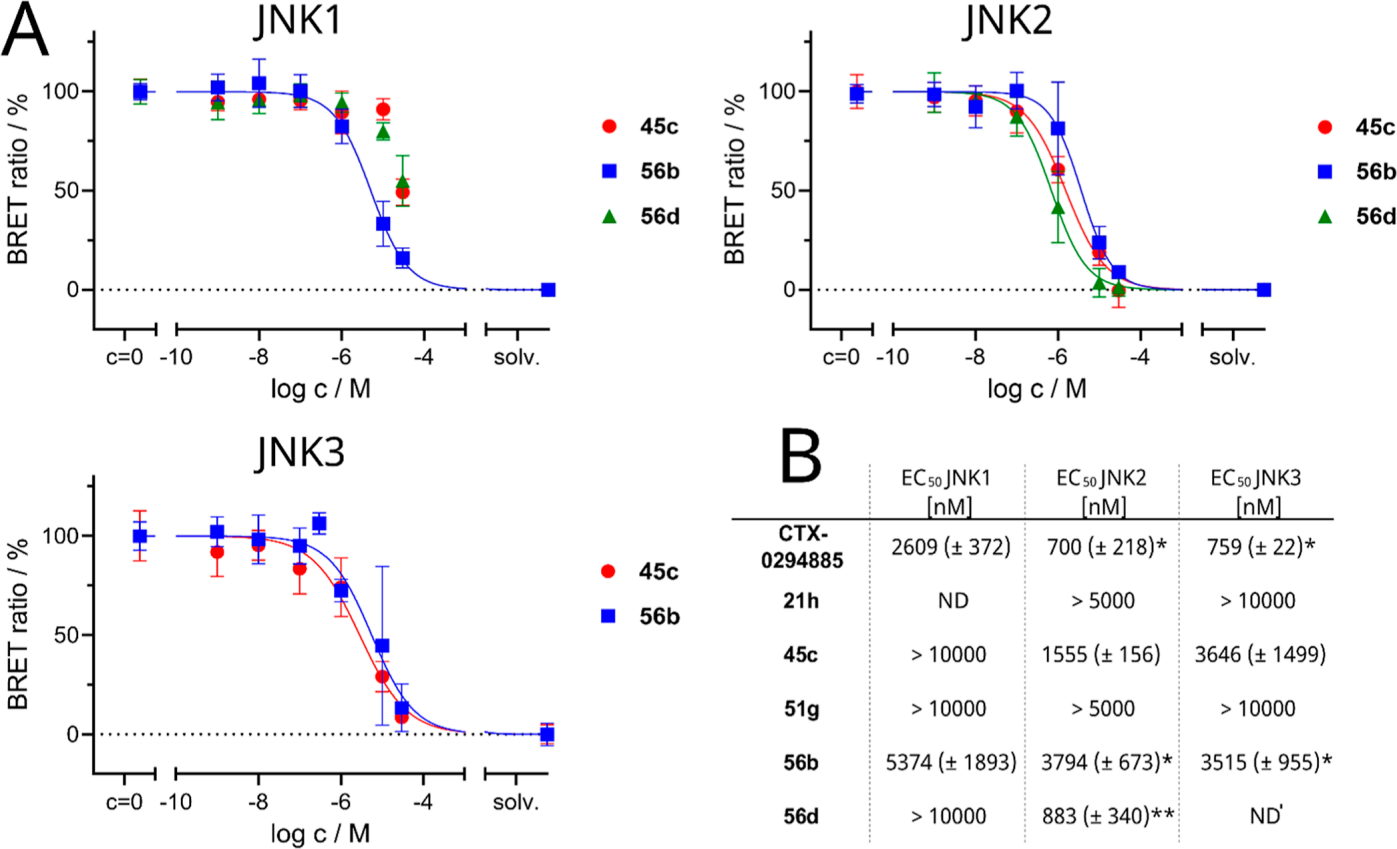
(A) Displacement curves from NanoBRET competition binding experiments for each respective JNK isoform. (B) Table with determined EC_50_. Multikinase inhibitor CTX-0294885 was used as a noncovalent reference inhibitor with already published NanoBRET EC_50_ values.^30–32^ If not further noted *n* = 2; *n = 3; **n = 4; ND = not determined; ND′ = not determinable.

To determine the selectivity profile of our covalent candidate **56d** a kinome screening was carried out in a representative panel consisting of 97 kinases (scanEDGE—KINOMEscan). The results are depicted in Figure 7. **56d** showed a clean kinome profile at a compound concentration of 500 nM, showing a percentage of control (POC) of 1.4% for JNK2 and 0.3% for JNK3 respectively. All other kinases tested showed a POC > 35%. Additionally, *K*_d_ values of **56d** were measured employing the same assay platform, which was used for the POC determination, orthogonally reconfirming affinity and JNK2/3 selectivity of **56d**.

**Figure 7:**
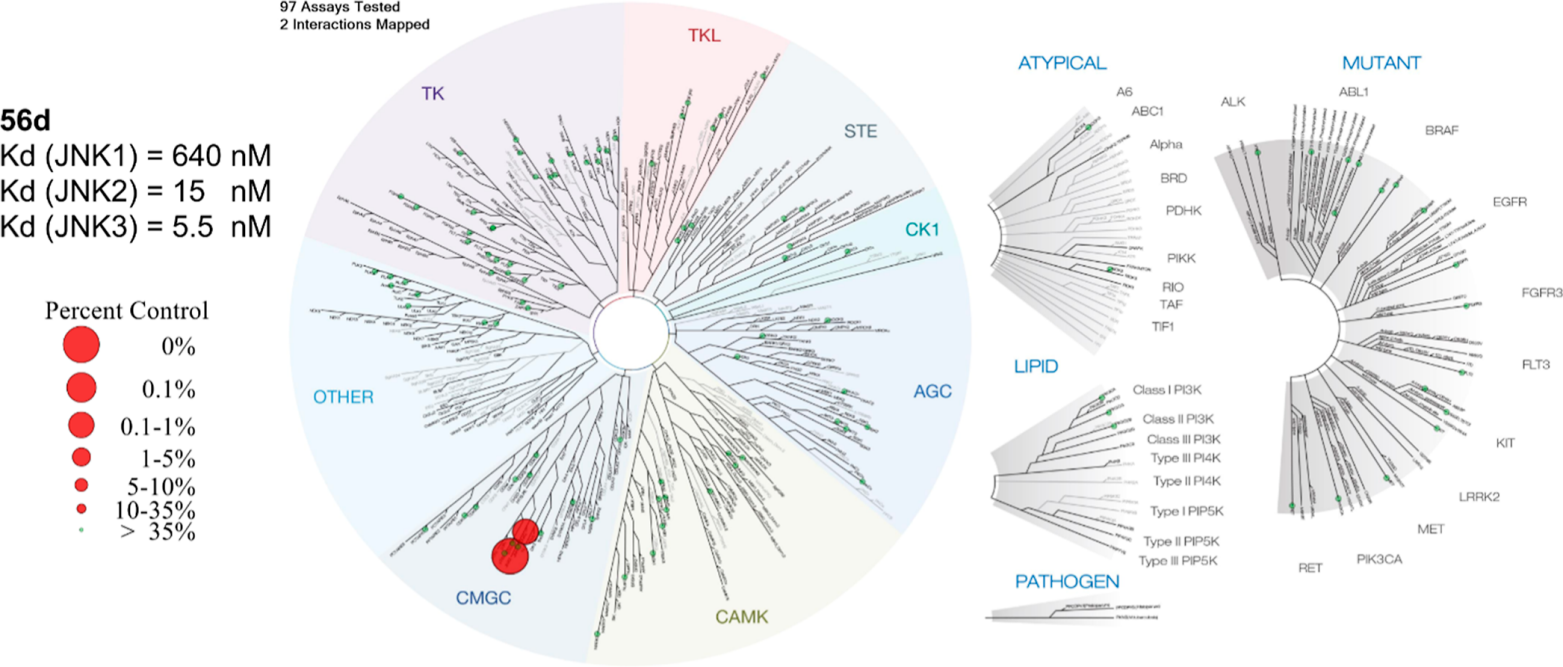
Kinome screening and *K*_d_-value determination (duplicate, 11-point dose–response) carried out by Eurofins DiscoverX employing their KINOMEscan assay format.^33^ Inhibitor **56d** only showed significant POC for JNK2 and JNK3 respectively (by default, no binding is defined as POC > 35%). Applied screening concentration was 500 nM. Image generated using TREEspot Software Tool and reprinted with permission from KINOMEscan, a division of DiscoverX Corporation, © DISCOVERX CORPORATION 2010.

### Chemistry Section

Several routes of synthesis had to be designed for the intended modifications. For modifications targeting the HR-I, we followed the route of K. Zheng et al. with minor adjustments to the second main building block **5** (Scheme 1).^17^ The initial C–N linkage between 4-nitro-1*H*-pyrazole and varieties of the central aromatic structures via copper catalyzed Ullmann-type reactions **a**) turned out to be the bottleneck of this scaffold in general (see also reaction **a**) Scheme 4/5). This coupling is difficult to realize via alternative syntheses and thus limits the effectiveness of a modular approach, with us not being able to introduce this coupling at a later stage. After the generation of this building block **2**, the synthesis splits and becomes more orthogonal. The synthesis of the piperidine building block was carried out using the *tert*-butyloxycarbonyl (Boc)-protected derivative **3**. The further conversion of 4-nitropyrazole via hydrogenation, produced building block **5**, which was then reacted with intermediate **2** via amide coupling using 1-ethyl-3-(3-(dimethylamino)­propyl)­carbodiimide (EDC) HCl and 1-hydroxybenzotriazole (HOBt). The ongoing synthesis proceeded in three linear steps: the first being a Béchamp reduction using Fe (0)-powder (**f**)), followed by a urea formation reaction using isocyanates in the second step (**g**)), and direct deprotection without isolation of the intermediate (**h**)). This yielded the corresponding target compounds presented in Table 1. The analog synthesis of methylated compounds depicted in Table 6 (**45a**–**c**) is shown in the Supporting Information (Scheme S6). In this series only methylation at the 2 and 3 position of the aromatic core were synthetically feasible, due to steric hindrances at the 4 and 6 position not allowing Ullmann coupling reactions.

**Scheme 1:**
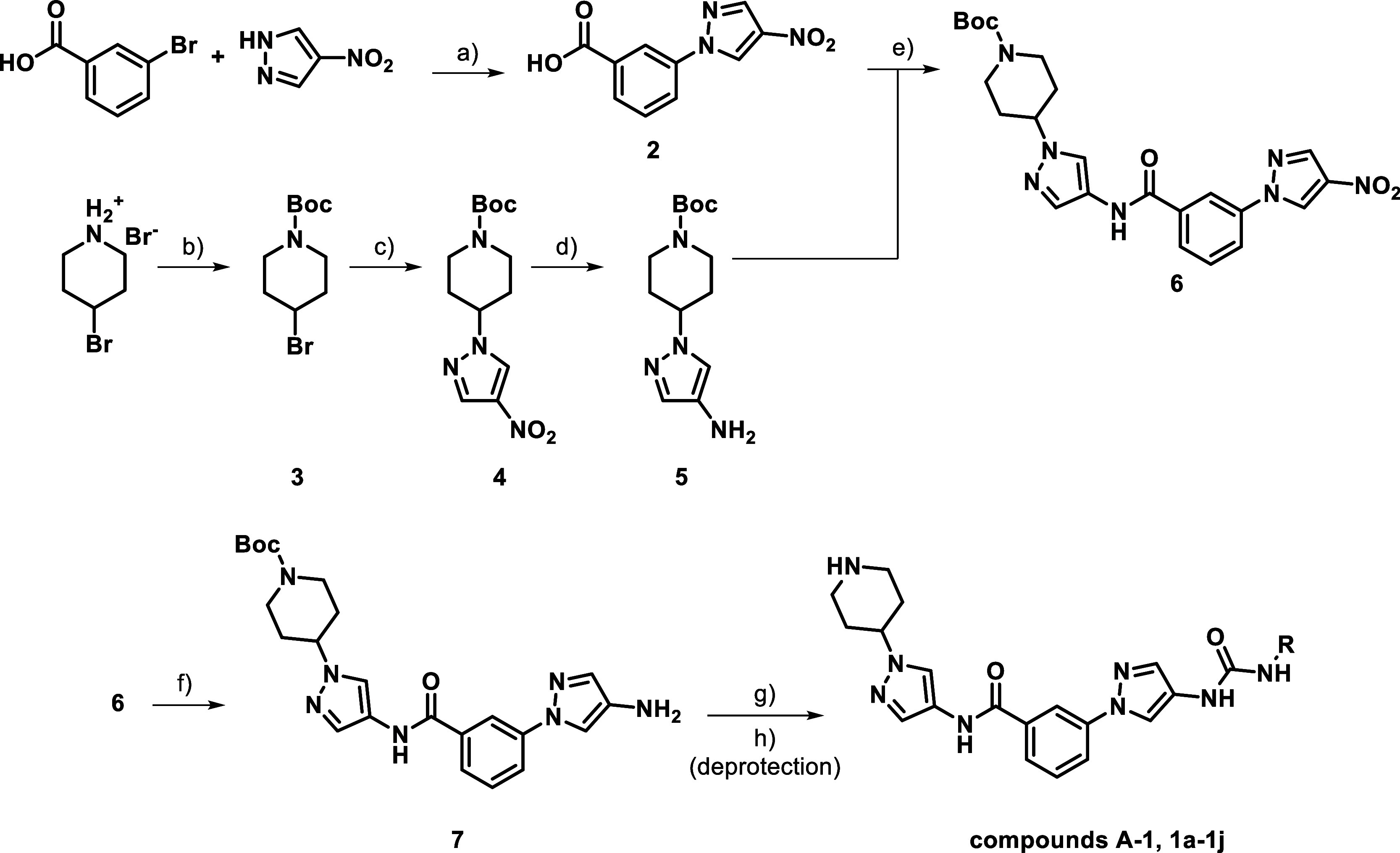
General Amide-Route^a^

a Reaction conditions and reagents exemplified by the synthesis of **1f**: **a**) 4-nitro-1*H*-pyrazole (1 equiv), 3-bromobenzoic acid (1.5 equiv), Cs_2_CO_3_ (3 equiv), Cu­(I)I (0.2 equiv), *trans*-*N*,*N*-dimethylcyclohexane-1,2-diamine (0.4 equiv), DMF (dry), 95 °C (87%); **b**) 4-bromopiperidin-1-ium bromide (1 equiv), di-*tert*-butyl dicarbonate (1 equiv) DIPEA (2 equiv), DCM (dry), room temperature (RT) (93%); **c**) **3** (1.51 equiv), 4-nitro-1*H*-pyrazole (1 equiv), K_2_CO_3_ (3 equiv), ACN (dry), 85 °C (47%); **d**) **4** (1 equiv), Pd/C (10%) (0.014 equiv), H_2_ (7 bar) via miniclave glas reactor, EtOAc, 40 °C (98%); **e**) **2** (1 equiv), **5** (1.02 equiv), EDC HCl (2 equiv), HOBt monohydrate +20 wt % water (2 equiv), DIEPA (3 equiv), DCM, RT (82%); **f**) **6** (1 equiv), Fe(0) (10 equiv), NH_4_Cl (10 equiv), EtOH/H2O (2:1), 65 °C (89%); **g**) **7** (1 equiv), 1-isocyanatonaphthalene (1 equiv), DCM, RT; **h**) product of g) (1 equiv), 1.25 M HCl (EtOH) (7.5 equiv), EtOH, 60 °C (64% (over two steps (**HCl salt**)).

For the solvent region or HR-II addressing modifications we used a strategy based on esterification (Scheme 2). The methyl ester obtained from intermediate **2** was subjected to a Béchamp reduction, leading to intermediate **18**. Further conversion with 1-isocyanatonaphthalene yielded intermediate **19**. After deprotection, amide coupling was performed using again EDC HCl and HOBt yielding the target compounds with a great variety of functionalities. It can be highlighted that we were able to perform every step except the final derivatization (**e**)) without the necessity of chromatographic purification methods allowing fast up-scaling.

**Scheme 2:**
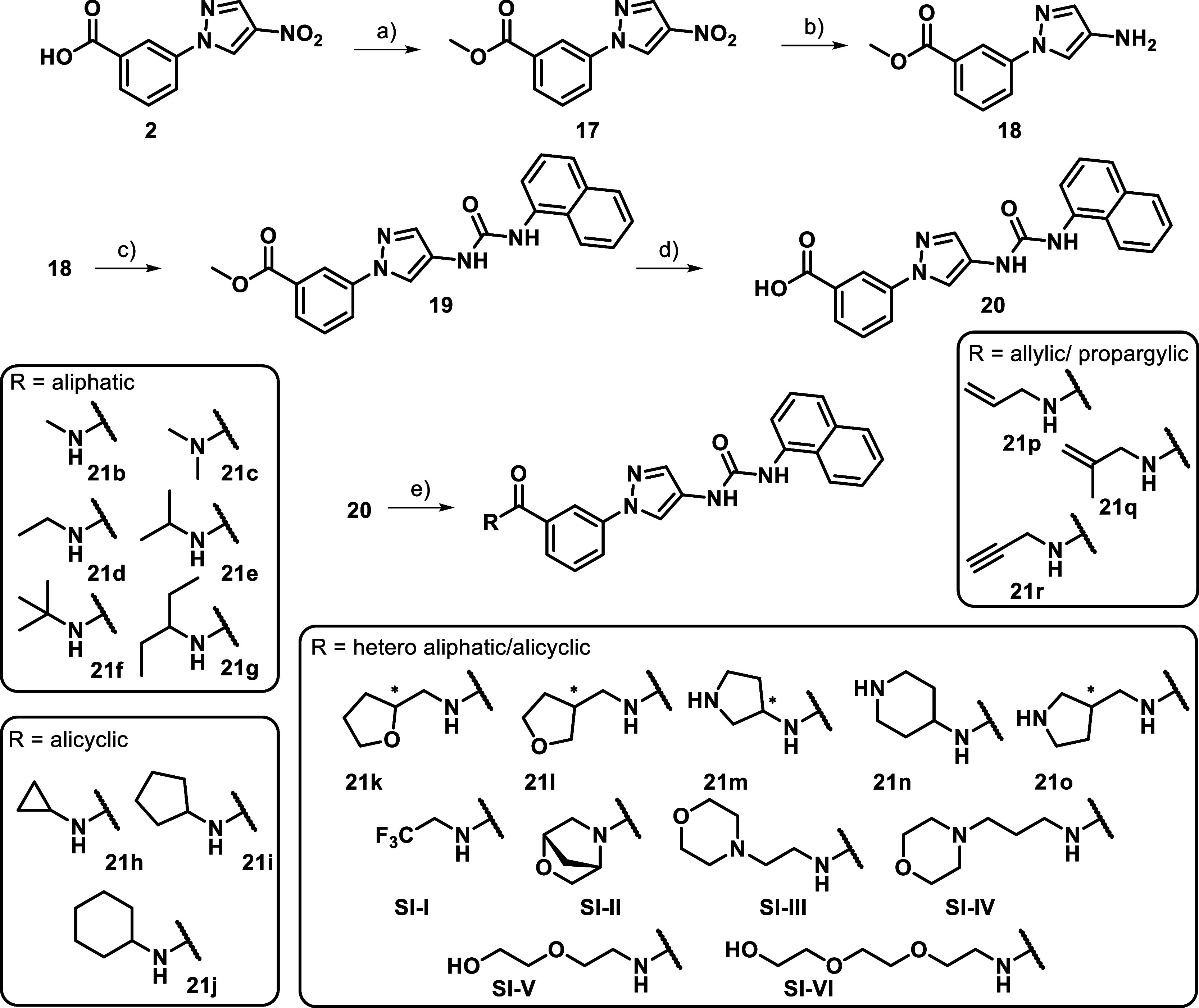
Ester-Route^a^

a Reaction conditions and reagents exemplified by the synthesis of **21h**: **a**) **2** (1 equiv), MeOH (excess), H_2_SO_4_(conc.) (cat.), 65 °C (76%); **b**) **17** (1 equiv), Fe(0) (5 equiv), NH_4_Cl (5 equiv), EtOH:H_2_O (3:1), 60–65 °C (91%); **c**) **18** (1 equiv), 1-isocyanatonaphthalene (1 equiv), DCM (dry), RT (87%); **d**) **19** (1 equiv), KOH (7.5 equiv), MeOH:H_2_O (20:1), 60 °C (86%); **e**) **20** (1 equiv), cyclopropylamine (2 equiv), EDC HCl (2 equiv), HOBt monohydrate +14 wt % water (2 equiv), DIPEA (4 equiv), DMF (dry), RT (92%).

The short set of para-substituted compounds of Table 4 were synthesized with their own “ester-route” (Scheme 3), comparable to Scheme 2. However, an Ullmann type reaction could not be carried out with para-bromo benzoic acid and therefore, as a work around, in a first step 4-fluorobenzonitrile had to be subjected to a S_N_Ar reaction with 4-nitropyrazole (**a**)) to generate intermediate **22**. A consecutive acidic nitrile hydrolysis, lead to the sought after para-substituted benzoic acid **23**. With this building block, the linear synthesis was similar to the ester route introducing a methyl protection group to form **24** followed by Béchamp reduction producing the pyrazole amine **25**. This intermediate was than subjected to a urea formation reaction using 1-isocyanatonaphthalene to generate **26** which after basic deprotection yielded the key-intermediate **27**. In contrast to amide coupling reactions performed with the key-meta-intermediate **20**, molecule **27** was coupled directly with 1-[Bis­(dimethylamino)­methylene]-1*H*-1,2,3-triazolo­[4,5-*b*]­pyridinium 3-oxid hexafluorophosphate (HATU), whereby a certain activation time of the benzoic acid also had to be maintained.

**Scheme 3:**
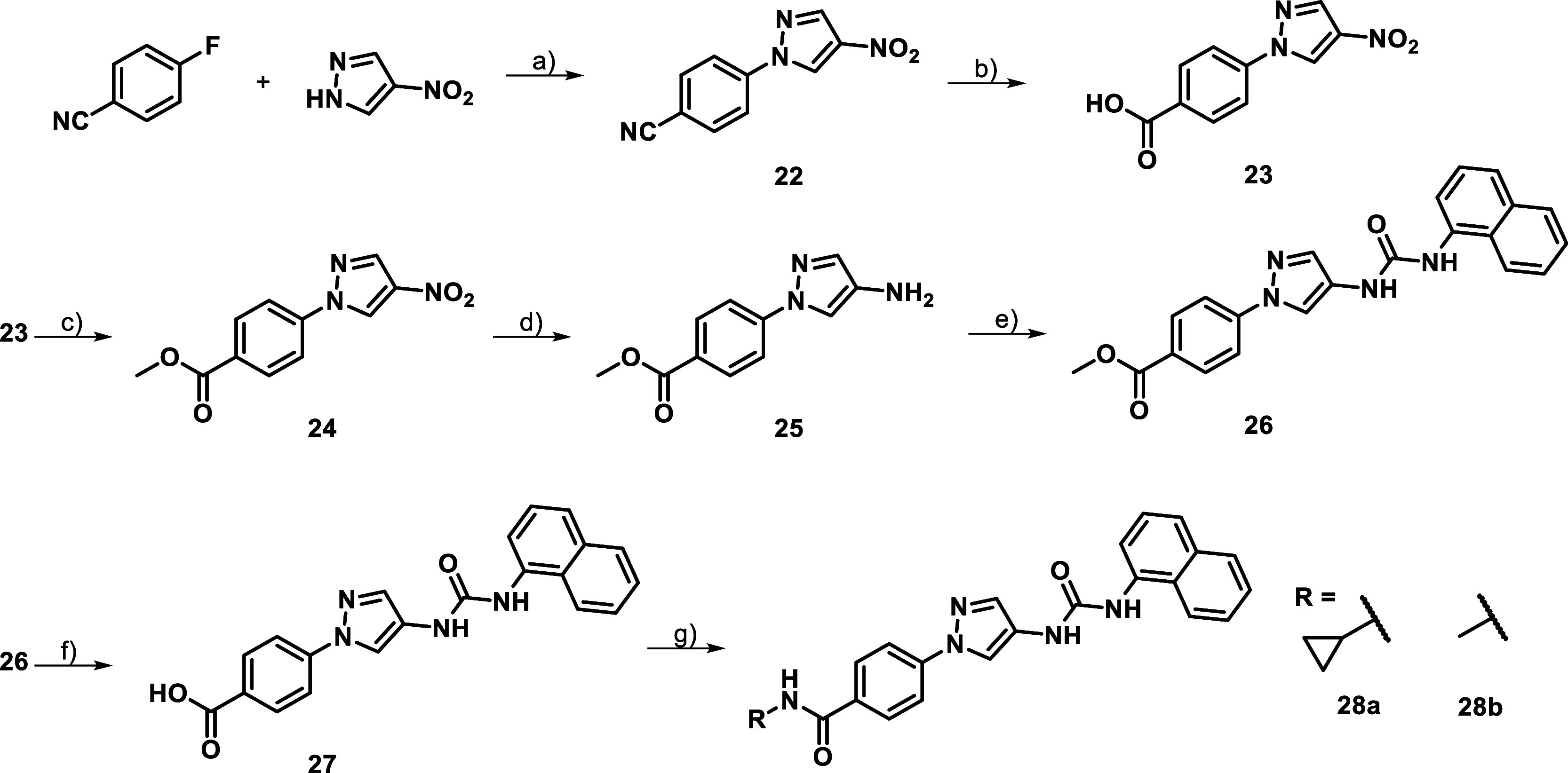
Para-Ester-Route^a^

a Reaction conditions and reagents exemplified by the synthesis of **28a**: **a**) 4-fluorobenzonitrile (1 equiv), 4-nitro-1*H*-pyrazole (1.1 equiv), K_2_CO_3_ (2.5 equiv), DMSO, 120 °C (quant.); **b**) **22** (1 equiv), 37% HCl (aq.), H_2_O; 100 °C (69%); **c**) **23** (1 equiv), H_2_SO_4_ (conc.) (cat.), MeOH, 65 °C (79%); **d**) **24** (1 equiv), Fe(0) (10 equiv), NH_4_Cl (10 equiv), EtOH:H_2_O (4:1), 60 °C (89%); **e**) **25** (1 equiv), 1-isocyanatonaphthalene (1 equiv), DCM (dry), RT (92%); **f**) **26** (1 equiv), KOH (6 equiv), MeOH:THF:H_2_O (10:10:1), 60 °C (89%); **g**) **27** (1 equiv), HATU (1.5 equiv), DIPEA (3 equiv), cyclopropylamine (2 equiv) DMF (dry), RT (95%).

The “amide first” approach of Scheme 1 was further modified to implement the subroute depicted in Scheme 4, which was used for the insertion of nitrogen atoms into the central aromatic core. Isonicotinic acid-based building block **29** could again be synthesized via Ullmann coupling, whereas the picolinic acid-derived intermediate **30** was generated by using an acid catalyzed S_N_Ar-reaction employing methanesulfonic acid (**b**)). For comparison, unsubstituted pyridinyl derivatives as depicted in Table 2 (**16b**–**d**) were easily synthesized by standard Ullmann coupling conditions (see Scheme S2). To introduce volatile methylamine (aq.) in an amide coupling reaction, the preactivation of the above-mentioned acids with carbonyl diimidazole (CDI) as a condensing agent proved to be beneficial compared to one-pot methods resulting in *N*-methylisonicotin- (**31**) and *N*-methylpicolinamides (**32**). Following standard Béchamp conditions (**d**)) and with the subsequent addition of 1-isocyanatonaphthalene, inhibitors **35a** and **36a** were realized.

**Scheme 4:**
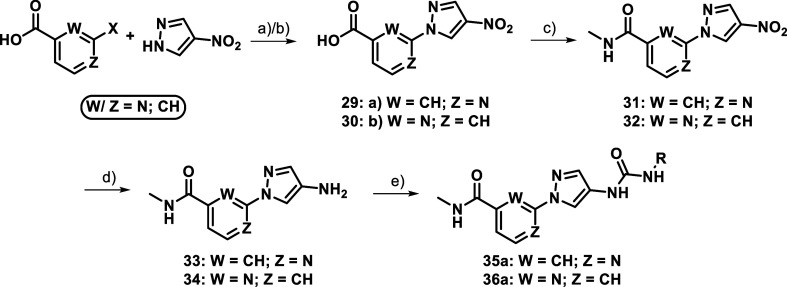
Extended Amide-Route^a^

a Reaction conditions and reagents exemplified by the synthesis of **35a**, **36a** (only step b)): **a**) 2-bromoisonicotinic acid (1 equiv), 4-nitro-1*H*-pyrazole (1 equiv), K_2_CO_3_ (3 equiv), Cu­(I)I (0.1 equiv), *trans*-*N*,*N*-dimethylcyclohexane-1,2-diamine (0.2 equiv), DMF, 95 °C (95%); **b**) 6-fluoropicolinic acid (1 equiv), 4-nitro-1*H*-pyrazole (1 equiv), methanesulfonic acid (5 equiv), Dioxane, 101 °C (66%); **c**) **29** (1 equiv), CDI (1.1 equiv), 40% methylamine (aq.) (2.5 equiv), THF (dry), RT (79%); **d**) **31** (1 equiv), Fe(0) (5 equiv), NH_4_Cl (5 equiv), EtOH:H_2_O (4:1), 60 °C (65%); **e**) **33** (1 equiv), 1-isocyanatonaphthalene (1 equiv), THF (dry), RT (70%).

For the final two series, depicted in Tables 7 and 8, compounds bearing an inverted anilide, sulfonanilide functionality were synthesized as well as *N*-alkylated anilines (Scheme 5). Like with the benzoic acid derivatives, meta-bromoaniline was coupled with 4-nitro-1*H*-pyrazole, resulting in intermediate **46**. To avoid unselective amidation in the follow-up, the free aniline was Boc-protected to allow orthogonal synthesis. The resulting product **47** was converted to **48** and subsequently to **49** using above-mentioned standard procedures. Boc deprotection using hydrochloric acid in EtOH gave the key-intermediate **50** (HCl), which was then subjected to various modifying conditions.

**Scheme 5:**
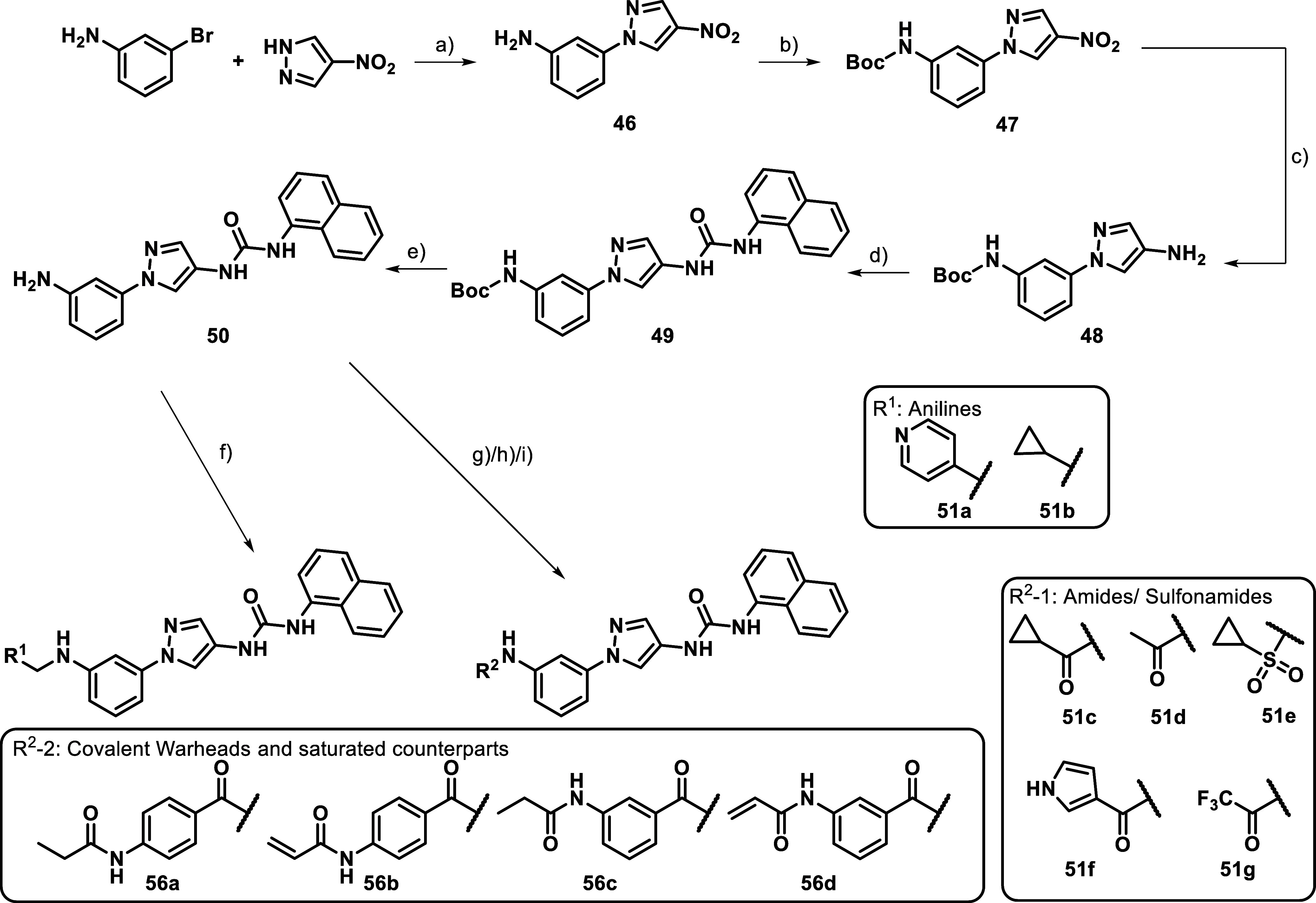
Inverted Amide Route^a^

a Reaction conditions and reagents exemplified by the synthesis of **51d**, **51a** (only step f)), **56d** (only step h)) and **51e** (only step (i)): **a**) 4-nitro-1*H*-pyrazole (1 equiv), *m*-bromoaniline (1.2 equiv), K_2_CO_3_ (3 equiv), Cu­(I)I (0.1 equiv), *trans*-*N*,*N*-dimethylcyclohexane-1,2-diamine (0.2 equiv), DMF, 100 °C (41%); **b**) **46** (1 equiv), di-*tert*-butyl dicarbonate (1 equiv), *t*-BuOH, 40–50 °C (70%); **c**) **47**(1 equiv), Fe(0) (5 equiv), NH_4_Cl (5 equiv), EtOH:H_2_O (3:1), 60 °C (76%); **d**) **48** (1 equiv), 1-isocyanatonaphthalene (1 equiv), DCM (dry), RT (83%); e) **49** (1 equiv), 1.25 M HCl (EtOH) (5 equiv), EtOH, 50–60 °C (80%, **HCl salt**); **f**) **50** (1 equiv), isonicotinaldehyde (1.05 equiv), NaCNBH_3_ (1.5 equiv), AcOH/AcONa (pH adjustment), RT (74%); **g**) **50** (1 equiv), acetyl chloride (1 equiv), Pyridine, 0 °C-RT (54%); **h**) **50** (1 equiv), HATU (2 equiv), DIPEA (5 equiv), **52** (1.2 equiv), DMF, RT, (13%); **i**) **50** (1 equiv), cyclopropanesulfonyl chloride (1 equiv), THF:Pyridine (2:1), 0 °C-RT (18%).

For the introduction of respective anilines **51a** and **51b**, **50** was subjected to reductive amination with the corresponding aldehydes and NaCNBH_3_. Amides (**51c + d**) and sulfonamides (**51e**) were likewise introduced using acid chlorides with pyridine, except when the corresponding building blocks were not available e.g. stable as acid chlorides like for inhibitors **51f** (introduced using HATU) and **51g**, which was generated using 2,2,2-trifluoroacetic anhydride as an acylating agent. The introduction of the covalent, electrophilic acrylamide warheads and their corresponding propyl amide derivatives was realized via prior synthesis of the precursors **52**–**55**. With the warhead already combined with the linking amino benzoic acid, these intermediates were then connected in an amid coupling reaction to **50** using HATU (**56a**–**d**).

## Conclusion

In this study we were able to further develop the reversible binding aminopyrazole-based scaffold to generate inhibitors, showing high preference as well as high potency for JNK2/3. Modifications have been made to improve the HR-I, HR-II or solvent orientated moieties and the central aromatic core of the initial scaffold. First, we optimized HR-I and HR-II addressing moieties, leading to JNK2/JNK3 inhibitors **21b** and **21p**, which showed excellent selectivity versus the JNK1 isoform (JNK1/JNK2 selectivity, 147- and 158-fold, respectively). In contrast to them, inhibitor **21h** showed higher activity on both targets (IC_50_(JNK2) = 73 nM; IC_50_(JNK3) = 45 nM) but a slightly lower JNK1/JNK2 selectivity ratio of 83-fold. Further modifications on the central phenyl ring resulted in the most potent dual JNK2/3 inhibitor of this series (compound **45a**, (IC_50_(JNK2) = 4 nM; IC_50_(JNK3) = 6 nM), displaying a 12-fold selectivity versus JNK1.The inversion of the solvent directed amide moiety of **21b** resulted in a complete loss of JNK1 affinity, leading to the balanced dual JNK2/3 inhibitor **51d**, which showed the highest JNK1/JNK2 selectivity ratio (>340-fold).We demonstrated that inhibitors **21b**, **21h** and **51d** are metabolically stable and that **21b** can penetrate into murine liver carcinoma cells, and show effect on the downstream substrate phosphorylation. Our reversible inhibitors showed additionally quite good PK properties.

To boost activity and selectivity within the kinome, we attached a covalent warhead onto this “ligand first” approach and therefore generated inhibitor **56d**, which proved to be a covalent JNK2/3 selective inhibitor, binding with low promiscuity and good *k*_inact/_ *K*_I_ values ((JNK2) = 38,200 M^–1^ s^–1^; (JNK3) = 70,100 M^–1^ s^–1^). Ultimately, we were able to present target engagement and even isoform selectivity in cells with our employed NanoBRET assay for our covalent lead compound **56d**. Moreover, with its clean kinome profile, **56d** fulfils almost all of the criteria of a covalently acting high quality chemical JNK2/3 selective probe.

Although more research is needed, it is indicated that each JNK has its own regulatory subrole by which the individual isoforms can be distinguished. The development of isoform selective chemical probes could provide further insight into the differences between the isoforms and how a selective blockade might be useful for further studies and clinical applications. The here presented compounds could be used as tools to further investigate the outcome of isoform selective inhibition of JNK2/3 in vitro and in vivo.

## Experimental Section

### Material and Methods

The reagents were of commercial quality and used without further purification, if not stated otherwise. Preparative flash chromatography was carried out on an Interchim PuriFlash 430 or PuriFLash XS420 (Interchim, Montluçon, FR) using Geduran Si 60–200 μm silica gel (Merck, Darmstadt, DE) for precolumns and Davisil LC60Å 20–45 μm silica gel (Grace Davison, Columbia, MD, USA) for preparative normal phase columns as well as prepacked puriFlash C18AQ separation columns for reverse phase purification. Thin layer chromatography (TLC) analyzes were carried out on TLC silica gel 60 F254 (Merck, Darmstadt, DE) and ALUGRAMXtra SIL G UV254 (Macherey Nagel, Düren, DE) silica plates. The nuclear magnetic resonance (NMR) spectra were measured on a Bruker AVANCE III HD, -HDX 400 or -HDX 700. The respective ^1^H and ^13^C NMR spectra were calibrated against the residual proton or ^13^C signals of the deuterated solvents. Signals are reported in parts per million (ppm) relative to tetramethylsilane (δ = 0 ppm). Mass spectra of TLC-MS­(ESI) measurements were measured on an Advion Expression compact mass spectrometer (Advion, Ithaca, NY, USA). Mass spectra measured using an APCI ion source, were carried out on an Advion Expression-S Compact mass spectrometer (Advion, Ithaca, NY, USA) via atmospheric solid analysis probe (ASAP). HRMS­(ESI) measurements were conducted as described by Sander et al.^34^ The purity of compounds presented was ≥ 95% if not stated otherwise. Purity was measured via high performance liquid chromatography (HPLC) on an Agilent Technologies 1100 series system (Agilent Technologies Inc., Santa Clara, CA, USA) carrying a Phenomenex Luna 5 μ (150 mm × 4.6 mm, 5 μm) reversed phase C8 separation column (Phenomenex, Torrance, CA, USA). The mobile phase consisted of phase A (MeOH) and phase B (0.01 M KH_2_PO_4_-Buffer, pH = 2.3) and elution was performed at a flow rate of 1.5 mL/min with an injection volume of 10 μL. The gradient elution started with a percentage of 40% of phase A and was increased to A = 85% over an 8 min gradient. After staying at 85% for 5 min, the system was backwashed to 40% A over 1 min after which the initial gradient was maintained for another 2 min (16 min per run in total). The purity was determined at 254 and 230 nm employing a UV/vis diode array detector (DAD). If not stated otherwise, the purity is ≥ 95% at both wavelengths. Attenuated total reflection Fourier transformation infrared (ATR-FTIR) spectra were recorded on a Cary 630 FTIR spectrometer (Agilent Technologies Inc., Santa Clara, CA, USA).

### Biochemical Assay (^33^PanQuinase)

The biochemical IC_50_ determination was performed with the radiometric ^33^PanQuinase assay by the German facility of Reaction Biology Corporation (Freiburg, DE) for all three JNK isoforms. The assays were performed at apparent Km ATP levels of each distinct isoform. The determination was performed in singlicates starting with a compound concentration of 100 μM in DMSO and ending at a concentration of 3 nM (10-point measurement; semilogarithmic dilution steps). The compounds were incubated in ScintiPlate-96 (PerkinElmer, Waltham, MA, USA) microtiter plates with protein for 60 min at 30 °C. ATF2 was used as a native substrate. For more information about the procedure contact the service provider.^24^

### TDI Determination (PhosphoSens)

The TDI and IC_50_ determination for the covalent inhibitors **56d** and **56b** was performed by AssayQuant Technologies, Inc. (Marlborough, MA, USA) employing their PhosphoSens chelation-enhanced fluorescence assay. A designed sensor peptide substrate carrying a Sulfonamido-oxine fluorophore was used as substrate.^35^ The testing consisted of a double IC_50_ determination, once with, once without a 60 min preincubation (without ATP) time period in duplicate, respectively. Protein and compounds were incubated in the presence of ATP for a total of 240 min (±60 min preincubation). It was measured in a 12 -point dose–response curve (3-fold dilution steps) starting at 10 μM inhibitor for each JNK isoform at Km ATP values. For more information about the procedure contact the service provider.

### Western Blot Assay

Adherend murine *Nras* ^*G12V*^; *Cdkn2a* ^*ARF*–/–^ hepatocellular carcinoma (HCC) cells were cultivated in 10 cm Ø Petri dishes and DMEM full growth medium (See Supporting Information) within a CO_2_-incubator at 37 °C. After the cells were trypsinized with 1 mL of 0.05% trypsin–EDTA (gibco, ThermoFischer scientific, Waltham, MA, USA) solution, they were pooled and counted via Neubauer counting chamber. 2 × 10^5^ cells were seeded within 2 mL of DMEM full growth medium in 6-well plates and cultivated overnight. Twenty-four h after seeding, 2 mL of 2× concentrated DMSO or inhibitor solutions in DMEM full growth medium were added to the cells, to generate the respective end concentrations of inhibitors for each condition. The cells were incubated for 2 h after which they were treated again with 0.286 mL of 2 M Sorbitol solution in phosphate-buffered saline (PBS) buffer (pH adjusted to 7.2) to obtain a final solution of 133 mM sorbitol. The cells were incubated for one more h, after which they were scraped into Eppendorf vials and quickly centrifuged. The supernatant was discarded and the resulting cell pellet was quickly lyzed with 200 μL of RIPA-lysis buffer. The samples were then sonicated until all visible cell particles were dissolved, while cooling permanently. The protein concentration per sample was determined with the DC Protein Assay Kit (Bio-Rad Laboratories GmbH, Feldkirchen, DE) according to the manufacturer’s protocol. Corresponding loadings were prepared using PBS- (pH = 7.4) and Laemmli-buffer while aligning protein concentration (55 μg). The loadings were incubated 5–10 min at 96 °C. Loadings were then subjected to standard protocols for SDS-PAGE using 10% acrylamide running gels and corresponding SDS-running buffer. Samples ran through the gels at 90–130 V for 1.5–2 h. The gels were then transferred onto Immobilon-P PVDF-membranes (Merck KGaA, Darmstadt, DE) in transfer buffer at 120 V for 1.5 h. The resulting blots were then blocked overnight with 5% BSA solution in TBS-T-buffer and subsequently incubated with p-c-Jun antibody (Ser73; #9164; Cell Signaling Technology, Inc., Danvers, MA USA) or Vinculin antibody (V9131; Sigma-Aldrich, Merck Corp., St. Louis, MO, USA). The resulting blots were analyzed via a ChemiDoc MP imaging system (Bio-Rad Laboratories GmbH, Feldkirchen, DE).

### Metabolic Stability in Mouse Liver Microsomes (MLM)

Pooled liver microsomes from mice (male) were purchased from Sekisui XenoTech, LLC, Kansas City, KS, USA. Metabolic stability assays were performed in the presence of an NADPH-regenerating system consisting of 5 mM glucose-6-phosphate, 5 U/mL glucose-6-phosphate dehydrogenase, and 1 mM NADP+. Liver microsomes (20 mg/mL), NADPH-regenerating system, and 4 mM MgCl_2_·6H_2_O in 0.1 M TRIS–HCl-buffer (pH 7.4) were preincubated for 5 min at 37 °C and 750 rpm on a shaker. The reaction was started by adding the preheated compounds in DMSO at concentrations of 10 mM (**21h**, **21b**, **51d**), respectively, resulting in respective final concentrations of 0.1 mM (**21h**, **21b**, **51d**). The reaction was quenched at selected time points (0, 10, 20, 30, 60, and 120 min) by pipetting 100 μL of internal standard (ketoprofen) in acetonitrile at concentrations of 0.25 mM (**21h**) and 0.2 mM (**21b**, **51d**), respectively. The samples were vortexed for 30 s and centrifuged (21,910 relative centrifugal force, 4 °C, 20 min). The supernatant was used directly for LC–MS analysis. All compound incubations were conducted at least in triplicates. Additionally, a negative control containing BSA (20 mg/mL) instead of liver microsomes and a positive control using Verapamil instead of compound were performed. A limit of 1% organic solvent during incubation was not exceeded. Sample separation and detection were performed on an Alliance 2695 Separations Module HPLC system (Waters Corporation, Milford, MA, USA) equipped with a Phenomenex Kinetex 2.6 μm XB-C18 100 Å 50 × 3 mm column (Phenomenex Inc., Torrance, CA, USA) coupled to an Alliance 2996 Photodiode Array Detector and a MICROMASS QUATTRO micro API mass spectrometer (both Waters Corporation, Milford, MA, USA) using electrospray ionization in positive mode. Mobile phase A: 90% water, 10% acetonitrile and additionally 0.1% formic acid (v/v), mobile phase B: 100% acetonitrile with additionally 0.1% formic acid (v/v). The gradient was set to 0–2.5 min 0% B, 2.5–10 min from 0 to 40% B, 10–12 min 40% B, 12–12.01 min from 40 to 0% B, 12.01–17 min 0% B (**21h**, **21b**, **51d**) at a flow rate of 0.7 mL/min. Samples were maintained at 10 °C, the column temperature was set to 20 °C with an injection volume of 5 μL. Spray, cone, extractor, and RF lens voltages were at 4 kV, 30 V, 8 and 2 V, respectively. The source and desolvation temperatures were set to 120 and 350 °C, respectively, and the desolvation gas flow was set to 750 L/h. Data analysis was conducted using MassLynx 4.1 software (Waters Corporation, Milford, MA, USA).

### Pharmacokinetic Study

All animal experiments were performed following the protocols evaluated and approved by the “Landesamt für Verbraucherschutz des Saarlandes” (Ethics Approval Number: 2.4.2.2.-06-2023).

Blood concentration/time profiles were determined by Pharmacelcus GmbH (Saarbrücken, DE). The service provider states that all experimental procedures were approved and conducted in accordance with the regulations of the local animal welfare authorities (Landesamt für Gesundheit and Verbraucherschutz, Abteilung Lebensmittel- and Veterinärwesen, Saarbrücken). The six different test compounds were tested in two separate cassettes via intravenous bolus injection in one of the lateral tail veins of C57BL/6J mice (triplicate). The compounds were administered in a vehicle-solution consisting of DMA/PEG400/Sulfobutylether-β-Cyclodextrin in water (20% w/v) (10/60/30) and the three respective compounds at a concentration of 2 mg/mL per compound at a 10 mg/kg dosage. Blood samples were taken at the following seven time points: 5 min, 15 min, 30 min, 1 h, 3 h, 8 h, 24 h post dose. The compounds were quantified in the samples via LC–MS.

### NanoBRET TE Assay (EC_50_ and Kinetic Measurement)

The NanoBRET TE intracellular assay and the target residence time format implemented on the NanoBRET platform was performed as described previously by Hoffelner et al.^29^ The final K10 tracer (Promega, Mannheim, DE) concentration was 250 nM for all of the three JNK isoforms.

### GSH Stability Assay

The GSH assay was performed as described previously by M. Schwarz et al. which is based on an adjusted version of an assay from Keeley et al.^36,37^ Experiment was performed in triplicate for each entity tested. Each sample consisted of 500 μL of a mixture of solution A + B. Solution A: 20 μL of a 10 mM compound solution in DMSO diluted with 980 μL of a 1:1 mixture of HEPES-Buffer (pH = 7.5; contents listed in Supporting Information) and ACN (HPLC grade). Solution B: 6.15 mg of GSH was dissolved in 600 μL of ACN + 400 μL of a 1 mM indoprofene (internal standard) solution in ACN and 1 mL of HEPES-Buffer. This resulted in a final concentration of 100 μM compound, 100 μM internal standard and 5 mM GSH per sample. The samples were permanently incubated at 40 °C, while measuring. The reaction and reduction of compound was monitored via decreasing compound area under the curve (AUC) observed in the corresponding HPLC chromatogram at 10 consecutive time spots: *t*_0_ = 0 h; *t*_1_ = 1.71 h; *t*_2_ = 3.42 h; *t*_3_ = 5.13 h; *t*_4_ = 6.83 h; *t*_5_ = 8.54 h; *t*_6_ = 10.25 h; *t*_7_ = 11.96 h; *t*_8_ = 13.67 h; *t*_9_ = 15.38 h. The analysis was conducted via a different HPLC setup/method: A Phenomenex Kinetex C8 100A (150 × 4.6 mm, 2.6 μm) (Phenomenex Inc. Torrance, CA, USA) separation column was used: injection volume: 5 μL; flow rate: 0.5 mL/min; mobile Phase: phase A (MeOH) and phase B (0.01 M KH_2_PO_4_-Buffer, pH = 2.3); gradient elution: 40% of phase A was increased to 95% within 9 min. This gradient was maintained for 1 min. Then the system was reequilibrated with 40% of phase A within 1 min. The gradient was then held for 5 more min (16 min per run in total). The AUC of the monitored compound was determined at 254 nm.

### Inactivation Efficiency (*k*_inact_/*K*_I_) Determination

*k*_inact_/*K*_I_ determination was performed by AssayQuant Technologies, Inc. (Marlborough, MA, USA). Inhibitor dose response curves were generated at 24 doses in duplicate using their PhosphoSens-Platform. The progression curves were subjected to a two-step global fit, to independently determine *k*_incact_ and *K*_I_ values.

### Intact Protein Mass Spectrometry

The UHPLC-system consisted of an Agilent (Waldbronn, Germany) 1290 Infinity binary pump (G4220A) and a thermostated column compartment (G1316C) with integrated 2-pos 6-port valve for online desalting of the samples. Mobile phase A (water + 0.1% (v/v) formic acid) and mobile phase B (acetonitrile + 0.1% (v/v) formic acid) were used for gradient elution on a BIOshell Protein C4 HPLC column (5 cm × 2.1 mm, 3.4 μm, 400 Å; Merck, Darmstadt, Germany). The flow rate was set to 1.0 mL/min with the following gradient settings: 0–1 min: 5% B, 1–5 min: 5–80% B, 5–6 min: 80% B, 6.01 min: 5% B, 6.01–6.5 min: 5% B. The column temperature was held at 60 °C during the whole analysis. The UHPLC-system was coupled to a TripleTOF 5600+ mass spectrometer from Sciex (Darmstadt, Germany) using a Duospray ion source in positive ionization mode and the following source and MS parameters: curtain gas (CUR): 30 psi, nebulizing gas (GS1): 50 psi, heater gas (GS2): 40 psi, ion spray floating voltage (ISVF): 5500 V, source temperature (TEM): 450 °C, collision energy (CE): 10 V, declustering potential (DP): 100 V. The acquisition was performed in the TOF mode of the mass spectrometer using a mass range from 100 to 5000 *m*/*z* with an accumulation time of 500 ms. Moreover, the IntactProteinMode-script from Sciex was used to optimize MS settings for protein analysis. The Analyst TF 1.8.1 software and the PeakView software 2.2.0 (both Sciex) were used for data acquisition and data analysis, respectively. Mass spectra were deconvoluted using the BioToolKit 2.2.0 software package. The recombinant protein-batch used in the labeling experiment was purchased from Reaction Biology Corp. (Freiburg, DE). Active, full length, untagged human JNK2 was used which was expressed in Escherichia coli (Product No. 0459-0000-1—ProQuinase JNK2). The explicit amino acid-sequence is provided by the vendor. Incubation: JNK2 protein was thawed on ice. Storage buffer consisting of 50 mM HEPES (pH = 7.5); 100 mM NaCl; 5 mM DTT + 20% glycerol was added to Protein LoBind Eppendorf tubes (Hamburg, DE). 200 μM stock solution of **56d** in DMSO (negative control only DMSO) were added as well as the protein (final concentration 0.1 mg/mL) at a ratio of five to one (final concentration of compound: 10 μM). The mixtures were incubated for 255 min at 20 °C on a shaker. After completion of the incubation the samples were frozen at −80 °C until they were analyzed via Mass spectrometry.

### Kinomscreen and *K*_d_ Determination

The selectivity within the kinome was determined for inhibitor **56d** at 500 nM employing the commercially available scanEDGE kinase assay panel (Eurofins DiscoverX LLC, San Diego, CA, USA) containing 97 different kinases tested via their KINOMEscan screening platform. Prescreening was performed on the same platform, but *K*_d_-values were recorded via 11-point dose response curves (3-fold dilution series) in duplicate.^33^

### Chemistry

For more detailed information about the synthesis please refer to the Supporting Information.

#### General Synthetic Procedure A: Ullmann-type Reaction with Pyrazoles

In an appropriate Schlenk flask 4-nitro-1*H*-pyrazole (1 equiv) was combined with the corresponding aryl halide (0.85−1.5 equiv) and dry Cs_2_CO_3_/K_2_CO_3_ (3 equiv). The components were suspended in dry DMF and the vessel was then alternately evacuated and flushed with argon for three consecutive times. In the argon countercurrent, Cu­(I)I (0.1–0.3 equiv) was introduced as well as *trans*-*N*,*N*′-dimethylcyclohexane-1,2-diamine (0.2–0.4 equiv). The reaction was then sealed and heated up to 90–100 °C until total consumption of the pyrazole or until the reaction was terminated. The reaction was carefully quenched with 10% HCl (aq.) and/or water. Precipitated product was filtered, rinsed with more 10% HCl (aq.) or water and then dried in a convection oven. If the product did not precipitate, the acidified organic phase was extracted 3× with EtOAc, dried over Na_2_SO_4_, filtered and then purified further.

#### General Synthetic Procedure B1: Amide Coupling Using EDC HCl + HOBt

The corresponding carboxylic acid (1 equiv) as well as HOBt (monohydrate) containing 14–20% water (2 equiv) and EDC HCl (2 equiv) were put into an appropriate vessel and dissolved in dry DCM, THF or DMF. Subsequently DIPEA (3–6 equiv) was added while the reaction was cooled via ice bath. After 5–20 min the amine (1–2 equiv) was added and the cooling was removed. After full conversion the reaction was quenched with water and precipitated product was filtered off. If no product was precipitating the mixture was extracted with EtOAc. The combined organic phases were dried over Na_2_SO_4_, filtered and evaporated to dryness. If required, the product was purified via flash chromatography (MeOH/DCM).

#### General Synthetic Procedure B2: Amide Coupling Using CDI

The corresponding carboxylic acid (1 equiv) as well as CDI (1.1–2 equiv) were placed in an appropriate flask and dissolved in dry THF at RT. The acid activation was now monitored after a few hours by taking a sample and combining it with isobutyl amine. The sample was then subjected to TLC-MS. If the isopentyl amine derivative was detectable and quantitatively present, the corresponding amine (2.0−3.0 equiv) was added to the reaction and the mixture was stirred overnight. The obtained product was further purified.

#### General Synthetic Procedure B3: Amide Coupling Using Acid Chlorides

The corresponding amines (1 equiv) were placed in an appropriate flask and dissolved in Pyridine or DMF (in case of DMF, bases like pyridine were added also (0.85 equiv). While cooling the reaction, the fitting acyl chloride (1 equiv) was added slowly via syringe. The reaction was stirred for 5 min, after which the reaction was allowed to slowly come up to RT. The reaction stirred at RT until full conversion. The obtained product was purified further via flash chromatography or other work-ups.

#### General Synthetic Procedure B4: Amide Coupling Using HATU

The corresponding carboxylic acid (1−1.2 equiv) as well as HATU (1.25−2 equiv) were placed in an appropriate flask and dissolved in dry DMF. DIPEA (3–5 equiv) was added to the solution. Depending on the carboxylic acid component used, the reaction was allowed to either stir for 30–60 min or overnight, or the amine component was added directly. Subsequently the corresponding amine (2 equiv) was added and reaction was continued to stir overnight or until full conversion. The mixture was then quenched with demin·H_2_O. If product precipitated, it was filtered off and dried. The product was purified further via flash chromatography if needed.

#### General Synthetic Procedure C: Reduction of *N*-Arylated-4-nitropyrazoles

The corresponding 4-nitropyrazol (1 equiv) was combined with an excess of Fe(0) powder (5–10 equiv) and NH_4_Cl (5–10 equiv). The reaction was suspended in an EtOH/H_2_O mixture (2–4:1) and heated to 60–70 °C for 45 min—3 h or until full consumption of the nitro compound. After the reaction cooled down, it was filtered over Celite, which was rinsed heavily with MeOH or EtOH. The alcoholic component was then removed and the mixture was taken up with EtOAc and washed with 0.5−2M NaOH (aq.), or saturated NaHCO_3_ (aq.) solution. After the watery phase was reextracted with EtOAc, the combined organic layers were dried over Na_2_SO_4_, filtered and evaporated to dryness.

#### General Synthetic Procedure D: Urea-formation with Isocyanates

The corresponding amine (1 equiv) was placed inside a flask and dissolved in dry DMC, Toluol, or THF. Subsequently the isocyanate (1 equiv) was added via syringe or pipet. The mixture was stirred overnight at RT or until full conversion. The reaction was either filtered directly to obtain the pure product or quenched with MeOH. After the solvent was evaporated the product was purified using flash chromatography (MeOH/DCM).

#### General Synthetic Procedure E: Boc Deprotection

The purified Boc protected amine (1 equiv) was placed inside a flask and dissolved in EtOH. An excess of 1.25 M HCl (EtOH) (5–10 equiv) was added to the solution, which was heated to 50–60 °C and stirred overnight. After evaporation of the solvent the pure, solid product was isolated and further dried in high vacuum.

### Compounds of Table 1

#### 3-(4-(3-(2-Chlorophenyl)­ureido)-1*H*-pyrazol-1-yl)-*N*-(1-(piperidin-4-yl)-1*H*-pyrazol-4-yl)­benzamide (**A-1**) (**HCl Salt**)

(96 mg; 79%) ^1^H NMR (400 MHz, DMSO): δ 10.78 (s, 1H), 9.77 (s, 1H), 9.23 (d, *J* = 9.1 Hz, 1H), 9.07–8.93 (m, 1H), 8.60 (s, 1H), 8.52 (s, 1H), 8.40 (s, 1H), 8.19 (dd, *J* = 8.3, 1.3 Hz, 1H), 8.13 (s, 1H), 8.01 (dd, *J* = 8.1, 1.3 Hz, 1H), 7.87 (d, *J* = 7.8 Hz, 1H), 7.84 (s, 1H), 7.72 (s, 1H), 7.62 (t, *J* = 7.9 Hz, 1H), 7.45 (dd, *J* = 8.0, 1.3 Hz, 1H), 7.33–7.25 (m, 1H), 7.02 (td, *J* = 8.0, 1.4 Hz, 1H), 4.56–4.47 (m, 1H), 3.41–3.34 (m, 2H), 3.11–2.99 (m, 2H), 2.22–2.13 (m, 4H); ^13^C NMR (101 MHz, DMSO): δ 162.8, 152.1, 139.8, 136.1, 135.3, 133.1, 130.6, 129.8, 129.2, 127.5, 124.8, 124.5, 123.2, 122.0, 121.7, 121.2, 120.6, 119.3, 116.5, 116.5, 55.1, 42.1, 28.6; FTIR [cm^–1^]: 3216, 3088, 2706, 2372, 1691, 1653, 1577, 1526, 1437, 1388; TLC-MS­(ESI) *m*/*z*: 505.4 [M + H]^+^; 503.5 [M – H]^−^; 539.5 [M + Cl]^−^; HRMS­(ESI) *m*/*z*: calcd for [M + H]^+^, 505.18610; found, 505.1862; HPLC *t*_ret_: 5.34 min.

#### 3-(4-(3-(2-Fluorophenyl)­ureido)-1*H*-pyrazol-1-yl)-*N*-(1-(piperidin-4-yl)-1*H*-pyrazol-4-yl)­benzamide (**1a**)

(20 mg; 37%) ^1^H NMR (400 MHz, DMSO): δ 10.64 (d, *J* = 13.4 Hz, 1H), 9.10 (d, *J* = 6.8 Hz, 1H), 8.69 (d, *J* = 2.2 Hz, 1H), 8.59 (s, 1H), 8.35 (s, 1H), 8.16 (td, *J* = 8.3, 1.4 Hz, 1H), 8.11 (s, 1H), 8.01 (dd, *J* = 8.1, 1.3 Hz, 1H), 7.84 (t, *J* = 3.8 Hz, 2H), 7.70–7.59 (m, 2H), 7.27–7.21 (m, 1H), 7.14 (t, *J* = 7.7 Hz, 1H), 7.05–6.98 (m, 1H), 4.38 (dt, *J* = 14.8, 5.4 Hz, 1H), 3.27 (d, *J* = 12.7 Hz, 2H), 2.88 (dt, *J* = 12.2, 6.2 Hz, 2H), 2.15–2.05 (m, 2H), 2.03–1.94 (m, 2H); FTIR [cm^–1^]: 3264, 3071, 2950, 2850, 1669, 1584, 1540, 1490, 1454, 1396; TLC-MS­(ESI) *m*/*z*: 489.4 [M + H]^+^; 511.5 [M + Na]^+^; 487.4 [M – H]^−^; 523.4 [M + Cl]^−^; HRMS­(ESI) *m*/*z*: calcd for [M + H]^+^, 489.21565; found, 489.2160; HPLC *t*_ret_: 4.80 min.

#### *N*-(1-(Piperidin-4-yl)-1*H*-pyrazol-4-yl)-3-(4-(3-(*o*-tolyl)­ureido)-1*H*-pyrazol-1-yl)­benzamide (**1b**) (**HCl Salt**)

(26 mg; 44%) ^1^H NMR (400 MHz, DMSO): δ 10.75 (s), 9.49 (s), 9.17 (d, *J* = 9.8 Hz), 8.93 (q, *J* = 9.6 Hz), 8.57 (s), 8.35 (d, *J* = 20.2 Hz), 8.13 (s), 8.00 (d, *J* = 7.7 Hz), 7.86 (t, *J* = 8.2 Hz), 7.80 (s), 7.71 (s), 7.61 (t, *J* = 7.5 Hz), 7.20–7.09 (m), 6.93 (t, *J* = 7.0 Hz), 4.60–4.43 (m), 3.38 (d, *J* = 9.4 Hz), 3.05 (s), 2.27 (s), 2.17 (s); ^13^C NMR (101 MHz, DMSO): δ 162.8, 152.6, 139.8, 137.6, 135.3, 133.0, 130.5, 130.1, 129.8, 127.4, 126.1, 124.9, 124.7, 122.4, 121.6, 120.7, 120.5, 119.3, 116.3, 116.1, 55.1, 42.2, 28.6, 18.1; FTIR [cm^–1^]: 3396, 3228, 3101, 1692, 1605, 1577, 1534, 1488, 1471, 1454; TLC-MS­(ESI) *m*/*z*: 485.7 [M + H]^+^; 483.8 [M – H]^−^; 519.7 [M + Cl]^−^; HRMS­(ESI) *m*/*z*: calcd for [M + H]^+^, 485.24072; found, 485.2401; HPLC *t*_ret_: 4.76 min.

#### 3-(4-(3-Cyclopentylureido)-1*H*-pyrazol-1-yl)-*N*-(1-(piperidin-4-yl)-1*H*-pyrazol-4-yl)­benzamide (**1c**) (**HCl Salt**)

(43 mg; 84%) ^1^H NMR (400 MHz, MeOD): δ 8.32 (s, 1H), 8.26 (s, 1H), 8.20 (s, 1H), 7.90 (d, *J* = 8.0 Hz, 1H), 7.83 (d, *J* = 7.5 Hz, 1H), 7.73 (s, 1H), 7.67 (s, 1H), 7.60 (t, *J* = 7.7 Hz, 1H), 4.56 (s, 1H), 4.05 (q, 1H), 3.57 (d, *J* = 11.9 Hz, 2H), 3.23 (t, *J* = 11.3 Hz, 2H), 2.39–2.21 (m, 4H), 2.02–1.91 (m, 2H), 1.80–1.56 (m, 4H), 1.53–1.41 (m, 2H); ^13^C NMR (101 MHz, MeOD): δ 166.3, 141.8, 136.8, 134.7, 132.5, 131.0, 126.4, 125.9, 123.0, 122.6, 121.7, 118.4, 57.0, 53.1, 44.2, 34.2, 30.2, 24.5; FTIR [cm^–1^]: 2944, 2867, 2719, 2499, 1642, 1583, 1559, 1491, 1388, 1281; TLC-MS­(ESI) *m*/*z*: 463.7 [M + H]^+^; 497.8 [M + Cl]^−^; HRMS­(ESI) *m*/*z*: calcd for [M + H]^+^, 463.25637; found, 463.2555; *t*_ret_: 4.29 min.

#### 3-(4-(3-Cyclopropylureido)-1*H*-pyrazol-1-yl)-*N*-(1-(piperidin-4-yl)-1*H*-pyrazol-4-yl)­benzamide (**1d**) (**TFA Salt**)

(21 mg; 35%) ^1^H NMR (400 MHz, DMSO): δ 10.68 (s, 1H), 8.72 (br s, 2H), 8.46 (d, *J* = 7.9 Hz, 2H), 8.30 (s, 1H), 8.13 (s, 1H), 7.95 (dd, *J* = 8.1, 1.3 Hz, 1H), 7.82 (d, *J* = 7.8 Hz, 1H), 7.74 (s, 1H), 7.68 (s, 1H), 7.61 (t, *J* = 7.9 Hz, 1H), 6.63 (d, *J* = 2.2 Hz, 1H), 4.57–4.47 (m, 1H), 3.45–3.38 (m, 2H), 3.07 (td, *J* = 12.3, 2.7 Hz, 2H), 2.58–2.51 (m, 1H), 2.23–2.06 (m, 4H), 0.73–0.57 (m, 2H), 0.49–0.33 (m, 2H); ^13^C NMR (101 MHz, DMSO): δ 162.9, 158.6–157.5 (m), 155.9, 139.9, 135.3, 133.2, 130.6, 129.8, 125.6, 124.5, 121.6, 120.4, 119.5, 117.5 (dd, *J* = 611.6, 311.4 Hz), 116.3, 116.1, 55.1, 42.4, 28.8, 22.5, 6.6; FTIR [cm^–1^]: 3295, 3071, 3001, 2731, 2498, 1643, 1583, 1551, 1340, 1200; TLC-MS­(ESI) *m*/*z*: 435.6 [M + H]^+^; 433.7 [M – H]^−^; HRMS­(ESI) *m*/*z*: calcd for [M + H]^+^, 435.22507; found, 435.2251; HPLC *t*_ret_: 2.61 min.

#### 3-(4-(3-Phenylureido)-1*H*-pyrazol-1-yl)-*N*-(1-(piperidin-4-yl)-1*H*-pyrazol-4-yl)­benzamide (**1e**) (**HCl Salt**)

(20 mg; 26%) ^1^H NMR (400 MHz, DMSO): δ 10.73 (s, 1H), 9.25 (s, 1H), 9.19 (d, *J* = 8.0 Hz, 1H), 9.06 (s, 1H), 8.95 (d, *J* = 8.4 Hz, 1H), 8.57 (s, 1H), 8.37 (s, 1H), 8.13 (s, 1H), 8.00 (d, *J* = 7.6 Hz, 1H), 7.85 (d, *J* = 7.4 Hz, 1H), 7.80 (s, 1H), 7.71 (s, 1H), 7.61 (t, *J* = 7.8 Hz, 1H), 7.48 (d, *J* = 7.9 Hz, 2H), 7.27 (t, *J* = 7.6 Hz, 2H), 6.95 (t, *J* = 7.2 Hz, 1H), 4.57–4.45 (m, 1H), 3.38 (d, *J* = 11.4 Hz, 2H), 3.05 (s, 2H), 2.18 (s, 4H); ^13^C NMR (101 MHz, DMSO): δ 162.8, 152.5, 139.9, 139.8, 135.3, 133.1, 130.5, 129.7, 128.7, 124.8, 124.7, 121.6, 121.6, 120.8, 120.5, 119.3, 117.9, 116.3, 55.1, 42.1, 28.6; FTIR [cm^–1^]: 3270, 3043, 2796, 2712, 2501, 1595, 1553, 1490, 1442, 1388; TLC-MS­(ESI) *m*/*z*: 471.9 [M + H]^+^; 505.8 [M + Cl]^−^; HRMS­(ESI) *m*/*z*: calcd for [M + H]^+^, 471.22507; found, 471.2241; HPLC *t*_ret_: 4.34 min.

#### 3-(4-(3-(Naphthalen-1-yl)­ureido)-1*H*-pyrazol-1-yl)-*N*-(1-(piperidin-4-yl)-1*H*-pyrazol-4-yl)­benzamide (**1f**) (**HCl Salt**)

(61 mg; 64%) ^1^H NMR (400 MHz, DMSO): δ 10.77 (s, 1H), 9.83 (s, 1H), 9.38 (s, 1H), 9.22 (d, *J* = 9.6 Hz, 1H), 9.07–8.91 (m, 1H), 8.62 (s, 1H), 8.39 (d, *J* = 9.2 Hz, 2H), 8.14 (s, 1H), 8.12–8.08 (m, 1H), 8.02 (dd, *J* = 8.1, 1.3 Hz, 1H), 7.94–7.89 (m, 1H), 7.89–7.83 (m, 2H), 7.72 (s, 1H), 7.62 (t, *J* = 8.3 Hz, 2H), 7.58–7.50 (m, 2H), 7.46 (t, *J* = 7.9 Hz, 1H), 4.59–4.45 (m, 1H), 3.41–3.34 (m, 2H), 3.10–2.99 (m, 2H), 2.25–2.10 (m, 4H); ^13^C NMR (101 MHz, DMSO): δ 162.8, 153.0, 139.9, 135.3, 134.8, 133.8, 133.0, 130.6, 129.8, 128.3, 125.9, 125.7, 125.6, 125.0, 124.8, 122.6, 121.9, 121.7, 120.6, 119.4, 116.6, 116.3, 116.2, 55.2, 42.2, 28.7; FTIR [cm^–1^]: 3234, 3086, 2751, 2548, 1690, 1653, 1547, 1488, 1386, 1340; TLC-MS­(ESI) *m*/*z*: 521.1 [M + H]^+^; 555.5 [M + Cl]^−^; HRMS­(ESI) *m*/*z*: calcd for [M + H]^+^, 521.24072; found, 521.2405; HPLC *t*_ret_: 5.55 min.

#### 3-(4-(3-(2,3-Dimethylphenyl)­ureido)-1*H*-pyrazol-1-yl)-*N*-(1-(piperidin-4-yl)-1*H*-pyrazol-4-yl)­benzamide (**1g**) (**HCl Salt**)

(64 mg; 72%). ^1^H NMR (400 MHz, DMSO): δ 10.74 (s, 1H), 9.33 (s, 1H), 9.16 (d, *J* = 9.4 Hz, 1H), 8.92 (dd, *J* = 18.9, 9.4 Hz, 1H), 8.55 (s, 1H), 8.36 (s, 1H), 8.32 (s, 1H), 8.13 (s, 1H), 7.99 (dd, *J* = 8.1, 1.0 Hz, 1H), 7.85 (d, *J* = 7.7 Hz, 1H), 7.79 (s, 1H), 7.71 (s, 1H), 7.64–7.54 (m, 2H), 7.02 (t, *J* = 7.8 Hz, 1H), 6.88 (d, *J* = 7.4 Hz, 1H), 4.55–4.45 (m, 1H), 3.38 (d, *J* = 12.4 Hz, 2H), 3.05 (s, 2H), 2.25 (s, 3H), 2.21–2.13 (m, 7H); ^13^C NMR (101 MHz, DMSO): δ 162.8, 152.9, 139.9, 137.2, 136.5, 135.3, 133.0, 130.6, 129.8, 127.3, 125.2, 125.1, 124.7, 124.7, 121.6, 120.5, 120.1, 119.3, 116.3, 116.1, 55.1, 42.2, 28.7, 20.4, 13.7; FTIR [cm^–1^]: 3243, 2938, 2800, 2712, 2492, 1653, 1584, 1540, 1395, 1278; TLC-MS­(ESI) *m*/*z*: 500.1 [M + H]^+^; 534.1 [M + Cl]^−^; HRMS­(ESI) *m*/*z*: calcd for [M + H]^+^, 499.25637; found, 499.2554; HPLC *t*_ret_: 5.16 min.

#### *N*-(1-(Piperidin-4-yl)-1*H*-pyrazol-4-yl)-3-(4-(3-(2-(trifluoromethyl)­phenyl)­ureido)-1*H*-pyrazol-1-yl)­benzamide (**1h**) (**HCl Salt**)

(81 mg; 85%) ^1^H NMR (400 MHz, DMSO): δ 10.76 (s, 1H), 9.63 (s, 1H), 9.20 (d, *J* = 9.9 Hz, 1H), 8.97 (dd, *J* = 19.6, 9.9 Hz, 1H), 8.59 (s, 1H), 8.39 (s, 1H), 8.28 (s, 1H), 8.13 (s, 1H), 8.01–7.95 (m, 2H), 7.86 (d, *J* = 7.9 Hz, 1H), 7.83 (s, 1H), 7.71 (s, 1H), 7.68 (d, *J* = 7.9 Hz, 1H), 7.63 (q, *J* = 7.6 Hz, 2H), 7.28 (t, *J* = 7.6 Hz, 1H), 4.51 (dt, *J* = 15.0, 7.6 Hz, 1H), 3.38 (d, *J* = 12.6 Hz, 2H), 3.10–2.99 (m, 2H), 2.21–2.13 (m, 4H); ^13^C NMR (101 MHz, DMSO): δ 162.8, 152.4, 139.8, 136.5 (q, *J* = 1.8 Hz), 135.3, 133.1, 132.8, 130.6, 129.7, 128.1–119.8 (m), 125.9 (q, *J* = 5.3 Hz), 125.5, 124.8, 124.5, 123.6, 121.6, 120.6, 119.9 (q, *J* = 29.0 Hz) 119.3, 116.6, 116.5, 55.1, 42.1, 28.6; FTIR [cm^–1^]: 2932, 2792, 2710, 1662, 1585, 1539, 1490, 1452, 1395, 1318; TLC-MS­(ESI) *m*/*z*: 539.4 [M + H]^+^; 537.3 [M – H]^−^; 573.3 [M + Cl]^−^; HRMS­(ESI) *m*/*z*: calcd for [M + H]^+^, 539.21246; found, 539.2126; HPLC t_ret_: 6.92 min.

#### *N*-(1-(Piperidin-4-yl)-1*H*-pyrazol-4-yl)-3-(4-(3-(3-(trifluoromethyl)­phenyl)­ureido)-1*H*-pyrazol-1-yl)­benzamide (**1i**) (**HCl Salt**)

(75 mg; 78%). ^1^H NMR (400 MHz, DMSO): δ 10.72 (s, 1H), 9.76 (s, 1H), 9.22 (s, 1H), 9.14 (d, *J* = 9.5 Hz, 1H), 8.90 (dd, *J* = 18.8, 9.3 Hz, 1H), 8.61 (s, 1H), 8.38 (s, 1H), 8.13 (s, 1H), 8.08 (s, 1H), 8.02 (dd, *J* = 8.1, 1.4 Hz, 1H), 7.86 (d, *J* = 7.8 Hz, 1H), 7.82 (s, 1H), 7.71 (s, 1H), 7.65–7.57 (m, 1H), 7.50 (t, *J* = 7.9 Hz, 1H), 7.29 (d, *J* = 7.6 Hz, 1H), 4.58–4.45 (m, 1H), 3.39 (d, *J* = 12.6 Hz, 1H), 3.13–2.98 (m, 1H), 2.23–2.11 (m, *J* = 13.7, 6.9 Hz, 2H); ^13^C NMR (101 MHz, DMSO): δ 162.8, 152.4, 140.8, 139.8, 135.3, 133.2, 130.6, 129.9, 129.8, 129.5 (q, *J* = 31.2 Hz), 124.8, 124.4, 124.2 (q, *J* = 272.3 Hz), 121.6, 121.4, 120.6, 119.4, 117.8 (dd, *J* = 7.7, 3.7 Hz),116.7, 116.3, 113.7 (q, *J* = 3.9 Hz), 55.1, 42.2, 28.7; FTIR [cm^–1^]: 3261, 3092, 2797, 2717, 2496, 1653, 1599, 1559, 1490, 1445; LC–MS­(APCI) *m*/*z*: 538.7 [M + H]^+^; HRMS­(ESI) *m*/*z*: calcd for [M + H]^+^, 539.21246; found, 539.2129; HPLC t_ret_: 6.29 min.

#### *N*-(1-(Piperidin-4-yl)-1*H*-pyrazol-4-yl)-3-(4-(3-(4-(trifluoromethyl)­phenyl)­ureido)-1*H*-pyrazol-1-yl)­benzamide (**1j**) (**HCl Salt**)

(27 mg; 28%). ^1^H NMR (400 MHz, DMSO): δ 10.73 (s, 1H), 9.70 (s, 1H), 9.17 (s, 1H), 9.02 (d, *J* = 9.0 Hz, 1H), 8.79 (d, *J* = 9.2 Hz, 1H), 8.60 (s, 1H), 8.37 (s, 1H), 8.14 (s, 1H), 8.02 (d, *J* = 7.8 Hz, 1H), 7.85 (d, *J* = 11.6 Hz, 2H), 7.72–7.67 (m, 3H), 7.66–7.60 (m, 3H), 4.56–4.47 (m, 1H), 3.40 (d, *J* = 12.1 Hz, 2H), 3.12–2.98 (m, 2H), 2.23–2.08 (m, 4H); ^13^C NMR (101 MHz, DMSO): δ 162.8, 152.2, 143.7, 139.8, 135.3, 133.3, 130.6, 129.8, 126.1 (dd, *J* = 7.4, 3.6 Hz), 124.9, 124.6 (dd, *J* = 541.7, 270.8 Hz), 124.4, 121.6, 121.6 (q, *J* = 31.8 Hz).120.6, 119.4, 117.6, 116.7, 116.4, 55.1, 42.3, 28.7; FTIR [cm^–1^]: 3242, 3205, 3092, 2843, 2740, 2549, 1706, 1647, 1609, 1583; TLC-MS­(ESI) *m*/*z*: 539.2 [M + H]^+^; 537.5 [M – H]^−^; 573.6 [M + Cl]^−^; HRMS­(ESI) *m*/*z*: calcd for [M + H]^+^, 539.21246; found, 539.2117; HPLC t_ret_: 6.23 min.

### Compounds of Table 2

#### 1-(Naphthalen-1-yl)-3-(1-phenyl-1*H*-pyrazol-4-yl)­urea (**16a**)

(77 mg; 37%) ^1^H NMR (400 MHz, DMSO): δ 8.95 (s, 1H), 8.84 (s, 1H), 8.48 (s, 1H), 8.12 (d, *J* = 8.4 Hz, 1H), 8.04 (d, *J* = 7.5 Hz, 1H), 7.93 (d, *J* = 7.6 Hz, 1H), 7.85 (s, 1H), 7.83–7.79 (m, 2H), 7.64 (d, *J* = 8.2 Hz, 1H), 7.62–7.57 (m, 1H), 7.57–7.52 (m, 1H), 7.51–7.45 (m, 3H), 7.28 (t, *J* = 7.4 Hz, 1H); ^13^C NMR (101 MHz, DMSO): δ 152.8, 139.8, 134.5, 133.7, 132.9, 129.5, 128.4, 126.0, 125.9, 125.7, 125.7, 124.5, 122.9, 121.4, 117.8, 117.3, 116.3; FTIR [cm^–1^]: 3267, 3097, 3050, 1638, 1584, 1501, 1415, 1382, 1344, 1247; TLC-MS­(ESI) *m*/*z*: 351.2 [M + Na]^+^; 327.0 [M – H]^−^; 363.1 [M + Cl]^−^; HRMS­(ESI) *m*/*z*: calcd for [M + H]^+^, 329.13961; found, 329.1410; HPLC *t*_ret_: 8.03 min.

#### 1-(Naphthalen-1-yl)-3-(1-(pyridin-2-yl)-1*H*-pyrazol-4-yl)­urea (**16b**)

(128 mg; 62%). ^1^H NMR (400 MHz, DMSO): δ 9.02 (s, 1H), 8.87 (s, 1H), 8.72 (s, 1H), 8.46 (dd, *J* = 4.8, 0.7 Hz, 1H), 8.12 (d, *J* = 8.3 Hz, 1H), 8.03–7.89 (m, 5H), 7.65 (d, *J* = 8.2 Hz, 1H), 7.62–7.46 (m, 3H), 7.31 (ddd, *J* = 6.8, 4.8, 0.9 Hz, 1H); ^13^C NMR (101 MHz, DMSO): δ 152.8, 151.0, 148.3, 139.3, 134.4, 134.1, 133.7, 128.4, 126.2, 125.9, 125.9, 125.7, 124.7, 123.1, 121.5, 121.4, 117.7, 115.1, 111.3; FTIR [cm^–1^]: 3273, 3098, 3046, 1638, 1593, 1570, 1471, 1456, 1395, 1369; TLC-MS­(ESI) *m*/*z*: 352.2 [M + Na]^+^; 384.2 [M + Na + MeOH]^+^; 328.1 [M – H]^−^; 364.1 [M + Cl]^−^; HRMS­(ESI) *m*/*z*: calcd for [M + H]^+^, 330.13486; found, 330.1346; HPLC *t*_ret_: 7.48 min.

#### 1-(Naphthalen-1-yl)-3-(1-(pyridin-3-yl)-1*H*-pyrazol-4-yl)­urea (**16c**)

(116 mg; 56%) ^1^H NMR (400 MHz, DMSO): δ 9.09 (s, 1H), 9.00 (s, 1H), 8.85 (s, 1H), 8.58 (s, 1H), 8.49 (d, *J* = 3.4 Hz, 1H), 8.22 (d, *J* = 7.7 Hz, 1H), 8.12 (d, *J* = 8.2 Hz, 1H), 8.03 (d, *J* = 7.4 Hz, 1H), 7.98–7.87 (m, 2H), 7.67–7.46 (m, 5H); ^13^C NMR (101 MHz, DMSO): δ 152.8, 146.7, 139.3, 136.1, 134.4, 133.9, 133.7, 128.4, 126.0, 125.9, 125.9, 125.7, 125.1, 125.0, 124.2, 123.0, 121.4, 117.4, 116.5; FTIR [cm^–1^]: 3263, 3109, 3040, 1638, 1579, 1554, 1481, 1384, 1345, 1249; TLC-MS­(ESI) *m*/*z*: 352.1 [M + Na]^+^; 384.2 [M + Na + MeOH]^+^; 328.1 [M – H]^−^; 364.1 [M + Cl]^−^; HRMS­(ESI) *m*/*z*: calcd for [M + H]^+^, 330.13486; found, 330.1350; HPLC *t*_ret_: 6.62 min.

#### 1-(Naphthalen-1-yl)-3-(1-(pyridin-4-yl)-1*H*-pyrazol-4-yl)­urea (**16d**)

(112 mg; 55%). ^1^H NMR (400 MHz, DMSO): δ 9.05 (s, 1H), 8.89 (s, 1H), 8.65 (s, 1H), 8.63–8.57 (m, 2H), 8.11 (d, *J* = 8.3 Hz, 1H), 8.02 (d, *J* = 7.5 Hz, 1H), 7.98 (s, 1H), 7.94 (d, *J* = 7.8 Hz, 1H), 7.86–7.80 (m, 2H), 7.65 (d, *J* = 8.1 Hz, 1H), 7.62–7.53 (m, 2H), 7.48 (t, *J* = 7.9 Hz, 1H); ^13^C NMR (101 MHz, DMSO): δ 152.8, 151.1, 145.5, 134.9, 134.3, 133.8, 128.4, 126.1, 126.0, 125.9, 125.8, 125.5, 123.1, 121.4, 117.5, 116.1, 111.6; FTIR [cm^–1^]: 3271, 3109, 3063, 1641, 1591, 1559, 1502, 1411, 1370, 1345; TLC-MS­(ESI) *m*/*z*: 352.3 [M + Na]^+^; 384.3 [M + Na + MeOH]+; 328.2 [M – H]^−^; 364.2 [M + Cl]^−^; HRMS­(ESI) *m*/*z*: calcd for [M + H]^+^, 330.13486; found, 330.1356; HPLC *t*_ret_: 5.25 min.

### Compounds of Table 3

#### 3-(4-(3-(Naphthalen-1-yl)­ureido)-1*H*-pyrazol-1-yl)­benzoic Acid (**20**)

(2500 mg; 86%) ^1^H NMR (400 MHz, DMSO): δ 13.23 (br s, 1H), 8.97 (s, 1H), 8.87 (s, 1H), 8.56 (s, 1H), 8.34 (s, 1H), 8.12 (d, *J* = 8.3 Hz, 1H), 8.09–7.99 (m, 2H), 7.96–7.87 (m, 2H), 7.84 (d, *J* = 7.6 Hz, 1H), 7.68–7.51 (m, *J* = 21.1, 15.2, 7.5 Hz, 4H), 7.48 (t, *J* = 7.9 Hz, 1H); ^13^C NMR (101 MHz, DMSO): δ 166.8, 152.9, 139.9, 134.4, 133.8, 133.4, 132.2, 130.0, 128.4, 126.4, 126.1, 125.9, 125.9, 125.7, 124.9, 123.0, 121.8, 121.4, 118.3, 117.5, 116.4; FTIR [cm^–1^]: 3267, 3110, 3068, 3052, 1702, 1685, 1636, 1584, 1555, 1491; TLC-MS­(ESI) *m*/*z*: 371.1 [M – H]^−^; HRMS­(ESI) *m*/*z*: calcd for [M + H]^+^, 373.12944; found, 373.1300; HPLC *t*_ret_: 7.31 min.

#### 3-(4-(3-(Naphthalen-1-yl)­ureido)-1*H*-pyrazol-1-yl)­benzamide (**21a**)

(33 mg, 22%) ^1^H NMR (400 MHz, DMSO): δ 8.98 (s, 1H), 8.86 (s, 1H), 8.59 (s, 1H), 8.27 (s, 1H), 8.17 (s, 1H), 8.12 (d, *J* = 8.3 Hz, 1H), 8.03 (d, *J* = 7.5 Hz, 1H), 8.00–7.91 (m, 2H), 7.86 (s, 1H), 7.77 (d, *J* = 7.5 Hz, 1H), 7.65 (d, *J* = 8.1 Hz, 1H), 7.63–7.52 (m, 3H), 7.53–7.37 (m, 2H); ^13^C NMR (101 MHz, DMSO): δ 167.1, 152.8, 139.8, 135.6, 134.4, 133.7, 133.1, 129.5, 128.4, 126.0, 125.9, 125.9, 125.7, 124.7, 123.0, 121.4, 120.4, 117.3, 116.5, 116.4; FTIR [cm^–1^]: 3301, 3167, 3108, 1643, 1627, 1610, 1592, 1583, 1547, 1499; TLC-MS­(ESI) *m*/*z*: 393.8 [M + Na]^+^; 425.7 [M + Na + MeOH]^+^; 370.0 [M – H]^−^; 405.9 [M + Cl]^−^; HRMS­(ESI) *m*/*z*: calcd for [M + H]^+^, 372.14542; found, 372.1452; HPLC *t*_ret_: 6.45 min.

#### *N*-Methyl-3-(4-(3-(naphthalen-1-yl)­ureido)-1*H*-pyrazol-1-yl)­benzamide (**21b**)

(346 mg; 84%) ^1^H NMR (400 MHz, DMSO): δ 8.98 (s, 1H), 8.86 (s, 1H), 8.65–8.60 (m, *J* = 4.5 Hz, 1H), 8.59 (s, 1H), 8.24 (t, *J* = 1.7 Hz, 1H), 8.13 (d, *J* = 8.4 Hz, 1H), 8.06–8.02 (m, *J* = 7.7, 0.7 Hz, 1H), 7.98–7.91 (m, *J* = 11.4, 5.7, 0.9 Hz, 2H), 7.86 (s, 1H), 7.74 (d, *J* = 7.8 Hz, 1H), 7.65 (d, *J* = 8.2 Hz, 1H), 7.62–7.52 (m, 3H), 7.48 (t, *J* = 7.9 Hz, 1H), 2.83 (d, *J* = 4.5 Hz, 3H); ^13^C NMR (101 MHz, DMSO): δ 165.8, 152.8, 139.8, 135.8, 134.4, 133.7, 133.1, 129.6, 128.4, 126.0, 125.9, 125.8, 125.7, 124.7, 124.4, 123.0, 121.4, 120.2, 117.4, 116.4, 116.1, 26.3; FTIR [cm^–1^]: 3288, 3231, 3081, 2948, 1647, 1584, 1546, 1482, 1394, 1385; TLC-MS­(ESI) *m*/*z*: 408.0 [M + Na]^+^; 384.1 [M – H]^−^; 420.0 [M + Cl]^−^; HRMS­(ESI) *m*/*z*: calcd for [M + H]^+^, 386.16107; found, 386.1624; HPLC *t*_ret_: 6.71 min.

#### *N*,*N*-Dimethyl-3-(4-(3-(naphthalen-1-yl)­ureido)-1*H*-pyrazol-1-yl)­benzamide (**21c**)

(23 mg; 31%) ^1^H NMR (400 MHz, DMSO): δ 8.97 (s, 1H), 8.86 (s, 1H), 8.54 (s, 1H), 8.12 (d, *J* = 8.2 Hz, 1H), 8.03 (d, *J* = 7.5 Hz, 1H), 7.93 (d, *J* = 7.8 Hz, 1H), 7.91–7.86 (m, 2H), 7.83 (s, 1H), 7.64 (d, *J* = 8.1 Hz, 1H), 7.62–7.51 (m, 3H), 7.48 (t, *J* = 7.9 Hz, 1H), 7.28 (d, *J* = 7.5 Hz, 1H), 3.01 (s, 3H), 2.94 (s, 3H); ^13^C NMR (101 MHz, DMSO): δ 169.3, 152.8, 139.6, 137.9, 134.4, 133.7, 133.3, 129.6, 128.4, 126.0, 125.9, 125.9, 125.7, 124.7, 123.9, 122.9, 121.4, 118.3, 117.3, 116.4, 116.0, 34.7; FTIR [cm^–1^]: 3295, 3046, 2924, 1606, 1581, 1549, 1482, 1388, 1341, 1252; TLC-MS­(ESI) *m*/*z*: 422.0 [M + Na]^+^; 398.1 [M – H]^−^; 434.1 [M + Cl]^−^; HRMS­(ESI) *m*/*z*: calcd for [M + H]^+^, 400.17672; found, 400.1776; HPLC *t*_ret_: 6.96 min.

#### *N*-Ethyl-3-(4-(3-(naphthalen-1-yl)­ureido)-1*H*-pyrazol-1-yl)­benzamide (**21d**)

(45 mg; 60%) ^1^H NMR (400 MHz, DMSO): δ 8.99 (s, 1H), 8.87 (s, 1H), 8.67 (s, 1H), 8.59 (s, 1H), 8.23 (s, 1H), 8.12 (d, *J* = 8.2 Hz, 1H), 8.04 (d, *J* = 7.4 Hz, 1H), 7.95 (t, *J* = 8.9 Hz, 2H), 7.86 (s, 1H), 7.75 (d, *J* = 7.4 Hz, 1H), 7.65 (d, *J* = 8.0 Hz, 1H), 7.62–7.52 (m, 3H), 7.48 (t, *J* = 7.8 Hz, 1H), 3.40–3.26 (m, *J* = 12.4 Hz, 2H), 1.16 (t, *J* = 7.0 Hz, 3H); ^13^C NMR (101 MHz, DMSO): δ 165.2, 152.8, 139.8, 136.0, 134.4, 133.7, 133.1, 129.6, 128.4, 126.0, 125.9, 125.9, 125.7, 124.7, 124.5, 123.0, 121.4, 120.2, 117.4, 116.4, 116.2, 34.1, 14.8; FTIR [cm^–1^]: 3272, 2971, 2932, 2875, 1639, 1586, 1540, 1490, 1385, 1311; TLC-MS­(ESI) *m*/*z*: 422.0 [M + Na]^+^; 398.1 [M – H]^−^; 434.1 [M + Cl]^−^; HRMS­(ESI) *m*/*z*: calcd for [M + H]^+^, 400.17672; found, 400.1776; HPLC *t*_ret_: 7.36 min.

#### *N*-Isopropyl-3-(4-(3-(naphthalen-1-yl)­ureido)-1*H*-pyrazol-1-yl)­benzamide (**21e**)

(52 mg; 67%) ^1^H NMR (400 MHz, DMSO): δ 9.00 (s, 1H), 8.88 (s, 1H), 8.59 (s, 1H), 8.43 (d, *J* = 7.6 Hz, 1H), 8.22 (s, 1H), 8.12 (d, *J* = 8.3 Hz, 1H), 8.03 (d, *J* = 7.4 Hz, 1H), 7.98–7.91 (m, 2H), 7.86 (s, 1H), 7.75 (d, *J* = 7.7 Hz, 1H), 7.65 (d, *J* = 8.1 Hz, 1H), 7.62–7.52 (m, 3H), 7.48 (t, *J* = 7.9 Hz, 1H), 4.14 (dh, *J* = 13.0, 6.4 Hz, 1H), 1.20 (d, *J* = 6.6 Hz, 6H); ^13^C NMR (101 MHz, DMSO): δ 164.6, 152.8, 139.7, 136.1, 134.4, 133.7, 133.1, 129.5, 128.4, 126.0, 125.9, 125.9, 125.7, 124.7, 124.7, 123.0, 121.4, 120.2, 117.4, 116.5, 116.3, 41.1, 22.3; FTIR [cm^–1^]: 3288, 3241, 3110, 3060, 2968, 2934, 2873, 1646, 1633, 1585; TLC-MS­(ESI) *m*/*z*: 436.0 [M + Na]^+^; 412.2 [M – H]^−^; 448.1 [M + Cl]^−^; HRMS­(ESI) *m*/*z*: calcd for [M + H]^+^, 414.19238; found, 414.1936; HPLC *t*_ret_: 7.82 min.

#### *N*-(*tert*-Butyl)-3-(4-(3-(naphthalen-1-yl)­ureido)-1*H*-pyrazol-1-yl)­benzamide (**21f**)

(69 mg; 86%) ^1^H NMR (400 MHz, DMSO): δ 8.99 (s, 1H), 8.87 (s, 1H), 8.58 (s, 1H), 8.17–8.11 (m, 2H), 8.03 (d, *J* = 7.1 Hz, 1H), 7.99–7.90 (m, 3H), 7.86 (s, 1H), 7.73–7.68 (m, 1H), 7.65 (d, *J* = 8.2 Hz, 1H), 7.62–7.51 (m, 3H), 7.48 (t, *J* = 7.9 Hz, 1H), 1.41 (s, 9H); ^13^C NMR (101 MHz, DMSO): δ 165.6, 152.8, 139.6, 137.2, 134.4, 133.7, 133.1, 129.3, 128.4, 126.0, 125.9, 125.9, 125.7, 124.7, 124.7, 123.0, 121.4, 120.0, 117.4, 116.6, 116.6, 50.9, 28.6; FTIR [cm^–1^]: 3405, 3258, 2963, 1694, 1633, 1550, 1493, 1444, 1400, 1342; TLC-MS­(ESI) *m*/*z*: 450.1 [M + Na]^+^; 426.2 [M – H]^−^; 462.2 [M + Cl]^−^; HRMS­(ESI) *m*/*z*: calcd for [M + H]^+^, 428.20803; found, 428.2104; HPLC *t*_ret_: 8.37 min.

#### 3-(4-(3-(Naphthalen-1-yl)­ureido)-1*H*-pyrazol-1-yl)-*N*-(pentan-3-yl)­benzamide (**21g**)

(69 mg; 97%) ^1^H NMR (400 MHz, DMSO): δ 8.99 (s, 1H), 8.87 (s, 1H), 8.59 (s, 1H), 8.32–8.18 (m, 2H), 8.12 (d, *J* = 8.1 Hz, 1H), 8.03 (d, *J* = 7.3 Hz, 1H), 7.95 (t, *J* = 7.4 Hz, 2H), 7.87 (s, 1H), 7.76 (d, *J* = 7.3 Hz, 1H), 7.69–7.52 (m, 4H), 7.48 (t, *J* = 7.7 Hz, 1H), 3.89–3.73 (m, 1H), 1.65–1.44 (m, 4H), 0.88 (t, *J* = 7.0 Hz, 6H); ^13^C NMR (101 MHz, DMSO): δ 165.5, 152.8, 139.7, 136.3, 134.4, 133.7, 133.1, 129.5, 128.4, 126.0, 125.9, 125.9, 125.7, 124.7, 123.0, 121.4, 120.2, 117.4, 116.5, 116.3, 52.2, 27.00, 10.7; FTIR [cm^–1^]: 3329, 3276, 3229, 3073, 2960, 2930, 2873, 1644, 1629, 1551M; TLC-MS­(ESI) *m*/*z*: 442.9 [M + H]^+^; 463.8 [M + Na]^+^; 439.9 [M – H]^−^; 475.8 [M + Cl]^−^; HRMS­(ESI) *m*/*z*: calcd for [M + H]^+^, 442.22368; found, 442.2252; HPLC *t*_ret_: 8.64 min.

#### *N*-Cyclopropyl-3-(4-(3-(naphthalen-1-yl)­ureido)-1*H*-pyrazol-1-yl)­benzamide (**21h**)

(61 mg; 92%) ^1^H NMR (400 MHz, DMSO): δ 8.98 (s, 1H), 8.86 (s, 1H), 8.64 (d, *J* = 3.1 Hz, 1H), 8.58 (s, 1H), 8.20 (s, 1H), 8.12 (d, *J* = 8.3 Hz, 1H), 8.03 (d, *J* = 7.5 Hz, 1H), 7.95 (t, *J* = 7.8 Hz, 2H), 7.86 (s, 1H), 7.72 (d, *J* = 7.6 Hz, 1H), 7.67–7.52 (m, 4H), 7.48 (t, *J* = 7.8 Hz, 1H), 2.93–2.82 (m, 1H), 0.77–0.68 (m, 2H), 0.65–0.54 (m, 2H); ^13^C NMR (101 MHz, DMSO): δ 166.7, 152.8, 139.7, 135.7, 134.4, 133.7, 133.1, 129.5, 128.4, 126.0, 125.9, 125.9, 125.7, 124.7, 124.5, 123.0, 121.4, 120.3, 117.4, 116.4, 116.2, 23.1, 5.8; FTIR [cm^–1^]: 3267, 3083, 3047, 3005, 1640, 1586, 1558, 1528, 1491, 1384; TLC-MS­(ESI) *m*/*z*: 444.9 [M + H + MeOH]^+^; 410.0 [M – H]^−^; 445.8 [M + Cl]^−^; HRMS­(ESI) *m*/*z*: calcd for [M + H]^+^, 412.17672; found, 412.1772; HPLC *t*_ret_: 7.41 min.

#### *N*-Cyclopentyl-3-(4-(3-(naphthalen-1-yl)­ureido)-1*H*-pyrazol-1-yl)­benzamide (**21i**)

(35 mg; 57%) ^1^H NMR (400 MHz, DMSO): δ 8.99 (s, 1H), 8.87 (s, 1H), 8.59 (s, 1H), 8.50 (d, *J* = 7.2 Hz, 1H), 8.21 (s, 1H), 8.12 (d, *J* = 8.4 Hz, 1H), 8.04 (d, *J* = 7.1 Hz, 1H), 7.98–7.91 (m, *J* = 10.2, 4.2 Hz, 2H), 7.87 (s, 1H), 7.75 (d, *J* = 7.8 Hz, 1H), 7.65 (d, *J* = 8.2 Hz, 1H), 7.62–7.53 (m, *J* = 10.6, 6.8, 1.9 Hz, 3H), 7.48 (t, *J* = 7.9 Hz, 1H), 4.31–4.20 (m, 1H), 1.97–1.85 (m, 2H), 1.78–1.65 (m, 2H), 1.62–1.49 (m, *J* = 9.0, 4.6 Hz, 4H); ^13^C NMR (101 MHz, DMSO): δ 165.2, 152.8, 139.7, 136.1, 134.4, 133.7, 133.1, 129.4, 128.4, 126.0, 125.9, 125.9, 125.7, 124.7, 123.0, 121.4, 120.2, 117.4, 116.5, 116.4, 51.0, 32.1, 23.7; FTIR [cm^–1^]: 3284, 3248, 3058, 2954, 2866, 1646, 1633, 1584, 1542, 1486; TLC-MS­(ESI) *m*/*z*: 438.2 [M – H]^−^; HRMS­(ESI) *m*/*z*: calcd for [M + H]^+^, 440.20803; found, 440.2076; HPLC *t*_ret_: 8.50 min.

#### *N*-Cyclohexyl-3-(4-(3-(naphthalen-1-yl)­ureido)-1*H*-pyrazol-1-yl)­benzamide (**21j**)

(39 mg; 53%) ^1^H NMR (400 MHz, DMSO): δ 8.98 (s, 1H), 8.86 (s, 1H), 8.58 (s, 1H), 8.41 (d, *J* = 7.9 Hz, 1H), 8.24–8.18 (m, 1H), 8.12 (d, *J* = 8.4 Hz, 1H), 8.05–8.00 (m, 1H), 7.97–7.91 (m, 2H), 7.86 (s, 1H), 7.75 (d, *J* = 7.8 Hz, 1H), 7.65 (d, *J* = 8.2 Hz, 1H), 7.62–7.52 (m, 3H), 7.48 (t, *J* = 7.9 Hz, 1H), 3.86–3.73 (m, *J* = 7.2, 3.4 Hz, 1H), 1.91–1.69 (m, 4H), 1.62 (d, *J* = 12.4 Hz, 1H), 1.40–1.24 (m, 4H), 1.19–1.06 (m, 1H); ^13^C NMR (101 MHz, DMSO): δ 164.6, 152.8, 139.7, 136.2, 134.4, 133.7, 133.1, 129.5, 128.4, 126.0, 125.9, 125.9, 125.7, 124.7, 123.0, 121.4, 120.2, 117.4, 116.5, 116.4, 48.5, 32.4, 25.3, 25.0; FTIR [cm^–1^]: 3273, 2930, 2852, 1633, 1585, 1539, 1486, 1388, 1328, 1236; TLC-MS­(ESI) *m*/*z*: 476.0 [M + Na]^+^; 452.1 [M – H]^−^; 487.7 [M + Cl]^−^; HRMS­(ESI) *m*/*z*: calcd for [M + H]^+^, 454.22368; found, 454.2241; HPLC *t*_ret_: 8.88 min.

#### (*RS*)-3-(4-(3-(Naphthalen-1-yl)­ureido)-1*H*-pyrazol-1-yl)-*N*-((tetrahydrofuran-2-yl)­methyl)­benzamide (**21k**)

(52 mg; 71%) ^1^H NMR (400 MHz, DMSO): δ 8.99 (s, 1H), 8.87 (s, 1H), 8.77 (t, *J* = 5.8 Hz, 1H), 8.60 (s, 1H), 8.25 (t, *J* = 1.7 Hz, 1H), 8.12 (d, *J* = 8.4 Hz, 1H), 8.04 (dd, *J* = 7.6, 0.8 Hz, 1H), 7.99–7.95 (m, 1H), 7.95–7.91 (m, 1H), 7.86 (s, 1H), 7.79–7.73 (m, 1H), 7.65 (d, *J* = 8.2 Hz, 1H), 7.62–7.52 (m, 3H), 7.48 (t, *J* = 7.9 Hz, 1H), 4.01 (p, *J* = 6.3 Hz, 1H), 3.83–3.75 (m, 1H), 3.64 (dd, *J* = 14.3, 7.6 Hz, 1H), 3.35 (t, *J* = 5.9 Hz, 2H), 1.98–1.75 (m, 3H), 1.65–1.55 (m, 1H); ^13^C NMR (101 MHz, DMSO): δ 165.6, 152.8, 139.7, 135.7, 134.4, 133.7, 133.1, 129.6, 128.4, 126.0, 125.9, 125.9, 125.7, 124.7, 124.6, 123.0, 121.4, 120.3, 117.3, 116.5, 116.5, 77.1, 67.1, 43.5, 28.7, 25.1; FTIR [cm^–1^]: 3260, 2952, 2924, 2855, 1639, 1585, 1540, 1482, 1383, 1314; TLC-MS­(ESI) *m*/*z*: 454.0 [M – H]^−^; 489.8 [M + Cl]^−^; HRMS­(ESI) *m*/*z*: calcd for [M + H]^+^, 456.20294; found, 456.2026; HPLC *t*_ret_: 7.50 min.

#### (*RS*)-3-(4-(3-(Naphthalen-1-yl)­ureido)-1*H*-pyrazol-1-yl)-*N*-((tetrahydrofuran-3-yl)­methyl)­benzamide (**21l**)

(40 mg; 54%) ^1^H NMR (400 MHz, DMSO): δ 8.99 (s, 1H), 8.87 (s, 1H), 8.79 (t, *J* = 5.7 Hz, 1H), 8.59 (s, 1H), 8.27–8.20 (m, 1H), 8.12 (d, *J* = 8.4 Hz, 1H), 8.06–8.01 (m, 1H), 8.00–7.95 (m, 1H), 7.95–7.92 (m, 1H), 7.87 (s, 1H), 7.75 (d, *J* = 7.8 Hz, 1H), 7.65 (d, *J* = 8.2 Hz, 1H), 7.62–7.52 (m, 3H), 7.48 (t, *J* = 7.9 Hz, 1H), 3.76 (td, *J* = 8.0, 5.8 Hz, 1H), 3.70 (dd, *J* = 8.5, 7.0 Hz, 1H), 3.63 (dd, *J* = 14.8, 7.9 Hz, 1H), 3.51 (dd, *J* = 8.5, 5.2 Hz, 1H), 3.33–3.20 (m, 2H), 2.54 (d, *J* = 5.8 Hz, 1H), 2.02–1.90 (m, 1H), 1.68–1.57 (m, 1H); ^13^C NMR (101 MHz, DMSO): δ 165.6, 152.8, 139.8, 135.8, 134.4, 133.7, 133.1, 129.6, 128.4, 126.0, 125.9, 125.9, 125.7, 124.7, 124.6, 123.0, 121.4, 120.3, 117.4, 116.4, 116.3, 70.5, 66.8, 42.0, 38.9, 29.5; FTIR [cm^–1^]: 3288, 3253, 3101, 3070, 2959, 2850, 1647, 1584, 1546, 1483; TLC-MS­(ESI) *m*/*z*: 478.2 [M + Na]^+^; 454.2 [M – H]^−^; 489.8 [M + Cl]^−^; HRMS­(ESI) *m*/*z*: calcd for [M + H]^+^, 456.20294; found, 456.2033; HPLC *t*_ret_: 7.20 min.

#### (*RS*)-3-(4-(3-(Naphthalen-1-yl)­ureido)-1*H*-pyrazol-1-yl)-*N*-(pyrrolidin-3-yl)­benzamide (**21m**) (**HCl Salt**)

(45 mg; 59%) ^1^H NMR (400 MHz, DMSO): δ 9.62 (s, 1H), 9.35–9.17 (m, *J* = 17.4 Hz, 3H), 8.95 (d, *J* = 6.3 Hz, 1H), 8.60 (s, 1H), 8.34–8.29 (m, *J* = 7.9 Hz, 2H), 8.08 (d, *J* = 7.4 Hz, 1H), 8.00 (dd, *J* = 8.1, 1.1 Hz, 1H), 7.94–7.89 (m, 1H), 7.86 (s, 1H), 7.80 (d, *J* = 7.7 Hz, 1H), 7.64–7.51 (m, 4H), 7.47 (t, *J* = 7.9 Hz, 1H), 4.62–4.53 (m, 1H), 3.48–3.35 (m, 2H), 3.31–3.20 (m, 2H), 2.22 (dq, *J* = 14.8, 7.5 Hz, 1H), 2.06 (td, *J* = 13.0, 6.0 Hz, 1H); ^13^C NMR (101 MHz, DMSO): δ 165.8, 153.0, 139.7, 135.2, 134.7, 133.7, 133.1, 129.6, 128.3, 125.9, 125.8, 125.6, 124.9, 124.8, 122.6, 121.7, 120.6, 116.7, 116.5, 116.3, 49.2, 49.1, 43.6, 29.8; FTIR [cm^–1^]: 3246, 2958, 2748, 2087, 1647, 1539, 1490, 1388, 1341, 1252; TLC-MS­(ESI) *m*/*z*: 441.4 [M + H]^+^; 439.4 [M – H]^−^; 475.3 [M + Cl]^−^; HRMS­(ESI) *m*/*z*: calcd for [M + H]^+^, 441.20327; found, 441.2032; HPLC *t*_ret_: 4.97 min.

#### 3-(4-(3-(Naphthalen-1-yl)­ureido)-1*H*-pyrazol-1-yl)-*N*-(piperidin-4-yl)­benzamide (**21n**) (**HCl Salt**)

(66 mg; 83%) ^1^H NMR (400 MHz, DMSO): δ 9.72 (s, 1H), 9.31 (s, 1H), 8.99–8.81 (m, 2H), 8.73 (d, *J* = 7.4 Hz, 1H), 8.58 (s, 1H), 8.35 (d, *J* = 7.9 Hz, 1H), 8.26 (s, 1H), 8.09 (d, *J* = 7.4 Hz, 1H), 7.98 (dd, *J* = 8.1, 1.1 Hz, 1H), 7.94–7.89 (m, 1H), 7.84 (s, 1H), 7.77 (d, *J* = 7.7 Hz, 1H), 7.62 (d, *J* = 8.1 Hz, 1H), 7.59–7.51 (m, 3H), 7.47 (t, *J* = 7.9 Hz, 1H), 4.16–4.05 (m, 1H), 3.32 (d, *J* = 12.5 Hz, 2H), 3.02 (dd, *J* = 21.8, 11.5 Hz, 2H), 2.05–1.94 (m, 2H), 1.88–1.75 (m, 2H); ^13^C NMR (101 MHz, DMSO): δ 165.2, 153.0, 139.7, 135.6, 134.7, 133.8, 133.0, 129.5, 128.3, 125.9, 125.8, 125.6, 124.9, 124.8, 122.6, 121.8, 120.4, 116.7, 116.5, 116.2, 44.5, 42.2, 28.2; FTIR [cm^–1^]: 3241, 3035, 2928, 2725, 2486, 1583, 1540, 1488, 1388, 1341; TLC-MS­(ESI) *m*/*z*: 455.4 [M + H]^+^; 477.4 [M + Na]^+^; 453.4 [M – H]^−^; 489.5 [M + Cl]^−^; HRMS­(ESI) *m*/*z*: calcd for [M + H]^+^, 455.21892; found, 455.2177; HPLC *t*_ret_: 5.06 min.

#### (*RS*)-3-(4-(3-(Naphthalen-1-yl)­ureido)-1*H*-pyrazol-1-yl)-*N*-(pyrrolidin-3-ylmethyl)­benzamide (**21o**) (**HCl Salt**)

(14 mg; 18%; **HCl salt**) ^1^H NMR (400 MHz, DMSO): δ 9.68 (s, 1H), 9.29 (s, 1H), 9.09 (br s, 2H), 8.93 (t, *J* = 5.7 Hz, 1H), 8.59 (s, 1H), 8.34 (d, *J* = 7.9 Hz, 1H), 8.28 (s, 1H), 8.08 (d, *J* = 7.2 Hz, 1H), 7.99 (dd, *J* = 8.1, 1.3 Hz, 1H), 7.94–7.89 (m, 1H), 7.84 (s, 1H), 7.77 (d, *J* = 7.8 Hz, 1H), 7.64–7.51 (m, 4H), 7.47 (t, *J* = 7.9 Hz, 1H), 3.41–3.34 (m, 2H), 3.32–3.19 (m, 2H), 3.17–3.07 (m, 1H), 2.97–2.89 (m, 1H), 2.61–2.54 (m, 1H), 2.08–1.97 (m, 1H), 1.69 (dq, *J* = 13.0, 8.2 Hz, 1H); ^13^C NMR (101 MHz, DMSO): δ 165.9, 153.0, 139.8, 135.5, 134.7, 133.7, 133.0, 129.6, 128.3, 125.9, 125.8, 125.6, 124.9, 124.6, 122.6, 121.8, 120.4, 116.7, 116.2, 47.7, 44.3, 40.9, 37.9, 27.7; FTIR [cm^–1^]: 3261, 3047, 2751, 1636, 1539, 1490, 1388, 1342, 1252, 1169; TLC-MS­(ESI) *m*/*z*: 455.4 [M + H]^+^; HRMS­(ESI) *m*/*z*: calcd for [M + H]^+^, 455.21892; found, 455.2182; HPLC *t*_ret_: 5.06 min.

#### N-Allyl-3-(4-(3-(naphthalen-1-yl)­ureido)-1*H*-pyrazol-1-yl)­benzamide (**21p**)

(57 mg; 74%) ^1^H NMR (400 MHz, DMSO): δ 9.00 (br s, 1H), 8.88 (br s, 2H), 8.61 (s, 1H), 8.27 (t, *J* = 1.7 Hz, 1H), 8.12 (d, *J* = 8.4 Hz, 1H), 8.06–8.01 (m, 1H), 7.98 (dd, *J* = 8.1, 1.3 Hz, 1H), 7.95–7.91 (m, 1H), 7.86 (s, 1H), 7.78 (d, *J* = 7.9 Hz, 1H), 7.65 (d, *J* = 8.2 Hz, 1H), 7.62–7.52 (m, 3H), 7.48 (t, *J* = 7.9 Hz, 1H), 5.93 (ddt, *J* = 17.1, 10.4, 5.3 Hz, 1H), 5.20 (dq, *J* = 17.2, 1.7 Hz, 1H), 5.11 (dq, *J* = 10.3, 1.5 Hz, 1H), 3.95 (s, 2H); ^13^C NMR (101 MHz, DMSO): δ 165.3, 152.8, 139.8, 135.7, 135.3, 134.4, 133.7, 133.1, 129.6, 128.4, 126.0, 125.9, 125.9, 125.7, 124.8, 124.6, 123.0, 121.4, 120.4, 117.3, 116.4, 116.2, 115.3, 41.6; FTIR [cm^–1^]: 3285, 3232, 3057, 2906, 1644, 1584, 1539, 1488, 1395, 1344; TLC-MS­(ESI) *m*/*z*: 434.1 [M + Na]^+^; 410.2 [M – H]^−^; 446.3 [M + Cl]^−^; HRMS­(ESI) *m*/*z*: calcd for [M + H]^+^, 412.17672; found, 412.1766; HPLC *t*_ret_: 7.55 min.

#### N-(2-Methylallyl)-3-(4-(3-(naphthalen-1-yl)­ureido)-1*H*-pyrazol-1-yl)­benzamide (**21q**)

(62 mg; 78%) ^1^H NMR (400 MHz, DMSO): δ 8.99 (s, 1H), 8.91–8.81 (m, 2H), 8.61 (s, 1H), 8.28 (s, 1H), 8.12 (d, *J* = 8.3 Hz, 1H), 8.03 (d, *J* = 7.5 Hz, 1H), 7.98 (d, *J* = 7.9 Hz, 1H), 7.94 (d, *J* = 7.9 Hz, 1H), 7.86 (s, 1H), 7.78 (d, *J* = 7.5 Hz, 1H), 7.65 (d, *J* = 8.1 Hz, 1H), 7.62–7.53 (m, 3H), 7.48 (t, *J* = 7.8 Hz, 1H), 4.84 (d, *J* = 11.0 Hz, 2H), 3.87 (d, *J* = 5.1 Hz, 2H), 1.74 (s, 3H); ^13^C NMR (101 MHz, DMSO): δ 165.3, 152.8, 142.5, 139.8, 135.7, 134.4, 133.7, 133.1, 129.6, 128.4, 126.0, 125.9, 125.9, 125.7, 124.7, 124.6, 123.0, 121.4, 120.4, 117.4, 116.5, 116.2, 110.0, 44.6, 20.3; FTIR [cm^–1^]: 3271, 3069, 2971, 2915, 1642, 1585, 1543, 1485, 1395, 1343; TLC-MS­(ESI) *m*/*z*: 448.2 [M + Na]^+^; 424.2 [M – H]^−^; 460.2 [M + Cl]^−^; HRMS­(ESI) *m*/*z*: calcd for [M + H]^+^, 426.19238; found, 426.1925; HPLC *t*_ret_: 7.99 min.

#### 3-(4-(3-(Naphthalen-1-yl)­ureido)-1*H*-pyrazol-1-yl)-*N*-(prop-2-yn-1-yl)­benzamide (**21r**)

(70 mg; 91%) ^1^H NMR (400 MHz, DMSO): δ 9.16 (t, *J* = 5.4 Hz, 1H), 9.01 (s, 1H), 8.88 (s, 1H), 8.62 (s, 1H), 8.27 (s, 1H), 8.12 (d, *J* = 8.3 Hz, 1H), 8.04 (d, *J* = 7.5 Hz, 1H), 8.00 (dd, *J* = 8.1, 1.2 Hz, 1H), 7.94 (d, *J* = 7.8 Hz, 1H), 7.87 (s, 1H), 7.77 (d, *J* = 7.7 Hz, 1H), 7.65 (d, *J* = 8.2 Hz, 1H), 7.62–7.53 (m, 3H), 7.48 (t, *J* = 7.9 Hz, 1H), 4.11 (dd, *J* = 5.3, 2.3 Hz, 2H), 3.17 (t, *J* = 2.3 Hz, 1H); ^13^C NMR (101 MHz, DMSO): δ 165.2, 152.8, 139.8, 135.0, 134.5, 133.8, 133.1, 129.8, 128.4, 126.0, 125.9, 125.9, 125.7, 124.8, 124.6, 123.0, 121.4, 120.6, 117.3, 116.4, 116.3, 81.2, 73.1, 28.6; FTIR [cm^–1^]: 3270, 3092, 3047, 1639, 1585, 1559, 1534, 1485, 1394, 1342; TLC-MS­(ESI) *m*/*z*: 432.2 [M + Na]+; 464.2 [M + Na + MeOH]^+^; 408.3 [M – H]^−^; 444.3 [M + Cl]^−^; HRMS­(ESI) *m*/*z*: calcd for [M + H]^+^, 410.16107; found, 410.1605; HPLC *t*_ret_: 7.17 min.

### Compounds of Table 4

#### N-Cyclopropyl-4-(4-(3-(naphthalen-1-yl)­ureido)-1*H*-pyrazol-1-yl)­benzamide (**28a**)

(68 mg; 95%) ^1^H NMR (400 MHz, DMSO): δ 8.99 (s, 1H), 8.87 (s, 1H), 8.57 (s, 1H), 8.47 (d, *J* = 3.9 Hz, 1H), 8.12 (d, *J* = 8.3 Hz, 1H), 8.03 (d, *J* = 7.5 Hz, 1H), 7.97–7.85 (m, 6H), 7.65 (d, *J* = 8.1 Hz, 1H), 7.62–7.53 (m, 2H), 7.48 (t, *J* = 7.9 Hz, 1H), 2.91–2.81 (m, 1H), 0.75–0.66 (m, 2H), 0.62–0.52 (m, 2H); ^13^C NMR (101 MHz, DMSO): δ 166.6, 152.8, 141.5, 134.4, 133.7, 133.6, 131.2, 128.7, 128.4, 126.0, 125.9, 125.9, 125.7, 125.0, 123.0, 121.4, 117.4, 117.0, 116.3, 23.1, 5.7; FTIR [cm^–1^]: 3265, 3094, 3011, 1636, 1610, 1591, 1540, 1508, 1500, 1457; TLC-MS­(ESI) *m*/*z*: 433.9 [M + Na]^+^; 410.1 [M – H]^−^; 446.0 [M + Cl]^−^; HRMS­(ESI) *m*/*z*: calcd for [M + H]^+^, 412.17672; found, 412.1770; HPLC *t*_ret_: 7.23 min.

#### *N*-Methyl-4-(4-(3-(naphthalen-1-yl)­ureido)-1*H*-pyrazol-1-yl)­benzamide (**28b**)

(26 mg; 39%) ^1^H NMR (400 MHz, DMSO): δ 8.99 (s, 1H), 8.86 (s, 1H), 8.56 (s, 1H), 8.51–8.40 (m, 1H), 8.12 (d, *J* = 8.1 Hz, 1H), 8.03 (d, *J* = 7.3 Hz, 1H), 7.99–7.84 (m, 6H), 7.65 (d, *J* = 8.0 Hz, 1H), 7.57 (dt, *J* = 14.6, 6.8 Hz, 2H), 7.48 (t, *J* = 7.8 Hz, 1H), 2.80 (d, *J* = 3.7 Hz, 3H); ^13^C NMR (101 MHz, DMSO): δ 165.8, 152.8, 141.5, 134.4, 133.7, 133.7, 131.4, 128.6, 128.4, 126.0, 125.9, 125.9, 125.7, 125.0, 123.0, 121.4, 117.3, 117.1, 116.3, 26.3; FTIR [cm^–1^]: 3261, 3092, 3055, 1636, 1610, 1593, 1554, 1508, 1502, 1405; TLC-MS­(ESI) *m*/*z*: 407.9 [M + Na]^+^; 384.0 [M – H]^−^; 420.2 [M + Cl]^−^; HRMS­(ESI) *m*/*z*: calcd for [M + H]^+^, 386.16107; found, 386.1625; HPLC *t*_ret_: 6.72 min.

### Compounds of Table 5

#### *N*-Methyl-2-(4-(3-(naphthalen-1-yl)­ureido)-1*H*-pyrazol-1-yl)­isonicotinamide (**35a**)

(62 mg; 70%) ^1^H NMR (400 MHz, DMSO): δ 9.04 (s, 1H), 8.96–8.85 (m, 2H), 8.73 (s, 1H), 8.58 (d, *J* = 4.7 Hz, 1H), 8.28 (s, 1H), 8.11 (d, *J* = 8.1 Hz, 1H), 8.00 (d, *J* = 7.4 Hz, 1H), 7.97–7.90 (m, 2H), 7.70–7.63 (m, 2H), 7.57 (dt, *J* = 14.7, 6.8 Hz, 2H), 7.49 (t, *J* = 7.8 Hz, 1H), 2.83 (d, *J* = 3.7 Hz, 3H); ^13^C NMR (101 MHz, DMSO): δ 164.4, 152.8, 151.6, 149.0, 144.5, 134.5, 134.4, 133.7, 128.4, 126.2, 125.9, 125.9, 125.7, 125.0, 123.2, 121.5, 118.7, 117.7, 115.3, 109.0, 26.4; FTIR [cm^–1^]: 3307, 3247, 3049, 2932, 2871, 1642, 1609, 1592, 1550, 1456; TLC-MS­(ESI) *m*/*z*: 408.9 [M + Na]^+^; 440.8 [M + Na + MeOH]^+^; 385.0 [M – H]^−^; 420.9 [M + Cl]^−^; HRMS­(ESI) *m*/*z*: calcd for [M + H]^+^, 387.15632; found, 387.1563; HPLC *t*_ret_: 6.77 min.

#### *N*-Methyl-6-(4-(3-(naphthalen-1-yl)­ureido)-1*H*-pyrazol-1-yl)­picolinamide (**36a**)

(16 mg; 26%) ^1^H NMR (400 MHz, DMSO): δ 9.09 (s, 1H), 9.03–8.93 (m, 2H), 8.89 (s, 1H), 8.15–8.02 (m, 4H), 7.97–7.92 (m, 2H), 7.90 (d, *J* = 7.0 Hz, 1H), 7.65 (d, *J* = 8.1 Hz, 1H), 7.62–7.52 (m, 2H), 7.49 (t, *J* = 7.9 Hz, 1H), 2.88 (d, *J* = 4.5 Hz, 3H); ^13^C NMR (101 MHz, DMSO): δ 163.7, 152.9, 150.0, 148.9, 140.6, 135.3, 134.4, 133.7, 128.4, 126.0, 125.9, 125.9, 125.7, 124.7, 123.1, 121.4, 119.0, 117.5, 117.1, 113.9, 26.0; FTIR [cm^–1^]: 3273, 3109, 3051, 1634, 1600, 1579, 1465, 1381, 1267, 1218; TLC-MS­(ESI) *m*/*z*: 408.8 [M + Na]^+^; 384.9 [M – H]^−^; 420.9 [M + Cl]^−^; HRMS­(ESI) *m*/*z*: calcd for [M + H]^+^, 387.15632; found, 387.1547; HPLC *t*_ret_: 7.47 min.

### Compounds of Table 6

#### 2-Methyl-5-(4-(3-(naphthalen-1-yl)­ureido)-1*H*-pyrazol-1-yl)-*N*-(1-(piperidin-4-yl)-1*H*-pyrazol-4-yl)­benzamide (**45a**) (**HCl Salt**)

(54 mg; 93%) ^1^H NMR (400 MHz, DMSO): δ 10.54 (s, 1H), 9.48 (s, 1H), 9.17 (s, 1H), 9.01 (d, *J* = 9.7 Hz, 1H), 8.81–8.68 (m, 1H), 8.53 (s, 1H), 8.32–8.24 (m, 1H), 8.08 (s, 1H), 8.06 (dd, *J* = 7.6, 0.9 Hz, 1H), 7.94–7.90 (m, 1H), 7.87 (d, *J* = 2.3 Hz, 1H), 7.85–7.79 (m, 2H), 7.64–7.59 (m, 2H), 7.59–7.51 (m, 2H), 7.46 (t, *J* = 7.9 Hz, 1H), 7.40 (d, *J* = 8.5 Hz, 1H), 4.56–4.45 (m, 1H), 3.39 (d, *J* = 12.7 Hz, 2H), 3.12–2.99 (m, 2H), 2.40 (s, 3H), 2.22–2.11 (m, 4H); FTIR [cm^–1^]: 3263, 3129, 3043, 2922, 2791, 2469, 1644, 1598, 1540, 1499; TLC-MS­(ESI) *m*/*z*: 535.5 [M + H]^+^; 533.5 [M – H]^−^; 569.4 [M + Cl]^−^; HRMS­(ESI) *m*/*z*: calcd for [M + H]^+^, 535.25637; found, 535.2556; HPLC *t*_ret_: 5.31 min.

#### 3-Methyl-5-(4-(3-(naphthalen-1-yl)­ureido)-1*H*-pyrazol-1-yl)-*N*-(1-(piperidin-4-yl)-1*H*-pyrazol-4-yl)­benzamide (**45b**) (**HCl Salt**)

(70 mg; 74%) ^1^H NMR (400 MHz, DMSO): δ 10.68 (s, 1H), 9.74 (s, 1H), 9.32 (s, 1H), 9.15 (d, *J* = 10.1 Hz, 1H), 8.91 (q, *J* = 9.9 Hz, 1H), 8.59 (s, 1H), 8.40–8.32 (m, 1H), 8.21–8.17 (m, 1H), 8.13 (s, 1H), 8.09 (dd, *J* = 7.6, 0.8 Hz, 1H), 7.93–7.90 (m, 1H), 7.87 (s, 1H), 7.84 (s, 1H), 7.70 (s, 1H), 7.68 (s, 1H), 7.61 (d, *J* = 8.2 Hz, 1H), 7.59–7.51 (m, 2H), 7.47 (t, *J* = 7.9 Hz, 1H), 4.52 (dt, *J* = 15.0, 7.6 Hz, 1H), 3.39 (d, *J* = 12.6 Hz, 2H), 3.11–2.98 (m, 2H), 2.46 (s, 3H), 2.20–2.13 (m, 4H); ^13^C NMR (101 MHz, DMSO): δ 162.9, 153.0, 140.0, 140.0, 135.2, 134.7, 133.7, 132.8, 130.5, 128.3, 125.8, 125.7, 125.6, 125.3, 124.8, 122.6, 121.8, 121.7, 121.0, 119.3, 116.6, 116.2, 113.7, 55.1, 42.2, 28.7, 21.0; FTIR [cm^–1^]: 3264, 3044, 2920, 2794, 2714, 1662, 1593, 1540, 1390, 1341; TLC-MS­(ESI) *m*/*z*: 535.4 [M + H]^+^; 569.5 [M + Cl]^−^; HRMS­(ESI) *m*/*z*: calcd for [M + H]^+^, 535.25637; found, 535.2547; HPLC *t*_ret_: 9.19 min.

#### *N*-Cyclopropyl-2-methyl-5-(4-(3-(naphthalen-1-yl)­ureido)-1*H*-pyrazol-1-yl)­benzamide (**45c**)

(44 mg; 42%) ^1^H NMR (400 MHz, DMSO): δ 8.93 (s, 1H), 8.84 (s, 1H), 8.50 (s, 1H), 8.43 (d, *J* = 4.3 Hz, 1H), 8.11 (d, *J* = 8.3 Hz, 1H), 8.06–8.01 (m, 1H), 7.96–7.91 (m, 1H), 7.83 (s, 1H), 7.74 (dd, *J* = 8.3, 2.4 Hz, 1H), 7.68 (d, *J* = 2.3 Hz, 1H), 7.64 (d, *J* = 8.2 Hz, 1H), 7.62–7.53 (m, 2H), 7.48 (t, *J* = 7.9 Hz, 1H), 7.33 (d, *J* = 8.4 Hz, 1H), 2.88–2.80 (m, 1H), 2.33 (s, 3H), 0.75–0.67 (m, 2H), 0.59–0.52 (m, 2H); ^13^C NMR (101 MHz, DMSO): δ 169.4, 152.8, 137.8, 137.4, 134.4, 133.7, 132.8, 132.6, 131.5, 128.4, 126.0, 125.9, 125.7, 124.5, 122.9, 121.4, 118.4, 117.3, 116.5, 116.4, 22.7, 18.8, 5.8; FTIR [cm^–1^]: 3235, 3223, 3047, 3015, 1636, 1540, 1497, 1388, 1251, 1237; TLC-MS­(ESI) *m*/*z*: 448.3 [M + Na]^+^; 424.5 [M – H]^−^; HRMS­(ESI) *m*/*z*: calcd for [M + H]^+^, 426.19238; found, 426.1942; HPLC *t*_ret_: 7.44 min.

### Compounds of Table 7

#### 1-(Naphthalen-1-yl)-3-(1-(3-((pyridin-4-ylmethyl)­amino)­phenyl)-1*H*-pyrazol-4-yl)­urea (**51a**)

(77 mg; 74%) ^1^H NMR (400 MHz, DMSO): δ 8.92 (s, 1H), 8.83 (s, 1H), 8.50 (dd, *J* = 4.6, 1.4 Hz, 2H), 8.32 (s, 1H), 8.11 (d, *J* = 8.3 Hz, 1H), 8.02 (d, *J* = 7.1 Hz, 1H), 7.93 (d, *J* = 7.7 Hz, 1H), 7.76 (s, 1H), 7.66–7.51 (m, 3H), 7.47 (t, *J* = 7.9 Hz, 1H), 7.37 (d, *J* = 5.8 Hz, 2H), 7.13 (t, *J* = 8.0 Hz, 1H), 7.02 (t, *J* = 1.9 Hz, 1H), 6.93 (dd, *J* = 7.9, 1.4 Hz, 1H), 6.66 (t, *J* = 6.2 Hz, 1H), 6.46 (dd, *J* = 8.1, 1.7 Hz, 1H), 4.40 (d, *J* = 6.1 Hz, 2H); ^13^C NMR (101 MHz, DMSO): δ 152.8, 149.6, 149.4, 149.3, 140.7, 134.5, 133.7, 132.3, 129.9, 128.4, 126.0, 125.9, 125.7, 124.2, 122.9, 122.2, 121.4, 117.3, 116.2, 109.9, 105.7, 101.8, 45.3; FTIR [cm^–1^]: 3277, 3052, 2849, 1638, 1552, 1497, 1449, 1388, 1319, 1244; TLC-MS­(ESI) *m*/*z*: 434.8 [M + H]^+^; 456.8 [M + Na]^+^; 432.9 [M – H]^−^; 468.8 [M + Cl]^−^; HRMS­(ESI) *m*/*z*: calcd for [M + H]^+^, 435.19271; found, 435.1925; HPLC *t*_ret_: 5.82 min.

#### 1-(1-(3-((Cyclopropylmethyl)­amino)­phenyl)-1*H*-pyrazol-4-yl)-3-(naphthalen-1-yl)­urea (**51b**)

(52 mg; 50%) ^1^H NMR (400 MHz, DMSO): δ 8.90 (s, 1H), 8.83 (s, 1H), 8.35 (s, 1H), 8.11 (d, *J* = 8.4 Hz, 1H), 8.03 (d, *J* = 7.5 Hz, 1H), 7.93 (d, *J* = 7.6 Hz, 1H), 7.77 (s, 1H), 7.64 (d, *J* = 8.2 Hz, 1H), 7.61–7.52 (m, 2H), 7.47 (t, *J* = 7.9 Hz, 1H), 7.14 (t, *J* = 8.0 Hz, 1H), 7.00 (t, *J* = 1.9 Hz, 1H), 6.90 (dd, *J* = 7.9, 1.3 Hz, 1H), 6.50 (dd, *J* = 8.1, 1.7 Hz, 1H), 5.95 (t, *J* = 5.4 Hz, 1H), 2.95 (t, *J* = 6.0 Hz, 2H), 1.09–1.03 (m, 1H), 0.52–0.45 (m, 2H), 0.26–0.21 (m, 2H); ^13^C NMR (101 MHz, DMSO): δ 152.8, 150.0, 140.7, 134.5, 133.7, 132.2, 129.7, 128.4, 126.0, 125.9, 125.7, 124.1, 122.9, 121.4, 117.3, 116.3, 109.8, 105.0, 101.2, 47.4, 10.5, 3.5; FTIR [cm^–1^]: 3377, 3272, 3001, 2922, 2851, 1639, 1588, 1560, 1497, 1437; TLC-MS­(ESI) *m*/*z*: 420.3 [M + Na]^+^; 452.3 [M + Na + MeOH]^+^; 396.4 [M – H]^−^; 432.4 [M + Cl]^−^; HRMS­(ESI) *m*/*z*: calcd for [M + H]^+^, 398.19746; found, 398.1982; HPLC *t*_ret_: 8.59 min.

#### *N*-(3-(4-(3-(Naphthalen-1-yl)­ureido)-1*H*-pyrazol-1-yl)­phenyl)­cyclopropanecarboxamide (**51c**)

(40 mg; 74%) ^1^H NMR (400 MHz, DMSO): δ 10.38 (s, 1H), 8.96 (s, 1H), 8.85 (s, 1H), 8.41 (s, 1H), 8.17 (s, 1H), 8.11 (d, *J* = 8.3 Hz, 1H), 8.03 (d, *J* = 7.5 Hz, 1H), 7.93 (d, *J* = 7.9 Hz, 1H), 7.82 (s, 1H), 7.66–7.52 (m, 3H), 7.51–7.36 (m, 4H), 1.84–1.75 (m, 1H), 0.89–0.75 (m, 4H); ^13^C NMR (101 MHz, DMSO): δ 171.9, 152.8, 140.4, 140.1, 134.4, 133.7, 132.7, 129.8, 128.4, 126.0, 125.9, 125.7, 124.6, 123.0, 121.4, 117.4, 116.0, 112.1, 108.4, 14.6, 7.3; FTIR [cm^–1^]: 3311, 3280, 3099, 3078, 3010, 2920, 1644, 1609, 1588, 1551; TLC-MS­(ESI) *m*/*z*: 434.1 [M + Na]^+^; 410.1 [M – H]^−^; 446.1 [M + Cl]^−^; HRMS­(ESI) *m*/*z*: calcd for [M + H]^+^, 412.17672; found, 412.1767; HPLC *t*_ret_: 7.86 min.

#### *N*-(3-(4-(3-(Naphthalen-1-yl)­ureido)-1*H*-pyrazol-1-yl)­phenyl)­acetamide (**51d**)

(366 mg; 60%) ^1^H NMR (400 MHz, DMSO): δ 10.11 (s, 1H), 9.03 (s, 1H), 8.89 (s, 1H), 8.40 (s, 1H), 8.16–8.11 (m, *J* = 8.0 Hz, 2H), 8.05–8.00 (m, *J* = 7.0 Hz, 1H), 7.95–7.91 (m, *J* = 7.8 Hz, 1H), 7.82 (s, 1H), 7.64 (d, *J* = 8.2 Hz, 1H), 7.61–7.52 (m, 2H), 7.50–7.42 (m, 3H), 7.41–7.36 (m, 1H), 2.08 (s, 3H); ^13^C NMR (101 MHz, DMSO): δ 168.5, 152.8, 140.4, 140.1, 134.5, 133.7, 132.7, 129.8, 128.4, 126.0, 125.9, 125.7, 124.6, 122.9, 121.4, 117.4, 116.1, 116.1, 112.2, 108.4, 24.1; FTIR [cm^–1^]: 3271, 3101, 3055, 2919, 1654, 1644, 1609, 1587, 1547, 1491; TLC-MS­(ESI) *m*/*z*: 407.9 [M + Na]^+^; 384.1 [M – H]^−^; 420.1 [M + Cl]^−^; HRMS­(ESI) *m*/*z*: calcd for [M + H]^+^, 386.16107; found, 386.1615; HPLC *t*_ret_: 6.91 min.

#### *N*-(3-(4-(3-(Naphthalen-1-yl)­ureido)-1*H*-pyrazol-1-yl)­phenyl)­cyclopropanesulfonamide (**51e**)

(19 mg; 20%) ^1^H NMR (400 MHz, DMSO): δ 9.92 (br s, 1H), 9.00 (s, 1H), 8.87 (s, 1H), 8.43 (s, 1H), 8.12 (d, *J* = 8.3 Hz, 1H), 8.02 (d, *J* = 7.4 Hz, 1H), 7.94 (d, *J* = 7.7 Hz, 1H), 7.84 (s, 1H), 7.77–7.69 (m, 1H), 7.65 (d, *J* = 8.1 Hz, 1H), 7.62–7.52 (m, 2H), 7.52–7.44 (m, 2H), 7.41 (t, *J* = 8.1 Hz, 1H), 7.19–7.07 (m, 1H), 2.75–2.62 (m, 1H), 1.02–0.89 (m, 4H); ^13^C NMR (101 MHz, DMSO): δ 152.8, 140.4, 139.7, 134.4, 133.8, 133.0, 130.3, 128.4, 126.0, 125.9, 125.9, 125.7, 124.7, 123.0, 121.4, 117.4, 117.0, 116.2, 112.7, 109.2, 29.6, 5.0; FTIR [cm^–1^]: 3292, 3202, 3122, 3053, 2923, 2850, 1643, 1600, 1586, 1550; TLC-MS­(ESI) *m*/*z*: 470.2 [M + Na]^+^; 446.2 [M – H]^−^; 482.3 [M + Cl]^−^; HRMS­(ESI) *m*/*z*: calcd for [M + H]^+^, 448.14371; found, 448.1437; HPLC *t*_ret_: 7.62 min.

#### *N*-(3-(4-(3-(Naphthalen-1-yl)­ureido)-1*H*-pyrazol-1-yl)­phenyl)-1*H*-pyrrole-3-carboxamide (**51f**)

(17 mg; 6%) ^1^H NMR (400 MHz, DMSO): δ 11.33 (s, 1H), 9.66 (s, 1H), 9.04 (s, 1H), 8.91 (s, 1H), 8.43 (s, 1H), 8.25 (s, 1H), 8.13 (d, *J* = 8.2 Hz, 1H), 8.03 (d, *J* = 7.4 Hz, 1H), 7.94 (d, *J* = 7.8 Hz, 1H), 7.82 (s, 1H), 7.73 (d, *J* = 7.6 Hz, 1H), 7.66–7.52 (m, 4H), 7.50–7.36 (m, 3H), 6.84 (d, *J* = 1.5 Hz, 1H), 6.68 (s, 1H); ^13^C NMR (101 MHz, DMSO): δ 163.0, 152.8, 141.0, 140.0, 134.5, 133.7, 132.6, 129.6, 128.4, 126.0, 125.9, 125.7, 124.5, 123.0, 121.6, 121.4, 119.4, 118.9, 117.3, 116.8, 116.0, 111.8, 109.1, 107.8; FTIR [cm^–1^]: 3254, 1636, 1597, 1540, 1526, 1448, 1388, 1334, 1247, 1196; TLC-MS­(ESI) *m*/*z*: 459.3 [M + Na]^+^; 435.4 [M – H]^−^; 471.1 [M + Cl]^−^; HRMS­(ESI) *m*/*z*: calcd for [M + H]^+^, 437.17197; found, 437.1741; HPLC *t*_ret_: 7.18 min.

#### 2,2,2-Trifluoro-*N*-(3-(4-(3-(naphthalen-1-yl)­ureido)-1*H*-pyrazol-1-yl)­phenyl)­acetamide (**51g**)

(43 mg; 37%) ^1^H NMR (400 MHz, DMSO): δ 11.41 (s, 1H), 8.98 (s, 1H), 8.86 (s, 1H), 8.48 (s, 1H), 8.21 (t, *J* = 2.0 Hz, 1H), 8.12 (d, *J* = 8.4 Hz, 1H), 8.03 (dd, *J* = 7.6, 0.8 Hz, 1H), 7.96–7.91 (m, 1H), 7.86 (s, 1H), 7.68–7.63 (m, 2H), 7.62–7.57 (m, 2H), 7.57–7.45 (m, 3H); ^13^C NMR (101 MHz, DMSO): δ 154.65 (q, *J* = 37.4 Hz), 152.8, 140.1, 137.4, 134.4, 133.7, 133.1, 130.2, 128.4, 126.0, 125.9, 125.9, 125.7, 124.8, 123.0, 121.4, 118.0, 117.4, 116.1, 115.7 (q, *J* = 288.4 Hz),114.6, 110.5; FTIR [cm^–1^]: 3343, 3273, 1735, 1706, 1648, 1597, 1560, 1497, 1463, 1390; TLC-MS­(ESI) *m*/*z*: 461.9 [M + Na]^+^; 438.0 [M – H]^−^; HRMS­(ESI) *m*/*z*: calcd for [M + H]^+^, 440.13281; found, 440.1336; HPLC *t*_ret_: 8.29 min.

### Compounds of Table 8

#### *N*-(3-(4-(3-(Naphthalen-1-yl)­ureido)-1*H*-pyrazol-1-yl)­phenyl)-4-propionamidobenzamide (**56a**)

(5 mg; 7%) ^1^H NMR (700 MHz, DMSO): δ 10.27 (s, 1H), 10.16 (s, 1H), 8.97 (s, 1H), 8.86 (s, 1H), 8.45 (s, 1H), 8.31 (t, *J* = 1.9 Hz, 1H), 8.12 (d, *J* = 8.4 Hz, 1H), 8.03 (d, *J* = 7.4 Hz, 1H), 7.97 (d, *J* = 8.7 Hz, 2H), 7.94 (d, *J* = 8.0 Hz, 1H), 7.83 (s, 1H), 7.79–7.71 (m, 3H), 7.65 (d, *J* = 8.1 Hz, 1H), 7.62–7.59 (m, 1H), 7.57–7.54 (m, 1H), 7.51 (dd, *J* = 8.1, 1.4 Hz, 1H), 7.48 (t, *J* = 7.9 Hz, 1H), 7.44 (t, *J* = 8.1 Hz, 1H), 2.37 (q, *J* = 7.5 Hz, 2H), 1.10 (t, *J* = 7.5 Hz, 3H); ^13^C NMR (176 MHz, DMSO): δ 172.47, 165.03, 152.80, 142.51, 140.43, 140.00, 134.45, 133.74, 132.69, 129.70, 128.67, 128.61, 128.42, 125.98, 125.91, 125.71, 124.59, 122.96, 121.39, 118.14, 117.33, 117.27, 116.06, 112.62, 109.61, 29.59, 9.50; FTIR [cm^–1^]: 3281, 3237, 3110, 2975, 1659, 1643, 1605, 1587, 1554, 1522; TLC-MS­(ESI) *m*/*z*: 541.3 [M + Na]^+^; 517.3 [M – H]^−^; 553.2 [M + Cl]^−^; HRMS­(ESI) *m*/*z*: calcd for [M + H]^+^, 519.21384; found, 519.2137; HPLC *t*_ret_: 7.93 min.

#### 4-Acrylamido-*N*-(3-(4-(3-(naphthalen-1-yl)­ureido)-1*H*-pyrazol-1-yl)­phenyl)­benzamide (**56b**)

(12 mg; 8%) ^1^H NMR (700 MHz, DMSO): δ 10.44 (s, 1H), 10.31 (s, 1H), 8.97 (s, 1H), 8.86 (s, 1H), 8.46 (s, 1H), 8.32 (t, *J* = 1.8 Hz, 1H), 8.12 (d, *J* = 8.4 Hz, 1H), 8.03 (d, *J* = 7.4 Hz, 1H), 8.00 (d, *J* = 8.7 Hz, 2H), 7.94 (d, *J* = 8.0 Hz, 1H), 7.86–7.81 (m, 3H), 7.74 (d, *J* = 8.0 Hz, 1H), 7.65 (d, *J* = 8.1 Hz, 1H), 7.61–7.59 (m, 1H), 7.56–7.54 (m, 1H), 7.51 (dd, *J* = 8.1, 1.3 Hz, 1H), 7.48 (t, *J* = 7.8 Hz, 1H), 7.45 (t, *J* = 8.1 Hz, 1H), 6.48 (dd, *J* = 16.9, 10.2 Hz, 1H), 6.32 (dd, *J* = 17.0, 1.7 Hz, 1H), 5.82 (dd, *J* = 10.2, 1.7 Hz, 1H); ^13^C NMR (176 MHz, DMSO): δ 165.0, 163.5, 152.8, 142.1, 140.4, 140.0, 134.5, 133.8, 132.7, 131.6, 129.7, 129.3, 128.8, 128.4, 127.7, 126.0, 125.9, 125.7, 124.6, 123.0, 121.4, 118.6, 117.3, 117.3, 116.1, 112.7, 109.6; FTIR [cm^–1^]: 3247, 1664, 1643, 1605, 1590, 1558, 1524, 1495, 1464, 1410; TLC-MS­(ESI) *m*/*z*: 539.3 [M + Na]^+^; 515.4 [M – H]^−^; HRMS­(ESI) *m*/*z*: calcd for [M + H]^+^, 517.19819; found, 517.1972; HPLC *t*_ret_: 7.86 min.

#### *N*-(3-(4-(3-(Naphthalen-1-yl)­ureido)-1*H*-pyrazol-1-yl)­phenyl)-3-propionamidobenzamide (**56c**)

(37 mg; 22%) ^1^H NMR (700 MHz, DMSO): δ 10.41 (s, 1H), 10.08 (s, 1H), 8.97 (s, 1H), 8.86 (s, 1H), 8.46 (s, 1H), 8.31 (t, *J* = 1.9 Hz, 1H), 8.14–8.11 (m, 2H), 8.03 (d, *J* = 7.5 Hz, 1H), 7.94 (d, *J* = 8.0 Hz, 1H), 7.86 (dd, *J* = 8.1, 1.0 Hz, 1H), 7.84 (s, 1H), 7.75–7.72 (m, 1H), 7.66–7.63 (m, 2H), 7.61–7.58 (m, 1H), 7.56–7.51 (m, 2H), 7.50–7.44 (m, 3H), 2.36 (q, *J* = 7.6 Hz, 2H), 1.11 (t, *J* = 7.6 Hz, 3H); ^13^C NMR (176 MHz, DMSO): δ 172.3, 165.8, 152.8, 140.3, 140.0, 139.6, 135.5, 134.5, 133.8, 132.7, 129.8, 128.8, 128.4, 126.0, 125.9, 125.7, 124.6, 123.0, 122.1, 121.9, 121.4, 118.6, 117.3, 116.1, 112.8, 109.7, 29.5, 9.6; FTIR [cm^–1^]: 3270, 3110, 3058, 2968.1639, 1605, 1588, 1543, 1496, 1485; TLC-MS­(ESI) *m*/*z*: 541.3 [M + Na]^+^; 517.4 [M – H]^−^; HRMS­(ESI) *m*/*z*: calcd for [M + H]^+^, 519.21384; found, 519.2138; HPLC *t*_ret_: 7.98 min.

#### 3-Acrylamido-*N*-(3-(4-(3-(naphthalen-1-yl)­ureido)-1*H*-pyrazol-1-yl)­phenyl)­benzamide (**56d**)

(16 mg; 13%) ^1^H NMR (700 MHz, DMSO): δ 10.44 (s, 1H), 10.37 (s, 1H), 8.97 (s, 1H), 8.86 (s, 1H), 8.46 (s, 1H), 8.32–8.30 (m, 1H), 8.21–8.18 (m, 1H), 8.12 (d, *J* = 8.4 Hz, 1H), 8.03 (d, *J* = 7.4 Hz, 1H), 7.97–7.92 (m, 2H), 7.84 (s, 1H), 7.74 (d, *J* = 8.0 Hz, 1H), 7.70 (d, *J* = 7.7 Hz, 1H), 7.65 (d, *J* = 8.1 Hz, 1H), 7.61–7.58 (m, 1H), 7.55 (d, *J* = 7.6 Hz, 1H), 7.54–7.49 (m, 2H), 7.49–7.44 (m, 2H), 6.47 (dd, *J* = 17.0, 10.2 Hz, 1H), 6.30 (dd, *J* = 17.0, 1.6 Hz, 1H), 5.80 (dd, *J* = 10.2, 1.6 Hz, 1H); ^13^C NMR (176 MHz, DMSO): δ 165.7, 163.4, 152.8, 140.3, 140.0, 139.2, 135.6, 134.5, 133.7, 132.8, 131.7, 129.8, 128.9, 128.4, 127.3, 126.0, 125.9, 125.7, 124.6, 123.0, 122.5, 122.4, 121.4, 118.9, 117.3, 116.1, 112.9, 109.7; FTIR [cm^–1^]: 3280, 3237, 3107, 1644, 1588, 1546, 1485, 1465, 1385, 1314; TLC-MS­(ESI) *m*/*z*: 539.4 [M + Na]^+^; 515.4 [M – H]^−^; 551.4 [M + Cl]^−^; HRMS­(ESI) *m*/*z*: calcd for [M + H]^+^, 517.19819; found, 517.1988; HPLC *t*_ret_: 7.94 min.

## Supplementary Material





## Notes

Western Blots; Metabolic stability data of **21h**, **21b** and **51d** in mouse liver microsomes; Pharmacokinetic study data; Determination of time dependent inhibition; enzyme labeling mass spectra; Glutathione stability assays for Afatinib and **56d**; *k*_inact_/*K*_I_ determination; Kinomscreen; Additional evaluated compounds; Synthetic procedures of precursor and inhibitor compounds; NMR spectra; HPLC traces (PDF)
Molecular formula strings (CSV)

## Abbreviations

ACN: acetonitrile
ASAP: atmospheric solid analysis probe
AUC: area under the curve
Boc: *tert*-butyloxycarbonyl
CDI: 1,1′-carbonyldiimidazole
BSA: bovine serum albumin
DAD: diode array detector
DIPEA: *N,N*-diisopropylethylamine
DMA: Dimethylacetamide
EDC: 1-ethyl-3-(3-(dimethylamino)­propyl)­carbodiimide
EtOAc: ethyl acetate
GSH: glutathione
HATU: 1-[Bis­(dimethylamino)­methylene]-1*H*-1,2,3-triazolo­[4,5-*b*]­pyridinium 3-oxid hexafluorophosphateHEPES, (4-(2-hydroxyethyl)-1-piperazineethanesulfonic acid)
HOBt: 1-hydroxybenzotriazole
HPLC: high performance liquid chromatography
HR: hydrophobic region
IPMS: intact protein mass spectrometry
JNK: c-Jun N-terminal kinase
LCMS: liquid chromatography mass spectrometry
MAPK: mitogen-activated protein kinase
MKK4: mitogen-activated protein kinase kinase 4
NMR: nuclear magnetic resonance
PBS: phosphate-buffered saline
PEG400: polyethylene glycol 400
PK: pharmacokinetic
POC: percentage of control
PVDF: polyvinylidene fluoride
RIPA-buffer: Radioimmunoprecipitation assay-buffer
RT: room temperature
TDI: time dependent inhibition
TEA: triethylamine
TFA: trifluoroacetic acid
SAR: structure activity relationship
SDS-PAGE: Sodium Dodecyl Sulfate – PolyAcrylamide Gel Electrophoresis
TBST-buffer: Tris-buffered saline with Tween20-buffer
TLC: thin layer chromatography
