# Supplementary material for: A “Ligand First” Approach toward Selective, Covalent JNK2/3 Inhibitors
Source: J Med Chem. 2025 May 22;68(11):12004–28. doi: 10.1021/acs.jmedchem.5c00884 (PMC12169684; doi:10.1021/acs.jmedchem.5c00884)
Supplement: Supplementary file 1 [file jm5c00884_si_001.pdf]

Supplementary Information (SI)

A “Ligand First” Approach Toward Selective, Covalent JNK2/3 Inhibitors

Valentin R. Wydra[1], Nicole Plank[2], Stefan Zwirner[3], Roland Selig[1,4], Alexander Rasch[1], Benedikt Masberg[5], Michael Lämmerhofer[5], Lars Zender[3,4,6,7,8], Pierre Koch[2], Wolfgang Albrecht[4], Stefan Laufer[1,6,7,8]*

[1]Department of Pharmaceutical/Medicinal Chemistry, Eberhard Karls Universität Tübingen, Auf der Morgenstelle 8, 72076, Tübingen, DE, Germany

[2]Department of Pharmaceutical/Medicinal Chemistry II, Institute of Pharmacy, University of Regensburg, Universitätsstraße 31, 93053 Regensburg, Germany

[3]Department of Medical Oncology and Pneumology (Internal Medicine VIII), University Hospital of Tübingen, Otfried-Müller-Straße 14, 72076 Tübingen, Germany

[4]HepaRegenix GmbH, Eisenbahnstraße 63, 72072, Tübingen, Germany

[5]Pharmaceutical (Bio-) Analysis, Institute of Pharmaceutical Sciences, Department of Pharmaceutical/Medicinal Chemistry, Eberhard Karls Universität Tübingen, Tübingen, 72076, Germany

[6]IFIT Cluster of Excellence EXC 2180 ‘Image Guided and Functionally Instructed Tumor Therapies’, Eberhard Karls University of Tübingen, 72076, Tübingen, Germany

[7]German Cancer Research Consortium (DKTK), Partner Site Tübingen, German Cancer Research Center (DKFZ), 69120, Heidelberg, Germany

[8]Tübingen Center for Academic Drug Discovery (TüCAD2), Auf der Morgenstelle 8, 72076, Tübingen, DE, Germany

1 DOWNSTREAM TARGET PHOSPHORYLATION ASSAY (WESTERN BLOTTING) 4
2 METABOLIC STABILITY 7
3 PHARMACOKINETIC STUDY 11
4 TDI DETERMINATION 18
5 TARGET BINDING WHOLE ENZYME LABELING MS 19
6 GSH STABILITY ASSAY 22
7 K_{INACT}/K_{I} DETERMINATION 24
8 KINOMSCREEN: SCANEDGE - KINOMESCAN™ PROFILING[5] 26
9 ADDITIONAL EVALUATED COMPOUNDS 29
10 SYNTHETIC PROCEDURES 31
10.1 General procedure A: Ullmann-type reaction with pyrazoles 31
10.2 General procedure B: Amid coupling 31
10.2.1 General Procedure B1: Using EDC HCl + HOBt 31
10.2.2 General Procedure B2: Using CDI 31
10.2.3 General Procedure B3: Using acid chlorides 31
10.2.4 General Procedure B4: Using HATU 31
10.3 General procedure C: Reduction of *N*-arylated-4-nitropyrazoles 32
10.4 General procedure D: Urea-formation with isocyanates 32
10.5 General procedure E: Boc deprotection 32
10.6 Scaffold synthesis Table 1: 33
10.7 Compound synthesis Table 1: 36
10.8 Scaffold synthesis Table 2: 44
10.9 Compound synthesis Table 2: 47
10.10 Scaffold synthesis Table 3: 49
10.11 Compound synthesis Table 3: 50
10.12 Compound synthesis additional compounds: 62
10.13 Scaffold synthesis Table 4: 66
10.14 Compound synthesis Table 4: 68
10.15 Scaffold synthesis Table 5: 70
10.16 Compound synthesis Table 5: 72
10.17 Scaffold synthesis Table 6: 74
10.18 Compound synthesis table 6: 78
10.19 Scaffold synthesis Table 7: 81
10.20 Compound synthesis Table 7: 83
10.21 Scaffold synthesis Table 8: 88
10.22 Compound synthesis Table 8: 89
11 NMR/ HPLC-ANALYTICS 91
11.1 Tabel 1 92
11.2 Table 2 105
11.3 Table 3 111
11.4 Additional Compounds 135
11.5 Table 4 147
11.6 Table 5 150
11.7 Table 6 154
11.8 Table 7 159
11.9 Table 8 169
12 SUPPLEMENTARY REFERENCES 178

Table of contents: Figures SI

FIGURE S1: TRIPLICATE P-C-JUN WB 5
FIGURE S2: TRIPLICATE P-C-JUN WB 6
FIGURE S3: METABOLIC STUDIES IN MLM 10
FIGURE S4: PK STUDIES I.V. CASSETTE DOSING 11
FIGURE S5: DOSE-RESPONSE CURVES FOR **56D** SHOWING NOTICEABLE "HOOK"-EFFECT 18
FIGURE S6: ACTIVATED JNK2 PROTEIN INCUBATED WITH DMSO FOR 4.25H. 19
FIGURE S7: ACTIVATED JNK2 PROTEIN INCUBATED WITH **56D** FOR 4.25H. 20
FIGURE S8: COMBINED CHROMATOGRAMS & SPECTRA 21
FIGURE S9: GSH STABILITY FOR AFATINIB 22
FIGURE S10: GSH STABILITY FOR **56D** 23
FIGURE S11: K_{INACT}/K_I DETERMINATION FOR **56D** ON JNK2 24
FIGURE S12: K_{INACT}/K_I DETERMINATION FOR **56D** ON JNK3 25
FIGURE S13: KINOM PROFILE OF **56D** 26

Table of contents: Schemes SI

SCHEME S1: SYNTHESIS TABLE 1 33
SCHEME S2: SYNTHESIS TABLE 2 44
SCHEME S3: SYNTHESIS TABLE 3 AND ADDITIONAL COMPOUNDS 49
SCHEME S4: SYNTHESIS TABLE 4 66
SCHEME S5: SYNTHESIS TABLE 5 70
SCHEME S6: SYNTHESIS TABLE 6 74
SCHEME S7: SYNTHESIS TABLE 7 AND TABLE 8 81

Table of contents: Table SI

TABLE S1: MEDIA AND BUFFERS USED IN WESTERN BLOTTING 4
TABLE S2: **21B** METABOLIC STABILITY IN MOUSE LIVER MICROSOMES 7
TABLE S3: **21H** METABOLIC STABILITY IN MOUSE LIVER MICROSOMES 8
TABLE S4: **51D** METABOLIC STABILITY IN MOUSE LIVER MICROSOMES 9
TABLE S5: PHARMACOKINETIC DATA OF **21B** 12
TABLE S6: PHARMACOKINETIC DATA OF **21H** 13
TABLE S7: PHARMACOKINETIC DATA OF **51A** 14
TABLE S8: PHARMACOKINETIC DATA OF **16B** 15
TABLE S9: PHARMACOKINETIC DATA OF **21P** 16
TABLE S10: PHARMACOKINETIC DATA OF **51D** 17
TABLE S11: TDI DETERMINATION 18
TABLE S12: HEPES-BUFFER FOR GSH ASSAY 22
TABLE S13: K_{INACT}/K_I DATA 24
TABLE S14: KINOM PROFILER DATA 27
TABLE S15: ADDITIONAL COMPOUNDS 29

1 Downstream target phosphorylation assay (Western blotting)

Table S1: Media and buffers used in Western Blotting

DMEM full growth medium:

Dulbeccos Modified Eagle Medium (DMEM) (gibco)	500 mL
fetal bovine serum (FBS)	50 mL
Sodium Pyruvate (100 mM) (gibco)	5 mL
Pen Strep (gibco)	5 mL
MEM Non-Essential Amino Acids (NEAA) (100x) (gibco)	5 mL

(RIPA)Lysis buffer:

1M Tris-HCl (pH = 8)	500µL
5M NaCl	300µL
Triton X-100	100µL
10% Sodium deoxycholate (protect from light)	500µL
10% SDS	100µL
Protease Inhibitor Mini pill (Roche, Ref#4693159001)	1 Tabl.
PhosSTOP (Roche)	1 Tabl.
Fill up to 10 mL with MilliQ Aq.	

Loading buffer:

1M Tris (pH = 6,8)	10ml
SDS	4,0g
Glycerol	20mL
β-Mercaptoethanol	10mL
Bromophenol blue	0.1g
Fill up to 50 mL with MilliQ Aq.	

SDS-running buffer:

Tris Base (2,5 mM)	3.02g
Glycine (19,2 mM)	14.4g
SDS (0.001%)	1g
Fill up to 1000 mL with MilliQ Aq.	

Transfer buffer:

Tris Base (2,5 mM)	3.02g
Glycin (19,2 mM)	14.4g
MeOH (15%)	150 mL
Fill up to 1000 mL with MilliQ Aq.	

Figure S1: Triplicate p-c-Jun WB

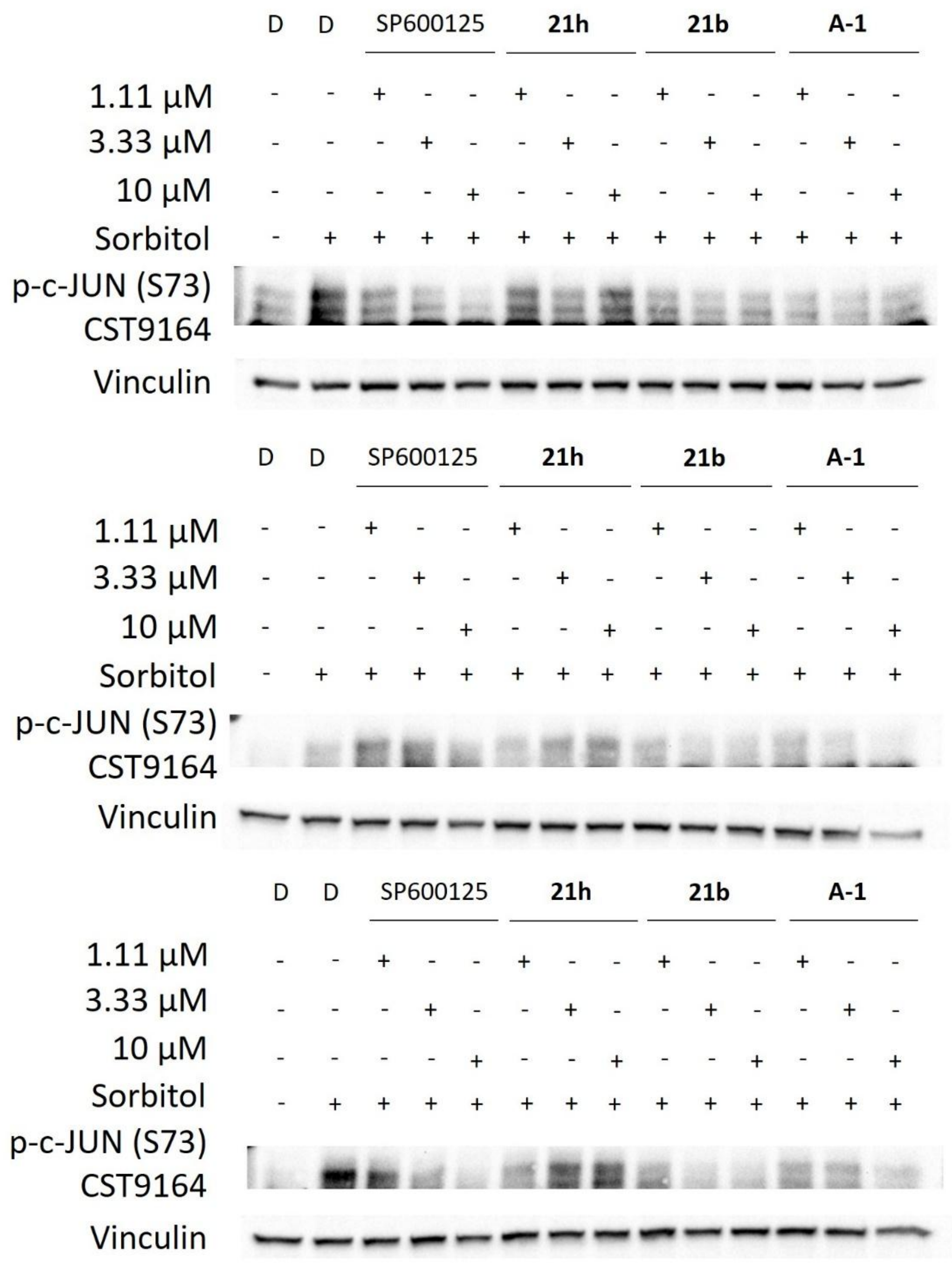

Blots:

Figure S2: Triplicate p-c-Jun WB	
**p-c-Jun (CST#9164)**	**Vinculin (Sigma Aldrich V9131)**
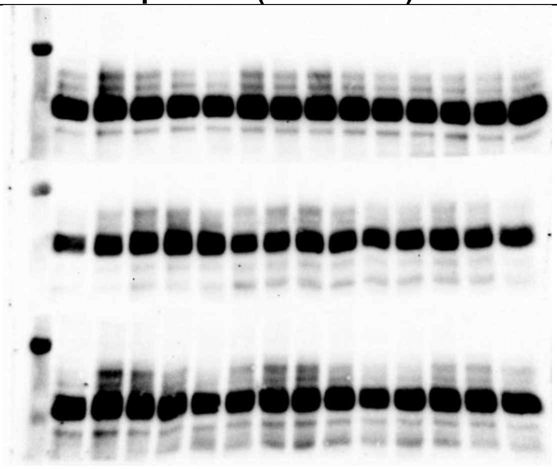	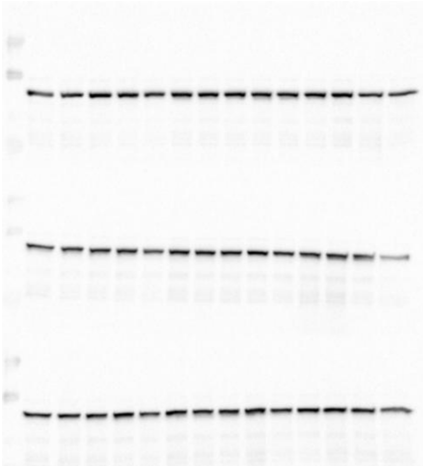

2 Metabolic stability

Table S2: **21b** metabolic stability in mouse liver microsomes

	time [min]	quantity [%]	SD [%]	1/2 SD [%]		metabolite [%]	SD [%]	1/2 SD [%]
**21b**	0	100.00	0.00	0.00	M1 *m/z*: 402.4 [+O; +16]	0.00	0.00	0.00
	10	103.89	3.05	1.52		0.75	0.17	0.08
	20	104.57	13.55	6.77		1.46	0.36	0.18
	30	99.29	7.31	3.65		1.98	0.21	0.11
	60	101.45	6.82	3.41		2.99	0.68	0.34
	120	92.60	2.09	1.04		2.85	0.18	0.09
**21b** negative control	0	100.00	n/a	n/a	M2 *m/z*:	n/a	n/a	n/a
	10	88.03	n/a	n/a		n/a	n/a	n/a
	20	100.51	n/a	n/a		n/a	n/a	n/a
	30	94.11	n/a	n/a		n/a	n/a	n/a
	60	90.43	n/a	n/a		n/a	n/a	n/a
	120	85.92	n/a	n/a		n/a	n/a	n/a
**21b** positive control	0	100.00	n/a	n/a	M3 *m/z*:	n/a	n/a	n/a
	10	83.85	n/a	n/a		n/a	n/a	n/a
	20	73.54	n/a	n/a		n/a	n/a	n/a
	30	67.12	n/a	n/a		n/a	n/a	n/a
	60	66.57	n/a	n/a		n/a	n/a	n/a
	120	57.36	n/a	n/a		n/a	n/a	n/a

other metabolites	m/z: none

Table S3: **21h** metabolic stability in mouse liver microsomes

	time [min]	quantity [%]	SD [%]	1/2 SD [%]		metabolite [%]	SD [%]	1/2 SD [%]
**21h**	0	100.00	0.00	0.00	M1 *m/z*: 428.5 [+O; +16]	0.00	0.00	0.00
	10	99.51	6.25	3.13		0.97	0.06	0.03
	20	101.46	3.92	1.96		1.31	0.18	0.09
	30	107.69	3.82	1.91		1.48	0.18	0.09
	60	102.51	3.38	1.69		1.79	0.20	0.10
	120	98.80	5.81	2.90		2.15	0.14	0.07
**21h** negative control	0	100.00	n/a	n/a	M2 *m/z*:	n/a	n/a	n/a
	10	94.20	n/a	n/a		n/a	n/a	n/a
	20	109.55	n/a	n/a		n/a	n/a	n/a
	30	109.19	n/a	n/a		n/a	n/a	n/a
	60	101.49	n/a	n/a		n/a	n/a	n/a
	120	96.66	n/a	n/a		n/a	n/a	n/a
**21h** positive control	0	100.00	n/a	n/a	M3 *m/z*:	n/a	n/a	n/a
	10	83.85	n/a	n/a		n/a	n/a	n/a
	20	73.54	n/a	n/a		n/a	n/a	n/a
	30	67.12	n/a	n/a		n/a	n/a	n/a
	60	66.57	n/a	n/a		n/a	n/a	n/a
	120	57.36	n/a	n/a		n/a	n/a	n/a

other metabolites	m/z: none

Table S4: **51d** metabolic stability in mouse liver microsomes

	time [min]	quantity [%]	SD [%]	1/2 SD [%]		metabolite [%]	SD [%]	1/2 SD [%]
**51d**	0	100.00	0.00	0.00	M1 *m/z*: 402.4 [+O; +16]	0.16	0.07	0.04
	10	92.48	2.42	1.21		0.91	0.27	0.14
	20	93.84	14.28	7.14		1.22	0.18	0.09
	30	98.19	4.53	2.27		1.57	0.02	0.01
	60	94.67	5.64	2.82		1.44	0.11	0.05
	120	90.08	8.13	4.06		1.53	0.42	0.21
**51d** negative control	0	100.00	n/a	n/a	M2 *m/z:*	n/a	n/a	n/a
	10	94.97	n/a	n/a		n/a	n/a	n/a
	20	97.74	n/a	n/a		n/a	n/a	n/a
	30	89.26	n/a	n/a		n/a	n/a	n/a
	60	95.16	n/a	n/a		n/a	n/a	n/a
	120	85.02	n/a	n/a		n/a	n/a	n/a
**51d** positive control	0	100.00	n/a	n/a	M3 *m/z*:	n/a	n/a	n/a
	10	83.85	n/a	n/a		n/a	n/a	n/a
	20	73.54	n/a	n/a		n/a	n/a	n/a
	30	67.12	n/a	n/a		n/a	n/a	n/a
	60	66.57	n/a	n/a		n/a	n/a	n/a
	120	57.36	n/a	n/a		n/a	n/a	n/a

**other metabolites**	m/z: none

Figure S3: Metabolic studies in MLM

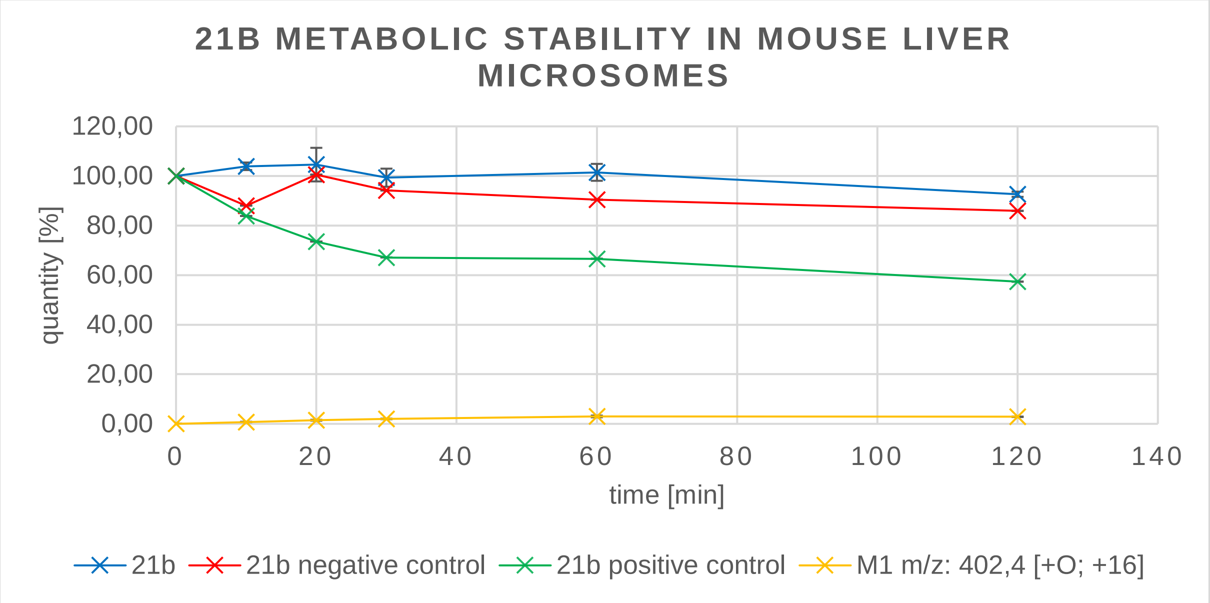

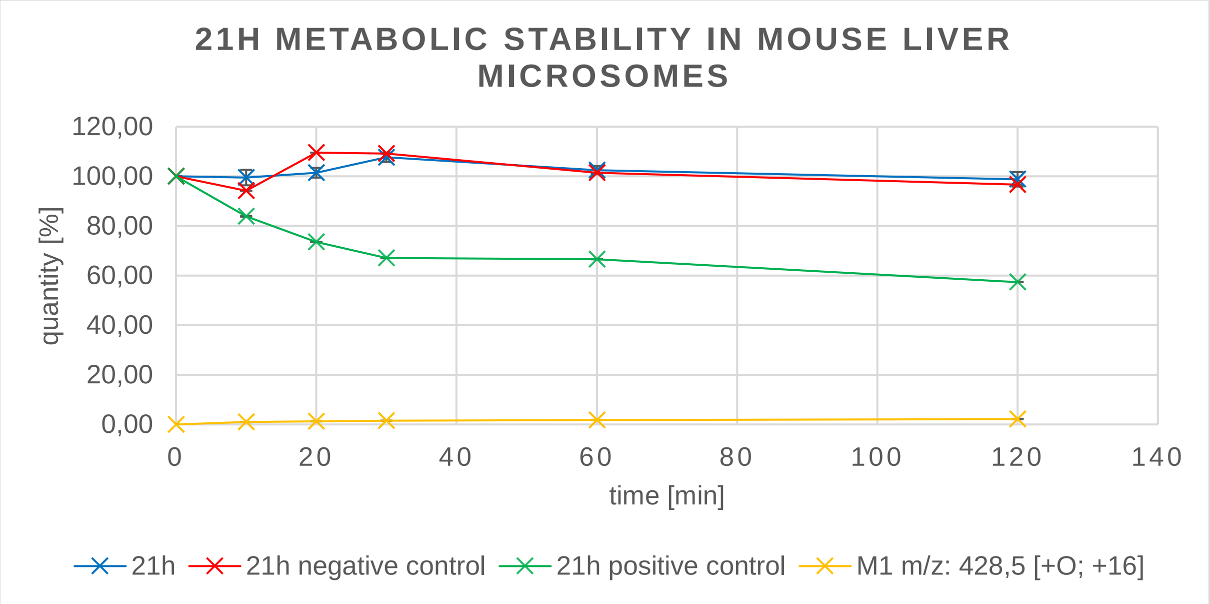

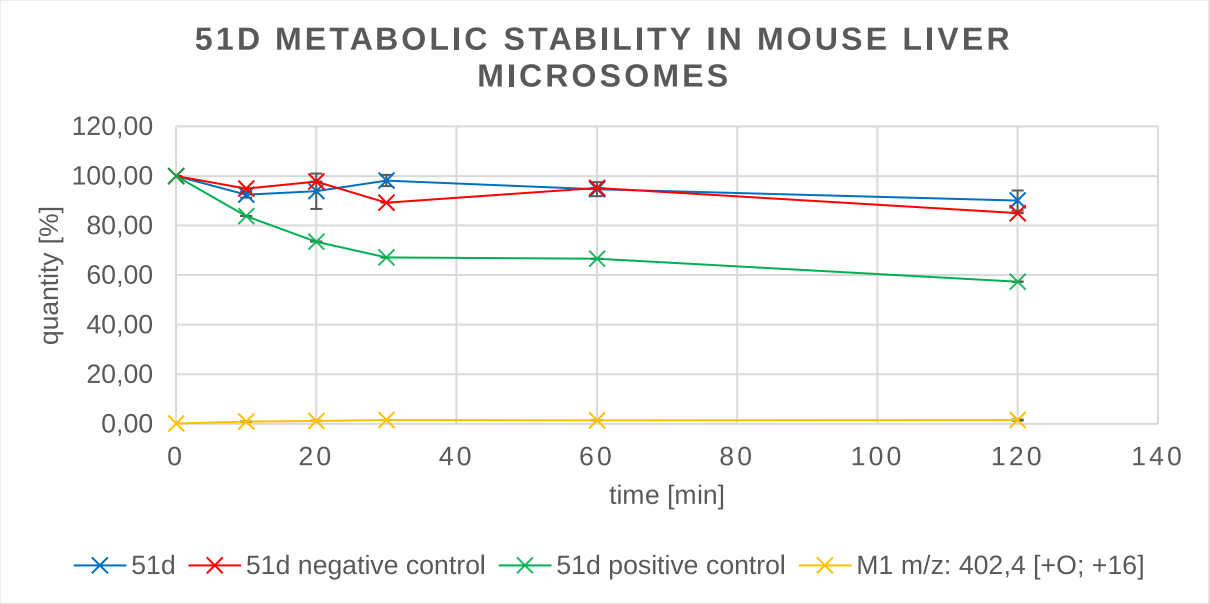

3 Pharmacokinetic study

Figure S4: PK studies i.v. cassette dosing

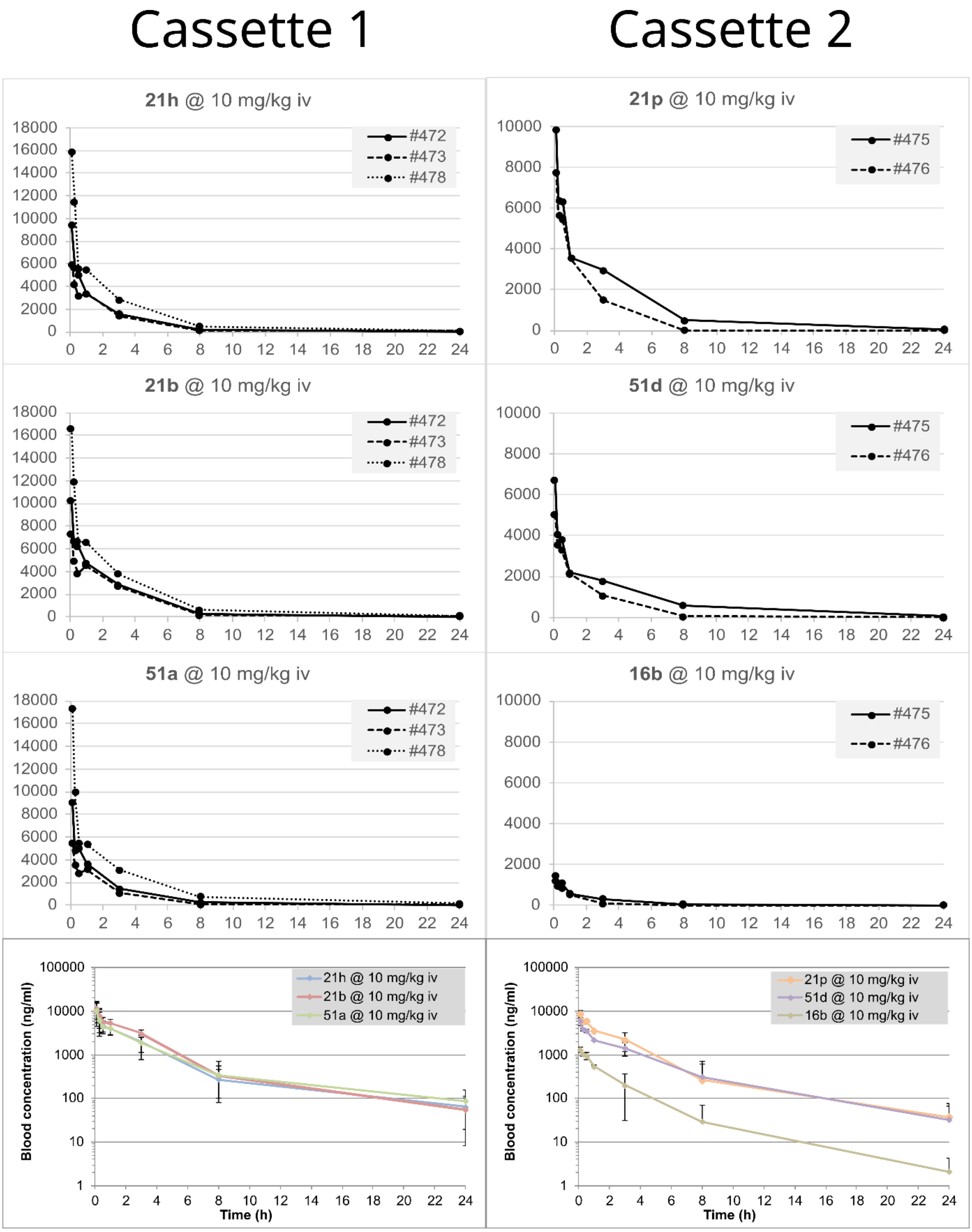

Table S5: Pharmacokinetic data of **21b**

**21b @ 10 mg/kg iv**							
**Time (h)**	**Mouse ID**	**Blood conc. [ng/ml]**	**Mean [ng/ml]**	**SD [ng/ml]**	**CV%**	**Conc [µM]**	**Manifold of IC50**
0.083	#472<br>#473<br>#478	10231.3<br>7262.6<br>16555.8	11349.9	4746.5	41.8	29.4	289.6
0.25	#472<br>#473<br>#478	6617.2<br>4897.5<br>11938.2	7817.6	3670.7	47.0	20.3	199.4
0.5	#472<br>#473<br>#478	6202.0<br>3820.8<br>6635.3	5552.7	1515.5	27.3	14.4	141.7
1	#472<br>#473<br>#478	4755.1<br>4515.6<br>6568.0	5279.6	1122.2	21.3	13.7	134.7
3	#472<br>#473<br>#478	2820.8<br>2664.4<br>3771.5	3085.6	599.2	19.4	8.0	78.7
8	#472<br>#473<br>#478	267.7<br>140.2<br>590.7	332.9	232.2	69.8	0.9	8.5
24	#472<br>#473<br>#478	6.4<br>99.7<br>59.3	55.1	46.8	84.9	0.1	1.4

IC50 (JNK2) 1.017E-07

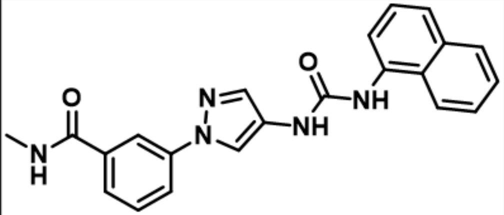

Molecular Weight: 385,43

Table S6: Pharmacokinetic data of **21h**

21h @ 10 mg/kg iv							
**Time (h)**	**Mouse ID**	**Blood conc. [ng/ml]**	**Mean [ng/ml]**	**SD [ng/ml]**	**CV%**	**Conc [µM]**	**Manifold of IC50**
0.083	#472<br>#473<br>#478	9425.5<br>5982.5<br>15827.4	10411.8	4996.0	48.0	25.3	347.6
0.25	#472<br>#473<br>#478	5702.3<br>4175.8<br>11424.6	7100.9	3821.4	53.8	17.3	237.1
0.5	#472<br>#473<br>#478	5058.0<br>3153.5<br>5595.4	4602.3	1283.2	27.9	11.2	153.7
1	#472<br>#473<br>#478	3334.7<br>3386.5<br>5450.2	4057.1	1206.7	29.7	9.9	135.5
3	#472<br>#473<br>#478	1575.2<br>1411.6<br>2842.9	1943.3	783.4	40.3	4.7	64.9
8	#472<br>#473<br>#478	231.1<br>106.2<br>481.2	272.9	191.0	70.0	0.7	9.1
24	#472<br>#473<br>#478	12.9<br>93.7<br>88.2	64.9	45.1	69.5	0.2	2.2

IC50 (JNK2) 7.279E-08

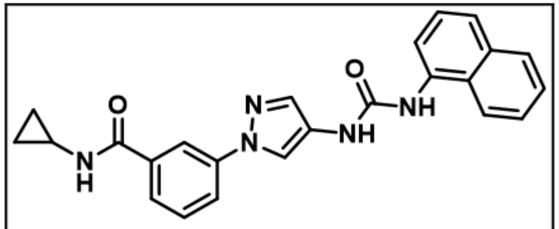

Molecular Weight: 411,47

Table S7: Pharmacokinetic data of **51a**

51a @ 10 mg/kg iv							
**Time (h)**	**Mouse ID**	**Blood conc. [ng/ml]**	**Mean [ng/ml]**	**SD [ng/ml]**	**CV%**	**Conc [µM]**	**Manifold of IC50**
0.083	#472<br>#473<br>#478	8998.0<br>5471.0<br>17348.4	10605.8	6099.7	57.5	24.4	53.5
0.25	#472<br>#473<br>#478	4830.7<br>3514.2<br>9993.6	6112.8	3424.7	56.0	14.1	30.9
0.5	#472<br>#473<br>#478	4962.2<br>2804.2<br>5487.3	4417.9	1421.9	32.2	10.2	22.3
1	#472<br>#473<br>#478	3623.1<br>3117.1<br>5322.4	4020.9	1155.2	28.7	9.3	20.3
3	#472<br>#473<br>#478	1428.3<br>1058.4<br>3106.1	1864.2	1091.3	58.5	4.3	9.4
8	#472<br>#473<br>#478	220.8<br>65.9<br>755.7	347.5	361.9	104.2	0.8	1.8
24	#472<br>#473<br>#478	23.3<br>79.8<br>157.3	86.8	67.3	77.5	0.2	0.4

IC50 (JNK2) 4.559E-07

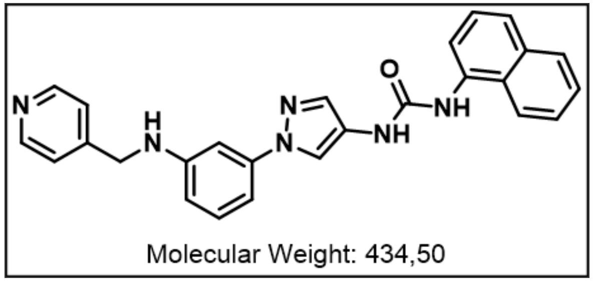

Table S8: Pharmacokinetic data of **16b**

16b @ 10 mg/kg iv							
**Time (h)**	**Mouse ID**	**Blood conc. [ng/ml]**	**Mean [ng/ml]**	**SD [ng/ml]**	**CV%**	**Conc [µM]**	**Manifold of IC50**
0.083	#475	1463.6	1330.0	189.0	14.2	4.0	12.1
	#476	1196.3					
	#477	13498.0					
0.25	#475	1088.7	1019.7	97.6	9.6	3.1	9.3
	#476	950.7					
	#477	5335.4					
0.5	#475	1073.8	943.4	184.4	19.5	2.9	8.6
	#476	813.0					
	#477	3653.5					
1	#475	571.5	541.2	42.9	7.9	1.6	4.9
	#476	510.9					
	#477	3123.2					
3	#475	316.4	198.6	166.6	83.9	0.6	1.8
	#476	80.8					
	#477	1782.0					
8	#475	57.6	29.5	39.6	134.2	0.1	0.3
	#476	1.5					
	#477	1617.5					
24	#475	3.6	2.1	2.1	100.8	0.0	0.0
	#476	0.6					
	#477	856.9					

IC50 (JNK2) 3.337E-07

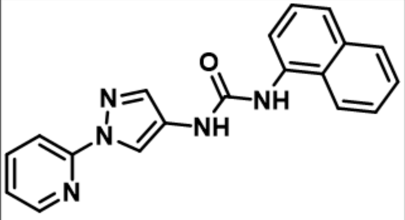

Molecular Weight: 329,36

Table S9: Pharmacokinetic data of **21p**

**21p @ 10 mg/kg iv**							
**Time (h)**	**Mouse ID**	**Blood conc. [ng/ml]**	**Mean [ng/ml]**	**SD [ng/ml]**	**CV%**	**Conc [µM]**	**Manifold of IC50**
0.083	#475	9851.3	8802.4	1483.3	16.9	21.4	170.3
	#476	7753.6					
	#477	73655.8					
0.25	#475	6352.7	5990.1	512.8	8.6	14.6	115.9
	#476	5627.5					
	#477	24114.4					
0.5	#475	6327.5	5879.1	634.1	10.8	14.3	113.8
	#476	5430.8					
	#477	14473.2					
1	#475	3568.5	3549.2	27.3	0.8	8.6	68.7
	#476	3529.9					
	#477	12368.3					
3	#475	2959.9	2233.2	1027.7	46.0	5.4	43.2
	#476	1506.4					
	#477	7939.2					
8	#475	504.1	264.0	339.6	128.7	0.6	5.1
	#476	23.8					
	#477	6561.8					
24	#475	65.6	37.2	40.1	107.7	0.1	0.7
	#476	8.9					
	#477	5378.5					

IC50 (JNK2) 1.256E-07

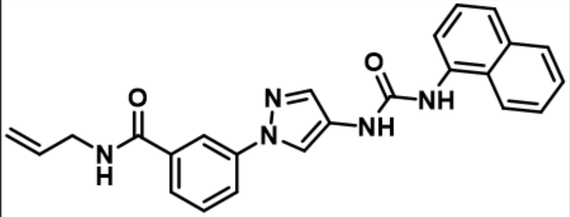

Molecular Weight: 411,47

Table S10: Pharmacokinetic data of **51d**

**51d @ 10 mg/kg iv**							
**Time (h)**	**Mouse ID**	**Blood conc. [ng/ml]**	**Mean [ng/ml]**	**SD [ng/ml]**	**CV%**	**Conc [µM]**	**Manifold of IC50**
0.083	#475	6718.9	5870.2	1200.2	20.4	15.2	51.8
	#476	5021.6					
	#477	57347.7					
0.25	#475	4047.3	3807.9	338.6	8.9	9.9	33.6
	#476	3568.5					
	#477	20156.0					
0.5	#475	3840.3	3566.4	387.3	10.9	9.3	31.5
	#476	3292.5					
	#477	13616.7					
1	#475	2198.8	2177.6	29.9	1.4	5.6	19.2
	#476	2156.4					
	#477	11361.1					
3	#475	1775.2	1422.9	498.1	35.0	3.7	12.6
	#476	1070.7					
	#477	6307.2					
8	#475	581.2	312.6	380.0	121.6	0.8	2.8
	#476	43.9					
	#477	5277.1					
24	#475	57.6	32.6	35.4	108.5	0.1	0.3
	#476	7.6					
	#477	3830.7					

IC50 (JNK2) 2.940E-07

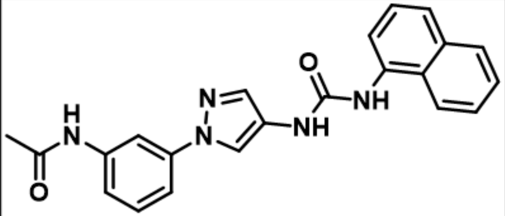

Molecular Weight: 385,43

4 TDI determination

Table S11: TDI Determination

Employing the PhosphoSens® chelation-enhanced fluorescence assay (AssayQuant Technologies, Inc.).[1]

Compound ID	IC50 (nM) - Sorted by compounds								
	ATP Km								
	4 nM JNK1			4 nM JNK2			5 nM JNK3		
	0 min Pre-incubation IC50 (nM)	60 min Pre-incubation IC50 (nM)	Fold-Change	0 min Pre-incubation IC50 (nM)	60 min Pre-incubation IC50 (nM)	Fold-Change	0 min Pre-incubation IC50 (nM)	60 min Pre-incubation IC50 (nM)	Fold-Change
56d	10000	10000	**N/A**	10000	25	**N/A**	10000	40	**N/A**
56b	10000	10000	**N/A**	10000	10000	**N/A**	10000	10000	**N/A**

Figure S5: Dose-response curves for **56d** showing noticeable "Hook"-effect

56d, JNK2

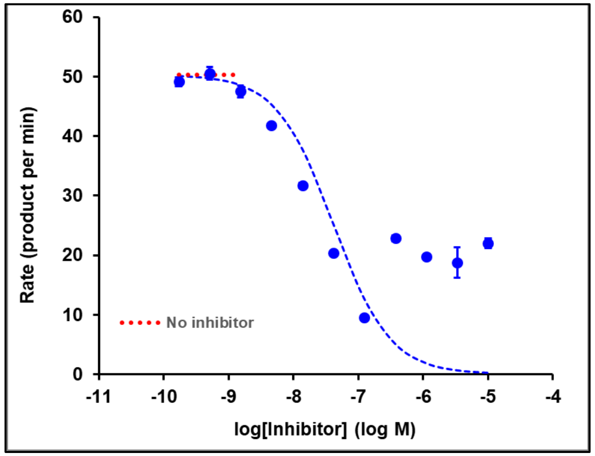

56d, JNK3

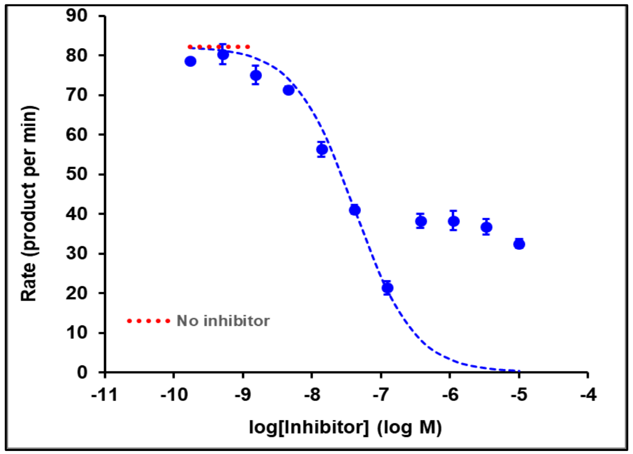

5 Target Binding whole enzyme labeling MS

Figure S6: Activated JNK2 protein incubated with DMSO for 4.25h.

Sample Name	Protein	Compound	P:C-Ratio	protein Mass (measured) [Da]	compound mass [Da]	theo. mass (protein) [Da]	detected mass (adduct) [Da]
JNK2-DMSO-VW	JNK2 recombinant Activated	-	-	multiple	-	49,613.62<br>49,708.59<br>(MonoPhos)	49,732.8<br>$[Prot+PO_4H_2+Na]^+$

Chromatogram

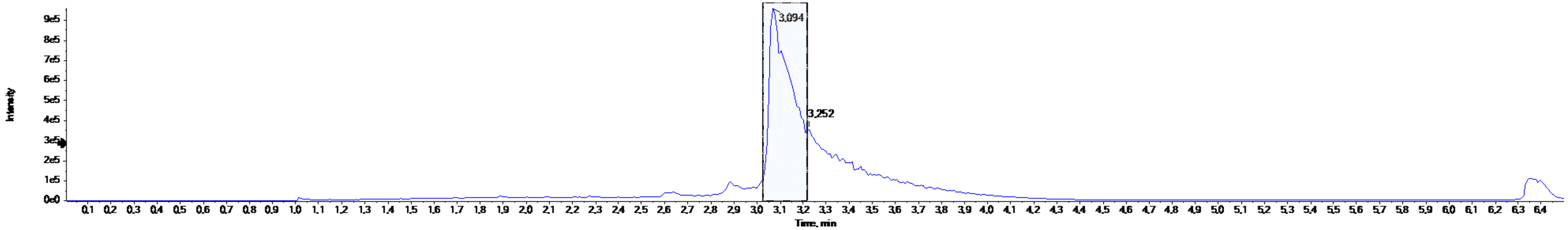

Deconvoluted spectra

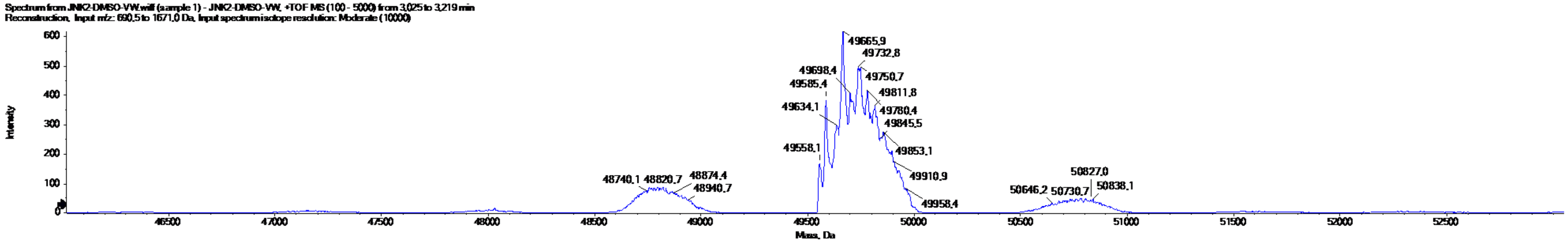

Figure S7: Activated JNK2 protein incubated with **56d** for 4.25h.

Sample Name	Protein	Compound	P:C-Ratio	protein Mass (measured) [Da]	compound mass [Da]	theo. mass (protein) [Da]	detected mass (adduct) [Da]
JNK2-56d-VW	JNK2 recombinant Activated	56d	1:5	multiple	516.1910	49,613.62<br>49,708.59<br>(MonoPhos)	multiple

Chromatogram

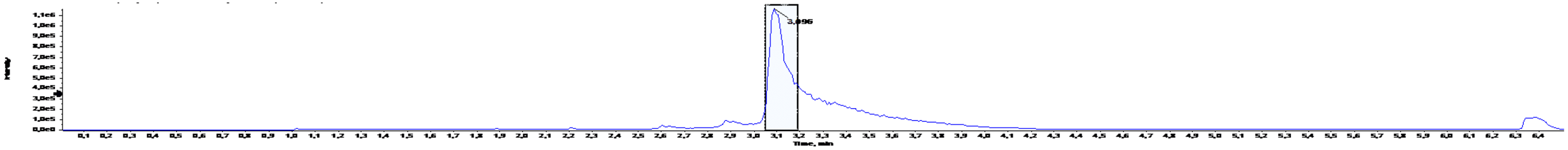

Deconvoluted spectra

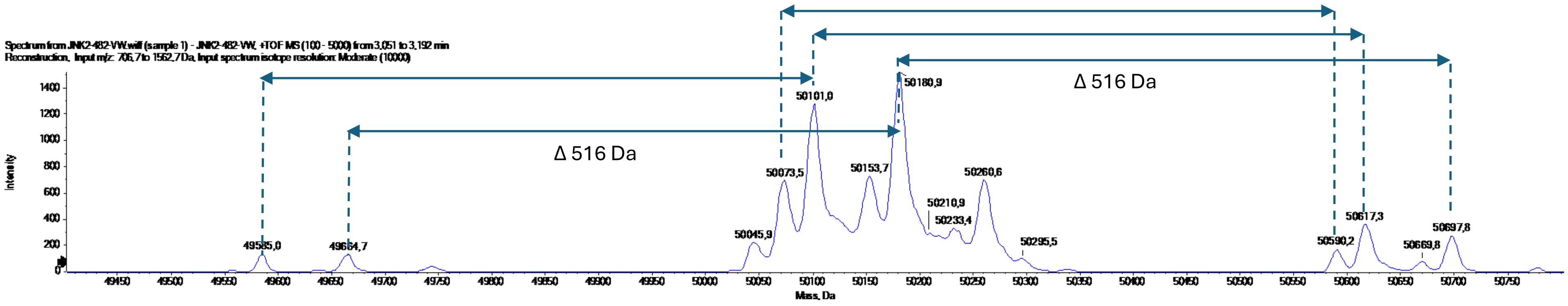

Figure S8: Combined chromatograms & spectra

Combined chromatogram

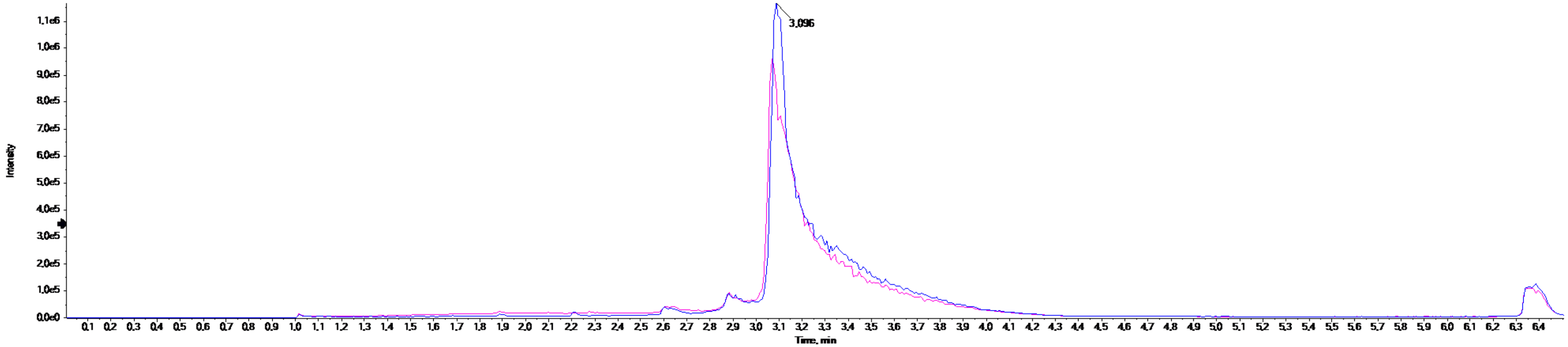

Combined spectra

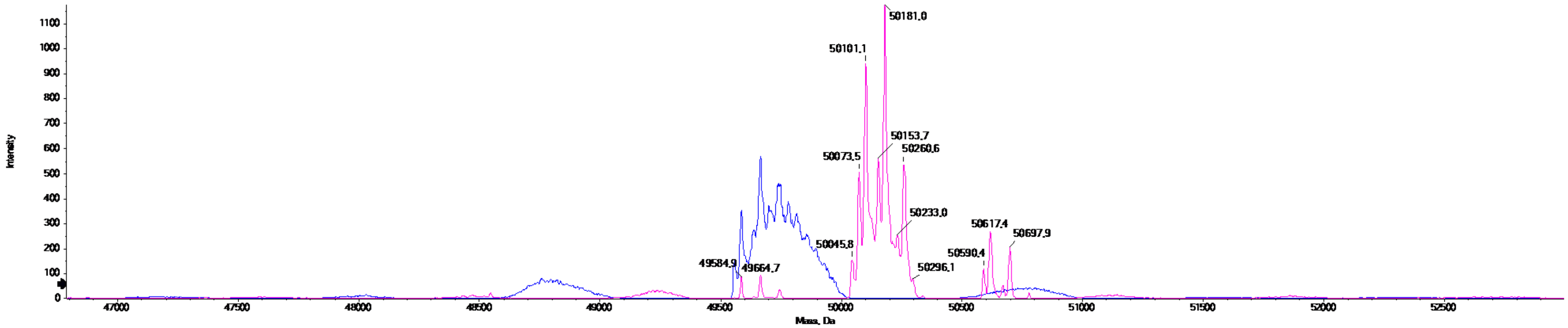

Magenta color: labeled Protein signal

Blue color: blank Protein signal

6 GSH stability assay

HEPES-Buffer (pH = 7.5)

(derived from reaction buffer used in HotSpot^{TM} assay from Reaction biology):[2]

Table S12: HEPES-Buffer for GSH assay

HEPES (pH = 7.5, adjusted)	20 mM
$MgCl_2$	10 mM
EGTA	1 mM
Brij35	0.01%
Na_3VO_4	0.1 mM
DMSO	1%

The GSH assay was performed as described previously by M. Schwarz et al. which is based on an adjusted version of an assay from Keeley et al.[3, 4]

Figure S9: GSH stability for Afatinib

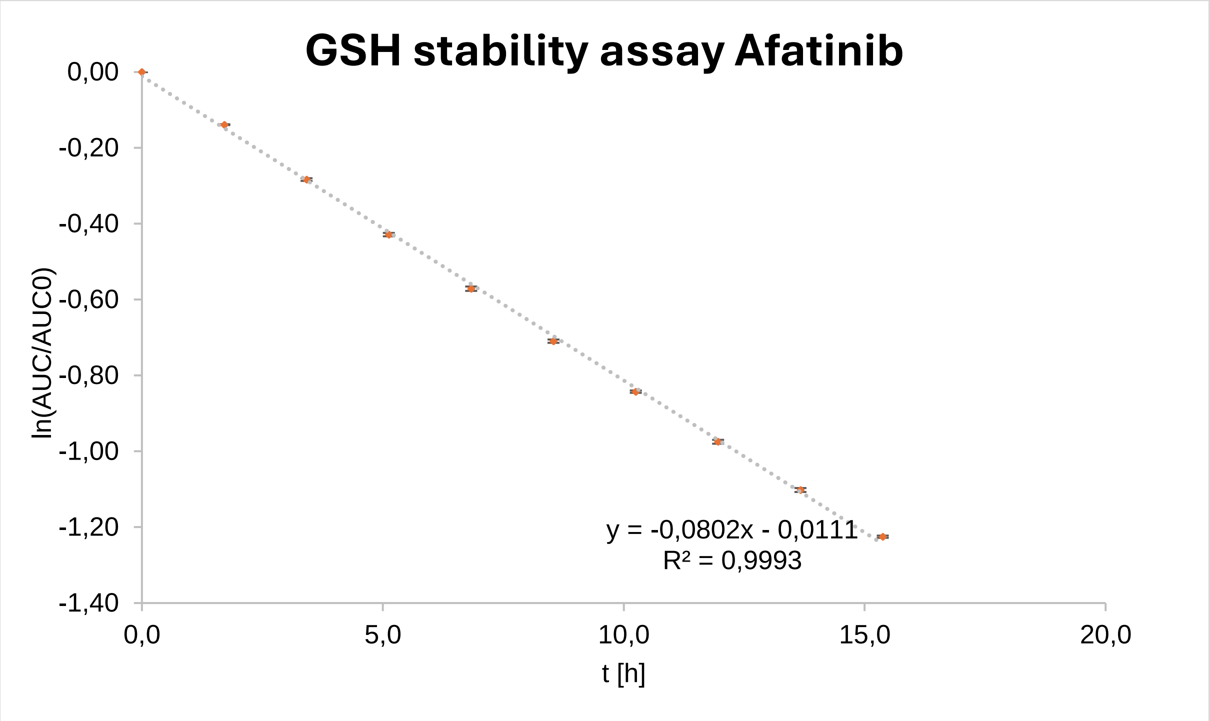

Linear regression formula: $y = -0.0802x - 0.0111$

Reaction rate k: $\ln\left(\frac{AUC}{AUC_0}\right) = k * t\,; k_{(Afatinib)} = 0.0802\frac{1}{h}$

Half-life ($t_{1/2}$): $t_{1/2} = \frac{ln2}{k}; t_{1/2\,(Afatinib)} = \frac{ln2}{0.0802\frac{1}{h}} = 8.64\,h$

Figure S10: GSH stability for **56d**

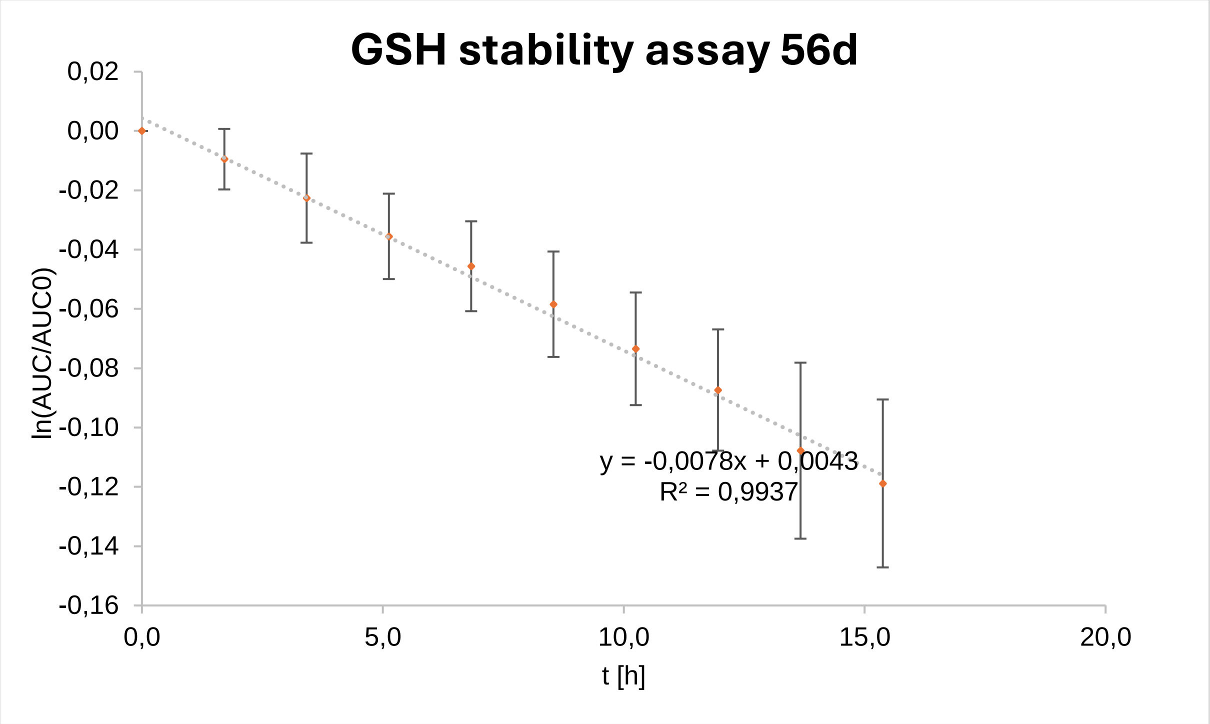

Linear regression formula: $y = -0.0078x - 0.0043$

Reaction rate k: $\ln\left(\frac{AUC}{AUC_0}\right) = k * t\,;k_{(56d)} = 0.0078\frac{1}{h}$

Half-life ($t_{1/2}$): $t_{1/2} = \frac{ln2}{k}; t_{1/2\,(56d)} = \frac{ln2}{0.0078\frac{1}{h}} = 88.87\,h$

7 k_{inact}/K_I determination

Table S13: k_{inact}/K_I data

Compound	Enzyme Target	Enzyme conc. (nM)	ATP (µM)	Sensor (µM)	1- or 2-step	k_{inact} / K_I (M-1sec-1)		k_{inact} (sec-1)		K_I app (µM)		R^2
						Fitted value	Std. error	Fitted value	Std. error	Fitted value	Std. error	
**56d**	**JNK2**	1.0	19.4	20	2-step	38,200	470	0.000113	0.0000004	0.00297	0.00004	0.9745
**56d**	**JNK3**	1.0	13.1	20	2-step	70,100	1,500	0.000124	0.0000007	0.00177	0.00004	0.9533

Figure S11: k_{inact}/K_I determination for **56d** on JNK2

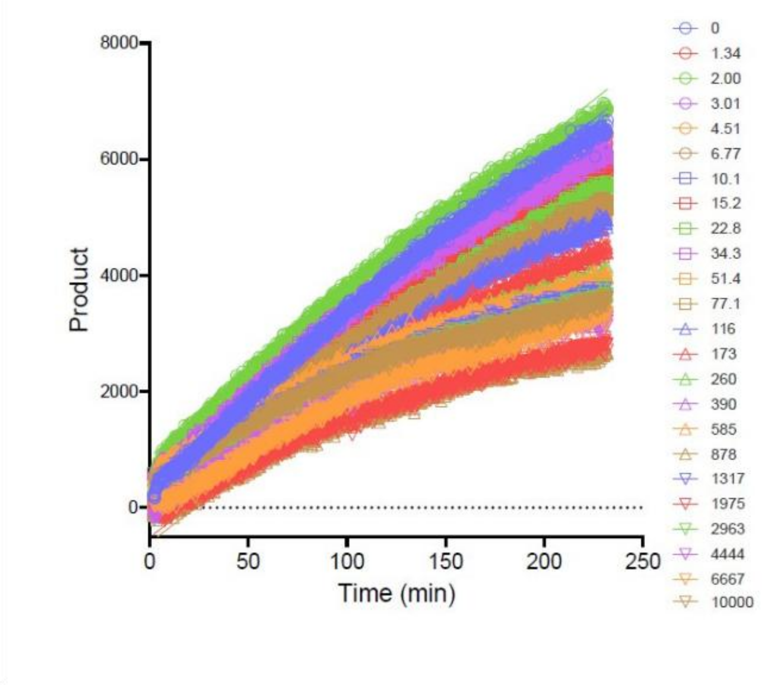

Preferred model	Two-step KINACT / KI

Comparison of Fits	
Null hypothesis	One-step KINACT / KI
Alternative hypothesis	Two-step KINACT / KI
P value	<0.0001
Conclusion (alpha = 0.05)	Reject null hypothesis
Preferred model	Two-step KINACT / KI
F (DFn, DFd)	71595 (1, 55269)

One-step KINACT / KI	
Best-fit values	
A	1041
I	= 0.000
S	= 1000
KM	= 19.40
BG	1022
V0	23.00
Kinact / Ki	1041
Std. Error	
A	8.798
BG	8.732
V0	0.02947
Kinact / Ki	8.798
R squared	0.9415
Sum of Squares	8194819604

Two-step KINACT / KI	
Best-fit values	
KINACT	0.0001134
I	= 0.000
KI	2.969e-009
S	= 1000
KM	= 19.40
BG	489.0
V0	27.55
Kinact / KI	38187
Std. Error	
KINACT	4.257e-007
KI	4.004e-011
BG	6.420
V0	0.03102
Kinact / KI	468.6
R squared	0.9745
Sum of Squares	3570127328

Figure S12: k_{inact}/K_I determination for **56d** on JNK3

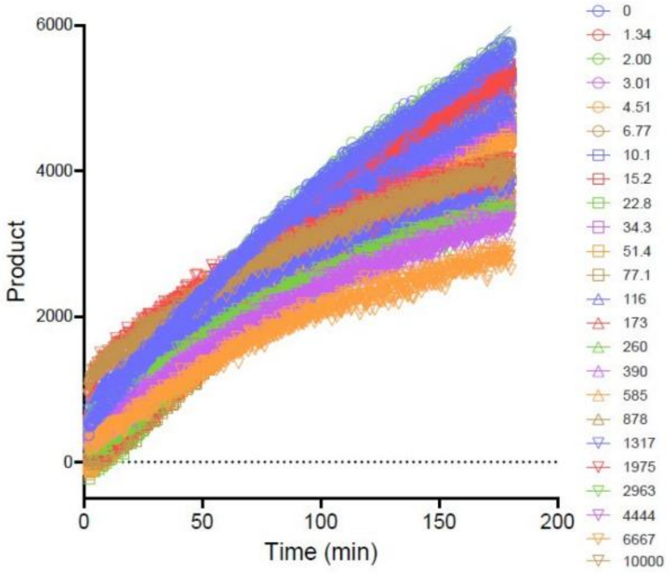

Preferred model	Two-step KINACT / KI

Comparison of Fits	
Null hypothesis	One-step KINACT / KI
Alternative hypothesis	Two-step KINACT / KI
P value	<0.0001
Conclusion (alpha = 0.05)	Reject null hypothesis
Preferred model	Two-step KINACT / KI
F (DFn, DFd)	23553 (1, 42789)

One-step KINACT / KI	
Best-fit values	
A	1640
I	= 0.000
S	= 1000
KM	= 13.10
BG	1161
V0	24.60
Kinact / Ki	1640

Std. Error	
A	17.94
BG	8.938
V0	0.03860
Kinact / Ki	17.94

R squared	0.9276
Sum of Squares	5153546515

Two-step KINACT / KI	
Best-fit values	
KINACT	0.0001240
I	= 0.000
KI	1.768e-009
S	= 1000
KM	= 13.10
BG	759.4
V0	29.00
Kinact / KI	70131

Std. Error	
KINACT	7.371e-007
KI	3.986e-011
BG	8.040
V0	0.05040
Kinact / KI	1469

R squared	0.9533
Sum of Squares	3323906460

8 Kinomscreen: scanEDGE - KINOMEscan™ Profiling[5]

Figure S13: Kinom profile of **56d**

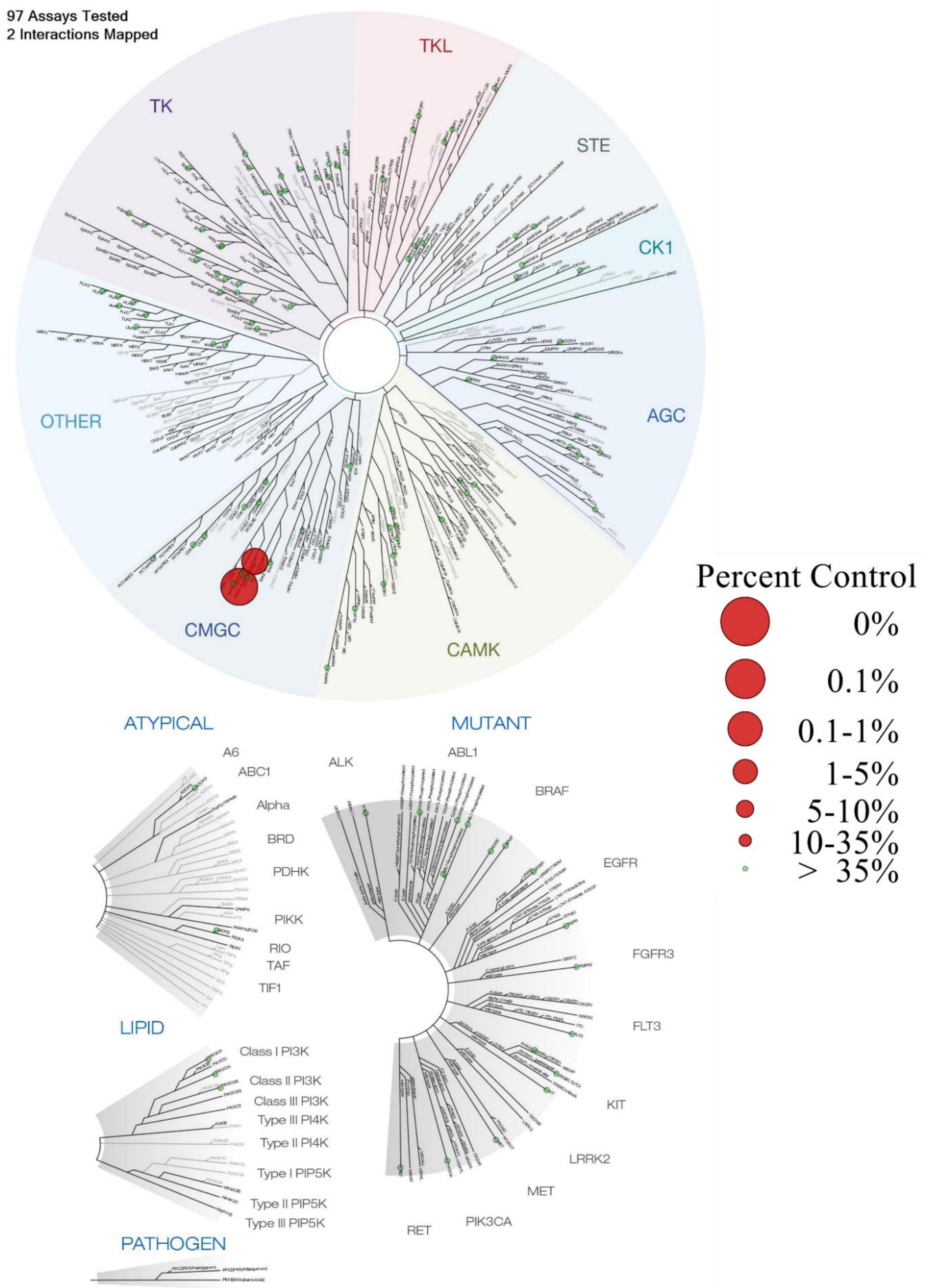

Image generated using TREE*spot*™ Software Tool and reprinted with permission from KINOMEscan®, a division of DiscoveRx Corporation, © DISCOVERX CORPORATION 2010.

Table S14: Kinom profiler data

Kinase	POC @ 500 nM (inhibitor 56d)
**ABL1(E255K)-phosphorylated**	65
**ABL1(T315I)-phosphorylated**	100
**ABL1-nonphosphorylated**	45
**ABL1-phosphorylated**	88
**ACVR1B**	80
**ADCK3**	98
**AKT1**	100
**AKT2**	100
**ALK**	100
**AURKA**	100
**AURKB**	100
**AXL**	87
**BMPR2**	100
**BRAF**	100
**BRAF(V600E)**	100
**BTK**	100
**CDK11**	100
**CDK2**	98
**CDK3**	100
**CDK7**	100
**CDK9**	98
**CHEK1**	99
**CSF1R**	100
**CSNK1D**	88
**CSNK1G2**	93
**DCAMKL1**	96
**DYRK1B**	93
**EGFR**	91
**EGFR(L858R)**	93
**EPHA2**	100
**ERBB2**	47
**ERBB4**	82
**ERK1**	99
**FAK**	100
**FGFR2**	64
**FGFR3**	99
**FLT3**	93
**GSK3B**	100
**IGF1R**	92
**IKK-alpha**	100
**IKK-beta**	100
**INSR**	100
**JAK2(JH1domain-catalytic)**	100
**JAK3(JH1domain-catalytic)**	100
**JNK1**	65
**JNK2**	1.4
**JNK3**	0.3
**KIT**	89
**KIT(D816V)**	100
**KIT(V559D,T670I)**	100
**LKB1**	100

**MAP3K4**	92
**MAPKAPK2**	100
**MARK3**	100
**MEK1**	95
**MEK2**	95
**MET**	81
**MKNK1**	100
**MKNK2**	100
**MLK1**	86
**p38-alpha**	100
**p38-beta**	93
**PAK1**	97
**PAK2**	100
**PAK4**	91
**PCTK1**	100
**PDGFRA**	100
**PDGFRB**	84
**PDPK1**	100
**PIK3C2B**	100
**PIK3CA**	100
**PIK3CG**	100
**PIM1**	95
**PIM2**	100
**PIM3**	84
**PKAC-alpha**	100
**PLK1**	100
**PLK3**	100
**PLK4**	100
**PRKCE**	42
**RAF1**	62
**RET**	100
**RIOK2**	100
**ROCK2**	100
**RSK2(Kin.Dom.1-N-terminal)**	100
**SNARK**	100
**SRC**	100
**SRPK3**	100
**TGFBR1**	70
**TIE2**	100
**TRKA**	100
**TSSK1B**	100
**TYK2(JH1domain-catalytic)**	100
**ULK2**	100
**VEGFR2**	96
**YANK3**	100
**ZAP70**	85

9 Additional evaluated Compounds

Table S15: Additional Compounds

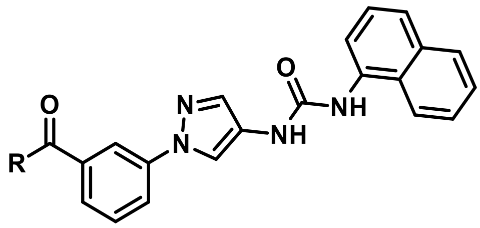

	R	JNK1 IC_{50} [nM]	JNK2 IC_{50} [nM]	JNK3 IC_{50} [nM]	Selectivity (IC_{50} JNK1/ IC_{50} JNK2)
**SI-I**	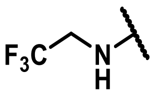	**13390**	**9598**	**9948**	**1**
**SI-II**	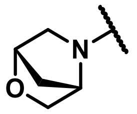	**35050**	**4545**	**6069**	**8**
**SI-III**	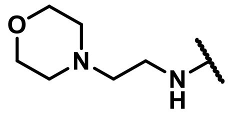	**1379**	**115**	**126**	**12**
**SI-IV**	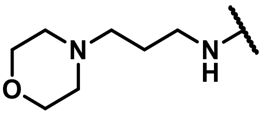	**659**	**49**	**66**	**13**
**SI-V**	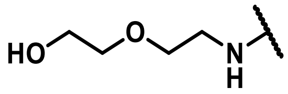	**5333**	**229**	**235**	**23**
**SI-VI**	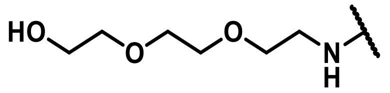	**8966**	**533**	**418**	**17**

3-(4-(3-(naphthalen-1-yl)ureido)-1H-pyrazol-1-yl)-N-(2,2,2-trifluoroethyl)benzamide ***(SI-I)***. (59 mg; 69%) ^{1}H NMR (400 MHz, DMSO) δ 9.31 (t, J = 6.1 Hz, 1H), 9.02 (s, 1H), 8.89 (s, 1H), 8.62 (s, 1H), 8.30 (s, 1H), 8.13 (d, J = 8.3 Hz, 1H), 8.07 – 8.00 (m, 2H), 7.94 (d, J = 7.9 Hz, 1H), 7.88 (s, 1H), 7.80 (d, J = 7.7 Hz, 1H), 7.66 – 7.53 (m, 4H), 7.48 (t, J = 7.9 Hz, 1H), 4.14 (qd, J = 9.7, 6.6 Hz, 2H); ^{13}C NMR (101 MHz, DMSO) δ 166.2, 152.8, 139.9, 134.5, 134.4, 133.7, 133.2, 129.9, 128.4, 126.0, 125.9, 125.9, 125.7, 124.8, 124.8 (q, J = 279.4 Hz),124.8, 123.0, 121.4, 121.0, 117.4, 116.4, 40.8 – 39.7 (m); FTIR [cm^{-1}]: 3252, 3069, 2958, 1644, 1585, 1545, 1485, 1395, 1319, 1262; TLC-MS(ESI) *m/z*: 452.1 [M-H]-; 488.1 [M+Cl]$^{-}$; HRMS(ESI) *m/z*: calcd. for [M+H]$^{+}$: 454.14846; found: 454.1492; HPLC t_{ret}: 7.94 min.

1-(1-(3-((1S,4S)-2-oxa-5-azabicyclo[2.2.1]heptane-5-carbonyl)phenyl)-1H-pyrazol-4-yl)-3-(naphthalen-1-yl)urea ***(SI-II)***. (46 mg; 63%) ^{1}H NMR (400 MHz, DMSO) δ 9.01 (d, J = 3.9 Hz, 1H), 8.89 (s, 1H), 8.56 (s, 1H), 8.13 (d, J = 8.4 Hz, 1H), 8.04 (d, J = 7.6 Hz, 1H), 7.96 – 7.85 (m, 4H), 7.64 (d, J = 8.2 Hz, 1H), 7.62 – 7.51 (m, 3H), 7.48 (t, J = 7.9 Hz, 1H), 7.39 (dd, J = 20.3, 7.6 Hz, 1H), 4.77 (d, J = 74.9 Hz, 1H), 4.48 (d, J = 75.2 Hz, 1H), 3.89 (dd, J = 24.2, 7.3 Hz, 1H), 3.73 (ddd, J = 33.8, 7.5, 1.2 Hz, 1H), 3.57 – 3.48 (m, 1H), 3.31 (dd, J = 32.8, 10.8 Hz, 1H), 1.93 (ddd, J = 20.8, 10.0, 1.6 Hz, 1H), 1.80 (dd, J = 31.8, 9.9 Hz, 1H); ^{13}C NMR (101 MHz, DMSO) δ 167.9, 167.0, 152.8, 139.8, 139.7, 137.7, 137.3, 134.5, 133.7, 133.4, 133.3, 133.3, 129.8, 129.7, 129.1, 128.8, 128.4, 126.0, 125.9, 125.9, 125.7, 124.8, 124.7, 124.3, 124.1, 122.9, 121.4, 119.3, 119.0, 117.3, 117.2, 116.5, 116.5, 116.4, 116.3, 75.6, 75.1, 73.6, 73.0, 59.9, 57.7, 56.2, 54.3, 36.7, 35.0 (doubling of signals probably because of rotational isomers); FTIR [cm^{-1}]: 3292, 3053, 2950, 2876, 1701, 1542, 1465, 1388, 1251, 1206; TLC-MS(ESI) *m/z*: 475.8 [M+Na]$^{+}$; 451.8 [M-H]$^{-}$; 487.8 [M+Cl]$^{-}$; HRMS(ESI) *m/z*: calcd. for [M+H]$^{+}$: 454.18729; found: 454.1881; HPLC t_{ret}: 6.70 min.

N-(2-morpholinoethyl)-3-(4-(3-(naphthalen-1-yl)ureido)-1H-pyrazol-1-yl)benzamide ***(SI-III)***. (14 mg; 27%). ^{1}H NMR (400 MHz, DMSO) δ 9.02 (s, 1H), 8.89 (s, 1H), 8.64 (t, J = 5.6 Hz, 1H), 8.59 (s, 1H), 8.26 – 8.21 (m, 1H), 8.12 (d, J = 8.3 Hz, 1H), 8.03 (d, J = 7.3 Hz, 1H), 7.99 – 7.92 (m, 2H), 7.86 (s, 1H), 7.74 (d, J = 7.8 Hz, 1H), 7.65 (d, J = 8.2 Hz, 1H), 7.62 – 7.53 (m, 3H), 7.48 (t, J = 7.9 Hz, 1H), 3.58 (t, 4H), 3.42 (dd, J = 13.0, 6.6 Hz, 2H), 2.49 – 2.46 (m, 2H), 2.45 – 2.39 (m, 4H); ^{13}C NMR (101 MHz, DMSO) δ 165.4, 152.8, 139.8, 135.8, 134.4, 133.7, 133.1, 129.6, 128.4, 126.0, 125.9, 125.9, 125.7, 124.8, 124.5, 123.0, 121.4, 120.3, 117.3, 116.4, 116.2, 66.2, 57.4, 53.3, 36.7; FTIR [cm^{-1}]: 3259, 3053, 2926, 2851, 2810, 1636, 1583, 1540, 1487, 1388; TLC-MS(ESI) *m/z*: 484.8 [M+Na]$^{+}$; 506.9 [M+Na]$^{+}$; 482.8 [M-H]$^{-}$; 518.6 [M+Cl]$^{-}$; HRMS(ESI) *m/z*: calcd. for [M+H]$^{+}$: 485.22949; found: 485.2292; HPLC t_{ret}: 4.91 min.

N-(3-morpholinopropyl)-3-(4-(3-(naphthalen-1-yl)ureido)-1H-pyrazol-1-yl)benzamide ***(SI-IV)***. (16 mg; 26%) ^{1}H NMR (400 MHz, DMSO) δ 9.00 (s, 1H), 8.87 (s, 1H), 8.68 (t, J = 5.5 Hz, 1H), 8.59 (s, 1H), 8.25 – 8.20 (m, 1H), 8.12 (d, J = 8.4 Hz, 1H), 8.03 (dd, J = 7.6, 0.8 Hz, 1H), 7.98 – 7.91 (m, 2H), 7.86 (s, 1H), 7.74 (d, J = 7.9 Hz, 1H), 7.65 (d, J = 8.2 Hz, 1H), 7.62 – 7.53 (m, 3H), 7.48 (t, J = 7.9 Hz, 1H), 3.60 – 3.54 (m, 4H), 3.38 – 3.25 (m, 2H), 2.43 – 2.27 (m, 6H), 1.77 – 1.66 (m, 2H); ^{13}C NMR (101 MHz, DMSO) δ 165.4, 152.8, 139.8, 135.9, 134.4, 133.7, 133.1, 129.6, 128.4, 126.0, 125.9, 125.9, 125.7, 124.7, 124.5, 123.0, 121.4, 120.2, 117.3, 116.4, 116.2, 66.2, 56.1, 53.4, 37.9, 26.0; FTIR [cm^{-1}]: 3297, 3056, 2924, 2851, 2810, 1629, 1582, 1540, 1488, 1388; TLC-MS(ESI) *m/z*: 498.9 [M+H]$^{+}$; 520.6 [M+Na]$^{+}$; 496.7 [M-H]$^{-}$; 532.7 [M+Cl]$^{-}$; HRMS(ESI) *m/z*: calcd. for [M+H]$^{+}$: 499.24514; found: 499.2429; HPLC t_{ret}: 5.01 min.

N-(2-(2-hydroxyethoxy)ethyl)-3-(4-(3-(naphthalen-1-yl)ureido)-1H-pyrazol-1-yl)benzamide ***(SI-V)***. (48 mg; 56%) ^{1}H NMR (400 MHz, DMSO) δ 8.99 (s, 1H), 8.87 (s, 1H), 8.73 (t, J = 5.5 Hz, 1H), 8.60 (s, 1H), 8.28 – 8.23 (m, 1H), 8.12 (d, J = 8.4 Hz, 1H), 8.04 (dd, J = 7.6, 0.7 Hz, 1H), 7.97 (ddd, J = 8.1, 2.2, 0.8 Hz, 1H), 7.95 – 7.91 (m, 1H), 7.87 (s, 1H), 7.78 – 7.73 (m, 1H), 7.65 (d, J = 8.2 Hz, 1H), 7.62 – 7.53 (m, 3H), 7.48 (t, J = 7.9 Hz, 1H), 4.60 (t, J = 5.3 Hz, 1H), 3.57 (t, J = 6.0 Hz, 2H), 3.54 – 3.45 (m, 6H); ^{13}C NMR (101 MHz, DMSO) δ 165.6, 152.8, 139.8, 135.7, 134.4, 133.7, 133.1, 129.6, 128.4, 126.0, 125.9, 125.9, 125.7, 124.7, 124.5, 123.0, 121.4, 120.3, 117.4, 116.4, 116.3, 72.2, 68.9, 60.2, 39.4; FTIR [cm^{-1}]: 3265, 2925, 2857, 1639, 1586, 1539, 1484, 1388, 1342, 1307; TLC-MS(ESI) *m/z*: 484.2 [M+Na]$^{+}$; 458.0 [M-H]$^{-}$; 494.1 [M+Cl]$^{-}$; HRMS(ESI) *m/z*: calcd. for [M+H]$^{+}$: 460.19785; found: 460.1982; HPLC t_{ret}: 6.66 min.

N-(2-(2-(2-hydroxyethoxy)ethoxy)ethyl)-3-(4-(3-(naphthalen-1-yl)ureido)-1H-pyrazol-1-yl)benzamide ***(SI-VI)***. (72 mg; 76%). ^{1}H NMR (400 MHz, DMSO) δ 8.99 (s, 1H), 8.87 (s, 1H), 8.75 (t, J = 5.5 Hz, 1H), 8.60 (s, 1H), 8.27 – 8.23 (m, 1H), 8.12 (d, J = 8.4 Hz, 1H), 8.06 – 8.02 (m, 1H), 7.97 (dd, J = 8.1, 1.3 Hz, 1H), 7.95 – 7.92 (m, 1H), 7.86 (s, 1H), 7.76 (d, J = 7.8 Hz, 1H), 7.65 (d, J = 8.2 Hz, 1H), 7.62 – 7.53 (m, 3H), 7.48 (t, J = 7.9 Hz, 1H), 4.57 (t, J = 5.5 Hz, 1H), 3.58 – 3.51 (m, 6H), 3.43 (ddd, J = 10.6, 8.4, 3.4 Hz, 6H). ^{13}C NMR (101 MHz, DMSO) δ 165.5, 152.8, 139.8, 135.7, 134.4, 133.7, 133.1, 129.6, 128.4, 126.0, 125.9, 125.9, 125.7, 124.7, 124.5, 123.0, 121.4, 120.3, 117.3, 116.4, 116.2, 72.4, 69.7, 69.7, 68.9, 60.2.(one aliphatic signal below DMSO-peak); DEPT: ^{13}C NMR (101 MHz, DMSO) δ 133.1, 129.6, 128.4, 125.9, 125.9, 125.7, 124.6, 123.0, 121.4, 120.3, 117.3, 116.4, 116.2, 72.4, 69.7, 69.7, 68.9, 60.2, 39.3; FTIR [cm^{-1}]: 3274, 2922, 2861, 1638, 1584, 1543, 1487, 1388, 1344, 1216; TLC-MS(ESI) *m/z*: 526.3 [M+Na]$^{+}$; 502.3 [M-H]$^{-}$; 538.2 [M+Cl]$^{-}$; HRMS(ESI) *m/z*: calcd. for [M+H]$^{+}$: 504.22407; found: 504.2245; HPLC t_{ret}: 6.69 min.

10 Synthetic procedures

General Information:

10.1 General procedure A: Ullmann-type reaction with pyrazoles

In an appropriate Schlenk flask 4-nitro-1*H*-pyrazole (1 eq.) was combined with the corresponding aryl halide (0.85-1.5 eq.) and dry Cs_2CO_3/K_2CO_3 (3 eq.). The components were suspended in dry DMF and the vessel was then alternately evacuated and flushed with argon for three consecutive times. In the argon countercurrent, Cu(I)I (0.1 - 0.3 eq.) was introduced as well as *trans*-*N*,*N'*-dimethylcyclohexane-1,2-diamine (0.2 – 0.4 eq.). The reaction was then sealed and heated up to 90-100 °C until total consumption of the pyrazole or until the reaction was terminated. The reaction was carefully quenched with 10% HCl (aq.) and water. Precipitated product was filtered, rinsed with more 10% HCl (aq.) or water and then dried in a convection oven. If the product did not precipitate, the acidified organic phase was extracted 3x with EtOAc, dried over Na_2SO_4, filtered and then purified further.

10.2 General procedure B: Amid coupling

10.2.1 General Procedure B1: Using EDC HCl + HOBt

The corresponding carboxylic acid (1 eq.) as well as HOBt (monohydrate) containing 14-20 wt.% water (2 eq.) and EDC HCl (2 eq.) were put into an appropriate vessel and were dissolved in dry DCM, THF or DMF. Subsequently DIPEA (3-6 eq.) was added while the reaction was cooled via ice bath. After 5-20 min the amine (1-2 eq.) was added and the cooling was removed. After full conversion the reaction was quenched with water and precipitated product was filtered of. If no product was precipitating the mixture was extracted with EtOAc. The combined organic phases were dried over Na_2SO_4, filtered and evaporated to dryness. If required the product was purified via flash chromatography (MeOH/DCM).

10.2.2 General Procedure B2: Using CDI

The corresponding carboxylic acid (1 eq.) as well as CDI (1.1-2 eq.) were placed in an appropriate flask and were dissolved in dry THF at RT. The acid activation was now monitored after a few hours via taking a sample and combining it with isobutyl amine. The sample was then subjected to TLC-MS. If the isopentyl amine derivative was detectable and quantitatively present, the corresponding amine (2.0-3.0 equiv) was added to the reaction and the mixture was stirred overnight. The obtained product was further purified.

10.2.3 General Procedure B3: Using acid chlorides

The corresponding amines (1 eq.) were placed in an appropriate flask and dissolved in Pyridine or DMF (in case of DMF, pyridine was added also (0.85 eq.). While cooling the reaction, the fitting acyl chloride (1 eq.) was added slowly via syringe. The reaction was stirred for 5 min, after which the reaction was allowed to slowly come up to RT. The reaction stirred at RT until full conversion. The obtained product was purified further via flash chromatography or other work-ups.

10.2.4 General Procedure B4: Using HATU

The corresponding carboxylic acid (1-1.2 eq.) as well as HATU (1.25-2 eq.) were placed in an appropriate flask and dissolved in dry DMF. DIPEA (3–5 equiv) was added to the solution. Depending on the carboxylic acid component used, the reaction was allowed to either stir for 30–60 minutes or overnight, or the amine component was added directly. Subsequently the

corresponding amine (2 eq.) was added and reaction was continued to stir overnight or until full conversion. The mixture was then quenched with demin H_2O. If product precipitated, it was filtered off and dried. The product was purified further via flash chromatography if needed.

10.3 General procedure C: Reduction of *N*-arylated-4-nitropyrazoles

The corresponding 4-nitropyrazol (1 eq.) was combined with an excess of Fe powder (5-10 eq.) and NH_4Cl (5-10 eq.). The reaction was suspended in an EtOH:H_2O mixture (2-4:1) and heated to 60-70 °C for 45 min – 3 h or until full consumption of the nitro compound. After the reaction cooled down, it was filtered over celite, which was rinsed heavily with MeOH or EtOH. The alcoholic component was then removed and the mixture was taken up with EtOAc und washed with 0.5-2 M NaOH (aq.), or saturated $NaHCO_3$ (aq.) solution. After the watery phase was reextracted with EtOAc, the combined organic layers were dried over Na_2SO_4, filtered and evaporated to dryness.

10.4 General procedure D: Urea-formation with isocyanates

The corresponding amine (1 eq.) was placed inside a flask and dissolved in dry DCM, Toluol or THF. Subsequently the isocyanate (1 eq.) was added via syringe or pipet. The mixture was stirred overnight at RT or until full conversion. The reaction was either filtered directly to obtain the pure product or quenched with MeOH. After the solvent was evaporated the product was purified using flash chromatography (MeOH/DCM).

10.5 General procedure E: Boc deprotection

The purified Boc protected amine (1 eq.) was placed inside a flask and dissolved in EtOH. An excess of 1.25 M HCl (EtOH) (5-10 eq.) was added to the solution, which was heated to 50-60 °C and stirred overnight. After evaporation of the solvent the pure, solid product was isolated and further dried in high vacuum.

10.6 Scaffold synthesis Table 1:

Scheme S1: Synthesis Table 1

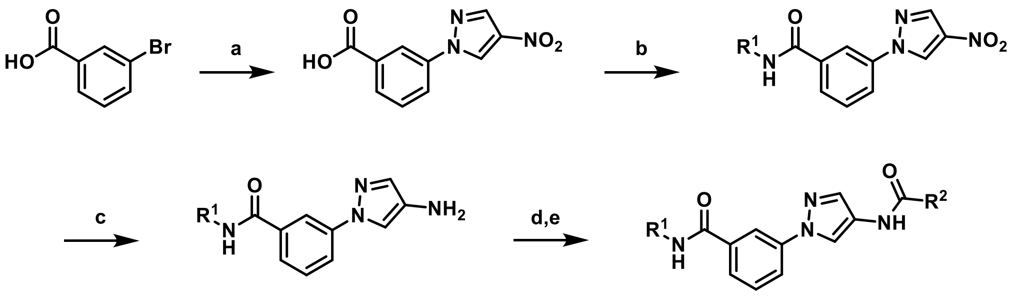

Reaction conditions and reagents exemplified by the synthesis of **1f**: **a)** 4-nitro-1*H*-pyrazole (1 eq.), 3-bromobenzoic acid (1.5 eq.), Cs_2CO_3 (3 eq.), Cu(I)I (0.2 eq.), *trans-N,N´*-dimethylcyclohexane-1,2-diamine (0.4 eq.), DMF (dry), 95 °C (87%); **b) 2** (1 eq.), **4** (1.02 eq.), EDC HCl (2 eq.), HOBt monohydrate + 20 wt.% water (2 eq.) , DIPEA (3 eq.), DCM, RT (82%); **c) 6** (1 eq.), Fe(0) (10 eq.), NH_4Cl (10 eq.), EtOH:H_2O (2:1), 65 °C (89%); **d) 7** (1 eq.), 1-isocyanatonaphthalene (1 eq), DCM, RT (crude); **e)** product of d) (1 eq.), 1.25 M HCl (EtOH) (7,5 eq.), EtOH, 60 °C (64%). (Synthesis after K. Zheng et. al with minor adjustments)[6]

3-(4-nitro-1*H*-pyrazol-1-yl)benzoic acid (**2**)

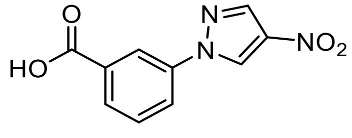

The reaction followed general procedure A: Ullmann-type reaction with pyrazoles.

5000 mg 4-nitro-1*H*-pyrazole (44.2 mmol; 1 eq.), 13333 mg 3-brombenzoic acid (66.3 mmol; 1.5 eq.) and 43220 mg Cs_2CO_3 (132.6 mmol; 3 eq.) were suspended in 200 mL of dry DMF. After degasification of the mixture and after putting the system under argon, 2.79 mL of ligand (0.4 eq.) and 1684 mg of Cu(I)I (0.2 eq.) were introduced. The reaction was sealed and heated to 95 °C for 20 h. Monitoring via HPLC and TLC (EtOAc/Hexane (50/50) + 0.5% AcOH) showed full consumption of 4-nitro-1*H*-pyrazole. The reaction was quenched with 10% HCl (aq.). The precipitated product was filtered of and washed with 10% HCl (aq.) and water to yield (**2**) as a white solid (9020 mg; 87%). ^1H NMR (400 MHz, DMSO) δ 13.36 (br s, 1H), 9.87 – 9.67 (m, 1H), 8.57 (s, 1H), 8.51 – 8.42 (m, 1H), 8.20 (ddd, J = 8.2, 2.4, 1.0 Hz, 1H), 8.03 – 7.92 (m, 1H), 7.69 (t, J = 8.0 Hz, 1H); ^{13}C NMR (101 MHz, DMSO) δ 166.3, 138.5, 137.1, 137.0, 132.4, 130.1, 128.8, 128.5, 123.5, 120.1; TLC-MS(ESI) *m/z*: 232.0 $[M-H]^-$.

tert-butyl 4-bromopiperidine-1-carboxylate (**3**)

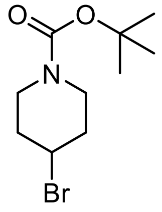

2000 mg of 4-Bromopiperidine hydrobromide (8.2 mmol, 1 eq.) was suspended in 20 mL of dry DCM. Dropwise 2.78 mL of DIPEA (16.3 mmol; 2 eq.) was added while the mixture was cooled via ice bath. After the exothermic reaction had subsided, 1782 mg of di-*tert*-butyl dicarbonate

(8.2 mmol, 1 eq.) was added and the cooling was removed. The reaction was stirred at RT overnight. The solution was quenched with demin. Water and 1 M HCl (aq.) and extracted 2x with DCM. The organic phase was further washed 1x with saturated NH_4Cl (aq.) solution and 1x with brine. After the organic phase was dried over Na_2SO_4, filtered and evaporated the crude product could be purified via flash chromatography (EtOAc/PE: 10/90 isocratic) to yield (**3**) as a yellow oil (2009 mg; 93%). ^1H NMR (400 MHz, CDCl3) δ 4.37 – 4.29 (m, 1H), 3.72 – 3.62 (m, 2H), 3.34 – 3.26 (m, 2H), 2.13 – 2.03 (m, 2H), 1.97 – 1.86 (m, 2H), 1.45 (s, 9H); ^{13}C NMR (101 MHz, CDCl3) δ 154.7, 79.9, 49.6, 42.2, 35.7, 28.5.

tert-butyl 4-(4-nitro-1*H*-pyrazol-1-yl)piperidine-1-carboxylate (**4**)

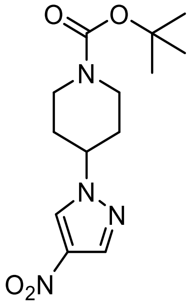

3500 mg of 4-nitro-1*H*-pyrazole (31 mmol; 1 eq.) and 12833 mg of K_2CO_3 (92.9 mmol; 3 eq.) were suspended in 200 mL of dry ACN. After 30 min of stirring 12346 mg of (**3**) (46.7 mmol; 1.51 eq.) was added dissolved in 2x 50 mL of dry ACN. The reaction was heated to reflux for 48h after which the conversion came to a hold. The mixture was diluted with 200 mL of demin. water and extracted 4x with EtOAc. The organic phase was dried over Na_2SO_4, filtered and evaporated and then purified via flash chromatography (EtOAc/PE: 20/80 – 50/50). The still crude product was then washed 3x with 2 M NaOH (aq.) solution to remove remaining 4-nitro-1*H*-pyrazole. After drying and evaporating of the organic phase, one could obtain (**4**) as a yellowish oil which crystalized in the deep freezer (-22 °C) as a white solid (4308 mg; 47%). ^1H NMR (400 MHz, CDCl3) δ 8.16 (s, 1H), 8.07 (s, 1H), 4.34 – 4.21 (m, 3H), 2.89 (t, J = 12.8 Hz, 2H), 2.16 (d, J = 12.4 Hz, 2H), 1.90 (qd, J = 12.2, 3.9 Hz, 2H), 1.47 (s, 9H); ^{13}C NMR (101 MHz, CDCl3) δ 154.6, 135.9, 135.7, 126.4, 80.4, 60.8, 42.6, 32.1, 28.5; TLC-MS(ESI) *m/z*: 196.8 $[M\text{-}Boc+H]^+$; 230.7 $[M\text{-}Boc+Cl]^-$.

tert-butyl 4-(4-amino-1*H*-pyrazol-1-yl)piperidine-1-carboxylate (**5**)

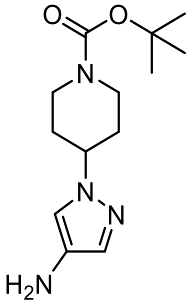

3000 mg of (**4**) (10.1 mmol, 1 eq.) was dissolved in 90 mL of EtOAc within a miniclave® glas reactor (Büchi). The reactor was then alternately evacuated and flushed with argon for 3 consecutive times. In the argon countercurrent 150 mg of (10%) Pd/C (0.142 mmol (Pd); 0.014 eq) was added to the solution. The reactor was loaded with 7 bar of H_2 and the reaction stirred at 40 °C for 72h. After total conversion the mixture was quickly filtered over cotton and purified via flash chromatography (MeOH/DCM: 4/96 – 10/90) to yield (**5**) as a raspberry red oil which crystalizes over time into a bright red solid (2648 mg; 98%). ^1H NMR (400 MHz, DMSO) δ 7.06 (d, J = 0.8 Hz, 1H), 6.90 (d, J = 0.8 Hz, 1H), 4.12 (tt, J = 11.4, 4.0 Hz, 1H), 3.99 (d, J = 12.1

Hz, 2H), 3.79 (s, 2H), 2.85 (br s, 2H), 1.95 – 1.84 (m, 2H), 1.67 (qd, J = 12.4, 4.4 Hz, 2H), 1.41 (s, 9H); ^{13}C NMR (101 MHz, DMSO) δ 153.9, 130.6, 128.9, 114.5, 78.8, 57.7, 42.4(signal suppressed), 31.9, 28.1; TLC-MS(ESI) *m/z*: 289.6 [M+Na]$^+$.

tert-butyl 4-(4-(3-(4-nitro-1*H*-pyrazol-1-yl)benzamido)-1*H*-pyrazol-1-yl)piperidine-1-carboxylate (**6**)

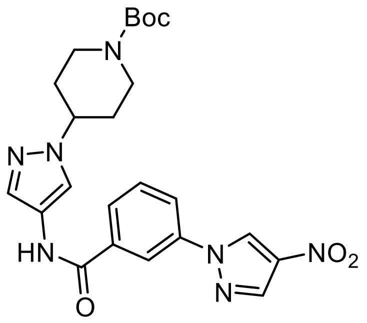

The reaction followed general procedure B1: Amide coupling using EDC HCl + HOBt.

2250 mg of (**2**) (9.6 mmol; 1 eq.) as well as 3700 mg of EDC HCl (19.3 mmol 2 eq.) and 3546 mg of HOBt monohydrate (19.3 mmol; 2 eq.) (containing 20 wt.% water) were put into a flask and suspended in 40 mL of dry DCM, while the mixture was cooled via ice bath. 5 mL of DIPEA (28.9 mmol; 3 eq.) were added and the mixture was allowed to stir for 20 min. Afterwards 2621 mg of amine (**5**) (9.8 mmol; 1.02 eq.) were added and the cooling was removed. The reaction stirred overnight. The mixture was quenched with 100 mL of demin. water and then extracted 3 consecutive times with EtOAc. The combined organic layers were dried over Na_2SO_4, filtered and evaporated to dryness. The crude product was then purified using flash chromatography (MeOH/DCM: 2/98 – 10/90) to yield (**6**) as red-orange flakes (3809 mg; 82%). ^{1}H NMR (400 MHz, DMSO) δ 10.60 (s, 1H), 9.71 (s, 1H), 8.62 (s, 1H), 8.49 (t, J = 1.8 Hz, 1H), 8.15 (dd, J = 8.1, 1.5 Hz, 1H), 8.11 (s, 1H), 8.00 (d, J = 8.0 Hz, 1H), 7.72 (t, J = 8.0 Hz, 1H), 7.65 (s, 1H), 4.37 (tt, J = 11.3, 3.9 Hz, 1H), 4.04 (d, J = 12.4 Hz, 2H), 2.90 (s, 2H), 2.05 – 1.95 (m, 2H), 1.78 (qd, J = 12.4, 4.3 Hz, 2H), 1.42 (s, 9H); ^{13}C NMR (101 MHz, DMSO) δ 162.3, 153.8, 138.4, 137.0, 136.9, 135.7, 130.3, 129.9, 128.3, 127.1, 122.2, 121.2, 119.2, 118.5, 78.7, 58.0, 42.3 (signal suppressed), 31.8, 28.0; TLC-MS(ESI) *m/z*: 504.6 [M+Na]$^+$; 480.6 [M-H]$^-$; 516.6 [M+Cl]$^-$.

tert-butyl 4-(4-(3-(4-amino-1*H*-pyrazol-1-yl)benzamido)-1*H*-pyrazol-1-yl)piperidine-1-carboxylate (**7**)

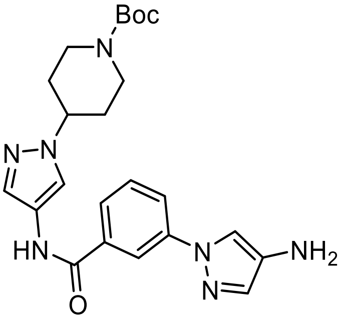

The reaction followed general procedure C: Reduction of *N*-arylated-4-nitropyrazoles.

500 mg of (**6**) (1.04 mmol; 1 eq.) were combined with 580 mg of Fe powder (10.4 mmol; 10 eq.) and 555 mg of NH_4Cl (10.4 mmol; 10 eq.). The solids were then suspended in 45 mL of a mixture of EtOH:H_2O (2:1). The reaction was heated to 65-70 °C for 45 min after which monitoring via HPLC and TLC showed full conversion. After cooling to RT the reaction was

filtered through celite. The alcoholic components were evaporated and the remaining suspension was diluted with EtOAc and subsequently washed 2x with 2M NaOH (aq.). The aquatic layer was 1x re-extracted with EtOAc. The combined organic layers were dried over Na_2SO_4, filtered and evaporated to dryness to yield (**7**) as a red solid (418 mg; 89%). ^{1}H NMR (400 MHz, DMSO) δ 10.54 (s, 1H), 8.22 (s, 1H), 8.10 (s, 1H), 7.85 (dd, J = 8.1, 1.4 Hz, 1H), 7.77 (s, 1H), 7.74 (d, J = 7.9 Hz, 1H), 7.64 (s, 1H), 7.56 (t, J = 7.9 Hz, 1H), 7.32 (s, 1H), 4.36 (tt, J = 11.3, 3.8 Hz, 1H), 4.27 (br s, 2H), 4.04 (d, J = 11.9 Hz, 2H), 2.90 (br s, 2H), 1.99 (d, J = 10.2 Hz, 2H), 1.78 (qd, J = 12.4, 4.3 Hz, 2H), 1.42 (s, 9H); ^{13}C NMR (101 MHz, DMSO) δ 163.0, 153.9, 140.1, 135.4, 133.9, 133.1, 130.3, 129.6, 123.6, 121.4, 119.7, 119.2, 115.8, 112.1, 78.8, 58.0, 42.4(signal suppressed), 31.9, 28.1; TLC-MS(ESI) *m/z*: 474.7 $[M+Na]^+$; 450.7 $[M-H]^-$; 486.7 $[M+Cl]^-$.

10.7 Compound synthesis Table 1:

3-(4-(3-(2-chlorophenyl)ureido)-1*H*-pyrazol-1-yl)-*N*-(1-(piperidin-4-yl)-1*H*-pyrazol-4-yl)benzamide (**A-1**) (**HCl salt**)

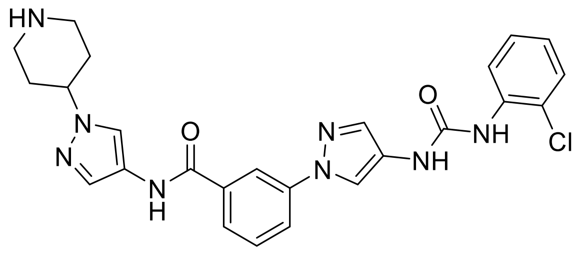

The reaction followed general procedure D(step 1)+E(step 2): Urea-formation with isocyanates + Boc deprotection.

Step 1: 101 mg (**7**) (0.223 mmol; 1 eq.) were dissolved in 12 mL of dry DCM. At RT 27 µL of 2-chlorophenyl isocyanate (0.223 mmol; 1 eq.) were then added via pipet. After 22 h the conversion was not finished, therefore 10 µL of isocyanate were further added. After 24 h the reaction was completed. Subsequently the solvent was evaporated and the product was purified via flash chromatography (MeOH/DCM: 3/97 – 10/90). The intermediate product was then directly used in step 2.

Step 2: The product of step 1 was dissolved in 10 mL of EtOH. 0.9 mL of 1.25 M HCl in EtOH (1.118 mmol; 5 eq.) were added to the mixture and the reaction was heated to 50 °C for 22 h. After reaction monitoring via HPLC showed total conversion, the solvent was evaporated. The product was suspended in Et_2O, filtered off and dried further until completely dry to yield (**A-1**) as a yellow solid (96 mg; 79%; **HCl salt**). ^{1}H NMR (400 MHz, DMSO) δ 10.78 (s, 1H), 9.77 (s, 1H), 9.23 (d, J = 9.1 Hz, 1H), 9.07 – 8.93 (m, 1H), 8.60 (s, 1H), 8.52 (s, 1H), 8.40 (s, 1H), 8.19 (dd, J = 8.3, 1.3 Hz, 1H), 8.13 (s, 1H), 8.01 (dd, J = 8.1, 1.3 Hz, 1H), 7.87 (d, J = 7.8 Hz, 1H), 7.84 (s, 1H), 7.72 (s, 1H), 7.62 (t, J = 7.9 Hz, 1H), 7.45 (dd, J = 8.0, 1.3 Hz, 1H), 7.33 – 7.25 (m, 1H), 7.02 (td, J = 8.0, 1.4 Hz, 1H), 4.56 – 4.47 (m, 1H), 3.41 – 3.34 (m, 2H), 3.11 – 2.99 (m, 2H), 2.22 – 2.13 (m, 4H); ^{13}C NMR (101 MHz, DMSO) δ 162.8, 152.1, 139.8, 136.1, 135.3, 133.1, 130.6, 129.8, 129.2, 127.5, 124.8, 124.5, 123.2, 122.0, 121.7, 121.2, 120.6, 119.3, 116.5, 116.5, 55.1, 42.1, 28.6; FTIR [cm^{-1}]: 3216, 3088, 2706, 2372, 1691, 1653, 1577, 1526, 1437, 1388; TLC-MS(ESI) *m/z*: 505.4 $[M+H]^+$; 503.5 $[M-H]^-$; 539.5 $[M+Cl]^-$; HRMS(ESI) *m/z*: calcd. for $[M+H]^+$: 505.18610; found: 505.1862; HPLC t_{ret}: 5.34 min.

3-(4-(3-(2-fluorophenyl)ureido)-1*H*-pyrazol-1-yl)-*N*-(1-(piperidin-4-yl)-1*H*-pyrazol-4-yl)benzamide (**1a**)

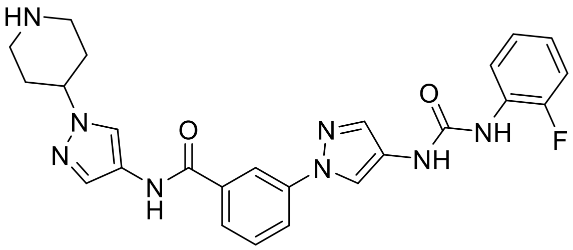

The reaction followed general procedure D(step 1): Urea formation with isocyanates.

Step 1: 55 mg (**7**) (0.122 mmol; 1 eq.) were dissolved in 7 mL of dry DCM. At RT 14 µL of 2-fluorophenyl isocyanate (0.122 mmol; 1 eq.) were then added via pipet. After 4 h the intermediate product in solution was then directly used in step 2.

Step 2: To the solution of step 1 1 mL of TFA was added. The reaction was stirred for 30 min and then evaporated to dryness. Saturated $NaHCO_3$ (aq.) solution was added and the suspension was stirred overnight. The reaction was extracted with EtOAc, dried over Na_2SO_4, filtered and evaporated. The crude product was purified via flash chromatography (MeOH/DCM_3: 5/95 – 15/85 + 2 N NH_3). The product was dried until completely dry to yield (**1a**) as a red solid (20 mg; 37%). ^{1}H NMR (400 MHz, DMSO) δ 10.64 (d, J = 13.4 Hz, 1H), 9.10 (d, J = 6.8 Hz, 1H), 8.69 (d, J = 2.2 Hz, 1H), 8.59 (s, 1H), 8.35 (s, 1H), 8.16 (td, J = 8.3, 1.4 Hz, 1H), 8.11 (s, 1H), 8.01 (dd, J = 8.1, 1.3 Hz, 1H), 7.84 (t, J = 3.8 Hz, 2H), 7.70 – 7.59 (m, 2H), 7.27 – 7.21 (m, 1H), 7.14 (t, J = 7.7 Hz, 1H), 7.05 – 6.98 (m, 1H), 4.38 (dt, J = 14.8, 5.4 Hz, 1H), 3.27 (d, J = 12.7 Hz, 2H), 2.88 (dt, J = 12.2, 6.2 Hz, 2H), 2.15 – 2.05 (m, 2H), 2.03 – 1.94 (m, 2H); FTIR [cm^{-1}]: 3264, 3071, 2950, 2850, 1669, 1584, 1540, 1490, 1454, 1396; TLC-MS(ESI) *m/z*: 489.4 [M+H]$^+$; 511.5 [M+Na]$^+$; 487.4 [M-H]$^-$; 523.4 [M+Cl]$^-$; HRMS(ESI) *m/z*: calcd. for [M+H]$^+$: 489.21565; found: 489.2160; HPLC t_{ret}: 4.80 min.

N-(1-(piperidin-4-yl)-1*H*-pyrazol-4-yl)-3-(4-(3-(*o*-tolyl)ureido)-1*H*-pyrazol-1-yl)benzamide (**1b**) (**HCl salt**)

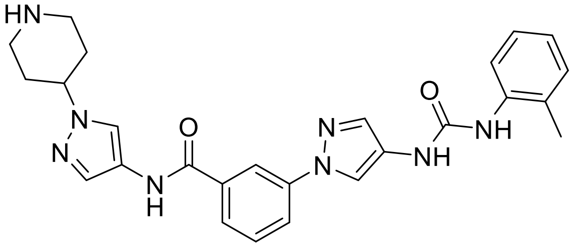

The reaction followed general procedure D(step 1)+E(step 2): Urea-formation with isocyanates + Boc deprotection.

Step 1: 50 mg (**7**) (0.111 mmol; 1 eq.) were dissolved in 5 mL of dry DCM. At RT 16 µL of 1-isocyanato-2-methylbenzene (0.111 mmol; 1 eq.) were then added via pipet. After 24 h the conversion was finished. Subsequently the solvent was evaporated and the product was purified via flash chromatography (MeOH/DCM: 2/98 – 10/90). The intermediate product was then directly used in step 2.

Step 2: The product of step 1 was dissolved in 10 mL of EtOH. 0.44 mL of 1.25 M HCl in EtOH (0.554 mmol; 5 eq.) were added to the mixture and the reaction was heated to 60 °C for 24 h. After reaction monitoring via HPLC showed total conversion, the solvent was evaporated. The product was dried further until completely dry to yield (**1b**) as a light brown solid (26 mg; 44%; **HCl salt**). ^{1}H NMR (400 MHz, DMSO) δ 10.75 (s), 9.49 (s), 9.17 (d, J = 9.8 Hz), 8.93 (q, J = 9.6 Hz), 8.57 (s), 8.35 (d, J = 20.2 Hz), 8.13 (s), 8.00 (d, J = 7.7 Hz), 7.86 (t, J = 8.2 Hz), 7.80 (s), 7.71 (s), 7.61 (t, J = 7.5 Hz), 7.20 – 7.09 (m), 6.93 (t, J = 7.0 Hz), 4.60 – 4.43 (m), 3.38 (d,

J = 9.4 Hz), 3.05 (s), 2.27 (s), 2.17 (s); ^{13}C NMR (101 MHz, DMSO) δ 162.8, 152.6, 139.8, 137.6, 135.3, 133.0, 130.5, 130.1, 129.8, 127.4, 126.1, 124.9, 124.7, 122.4, 121.6, 120.7, 120.5, 119.3, 116.3, 116.1, 55.1, 42.2, 28.6, 18.1; FTIR [cm^{-1}]: 3396, 3228, 3101, 1692, 1605, 1577, 1534, 1488, 1471, 1454; TLC-MS(ESI) *m/z*: 485.7 [M+H]$^+$; 483.8 [M-H]$^-$; 519.7 [M+Cl]$^-$; HRMS(ESI) *m/z*: calcd. for [M+H]$^+$: 485.24072; found: 485.2401; HPLC t_{ret}: 4.76 min.

3-(4-(3-cyclopentylureido)-1*H*-pyrazol-1-yl)-*N*-(1-(piperidin-4-yl)-1*H*-pyrazol-4-yl)benzamide (**1c**) (**HCl salt**)

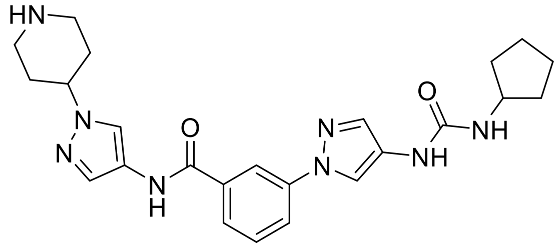

The reaction followed general procedure D(step 1)+E(step 2): Urea-formation with isocyanates + Boc deprotection.

Step 1: 50 mg (**7**) (0.111 mmol; 1 eq.) were dissolved in 5 mL of dry DCM. At RT 25 µL of isocyanatocyclopentane (0.111 mmol; 1 eq.) were then added via pipet. After 24 h the solvent was evaporated and the product was purified via flash chromatography (MeOH/DCM: 2/98 – 10/90). The intermediate product was then directly used in step 2.

Step 2: The product of step 1 was dissolved in 10 mL of EtOH. 0.28 mL of 4 M HCl in Dioxan (1.107 mmol; 10 eq.) were added to the mixture and the reaction was stirred at RT for 24 h. After reaction monitoring via HPLC showed total conversion, the solvent was evaporated. The product was dried further until completely dry to yield (**1c**) as a brown solid (43 mg; 84%; **HCl salt**). ^{1}H NMR (400 MHz, MeOD) δ 8.32 (s, 1H), 8.26 (s, 1H), 8.20 (s, 1H), 7.90 (d, J = 8.0 Hz, 1H), 7.83 (d, J = 7.5 Hz, 1H), 7.73 (s, 1H), 7.67 (s, 1H), 7.60 (t, J = 7.7 Hz, 1H), 4.56 (s, 1H), 4.05 (q, 1H), 3.57 (d, J = 11.9 Hz, 2H), 3.23 (t, J = 11.3 Hz, 2H), 2.39 – 2.21 (m, 4H), 2.02 – 1.91 (m, 2H), 1.80 – 1.56 (m, 4H), 1.53 – 1.41 (m, 2H); ^{13}C NMR (101 MHz, MeOD) δ 166.3, 141.8, 136.8, 134.7, 132.5, 131.0, 126.4, 125.9, 123.0, 122.6, 121.7, 118.4, 57.0, 53.1, 44.2, 34.2, 30.2, 24.5; FTIR [cm^{-1}]: 2944, 2867, 2719, 2499, 1642, 1583, 1559, 1491, 1388, 1281; TLC-MS(ESI) *m/z*: 463.7 [M+H]$^+$; 497.8 [M+Cl]$^-$; HRMS(ESI) *m/z*: calcd. for [M+H]$^+$: 463.25637; found: 463.2555; HPLC t_{ret}: 4.29 min.

3-(4-(3-cyclopropylureido)-1*H*-pyrazol-1-yl)-*N*-(1-(piperidin-4-yl)-1*H*-pyrazol-4-yl)benzamide (**1d**) (**TFA salt**)

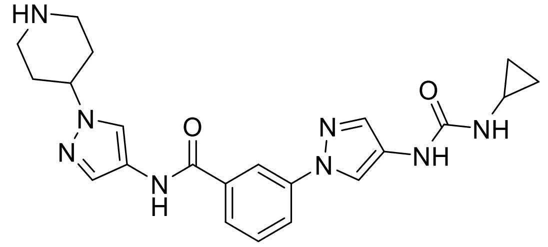

The reaction followed general procedure D(step 1): Urea-formation with isocyanates.

Step 1: 50 mg (**7**) (0.111 mmol; 1 eq.) were dissolved in 5 mL of dry DCM. At RT 8 µL of isocyanatocyclopropane (0.111 mmol; 1 eq.) were then added via pipet. After 24 h the intermediate product in solution was then directly used in step 2.

Step 2: To the solution of step 1 1 mL of TFA was added. The reaction was stirred for 30 min and then evaporated to dryness. The reaction was extracted with EtOAc, dried over Na_2SO_4, filtered and evaporated. The crude product was purified via flash chromatography (MeOH/DCM_3: 5/95 – 15/85 + 2 N NH_3). The product was dried until completely dry to yield (**1d**) as an orange solid (21 mg; 35%; **TFA salt**). ^{1}H NMR (400 MHz, DMSO) δ 10.68 (s, 1H), 8.72 (br s, 2H), 8.46 (d, J = 7.9 Hz, 2H), 8.30 (s, 1H), 8.13 (s, 1H), 7.95 (dd, J = 8.1, 1.3 Hz, 1H), 7.82 (d, J = 7.8 Hz, 1H), 7.74 (s, 1H), 7.68 (s, 1H), 7.61 (t, J = 7.9 Hz, 1H), 6.63 (d, J = 2.2 Hz, 1H), 4.57 – 4.47 (m, 1H), 3.45 – 3.38 (m, 2H), 3.07 (td, J = 12.3, 2.7 Hz, 2H), 2.58 – 2.51 (m, 1H), 2.23 – 2.06 (m, 4H), 0.73 – 0.57 (m, 2H), 0.49 – 0.33 (m, 2H); ^{13}C NMR (101 MHz, DMSO) δ 162.9, 158.6 – 157.5 (m), 155.9, 139.9, 135.3, 133.2, 130.6, 129.8, 125.6, 124.5, 121.6, 120.4, 119.5, 117.5 (dd, J = 611.6, 311.4 Hz), 116.3, 116.1, 55.1, 42.4, 28.8, 22.5, 6.6; FTIR [cm^{-1}]: 3295, 3071, 3001, 2731, 2498, 1643, 1583, 1551, 1340, 1200; TLC-MS(ESI) *m/z*: 435.6 $[M+H]^+$; 433.7 $[M-H]^-$; HRMS(ESI) *m/z*: calcd. for $[M+H]^+$: 435.22507; found: 435.2251; HPLC t_{ret}: 2.61 min.

3-(4-(3-phenylureido)-1*H*-pyrazol-1-yl)-*N*-(1-(piperidin-4-yl)-1*H*-pyrazol-4-yl)benzamide (**1e**) (**HCl salt**)

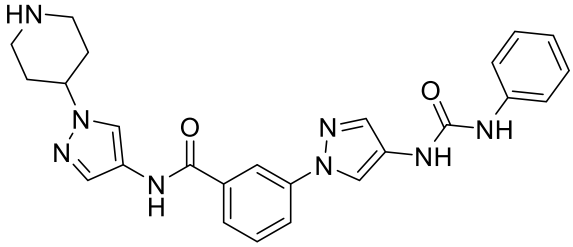

The reaction followed general procedure D(step 1)+E(step 2): Urea-formation with isocyanates + Boc deprotection.

Step 1: 74 mg (**7**) (0.164 mmol; 1 eq.) were dissolved in 10 mL of dry Toluol. At RT 18 µL of isocyanatobenzene (0.164 mmol; 1 eq.) were then added via pipet. After 24 h the conversion was finished. Subsequently the solvent was evaporated and the product was purified via flash chromatography (MeOH/DCM: 5/95 – 10/90). The intermediate product was then directly used in step 2.

Step 2: The product of step 1 was dissolved in 10 mL of EtOH. 1.3 mL of 1.25 M HCl in EtOH (1.639 mmol; 10 eq.) were added to the mixture and the reaction was heated to 60 °C for 24 h. After reaction monitoring via HPLC showed total conversion, the solvent was evaporated. The product was suspended in Et_2O, filtered off and dried further until completely dry to yield (**1e**) as a yellow solid (20 mg; 26%; **HCl salt**). ^{1}H NMR (400 MHz, DMSO) δ 10.73 (s, 1H), 9.25 (s, 1H), 9.19 (d, J = 8.0 Hz, 1H), 9.06 (s, 1H), 8.95 (d, J = 8.4 Hz, 1H), 8.57 (s, 1H), 8.37 (s, 1H), 8.13 (s, 1H), 8.00 (d, J = 7.6 Hz, 1H), 7.85 (d, J = 7.4 Hz, 1H), 7.80 (s, 1H), 7.71 (s, 1H), 7.61 (t, J = 7.8 Hz, 1H), 7.48 (d, J = 7.9 Hz, 2H), 7.27 (t, J = 7.6 Hz, 2H), 6.95 (t, J = 7.2 Hz, 1H), 4.57 – 4.45 (m, 1H), 3.38 (d, J = 11.4 Hz, 2H), 3.05 (s, 2H), 2.18 (s, 4H); ^{13}C NMR (101 MHz, DMSO) δ 162.8, 152.5, 139.9, 139.8, 135.3, 133.1, 130.5, 129.7, 128.7, 124.8, 124.7, 121.6, 121.6, 120.8, 120.5, 119.3, 117.9, 116.3, 55.1, 42.1, 28.6; FTIR [cm^{-1}]: 3270, 3043, 2796, 2712, 2501, 1595, 1553, 1490, 1442, 1388; TLC-MS(ESI) *m/z*: 471.9 $[M+H]^+$; 505.8 $[M+Cl]^-$; HRMS(ESI) *m/z*: calcd. for $[M+H]^+$: 471.22507; found: 471.2241; HPLC t_{ret}: 4.35 min.

3-(4-(3-(naphthalen-1-yl)ureido)-1*H*-pyrazol-1-yl)-*N*-(1-(piperidin-4-yl)-1*H*-pyrazol-4-yl)benzamide (**1f)** (**HCl salt**)

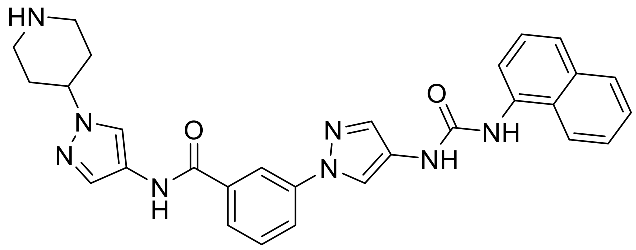

The reaction followed general procedure D(step 1)+E(step 2): Urea-formation with isocyanates + Boc deprotection.

Step 1: 77 mg (**7**) (0.171 mmol; 1 eq.) were dissolved in 10 mL of dry DCM. At RT 25 µL of 1-isocyanatonaphthalene (0.171 mmol; 1 eq.) were then added via pipet. After 24 h the solvent was evaporated and the product was purified via flash chromatography (MeOH/DCM: 5/95 – 10/90). The intermediate product was then directly used in step 2.

Step 2: The product of step 1 was dissolved in 12 mL of EtOH. 1 mL of 1.25 M HCl in EtOH (1.279 mmol; 7.5 eq.) was added to the mixture and the reaction was heated to 60 °C for 24 h. After reaction monitoring via HPLC showed total conversion, the solvent was evaporated. The product was suspended in Et_2O, filtered off and dried further until completely dry to yield (**1f**) as a light brown solid (61 mg; 64%; **HCl salt**). ^{1}H NMR (400 MHz, DMSO) δ 10.77 (s, 1H), 9.83 (s, 1H), 9.38 (s, 1H), 9.22 (d, J = 9.6 Hz, 1H), 9.07 – 8.91 (m, 1H), 8.62 (s, 1H), 8.39 (d, J = 9.2 Hz, 2H), 8.14 (s, 1H), 8.12 – 8.08 (m, 1H), 8.02 (dd, J = 8.1, 1.3 Hz, 1H), 7.94 – 7.89 (m, 1H), 7.89 – 7.83 (m, 2H), 7.72 (s, 1H), 7.62 (t, J = 8.3 Hz, 2H), 7.58 – 7.50 (m, 2H), 7.46 (t, J = 7.9 Hz, 1H), 4.59 – 4.45 (m, 1H), 3.41 – 3.34 (m, 2H), 3.10 – 2.99 (m, 2H), 2.25 – 2.10 (m, 4H); ^{13}C NMR (101 MHz, DMSO) δ 162.8, 153.0, 139.9, 135.3, 134.8, 133.8, 133.0, 130.6, 129.8, 128.3, 125.9, 125.7, 125.6, 125.0, 124.8, 122.6, 121.9, 121.7, 120.6, 119.4, 116.6, 116.3, 116.2, 55.2, 42.2, 28.7; FTIR [cm^{-1}]: 3234, 3086, 2751, 2548, 1690, 1653, 1547, 1488, 1386, 1340; TLC-MS(ESI) *m/z*: 521.1 [M+H]$^+$; 555.5 [M+Cl]$^-$; HRMS(ESI) *m/z*: calcd. for [M+H]$^+$: 521.24072; found: 521.2405; HPLC t_{ret}: 5.55 min.

3-(4-(3-(2,3-dimethylphenyl)ureido)-1*H*-pyrazol-1-yl)-*N*-(1-(piperidin-4-yl)-1*H*-pyrazol-4-yl)benzamide (**1g**) (**HCl salt**)

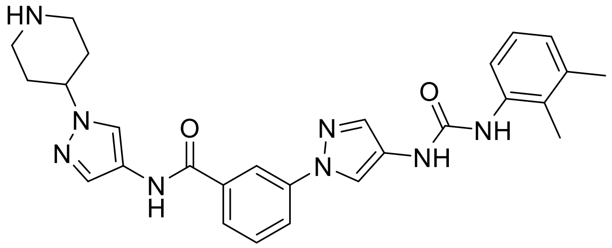

The reaction followed general procedure D(step 1)+E(step 2): Urea-formation with isocyanates + Boc deprotection.

Step 1: 75 mg (**7**) (0.166 mmol; 1 eq.) were dissolved in 6 mL of dry DCM. At RT 23 µL of 1-isocyanato-2,3-dimethylbenzene (0.166 mmol; 1 eq.) were then added via pipet. After 24 h the solvent was evaporated and the product was purified via flash chromatography (MeOH/DCM: 5/95 – 10/90). The intermediate product was then directly used in step 2.

Step 2: The product of step 1 was dissolved in 12 mL of EtOH. 1.33 mL of 1.25 M HCl in EtOH (1.661 mmol; 10 eq.) was added to the mixture and the reaction was heated to 60 °C for 24 h. After reaction monitoring via HPLC showed total conversion, the solvent was evaporated. The product was suspended in Et_2O, filtered off and dried further until completely dry to yield (**1g**) as a light brown-yellow solid (64 mg; 72%; **HCl salt**). ^{1}H NMR (400 MHz, DMSO) δ 10.74 (s,

1H), 9.33 (s, 1H), 9.16 (d, J = 9.4 Hz, 1H), 8.92 (dd, J = 18.9, 9.4 Hz, 1H), 8.55 (s, 1H), 8.36 (s, 1H), 8.32 (s, 1H), 8.13 (s, 1H), 7.99 (dd, J = 8.1, 1.0 Hz, 1H), 7.85 (d, J = 7.7 Hz, 1H), 7.79 (s, 1H), 7.71 (s, 1H), 7.64 – 7.54 (m, 2H), 7.02 (t, J = 7.8 Hz, 1H), 6.88 (d, J = 7.4 Hz, 1H), 4.55 – 4.45 (m, 1H), 3.38 (d, J = 12.4 Hz, 2H), 3.05 (s, 2H), 2.25 (s, 3H), 2.21 – 2.13 (m, 7H); ^{13}C NMR (101 MHz, DMSO) δ 162.8, 152.9, 139.9, 137.2, 136.5, 135.3, 133.0, 130.6, 129.8, 127.3, 125.2, 125.1, 124.7, 124.7, 121.6, 120.5, 120.1, 119.3, 116.3, 116.1, 55.1, 42.2, 28.7, 20.4, 13.7; FTIR [cm^{-1}]: 3243, 2938, 2800, 2712, 2492, 1653, 1584, 1540, 1395, 1278; TLC-MS(ESI) *m/z*: 500.1 [M+H]$^+$; 534.1 [M+Cl]$^-$; HRMS(ESI) *m/z*: calcd. for [M+H]$^+$: 499.25637; found: 499.2554; HPLC t_{ret}: 5.16 min.

N-(1-(piperidin-4-yl)-1*H*-pyrazol-4-yl)-3-(4-(3-(2-(trifluoromethyl)phenyl)ureido)-1*H*-pyrazol-1-yl)benzamide (**1h**) (**HCl salt**)

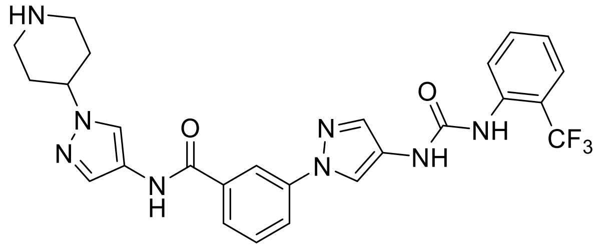

The reaction followed general procedure D(step 1)+E(step 2): Urea-formation with isocyanates + Boc deprotection.

Step 1: 75 mg (**7**) (0.166 mmol; 1 eq.) were dissolved in 6 mL of dry DCM. At RT 25 µL of 1-isocyanato-2-(trifluoromethyl)benzene (0.166 mmol; 1 eq.) were then added via pipet. After 24 h the solvent was evaporated and the product was purified via flash chromatography (MeOH/DCM: 5/95 – 10/90). The intermediate product was then directly used in step 2.

Step 2: The product of step 1 was dissolved in 10 mL of EtOH. 1.33 mL of 1.25 M HCl in EtOH (1.661 mmol; 10 eq.) was added to the mixture and the reaction was heated to 60 °C for 24 h. After reaction monitoring via HPLC showed total conversion, the solvent was evaporated. The product was suspended in Et_2O, filtered off and dried further until completely dry to yield (**1h**) as a cream-colored solid (81 mg; 85%; **HCl salt**). ^{1}H NMR (400 MHz, DMSO) δ 10.76 (s, 1H), 9.63 (s, 1H), 9.20 (d, J = 9.9 Hz, 1H), 8.97 (dd, J = 19.6, 9.9 Hz, 1H), 8.59 (s, 1H), 8.39 (s, 1H), 8.28 (s, 1H), 8.13 (s, 1H), 8.01 – 7.95 (m, 2H), 7.86 (d, J = 7.9 Hz, 1H), 7.83 (s, 1H), 7.71 (s, 1H), 7.68 (d, J = 7.9 Hz, 1H), 7.63 (q, J = 7.6 Hz, 2H), 7.28 (t, J = 7.6 Hz, 1H), 4.51 (dt, J = 15.0, 7.6 Hz, 1H), 3.38 (d, J = 12.6 Hz, 2H), 3.10 – 2.99 (m, 2H), 2.21 – 2.13 (m, 4H); ^{13}C NMR (101 MHz, DMSO) δ 162.8, 152.4, 139.8, 136.5 (q, J = 1.8 Hz), 135.3, 133.1, 132.8, 130.6, 129.7, 128.1 – 119.8 (m), 125.9 (q, J = 5.3 Hz), 125.5, 124.8, 124.5, 123.6, 121.6, 120.6, 119.9 (q, J = 29.0 Hz) 119.3, 116.6, 116.5, 55.1, 42.1, 28.6; FTIR [cm^{-1}]: 2932, 2792, 2710, 1662, 1585, 1539, 1490, 1452, 1395, 1318; TLC-MS(ESI) *m/z*: 539.4 [M+H]$^+$; 537.3 [M-H]$^-$; 573.3 [M+Cl]$^-$; HRMS(ESI) *m/z*: calcd. for [M+H]$^+$: 539.21246; found: 539.2126; HPLC t_{ret}: 6.92 min.

N-(1-(piperidin-4-yl)-1*H*-pyrazol-4-yl)-3-(4-(3-(3-(trifluoromethyl)phenyl)ureido)-1*H*-pyrazol-1-yl)benzamide (**1i**) (**HCl salt**)

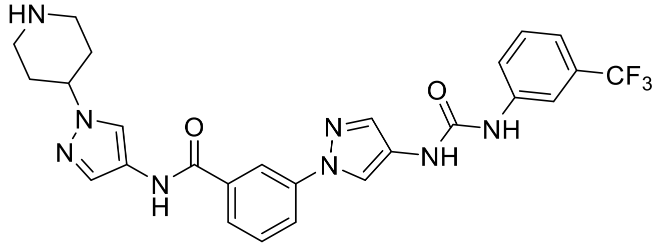

The reaction followed general procedure D(step 1)+E(step 2): Urea-formation with isocyanates + Boc deprotection.

Step 1: 75 mg (**7**) (0.166 mmol; 1 eq.) were dissolved in 6 mL of dry DCM. At RT 23 µL of 1-isocyanato-3-(trifluoromethyl)benzene (0.166 mmol; 1 eq.) were then added via pipet. After 24 h the solvent was evaporated and the product was purified via flash chromatography (MeOH/DCM: 5/95 – 10/90). The intermediate product was then directly used in step 2.

Step 2: The product of step 1 was dissolved in 10 mL of EtOH. 1.33 mL of 1.25 M HCl in EtOH (1.661 mmol; 10 eq.) was added to the mixture and the reaction was heated to 60 °C for 24 h. After reaction monitoring via HPLC showed total conversion, the solvent was evaporated. The product was suspended in Et_2O, filtered off and dried further until completely dry to yield (**1i**) as a light brown solid (75 mg; 78%; **HCl salt**). ^{1}H NMR (400 MHz, DMSO) δ 10.72 (s, 1H), 9.76 (s, 1H), 9.22 (s, 1H), 9.14 (d, J = 9.5 Hz, 1H), 8.90 (dd, J = 18.8, 9.3 Hz, 1H), 8.61 (s, 1H), 8.38 (s, 1H), 8.13 (s, 1H), 8.08 (s, 1H), 8.02 (dd, J = 8.1, 1.4 Hz, 1H), 7.86 (d, J = 7.8 Hz, 1H), 7.82 (s, 1H), 7.71 (s, 1H), 7.65 – 7.57 (m, 1H), 7.50 (t, J = 7.9 Hz, 1H), 7.29 (d, J = 7.6 Hz, 1H), 4.58 – 4.45 (m, 1H), 3.39 (d, J = 12.6 Hz, 1H), 3.13 – 2.98 (m, 1H), 2.23 – 2.11 (m, J = 13.7, 6.9 Hz, 2H); ^{13}C NMR (101 MHz, DMSO) δ 162.8, 152.4, 140.8, 139.8, 135.3, 133.2, 130.6, 129.9, 129.8, 129.5 (q, J = 31.2 Hz), 124.8, 124.4, 124.2 (q, J = 272.3 Hz), 121.6, 121.4, 120.6, 119.4, 117.8 (dd, J = 7.7, 3.7 Hz),116.7, 116.3, 113.7 (q, J = 3.9 Hz), 55.1, 42.2, 28.7; FTIR [cm^{-1}]: 3261, 3092, 2797, 2717, 2496, 1653, 1599, 1559, 1490, 1445; LC-MS(APCI) *m/z*: 538.7 [M+H]$^+$; HRMS(ESI) *m/z*: calcd. for [M+H]$^+$: 539.21246; found: 539.2129; HPLC t_{ret}: 6.29 min.

N-(1-(piperidin-4-yl)-1*H*-pyrazol-4-yl)-3-(4-(3-(4-(trifluoromethyl)phenyl)ureido)-1*H*-pyrazol-1-yl)benzamide (**1j**) (**HCl salt**)

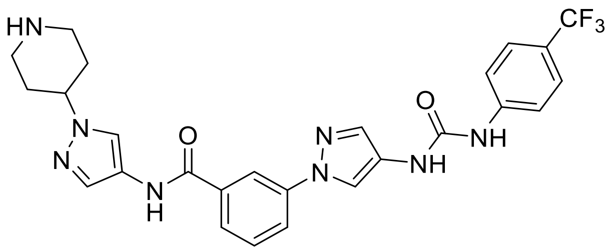

The reaction followed general procedure D(step 1)+E(step 2): Urea-formation with isocyanates + Boc deprotection.

Step 1: 75 mg (**7**) (0.166 mmol; 1 eq.) were dissolved in 6 mL of dry DCM. At RT 24 µL of 1-isocyanato-4-(trifluoromethyl)benzene (0.166 mmol; 1 eq.) were then added via pipet. After 24 h the solvent was evaporated and the product was purified via flash chromatography (MeOH/DCM: 5/95 – 10/90). The intermediate product was then directly used in step 2.

Step 2: The product of step 1 was dissolved in 10 mL of EtOH. 1.33 mL of 1.25 M HCl in EtOH (1.661 mmol; 10 eq.) was added to the mixture and the reaction was heated to 60 °C for 24 h. After reaction monitoring via HPLC showed total conversion, the solvent was evaporated. The product was suspended in Et_2O, filtered off and dried further until completely dry to yield (**1j**) as a light yellow solid (27 mg; 28%; **HCl salt**). ^{1}H NMR (400 MHz, DMSO) δ 10.73 (s, 1H), 9.70 (s, 1H), 9.17 (s, 1H), 9.02 (d, J = 9.0 Hz, 1H), 8.79 (d, J = 9.2 Hz, 1H), 8.60 (s, 1H), 8.37 (s, 1H), 8.14 (s, 1H), 8.02 (d, J = 7.8 Hz, 1H), 7.85 (d, J = 11.6 Hz, 2H), 7.72 – 7.67 (m, 3H), 7.66 – 7.60 (m, 3H), 4.56 – 4.47 (m, 1H), 3.40 (d, J = 12.1 Hz, 2H), 3.12 – 2.98 (m, 2H), 2.23 – 2.08 (m, 4H); ^{13}C NMR (101 MHz, DMSO) δ 162.8, 152.2, 143.7, 139.8, 135.3, 133.3, 130.6, 129.8, 126.1 (dd, J = 7.4, 3.6 Hz), 124.9, 124.6 (dd, J = 541.7, 270.8 Hz), 124.4, 121.6, 121.6 (q, J = 31.8 Hz).120.6, 119.4, 117.6, 116.7, 116.4, 55.1, 42.3, 28.7; FTIR [cm^{-1}]: 3242, 3205, 3092, 2843, 2740, 2549, 1706, 1647, 1609, 1583; TLC-MS(ESI) *m/z*: 539.2 [M+H]$^+$; 537.5 [M-

H]$^-$; 573.6 [M+Cl]$^-$; HRMS(ESI) *m/z*: calcd. for [M+H]$^+$: 539.21246; found: 539.2117; HPLC t_{ret}: 6.23 min.

10.8 Scaffold synthesis Table 2:

Scheme S2: Synthesis Table 2

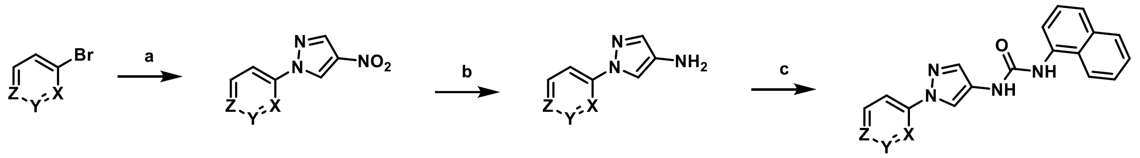

Reaction conditions and reagents exemplified by the synthesis of **16a**: **a)** 4-nitro-1*H*-pyrazole (1 eq.), bromobenzene (1.2 eq.), K_2CO_3 (3 eq.), *trans*-*N*,*N'*-dimethylcyclohexane-1,2-diamine (0.2 eq.), Cu(I)I (0.1 eq.), DMF (dry), 95 °C (100%); **b) 8** (1 eq.), Fe(0) (5 eq.), NH_4Cl (5 eq.), EtOH:H_2O (4:1), 60 °C (quant.); **c) 12** (1 eq.), 1-isocyanatonaphthalene (1 eq.), THF, RT (37%).

4-nitro-1-phenyl-1*H*-pyrazole (**8**)

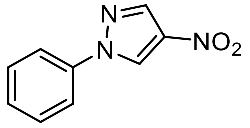

The reaction followed general procedure A: Ullmann-type reaction with pyrazoles.

4000 mg 4-nitro-1*H*-pyrazole (35.4 mmol; 1 eq.), 6665 mg bromobenzene (42.4 mmol; 1.2 eq.) and 14666 mg K_2CO_3 (106.1 mmol; 3 eq.) were suspended in 50 mL of dry DMF. After degasification of the mixture and after putting the system under argon, 1.12 mL of ligand (7.07 mmol; 0.2 eq.) and 674 mg of Cu(I)I (3.54 mmol; 0.1 eq.) were introduced. The reaction was sealed and heated to 95 °C for 20 h. The reaction was quenched with 200 mL of demin. H_2O. The precipitated, pure product was filtered of and washed thoroughly with water and dried in the convection oven to yield (**8**) as a green-yellow powder (6671 mg; 100%). ^1H NMR (400 MHz, DMSO) δ 9.64 (s, 1H), 8.55 (s, 1H), 7.95 (d, J = 7.8 Hz, 2H), 7.57 (t, J = 7.5 Hz, 2H), 7.45 (t, J = 7.1 Hz, 1H); ^{13}C NMR (101 MHz, DMSO) δ 138.4, 136.9, 136.8, 129.7, 128.3, 128.1, 119.4.

2-(4-nitro-1*H*-pyrazol-1-yl)pyridine (**9**)

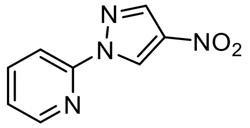

The reaction followed general procedure A: Ullmann-type reaction with pyrazoles.

2000 mg 4-nitro-1*H*-pyrazole (17.69 mmol; 1 eq.), 2794 mg 2-bromopyridine (17.69 mmol; 1 eq.) and 7333 mg K_2CO_3 (53.06 mmol; 3 eq.) were suspended in 50 mL of dry DMF. After degasification of the mixture and after putting the system under argon, 0.558 mL of ligand (3.54 mmol; 0.2 eq.) and 337 mg of Cu(I)I (1.77 mmol; 0.1 eq.) were introduced. The reaction was sealed and heated to 95 °C for 18 h. The reaction was quenched with demin. H_2O. The precipitated, pure product was filtered of and washed thoroughly with water and Et_2O and dried in the convection oven to yield (**9**) as a gray-brown solid (2899 mg; 86%). ^1H NMR (400 MHz, DMSO) δ 9.43 (s, 1H), 8.61 (s, 1H), 8.57 (d, J = 3.9 Hz, 1H), 8.10 (t, J = 7.7 Hz, 1H), 8.00 (d, J = 8.1 Hz, 1H), 7.57 – 7.48 (m, 1H). ^{13}C NMR (101 MHz, DMSO) δ 149.4, 148.8, 140.2, 137.7, 136.9, 126.6, 124.1, 113.0.

3-(4-nitro-1*H*-pyrazol-1-yl)pyridine (**10**)

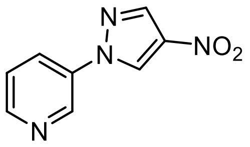

The reaction followed general procedure A: Ullmann-type reaction with pyrazoles.

2000 mg 4-nitro-1*H*-pyrazole (17.69 mmol; 1 eq.), 2794 mg 3-bromopyridine (17.69 mmol; 1 eq.) and 7333 mg K_2CO_3 (53.06 mmol; 3 eq.) were suspended in 50 mL of dry DMF. After degasification of the mixture and after putting the system under argon, 0.558 mL of ligand (3.54 mmol; 0.2 eq.) and 337 mg of Cu(I)I (1.77 mmol; 0.1 eq.) were introduced. The reaction was sealed and heated to 95 °C for 18 h. The reaction was quenched with demin. H_2O. The precipitated, pure product was filtered of and washed thoroughly with water and Et_2O and dried in the convection oven to yield (**10**) as a green-brown solid (1640 mg; 49%). ^{1}H NMR (400 MHz, DMSO) δ 9.74 (s, 1H), 9.20 (s, 1H), 8.67 (s, 1H), 8.61 (s, 1H), 8.35 (ddd, J = 8.4, 2.6, 1.3 Hz, 1H), 7.63 (dd, J = 8.2, 4.7 Hz, 1H); ^{13}C NMR (101 MHz, DMSO) δ 149.2, 140.9, 137.5, 137.1, 135.1, 128.8, 127.3, 124.4.

4-(4-nitro-1*H*-pyrazol-1-yl)pyridine (**11**)

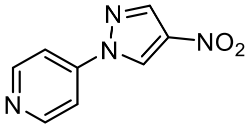

The reaction followed general procedure A: Ullmann-type reaction with pyrazoles.

2000 mg 4-nitro-1*H*-pyrazole (17.69 mmol; 1 eq.), 2923 mg 4-bromopyridine hydrochloride (15.02 mmol; 0.85 eq.) and 9777 mg K_2CO_3 (70.75 mmol; 4 eq.) were suspended in 50 mL of dry DMF. After degasification of the mixture and after putting the system under argon, 0.558 mL of ligand (3.54 mmol; 0.2 eq.) and 337 mg of Cu(I)I (1.77 mmol; 0.1 eq.) were introduced. The reaction was sealed and heated to 95 °C for 18 h. The reaction was quenched with demin. H_2O. The precipitated, pure product was filtered of and washed thoroughly with water and dried in the convection oven to yield (**11**) as a green-brown solid (1940 mg; 68%). ^{1}H NMR (400 MHz, DMSO) δ 9.86 (s, 1H), 8.78 (s, 2H), 8.63 (s, 1H), 8.01 (d, J = 5.4 Hz, 2H); ^{13}C NMR (101 MHz, DMSO) δ 151.4, 144.5, 137.9, 137.5, 128.9, 113.2.

1-phenyl-1*H*-pyrazol-4-amine (**12**)

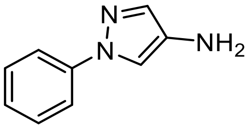

The reaction followed general procedure C: Reduction of *N*-arylated-4-nitropyrazoles.

500 mg of (**8**) (2.64 mmol; 1 eq.) were combined with 738 mg of Fe powder (13.22 mmol; 5 eq.) and 707 mg of NH_4Cl (13.22 mmol; 5 eq.). The solids were then suspended in 30 mL of a mixture of EtOH:H_2O (4:1). The reaction was heated to 60 °C for 2 h. After cooling to RT the reaction was filtered through celite. The alcoholic components were evaporated and the remaining suspension was diluted with EtOAc and subsequently washed 3x with 1 M NaOH (aq.). The aquatic layer was 1x re-extracted with EtOAc. The combined organic layers were dried over Na_2SO_4, filtered and evaporated to dryness to yield (**12**) (Purity: 96.1% at 254 nm; 93.2% at 230 nm) as a dark-red solid (quantitative). ^{1}H NMR (400 MHz, DMSO) δ 7.70 – 7.65 (m, 3H), 7.44 – 7.39 (m, 2H), 7.27 (d, J = 0.6 Hz, 1H), 7.20 – 7.15 (m, 1H), 4.17 (br s, 2H); ^{13}C NMR (101 MHz, DMSO) δ 140.1, 133.5, 132.7, 129.3, 124.7, 117.0, 112.1.

1-(pyridin-2-yl)-1*H*-pyrazol-4-amine (**13**)

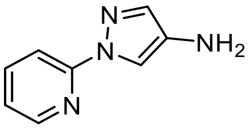

The reaction followed general procedure C: Reduction of *N*-arylated-4-nitropyrazoles.

500 mg of (**9**) (2.63 mmol; 1 eq.) were combined with 734 mg of Fe powder (13.15 mmol; 5 eq.) and 703 mg of NH_4Cl (13.15 mmol; 5 eq.). The solids were then suspended in 30 mL of a mixture of EtOH:H_2O (4:1). The reaction was heated to 60 °C for 2h. After cooling to RT the reaction was filtered through celite. The alcoholic components were evaporated and the remaining suspension was diluted with EtOAc and subsequently washed 2x with 1 M NaOH (aq.). The aquatic layer was 1x re-extracted with EtOAc. The combined organic layers were dried over Na_2SO_4, filtered and evaporated to dryness to yield (**13**) as a dark-red solid (393 mg; 93%). ^{1}H NMR (400 MHz, DMSO) δ 8.37 (ddd, J = 4.9, 1.8, 0.9 Hz, 1H), 7.90 – 7.83 (m, 2H), 7.81 – 7.76 (m, 1H), 7.35 (d, J = 0.7 Hz, 1H), 7.19 (ddd, J = 7.2, 4.9, 1.1 Hz, 1H), 4.30 (br s, 2H); ^{13}C NMR (101 MHz, DMSO) δ 151.2, 148.0, 138.8, 134.4, 133.6, 120.3, 110.9, 110.7.

1-(pyridin-3-yl)-1*H*-pyrazol-4-amine (**14**)

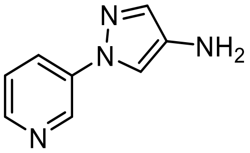

The reaction followed general procedure C: Reduction of *N*-arylated-4-nitropyrazoles.

500 mg of (**10**) (2.63 mmol; 1 eq.) were combined with 734 mg of Fe powder (13.15 mmol; 5 eq.) and 703 mg of NH_4Cl (13.15 mmol; 5 eq.). The solids were then suspended in 30 mL of a mixture of EtOH:H_2O (4:1). The reaction was heated to 60 °C for 2 h. After cooling to RT the reaction was filtered through celite. The alcoholic components were evaporated and the remaining suspension was diluted with EtOAc and subsequently washed 2x with 1 M NaOH (aq.). The aquatic layer was 1x re-extracted with EtOAc. The combined organic layers were dried over Na_2SO_4, filtered and evaporated to dryness to yield (**14**) as a black-purple solid (300 mg; 71%). ^{1}H NMR (400 MHz, DMSO) δ 8.96 (d, J = 2.5 Hz, 1H), 8.39 (dd, J = 4.7, 1.3 Hz, 1H), 8.06 (ddd, J = 8.4, 2.6, 1.4 Hz, 1H), 7.77 (s, 1H), 7.45 (dd, J = 8.3, 4.7 Hz, 1H), 7.34 (s, 1H), 4.26 (br s, 2H); ^{13}C NMR (101 MHz, DMSO) δ 145.7, 138.6, 136.3, 134.0, 133.7, 124.1, 124.0, 112.0.

1-(pyridin-4-yl)-1*H*-pyrazol-4-amine (**15**)

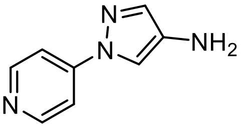

The reaction followed general procedure C: Reduction of *N*-arylated-4-nitropyrazoles.

500 mg of (**11**) (2.63 mmol; 1 eq.) were combined with 734 mg of Fe powder (13.15 mmol; 5 eq.) and 703 mg of NH_4Cl (13.15 mmol; 5 eq.). The solids were then suspended in 30 mL of a mixture of EtOH:H_2O (4:1). The reaction was heated to 60 °C for 2 h. After cooling to RT the reaction was filtered through celite. The alcoholic components were evaporated and the remaining suspension was diluted with EtOAc and subsequently washed 3x with 1 M NaOH (aq.). The aquatic layer was 1x re-extracted with EtOAc. The combined organic layers were

dried over Na_2SO_4, filtered and evaporated to dryness to yield (**15**) as a pinkish-red solid (243 mg; 58%). ^{1}H NMR (400 MHz, DMSO) δ 8.52 (dd, J = 4.8, 1.5 Hz, 2H), 7.81 – 7.78 (m, 1H), 7.65 (dd, J = 4.8, 1.6 Hz, 2H), 7.39 (s, 1H), 4.37 (br s, 2H); ^{13}C NMR (101 MHz, DMSO) δ 150.8, 145.6, 135.1, 134.6, 111.2, 110.9.

10.9 Compound synthesis Table 2:

1-(naphthalen-1-yl)-3-(1-phenyl-1*H*-pyrazol-4-yl)urea (**16a**)

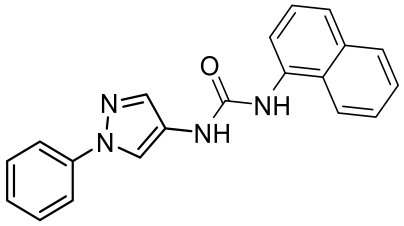

The reaction followed general procedure D: Urea-formation with isocyanates.

100 mg of (**12**) (0.63 mmol; 1 eq.) were dissolved in 15 mL of dry THF. While stirring, 90 µL of 1-isocyanatonaphthalene (0.63 mmol; 1 eq.) were added to the solution. The reaction was stirred at RT for 16h. The product precipitated, was filtered and washed with THF and Pentane to yield the pure compound (**16a**) as a white solid (77 mg; 37%). ^{1}H NMR (400 MHz, DMSO) δ 8.95 (s, 1H), 8.84 (s, 1H), 8.48 (s, 1H), 8.12 (d, J = 8.4 Hz, 1H), 8.04 (d, J = 7.5 Hz, 1H), 7.93 (d, J = 7.6 Hz, 1H), 7.85 (s, 1H), 7.83 – 7.79 (m, 2H), 7.64 (d, J = 8.2 Hz, 1H), 7.62 – 7.57 (m, 1H), 7.57 – 7.52 (m, 1H), 7.51 – 7.45 (m, 3H), 7.28 (t, J = 7.4 Hz, 1H).; ^{13}C NMR (101 MHz, DMSO) δ 152.8, 139.8, 134.5, 133.7, 132.9, 129.5, 128.4, 126.0, 125.9, 125.7, 125.7, 124.5, 122.9, 121.4, 117.8, 117.3, 116.3; FTIR [cm^{-1}]: 3267, 3097, 3050, 1638, 1584, 1501, 1415, 1382, 1344, 1247; TLC-MS(ESI) *m/z*: 351.2 [M+Na]$^+$; 327.0 [M-H]$^-$; 363.1 [M+Cl]$^-$; HRMS(ESI) *m/z*: calcd. for [M+H]$^+$: 329.13961; found: 329.1410; HPLC t_{ret}: 8.03 min.

1-(naphthalen-1-yl)-3-(1-(pyridin-2-yl)-1*H*-pyrazol-4-yl)urea (**16b**)

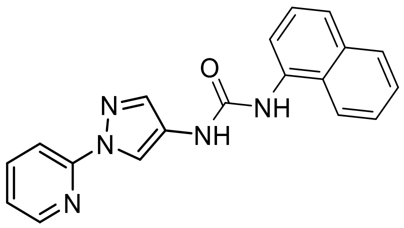

The reaction followed general procedure D: Urea-formation with isocyanates.

100 mg of (**15**) (0.62 mmol; 1 eq.) were dissolved in 10 mL of dry THF. While stirring, 90 µL of 1-isocyanatonaphthalene (0.62 mmol; 1 eq.) were added to the solution. The reaction was stirred at RT for 72 h. The product precipitated, was filtered and washed with THF and Pentane to yield the pure compound (**16b**) as an off-white solid (128 mg; 62%). ^{1}H NMR (400 MHz, DMSO) δ 9.02 (s, 1H), 8.87 (s, 1H), 8.72 (s, 1H), 8.46 (dd, J = 4.8, 0.7 Hz, 1H), 8.12 (d, J = 8.3 Hz, 1H), 8.03 – 7.89 (m, 5H), 7.65 (d, J = 8.2 Hz, 1H), 7.62 – 7.46 (m, 3H), 7.31 (ddd, J = 6.8, 4.8, 0.9 Hz, 1H); 13C NMR (101 MHz, DMSO) δ 152.8, 151.0, 148.3, 139.3, 134.4, 134.1, 133.7, 128.4, 126.2, 125.9, 125.9, 125.7, 124.7, 123.1, 121.5, 121.4, 117.7, 115.1, 111.3; FTIR [cm^{-1}]: 3273, 3098, 3046, 1638, 1593, 1570, 1471, 1456, 1395, 1369; TLC-MS(ESI) *m/z*: 352.2 [M+Na]$^+$; 384.2 [M+Na+MeOH]$^+$; 328.1 [M-H]$^-$; 364.1 [M+Cl]$^-$; HRMS(ESI) *m/z*: calcd. for [M+H]$^+$: 330.13486; found: 330.1346; HPLC t_{ret}: 7.48 min.

1-(naphthalen-1-yl)-3-(1-(pyridin-3-yl)-1*H*-pyrazol-4-yl)urea (**16c**)

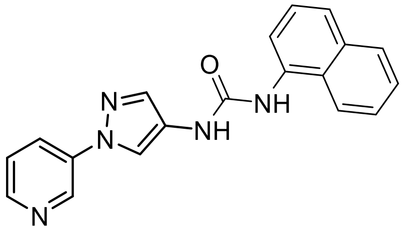

The reaction followed general procedure D: Urea-formation with isocyanates.

100 mg of (**14**) (0.62 mmol; 1 eq.) were dissolved in 10 mL of dry THF. While stirring, 90 µL of 1-isocyanatonaphthalene (0.62 mmol; 1 eq.) were added to the solution. The reaction was stirred at RT for 72 h. The product precipitated, was filtered and washed with THF and Pentane to yield the pure compound (**16c**) as an off-white solid (116 mg; 56%). ^{1}H NMR (400 MHz, DMSO) δ 9.09 (s, 1H), 9.00 (s, 1H), 8.85 (s, 1H), 8.58 (s, 1H), 8.49 (d, J = 3.4 Hz, 1H), 8.22 (d, J = 7.7 Hz, 1H), 8.12 (d, J = 8.2 Hz, 1H), 8.03 (d, J = 7.4 Hz, 1H), 7.98 – 7.87 (m, 2H), 7.67 – 7.46 (m, 5H); ^{13}C NMR (101 MHz, DMSO) δ 152.8, 146.7, 139.3, 136.1, 134.4, 133.9, 133.7, 128.4, 126.0, 125.9, 125.9, 125.7, 125.1, 125.0, 124.2, 123.0, 121.4, 117.4, 116.5; FTIR [cm^{-1}]: 3263, 3109, 3040, 1638, 1579, 1554, 1481, 1384, 1345, 1249; TLC-MS(ESI) *m/z*: 352.1 [M+Na]$^{+}$; 384.2 [M+Na+MeOH]$^{+}$; 328.1 [M-H]$^{-}$; 364.1 [M+Cl]$^{-}$; HRMS(ESI) *m/z*: calcd. for [M+H]$^{+}$: 330.13486; found: 330.1350; HPLC t_{ret}: 6.62 min.

1-(naphthalen-1-yl)-3-(1-(pyridin-4-yl)-1*H*-pyrazol-4-yl)urea (**16d**)

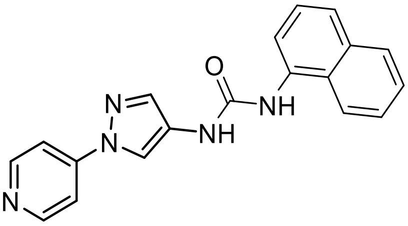

The reaction followed general procedure D: Urea-formation with isocyanates.

100 mg of (**13**) (0.62 mmol; 1 eq.) were dissolved in 10 mL of dry THF. While stirring, 90 µL of 1-isocyanatonaphthalene (0.62 mmol; 1 eq.) were added to the solution. The reaction was stirred at RT for 72h. The product precipitated, was filtered and washed with THF and Pentane to yield the pure compound (**16d**) as a white-pinkish solid (112 mg; 55%). ^{1}H NMR (400 MHz, DMSO) δ 9.05 (s, 1H), 8.89 (s, 1H), 8.65 (s, 1H), 8.63 – 8.57 (m, 2H), 8.11 (d, J = 8.3 Hz, 1H), 8.02 (d, J = 7.5 Hz, 1H), 7.98 (s, 1H), 7.94 (d, J = 7.8 Hz, 1H), 7.86 – 7.80 (m, 2H), 7.65 (d, J = 8.1 Hz, 1H), 7.62 – 7.53 (m, 2H), 7.48 (t, J = 7.9 Hz, 1H); ^{13}C NMR (101 MHz, DMSO) δ 152.8, 151.1, 145.5, 134.9, 134.3, 133.8, 128.4, 126.1, 126.0, 125.9, 125.8, 125.5, 123.1, 121.4, 117.5, 116.1, 111.6; FTIR [cm^{-1}]: 3271, 3109, 3063, 1641, 1591, 1559, 1502, 1411, 1370, 1345; TLC-MS(ESI) *m/z*: 352.3 [M+Na]$^{+}$; 384.3 [M+Na+MeOH]+; 328.2 [M-H]$^{-}$; 364.2 [M+Cl]$^{-}$; HRMS(ESI) *m/z*: calcd. for [M+H]$^{+}$: 330.13486; found: 330.1356; HPLC t_{ret}: 5.25 min.

10.10 Scaffold synthesis Table 3:

Scheme S3: Synthesis Table 3 and additional compounds

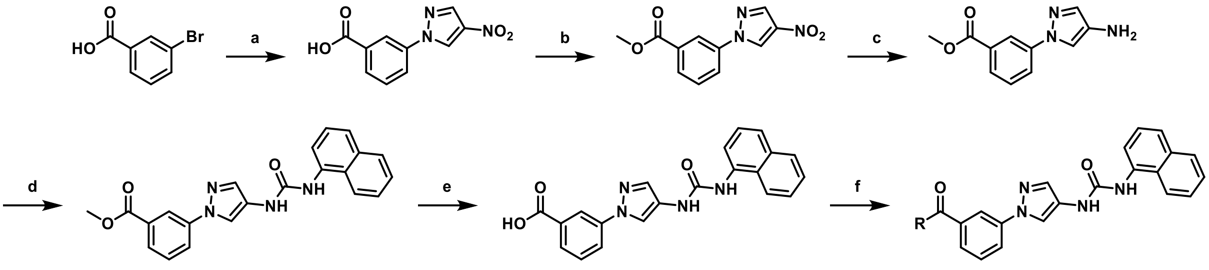

Reaction conditions and reagents exemplified by the synthesis of **21b**: **a)** 4-nitro-1*H*-pyrazole (1 eq.), 3-bromobenzoic acid (1.5 eq.), Cs_2CO_3 (3 eq.), Cu(I)I (0.2 eq.), *trans-N,N´*-dimethylcyclohexane-1,2-diamine (0.4 eq.), DMF (dry), 95 °C (87%); **b) 2** (1 eq.), H_2SO_4 (cat.), MeOH, 65 °C (76%); **c) 17** (1 eq.), Fe(0) (5 eq.), NH_4Cl (5 eq.), EtOH:H_2O (3:1), 60-65 °C (91%); **d) 18** (1 eq.), 1-isocyanatonaphthalene (1 eq.), DCM (dry), RT (81%); **e) 19** (1 eq.), KOH (7.5 eq.), MeOH:H_2O (20:1), 60 °C (86%); **f) 20** (1 eq.), EDC HCl (2 eq.), HOBt monohydrate (containing 14 wt.% water) (2 eq.), DIPEA (4 eq.), (2 M in THF) methylamine (2 eq.), THF, RT (84%).

methyl 3-(4-nitro-1*H*-pyrazol-1-yl)benzoate (**17**)

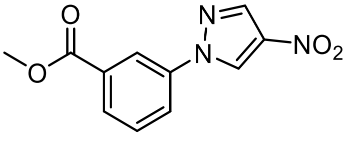

500 mg of (**2**) (2.144 mmol; 1 eq.) were suspended in 25 mL of MeOH. 3 drops of H_2SO_4 were added to the reaction, which was then heated to reflux for 20 h. After cooling the precipitated product was filtered of and washed carefully with a tiny amount of cold MeOH, to yield the pure product (**17**) as a white needles (403 mg; 76%). ^1H NMR (400 MHz, DMSO) δ 9.76 (s, 1H), 8.57 (s, 1H), 8.46 (s, 1H), 8.23 (d, J = 7.8 Hz, 1H), 7.99 (d, J = 7.5 Hz, 1H), 7.71 (t, J = 7.9 Hz, 1H), 3.90 (s, 3H); ^{13}C NMR (101 MHz, DMSO) δ 165.3, 138.6, 137.2, 137.0, 131.2, 130.4, 128.6, 128.6, 123.8, 119.9, 52.5; TLC-MS(ESI) *m/z*: 248.5 $[M+H]^+$.

methyl 3-(4-amino-1*H*-pyrazol-1-yl)benzoate (**18**)

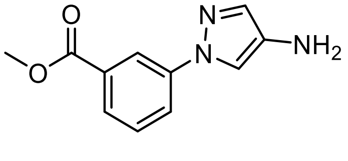

The reaction followed general procedure C: Reduction of *N*-arylated-4-nitropyrazoles.

6000 mg of (**17**) (24.27 mmol; 1 eq.) were combined with 6778 mg of Fe powder (121.35 mmol; 5 eq.) and 6491 mg of NH_4Cl (121.35 mmol; 5 eq.). The solids were then suspended in 500 mL of a mixture of EtOH:H_2O (3:1). The reaction was heated to 60 °C for 1¼ h. After reaction control via TLC the reaction was heated further to 65 °C for 3¾ h. After cooling to RT the reaction was filtered through celite. The alcoholic components were evaporated and the remaining suspension was diluted with EtOAc and subsequently washed 1x with brine and 1x with saturated $NaHCO_3$ (aq.) solution. The aquatic layer was 1x re-extracted with EtOAc. The combined organic layers were dried over Na_2SO_4, filtered and evaporated to dryness to yield (**18**) (Purity: 94.2% at 254 nm; 89.8% at 230 nm) as a brown-red solid (4797 mg; 91%). ^1H NMR (400 MHz, DMSO) δ 8.28 – 8.21 (m, 1H), 7.92 (ddd, J = 8.2, 2.3, 0.9 Hz, 1H), 7.77 (s,

1H), 7.76 – 7.72 (m, J = 7.8 Hz, 1H), 7.56 (t, J = 7.9 Hz, 1H), 7.32 (s, 1H), 4.25 (br s, 2H), 3.88 (s, 3H); ^{13}C NMR (101 MHz, DMSO) δ 165.8, 140.3, 134.0, 133.4, 130.8, 129.9, 125.1, 121.2, 117.3, 112.0, 52.3; TLC-MS(ESI) *m/z*: 218.0 $[M+H]^+$.

methyl 3-(4-(3-(naphthalen-1-yl)ureido)-1*H*-pyrazol-1-yl)benzoate (**19**)

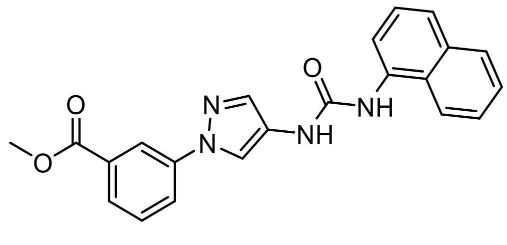

The reaction followed general procedure D: Urea-formation with isocyanates.

5290 mg of (**18**) (24.35 mmol; 1 eq.) were dissolved in 400 mL of dry DCM. While stirring and cooling via ice-water bath, 3.5 mL of 1-isocyanatonaphthalene (24.35 mmol; 1 eq.) were added to the solution in small doses. The reaction was stirred at RT for 20 h. The reaction was quenched with MeOH for 1 h. DCM was evaporated and precipitated product was filtered and washed with *i*-PrOH, Et_2O and Pentane to yield the pure compound (**19**) (Purity: 95.1% at 254 nm; 93.9% at 230 nm) as a pink fluffy solid (8171 mg; 87%). ^{1}H NMR (400 MHz, DMSO) δ 8.98 (s, 1H), 8.88 (s, 1H), 8.58 (s, 1H), 8.39 – 8.31 (m, 1H), 8.16 – 8.06 (m, J = 12.9, 4.9 Hz, 2H), 8.03 (d, J = 7.3 Hz, 1H), 7.94 (d, J = 7.7 Hz, 1H), 7.90 (s, 1H), 7.85 (d, J = 7.8 Hz, 1H), 7.69 – 7.51 (m, 4H), 7.48 (t, J = 7.9 Hz, 1H), 3.90 (s, 3H); ^{13}C NMR (101 MHz, DMSO) δ 165.7, 152.8, 140.0, 134.4, 133.8, 133.5, 131.0, 130.2, 128.4, 126.1, 126.1, 125.9, 125.9, 125.7, 124.9, 123.1, 122.1, 121.4, 118.0, 117.5, 116.4, 52.4; TLC-MS(ESI) *m/z*: 409.2 $[M+Na]^+$; 385.1 $[M-H]^-$; 421.0 $[M+Cl]^-$.

10.11 Compound synthesis Table 3:

3-(4-(3-(naphthalen-1-yl)ureido)-1*H*-pyrazol-1-yl)benzoic acid (**20**)

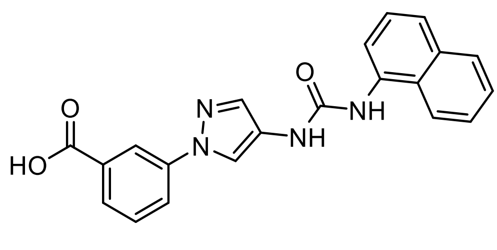

To an appropriate flask 3000 mg of (**19**) (7.76 mmol; 1 eq.) as well as 3267 mg of KOH (58.23 mmol; 7.5 eq.) were suspended in 262.5 mL of a mixture of of MeOH:H_2O (20:1). The reaction was heated to 60 °C. Monitoring via TLC (EtOAc/PE: 75/25 + AcOH) showed full conversion after 20 h. The suspension was vacuum filtered and washed with Et_2O. After drying the pinkish solid was put into a beaker and suspended in 400 mL of demin H_2O. 40 mL of 10% HCl (aq.) solution were added and the mixture was stirred vigorously for 10 min. The product was again vacuum filtered and dried in the oven to yield (**20**) as a white solid (2500 mg; 86%). ^{1}H NMR (400 MHz, DMSO) δ 13.23 (br s, 1H), 8.97 (s, 1H), 8.87 (s, 1H), 8.56 (s, 1H), 8.34 (s, 1H), 8.12 (d, J = 8.3 Hz, 1H), 8.09 – 7.99 (m, 2H), 7.96 – 7.87 (m, 2H), 7.84 (d, J = 7.6 Hz, 1H), 7.68 – 7.51 (m, J = 21.1, 15.2, 7.5 Hz, 4H), 7.48 (t, J = 7.9 Hz, 1H); ^{13}C NMR (101 MHz, DMSO) δ 166.8, 152.9, 139.9, 134.4, 133.8, 133.4, 132.2, 130,0 128.4, 126.4, 126.1, 125.9, 125.9, 125.7, 124.9, 123.0, 121.8, 121.4, 118.3, 117.5, 116.4; FTIR [cm^{-1}]: 3267, 3110, 3068, 3052, 1702, 1685, 1636, 1584, 1555, 1491; TLC-MS(ESI) *m/z*: 371.1 $[M-H]^-$; HRMS(ESI) *m/z*: calcd. for $[M+H]^+$: 373.12944; found: 373.1300; HPLC t_{ret}: 7.31 min.

3-(4-(3-(naphthalen-1-yl)ureido)-1*H*-pyrazol-1-yl)benzamide (**21a**)

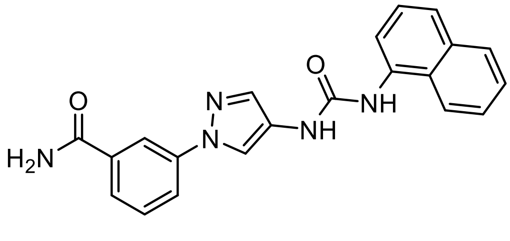

The reaction followed general procedure B2: Amide coupling using CDI.

150 mg of (**20**) (0.403 mmol; 1 eq.) and 130 mg of CDI (0.806 mmo; 2 eq.) were suspended in 20 mL of dry THF. The reaction was stirred at RT for 4 h after which monitoring showed quantitative activation of the carboxylic acid component. Subsequently 71 µL of 25% NH_3 (aq.) solution (0.806 mmol; 2 eq.) were added and the reaction was stirred for 20 h. The product precipitated, was filtered and washed thoroughly with MeOH and Et_2O and was dried under high vacuum to yield (**21a**) as a white solid (33 mg, 22%). ^{1}H NMR (400 MHz, DMSO) δ 8.98 (s, 1H), 8.86 (s, 1H), 8.59 (s, 1H), 8.27 (s, 1H), 8.17 (s, 1H), 8.12 (d, J = 8.3 Hz, 1H), 8.03 (d, J = 7.5 Hz, 1H), 8.00 – 7.91 (m, 2H), 7.86 (s, 1H), 7.77 (d, J = 7.5 Hz, 1H), 7.65 (d, J = 8.1 Hz, 1H), 7.63 – 7.52 (m, 3H), 7.53 – 7.37 (m, 2H); ^{13}C NMR (101 MHz, DMSO) δ 167.1, 152.8, 139.8, 135.6, 134.4, 133.7, 133.1, 129.5, 128.4, 126.0, 125.9, 125.9, 125.7, 124.7, 123.0, 121.4, 120.4, 117.3, 116.5, 116.4; FTIR [cm^{-1}]: 3301, 3167, 3108, 1643, 1627, 1610, 1592, 1583, 1547, 1499; TLC-MS(ESI) *m/z*: 393.8 $[M+Na]^+$; 425.7 $[M+Na+MeOH]^+$; 370.0 $[M-H]^-$; 405.9 $[M+Cl]^-$; HRMS(ESI) *m/z*: calcd. for $[M+H]^+$: 372.14542; found: 372.1452; HPLC t_{ret}: 6.45 min.

N-methyl-3-(4-(3-(naphthalen-1-yl)ureido)-1*H*-pyrazol-1-yl)benzamide (**21b**)

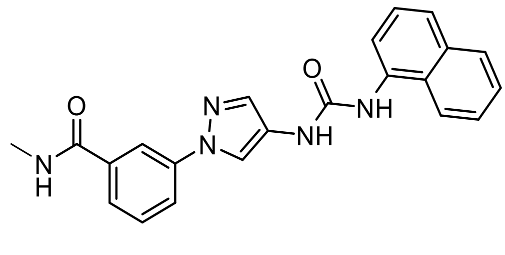

The reaction followed general procedure B1: Amide coupling using EDC HCl + HOBt.

400 mg of (**20**) (1.074 mmol; 1 eq.), 383 mg HOBt monohydrate (containing 14 wt.% water) (2.148 mmol; 2 eq.) and 412 mg EDC HCl (2.148 mmol; 2 eq.) were placed in a flask and suspended in 40 mL of dry THF. 0.748 mL of DIPEA (4.297 mmol; 4 eq.) were added and the reaction stirred for 5 min while it was cooled via ice-water bath. The cooling was removed and the reaction was left to stir for 1 h. 1.074 mL of methylamine (2 M in THF) (2.148 mmol; 2 eq.) were then added. HPLC monitoring showed full conversion after 20 h. The solvent was evaporated and the solids were reconstituted in EtOAc. The organic phase was washed 1x with demin. H_2O, 1x with saturated $NaHCO_3$ (aq.) solution and 1x with saturated NH_4Cl (aq.) solution. The aqueous phase was 1x re-extracted. After drying over Na_2SO_4, filtering and evaporating the organic solvent the crude product was purified via flash chromatography (MeOH/DCM: 2/98 – 10/90) and dried in high vacuum to yield compound (**21b**) as a white solid (346 mg; 84%). ^{1}H NMR (400 MHz, DMSO) δ 8.98 (s, 1H), 8.86 (s, 1H), 8.65 – 8.60 (m, J = 4.5 Hz, 1H), 8.59 (s, 1H), 8.24 (t, J = 1.7 Hz, 1H), 8.13 (d, J = 8.4 Hz, 1H), 8.06 – 8.02 (m, J = 7.7, 0.7 Hz, 1H), 7.98 – 7.91 (m, J = 11.4, 5.7, 0.9 Hz, 2H), 7.86 (s, 1H), 7.74 (d, J = 7.8 Hz, 1H), 7.65 (d, J = 8.2 Hz, 1H), 7.62 – 7.52 (m, 3H), 7.48 (t, J = 7.9 Hz, 1H), 2.83 (d, J = 4.5 Hz, 3H); ^{13}C NMR (101 MHz, DMSO) δ 165.8, 152.8, 139.8, 135.8, 134.4, 133.7, 133.1, 129.6, 128.4, 126.0, 125.9, 125.8, 125.7, 124.7, 124.4, 123.0, 121.4, 120.2, 117.4, 116.4, 116.1, 26.3; FTIR [cm^{-1}]: 3288, 3231, 3081, 2948, 1647, 1584, 1546, 1482, 1394, 1385; TLC-MS(ESI) *m/z*:

408.0 [M+Na]$^+$; 384.1 [M-H]$^-$; 420.0 [M+Cl]$^-$; HRMS(ESI) *m/z*: calcd. for [M+H]$^+$: 386.16107; found: 386.1624; HPLC t_{ret}: 6.71 min.

N,*N*-dimethyl-3-(4-(3-(naphthalen-1-yl)ureido)-1*H*-pyrazol-1-yl)benzamide (**21c**)

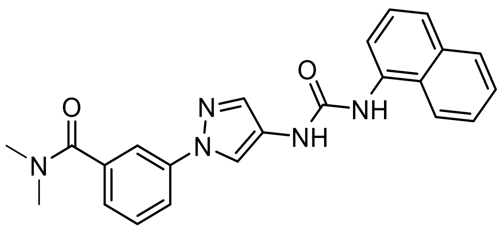

The reaction followed general procedure B1: Amide coupling using EDC HCl + HOBt.

70 mg of (**20**) (0.188 mmol; 1 eq.), 67 mg HOBt monohydrate (containing 14 wt.% water) (0.376 mmol; 2 eq.) and 72 mg EDC HCl (0.376 mmol; 2 eq.) were placed in a flask and suspended in 10 mL of dry DMF. 131 µL of DIPEA (0.752 mmol; 4 eq.) were added and the reaction stirred for 5 min while it was cooled via ice-water bath. 188 µL of dimethylamine (2 M in MeOH) (0.376 mmol; 2 eq.) were added and the cooling was removed shortly after. HPLC monitoring showed full conversion after 18 h. The reaction was quenched with 1 M NaOH (aq.) solution and EtOAc. The mixture was then extracted 2x with EtOAc. The organic phase was washed 1x with 1 M NaOH (aq.) solution, 1x with 1 M HCl (aq.) solution and 1x with brine, after which it was dried over Na_2SO_4, filtered and evaporated. The crude product was purified via flash chromatography (MeOH/DCM: 2/98 – 10/90) and dried in high vacuum to yield compound (**21c**) as a white-yellow solid (23 mg; 31%). ^{1}H NMR (400 MHz, DMSO) δ 8.97 (s, 1H), 8.86 (s, 1H), 8.54 (s, 1H), 8.12 (d, J = 8.2 Hz, 1H), 8.03 (d, J = 7.5 Hz, 1H), 7.93 (d, J = 7.8 Hz, 1H), 7.91 – 7.86 (m, 2H), 7.83 (s, 1H), 7.64 (d, J = 8.1 Hz, 1H), 7.62 – 7.51 (m, 3H), 7.48 (t, J = 7.9 Hz, 1H), 7.28 (d, J = 7.5 Hz, 1H), 3.01 (s, 3H), 2.94 (s, 3H); ^{13}C NMR (101 MHz, DMSO) δ 169.3, 152.8, 139.6, 137.9, 134.4, 133.7, 133.3, 129.6, 128.4, 126.0, 125.9, 125.9, 125.7, 124.7, 123.9, 122.9, 121.4, 118.3, 117.3, 116.4, 116.0, 34.7; FTIR [cm^{-1}]: 3295, 3046, 2924, 1606, 1581, 1549, 1482, 1388, 1341, 1252; TLC-MS(ESI) *m/z*: 422.0 [M+Na]$^+$; 398.1 [M-H]$^-$; 434.1 [M+Cl]$^-$; HRMS(ESI) *m/z*: calcd. for [M+H]$^+$: 400.17672; found: 400.1776; HPLC t_{ret}: 6.96 min.

N-ethyl-3-(4-(3-(naphthalen-1-yl)ureido)-1*H*-pyrazol-1-yl)benzamide (**21d**)

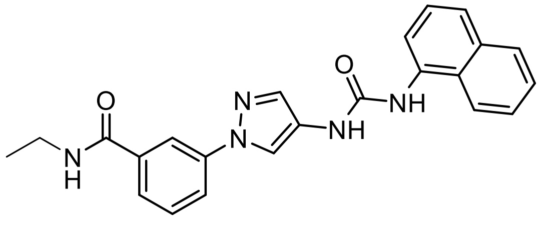

The reaction followed general procedure B1: Amide coupling using EDC HCl + HOBt.

70 mg of (**20**) (0.188 mmol; 1 eq.), 67 mg HOBt monohydrate (containing 14 wt.% water) (0.376 mmol; 2 eq.) and 72 mg EDC HCl (0.376 mmol; 2 eq.) were placed in a flask and suspended in 10 mL of dry DMF. 131 µL of DIPEA (0.752 mmol; 4 eq.) were added and the reaction stirred for 5 min while it was cooled via ice-water bath. 188 µL of ethylamine (2 M in MeOH) (0.376 mmol; 2 eq.) were added and the cooling was removed shortly after. HPLC monitoring showed full conversion after 18 h. The reaction was quenched with demin. H_2O after which the pure product precipitated. The product was filtered and dried, washed with Pentane and further dried under high vacuum to yield compound (**21d**) as a white-yellow solid

(45 mg; 60%). ^{1}H NMR (400 MHz, DMSO) δ 8.99 (s, 1H), 8.87 (s, 1H), 8.67 (s, 1H), 8.59 (s, 1H), 8.23 (s, 1H), 8.12 (d, J = 8.2 Hz, 1H), 8.04 (d, J = 7.4 Hz, 1H), 7.95 (t, J = 8.9 Hz, 2H), 7.86 (s, 1H), 7.75 (d, J = 7.4 Hz, 1H), 7.65 (d, J = 8.0 Hz, 1H), 7.62 – 7.52 (m, 3H), 7.48 (t, J = 7.8 Hz, 1H), 3.40 – 3.26 (m, J = 12.4 Hz, 2H), 1.16 (t, J = 7.0 Hz, 3H); ^{13}C NMR (101 MHz, DMSO) δ 165.2, 152.8, 139.8, 136.0, 134.4, 133.7, 133.1, 129.6, 128.4, 126.0, 125.9, 125.9, 125.7, 124.7, 124.5, 123.0, 121.4, 120.2, 117.4, 116.4, 116.2, 34.1, 14.8; FTIR [cm^{-1}]: 3272, 2971, 2932, 2875, 1639, 1586, 1540, 1490, 1385, 1311; TLC-MS(ESI) *m/z*: 422.0 [M+Na]$^+$; 398.1 [M-H]$^-$; 434.1 [M+Cl]$^-$; HRMS(ESI) *m/z*: calcd. for [M+H]$^+$: 400.17672; found: 400.1776; HPLC t_{ret}: 7.36 min.

N-isopropyl-3-(4-(3-(naphthalen-1-yl)ureido)-1*H*-pyrazol-1-yl)benzamide (**21e**)

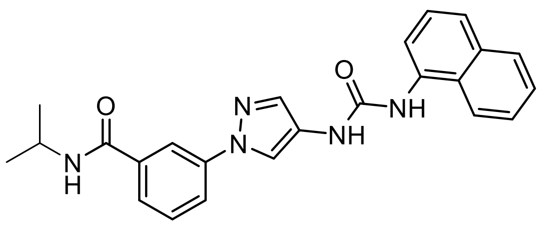

The reaction followed general procedure B1: Amide coupling using EDC HCl + HOBt.

70 mg of (**20**) (0.188 mmol; 1 eq.), 67 mg HOBt monohydrate (containing 14 wt.% water) (0.376 mmol; 2 eq.) and 72 mg EDC HCl (0.376 mmol; 2 eq.) were placed in a flask and suspended in 10 mL of dry DMF. 131 µL of DIPEA (0.752 mmol; 4 eq.) were added and the reaction stirred for 5 min while it was cooled via ice-water bath. 32 µL of isopropyl amine (0.376 mmol; 2 eq.) were added and the cooling was removed shortly after. HPLC monitoring showed full conversion after 18 h. The reaction was quenched with demin. H_2O after which the pure product precipitated. The product was filtered and dried, washed with Pentane and further dried under high vacuum to yield compound (**21e**) as a white solid (52 mg; 67%). ^{1}H NMR (400 MHz, DMSO) δ 9.00 (s, 1H), 8.88 (s, 1H), 8.59 (s, 1H), 8.43 (d, J = 7.6 Hz, 1H), 8.22 (s, 1H), 8.12 (d, J = 8.3 Hz, 1H), 8.03 (d, J = 7.4 Hz, 1H), 7.98 – 7.91 (m, 2H), 7.86 (s, 1H), 7.75 (d, J = 7.7 Hz, 1H), 7.65 (d, J = 8.1 Hz, 1H), 7.62 – 7.52 (m, 3H), 7.48 (t, J = 7.9 Hz, 1H), 4.14 (dh, J = 13.0, 6.4 Hz, 1H), 1.20 (d, J = 6.6 Hz, 6H); ^{13}C NMR (101 MHz, DMSO) δ 164.6, 152.8, 139.7, 136.1, 134.4, 133.7, 133.1, 129.5, 128.4, 126.0, 125.9, 125.9, 125.7, 124.7, 124.7, 123.0, 121.4, 120.2, 117.4, 116.5, 116.3, 41.1, 22.3; FTIR [cm^{-1}]: 3288, 3241, 3110, 3060, 2968, 2934, 2873, 1646, 1633, 1585; TLC-MS(ESI) *m/z*: 436.0 [M+Na]$^+$; 412.2 [M-H]$^-$; 448.1 [M+Cl]$^-$; HRMS(ESI) *m/z*: calcd. for [M+H]$^+$: 414.19238; found: 414.1936; HPLC t_{ret}: 7.82 min.

N-(*tert*-butyl)-3-(4-(3-(naphthalen-1-yl)ureido)-1*H*-pyrazol-1-yl)benzamide (**21f**)

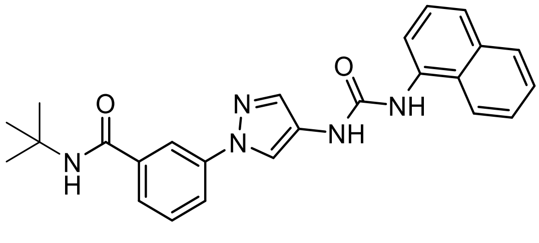

The reaction followed general procedure B1: Amide coupling using EDC HCl + HOBt.

70 mg of (**20**) (0.188 mmol; 1 eq.), 67 mg HOBt monohydrate (containing 14 wt.% water) (0.376 mmol; 2 eq.) and 72 mg EDC HCl (0.376 mmol; 2 eq.) were placed in a flask and suspended in 8 mL of dry DMF. 131 µL of DIPEA (0.752 mmol; 4 eq.) were added and the reaction stirred for 5 min while it was cooled via ice-water bath. 39 µL of isobutyl amine (0.376 mmol; 2 eq.) were added and the cooling was removed shortly after. HPLC monitoring showed

full conversion after 20 h. The reaction was quenched with demin. H_2O after which the pure product precipitated. The product was filtered and dried, washed with Et_2O and further dried under high vacuum to yield compound (**21f**) as a white solid (69 mg; 86%). ^{1}H NMR (400 MHz, DMSO) δ 8.99 (s, 1H), 8.87 (s, 1H), 8.58 (s, 1H), 8.17 – 8.11 (m, 2H), 8.03 (d, J = 7.1 Hz, 1H), 7.99 – 7.90 (m, 3H), 7.86 (s, 1H), 7.73 – 7.68 (m, 1H), 7.65 (d, J = 8.2 Hz, 1H), 7.62 – 7.51 (m, 3H), 7.48 (t, J = 7.9 Hz, 1H), 1.41 (s, 9H); ^{13}C NMR (101 MHz, DMSO) δ 165.6, 152.8, 139.6, 137.2, 134.4, 133.7, 133.1, 129.3, 128.4, 126.0, 125.9, 125.9, 125.7, 124.7, 124.7, 123.0, 121.4, 120.0, 117.4, 116.6, 116.6, 50.9, 28.6; FTIR [cm^{-1}]: 3405, 3258, 2963, 1694, 1633, 1550, 1493, 1444, 1400, 1342; TLC-MS(ESI) *m/z*: 450.1 [M+Na]$^+$; 426.2 [M-H]$^-$; 462.2 [M+Cl]$^-$; HRMS(ESI) *m/z*: calcd. for [M+H]$^+$: 428.20803; found: 428.2104; HPLC t_{ret}: 8.37 min.

3-(4-(3-(naphthalen-1-yl)ureido)-1*H*-pyrazol-1-yl)-*N*-(pentan-3-yl)benzamide (**21g**)

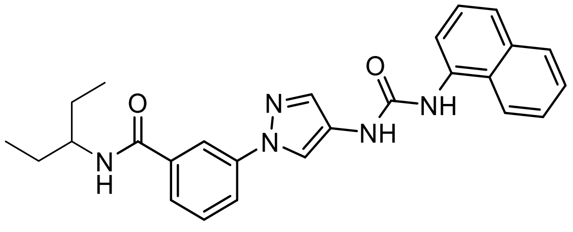

The reaction followed general procedure B1: Amide coupling using EDC HCl + HOBt.

60 mg of (**20**) (0.161 mmol; 1 eq.), 57 mg HOBt monohydrate (containing 14 wt.% water) (0.322 mmol; 2 eq.) and 62 mg EDC HCl (0.322 mmol; 2 eq.) were placed in a flask and suspended in 10 mL of dry DMF. 112 µL of DIPEA (0.645 mmol; 4 eq.) were added and the reaction stirred for 5 min while it was cooled via ice-water bath. 37 µL of pentan-3-amine (0.322 mmol; 2 eq.) were added and the cooling was removed shortly after. HPLC monitoring showed full conversion after 24 h. The reaction was quenched with demin. H_2O after which the pure product precipitated. The product was filtered and dried, washed with Et_2O and Pentane and further dried under high vacuum to yield compound (**21g**) as a white solid (69 mg; 97%). ^{1}H NMR (400 MHz, DMSO) δ 8.99 (s, 1H), 8.87 (s, 1H), 8.59 (s, 1H), 8.32 – 8.18 (m, 2H), 8.12 (d, J = 8.1 Hz, 1H), 8.03 (d, J = 7.3 Hz, 1H), 7.95 (t, J = 7.4 Hz, 2H), 7.87 (s, 1H), 7.76 (d, J = 7.3 Hz, 1H), 7.69 – 7.52 (m, 4H), 7.48 (t, J = 7.7 Hz, 1H), 3.89 – 3.73 (m, 1H), 1.65 – 1.44 (m, 4H), 0.88 (t, J = 7.0 Hz, 6H); ^{13}C NMR (101 MHz, DMSO) δ 165.5, 152.8, 139.7, 136.3, 134.4, 133.7, 133.1, 129.5, 128.4, 126.0, 125.9, 125.9, 125.7, 124.7, 123.0, 121.4, 120.2, 117.4, 116.5, 116.3, 52.2, 27.00, 10.7; FTIR [cm^{-1}]: 3329, 3276, 3229, 3073, 2960, 2930, 2873, 1644, 1629, 1551M; TLC-MS(ESI) *m/z*: 442.9 [M+H]$^+$; 463.8 [M+Na]$^+$; 439.9 [M-H]$^-$; 475.8 [M+Cl]$^-$; HRMS(ESI) *m/z*: calcd. for [M+H]$^+$: 442.22368; found: 442.2252; HPLC t_{ret}: 8.64 min.

N-cyclopropyl-3-(4-(3-(naphthalen-1-yl)ureido)-1*H*-pyrazol-1-yl)benzamide (**21h**)

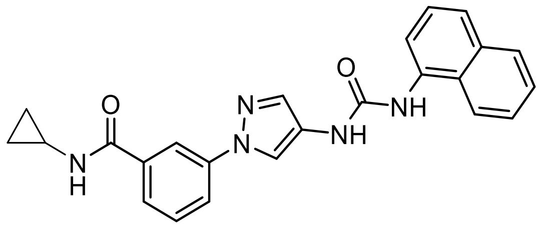

The reaction followed general procedure B1: Amide coupling using EDC HCl + HOBt.

60 mg of (**20**) (0.161 mmol; 1 eq.), 57 mg HOBt monohydrate (containing 14 wt.% water) (0.322 mmol; 2 eq.) and 62 mg EDC HCl (0.322 mmol; 2 eq.) were placed in a flask and suspended in 10 mL of dry DMF. 112 µL of DIPEA (0.645 mmol; 4 eq.) were added and the reaction stirred for 5 min while it was cooled via ice-water bath. 32 µL of cyclopropanamine

(0.322 mmol; 2 eq.) were added and the cooling was removed shortly after. HPLC monitoring showed full conversion after 24 h. The reaction was quenched with demin. H_2O after which the pure product precipitated. The product was filtered and dried, washed with Et_2O and further dried under high vacuum to yield compound (**21h**) as a white solid (61 mg; 92%). ^1H NMR (400 MHz, DMSO) δ 8.98 (s, 1H), 8.86 (s, 1H), 8.64 (d, J = 3.1 Hz, 1H), 8.58 (s, 1H), 8.20 (s, 1H), 8.12 (d, J = 8.3 Hz, 1H), 8.03 (d, J = 7.5 Hz, 1H), 7.95 (t, J = 7.8 Hz, 2H), 7.86 (s, 1H), 7.72 (d, J = 7.6 Hz, 1H), 7.67 – 7.52 (m, 4H), 7.48 (t, J = 7.8 Hz, 1H), 2.93 – 2.82 (m, 1H), 0.77 – 0.68 (m, 2H), 0.65 – 0.54 (m, 2H); ^{13}C NMR (101 MHz, DMSO) δ 166.7, 152.8, 139.7, 135.7, 134.4, 133.7, 133.1, 129.5, 128.4, 126.0, 125.9, 125.9, 125.7, 124.7, 124.5, 123.0, 121.4, 120.3, 117.4, 116.4, 116.2, 23.1, 5.8; FTIR [cm^{-1}]: 3267, 3083, 3047, 3005, 1640, 1586, 1558, 1528, 1491, 1384; TLC-MS(ESI) *m/z*: 444.9 $[M+H+MeOH]^+$; 410.0 $[M-H]^-$; 445.8 $[M+Cl]^-$; HRMS(ESI) *m/z*: calcd. for $[M+H]^+$: 412.17672; found: 412.1772; HPLC t_{ret}: 7.41 min.

N-cyclopentyl-3-(4-(3-(naphthalen-1-yl)ureido)-1*H*-pyrazol-1-yl)benzamide (**21i**)

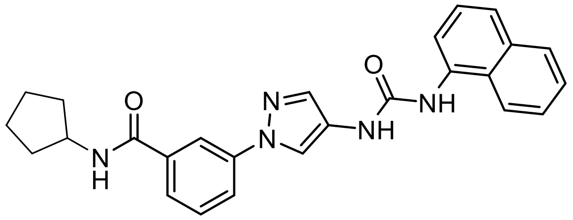

The reaction followed general procedure B1: Amide coupling using EDC HCl + HOBt.

57 mg of (**20** K^+-salt) (0.139 mmol; 1 eq.), 49 mg HOBt monohydrate (containing 14 wt.% water) (0.277 mmol; 2 eq.) and 53 mg EDC HCl (0.277 mmol; 2 eq.) were placed in a flask and suspended in 15 mL of dry DCM. 145 µL of DIPEA (0.833. mmol; 6 eq.) were added and the reaction stirred for 5 min while it was cooled via ice-water bath. 27 µL of cyclopentane amine (0.277 mmol; 2 eq.) were added and the cooling was removed shortly after. HPLC monitoring showed full conversion after 24 h. The solvent was evaporated and the solids were reconstituted in EtOAc. The organic phase was washed 1x with demin. H_2O, 1x with saturated $NaHCO_3$ (aq.) solution and 1x with saturated NH_4Cl (aq.) solution. The aqueous phase was 1x re-extracted. After drying over Na_2SO_4, filtering and evaporating the organic solvent the crude product was purified via flash chromatography (MeOH/DCM: 2/98 – 10/90) and dried in high vacuum to yield compound (**21i**) as a white solid (35 mg; 57%). ^1H NMR (400 MHz, DMSO) δ 8.99 (s, 1H), 8.87 (s, 1H), 8.59 (s, 1H), 8.50 (d, J = 7.2 Hz, 1H), 8.21 (s, 1H), 8.12 (d, J = 8.4 Hz, 1H), 8.04 (d, J = 7.1 Hz, 1H), 7.98 – 7.91 (m, J = 10.2, 4.2 Hz, 2H), 7.87 (s, 1H), 7.75 (d, J = 7.8 Hz, 1H), 7.65 (d, J = 8.2 Hz, 1H), 7.62 – 7.53 (m, J = 10.6, 6.8, 1.9 Hz, 3H), 7.48 (t, J = 7.9 Hz, 1H), 4.31 – 4.20 (m, 1H), 1.97 – 1.85 (m, 2H), 1.78 – 1.65 (m, 2H), 1.62 – 1.49 (m, J = 9.0, 4.6 Hz, 4H); ^{13}C NMR (101 MHz, DMSO) δ 165.2, 152.8, 139.7, 136.1, 134.4, 133.7, 133.1, 129.4, 128.4, 126.0, 125.9, 125.9, 125.7, 124.7, 123.0, 121.4, 120.2, 117.4, 116.5, 116.4, 51.0, 32.1, 23.7; FTIR [cm^{-1}]: 3284, 3248, 3058, 2954, 2866, 1646, 1633, 1584, 1542, 1486; TLC-MS(ESI) *m/z*: 438.2 $[M-H]^-$; HRMS(ESI) *m/z*: calcd. for $[M+H]^+$: 440.20803; found: 440.2076; HPLC t_{ret}: 8.50 min.

N-cyclohexyl-3-(4-(3-(naphthalen-1-yl)ureido)-1*H*-pyrazol-1-yl)benzamide (**21j**)

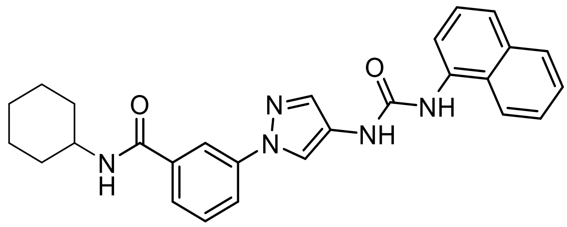

The reaction followed general procedure B1: Amide coupling using EDC HCl + HOBt.

60 mg of (**20**) (0.161 mmol; 1 eq.), 57 mg HOBt monohydrate (containing 14 wt.% water) (0.322 mmol; 2 eq.) and 62 mg EDC HCl (0.322 mmol; 2 eq.) were placed in a flask and suspended in 10 mL of dry DMF. 112 µL of DIPEA (0.645 mmol; 4 eq.) were added and the reaction stirred for 5 min while it was cooled via ice-water bath. 37 µL of cyclohexanamine (0.322 mmol; 2 eq.) were added and the cooling was removed shortly after. HPLC monitoring showed full conversion after 18 h. The reaction was quenched with demin. H_2O after which the crude product precipitated. The product was filtered and dried, washed with Et_2O. The product was dissolved in MeOH and purified via flash chromatography (MeOH/DCM: 0/100 – 10/90) and further dried under high vacuum to yield compound (**21j**) as a white solid (39 mg; 53%). ^{1}H NMR (400 MHz, DMSO) δ 8.98 (s, 1H), 8.86 (s, 1H), 8.58 (s, 1H), 8.41 (d, J = 7.9 Hz, 1H), 8.24 – 8.18 (m, 1H), 8.12 (d, J = 8.4 Hz, 1H), 8.05 – 8.00 (m, 1H), 7.97 – 7.91 (m, 2H), 7.86 (s, 1H), 7.75 (d, J = 7.8 Hz, 1H), 7.65 (d, J = 8.2 Hz, 1H), 7.62 – 7.52 (m, 3H), 7.48 (t, J = 7.9 Hz, 1H), 3.86 – 3.73 (m, J = 7.2, 3.4 Hz, 1H), 1.91 – 1.69 (m, 4H), 1.62 (d, J = 12.4 Hz, 1H), 1.40 – 1.24 (m, 4H), 1.19 – 1.06 (m, 1H); ^{13}C NMR (101 MHz, DMSO) δ 164.6, 152.8, 139.7, 136.2, 134.4, 133.7, 133.1, 129.5, 128.4, 126.0, 125.9, 125.9, 125.7, 124.7, 123.0, 121.4, 120.2, 117.4, 116.5, 116.4, 48.5, 32.4, 25.3, 25.0; FTIR [cm^{-1}]: 3273, 2930, 2852, 1633, 1585, 1539, 1486, 1388, 1328, 1236; TLC-MS(ESI) *m/z*: 476.0 [M+Na]$^{+}$; 452.1 [M-H]$^{-}$; 487.7 [M+Cl]$^{-}$; HRMS(ESI) *m/z*: calcd. for [M+H]$^{+}$: 454.22368; found: 454.2241; HPLC t_{ret}: 8.88 min.

(*RS*)-3-(4-(3-(naphthalen-1-yl)ureido)-1*H*-pyrazol-1-yl)-*N*-((tetrahydrofuran-2-yl)methyl)benzamide (**21k**)

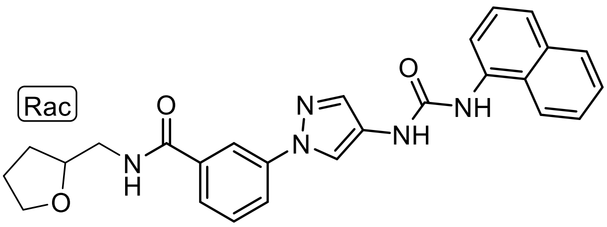

The reaction followed general procedure B1: Amide coupling using EDC HCl + HOBt.

60 mg of (**20**) (0.161 mmol; 1 eq.), 57 mg HOBt monohydrate (containing 14 wt.% water) (0.322 mmol; 2 eq.) and 62 mg EDC HCl (0.322 mmol; 2 eq.) were placed in a flask and suspended in 10 mL of dry THF. 112 µL of DIPEA (0.645 mmol; 4 eq.) were added and the reaction stirred for 5 min while it was cooled via ice-water bath. 33 µL of (tetrahydrofuran-2-yl)methanamine (0.322 mmol; 2 eq.) were added and the cooling was removed shortly after. HPLC monitoring showed full conversion after 24 h. The reaction was quenched with demin. H_2O after which the pure product precipitated. The product was filtered and dried, washed with Et_2O and further dried under high vacuum to yield compound (**21k**) as a white solid (52 mg; 71%). ^{1}H NMR (400 MHz, DMSO) δ 8.99 (s, 1H), 8.87 (s, 1H), 8.77 (t, J = 5.8 Hz, 1H), 8.60 (s, 1H), 8.25 (t, J = 1.7 Hz, 1H), 8.12 (d, J = 8.4 Hz, 1H), 8.04 (dd, J = 7.6, 0.8 Hz, 1H), 7.99 – 7.95 (m, 1H), 7.95 – 7.91 (m, 1H), 7.86 (s, 1H), 7.79 – 7.73 (m, 1H), 7.65 (d, J = 8.2 Hz, 1H), 7.62 – 7.52 (m, 3H), 7.48 (t, J = 7.9 Hz, 1H), 4.01 (p, J = 6.3 Hz, 1H), 3.83 – 3.75 (m, 1H), 3.64 (dd, J = 14.3, 7.6 Hz, 1H), 3.35 (t, J = 5.9 Hz, 2H), 1.98 – 1.75 (m, 3H), 1.65 – 1.55 (m, 1H); ^{13}C NMR (101 MHz, DMSO) δ 165.6, 152.8, 139.7, 135.7, 134.4, 133.7, 133.1, 129.6, 128.4, 126.0, 125.9, 125.9, 125.7, 124.7, 124.6, 123.0, 121.4, 120.3, 117.3, 116.5, 116.5, 77.1, 67.1, 43.5, 28.7, 25.1; FTIR [cm^{-1}]: 3260, 2952, 2924, 2855, 1639, 1585, 1540, 1482, 1383, 1314; TLC-MS(ESI) *m/z*: 454.0 [M-H]$^{-}$; 489.8 [M+Cl]$^{-}$; HRMS(ESI) *m/z*: calcd. for [M+H]$^{+}$: 456.20294; found: 456.2026; HPLC t_{ret}: 7.50 min.

(*RS*)-3-(4-(3-(naphthalen-1-yl)ureido)-1*H*-pyrazol-1-yl)-*N*-((tetrahydrofuran-3-yl)methyl)benzamide (**21l**)

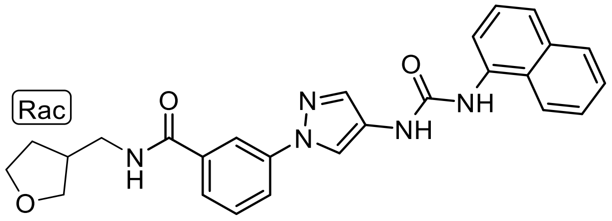

The reaction followed general procedure B1: Amide coupling using EDC HCl + HOBt.

60 mg of (**20**) (0.161 mmol; 1 eq.), 57 mg HOBt monohydrate (containing 14 wt.% water) (0.322 mmol; 2 eq.) and 62 mg EDC HCl (0.322 mmol; 2 eq.) were placed in a flask and suspended in 10 mL of dry THF. 112 µL of DIPEA (0.645 mmol; 4 eq.) were added and the reaction stirred for 5 min while it was cooled via ice-water bath. 33 µL of (tetrahydrofuran-3-yl)methanamine (0.322 mmol; 2 eq.) were added and the cooling was removed shortly after. HPLC monitoring showed full conversion after 24 h. The solvent was evaporated and the crude product was purified via flash chromatography (MeOH/DCM: 3/97 – 10/90) and further dried under high vacuum to yield compound (**21l**) as a white solid (40 mg; 54%). ^{1}H NMR (400 MHz, DMSO) δ 8.99 (s, 1H), 8.87 (s, 1H), 8.79 (t, J = 5.7 Hz, 1H), 8.59 (s, 1H), 8.27 – 8.20 (m, 1H), 8.12 (d, J = 8.4 Hz, 1H), 8.06 – 8.01 (m, 1H), 8.00 – 7.95 (m, 1H), 7.95 – 7.92 (m, 1H), 7.87 (s, 1H), 7.75 (d, J = 7.8 Hz, 1H), 7.65 (d, J = 8.2 Hz, 1H), 7.62 – 7.52 (m, 3H), 7.48 (t, J = 7.9 Hz, 1H), 3.76 (td, J = 8.0, 5.8 Hz, 1H), 3.70 (dd, J = 8.5, 7.0 Hz, 1H), 3.63 (dd, J = 14.8, 7.9 Hz, 1H), 3.51 (dd, J = 8.5, 5.2 Hz, 1H), 3.33 – 3.20 (m, 2H), 2.54 (d, J = 5.8 Hz, 1H), 2.02 – 1.90 (m, 1H), 1.68 – 1.57 (m, 1H); ^{13}C NMR (101 MHz, DMSO) δ 165.6, 152.8, 139.8, 135.8, 134.4, 133.7, 133.1, 129.6, 128.4, 126.0, 125.9, 125.9, 125.7, 124.7, 124.6, 123.0, 121.4, 120.3, 117.4, 116.4, 116.3, 70.5, 66.8, 42.0, 38.9, 29.5; FTIR [cm^{-1}]: 3288, 3253, 3101, 3070, 2959, 2850, 1647, 1584, 1546, 1483; TLC-MS(ESI) *m/z*: 478.2 [M+Na]$^{+}$; 454.2 [M-H]$^{-}$; 489.8 [M+Cl]$^{-}$; HRMS(ESI) *m/z*: calcd. for [M+H]$^{+}$: 456.20294; found: 456.2033; HPLC t_{ret}: 7.20 min.

(*RS*)-3-(4-(3-(naphthalen-1-yl)ureido)-1*H*-pyrazol-1-yl)-*N*-(pyrrolidin-3-yl)benzamide (**21m**) (**HCl salt**)

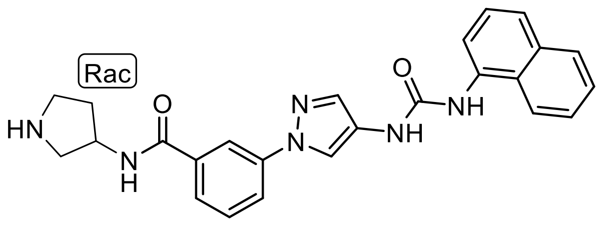

The reaction followed general procedure B1(step 1) + F(step 2) : Amide coupling using EDC HCl + HOBt + Boc deprotection

Step 1: 60 mg of (**20**) (0.161 mmol; 1 eq.), 62 mg of EDC HCl (0.322 mmol; 2 eq.) and 57 mg of HOBt monohydrate (containing 14 wt.% water) (0.322 mmol; 2 eq.) were dissolved in 10 mL of dry DMF, while cooling the mixture via ice-water bath. Subsequently 112 µL of DIPEA (0.645 mmol; 4 eq.) were added to the solution and the reaction stirred for 5 min. 60 mg of (*RS*)-*tert*-butyl 3-aminopyrrolidine-1-carboxylate (0.322 mmol; 2 eq.) were added, after which the cooling was removed and the solution was allowed to warm up to RT. Monitoring via HPLC showed total conversion after 24 h. The reaction was quenched with demin. H_2O. The pure intermediate product precipitated, was filtered off and washed with demin. H_2O and Et_2O. This intermediate was directly subjected to step 2.

Step 2: The intermediate from step 1 was dissolved in 10 mL of EtOH and 0.645 mL of 1.25 M HCl (EtOH) (0.806 mmol; 5 eq.) were added. The reaction was heated to 60 °C for 20 h. The solvent was evaporated to yield compound (**21m**) as a beige-yellow solid (45 mg; 59%; **HCl**

salt). ^{1}H NMR (400 MHz, DMSO) δ 9.62 (s, 1H), 9.35 – 9.17 (m, J = 17.4 Hz, 3H), 8.95 (d, J = 6.3 Hz, 1H), 8.60 (s, 1H), 8.34 – 8.29 (m, J = 7.9 Hz, 2H), 8.08 (d, J = 7.4 Hz, 1H), 8.00 (dd, J = 8.1, 1.1 Hz, 1H), 7.94 – 7.89 (m, 1H), 7.86 (s, 1H), 7.80 (d, J = 7.7 Hz, 1H), 7.64 – 7.51 (m, 4H), 7.47 (t, J = 7.9 Hz, 1H), 4.62 – 4.53 (m, 1H), 3.48 – 3.35 (m, 2H), 3.31 – 3.20 (m, 2H), 2.22 (dq, J = 14.8, 7.5 Hz, 1H), 2.06 (td, J = 13.0, 6.0 Hz, 1H); ^{13}C NMR (101 MHz, DMSO) δ 165.8, 153.0, 139.7, 135.2, 134.7, 133.7, 133.1, 129.6, 128.3, 125.9, 125.8, 125.6, 124.9, 124.8, 122.6, 121.7, 120.6, 116.7, 116.5, 116.3, 49.2, 49.1, 43.6, 29.8; FTIR [cm^{-1}]: 3246, 2958, 2748, 2087, 1647, 1539, 1490, 1388, 1341, 1252; TLC-MS(ESI) *m/z*: 441.4 $[M+H]^+$; 439.4 $[M-H]^-$; 475.3 $[M+Cl]^-$; HRMS(ESI) *m/z*: calcd. for $[M+H]^+$: 441.20327; found: 441.2032; HPLC t_{ret}: 4.97 min.

3-(4-(3-(naphthalen-1-yl)ureido)-1*H*-pyrazol-1-yl)-*N*-(piperidin-4-yl)benzamide (**21n**) (**HCl salt**)

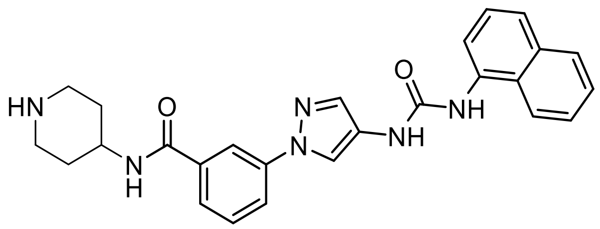

The reaction followed general procedure B1(step 1) + F(step 2) : Amide coupling using EDC HCl + HOBt + Boc deprotection

Step 1: 60 mg of (**20**) (0.161 mmol; 1 eq.), 62 mg of EDC HCl (0.322 mmol; 2 eq.) and 57 mg of HOBt monohydrate (containing 14 wt.% water) (0.322 mmol; 2 eq.) were dissolved in 10 mL of dry DMF, while cooling the mixture via ice-water bath. Subsequently 112 µL of DIPEA (0.645 mmol; 4 eq.) were added to the solution and the reaction stirred for 5 min. 64 mg of (*RS*)-*tert*-butyl 4-aminopiperidine-1-carboxylate (0.322 mmol; 2 eq.) were added, after which the cooling was removed and the solution was allowed to warm up to RT. Monitoring via HPLC showed total conversion after 24 h. The reaction was quenched with demin. H_2O. The pure intermediate product precipitated, was filtered off and washed with demin. H_2O and Et_2O. This intermediate was directly subjected to step 2.

Step 2: The intermediate from step 1 was dissolved in 10 mL of EtOH and 0.645 mL of 1.25 M HCl (EtOH) (0.806 mmol; 5 eq.) were added. The reaction was heated to 60 °C for 20 h. The solvent was evaporated to yield compound (**21n**) as a beige solid (66 mg; 83%; **HCl salt**). ^{1}H NMR (400 MHz, DMSO) δ 9.72 (s, 1H), 9.31 (s, 1H), 8.99 – 8.81 (m, 2H), 8.73 (d, J = 7.4 Hz, 1H), 8.58 (s, 1H), 8.35 (d, J = 7.9 Hz, 1H), 8.26 (s, 1H), 8.09 (d, J = 7.4 Hz, 1H), 7.98 (dd, J = 8.1, 1.1 Hz, 1H), 7.94 – 7.89 (m, 1H), 7.84 (s, 1H), 7.77 (d, J = 7.7 Hz, 1H), 7.62 (d, J = 8.1 Hz, 1H), 7.59 – 7.51 (m, 3H), 7.47 (t, J = 7.9 Hz, 1H), 4.16 – 4.05 (m, 1H), 3.32 (d, J = 12.5 Hz, 2H), 3.02 (dd, J = 21.8, 11.5 Hz, 2H), 2.05 – 1.94 (m, 2H), 1.88 – 1.75 (m, 2H); ^{13}C NMR (101 MHz, DMSO) δ 165.2, 153.0, 139.7, 135.6, 134.7, 133.8, 133.0, 129.5, 128.3, 125.9, 125.8, 125.6, 124.9, 124.8, 122.6, 121.8, 120.4, 116.7, 116.5, 116.2, 44.5, 42.2, 28.2; FTIR [cm^{-1}]: 3241, 3035, 2928, 2725, 2486, 1583, 1540, 1488, 1388, 1341; TLC-MS(ESI) *m/z*: 455.4 $[M+H]^+$; 477.4 $[M+Na]^+$; 453.4 $[M-H]^-$; 489.5 $[M+Cl]^-$; HRMS(ESI) *m/z*: calcd. for $[M+H]^+$: 455.21892; found: 455.2177; HPLC t_{ret}: 5.06 min.

(*RS*)-3-(4-(3-(naphthalen-1-yl)ureido)-1*H*-pyrazol-1-yl)-*N*-(pyrrolidin-3-ylmethyl)benzamide (**21o**) (**HCl salt**)

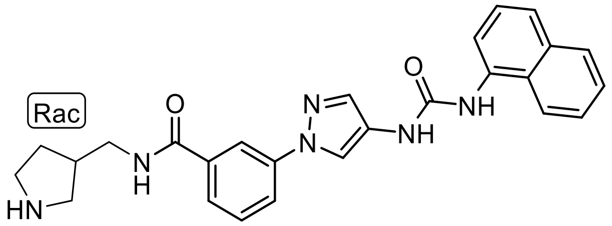

The reaction followed general procedure B1(step 1) + F(step 2) : Amide coupling using EDC HCl + HOBt + Boc deprotection

Step 1: 60 mg of (**20**) (0.161 mmol; 1 eq.), 62 mg of EDC HCl (0.322 mmol; 2 eq.) and 57 mg of HOBt monohydrate (containing 14 wt.% water) (0.322 mmol; 2 eq.) were dissolved in 10 mL of dry DMF, while cooling the mixture via ice-water bath. Subsequently 112 µL of DIPEA (0.645 mmol; 4 eq.) were added to the solution and the reaction stirred for 5 min. 64 mg of (*RS*)-*tert*-butyl 3-(aminomethyl)pyrrolidine-1-carboxylate (0.322 mmol; 2 eq.) were added, after which the cooling was removed and the solution was allowed to warm up to RT. Monitoring via HPLC showed total conversion after 24 h. The reaction was quenched with demin. H_2O. The pure intermediate product precipitated, was filtered off and washed with demin. H_2O and Et_2O. This intermediate was directly subjected to step 2.

Step 2: The intermediate from step 1 was dissolved in 10 mL of EtOH and 0.645 mL of 1.25 M HCl (EtOH) (0.806 mmol; 5 eq.) were added. The reaction was heated to 60 °C for 20 h. The solvent was evaporated to yield compound (**21o**) as a beige solid (14 mg; 18%; **HCl salt**). ^{1}H NMR (400 MHz, DMSO) δ 9.68 (s, 1H), 9.29 (s, 1H), 9.09 (br s, 2H), 8.93 (t, J = 5.7 Hz, 1H), 8.59 (s, 1H), 8.34 (d, J = 7.9 Hz, 1H), 8.28 (s, 1H), 8.08 (d, J = 7.2 Hz, 1H), 7.99 (dd, J = 8.1, 1.3 Hz, 1H), 7.94 – 7.89 (m, 1H), 7.84 (s, 1H), 7.77 (d, J = 7.8 Hz, 1H), 7.64 – 7.51 (m, 4H), 7.47 (t, J = 7.9 Hz, 1H), 3.41 – 3.34 (m, 2H), 3.32 – 3.19 (m, 2H), 3.17 – 3.07 (m, 1H), 2.97 – 2.89 (m, 1H), 2.61 – 2.54 (m, 1H), 2.08 – 1.97 (m, 1H), 1.69 (dq, J = 13.0, 8.2 Hz, 1H); ^{13}C NMR (101 MHz, DMSO) δ 165.9, 153.0, 139.8, 135.5, 134.7, 133.7, 133.0, 129.6, 128.3, 125.9, 125.8, 125.6, 124.9, 124.6, 122.6, 121.8, 120.4, 116.7, 116.2, 47.7, 44.3, 40.9, 37.9, 27.7; FTIR [cm^{-1}]: 3261, 3047, 2751, 1636, 1539, 1490, 1388, 1342, 1252, 1169; TLC-MS(ESI) *m/z*: 455.4 $[M+H]^+$; HRMS(ESI) *m/z*: calcd. for $[M+H]^+$: 455.21892; found: 455.2182; HPLC t_{ret}: 5.06 min.

N-allyl-3-(4-(3-(naphthalen-1-yl)ureido)-1*H*-pyrazol-1-yl)benzamide (**21p**)

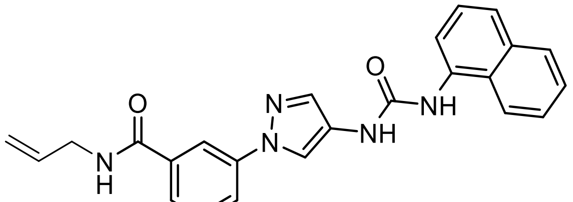

The reaction followed general procedure B1: Amide coupling using EDC HCl + HOBt.

70 mg of (**20**) (0.188 mmol; 1 eq.), 67 mg HOBt monohydrate (containing 14 wt.% water) (0.376 mmol; 2 eq.) and 72 mg EDC HCl (0.376 mmol; 2 eq.) were placed in a flask and suspended in 8 mL of dry DMF. 131 µL of DIPEA (0.752 mmol; 4 eq.) were added and the reaction stirred for 5 min while it was cooled via ice-water bath. 28 µL of prop-2-en-1-amine (0.376 mmol; 2 eq.) were added and the cooling was removed shortly after. HPLC monitoring showed full conversion after 18 h. The reaction was quenched with demin. H_2O after which the pure product precipitated. The product was filtered and dried, washed with Et_2O and further dried under high vacuum to yield compound (**21p**) as a white solid (57 mg; 74%). ^{1}H NMR (400 MHz, DMSO) δ 9.00 (br s, 1H), 8.88 (br s, 2H), 8.61 (s, 1H), 8.27 (t, J = 1.7 Hz, 1H), 8.12 (d, J = 8.4 Hz, 1H), 8.06 – 8.01 (m, 1H), 7.98 (dd, J = 8.1, 1.3 Hz, 1H), 7.95 – 7.91 (m, 1H), 7.86

(s, 1H), 7.78 (d, J = 7.9 Hz, 1H), 7.65 (d, J = 8.2 Hz, 1H), 7.62 – 7.52 (m, 3H), 7.48 (t, J = 7.9 Hz, 1H), 5.93 (ddt, J = 17.1, 10.4, 5.3 Hz, 1H), 5.20 (dq, J = 17.2, 1.7 Hz, 1H), 5.11 (dq, J = 10.3, 1.5 Hz, 1H), 3.95 (s, 2H); ^{13}C NMR (101 MHz, DMSO) δ 165.3, 152.8, 139.8, 135.7, 135.3, 134.4, 133.7, 133.1, 129.6, 128.4, 126.0, 125.9, 125.9, 125.7, 124.8, 124.6, 123.0, 121.4, 120.4, 117.3, 116.4, 116.2, 115.3, 41.6; FTIR [cm^{-1}]: 3285, 3232, 3057, 2906, 1644, 1584, 1539, 1488, 1395, 1344; TLC-MS(ESI) *m/z*: 434.1 [M+Na]$^+$; 410.2 [M-H]$^-$; 446.3 [M+Cl]$^-$; HRMS(ESI) *m/z*: calcd. for [M+H]$^+$: 412.17672; found: 412.1766; HPLC t_{ret}: 7.55 min.

N-(2-methylallyl)-3-(4-(3-(naphthalen-1-yl)ureido)-1*H*-pyrazol-1-yl)benzamide (**21q**)

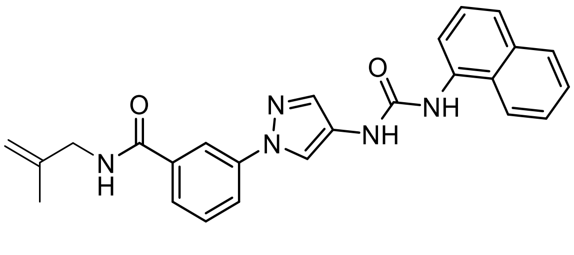

The reaction followed general procedure B1: Amide coupling using EDC HCl + HOBt.

70 mg of (**20**) (0.188 mmol; 1 eq.), 67 mg HOBt monohydrate (containing 14 wt.% water) (0.376 mmol; 2 eq.) and 72 mg EDC HCl (0.376 mmol; 2 eq.) were placed in a flask and suspended in 8 mL of dry DMF. 131 µL of DIPEA (0.752 mmol; 4 eq.) were added and the reaction stirred for 5 min while it was cooled via ice-water bath. 34 µL of 2-methylprop-2-en-1-amine (0.376 mmol; 2 eq.) were added and the cooling was removed shortly after. HPLC monitoring showed full conversion after 18 h. The reaction was quenched with demin. H_2O after which the pure product precipitated. The product was filtered and dried, washed with Et_2O and further dried under high vacuum to yield compound (**21r**) as a white solid (62 mg; 78%). ^{1}H NMR (400 MHz, DMSO) δ 8.99 (s, 1H), 8.91 – 8.81 (m, 2H), 8.61 (s, 1H), 8.28 (s, 1H), 8.12 (d, J = 8.3 Hz, 1H), 8.03 (d, J = 7.5 Hz, 1H), 7.98 (d, J = 7.9 Hz, 1H), 7.94 (d, J = 7.9 Hz, 1H), 7.86 (s, 1H), 7.78 (d, J = 7.5 Hz, 1H), 7.65 (d, J = 8.1 Hz, 1H), 7.62 – 7.53 (m, 3H), 7.48 (t, J = 7.8 Hz, 1H), 4.84 (d, J = 11.0 Hz, 2H), 3.87 (d, J = 5.1 Hz, 2H), 1.74 (s, 3H); ^{13}C NMR (101 MHz, DMSO) δ 165.3, 152.8, 142.5, 139.8, 135.7, 134.4, 133.7, 133.1, 129.6, 128.4, 126.0, 125.9, 125.9, 125.7, 124.7, 124.6, 123.0, 121.4, 120.4, 117.4, 116.5, 116.2, 110.0, 44.6, 20.3; FTIR [cm^{-1}]: 3271, 3069, 2971, 2915, 1642, 1585, 1543, 1485, 1395, 1343; TLC-MS(ESI) *m/z*: 448.2 [M+Na]$^+$; 424.2 [M-H]$^-$; 460.2 [M+Cl]$^-$; HRMS(ESI) *m/z*: calcd. for [M+H]$^+$: 426.19238; found: 426.1925; HPLC t_{ret}: 7.99 min.

3-(4-(3-(naphthalen-1-yl)ureido)-1*H*-pyrazol-1-yl)-*N*-(prop-2-yn-1-yl)benzamide (**21r**)

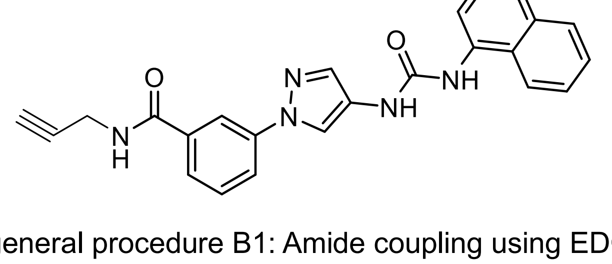

The reaction followed general procedure B1: Amide coupling using EDC HCl + HOBt.

70 mg of (**20**) (0.188 mmol; 1 eq.), 67 mg HOBt monohydrate (containing 14 wt.% water) (0.376 mmol; 2 eq.) and 72 mg EDC HCl (0.376 mmol; 2 eq.) were placed in a flask and suspended in 8 mL of dry DMF. 131 µL of DIPEA (0.752 mmol; 4 eq.) were added and the reaction stirred for 5 min while it was cooled via ice-water bath. 24 µL of prop-2-yn-1-amine (0.376 mmol; 2 eq.) were added and the cooling was removed shortly after. HPLC monitoring showed full conversion after 18 h. The reaction was quenched with demin. H_2O after which the

pure product precipitated. The product was filtered and dried, washed with Et_2O and further dried under high vacuum to yield compound (**21r**) as a cream-colored solid (70 mg; 91%). ^1H NMR (400 MHz, DMSO) δ 9.16 (t, J = 5.4 Hz, 1H), 9.01 (s, 1H), 8.88 (s, 1H), 8.62 (s, 1H), 8.27 (s, 1H), 8.12 (d, J = 8.3 Hz, 1H), 8.04 (d, J = 7.5 Hz, 1H), 8.00 (dd, J = 8.1, 1.2 Hz, 1H), 7.94 (d, J = 7.8 Hz, 1H), 7.87 (s, 1H), 7.77 (d, J = 7.7 Hz, 1H), 7.65 (d, J = 8.2 Hz, 1H), 7.62 – 7.53 (m, 3H), 7.48 (t, J = 7.9 Hz, 1H), 4.11 (dd, J = 5.3, 2.3 Hz, 2H), 3.17 (t, J = 2.3 Hz, 1H); ^{13}C NMR (101 MHz, DMSO) δ 165.2, 152.8, 139.8, 135.0, 134.5, 133.8, 133.1, 129.8, 128.4, 126.0, 125.9, 125.9, 125.7, 124.8, 124.6, 123.0, 121.4, 120.6, 117.3, 116.4, 116.3, 81.2, 73.1, 28.6; FTIR [cm^{-1}]: 3270, 3092, 3047, 1639, 1585, 1559, 1534, 1485, 1394, 1342; TLC-MS(ESI) *m/z*: 432.2 [M+Na]+; 464.2 $[M+Na+MeOH]^+$; 408.3 $[M-H]^-$; 444.3 $[M+Cl]^-$; HRMS(ESI) *m/z*: calcd. for $[M+H]^+$: 410.16107; found: 410.1605; HPLC t_{ret}: 7.17 min.

10.12 Compound synthesis additional compounds:

-(4-(3-(naphthalen-1-yl)ureido)-1*H*-pyrazol-1-yl)-*N*-(2,2,2-trifluoroethyl)benzamide (**SI-I**)

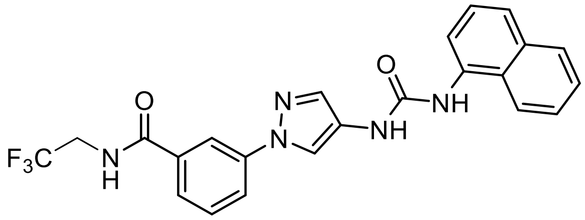

The reaction followed general procedure B1: Amide coupling using EDC HCl + HOBt.

70 mg of (**20**) (0.188 mmol; 1 eq.), 67 mg HOBt monohydrate (containing 14 wt.% water) (0.376 mmol; 2 eq.) and 72 mg EDC HCl (0.376 mmol; 2 eq.) were placed in a flask and suspended in 10 mL of dry DMF. 131 µL of DIPEA (0.752 mmol; 4 eq.) were added and the reaction stirred for 5 min while it was cooled via ice-water bath. 30 µL of 2,2,2-trifluoroethan-1-amine (0.376 mmol; 2 eq.) were added and the cooling was removed shortly after. HPLC monitoring showed full conversion after 18 h. The reaction was quenched with demin. H_2O after which the pure product precipitated. The product was filtered and dried, washed with Pentane and further dried under high vacuum to yield compound (**SI-I**) as an off-white solid (59 mg; 69%). ^{1}H NMR (400 MHz, DMSO) δ 9.31 (t, J = 6.1 Hz, 1H), 9.02 (s, 1H), 8.89 (s, 1H), 8.62 (s, 1H), 8.30 (s, 1H), 8.13 (d, J = 8.3 Hz, 1H), 8.07 – 8.00 (m, 2H), 7.94 (d, J = 7.9 Hz, 1H), 7.88 (s, 1H), 7.80 (d, J = 7.7 Hz, 1H), 7.66 – 7.53 (m, 4H), 7.48 (t, J = 7.9 Hz, 1H), 4.14 (qd, J = 9.7, 6.6 Hz, 2H); ^{13}C NMR (101 MHz, DMSO) δ 166.2, 152.8, 139.9, 134.5, 134.4, 133.7, 133.2, 129.9, 128.4, 126.0, 125.9, 125.9, 125.7, 124.8, 124.8 (q, J = 279.4 Hz),124.8, 123.0, 121.4, 121.0, 117.4, 116.4, 40.8 – 39.7 (m); FTIR [cm^{-1}]: 3252, 3069, 2958, 1644, 1585, 1545, 1485, 1395, 1319, 1262; TLC-MS(ESI) *m/z*: 452.1 [M-H]-; 488.1 $[M+Cl]^-$; HRMS(ESI) *m/z*: calcd. for $[M+H]^+$: 454.14846; found: 454.1492; HPLC t_{ret}: 7.94 min.

1-(1-(3-((1S,4S)-2-oxa-5-azabicyclo[2.2.1]heptane-5-carbonyl)phenyl)-1H-pyrazol-4-yl)-3-(naphthalen-1-yl)urea (**SI-II**)

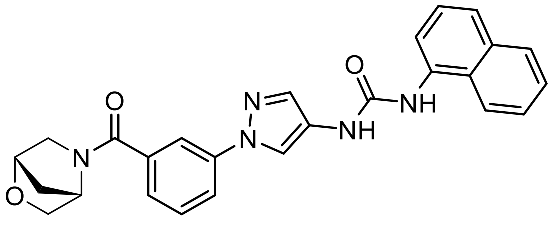

The reaction followed general procedure B1: Amide coupling using EDC HCl + HOBt.

60 mg of (**20**) (0.161 mmol; 1 eq.), 57 mg HOBt monohydrate (containing 14 wt.% water) (0.322 mmol; 2 eq.) and 62 mg EDC HCl (0.322 mmol; 2 eq.) were placed in a flask and suspended in 10 mL of dry THF. 168 µL of DIPEA (0.967 mmol; 6 eq.) were added and the reaction stirred for 5 min while it was cooled via ice-water bath. 44 mg of (1*S*,4*S*)-2-oxa-5-azabicyclo[2.2.1]heptan-5-ium chloride (0.322 mmol; 2 eq.) were added and the cooling was removed shortly after. HPLC monitoring showed full conversion after 24 h. The reaction was quenched with demin. H_2O. The mixture was extracted 3x with EtOAc. The organic phase was washed 1x with 1 M HCl (aq.) solution and 2x with saturated $NaHCO_3$ (aq.) solution. After drying the organic phase over Na_2SO_4, filtering and evaporating the organic solvent the crude product was purified via flash chromatography (MeOH/DCM: 1/99 – 10/90) and dried in high vacuum to yield compound (**SI-II**) as white needles (46 mg; 63%). ^{1}H NMR (400 MHz, DMSO) δ 9.01 (d, J = 3.9 Hz, 1H), 8.89 (s, 1H), 8.56 (s, 1H), 8.13 (d, J = 8.4 Hz, 1H), 8.04 (d, J = 7.6

Hz, 1H), 7.96 – 7.85 (m, 4H), 7.64 (d, J = 8.2 Hz, 1H), 7.62 – 7.51 (m, 3H), 7.48 (t, J = 7.9 Hz, 1H), 7.39 (dd, J = 20.3, 7.6 Hz, 1H), 4.77 (d, J = 74.9 Hz, 1H), 4.48 (d, J = 75.2 Hz, 1H), 3.89 (dd, J = 24.2, 7.3 Hz, 1H), 3.73 (ddd, J = 33.8, 7.5, 1.2 Hz, 1H), 3.57 – 3.48 (m, 1H), 3.31 (dd, J = 32.8, 10.8 Hz, 1H), 1.93 (ddd, J = 20.8, 10.0, 1.6 Hz, 1H), 1.80 (dd, J = 31.8, 9.9 Hz, 1H); ^{13}C NMR (101 MHz, DMSO) δ 167.9, 167.0, 152.8, 139.8, 139.7, 137.7, 137.3, 134.5, 133.7, 133.4, 133.3, 133.3, 129.8, 129.7, 129.1, 128.8, 128.4, 126.0, 125.9, 125.9, 125.7, 124.8, 124.7, 124.3, 124.1, 122.9, 121.4, 119.3, 119.0, 117.3, 117.2, 116.5, 116.5, 116.4, 116.3, 75.6, 75.1, 73.6, 73.0, 59.9, 57.7, 56.2, 54.3, 36.7, 35.0 (complex splitting of signals probably because of rotational isomers); FTIR [cm^{-1}]: 3292, 3053, 2950, 2876, 1701, 1542, 1465, 1388, 1251, 1206; TLC-MS(ESI) *m/z*: 475.8 [M+Na]$^+$; 451.8 [M-H]$^-$; 487.8 [M+Cl]$^-$; HRMS(ESI) *m/z*: calcd. for [M+H]$^+$: 454.18729; found: 454.1881; HPLC t_{ret}: 6.70 min.

N-(2-morpholinoethyl)-3-(4-(3-(naphthalen-1-yl)ureido)-1*H*-pyrazol-1-yl)benzamide (**SI-III**)

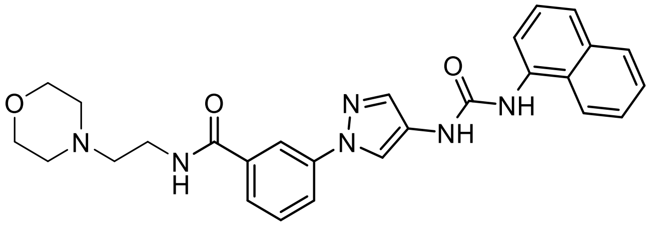

The reaction followed general procedure B1: Amide coupling using EDC HCl + HOBt.

40 mg of (**20**) (0.107 mmol; 1 eq.), 38 mg HOBt monohydrate (containing 14 wt.% water) (0.215 mmol; 2 eq.) and 41 mg EDC HCl (0.215 mmol; 2 eq.) were placed in a flask and suspended in 10 mL of dry THF. 56 µL of DIPEA (0.322 mmol; 3 eq.) were added and the reaction stirred for 5 min while it was cooled via ice-water bath. 28 µL of 2-morpholinoethan-1-amine (0.215 mmol; 2 eq.) were added and the cooling was removed shortly after. HPLC monitoring showed full conversion after 24 h. The reaction was quenched with MeOH and the organic solvent was evaporated. The residual solids were purified via flash chromatography (MeOH/DCM: 4/96 – 10/90 + 2M NH_3) and dried in high vacuum to yield compound (**SI-III**) as an white solid (14 mg; 27%). ^{1}H NMR (400 MHz, DMSO) δ 9.02 (s, 1H), 8.89 (s, 1H), 8.64 (t, J = 5.6 Hz, 1H), 8.59 (s, 1H), 8.26 – 8.21 (m, 1H), 8.12 (d, J = 8.3 Hz, 1H), 8.03 (d, J = 7.3 Hz, 1H), 7.99 – 7.92 (m, 2H), 7.86 (s, 1H), 7.74 (d, J = 7.8 Hz, 1H), 7.65 (d, J = 8.2 Hz, 1H), 7.62 – 7.53 (m, 3H), 7.48 (t, J = 7.9 Hz, 1H), 3.58 (t, 4H), 3.42 (dd, J = 13.0, 6.6 Hz, 2H), 2.49 – 2.46 (m, 2H), 2.45 – 2.39 (m, 4H); ^{13}C NMR (101 MHz, DMSO) δ 165.4, 152.8, 139.8, 135.8, 134.4, 133.7, 133.1, 129.6, 128.4, 126.0, 125.9, 125.9, 125.7, 124.8, 124.5, 123.0, 121.4, 120.3, 117.3, 116.4, 116.2, 66.2, 57.4, 53.3, 36.7; FTIR [cm^{-1}]: 3259, 3053, 2926, 2851, 2810, 1636, 1583, 1540, 1487, 1388; TLC-MS(ESI) *m/z*: 484.8 [M+Na]$^+$; 506.9 [M+Na]$^+$; 482.8 [M-H]$^-$; 518.6 [M+Cl]$^-$; HRMS(ESI) *m/z*: calcd. for [M+H]$^+$: 485.22949; found: 485.2292; HPLC t_{ret}: 4.91 min.

N-(3-morpholinopropyl)-3-(4-(3-(naphthalen-1-yl)ureido)-1*H*-pyrazol-1-yl)benzamide (**SI-IV**)

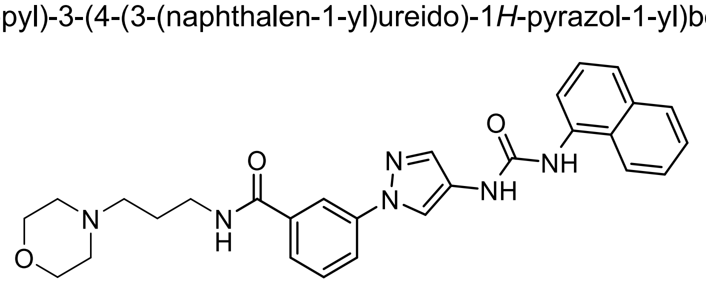

The reaction followed general procedure B1: Amide coupling using EDC HCl + HOBt.

50 mg of (**20** K^+ salt) (0.122 mmol; 1 eq.), 43 mg HOBt monohydrate (containing 14 wt.% water) (0.244 mmol; 2 eq.) and 47 mg EDC HCl (0.244 mmol; 2 eq.) were placed in a flask and suspended in 10 mL of dry DMF. 56 µL of DIPEA (0.322 mmol; 3 eq.) were added and the reaction stirred for 5 min while it was cooled via ice-water bath. 32 µL of 3-morpholinopropan-1-amine (0.244 mmol; 2 eq.) were added and the cooling was removed shortly after. HPLC monitoring showed full conversion after 24 h. The reaction was quenched with 1 M NaOH (aq.) solution. The mixture was extracted 3x with EtOAc. After drying the organic phase over Na_2SO_4, filtering and evaporating the organic solvent, the crude product was purified via flash chromatography (MeOH/DCM: 3/97 – 15/85 + 2 M NH_3) and dried in high vacuum to yield compound (**SI-IV**) as a white-yellow solid (16 mg; 26%). ^{1}H NMR (400 MHz, DMSO) δ 9.00 (s, 1H), 8.87 (s, 1H), 8.68 (t, J = 5.5 Hz, 1H), 8.59 (s, 1H), 8.25 – 8.20 (m, 1H), 8.12 (d, J = 8.4 Hz, 1H), 8.03 (dd, J = 7.6, 0.8 Hz, 1H), 7.98 – 7.91 (m, 2H), 7.86 (s, 1H), 7.74 (d, J = 7.9 Hz, 1H), 7.65 (d, J = 8.2 Hz, 1H), 7.62 – 7.53 (m, 3H), 7.48 (t, J = 7.9 Hz, 1H), 3.60 – 3.54 (m, 4H), 3.38 – 3.25 (m, 2H), 2.43 – 2.27 (m, 6H), 1.77 – 1.66 (m, 2H); 13C NMR (101 MHz, DMSO) δ 165.35, 152.79, 139.76, 135.93, 134.43, 133.74, 133.08, 129.59, 128.41, 125.99, 125.91, 125.88, 125.71, 124.74, 124.48, 122.98, 121.39, 120.20, 117.34, 116.40, 116.18, 66.21, 56.07, 53.36, 37.85, 25.97; FTIR [cm^{-1}]: 3297, 3056, 2924, 2851, 2810, 1629, 1582, 1540, 1488, 1388; TLC-MS(ESI) *m/z*: 498.9 $[M+H]^+$; 520.6 $[M+Na]^+$; 496.7 $[M-H]^-$; 532.7 $[M+Cl]^-$; HRMS(ESI) *m/z*: calcd. for $[M+H]^+$: 499.24514; found: 499.2429; HPLC t_{ret}: 5.01 min.

N-(2-(2-hydroxyethoxy)ethyl)-3-(4-(3-(naphthalen-1-yl)ureido)-1*H*-pyrazol-1-yl)benzamide (**SI-V**)

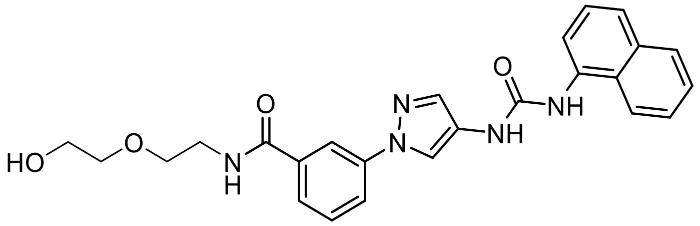

The reaction followed general procedure B1: Amide coupling using EDC HCl + HOBt.

70 mg of (**20**) (0.188 mmol; 1 eq.), 67 mg HOBt monohydrate (containing 14 wt.% water) (0.376 mmol; 2 eq.) and 72 mg EDC HCl (0.376 mmol; 2 eq.) were placed in a flask and suspended in 10 mL of dry THF. 131 µL of DIPEA (0.752 mmol; 4 eq.) were added and the reaction stirred for 5 min while it was cooled via ice-water bath. 38 µL of 2-(2-aminoethoxy)ethan-1-ol (0.376 mmol; 2 eq.) were added and the cooling was removed shortly after. HPLC monitoring showed full conversion after 20 h. The reaction was quenched with demin. H_2O and 10% HCl (aq.) solution. The mixture was extracted 3x with EtOAc. The organic phase was washed 2x with 1 M NaOH (aq.) solution and 1x with brine. After drying the organic phase over Na_2SO_4, filtering and evaporating the organic solvent, the crude product was purified via flash chromatography (MeOH/DCM: 2/98 – 10/90) and dried in high vacuum to yield compound (**SI-V**) as a white solid (48 mg; 56%). ^{1}H NMR (400 MHz, DMSO) δ 8.99 (s, 1H), 8.87 (s, 1H), 8.73 (t, J = 5.5 Hz, 1H), 8.60 (s, 1H), 8.28 – 8.23 (m, 1H), 8.12 (d, J = 8.4 Hz, 1H), 8.04 (dd, J = 7.6, 0.7 Hz, 1H), 7.97 (ddd, J = 8.1, 2.2, 0.8 Hz, 1H), 7.95 – 7.91 (m, 1H), 7.87 (s, 1H), 7.78 – 7.73 (m, 1H), 7.65 (d, J = 8.2 Hz, 1H), 7.62 – 7.53 (m, 3H), 7.48 (t, J = 7.9 Hz, 1H), 4.60 (t, J = 5.3 Hz, 1H), 3.57 (t, J = 6.0 Hz, 2H), 3.54 – 3.45 (m, 6H); ^{13}C NMR (101 MHz, DMSO) δ 165.6, 152.8, 139.8, 135.7, 134.4, 133.7, 133.1, 129.6, 128.4, 126.0, 125.9, 125.9, 125.7, 124.7, 124.5, 123.0, 121.4, 120.3, 117.4, 116.4, 116.3, 72.2, 68.9, 60.2, 39.4; FTIR [cm^{-1}]: 3265, 2925, 2857, 1639, 1586, 1539, 1484, 1388, 1342, 1307; TLC-MS(ESI) *m/z*: 484.2 $[M+Na]^+$; 458.0 $[M-H]^-$; 494.1 $[M+Cl]^-$; HRMS(ESI) *m/z*: calcd. for $[M+H]^+$: 460.19785; found: 460.1982; HPLC t_{ret}: 6.66 min.

N-(2-(2-(2-hydroxyethoxy)ethoxy)ethyl)-3-(4-(3-(naphthalen-1-yl)ureido)-1*H*-pyrazol-1-yl)benzamide (**SI-VI**)

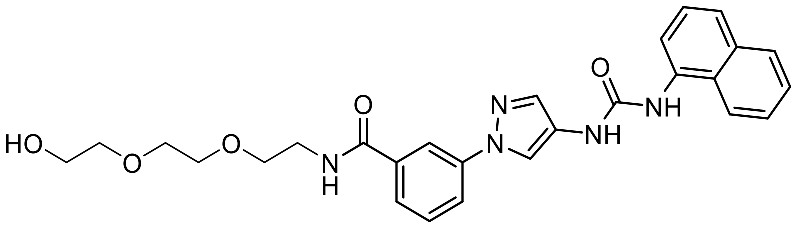

The reaction followed general procedure B1: Amide coupling using EDC HCl + HOBt.

70 mg of (**20**) (0.188 mmol; 1 eq.), 67 mg HOBt monohydrate (containing 14 wt.% water) (0.376 mmol; 2 eq.) and 72 mg EDC HCl (0.376 mmol; 2 eq.) were placed in a flask and suspended in 10 mL of dry THF. 131 µL of DIPEA (0.752 mmol; 4 eq.) were added and the reaction stirred for 5 min while it was cooled via ice-water bath. 52 µL of 2-(2-(2-aminoethoxy)ethoxy)ethan-1-ol (0.376 mmol; 2 eq.) were added and the cooling was removed shortly after. HPLC monitoring showed full conversion after 20 h. The reaction was quenched with demin. H_2O and 10% HCl (aq.) solution. The mixture was extracted 3x with EtOAc. The organic phase was washed 2x with 1 M NaOH (aq.) solution and 1x with brine. After drying the organic phase over Na_2SO_4, filtering and evaporating the organic solvent, the crude product was purified via flash chromatography (MeOH/DCM: 2/98 – 10/90) and dried in high vacuum to yield compound (**SI-VI**) as a white solid (72 mg; 76%). ^{1}H NMR (400 MHz, DMSO) δ 8.99 (s, 1H), 8.87 (s, 1H), 8.75 (t, J = 5.5 Hz, 1H), 8.60 (s, 1H), 8.27 – 8.23 (m, 1H), 8.12 (d, J = 8.4 Hz, 1H), 8.06 – 8.02 (m, 1H), 7.97 (dd, J = 8.1, 1.3 Hz, 1H), 7.95 – 7.92 (m, 1H), 7.86 (s, 1H), 7.76 (d, J = 7.8 Hz, 1H), 7.65 (d, J = 8.2 Hz, 1H), 7.62 – 7.53 (m, 3H), 7.48 (t, J = 7.9 Hz, 1H), 4.57 (t, J = 5.5 Hz, 1H), 3.58 – 3.51 (m, 6H), 3.43 (ddd, J = 10.6, 8.4, 3.4 Hz, 6H). ^{13}C NMR (101 MHz, DMSO) δ 165.5, 152.8, 139.8, 135.7, 134.4, 133.7, 133.1, 129.6, 128.4, 126.0, 125.9, 125.9, 125.7, 124.7, 124.5, 123.0, 121.4, 120.3, 117.3, 116.4, 116.2, 72.4, 69.7, 69.7, 68.9, 60.2.(one aliphatic signal below DMSO-peak); DEPT: ^{13}C NMR (101 MHz, DMSO) δ 133.1, 129.6, 128.4, 125.9, 125.9, 125.7, 124.6, 123.0, 121.4, 120.3, 117.3, 116.4, 116.2, 72.4, 69.7, 69.7, 68.9, 60.2, 39.3; FTIR [cm^{-1}]: 3274, 2922, 2861, 1638, 1584, 1543, 1487, 1388, 1344, 1216; TLC-MS(ESI) *m/z*: 526.3 [M+Na]$^+$; 502.3 [M-H]$^-$; 538.2 [M+Cl]$^-$; HRMS(ESI) *m/z*: calcd. for [M+H]$^+$: 504.22407; found: 504.2245; HPLC t_{ret}: 6.69 min.

10.13 Scaffold synthesis Table 4:

Scheme S4: Synthesis Table 4

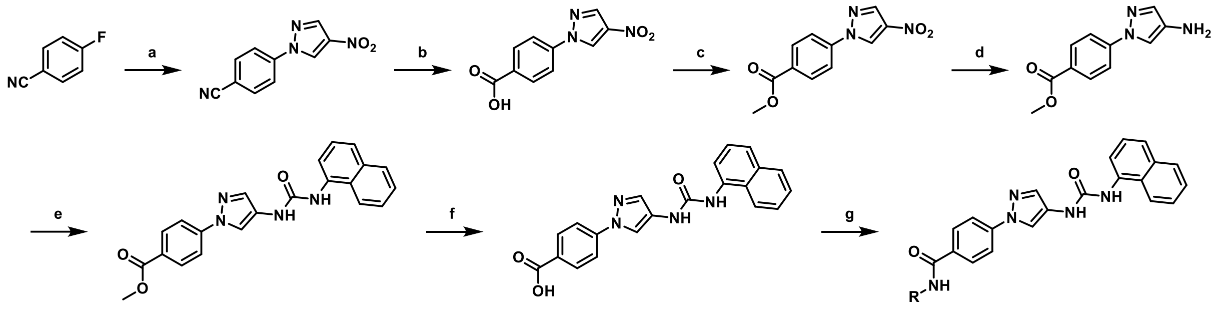

Reaction conditions and reagents exemplified by the synthesis of **28a**: **a)** 4-fluorobenzonitrile (1 eq.), 4-nitro-1*H*-pyrazole (1.1 eq.), K_2CO_3 (2.5 eq.), DMSO, 120 °C (quant.); **b) 22** (1 eq.), HCl (aq.), H_2O, 100 °C (69%); **c) 23** (1 eq.), H_2SO_4 (conc.) (cat.), MeOH, 65 °C (79%); **d) 24** (1 eq.), Fe(0) (10 eq.), NH_4Cl (10 eq.), EtOH:H2O (4:1), 60 °C (89%); **e) 25** (1 eq.), 1-isocyanatonaphthalene (1 eq.), DCM (dry), RT (92%); **f) 26** (1 eq.), KOH (6 eq.), MeOH:THF:H2O (10:10:1), 60 °C (89%); **g) 27** (1 eq.), HATU (1.5 eq.), DIPEA (3 eq.), cyclo-propylamine (2 eq.) DMF (dry), RT (95%).

4-(4-nitro-1*H*-pyrazol-1-yl)benzonitrile (**22**)

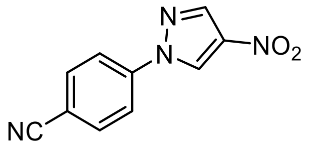

1015 mg of 4-fluorobenzonitrile (8.381 mmol; 1 eq.) as well as 1042 mg of 4-nitro-1*H*-pyrazole (9.219 mmol; 1.1 eq.) and 2896 mg of K_2CO_3 (20.952 mmol; 2.5 eq.) was placed in a flask and were dissolved in 25 mL of DMSO. The reaction was heated to 120 °C for 4 h. The reaction was quenched with demin. H_2O and a yellow solid precipitated. The solids were filtered off and washed thoroughly with demin. H_2O. The product was dried to yield (**22**) (Purity: 94.9% at 254 nm; 94.3% at 230 nm) as a yellow solid (quantitative). ^1H NMR (400 MHz, DMSO) δ 9.79 (s, 1H), 8.63 (s, 1H), 8.20 – 8.14 (m, 2H), 8.08 – 8.04 (m, 2H); ^{13}C NMR (101 MHz, DMSO) δ 141.4, 137.8, 137.4, 134.1, 129.0, 120.0, 118.2, 110.6.

4-(4-nitro-1*H*-pyrazol-1-yl)benzoic acid (**23**)

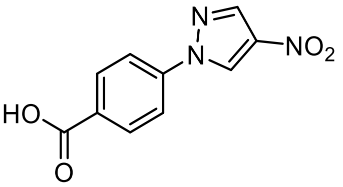

1500 mg of (**22**) (7.003 mmol; 1 eq.) were put in a flask and suspended in 10 mL of demin. H_2O and 20 mL of 37% HCl (aq.). The reaction was heated to reflux for 3 h and then for 19 h to 85-90 °C. On the next day 20 mL of additional 37% HCl (aq.) were added and the reaction was again heated to reflux for 3 h and 19 h to 85-90 °C. After cooling the suspension was filtered and dried to yield (**23**) as a grey white solid (1122 mg; 69%). ^1H NMR (400 MHz, DMSO) δ 13.22 (s, 1H), 9.77 (s, 1H), 8.60 (s, 1H), 8.12 – 8.06 (m, 4H); ^{13}C NMR (101 MHz, DMSO) δ 166.4, 141.3, 137.5, 137.2, 130.9, 130.2, 128.7, 119.3.

methyl 4-(4-nitro-1*H*-pyrazol-1-yl)benzoate (**24**)

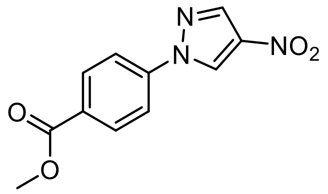

1000 mg of (**23**) (4.289 mmol; 1 eq.) were suspended in 25 mL of MeOH. 0.1mL of H_2SO_4 were added to the reaction, which was then heated to reflux for 24 h. After cooling the precipitated product was filtered of and washed carefully with a tiny amount of cold MeOH, to yield the pure product (**24**) as a yellow-white solid (840 mg; 79%). ^{1}H NMR (400 MHz, DMSO) δ 9.80 – 9.75 (m, 1H), 8.60 (s, 1H), 8.14 – 8.09 (m, 4H), 3.88 (s, 3H); ^{13}C NMR (101 MHz, DMSO) δ 165.3, 141.6, 137.5, 137.3, 130.8, 128.9, 128.7, 119.3, 52.4.

methyl 4-(4-amino-1*H*-pyrazol-1-yl)benzoate (**25**)

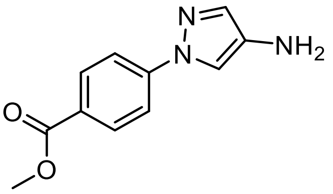

The reaction followed general procedure C: Reduction of *N*-arylated-4-nitropyrazoles.

800 mg of (**24**) (3.236 mmol; 1 eq.) were combined with 1731 mg of Fe powder (32.361 mmol; 10 eq.) and 1807 mg of NH_4Cl (32.361 mmol; 10 eq.). The solids were then suspended in 25 mL of a mixture of EtOH:H_2O (4:1). The reaction was heated to 60 °C for 2 h. After cooling to RT the reaction was filtered through celite. The alcoholic components were evaporated and the remaining suspension was diluted with EtOAc and subsequently washed 3x with 0.5 M NaOH (aq.). The aquatic layer was 1x re-extracted with EtOAc. The combined organic layers were dried over Na_2SO_4, filtered and evaporated to dryness to yield (**25**) as a yellowish solid (625 mg; 89%). ^{1}H NMR (400 MHz, DMSO) δ 8.01 – 7.97 (m, 2H), 7.83 – 7.79 (m, 2H), 7.77 (s, 1H), 7.36 (s, 1H), 4.33 (bs, 2H), 3.84 (s, 3H); ^{13}C NMR (101 MHz, DMSO) δ 165.7, 143.3, 134.4, 134.3, 130.7, 125.2, 116.5, 111.7, 52.0.

methyl 4-(4-(3-(naphthalen-1-yl)ureido)-1*H*-pyrazol-1-yl)benzoate (**26**)

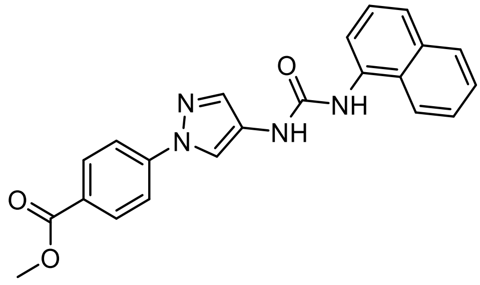

The reaction followed general procedure D: Urea-formation with isocyanates.

600 mg of (**25**) (2.762 mmol; 1 eq.) were dissolved in 25 mL of dry DCM. While stirring and cooling via ice-water bath, 0.4 mL of 1-isocyanatonaphthalene (2.762 mmol; 1 eq.) were added to the solution. The reaction was stirred at RT for 19 h. The precipitated product was filtered to yield the pure compound (**26**) as a white solid (980 mg; 92%). ^{1}H NMR (400 MHz, DMSO) δ 9.02 (s, 1H), 8.87 (s, 1H), 8.60 (s, 1H), 8.12 (d, J = 8.4 Hz, 1H), 8.08 – 8.02 (m, 3H), 7.99 – 7.92 (m, 4H), 7.65 (d, J = 8.2 Hz, 1H), 7.62 – 7.52 (m, 2H), 7.48 (t, J = 7.9 Hz, 1H), 3.86 (s,

3H); ^{13}C NMR (101 MHz, DMSO) δ 165.6, 152.8, 143.0, 134.4, 134.2, 133.7, 130.8, 128.4, 126.3, 126.0, 125.9, 125.9, 125.7, 125.3, 123.0, 121.4, 117.4, 117.3, 116.3, 52.1.

4-(4-(3-(naphthalen-1-yl)ureido)-1*H*-pyrazol-1-yl)benzoic acid (**27**)

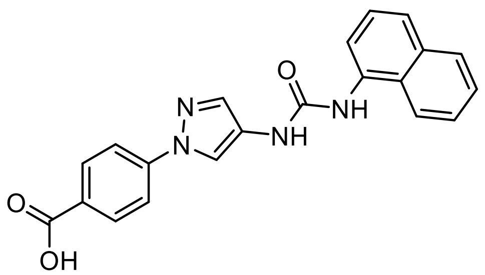

430 mg of (**26**) (1.113 mmol; 1 eq.) as well as 375 mg of KOH (6.677 mmol; 6 eq.) were added to a flask and suspended in a mixture of 12.5 mL of MeOH, 12.5 mL of THF and 1.25 mL of H_2O. The reaction was heated to 60 °C for 3d. After full conversion the organic solvent was evaporated and 10% HCl (aq.) was added. The reaction was left to stir for another 30 min. The precipitate was filtered, washed carefully with demin. H_2O and dried to yield (**27**) as a cream-colored sold (368 mg; 89%). ^{1}H NMR (400 MHz, DMSO) δ 10.73 (s, 1H), 10.16 (s, 1H), 8.52 (s, 1H), 8.40 (d, J = 7.8 Hz, 1H), 8.10 (d, J = 8.7 Hz, 2H), 8.07 – 8.02 (m, 1H), 7.95 – 7.90 (m, 2H), 7.82 (d, J = 8.7 Hz, 2H), 7.63 (d, J = 8.2 Hz, 1H), 7.59 – 7.52 (m, 2H), 7.49 (t, J = 7.9 Hz, 1H); ^{13}C NMR (101 MHz, DMSO) δ 169.3, 153.5, 141.0, 135.3, 134.9, 133.8, 133.2, 130.6, 128.2, 126.4, 125.8, 125.8, 125.6, 125.4, 122.5, 122.3, 117.5, 116.6, 115.8.

10.14 Compound synthesis Table 4:

N-cyclopropyl-4-(4-(3-(naphthalen-1-yl)ureido)-1*H*-pyrazol-1-yl)benzamide (**28a**)

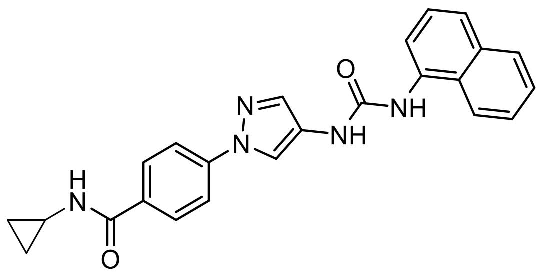

The reaction followed general procedure B4: Amide coupling using HATU.

65 mg of (**27**) (0.175 mmol; 1 eq.) as well as 100 mg of HATU (0,262 mmol; 1.5 eq.) were dissolved in 8 mL of dry DMF. 89 µL of DIPEA (0.524 mmol; 3 eq.) were added and the reaction stirred for 18 h at RT. Subsequently 24 µL of cyclopropylamine (0.349 mmol; 2 eq.) were added and the reaction stirred at RT for another 24 h. The resulting mixture was quenched with demin. H_2O and precipitating product was filtered off and dried to yield compound (**28a**) as an off-white solid (68 mg; 95%). ^{1}H NMR (400 MHz, DMSO) δ 8.99 (s, 1H), 8.87 (s, 1H), 8.57 (s, 1H), 8.47 (d, J = 3.9 Hz, 1H), 8.12 (d, J = 8.3 Hz, 1H), 8.03 (d, J = 7.5 Hz, 1H), 7.97 – 7.85 (m, 6H), 7.65 (d, J = 8.1 Hz, 1H), 7.62 – 7.53 (m, 2H), 7.48 (t, J = 7.9 Hz, 1H), 2.91 – 2.81 (m, 1H), 0.75 – 0.66 (m, 2H), 0.62 – 0.52 (m, 2H); ^{13}C NMR (101 MHz, DMSO) δ 166.6, 152.8, 141.5, 134.4, 133.7, 133.6, 131.2, 128.7, 128.4, 126.0, 125.9, 125.9, 125.7, 125.0, 123.0, 121.4, 117.4, 117.0, 116.3, 23.1, 5.7; FTIR [cm^{-1}]: 3265, 3094, 3011, 1636, 1610, 1591, 1540, 1508, 1500, 1457; TLC-MS(ESI) *m/z*: 433.9 [M+Na]$^{+}$; 410.1 [M-H]$^{-}$; 446.0 [M+Cl]$^{-}$; HRMS(ESI) *m/z*: calcd. for [M+H]$^{+}$: 412.17672; found: 412.1770; HPLC t_{ret}: 7.23 min.

N-methyl-4-(4-(3-(naphthalen-1-yl)ureido)-1*H*-pyrazol-1-yl)benzamide (**28b**)

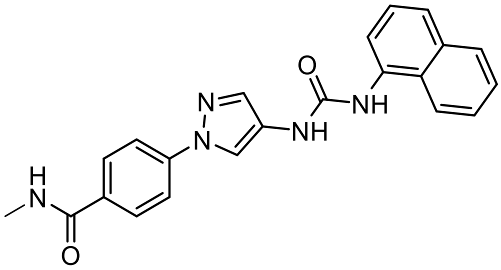

The reaction followed general procedure B4: Amide coupling using HATU.

65 mg of (**27**) (0.175 mmol; 1 eq.) as well as 100 mg of HATU (0,262 mmol; 1.5 eq.) were dissolved in 8 mL of dry DMF. 89 µL of DIPEA (0.524 mmol; 3 eq.) were added and the reaction stirred for 18 h at RT. Subsequently 175µL of 2 M methylamine (THF) (0.349 mmol; 2 eq.) were added and the reaction stirred at RT for another 24 h. The resulting mixture was quenched with demin. H_2O and precipitating product was filtered off. The crude solid product was re-dissolved in THF. MeOH was added to the solution and THF was evaporated. Again precipitating product was filtered off and washed thoroughly with MeOH, Et_2O and Pentane to yield compound (**28b**) as a beige solid (26 mg; 39%). ^{1}H NMR (400 MHz, DMSO) δ 8.99 (s, 1H), 8.86 (s, 1H), 8.56 (s, 1H), 8.51 – 8.40 (m, 1H), 8.12 (d, J = 8.1 Hz, 1H), 8.03 (d, J = 7.3 Hz, 1H), 7.99 – 7.84 (m, 6H), 7.65 (d, J = 8.0 Hz, 1H), 7.57 (dt, J = 14.6, 6.8 Hz, 2H), 7.48 (t, J = 7.8 Hz, 1H), 2.80 (d, J = 3.7 Hz, 3H); ^{13}C NMR (101 MHz, DMSO) δ 165.8, 152.8, 141.5, 134.4, 133.7, 133.7, 131.4, 128.6, 128.4, 126.0, 125.9, 125.9, 125.7, 125.0, 123.0, 121.4, 117.3, 117.1, 116.3, 26.3; FTIR [cm^{-1}]: 3261, 3092, 3055, 1636, 1610, 1593, 1554, 1508, 1502, 1405; TLC-MS(ESI) *m/z*: 407.9 [M+Na]$^+$; 384.0 [M-H]$^-$; 420.2 [M+Cl]$^-$; HRMS(ESI) *m/z*: calcd. for [M+H]$^+$: 386.16107; found: 386.1625; HPLC t_{ret}: 6.72 min.

10.15 Scaffold synthesis Table 5:

Scheme S5: Synthesis Table 5

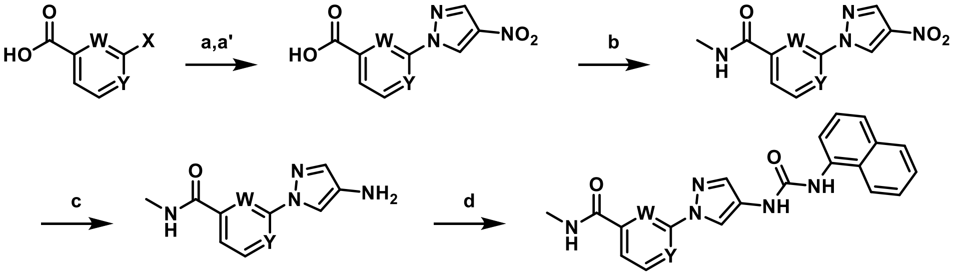

Reaction conditions and reagents exemplified by the synthesis of **35a** for W = CH, Y = N and of **36a** for W = N, Y = CH (only for a')): **a)** 2-bromoisonicotinic acid (1 eq.), 4-nitro-1*H*-pyrazole (1 eq.), K_2CO_3 (3 eq.), Cu(I)I (0.1 eq.), *trans-N,N'*-dimethylcyclohexane-1,2-diamine (0.2 eq.), DMF, 95 °C (95%); **a')** 6-fluoropicolinic acid (1 eq.), 4-nitro-1*H*-pyrazole (1 eq.), methanesulfonic acid (5 eq.), Dioxane, 101 °C (66%); **b) 29** (1 eq.), CDI (1.1 eq.), 40% methylamine (aq.) (2.5 eq.), THF (dry), RT (79%); **c) 31** (1 eq.), Fe(0) (5 eq.), NH_4Cl (5 eq.), EtOH:H_2O (4:1), 60 °C (65%); **d) 33** (1 eq.), 1-isocyanatonaphthalene (1 eq.), THF (dry), RT (70%).

2-(4-nitro-1*H*-pyrazol-1-yl)isonicotinic acid (**29**)

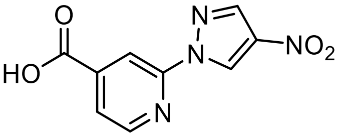

The reaction followed general procedure A: Ullmann-type reaction with pyrazoles.

1500 mg 4-nitro-1*H*-pyrazole (13.265 mmol; 1 eq.), 2680 mg 2-bromoisonicotinic acid (13.265 mmol; 1 eq.) and 5500 mg K_2CO_3 (39.795 mmol; 3 eq.) were suspended in 50 mL of DMF. After degasification of the mixture and after putting the system under argon, 0.418 mL of ligand (2.653 mmol; 0.2 eq.) and 253 mg of Cu(I)I (1.327 mmol; 0.1 eq.) were introduced. The reaction was sealed and heated to 95 °C for 20 h. The reaction was quenched with demin. H_2O and acidified using 10% HCl (aq.) after which the product precipitated. The solids were filtered off and dried in the convection oven to yield (**29**) as a white solid (2950 mg; 95%). The crude product was directly subjected to the follow-up reaction.

6-(4-nitro-1*H*-pyrazol-1-yl)picolinic acid (**30**)

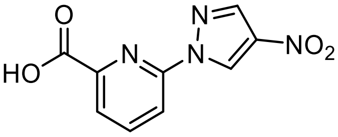

500 mg 4-nitro-1*H*-pyrazole (4.422 mmol; 1 eq.) and 624 mg 6-fluoropicolinic acid (4.422 mmol; 1 eq.) were suspended in 25 mL of Dioxane. 1.436 mL of methanesulfonic acid (22.108 mmol; 5 eq.) was slowly added to the solution and the reaction was heated to reflux for 20 h. The reaction was poured onto ice. The forming precipitate was filtered off and dried to yield (**30**) as a beige-white solid (680 mg; 66%). ^1H NMR (400 MHz, DMSO) δ 13.31 (br s, 1H), 9.82 (s, 1H), 8.64 – 8.60 (m, J = 0.5 Hz, 1H), 8.30 – 8.25 (m, 1H), 8.21 (dd, J = 8.2, 0.9

Hz, 1H), 8.13 (dd, J = 7.4, 0.9 Hz, 1H); ^{13}C NMR (101 MHz, DMSO) δ 164.6, 148.9, 146.7, 141.9, 137.9, 137.2, 127.6, 124.2, 116.3.

N-methyl-2-(4-nitro-1*H*-pyrazol-1-yl)isonicotinamide (**31**)

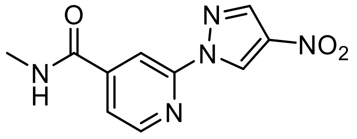

The reaction followed general procedure B2: Amide coupling using CDI.

To 500 mg of (**29**) (2.135 mmol; 1 eq.) dissolved in 12.5 mL of dry THF 381 mg of CDI (2.349 mmo; 1.1 eq.) dissolved in 2x 7.5 mL of dry THF was added. The reaction was stirred at RT for 1.5 h after which monitoring showed quantitative activation of the carboxylic acid component. Subsequently 370 µL of 40% methylamine (aq.) solution (4.270 mmol; 2.5 eq.) were added and the reaction was stirred for 2 h. The reaction was quenched with demin. H_2O and 10% HCl (aq.) solution. The mixture was extracted 2x with EtOAc. The organic phase was washed 1x with brine, was dried over Na_2SO_4, filtered and evaporated to yield (**31**) is a white solid (415 mg; 79%). ^{1}H NMR (400 MHz, DMSO) δ 9.45 (s, 1H), 8.97 (d, J = 4.0 Hz, 1H), 8.71 – 8.64 (m, 2H), 8.35 (s, 1H), 7.86 (dd, J = 5.0, 1.2 Hz, 1H), 2.83 (d, J = 4.5 Hz, 3H); ^{13}C NMR (101 MHz, DMSO) δ 163.7, 150.1, 149.6, 145.1, 137.8, 137.1, 126.9, 121.3, 110.6, 26.4; TLC-MS(ESI) *m/z*: 246.2 $[M-H]^-$.

N-methyl-6-(4-nitro-1*H*-pyrazol-1-yl)picolinamide (**32**)

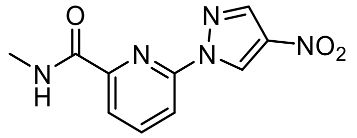

The reaction followed general procedure B2: Amide coupling using CDI.

To 500 mg of (**30**) (2.135 mmol; 1 eq.) dissolved in 12.5 mL of dry THF 433 mg of CDI (2.669 mmol; 1.25 eq.) dissolved in 12.5 mL of dry THF was added. The reaction was stirred at 50 °C for 1.5 h after which monitoring showed quantitative activation of the carboxylic acid component. Subsequently 555 µL of 40% methylamine (aq.) solution (6.406 mmol; 3 eq.) were added and the reaction was stirred for 22 h. The precipitating product was filtered of and washed with Et_2O to yield (**32**) is a white solid (283 mg; 54%). ^{1}H NMR (400 MHz, DMSO) δ 10.25 (s, 1H), 9.23 (d, J = 4.3 Hz, 1H), 8.62 (s, 1H), 8.24 (t, J = 7.8 Hz, 1H), 8.15 (d, J = 8.0 Hz, 1H), 8.07 (d, J = 7.4 Hz, 1H), 2.90 (d, J = 4.7 Hz, 3H); ^{13}C NMR (101 MHz, DMSO) δ 162.9, 148.9, 148.3, 141.7, 137.9, 137.3, 128.1, 121.3, 114.8, 25.9.

2-(4-amino-1*H*-pyrazol-1-yl)-*N*-methylisonicotinamide (**33**)

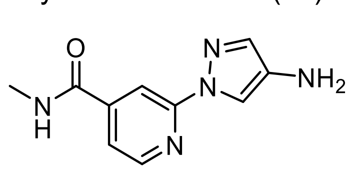

The reaction followed general procedure C: Reduction of *N*-arylated-4-nitropyrazoles.

350 mg of (**31**) (1.416 mmol; 1 eq.) were combined with 395 mg of Fe powder (7.079 mmol; 5 eq.) and 379 mg of NH_4Cl (7.079 mmol; 5 eq.). The solids were then suspended in 20 mL of a mixture of EtOH:H_2O (4:1). The reaction was heated to 60 °C for 1.5 h. After cooling to RT the reaction was filtered through celite. The alcoholic components were evaporated and the remaining suspension was diluted with EtOAc and subsequently washed 2x with 1 M NaOH

(aq.) solution and 1x with brine. The combined organic layers were dried over Na_2SO_4, filtered and evaporated to dryness to yield (**33**) as a brown-red solid (200 mg; 65%). ^1H NMR (400 MHz, DMSO) δ 8.83 (s, 1H), 8.48 (d, J = 4.7 Hz, 1H), 8.16 (s, 1H), 7.87 (s, 1H), 7.53 (d, J = 4.2 Hz, 1H), 7.39 (s, 1H), 4.36 (s, 2H), 2.81 (d, J = 3.8 Hz, 3H); ^{13}C NMR (101 MHz, DMSO) δ 164.5, 151.8, 148.8, 144.2, 134.8, 134.0, 117.6, 110.9, 108.5, 26.3; TLC-MS(ESI) *m/z*: 240.4 $[M+Na]^+$; 272.5 $[M+Na+MeOH]^+$; 216.3 $[M-H]^-$.

6-(4-amino-1*H*-pyrazol-1-yl)-*N*-methylpicolinamide (**34**)

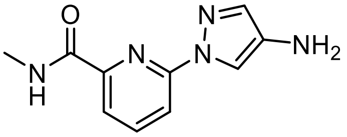

The reaction followed general procedure C: Reduction of *N*-arylated-4-nitropyrazoles.

250 mg of (**32**) (1.011 mmol; 1 eq.) were combined with 282 mg of Fe powder (5.056 mmol; 5 eq.) and 270 mg of NH_4Cl (5.056 mmol; 5 eq.). The solids were then suspended in 20 mL of a mixture of EtOH:H_2O (4:1). The reaction was heated to 60 °C for 1.5 h. After cooling to RT the reaction was filtered through celite. The alcoholic components were evaporated and the remaining suspension was diluted with EtOAc and subsequently washed 2x with 1 M NaOH (aq.) solution and 1x with brine. The combined organic layers were dried over Na_2SO_4, filtered and evaporated to dryness to yield (**34**) (Purity: 97.1% at 254 nm; 93.1% at 230 nm) as a yellow-brown solid (165 mg; 75%). ^1H NMR (400 MHz, DMSO) δ 8.90 (d, J = 3.5 Hz, 1H), 8.38 (s, 1H), 8.01 (t, J = 7.7 Hz, 1H), 7.93 (d, J = 8.1 Hz, 1H), 7.79 (d, J = 7.2 Hz, 1H), 7.39 (s, 1H), 4.30 (s, 2H), 2.86 (d, J = 4.6 Hz, 3H); ^{13}C NMR (101 MHz, DMSO) δ 163.7, 150.1, 148.5, 140.1, 135.2, 133.7, 117.9, 113.2, 112.2, 26.0; TLC-MS(ESI) *m/z*: 239.9 $[M+Na]^+$; 216.0 $[M-H]^-$

10.16 Compound synthesis Table 5:

N-methyl-2-(4-(3-(naphthalen-1-yl)ureido)-1*H*-pyrazol-1-yl)isonicotinamide (**35a**)

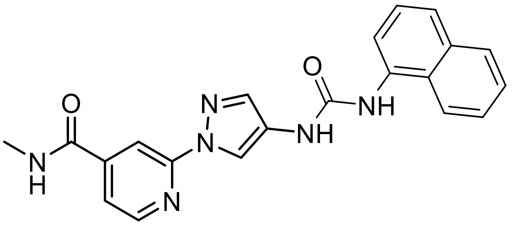

The reaction followed general procedure D: Urea-formation with isocyanates.

50 mg of (**33**) (0.230 mmol; 1 eq.) were dissolved in 10 mL of dry THF. 33 µL of 1-isocyanatonaphthalene (0.230 mmol; 1 eq.) were added slowly via pipette. The reaction stirred for 72 h at RT, while product precipitated. The solids were filtered off to yield (**35a**) as a white solid (62 mg; 70%). ^1H NMR (400 MHz, DMSO) δ 9.04 (s, 1H), 8.96 – 8.85 (m, 2H), 8.73 (s, 1H), 8.58 (d, J = 4.7 Hz, 1H), 8.28 (s, 1H), 8.11 (d, J = 8.1 Hz, 1H), 8.00 (d, J = 7.4 Hz, 1H), 7.97 – 7.90 (m, 2H), 7.70 – 7.63 (m, 2H), 7.57 (dt, J = 14.7, 6.8 Hz, 2H), 7.49 (t, J = 7.8 Hz, 1H), 2.83 (d, J = 3.7 Hz, 3H); ^{13}C NMR (101 MHz, DMSO) δ 164.4, 152.8, 151.6, 149.0, 144.5, 134.5, 134.4, 133.7, 128.4, 126.2, 125.9, 125.9, 125.7, 125.0, 123.2, 121.5, 118.7, 117.7, 115.3, 109.0, 26.4; FTIR [cm^{-1}]: 3307, 3247, 3049, 2932, 2871, 1642, 1609, 1592, 1550, 1456; TLC-MS(ESI) *m/z*: 408.9 $[M+Na]^+$; 440.8 $[M+Na+MeOH]^+$; 385.0 $[M-H]^-$; 420.9 $[M+Cl]^-$; HRMS(ESI) *m/z*: calcd. for $[M+H]^+$: 387.15632; found: 387.1563; HPLC t_{ret}: 6.77 min.

N-methyl-6-(4-(3-(naphthalen-1-yl)ureido)-1*H*-pyrazol-1-yl)picolinamide (**36a**)

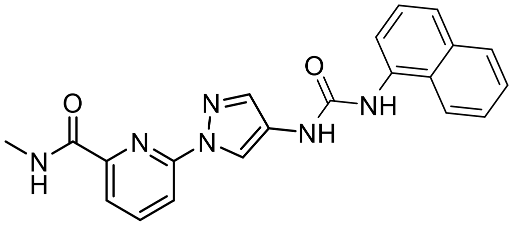

The reaction followed general procedure D: Urea-formation with isocyanates.

35 mg of (**34**) (0.161 mmol; 1 eq.) were dissolved in 10 mL of dry THF. 23 µL of 1-isocyanatonaphthalene (0.161 mmol; 1 eq.) were added slowly via pipette. The reaction stirred for 24 h at RT, while product precipitated. The solids were filtered off to yield (**36a**) as a white solid (16 mg; 26%). ^{1}H NMR (400 MHz, DMSO) δ 9.09 (s, 1H), 9.03 – 8.93 (m, 2H), 8.89 (s, 1H), 8.15 – 8.02 (m, 4H), 7.97 – 7.92 (m, 2H), 7.90 (d, J = 7.0 Hz, 1H), 7.65 (d, J = 8.1 Hz, 1H), 7.62 – 7.52 (m, 2H), 7.49 (t, J = 7.9 Hz, 1H), 2.88 (d, J = 4.5 Hz, 3H); ^{13}C NMR (101 MHz, DMSO) δ 163.7, 152.9, 150.0, 148.9, 140.6, 135.3, 134.4, 133.7, 128.4, 126.0, 125.9, 125.9, 125.7, 124.7, 123.1, 121.4, 119.0, 117.5, 117.1, 113.9, 26.0; FTIR [cm^{-1}]: 3273, 3109, 3051, 1634, 1600, 1579, 1465, 1381, 1267, 1218; TLC-MS(ESI) *m/z*: 408.8 [M+Na]$^{+}$; 384.9 [M-H]$^{-}$; 420.9 [M+Cl]$^{-}$; HRMS(ESI) *m/z*: calcd. for [M+H]$^{+}$: 387.15632; found: 387.1547; HPLC t_{ret}: 7.47 min.

.

10.17 Scaffold synthesis Table 6:

Scheme S6: Synthesis Table 6

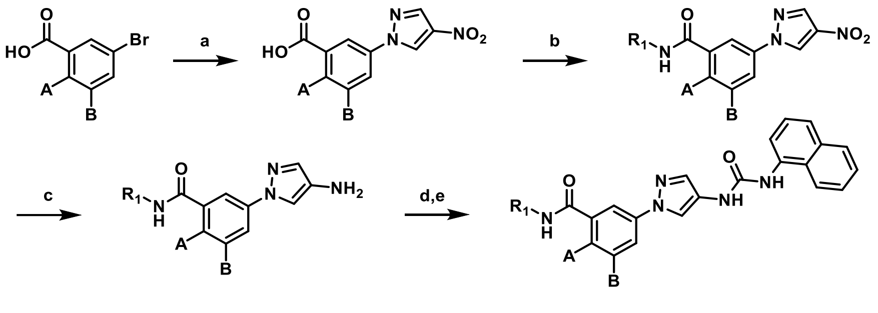

Reaction conditions and reagents exemplified by the synthesis of **45b** (A = H; B = CH_3): **a)** 4-nitro-1*H*-pyrazole (1 eq.), 5-bromo-3-methylbenzoic acid (1.2 eq.), *trans*-*N*,*N'*-dimethylcyclohexane-1,2-diamine (0.3 eq.), Cu(I)I (0.3 eq.), DMF, 100 °C (60%), **b) 40** (1 eq.), **5** (1 eq.), EDC HCl (2 eq.), HOBt monohydrate (containing 20 wt.% water) (2 eq.), DIPEA (3 eq.), DCM (dry), 0 °C - RT (69%); **c) 41** (1 eq.), Fe(0) (10 eq.), NH_4Cl (10 eq.), EtOH:H_2O (2:1), 65 °C (quant.); **d) 42** (1 eq.), 1-isocyanatonaphthalene (1 eq.), DCM (dry), RT (directly used in e)); **e)** product of d), 1.25 M HCl (EtOH) (7.5 eq.), EtOH, 60 °C (74%, over two steps, **HCl salt**).

2-methyl-5-(4-nitro-1*H*-pyrazol-1-yl)benzoic acid (**37**)

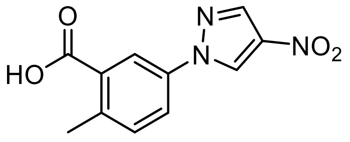

The reaction followed general procedure A: Ullmann-type reaction with pyrazoles.

700 mg 4-nitro-1*H*-pyrazole (6.19 mmol; 1 eq.), 1704 mg 5-bromo-2-methylbenzoic acid (7.92 mmol; 1.28 eq.) and 6051 mg Cs_2CO_3 (18.57 mmol; 3 eq.) were suspended in 40 mL of dry DMF. After degasification of the mixture and after putting the system under argon, 0.293 mL of ligand (1.86 mmol; 0.3 eq.) and 354 mg of Cu(I)I (1.86 mmol; 0.3 eq.) were introduced. The reaction was sealed and heated to 100 °C for 18 h. The reaction was quenched with demin. H_2O and acidified using 10% HCl (aq.). The solution was extracted 3x with EtOAc. The organic phase was washed 2x with Brine and then dried over Na_2SO_4, filtered and evaporated. The obtained solid was then recrystallized in MeOH. In remaining filtrate a second fraction of product precipitated. (**37**) was obtained as an off-white solid (1062 mg; 69%). ^{1}H NMR (400 MHz, DMSO) δ 13.22 (br s, 1H), 9.69 (s, 1H), 8.55 (s, 1H), 8.34 (d, J = 2.4 Hz, 1H), 8.02 (dd, J = 8.3, 2.5 Hz, 1H), 7.50 (d, J = 8.4 Hz, 1H), 2.56 (s, 3H); ^{13}C NMR (101 MHz, DMSO) δ 167.7, 139.1, 137.0, 136.9, 136.3, 132.9, 131.7, 128.2, 122.2, 121.0, 20.7; TLC-MS(ESI) *m/z*: 246.0 [M-H]$^-$.

tert-butyl 4-(4-(2-methyl-5-(4-nitro-1*H*-pyrazol-1-yl)benzamido)-1*H*-pyrazol-1-yl)piperidine-1-carboxylate (**38**)

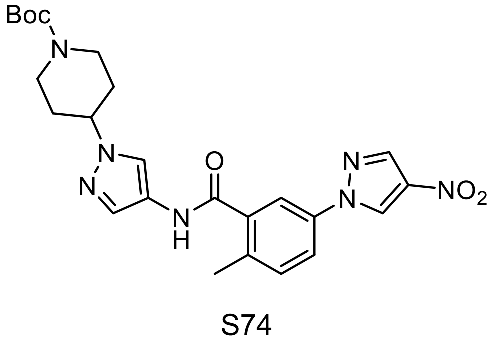

The reaction followed general procedure B1: Amide coupling using EDC HCl + HOBt.

300 mg of (**37**) (1.214 mmol; 1 eq.) as well as 465 mg of EDC HCl (2.427 mmol 2 eq.) and 432 mg of HOBt monohydrate (2.427 mmol; 2 eq.) (containing 14 wt.% water) were put into a flask and suspended in 20 mL of dry DCM, while the mixture was cooled via ice bath. 0.634 mL of DIPEA (3.641 mmol; 3 eq.) were added and the mixture was allowed to stir for 5 min. Afterwards 323 mg of amine (**5**) (1.214 mmol; 1 eq.) were added and the cooling was removed. The reaction stirred for 22 h at RT. The mixture was quenched with demin. water and 2 M NaOH (aq.) solution and then extracted 3 consecutive times with EtOAc. The combined organic layers were dried over Na_2SO_4, filtered and evaporated to dryness. The crude product was then purified using flash chromatography (MeOH/DCM: 2/98 – 10/90) to yield (**38**) as pinkish-white solid (58 mg; 10%). ^1H NMR (400 MHz, DMSO) δ 10.49 (s, 1H), 9.68 (s, 1H), 8.58 (s, 1H), 8.12 – 8.01 (m, J = 5.0 Hz, 2H), 7.96 (d, J = 8.1 Hz, 1H), 7.56 (s, 1H), 7.49 (d, J = 8.3 Hz, 1H), 4.36 (t, J = 11.2 Hz, 1H), 4.04 (d, J = 11.1 Hz, 2H), 2.89 (br s, 2H), 2.42 (s, 3H), 2.04 – 1.94 (m, J = 11.0 Hz, 2H), 1.84 – 1.71 (m, J = 20.1, 11.6 Hz, 2H), 1.42 (s, 9H); ^{13}C NMR (101 MHz, DMSO) δ 164.7, 153.9, 137.4, 137.0, 136.9, 136.0, 136.0, 132.0, 130.0, 128.1, 121.2, 120.3, 119.0, 118.3, 78.8, 58.1, 42.5, 31.9, 28.1, 19.0; TLC-MS(ESI) *m/z*: 518.5 $[M+Na]^+$; 494.4 $[M-H]^-$; 530.5 $[M+Cl]^-$.

tert-butyl 4-(4-(5-(4-amino-1*H*-pyrazol-1-yl)-2-methylbenzamido)-1*H*-pyrazol-1-yl)piperidine-1-carboxylate (**39**)

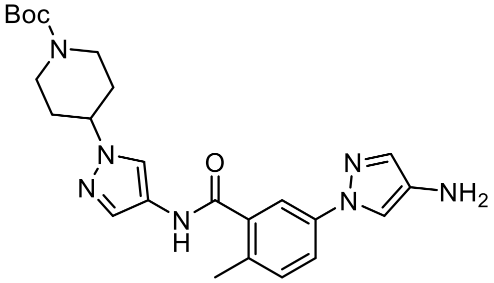

The reaction followed general procedure C: Reduction of *N*-arylated-4-nitropyrazoles.

140 mg of (**38**) (0.283 mmol; 1 eq.) were combined with 151 mg of Fe powder (2.825 mmol; 10 eq.) and 158 mg of NH_4Cl (2.825 mmol; 10 eq.). The solids were then suspended in 20 mL of a mixture of EtOH:H_2O (3:1). The reaction was heated to 60 °C for 90 min after which monitoring via HPLC and TLC showed full conversion. After cooling to RT the reaction was filtered through celite. The alcoholic components were evaporated and the remaining suspension was diluted with EtOAc and subsequently washed 2x with 2M NaOH (aq.). The aquatic layer was 1x re-extracted with EtOAc. The combined organic layers were dried over Na_2SO_4, filtered and evaporated to dryness and then purified using flash chromatography (MeOH/DCM: 2/98 – 10/90) to yield (**39**) (Purity: 96.0% at 254 nm; 93.8% at 230 nm) as a red solid (114 mg; 87%). ^1H NMR (400 MHz, DMSO) δ 10.43 (s, 1H), 8.05 (s, 1H), 7.73 (s, 2H), 7.67 (dd, J = 8.3, 2.4 Hz, 1H), 7.54 (s, 1H), 7.32 (d, J = 8.4 Hz, 1H), 7.26 (s, 1H), 4.35 (tt, J = 11.4, 3.9 Hz, 1H), 4.20 (br s, 2H), 4.04 (d, J = 12.0 Hz, 2H), 2.89 (br s, 2H), 2.35 (s, 3H), 2.05 – 1.95 (m, 2H), 1.83 – 1.70 (m, J = 12.4, 4.3 Hz, 2H), 1.42 (s, 9H); ^{13}C NMR (101 MHz, DMSO) δ 165.4, 153.9, 137.8, 137.1, 133.6, 132.6, 131.9, 131.6, 129.9, 121.3, 118.9, 117.9, 115.9, 112.2, 78.8, 58.0, 31.9, 28.07, 18.8.

3-methyl-5-(4-nitro-1*H*-pyrazol-1-yl)benzoic acid (**40**)

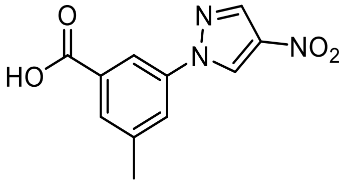

The reaction followed general procedure A: Ullmann-type reaction with pyrazoles.

250 mg 4-nitro-1*H*-pyrazole (2.21 mmol; 1 eq.), 571 mg 5-bromo-3-methylbenzoic acid (2.65 mmol; 1.2 eq.) and 2161 mg Cs_2CO_3 (6.63 mmol; 3 eq.) were suspended in 15 mL of dry DMF. After degasification of the mixture and after putting the system under argon, 0.105 mL of ligand (0.66 mmol; 0.3 eq.) and 126 mg of Cu(I)I (0.66 mmol; 0.3 eq.) were introduced. The reaction was sealed and heated to 100 °C for 16 h. The reaction was quenched with demin. H_2O and acidified using 10% HCl (aq.). The solution was extracted 3x with EtOAc. The organic phase was dried over Na_2SO_4, filtered and evaporated. The obtained solid was then washed with Et_2O to obtain the first clean product fraction. The remaining filtrate was evaporated, reconstituted in a miniscule amount of MeOH and then again filtered to obtain the secund clean product fraction. (**40**) was obtained as an off-white solid (330 mg; 60%). ^{1}H NMR (400 MHz, DMSO) δ 13.27 (br s, 1H), 9.69 (s, 1H), 8.54 (s, 1H), 8.24 (s, 1H), 8.03 (s, 1H), 7.79 (s, 1H), 2.44 (s, 3H); ^{13}C NMR (101 MHz, DMSO) δ 166.4, 140.1, 138.4, 137.0, 136.9, 132.2, 129.4, 128.3, 123.9, 117.4, 20.7; TLC-MS(ESI) *m/z*: 245.9 $[M-H]^-$.

tert-butyl 4-(4-(3-methyl-5-(4-nitro-1*H*-pyrazol-1-yl)benzamido)-1*H*-pyrazol-1-yl)piperidine-1-carboxylate (**41**)

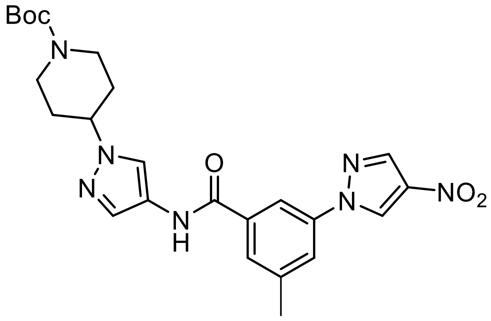

The reaction followed general procedure B1: Amide coupling using EDC HCl + HOBt.

300 mg of (**40**) (1.214 mmol; 1 eq.) as well as 465 mg of EDC HCl (2.427 mmol 2 eq.) and 464 mg of HOBt monohydrate (2.427 mmol; 2 eq.) (containing 20 wt.% water) were put into a flask and suspended in 15 mL of dry DCM, while the mixture was cooled via ice bath. 0.634 mL of DIPEA (3.641 mmol; 3 eq.) were added and the mixture was allowed to stir for 5 min. Afterwards 323 mg of amine (**5**) (1.214 mmol; 1 eq.) were added and the cooling was removed. The reaction stirred for 22 h at RT. The mixture was quenched with demin. water and 2 M NaOH (aq.) solution and then extracted 3 consecutive times with EtOAc. The combined organic layers were dried over Na_2SO_4, filtered and evaporated to dryness. The crude product was then purified using flash chromatography (MeOH/DCM: 2/98 – 10/90) to yield (**41**) (Purity: 97.3% at 254 nm; 94.8% at 230 nm) as an amber-colored solid (413 mg; 69%). ^{1}H NMR (400 MHz, DMSO) δ 10.55 (s, 1H), 9.67 (s, 1H), 8.60 (s, 1H), 8.29 (s, 1H), 8.10 (s, 1H), 8.01 (s, 1H), 7.82 (s, 1H), 7.64 (s, 1H), 4.41 – 4.32 (m, J = 7.3, 3.9 Hz, 1H), 4.04 (d, J = 12.2 Hz, 2H), 2.89 (br s, J = 5.7 Hz, 2H), 2.48 (s, 3H), 1.99 (dd, J = 12.3, 2.3 Hz, 2H), 1.78 (qd, J = 12.4, 4.3 Hz, 2H), 1.42 (s, 9H); ^{13}C NMR (101 MHz, DMSO) δ 162.4, 153.9, 140.0, 138.4, 137.1, 136.9, 135.6, 130.3, 128.3, 127.7, 122.6, 121.3, 119.2, 115.8, 78.8, 58.1, 42.3(signal suppressed), 31.9, 28.1, 21.0; TLC-MS(ESI) *m/z*: 518.1 $[M+Na]^+$; 494.1 $[M-H]^-$; 530.1 $[M+Cl]^-$.

tert-butyl 4-(4-(3-(4-amino-1*H*-pyrazol-1-yl)-5-methylbenzamido)-1*H*-pyrazol-1-yl)piperidine-1-carboxylate (**42**)

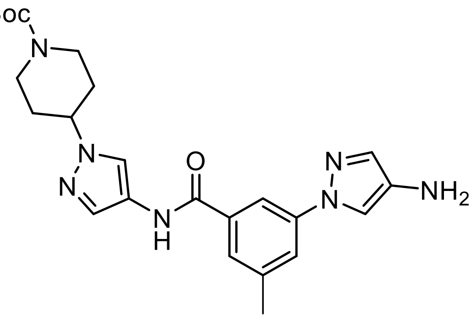

The reaction followed general procedure C: Reduction of *N*-arylated-4-nitropyrazoles.

380 mg of (**41**) (0.767 mmol; 1 eq.) were combined with 429 mg of Fe powder (7.673 mmol; 10 eq.) and 410 mg of NH_4Cl (7.673 mmol; 10 eq.). The solids were then suspended in 45 mL of a mixture of EtOH:H_2O (2:1). The reaction was heated to 65 °C for 45 min after which monitoring via HPLC and TLC showed full conversion. After cooling to RT the reaction was filtered through celite. The alcoholic components were evaporated and the remaining suspension was diluted with 2 M NaOH (aq.) and subsequently extracted 2x with EtOAc. The combined organic layers were dried over Na_2SO_4, filtered and evaporated to dryness to yield (**42**) (Purity: 94.3% at 254 nm; 93.3% at 230 nm) as a red solid (quantitative). ^{1}H NMR (400 MHz, DMSO) δ 10.48 (s, 1H), 8.09 (s, 1H), 8.01 (s, 1H), 7.74 (s, J = 8.6 Hz, 1H), 7.70 (s, 1H), 7.63 (s, 1H), 7.56 (s, 1H), 7.30 (s, 1H), 4.47 – 4.11 (m, 3H)(signal superimposed), 4.04 (d, J = 11.2 Hz, 2H), 2.90 (br s, 2H), 2.42 (s, 3H), 1.99 (d, J = 10.6 Hz, 2H), 1.83 – 1.73 (m, 2H), 1.42 (s, 9H); ^{13}C NMR (101 MHz, DMSO) δ 163.1, 153.9, 140.1, 139.2, 135.3, 133.8, 133.0, 130.2, 124.3, 121.4, 120.2, 119.2, 113.2, 112.2, 78.8, 58.0, 42.4(signal suppressed), 31.9, 28.1, 21.1; TLC-MS(ESI) *m/z*: 488.4 $[M+Na]^+$; 464.4 $[M-H]^-$; 500.3 $[M+Cl]^-$.

N-cyclopropyl-2-methyl-5-(4-nitro-1*H*-pyrazol-1-yl)benzamide (**43**)

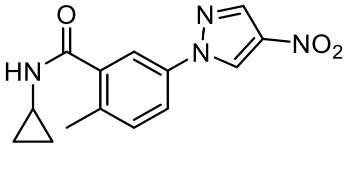

The reaction followed general procedure B4: Amide coupling using HATU.

190 mg of (**37**) (0.769 mmol; 1 eq.) and 584 mg of HATU (1.537 mmol; 2 eq) were dissolved in 16 mL of dry DMF. 0.392 mL of DIPEA (2.306 mmol; 3 eq.) was added to the solution and the reaction was allowed to stir for 1h at RT. After a reaction control showed full activation of the corresponding carboxylic acid, 107 µL of cyclopropylamine (1.537 mmol; 2 eq.) were added and the reaction was left to stir for another 23 h at RT. The reaction was quenched with demin. H_2O. The clean product precipitated and was filtered of to yield (**43**) as a light yellow solid (175 mg; 80%). ^{1}H NMR (400 MHz, DMSO) δ 9.65 (s, 1H), 8.56 (s, 1H), 8.44 (d, J = 3.9 Hz, 1H), 7.91 – 7.86 (m, 2H), 7.42 (d, J = 8.1 Hz, 1H), 2.88 – 2.81 (m, 1H), 2.36 (s, 3H), 0.74 – 0.67 (m, 2H), 0.59 – 0.52 (m, 2H); ^{13}C NMR (101 MHz, DMSO) δ 168.9, 138.0, 136.9, 136.8, 135.9, 135.6, 131.7, 128.0, 119.9, 118.1, 22.7, 18.9, 5.8; TLC-MS(ESI) *m/z*: 285.1 $[M-H]^-$.

5-(4-amino-1*H*-pyrazol-1-yl)-*N*-cyclopropyl-2-methylbenzamide (**44**)

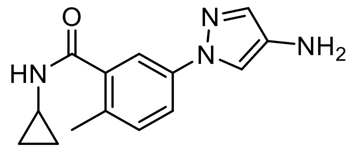

The reaction followed general procedure C: Reduction of *N*-arylated-4-nitropyrazoles.

160 mg of (**43**) (0.559 mmol; 1 eq.) were combined with 156 mg of Fe powder (2.794 mmol; 5 eq.) and 149 mg of NH_4Cl (2.794 mmol; 5 eq.). The solids were then suspended in 25 mL of a mixture of EtOH:H_2O (4:1). The reaction was heated to 60 °C for 5 h. After cooling to RT the reaction was filtered through celite. The alcoholic components were evaporated and the remaining suspension was diluted with EtOAc and subsequently washed 2x with saturated $NaHCO_3$ (aq.) solution. The aquatic layer was 1x re-extracted with EtOAc. The combined organic layers were dried over Na_2SO_4, filtered and evaporated to dryness to yield (**44**) (Purity: 91.5% at 254 nm; 87.8% at 230 nm) as a crude red solid which was directly subjected to the next step.

10.18 Compound synthesis table 6:

2-methyl-5-(4-(3-(naphthalen-1-yl)ureido)-1*H*-pyrazol-1-yl)-*N*-(1-(piperidin-4-yl)-1*H*-pyrazol-4-yl)benzamide (**45a**) (**HCl salt**)

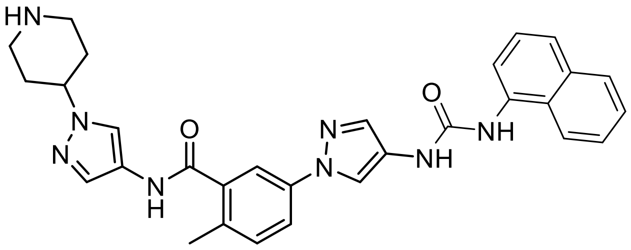

The reaction followed general procedure D(step 1)+E(step 2): Urea-formation with isocyanates + Boc deprotection.

Step 1: 50 mg (**39**) (0.107 mmol; 1 eq.) were dissolved in 10 mL of dry DCM. At RT 17 µL of 1-isocyanatonaphthalene (0.107 mmol; 1 eq.) were then added via pipet. After 24 h the solvent was evaporated and the product was purified via flash chromatography (MeOH/DCM: 1.5/98.5 – 10/90). The intermediate product was then directly used in step 2.

Step 2: The product of step 1 was dissolved in 10 mL of EtOH. 0.516 mL of 1.25 M HCl in EtOH (0.644 mmol; 6 eq.) was added to the mixture and the reaction was heated to 60 °C for 24 h. After reaction monitoring via HPLC showed total conversion, the solvent was evaporated. The product was suspended in Et_2O, filtered off and dried further until completely dry to yield (**45a**) as an orange brown solid (54 mg; 93%; **HCl salt**). ^1H NMR (400 MHz, DMSO) δ 10.54 (s, 1H), 9.48 (s, 1H), 9.17 (s, 1H), 9.01 (d, J = 9.7 Hz, 1H), 8.81 – 8.68 (m, 1H), 8.53 (s, 1H), 8.32 – 8.24 (m, 1H), 8.08 (s, 1H), 8.06 (dd, J = 7.6, 0.9 Hz, 1H), 7.94 – 7.90 (m, 1H), 7.87 (d, J = 2.3 Hz, 1H), 7.85 – 7.79 (m, 2H), 7.64 – 7.59 (m, 2H), 7.59 – 7.51 (m, 2H), 7.46 (t, J = 7.9 Hz, 1H), 7.40 (d, J = 8.5 Hz, 1H), 4.56 – 4.45 (m, 1H), 3.39 (d, J = 12.7 Hz, 2H), 3.12 – 2.99 (m, 2H), 2.40 (s, 3H), 2.22 – 2.11 (m, 4H); FTIR [cm^{-1}]: 3263, 3129, 3043, 2922, 2791, 2469, 1644, 1598, 1540, 1499; TLC-MS(ESI) *m/z*: 535.5 $[M+H]^+$; 533.5 $[M-H]^-$; 569.4 $[M+Cl]^-$; HRMS(ESI) *m/z*: calcd. for $[M+H]^+$: 535.25637; found: 535.2556; HPLC t_{ret}: 5.31 min.

3-methyl-5-(4-(3-(naphthalen-1-yl)ureido)-1*H*-pyrazol-1-yl)-*N*-(1-(piperidin-4-yl)-1*H*-pyrazol-4-yl)benzamide (**45b**) (**HCl salt**)

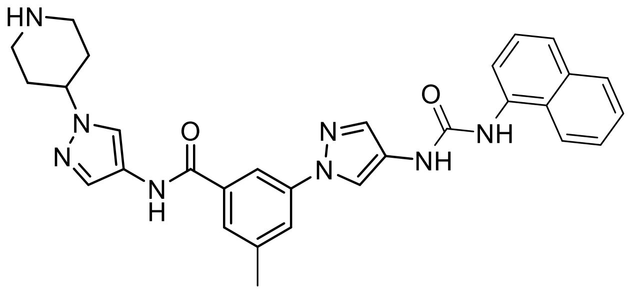

The reaction followed general procedure D(step 1)+E(step 2): Urea-formation with isocyanates + Boc deprotection.

Step 1: 77 mg (**42**) (0.165 mmol; 1 eq.) were dissolved in 10 mL of dry DCM. At RT 24 µL of 1-isocyanatonaphthalene (0.165 mmol; 1 eq.) were then added via pipet. After 24 h the solvent was evaporated and the product was purified via flash chromatography (MeOH/DCM: 4/96 – 10/90). The intermediate product was then directly used in step 2.

Step 2: The product of step 1 was dissolved in 12 mL of EtOH. 1 mL of 1.25 M HCl in EtOH (1.240 mmol; 7.5 eq.) was added to the mixture and the reaction was heated to 60 °C for 22 h. After reaction monitoring via HPLC showed total conversion, the solvent was evaporated. The product was suspended in Et_2O, filtered off and dried further until completely dry to yield (**45b**) as a beige solid (70 mg; 74%; **HCl salt**). ^{1}H NMR (400 MHz, DMSO) δ 10.68 (s, 1H), 9.74 (s, 1H), 9.32 (s, 1H), 9.15 (d, *J* = 10.1 Hz, 1H), 8.91 (q, *J* = 9.9 Hz, 1H), 8.59 (s, 1H), 8.40 – 8.32 (m, 1H), 8.21 – 8.17 (m, 1H), 8.13 (s, 1H), 8.09 (dd, *J* = 7.6, 0.8 Hz, 1H), 7.93 – 7.90 (m, 1H), 7.87 (s, 1H), 7.84 (s, 1H), 7.70 (s, 1H), 7.68 (s, 1H), 7.61 (d, *J* = 8.2 Hz, 1H), 7.59 – 7.51 (m, 2H), 7.47 (t, *J* = 7.9 Hz, 1H), 4.52 (dt, *J* = 15.0, 7.6 Hz, 1H), 3.39 (d, *J* = 12.6 Hz, 2H), 3.11 – 2.98 (m, 2H), 2.46 (s, 3H), 2.20 – 2.13 (m, 4H); ^{13}C NMR (101 MHz, DMSO) δ 162.9, 153.0, 140.0, 140.0, 135.2, 134.7, 133.7, 132.8, 130.5, 128.3, 125.8, 125.7, 125.6, 125.3, 124.8, 122.6, 121.8, 121.7, 121.0, 119.3, 116.6, 116.2, 113.7, 55.1, 42.2, 28.7, 21.0; FTIR [cm^{-1}]: 3264, 3044, 2920, 2794, 2714, 1662, 1593, 1540, 1390, 1341; TLC-MS(ESI) *m/z*: 535.4 [M+H]$^{+}$; 569.5 [M+Cl]$^{-}$; HRMS(ESI) *m/z*: calcd. for [M+H]$^{+}$: 535.25637; found: 535.2547; HPLC t_{ret}: 9.19 min.

N-cyclopropyl-2-methyl-5-(4-(3-(naphthalen-1-yl)ureido)-1*H*-pyrazol-1-yl)benzamide (**45c**)

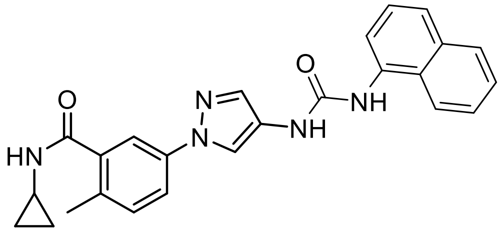

The reaction followed general procedure D: Urea-formation with isocyanates.

70 mg of (**44**) (0.273 mmol; 1 eq.) were dissolved in 11 mL of dry DCM. 39 µL of 1-isocyanatonaphthalene (0.273 mmol; 1 eq.) were added slowly via pipette. The reaction stirred for 24 h at RT. The reaction was quenched with MeOH, evaporated and purified via flash chromatography (MeOH/DCM: 3/97 – 10/90), to yield (**45c**) as a yellow-brown solid (44 mg; 42%). ^{1}H NMR (400 MHz, DMSO) δ 8.93 (s, 1H), 8.84 (s, 1H), 8.50 (s, 1H), 8.43 (d, J = 4.3 Hz, 1H), 8.11 (d, J = 8.3 Hz, 1H), 8.06 – 8.01 (m, 1H), 7.96 – 7.91 (m, 1H), 7.83 (s, 1H), 7.74 (dd, J = 8.3, 2.4 Hz, 1H), 7.68 (d, J = 2.3 Hz, 1H), 7.64 (d, J = 8.2 Hz, 1H), 7.62 – 7.53 (m, 2H), 7.48 (t, J = 7.9 Hz, 1H), 7.33 (d, J = 8.4 Hz, 1H), 2.88 – 2.80 (m, 1H), 2.33 (s, 3H), 0.75 – 0.67 (m, 2H), 0.59 – 0.52 (m, 2H); ^{13}C NMR (101 MHz, DMSO) δ 169.4, 152.8, 137.8, 137.4, 134.4, 133.7, 132.8, 132.6, 131.5, 128.4, 126.0, 125.9, 125.7, 124.5, 122.9, 121.4, 118.4, 117.3,

116.5, 116.4, 22.7, 18.8, 5.8; FTIR [cm^{-1}]: 3235, 3223, 3047, 3015, 1636, 1540, 1497, 1388, 1251, 1237; TLC-MS(ESI) *m/z*: 448.3 $[M+Na]^+$; 424.5 $[M-H]^-$; HRMS(ESI) *m/z*: calcd. for $[M+H]^+$: 426.19238; found: 426.1942; HPLC t_{ret}: 7.44 min.

10.19 Scaffold synthesis Table 7:

Scheme S7: Synthesis Table 7 and Table 8

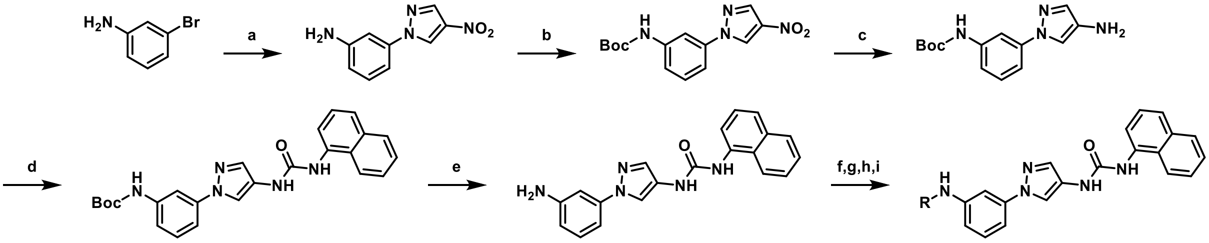

Reaction conditions and reagents exemplified by the synthesis of **51d**, **51a** (only step f)), **56d** (only step h)) and **51e** (only step i)): **a)** 4-nitro-1*H*-pyrazole (1 eq.), *m*-bromoaniline (1.2 eq.), K_2CO_3 (3 eq.), Cu(I)I (0.1 eq.), *trans-N,N*'-dimethylcyclohexane-1,2-diamine (0.2 eq.), DMF, 100 °C (41%); **b) 46** (1 eq.), Di-*tert*-butyl dicarbonate (1 eq.), *t*-BuOH, 40-50 °C (70%); **c) 47** (1 eq.), Fe(0) (5 eq.), NH_4Cl (5 eq.), EtOH:H_2O (3:1), 60 °C (76%); **d) 48** (1 eq.), 1-isocyanatonaphthalene (1 eq.), DCM (dry), RT (83%); **e) 49** (1 eq.), 1.25 M HCl (EtOH) (5 eq.), EtOH, 50-60 °C (80%, **HCl salt**); **f) 50** (1 eq.), isonicotinaldehyde (1.05 eq.), $NaCNBH_3$ (1.5 eq.), AcOH/AcONa (pH adjustment), RT (74%); **g) 50** (1 eq.), acetyl chloride (1 eq.), Pyridine, 0 °C-RT (54%); **h) 50** (1 eq.), HATU (2 eq.), DIPEA (5 eq.), **52** (acid) (1.2 eq.), DMF, RT, (13%); **i) 50** (1 eq.), cyclopropanesulfonyl chloride (1 eq.), THF: Pyridine (2:1), 0 °C-RT (18%).

3-(4-nitro-1*H*-pyrazol-1-yl)aniline (**46**)

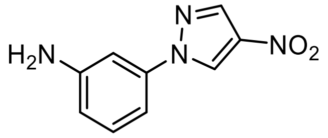

The reaction followed general procedure A: Ullmann-type reaction with pyrazoles.

3000 mg 4-nitro-1*H*-pyrazole (26.53 mmol; 1 eq.), 5477 mg m-bromoaniline (31.84 mmol; 1.2 eq.) and 10999 mg K_2CO_3 (79.59 mmol; 3 eq.) were suspended in 150 mL of DMF. After degasification of the mixture and after putting the system under argon, 0.837 mL of ligand (5.31 mmol; 0.2 eq.) and 505 mg of Cu(I)I (2.65 mmol; 0.1 eq.) were introduced. The reaction was sealed and heated to 100 °C for 20 h. The reaction was quenched with demin. H_2O. The precipitated, pure product was filtered of and washed thoroughly with water and Et_2O and dried in the convection oven to yield (**46**) as green-golden needles (2225 mg; 41%). ^{1}H NMR (400 MHz, DMSO) δ 9.44 (s, 1H), 8.48 (s, 1H), 7.16 (t, J = 8.0 Hz, 1H), 7.12 (t, J = 1.9 Hz, 1H), 7.02 (dd, J = 7.9, 1.2 Hz, 1H), 6.61 (dd, J = 8.0, 1.2 Hz, 1H), 5.49 (s, 2H); ^{13}C NMR (101 MHz, DMSO) δ 150.0, 139.2, 136.5, 136.5, 130.0, 127.7, 113.7, 106.3, 104.6; TLC-MS(ESI) *m/z*: 205.3 $[M+H]^+$; 203.1 $[M-H]^-$.

tert-butyl (3-(4-nitro-1*H*-pyrazol-1-yl)phenyl)carbamate (**47**)

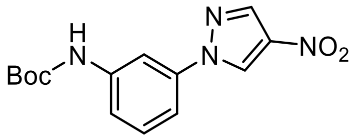

1800 mg of (**46**) (8.82 mmol; 1 eq.) and 1924 mg of Di-*tert*-butyl dicarbonate (8.82 mmol; 1 eq.) was suspended in 40 mL of *t*-BuOH. The reaction was heated to 40-50 °C for 20 h. After monitoring via HPLC showed full conversion, the reaction was quenched with EtOAc. The organic phase was washed 2x with brine. The aqueous phase was re-extracted 1x with EtOAc. The combine organic layers were dried over Na_2SO_4, filtered and evaporated. The crude

product was then purified using flash chromatography (EtOAc/PE: 10/90 – 35/65) to obtain a yellowish oil in which grey-white solids precipitated after cooling. The solids were filtered and washed with Pentane to yield (**47**) as a grey-white solid (1875 mg; 70%). ^{1}H NMR (400 MHz, DMSO) δ 9.67 (s, 1H), 9.54 (s, 1H), 8.54 (s, 1H), 8.20 (s, 1H), 7.56 – 7.50 (m, 1H), 7.45 – 7.39 (m, 2H), 1.49 (s, 9H); ^{13}C NMR (101 MHz, DMSO) δ 152.7, 140.8, 138.7, 136.8, 136.7, 129.9, 128.1, 117.7, 112.8, 109.4, 79.6, 28.1; TLC-MS(ESI) *m/z*: 327.1 $[M+Na]^+$; 303.0 $[M-H]^-$.

tert-butyl (3-(4-amino-1*H*-pyrazol-1-yl)phenyl)carbamate (**48**)

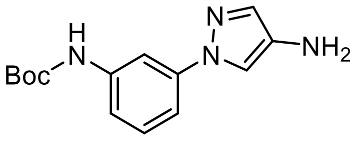

The reaction followed general procedure C: Reduction of *N*-arylated-4-nitropyrazoles.

1490 mg of (**47**) (4.90 mmol; 1 eq.) were combined with 1367 mg of Fe powder (24.48 mmol; 5 eq.) and 1310 mg of NH_4Cl (24.48 mmol; 5 eq.). The solids were then suspended in 40 mL of a mixture of EtOH:H_2O (3:1). The reaction was heated to 60°C for 1 h. After cooling to RT the reaction was filtered through celite. The alcoholic components were evaporated and the remaining suspension was diluted with EtOAc and subsequently washed 2x with 1 M NaOH (aq.). The combined organic layers were dried over Na_2SO_4, filtered and evaporated to dryness. The crude product was purified using flash chromatography (MeOH/DCM: 3/97 – 10/90) to yield (**48**) as a red solid (1020 mg; 76%). ^{1}H NMR (400 MHz, DMSO) δ 9.47 (s, 1H), 7.99 – 7.88 (m, 1H), 7.55 (d, J = 0.6 Hz, 1H), 7.31 – 7.16 (m, 4H), 4.18 (br s, 2H), 1.48 (s, 9H); ^{13}C NMR (101 MHz, DMSO) δ 152.7, 140.5, 140.4, 133.5, 132.5, 129.5, 114.5, 112.0, 110.6, 106.9, 79.2, 28.1; TLC-MS(ESI) *m/z*: 297.2 $[M+Na]^+$.

tert-butyl (3-(4-(3-(naphthalen-1-yl)ureido)-1*H*-pyrazol-1-yl)phenyl)carbamate (**49**)

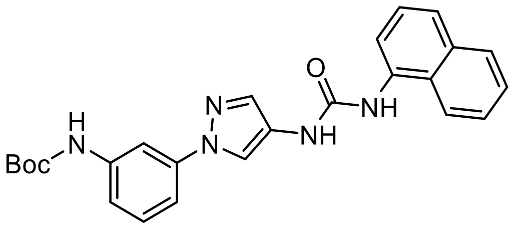

The reaction followed general procedure D: Urea-formation with isocyanates.

1725 mg of (**48**) (6.288 mmol; 1 eq.) were dissolved in 80 mL of dry DCM. 0.904 mL of 1-isocyanatonaphthalene (6.288 mmol; 1 eq.) were added slowly via syringe. The reaction stirred for 72 h at RT, while white-pinkish product precipitated. The solids were filtered off and washed with DCM and Pentane to yield (**49**) as a white-pinkish solid (2325 mg; 83%). ^{1}H NMR (400 MHz, DMSO) δ 9.55 (s, 1H), 8.95 (s, 1H), 8.84 (s, 1H), 8.38 (s, 1H), 8.12 (d, J = 8.3 Hz, 1H), 8.07 (s, 1H), 8.03 (d, J = 7.5 Hz, 1H), 7.93 (d, J = 7.9 Hz, 1H), 7.81 (s, 1H), 7.64 (d, J = 8.1 Hz, 1H), 7.57 (dt, J = 14.6, 7.0 Hz, 2H), 7.48 (t, J = 7.9 Hz, 1H), 7.39 – 7.28 (m, 3H), 1.49 (s, 9H); ^{13}C NMR (101 MHz, DMSO) δ 152.8, 152.7, 140.7, 140.2, 134.4, 133.8, 132.6, 129.7, 128.4, 126.1, 125.9, 125.7, 124.5, 123.0, 121.4, 117.5, 116.1, 115.4, 111.3, 107.5, 79.3, 28.1; TLC-MS(ESI) *m/z*: 466.1 $[M+Na]^+$; 442.1 $[M-H]^-$; 478.2 $[M+Cl]^-$.

1-(1-(3-aminophenyl)-1*H*-pyrazol-4-yl)-3-(naphthalen-1-yl)urea (**50**) (**HCl salt**)

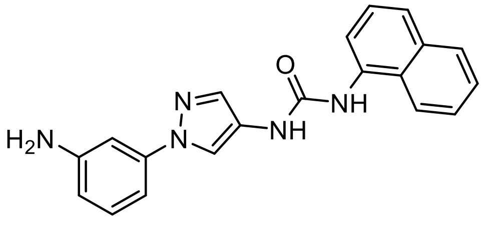

The reaction followed general procedure E: Boc deprotection.

1000 mg of (**49**) were dissolved in 30 mL of EtOH. Subsequently 9 mL of 1.25 M HCl (EtOH) (11.274 mmol; 5 eq.) were added and the reaction was heated to 50-60°C for 20 h. The pure product precipitated and was filtered off and washed with Pentane to yield (**50**) as a white-grey solid (687 mg; 80%; **HCl salt**). ^{1}H NMR (400 MHz, DMSO) δ 9.69 (s, 1H), 9.28 (s, 1H), 8.50 (s, 1H), 8.33 (d, J = 8.2 Hz, 1H), 8.07 (d, J = 7.5 Hz, 1H), 7.92 (d, J = 7.4 Hz, 1H), 7.85 (s, 2H), 7.80 (d, J = 8.0 Hz, 1H), 7.62 (d, J = 8.1 Hz, 1H), 7.59 – 7.51 (m, 3H), 7.46 (t, J = 7.9 Hz, 1H), 7.24 (d, J = 7.5 Hz, 1H), 5.14 (br s, 3H); ^{13}C NMR (101 MHz, DMSO) δ 153.0, 140.6, 134.7, 134.6, 133.8, 133.3, 130.9, 128.3, 125.9, 125.8, 125.6, 125.1, 122.7, 121.8, 119.3, 116.9, 116.0, 115.7, 111.8; TLC-MS(ESI) *m/z*: 365.9 $[M+Na]^+$; 397.9 $[M+Na+MeOH]^+$; 341.8 $[M-H]^-$; 377.8 $[M+Cl]^-$;

10.20 Compound synthesis Table 7:

1-(naphthalen-1-yl)-3-(1-(3-((pyridin-4-ylmethyl)amino)phenyl)-1*H*-pyrazol-4-yl)urea (**51a**)

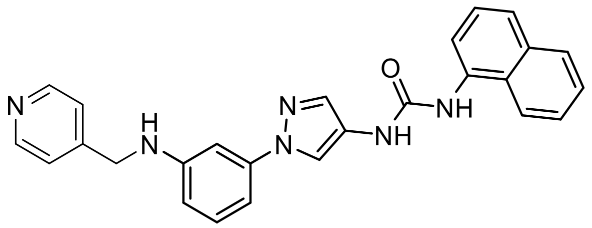

100 mg of (**50**) (**HCl salt**) (0.263 mmol; 1 eq.) and 29.6 mg of isonicotinaldehyde (0.276 mmol; 1.05 eq.) were suspended in 8 mL of MeOH. Subsequently AcOH and AcONa were added to adjust the pH to about 5. The reaction was left to stir for 1h. Afterwards 25 mg of $NaCNBH_3$ (0.395 mmol; 1.5 eq) were added and the reaction was allowed to stir for 20 h. The organic solvent was evaporated and the solids were reconstituted with EtOAc and 1 M NaOH (aq.) solution. The mixture was extracted 2x with EtOAc, dried over Na_2SO_4, filtered and evaporated. The crude product was further purified via flash chromatography (MeOH/DCM: 2.5/97.5 – 10/90 + 2 M NH_3) to yield (**51a**) as a beige-white solid (77 mg; 74%). ^{1}H NMR (400 MHz, DMSO) δ 8.92 (s, 1H), 8.83 (s, 1H), 8.50 (dd, J = 4.6, 1.4 Hz, 2H), 8.32 (s, 1H), 8.11 (d, J = 8.3 Hz, 1H), 8.02 (d, J = 7.1 Hz, 1H), 7.93 (d, J = 7.7 Hz, 1H), 7.76 (s, 1H), 7.66 – 7.51 (m, 3H), 7.47 (t, J = 7.9 Hz, 1H), 7.37 (d, J = 5.8 Hz, 2H), 7.13 (t, J = 8.0 Hz, 1H), 7.02 (t, J = 1.9 Hz, 1H), 6.93 (dd, J = 7.9, 1.4 Hz, 1H), 6.66 (t, J = 6.2 Hz, 1H), 6.46 (dd, J = 8.1, 1.7 Hz, 1H), 4.40 (d, J = 6.1 Hz, 2H); ^{13}C NMR (101 MHz, DMSO) δ 152.8, 149.6, 149.4, 149.3, 140.7, 134.5, 133.7, 132.3, 129.9, 128.4, 126.0, 125.9, 125.7, 124.2, 122.9, 122.2, 121.4, 117.3, 116.2, 109.9, 105.7, 101.8, 45.3; FTIR [cm^{-1}]: 3277, 3052, 2849, 1638, 1552, 1497, 1449, 1388, 1319, 1244; TLC-MS(ESI) *m/z*: 434.8 $[M+H]^+$; 456.8 $[M+Na]^+$; 432.9 $[M-H]^-$; 468.8 $[M+Cl]^-$; HRMS(ESI) *m/z*: calcd. for $[M+H]^+$: 435.19271; found: 435.1925; HPLC t_{ret}: 5.82 min.

1-(1-(3-((cyclopropylmethyl)amino)phenyl)-1*H*-pyrazol-4-yl)-3-(naphthalen-1-yl)urea (**51b**)

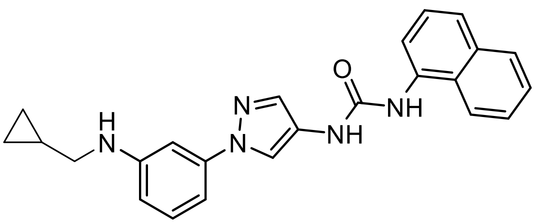

100 mg of (**50**) (**HCl salt**) (0.263 mmol; 1 eq.) and 21 µL of cyclopropyl aldehyde (0.276 mmol; 1.05 eq.) were suspended in 10 mL of MeOH. AcOH and AcONa were added to adjust the pH to about 5. The reaction was left to stir for 1h after which the reaction was cooled via ice-water bath to 0°C and 25 mg of $NaCNBH_3$ (0.395 mmol; 1.5 eq) were added. After 15 min the cooling was removed and the reaction was allowed to stir for 6h at RT. Subsequently, precipitated solids were dissolved by the addition of 10 ml DCM and the reaction is allowed to stir overnight. After 18h additional 20 µL of cyclopropyl aldehyde (0.263 mmol, 1 eq.) and 17 mg $NaCNBH_3$ (0.263 mmol;1 eq) were added at RT. The reaction showed full conversion after another 24h. The organic solvent was evaporated and the solids were reconstituted with EtOAc and 1 M NaOH (aq.) solution. The mixture was extracted with EtOAc and the organic phase was washed 2x with alkalized brine. The organic phase was dried over Na_2SO_4, filtered and evaporated. The crude product was further purified via flash chromatography (EtOAc/PE: 20/80 – 100/0) to yield (**51b**) as a brownish-white solid (52 mg; 50%). ^1H NMR (400 MHz, DMSO) δ 8.90 (s, 1H), 8.83 (s, 1H), 8.35 (s, 1H), 8.11 (d, J = 8.4 Hz, 1H), 8.03 (d, J = 7.5 Hz, 1H), 7.93 (d, J = 7.6 Hz, 1H), 7.77 (s, 1H), 7.64 (d, J = 8.2 Hz, 1H), 7.61 – 7.52 (m, 2H), 7.47 (t, J = 7.9 Hz, 1H), 7.14 (t, J = 8.0 Hz, 1H), 7.00 (t, J = 1.9 Hz, 1H), 6.90 (dd, J = 7.9, 1.3 Hz, 1H), 6.50 (dd, J = 8.1, 1.7 Hz, 1H), 5.95 (t, J = 5.4 Hz, 1H), 2.95 (t, J = 6.0 Hz, 2H), 1.09 – 1.03 (m, 1H), 0.52 – 0.45 (m, 2H), 0.26 – 0.21 (m, 2H); ^{13}C NMR (101 MHz, DMSO) δ 152.8, 150.0, 140.7, 134.5, 133.7, 132.2, 129.7, 128.4, 126.0, 125.9, 125.7, 124.1, 122.9, 121.4, 117.3, 116.3, 109.8, 105.0, 101.2, 47.4, 10.5, 3.5; FTIR [cm^{-1}]: 3377, 3272, 3001, 2922, 2851, 1639, 1588, 1560, 1497, 1437; TLC-MS(ESI) *m/z*: 420.3 $[M+Na]^+$; 452.3 $[M+Na+MeOH]^+$; 396.4 $[M-H]^-$; 432.4 $[M+Cl]^-$; HRMS(ESI) *m/z*: calcd. for $[M+H]^+$: 398.19746; found: 398.1982; HPLC t_{ret}: 8.59 min.

N-(3-(4-(3-(naphthalen-1-yl)ureido)-1*H*-pyrazol-1-yl)phenyl)cyclopropanecarboxamide (**51c**)

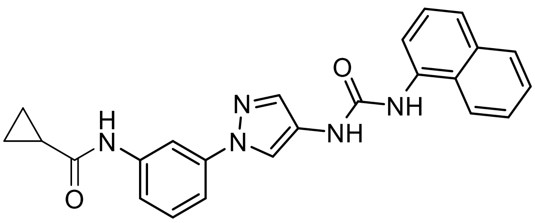

The reaction followed general procedure B3: Amide coupling using acid chlorides.

50 mg of (**50**) (**HCl salt**) (0.132 mmol; 1 eq.) were dissolved in 8 mL of dry Pyridine. The reaction was cooled via ice-water bath and dropwise 12 µL of cyclopropanecarbonyl chloride (0.132 mmol; 1 eq.) were added to the solution. The reaction stirred for 5 min after which the cooling was removed and reaction was left to stir for 22 h at RT. The reaction was quenched with 10% HCl (aq.) solution and EtOAc. The mixture was washed 3x with 10% HCl (aq.)solution. The aquatic phase was subsequently 1x re-extracted with EtOAc and the combined organic phases were again washed 1x against saturated $NaHCO_3$ (aq.) solution and 1x against brine. The combined organic phase was dried over Na_2SO_4,filtered and evaporated. The crude product was further purified using flash chromatography (MeOH/DCM: 1/99 – 10/90) to yield (**51c**) as a white solid (40 mg; 74%). ^1H NMR (400 MHz, DMSO) δ 10.38 (s, 1H), 8.96 (s, 1H), 8.85 (s, 1H), 8.41 (s, 1H), 8.17 (s, 1H), 8.11 (d, J = 8.3 Hz, 1H), 8.03 (d, J = 7.5 Hz,

1H), 7.93 (d, J = 7.9 Hz, 1H), 7.82 (s, 1H), 7.66 – 7.52 (m, 3H), 7.51 – 7.36 (m, 4H), 1.84 – 1.75 (m, 1H), 0.89 – 0.75 (m, 4H); ^{13}C NMR (101 MHz, DMSO) δ 171.9, 152.8, 140.4, 140.1, 134.4, 133.7, 132.7, 129.8, 128.4, 126.0, 125.9, 125.7, 124.6, 123.0, 121.4, 117.4, 116.0, 112.1, 108.4, 14.6, 7.3; FTIR [cm^{-1}]: 3311, 3280, 3099, 3078, 3010, 2920, 1644, 1609, 1588, 1551; TLC-MS(ESI) *m/z*: 434.1 [M+Na]$^+$; 410.1 [M-H]$^-$; 446.1 [M+Cl]$^-$; HRMS(ESI) *m/z*: calcd. for [M+H]$^+$: 412.17672; found: 412.1767; HPLC t_{ret}: 7.86 min.

N-(3-(4-(3-(naphthalen-1-yl)ureido)-1*H*-pyrazol-1-yl)phenyl)acetamide (**51d**)

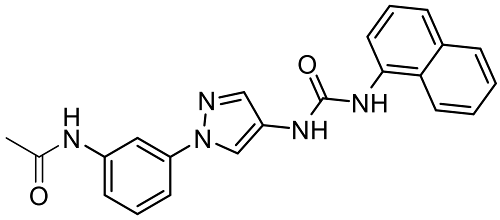

The reaction followed general procedure B3: Amide coupling using acid chlorides.

600 mg of (**50**) (**HCl salt**) (1.747 mmol; 1 eq.) were dissolved in 40 mL of dry Pyridine. The reaction was cooled via ice-water bath and dropwise 125 µL of acetyl chloride (1.747 mmol; 1 eq.) were added to the solution. The reaction stirred for 5 min after which the cooling was removed and reaction was left to stir for 20 h at RT. The reaction was quenched with 10% HCl (aq.) solution and EtOAc. The mixture was extracted 2x with EtOAc, washed 2x with 10% HCl (aq.)solution as well as 2x against saturated $NaHCO_3$ (aq.) solution. The combined organic phase was dried over Na_2SO_4,filtered and evaporated. The crude product was further purified using flash chromatography (MeOH/DCM: 2/98 – 10/90) to yield (**51d**) as a beige-white solid (366 mg; 60%). ^{1}H NMR (400 MHz, DMSO) δ 10.11 (s, 1H), 9.03 (s, 1H), 8.89 (s, 1H), 8.40 (s, 1H), 8.16 – 8.11 (m, J = 8.0 Hz, 2H), 8.05 – 8.00 (m, J = 7.0 Hz, 1H), 7.95 – 7.91 (m, J = 7.8 Hz, 1H), 7.82 (s, 1H), 7.64 (d, J = 8.2 Hz, 1H), 7.61 – 7.52 (m, 2H), 7.50 – 7.42 (m, 3H), 7.41 – 7.36 (m, 1H), 2.08 (s, 3H); ^{13}C NMR (101 MHz, DMSO) δ 168.5, 152.8, 140.4, 140.1, 134.5, 133.7, 132.7, 129.8, 128.4, 126.0, 125.9, 125.7, 124.6, 122.9, 121.4, 117.4, 116.1, 116.1, 112.2, 108.4, 24.1; FTIR [cm^{-1}]: 3271, 3101, 3055, 2919, 1654, 1644, 1609, 1587, 1547, 1491; TLC-MS(ESI) *m/z*: 407.9 [M+Na]$^+$; 384.1 [M-H]$^-$; 420.1 [M+Cl]$^-$; HRMS(ESI) *m/z*: calcd. for [M+H]$^+$: 386.16107; found: 386.1615; HPLC t_{ret}: 6.91 min.

N-(3-(4-(3-(naphthalen-1-yl)ureido)-1*H*-pyrazol-1-yl)phenyl)cyclopropanesulfonamide (**51e**)

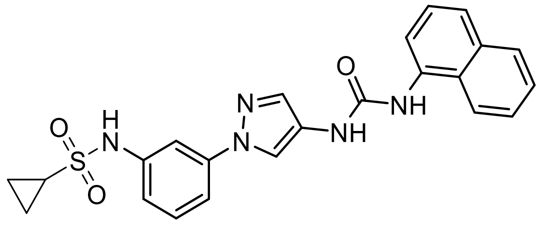

80 mg of (**50**) (**HCl salt**) (0.233 mmol; 1 eq.) were dissolved in 12 mL of a mixture of dry THF:Pyridine (2:1). The reaction was cooled via ice-water bath and dropwise 24 µL of cyclopropanesulfonyl chloride (0.233 mmol; 1 eq.) were added to the solution. The reaction stirred for 5 min after which the cooling was removed and reaction was left to stir for 20 h at RT. The reaction was quenched with 10% HCl (aq.) solution and EtOAc. The mixture was extracted 3x with EtOAc, washed 1x with 10% HCl (aq.)solution, 1x against 1 M NaOH (aq.) solution and 1x against brine. The combined organic phase was dried over Na_2SO_4,filtered and evaporated. The crude product was further purified using flash chromatography (MeOH/DCM: 4/96 – 10/90) to yield (**51e**) as a beige-brown solid (19 mg; 20%). ^{1}H NMR (400 MHz, DMSO) δ 9.92 (br s, 1H), 9.00 (s, 1H), 8.87 (s, 1H), 8.43 (s, 1H), 8.12 (d, J = 8.3 Hz, 1H), 8.02 (d, J =

7.4 Hz, 1H), 7.94 (d, J = 7.7 Hz, 1H), 7.84 (s, 1H), 7.77 – 7.69 (m, 1H), 7.65 (d, J = 8.1 Hz, 1H), 7.62 – 7.52 (m, 2H), 7.52 – 7.44 (m, 2H), 7.41 (t, J = 8.1 Hz, 1H), 7.19 – 7.07 (m, 1H), 2.75 – 2.62 (m, 1H), 1.02 – 0.89 (m, 4H); ^{13}C NMR (101 MHz, DMSO) δ 152.8, 140.4, 139.7, 134.4, 133.8, 133.0, 130.3, 128.4, 126.0, 125.9, 125.9, 125.7, 124.7, 123.0, 121.4, 117.4, 117.0, 116.2, 112.7, 109.2, 29.6, 5.0; FTIR [cm^{-1}]: 3292, 3202, 3122, 3053, 2923, 2850, 1643, 1600, 1586, 1550; TLC-MS(ESI) *m/z*: 470.2 [M+Na]$^+$; 446.2 [M-H]$^-$; 482.3 [M+Cl]$^-$; HRMS(ESI) *m/z*: calcd. for [M+H]$^+$: 448.14371; found: 448.1437; HPLC t_{ret}: 7.62 min.

N-(3-(4-(3-(naphthalen-1-yl)ureido)-1*H*-pyrazol-1-yl)phenyl)-1*H*-pyrrole-3-carboxamide (**51f**)

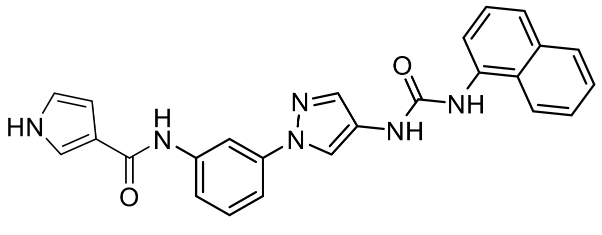

The reaction followed general procedure B4: Amide coupling using HATU.

80 mg of 1*H*-pyrrole-3-carboxylic acid (0.720 mmol; 1 eq.) and 342 mg of HATU (0.90 mmol; 1.25 eq) were dissolved in 20 mL of dry DMF. 0.367 mL of DIPEA (2.160 mmol; 3 eq.) were added to the solution and the reaction was allowed to stir for 14 h at RT. After a reaction control showed full activation of the corresponding carboxylic acid, 235mg of (**50**) (**HCl salt**) (0.684 mmol; 0.95 eq.) were added and the reaction was left to stir for another 6 h at RT. Since the conversion was inadequate 1 eq. of acid, 1 eq. of HATU and 3 eq. of DIPEA were added. The reaction was left to stir for 14 h and then quenched with demin. water. The precipitated product was filtered off and further purified using flash chromatography (MeOH/DCM) to yield (**51f**) as a red-beige solid (17 mg; 6%). ^{1}H NMR (400 MHz, DMSO) δ 11.33 (s, 1H), 9.66 (s, 1H), 9.04 (s, 1H), 8.91 (s, 1H), 8.43 (s, 1H), 8.25 (s, 1H), 8.13 (d, J = 8.2 Hz, 1H), 8.03 (d, J = 7.4 Hz, 1H), 7.94 (d, J = 7.8 Hz, 1H), 7.82 (s, 1H), 7.73 (d, J = 7.6 Hz, 1H), 7.66 – 7.52 (m, 4H), 7.50 – 7.36 (m, 3H), 6.84 (d, J = 1.5 Hz, 1H), 6.68 (s, 1H); ^{13}C NMR (101 MHz, DMSO) δ 163.0, 152.8, 141.0, 140.0, 134.5, 133.7, 132.6, 129.6, 128.4, 126.0, 125.9, 125.7, 124.5, 123.0, 121.6, 121.4, 119.4, 118.9, 117.3, 116.8, 116.0, 111.8, 109.1, 107.8; FTIR [cm^{-1}]: 3254, 1636, 1597, 1540, 1526, 1448, 1388, 1334, 1247, 1196; TLC-MS(ESI) *m/z*: 459.3 [M+Na]$^+$; 435.4 [M-H]$^-$; 471.1 [M+Cl]$^-$; HRMS(ESI) *m/z*: calcd. for [M+H]$^+$: 437.17197; found: 437.1741; HPLC t_{ret}: 7.18 min.

2,2,2-trifluoro-*N*-(3-(4-(3-(naphthalen-1-yl)ureido)-1*H*-pyrazol-1-yl)phenyl)acetamide (**51g**)

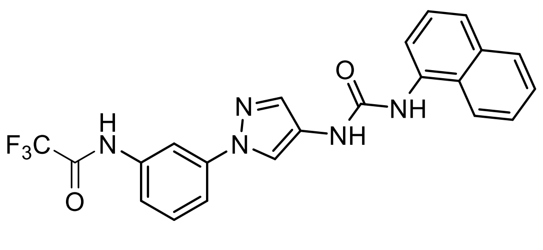

100 mg of (**50**) (**HCl salt**) (0.263 mmol; 1 eq.) was dissolved in 10 mL of dry THF. 73 µL of TEA (0.527 mmol; 2 eq.) were added. The reaction was cooled via ice-water bath and subsequently 44 µL of trifluoroacetic anhydride (0.316 mmol; 1.2 eq.) were added. The reaction stirred for 30 min after which the cooling was removed and the reaction was allowed to warm to RT and stir for 23,5 h. The precipitating product was filtered off and further purified via flash chromatography (MeOH/DCM: 1/99 – 10/90) to yield (**51g**) as a white solid (43 mg; 37%). ^{1}H NMR (400 MHz, DMSO) δ 11.41 (s, 1H), 8.98 (s, 1H), 8.86 (s, 1H), 8.48 (s, 1H), 8.21 (t, J = 2.0 Hz, 1H), 8.12 (d, J = 8.4 Hz, 1H), 8.03 (dd, J = 7.6, 0.8 Hz, 1H), 7.96 – 7.91 (m, 1H), 7.86

(s, 1H), 7.68 – 7.63 (m, 2H), 7.62 – 7.57 (m, 2H), 7.57 – 7.45 (m, 3H); ^{13}C NMR (101 MHz, DMSO) δ 154.65 (q, J = 37.4 Hz), 152.8, 140.1, 137.4, 134.4, 133.7, 133.1, 130.2, 128.4, 126.0, 125.9, 125.9, 125.7, 124.8, 123.0, 121.4, 118.0, 117.4, 116.1, 115.7 (q, J = 288.4 Hz),114.6, 110.5; FTIR [cm^{-1}]: 3343, 3273, 1735, 1706, 1648, 1597, 1560, 1497, 1463, 1390; TLC-MS(ESI) *m/z*: 461.9 $[M+Na]^+$; 438.0 $[M-H]^-$; HRMS(ESI) *m/z*: calcd. for $[M+H]^+$: 440.13281; found: 440.1336; HPLC t_{ret}: 8.29 min.

10.21 Scaffold synthesis Table 8:

3-acrylamidobenzoic acid (**52**)

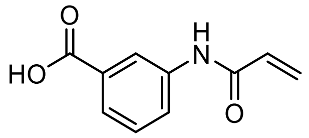

The reaction followed general procedure B3: Amide coupling using acid chlorides.

300 mg of 3-aminobenzoic acid (2.188 mmol; 1 eq.) were dissolved in 2.5 mL of dry DMF. 150 µL of Pyridine (1.859 mmol; 0.85 eq) were added and the reaction was cooled via ice-water bath. Dropwise 178 µL of acryloyl chloride (2.188 mmol; 1 eq.) were added to the solution. The reaction stirred for 5 min after which the cooling was removed and reaction was left to stir for 2 h at RT. The reaction was poured onto approximately 50 mL of acidified H_2O. The suspension was extracted 3x with EtOAc. The combined organic phase was washed 1x with brine, dried with Na_2SO_4,filtered and evaporated. The crude product was suspended in ACN and subsequently filtered off and dried to yield (**52)** (Purity: 94.6% at 254 nm; 91.8% at 230 nm) as a white solid (188 mg; 50%). ^{1}H NMR (400 MHz, DMSO) δ 12.97 (s, 1H), 10.32 (s, 1H), 8.29 (t, J = 1.8 Hz, 1H), 7.91 (ddd, J = 8.1, 2.1, 1.0 Hz, 1H), 7.67 – 7.61 (m, 1H), 7.45 (t, J = 7.9 Hz, 1H), 6.43 (dd, J = 17.0, 10.1 Hz, 1H), 6.28 (dd, J = 17.0, 2.0 Hz, 1H), 5.78 (dd, J = 10.0, 2.0 Hz, 1H); ^{13}C NMR (101 MHz, DMSO) δ 167.1, 163.3, 139.2, 131.7, 131.3, 129.1, 127.3, 124.3, 123.4, 120.1; TLC-MS(ESI) *m/z*: 236.0 $[M+2Na-H]^+$.

4-acrylamidobenzoic acid (**53**)

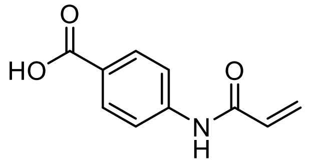

The reaction followed general procedure B3: Amide coupling using acid chlorides.

500 mg of 4-aminobenzoic acid (3.646 mmol; 1 eq.) were dissolved in 10 mL of dry DMF. 250 µL of Pyridine (3.099 mmol; 0.85 eq) were added and the reaction was cooled via Aceton-ice bath. Dropwise over 5 min, 297 µL of acryloyl chloride (3.646 mmol; 1 eq.) were added to the solution. The reaction was left to stir for 16 h in which it slowly warms up to RT. The reaction was poured onto acidified H_2O. The suspension was extracted 3x with EtOAc. The combined organic phase was dried with Na_2SO_4,filtered and evaporated. Remaining DMF was distilled of azeotropically with Toluol. The crude product was filtered of, washed with Et_2O and was further purified via reverse-phase flash chromatography (ACN/Water: 10/90 – 55/45 + 0.1% TFA) to yield (**53**) as a white solid (357 mg; 57%). ^{1}H NMR (400 MHz, DMSO) δ 12.72 (s, 1H), 10.43 (s, 1H), 7.98 – 7.87 (m, 2H), 7.82 – 7.73 (m, 2H), 6.46 (dd, J = 17.0, 10.1 Hz, 1H), 6.30 (dd, J = 17.0, 1.9 Hz, 1H), 5.80 (dd, J = 10.1, 2.0 Hz, 1H); ^{13}C NMR (101 MHz, DMSO) δ 166.9, 163.5, 143.0, 131.6, 130.4, 127.7, 125.4, 118.7; TLC-MS(ESI) *m/z*: 236.0 $[M+2Na-H]^+$; 268.1 $[M+2Na-H+MeOH]^+$; 190.0 $[M-H]^-$.

3-propionamidobenzoic acid (**54**)

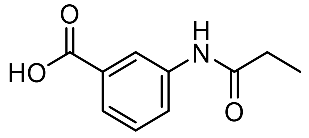

The reaction followed general procedure B3: Amide coupling using acid chlorides.

400 mg of 3-aminobenzoic acid (2.917 mmol; 1 eq.) were dissolved in 8 mL of dry Pyridine. While the reaction was cooled using an Aceton-ice bath, 255 µL of propionyl chloride (2.917 mmol; 1 eq.) were added dropwise via syringe. The reaction was allowed to come to RT and stirred for 4 d at RT. The reaction was diluted with EtOAc and 10% HCl (aq.) solution. The solution was extracted 3x with EtOAc. The organic phase was washed 2x with 10% HCl (aq.) solution, after which the organic phase was dried with Na_2SO_4,filtered and evaporated. The crude product was further purified using reverse-phase flash chromatography (ACN/H_2O: 5/95 – 100/0 + 0.1% TFA), to yield (**54**) as a white solid (405 mg; 80%). ^{1}H NMR (400 MHz, DMSO) δ 12.91 (s, 1H), 10.02 (s, 1H), 8.22 (s, 1H), 7.82 (dd, J = 8.1, 1.0 Hz, 1H), 7.64 – 7.54 (m, 1H), 7.40 (t, J = 7.9 Hz, 1H), 2.33 (q, J = 7.5 Hz, 2H), 1.09 (t, J = 7.5 Hz, 3H); ^{13}C NMR (101 MHz, DMSO) δ 172.2, 167.2, 139.6, 131.2, 128.9, 123.7, 123.1, 119.8, 29.5, 9.6; TLC-MS(ESI) *m/z*: 238.0 [M+2Na-H]$^+$; 270.0 [M+2Na-H+MeOH]$^+$; 192.0 [M-H]$^-$.

4-propionamidobenzoic acid (**55**)

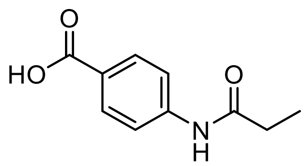

The reaction followed general procedure B3: Amide coupling using acid chlorides.

400 mg of 4-aminobenzoic acid (2.917 mmol; 1 eq.) were dissolved in 8 mL of dry Pyridine. While the reaction was cooled using an Aceton-ice bath, 255 µL of propionyl chloride (2.917 mmol; 1 eq.) were added dropwise via syringe. The reaction was allowed to come to RT and stirred for 4 d at RT. The reaction was diluted with EtOAc and 10% HCl (aq.) solution. The solution was extracted 3x with EtOAc. The organic phase was washed 2x with 10% HCl (aq.) solution, after which the organic phase was dried with Na_2SO_4,filtered and evaporated. The crude product was further purified using reverse-phase flash chromatography (ACN/H_2O: 8/92 – 100/0 + 0.1% TFA), to yield (**55**) as white crystals (372 mg; 73%). ^{1}H NMR (400 MHz, DMSO) δ 12.66 (br s, 1H), 10.15 (s, 1H), 7.87 (d, J = 8.7 Hz, 2H), 7.70 (d, J = 8.7 Hz, 2H), 2.35 (q, J = 7.5 Hz, 2H), 1.08 (t, J = 7.5 Hz, 3H); ^{13}C NMR (101 MHz, DMSO) δ 172.5, 167.0, 143.4, 130.4, 124.8, 118.2, 29.6, 9.5; TLC-MS(ESI) *m/z*: 238.0 [M+2Na-H]$^+$; 191.6 [M-H]$^-$;

10.22 Compound synthesis Table 8:

N-(3-(4-(3-(naphthalen-1-yl)ureido)-1*H*-pyrazol-1-yl)phenyl)-4-propionamidobenzamide (**56a**)

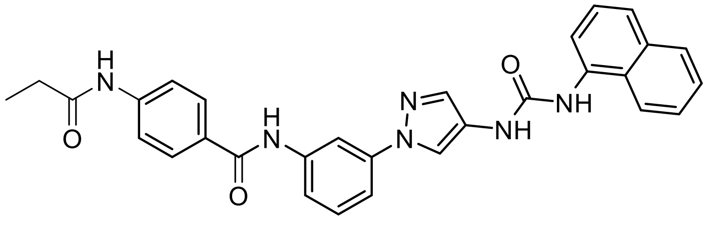

The reaction followed general procedure B4: Amide coupling using HATU.

25 mg of (**55**) (0.132 mmol; 1 eq.), 50 mg of (**50** HCl salt) (0.132 mmol; 1 eq.) as well as 100 mg of HATU (0.263 mmol; 2 eq.) were dissolved in 2 mL of dry DMF. 112 µL of DIPEA (0.658 mmol; 5 eq.) were added and the reaction stirred at RT for 18 h. The resulting mixture was directly purified via reversed phase flash chromatography (ACN/H_2O: 40/60 – 100/0 + TFA (0.1%)). After evaporation of the organic phase the compound precipitated, was filtrated off and washed with Et_2O and 20% MeOH in water to yield pure compound (**56a**) as a dark-brown

solid (5 mg; 7%). ^{1}H NMR (700 MHz, DMSO) δ 10.27 (s, 1H), 10.16 (s, 1H), 8.97 (s, 1H), 8.86 (s, 1H), 8.45 (s, 1H), 8.31 (t, J = 1.9 Hz, 1H), 8.12 (d, J = 8.4 Hz, 1H), 8.03 (d, J = 7.4 Hz, 1H), 7.97 (d, J = 8.7 Hz, 2H), 7.94 (d, J = 8.0 Hz, 1H), 7.83 (s, 1H), 7.79 – 7.71 (m, 3H), 7.65 (d, J = 8.1 Hz, 1H), 7.62 – 7.59 (m, 1H), 7.57 – 7.54 (m, 1H), 7.51 (dd, J = 8.1, 1.4 Hz, 1H), 7.48 (t, J = 7.9 Hz, 1H), 7.44 (t, J = 8.1 Hz, 1H), 2.37 (q, J = 7.5 Hz, 2H), 1.10 (t, J = 7.5 Hz, 3H); ^{13}C NMR (176 MHz, DMSO) δ 172.47, 165.03, 152.80, 142.51, 140.43, 140.00, 134.45, 133.74, 132.69, 129.70, 128.67, 128.61, 128.42, 125.98, 125.91, 125.71, 124.59, 122.96, 121.39, 118.14, 117.33, 117.27, 116.06, 112.62, 109.61, 29.59, 9.50; FTIR [cm^{-1}]: 3281, 3237, 3110, 2975, 1659, 1643, 1605, 1587, 1554, 1522; TLC-MS(ESI) *m/z*: 541.3 [M+Na]$^{+}$; 517.3 [M-H]$^{-}$; 553.2 [M+Cl]$^{-}$; HRMS(ESI) *m/z*: calcd. for [M+H]$^{+}$: 519.21384; found: 519.2137; HPLC t_{ret}: 7.93 min.

4-acrylamido-*N*-(3-(4-(3-(naphthalen-1-yl)ureido)-1*H*-pyrazol-1-yl)phenyl)benzamide (**56b**)

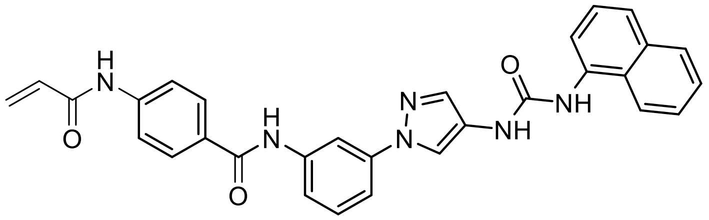

The reaction followed general procedure B4: Amide coupling using HATU.

63 mg of (**53**) (0.329 mmol; 1 eq.), 125 mg of (**50** HCl salt) (0.329 mmol; 1 eq.) as well as 250 mg of HATU (0.658 mmol; 2 eq.) were dissolved in 10 mL of dry DMF. 280 µL of DIPEA (1.645 mmol; 5 eq.) were added and the reaction stirred 92 h at RT. The resulting mixture was quenched with demin. H_2O and precipitating product was filtered off, washed with H_2O and Et_2O and dried. The compound was purified via flash chromatography (MeOH/DCM: 3.5/96.5 – 10/90). Ultimately, additional reversed phase flash chromatography purification (ACN/H_2O: 45/60 – 65/35 + TFA (0.1%)) yielded pure compound (**56b**) as a white solid (12 mg; 8%). ^{1}H NMR (700 MHz, DMSO) δ 10.44 (s, 1H), 10.31 (s, 1H), 8.97 (s, 1H), 8.86 (s, 1H), 8.46 (s, 1H), 8.32 (t, J = 1.8 Hz, 1H), 8.12 (d, J = 8.4 Hz, 1H), 8.03 (d, J = 7.4 Hz, 1H), 8.00 (d, J = 8.7 Hz, 2H), 7.94 (d, J = 8.0 Hz, 1H), 7.86 – 7.81 (m, 3H), 7.74 (d, J = 8.0 Hz, 1H), 7.65 (d, J = 8.1 Hz, 1H), 7.61 – 7.59 (m, 1H), 7.56 – 7.54 (m, 1H), 7.51 (dd, J = 8.1, 1.3 Hz, 1H), 7.48 (t, J = 7.8 Hz, 1H), 7.45 (t, J = 8.1 Hz, 1H), 6.48 (dd, J = 16.9, 10.2 Hz, 1H), 6.32 (dd, J = 17.0, 1.7 Hz, 1H), 5.82 (dd, J = 10.2, 1.7 Hz, 1H); ^{13}C NMR (176 MHz, DMSO) δ 165.0, 163.5, 152.8, 142.1, 140.4, 140.0, 134.5, 133.8, 132.7, 131.6, 129.7, 129.3, 128.8, 128.4, 127.7, 126.0, 125.9, 125.7, 124.6, 123.0, 121.4, 118.6, 117.3, 117.3, 116.1, 112.7, 109.6; FTIR [cm^{-1}]: 3247, 1664, 1643, 1605, 1590, 1558, 1524, 1495, 1464, 1410; TLC-MS(ESI) *m/z*: 539.3 [M+Na]$^{+}$; 515.4 [M-H]$^{-}$; HRMS(ESI) *m/z*: calcd. for [M+H]$^{+}$: 517.19819; found: 517.1972; HPLC t_{ret}: 7.86 min.

N-(3-(4-(3-(naphthalen-1-yl)ureido)-1*H*-pyrazol-1-yl)phenyl)-3-propionamidobenzamide (**56c**)

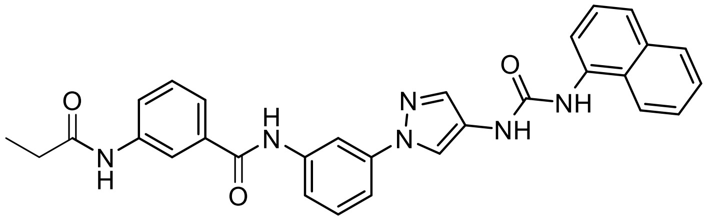

The reaction followed general procedure B4: Amide coupling using HATU.

76 mg of (**54**) (0.395 mmol; 1.2 eq.) as well as 213 mg of HATU (0.658 mmol; 2 eq.) were dissolved in 10 mL of dry DMF. 280 µL of DIPEA (1.645 mmol; 5 eq.) were added and the reaction stirred for 60 min at RT. Subsequently 125 mg of (**50** HCl salt) (0.329 mmol; 1 eq.)

were added and the reaction stirred at RT for another 18 h. The resulting mixture was quenched with demin. H_2O and precipitating product was filtered off. The compound was purified via reversed phase flash chromatography (ACN/H_2O: 30/70 – 60/40 + TFA (0.1%)) to yield pure compound (**56c**) as a light-brown solid (37 mg; 22%). ^{1}H NMR (700 MHz, DMSO) δ 10.41 (s, 1H), 10.08 (s, 1H), 8.97 (s, 1H), 8.86 (s, 1H), 8.46 (s, 1H), 8.31 (t, J = 1.9 Hz, 1H), 8.14 – 8.11 (m, 2H), 8.03 (d, J = 7.5 Hz, 1H), 7.94 (d, J = 8.0 Hz, 1H), 7.86 (dd, J = 8.1, 1.0 Hz, 1H), 7.84 (s, 1H), 7.75 – 7.72 (m, 1H), 7.66 – 7.63 (m, 2H), 7.61 – 7.58 (m, 1H), 7.56 – 7.51 (m, 2H), 7.50 – 7.44 (m, 3H), 2.36 (q, J = 7.6 Hz, 2H), 1.11 (t, J = 7.6 Hz, 3H); ^{13}C NMR (176 MHz, DMSO) δ 172.3, 165.8, 152.8, 140.3, 140.0, 139.6, 135.5, 134.5, 133.8, 132.7, 129.8, 128.8, 128.4, 126.0, 125.9, 125.7, 124.6, 123.0, 122.1, 121.9, 121.4, 118.6, 117.3, 116.1, 112.8, 109.7, 29.5, 9.6; FTIR [cm^{-1}]: 3270, 3110, 3058, 2968. 1639, 1605, 1588, 1543, 1496, 1485; TLC-MS(ESI) *m/z*: 541.3 [M+Na]$^+$; 517.4 [M-H]$^-$; HRMS(ESI) *m/z*: calcd. for [M+H]$^+$: 519.21384; found: 519.2138; HPLC t_{ret}: 7.98 min.

3-acrylamido-*N*-(3-(4-(3-(naphthalen-1-yl)ureido)-1*H*-pyrazol-1-yl)phenyl)benzamide (**56d**)

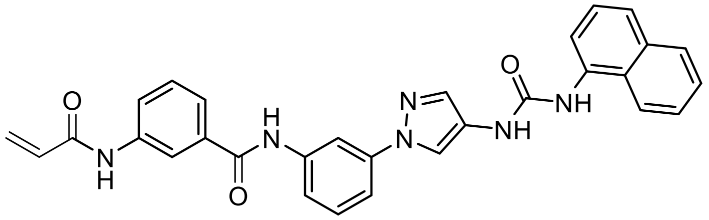

The reaction followed general procedure B4: Amide coupling using HATU.

60 mg of (**52**) (0.316 mmol; 1.2 eq.) as well as 200 mg of HATU (0.527 mmol; 2 eq.) were dissolved in 8 mL of dry DMF. 224 µL of DIPEA (1.316 mmol; 5 eq.) were added and the reaction stirred for 30 min at RT. Subsequently 100 mg of (**50** HCl salt) (0.263 mmol; 1 eq.) were added and the reaction stirred at RT for another 20 h. The resulting mixture was quenched with demin. H_2O and precipitating product was filtered off, washed with H_2O and Et_2O and dried. The compound was 2x purified via flash chromatography (MeOH/DCM: 2.5/97.5 – 10/90) as well as with redissolving the flashed product in DMF and precipitating via adding MeOH/Water mixtures. Ultimately, additional reversed phase flash chromatography purification (ACN/H_2O: 40/60 – 65/35 + TFA (0.1%)) yielded pure compound (**56d**) as a white solid (16 mg; 13%). ^{1}H NMR (700 MHz, DMSO) δ 10.44 (s, 1H), 10.37 (s, 1H), 8.97 (s, 1H), 8.86 (s, 1H), 8.46 (s, 1H), 8.32 – 8.30 (m, 1H), 8.21 – 8.18 (m, 1H), 8.12 (d, J = 8.4 Hz, 1H), 8.03 (d, J = 7.4 Hz, 1H), 7.97 – 7.92 (m, 2H), 7.84 (s, 1H), 7.74 (d, J = 8.0 Hz, 1H), 7.70 (d, J = 7.7 Hz, 1H), 7.65 (d, J = 8.1 Hz, 1H), 7.61 – 7.58 (m, 1H), 7.55 (d, J = 7.6 Hz, 1H), 7.54 – 7.49 (m, 2H), 7.49 – 7.44 (m, 2H), 6.47 (dd, J = 17.0, 10.2 Hz, 1H), 6.30 (dd, J = 17.0, 1.6 Hz, 1H), 5.80 (dd, J = 10.2, 1.6 Hz, 1H); ^{13}C NMR (176 MHz, DMSO) δ 165.7, 163.4, 152.8, 140.3, 140.0, 139.2, 135.6, 134.5, 133.7, 132.8, 131.7, 129.8, 128.9, 128.4, 127.3, 126.0, 125.9, 125.7, 124.6, 123.0, 122.5, 122.4, 121.4, 118.9, 117.3, 116.1, 112.9, 109.7; FTIR [cm^{-1}]: 3280, 3237, 3107, 1644, 1588, 1546, 1485, 1465, 1385, 1314; TLC-MS(ESI) *m/z*: 539.4 [M+Na]$^+$; 515.4 [M-H]$^-$; 551.4 [M+Cl]$^-$; HRMS(ESI) *m/z*: calcd. for [M+H]$^+$: 517.19819; found: 517.1988; HPLC t_{ret}: 7.94 min.

11 NMR/ HPLC-Analytics

For in vivo tested and representative compounds, HPLCs are shown.

11.1 Tabel 1

A-1:

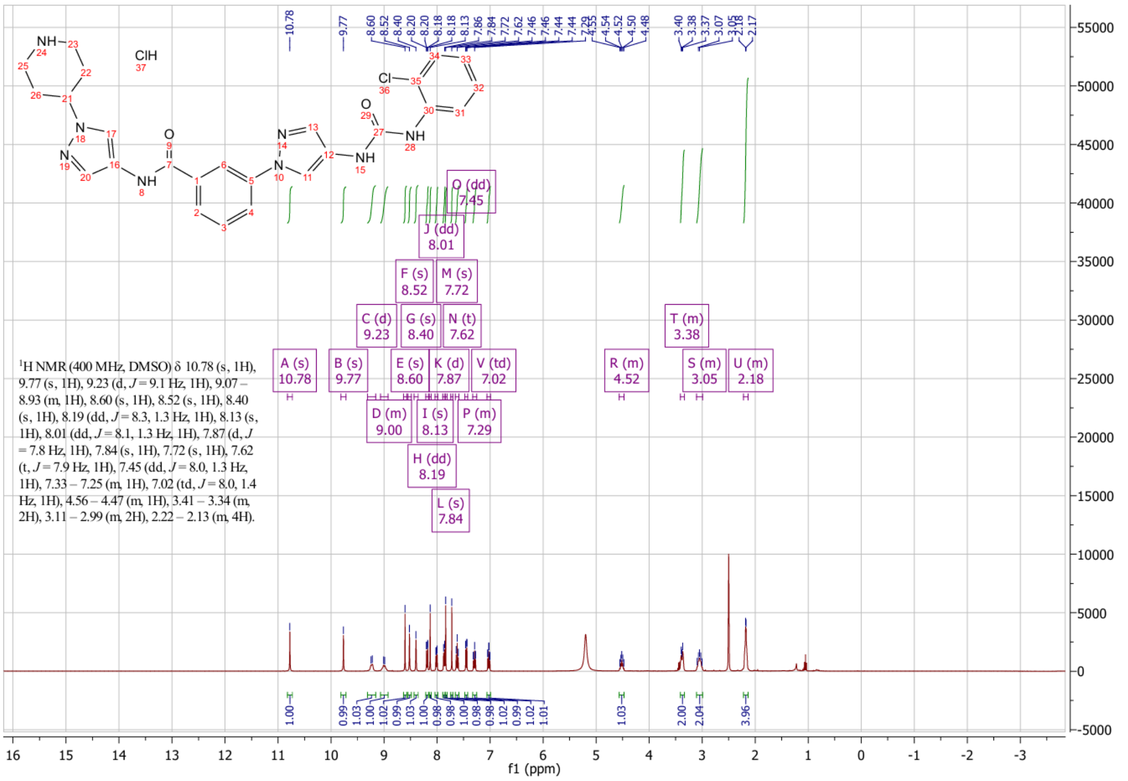

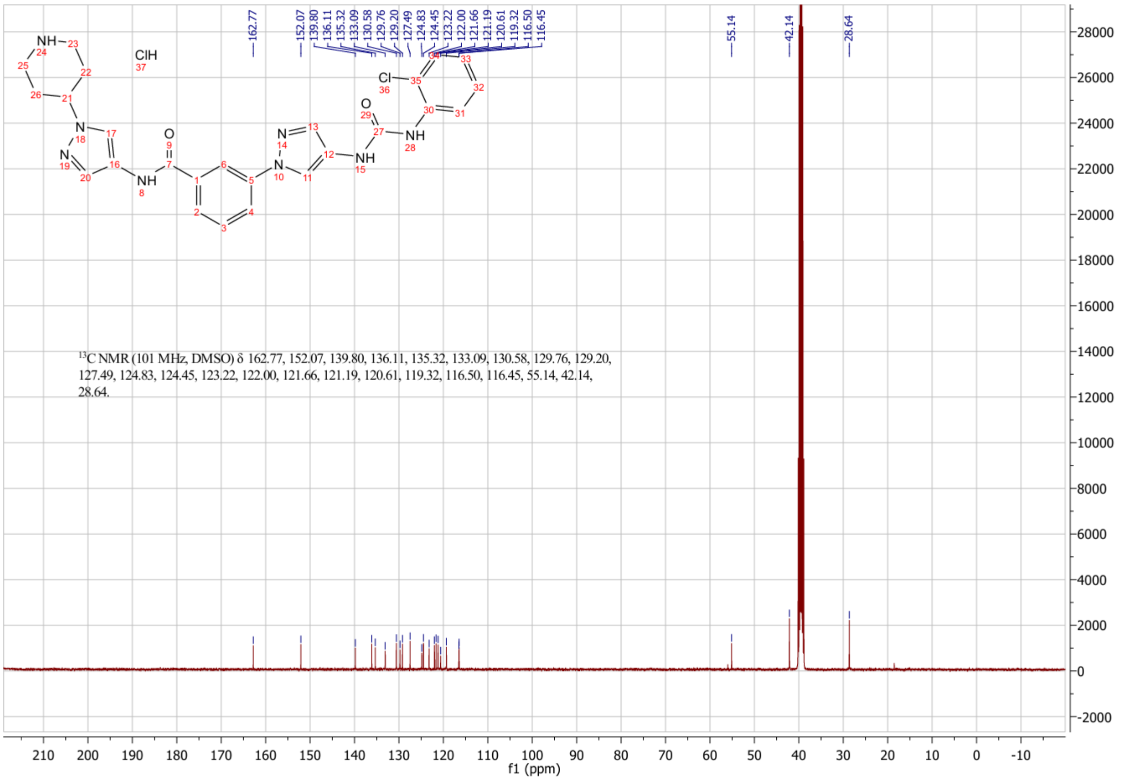

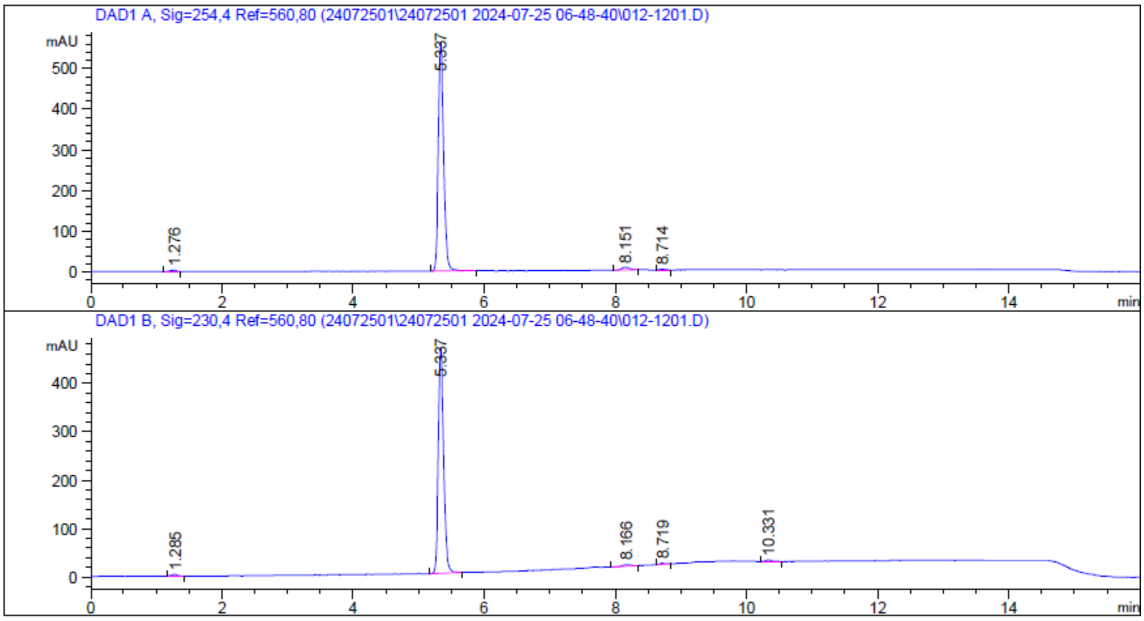

===

Area Percent Report

===

Sorted By : Signal
Multiplier: : 1.0000
Dilution: : 1.0000
Use Multiplier & Dilution Factor with ISTDs

Signal 1: DAD1 A, Sig=254,4 Ref=560,80

Peak #	RetTime [min]	Type	Width [min]	Area [mAU*s]	Height [mAU]	Area %
1	1.276	BB	0.0869	19.66621	3.06331	0.6003
2	5.337	BB	0.0863	3186.30688	561.01245	97.2556
3	8.151	BB	0.1191	57.52237	7.20527	1.7558
4	8.714	BB	0.0694	12.72240	2.58638	0.3883

Totals : 3276.21786 573.86742

Signal 2: DAD1 B, Sig=230,4 Ref=560,80

Peak #	RetTime [min]	Type	Width [min]	Area [mAU*s]	Height [mAU]	Area %
1	1.285	BB	0.0770	22.21203	3.98840	0.8214
2	5.337	BB	0.0860	2621.00024	462.98538	96.9247
3	8.166	BB	0.1138	26.03874	3.04379	0.9629
4	8.719	BB	0.0783	11.92199	2.16334	0.4409
5	10.331	BB	0.1008	22.98951	3.40310	0.8502

Totals : 2704.16251 475.58399

===

*** End of Report ***

1a

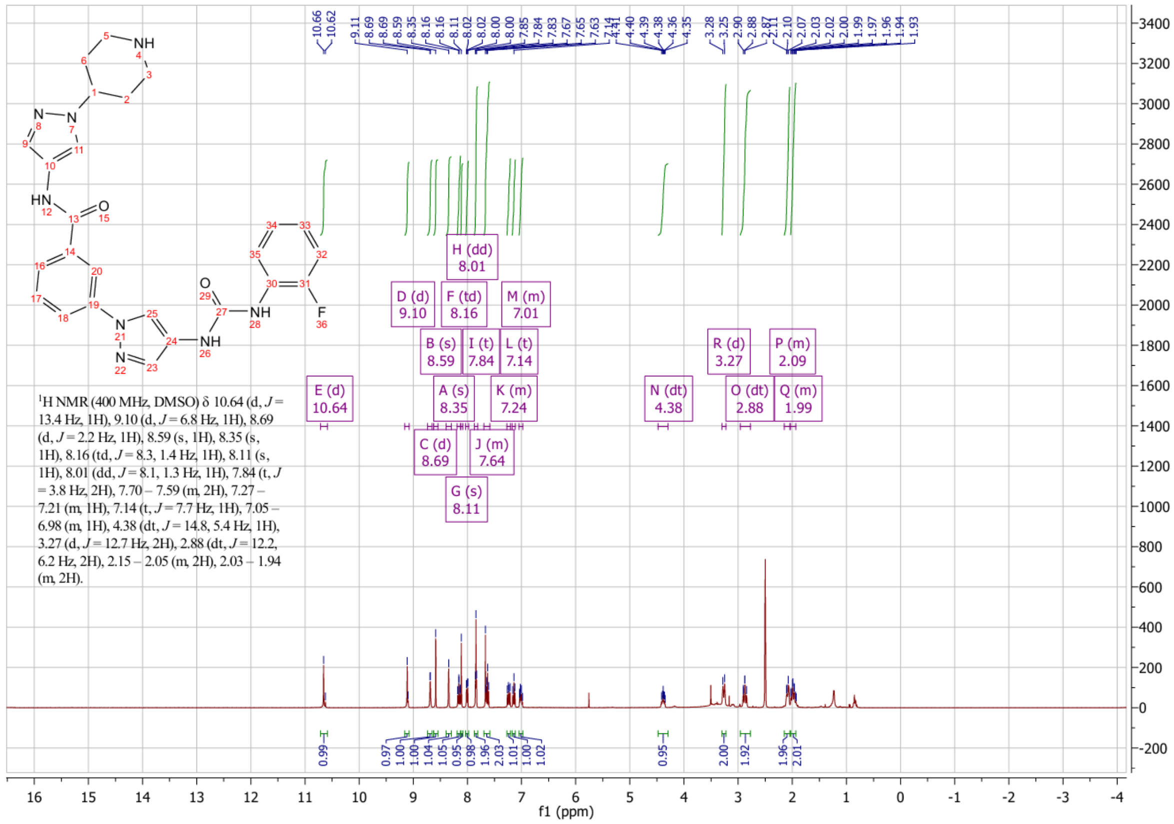

¹H NMR (400 MHz, DMSO) δ 10.64 (d, J = 13.4 Hz, 1H), 9.10 (d, J = 6.8 Hz, 1H), 8.69 (d, J = 2.2 Hz, 1H), 8.59 (s, 1H), 8.35 (s, 1H), 8.16 (td, J = 8.3, 1.4 Hz, 1H), 8.11 (s, 1H), 8.01 (dd, J = 8.1, 1.3 Hz, 1H), 7.84 (t, J = 3.8 Hz, 2H), 7.70 – 7.59 (m, 2H), 7.27 – 7.21 (m, 1H), 7.14 (t, J = 7.7 Hz, 1H), 7.05 – 6.98 (m, 1H), 4.38 (dt, J = 14.8, 5.4 Hz, 1H), 3.27 (d, J = 12.7 Hz, 2H), 2.88 (dt, J = 12.2, 6.2 Hz, 2H), 2.15 – 2.05 (m, 2H), 2.03 – 1.94 (m, 2H).
f1 (ppm)

1b

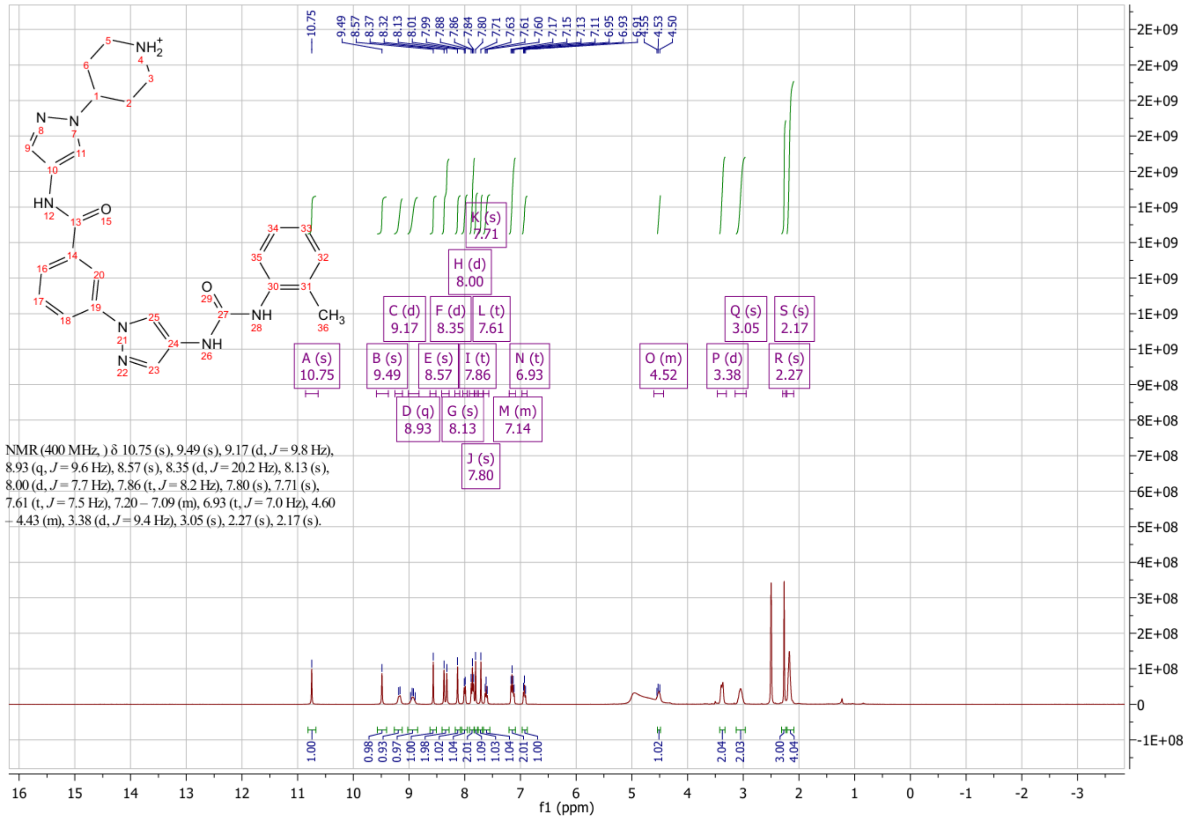
NMR (400 MHz,) δ 10.75 (s), 9.49 (s), 9.17 (d, J = 9.8 Hz), 8.93 (q, J = 9.6 Hz), 8.57 (s), 8.35 (d, J = 20.2 Hz), 8.13 (s), 8.00 (d, J = 7.7 Hz), 7.86 (t, J = 8.2 Hz), 7.80 (s), 7.71 (s), 7.61 (t, J = 7.5 Hz), 7.20 – 7.09 (m), 6.93 (t, J = 7.0 Hz), 4.60 – 4.43 (m), 3.38 (d, J = 9.4 Hz), 3.05 (s), 2.27 (s), 2.17 (s).
f1 (ppm)

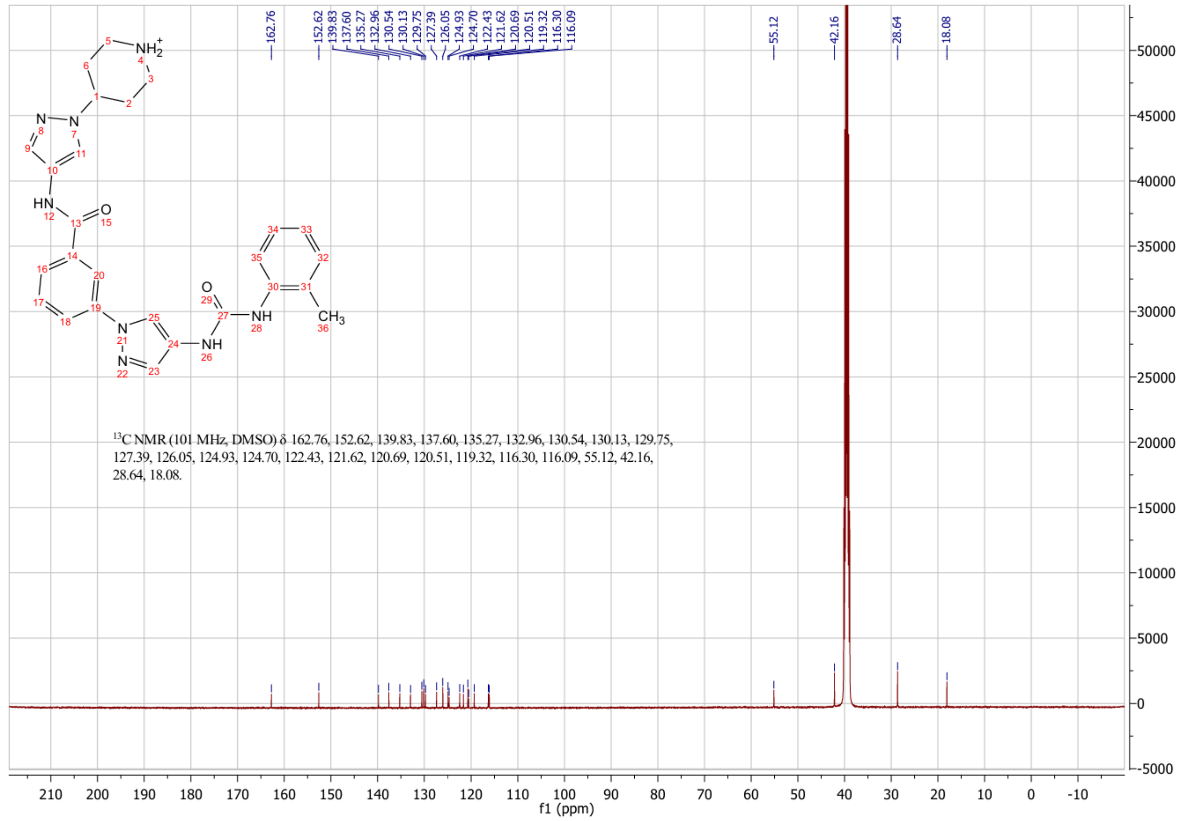
13C NMR (101 MHz, DMSO) δ 162.76, 152.62, 139.83, 137.60, 135.27, 132.96, 130.54, 130.13, 129.75, 127.39, 126.05, 124.93, 124.70, 122.43, 121.62, 120.69, 120.51, 119.32, 116.30, 116.09, 55.12, 42.16, 28.64, 18.08.
f1 (ppm)

1c

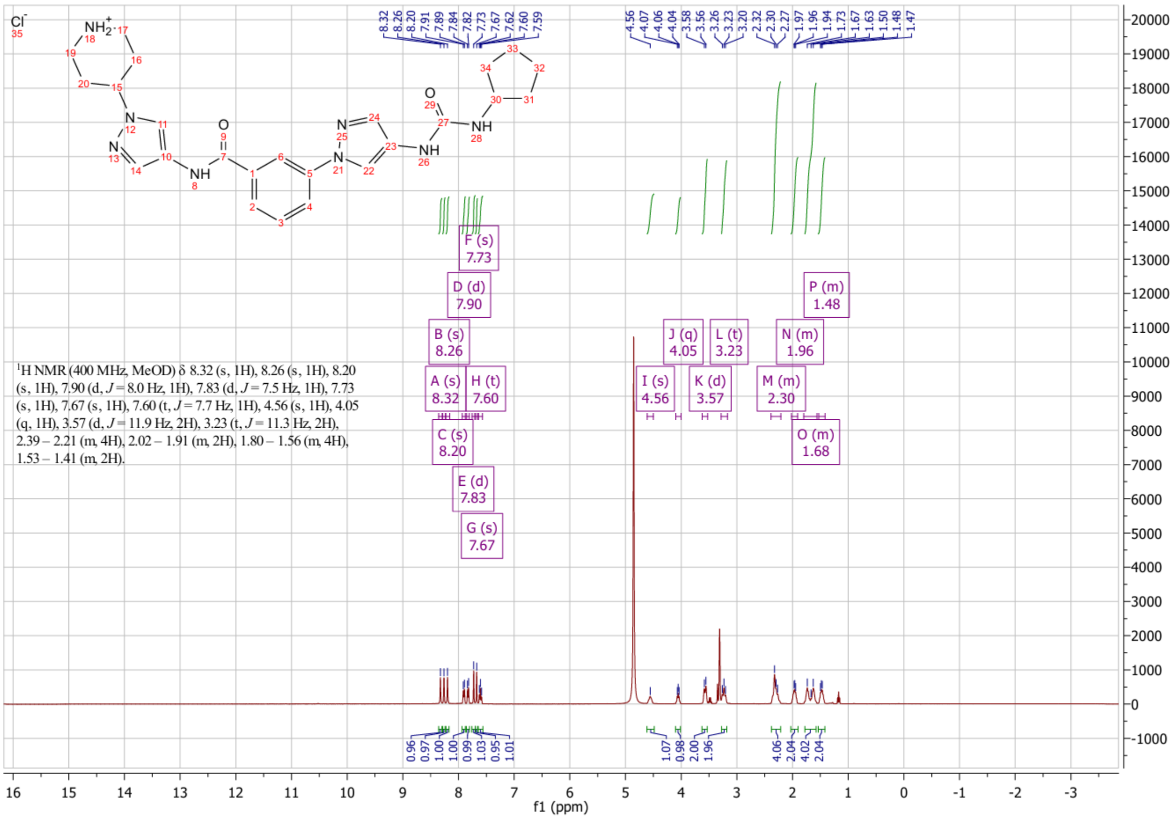

1H NMR (400 MHz, MeOD) δ 8.32 (s, 1H), 8.26 (s, 1H), 8.20 (s, 1H), 7.90 (d, J = 8.0 Hz, 1H), 7.83 (d, J = 7.5 Hz, 1H), 7.73 (s, 1H), 7.67 (s, 1H), 7.60 (t, J = 7.7 Hz, 1H), 4.56 (s, 1H), 4.05 (q, 1H), 3.57 (d, J = 11.9 Hz, 2H), 3.23 (t, J = 11.3 Hz, 2H), 2.39 – 2.21 (m, 4H), 2.02 – 1.91 (m, 2H), 1.80 – 1.56 (m, 4H), 1.53 – 1.41 (m, 2H).
f1 (ppm)

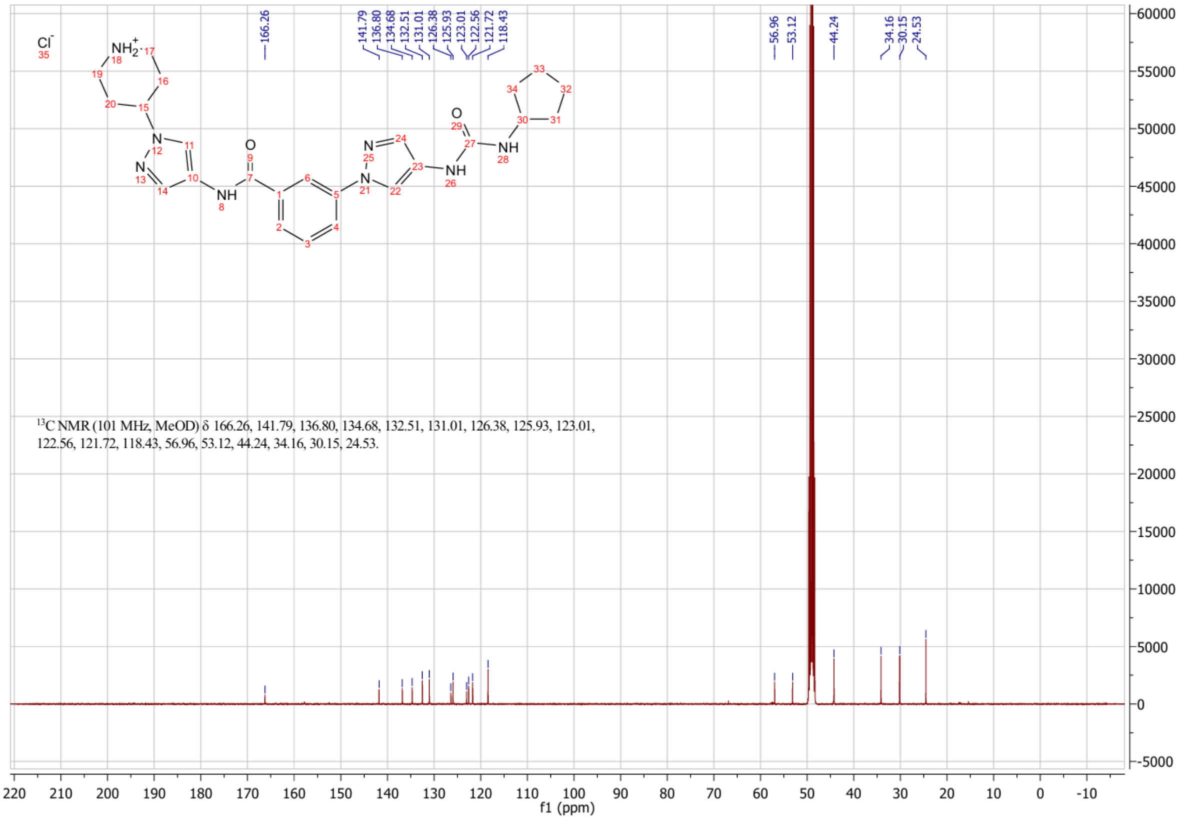

13C NMR (101 MHz, MeOD) δ 166.26, 141.79, 136.80, 134.68, 132.51, 131.01, 126.38, 125.93, 123.01, 122.56, 121.72, 118.43, 56.96, 53.12, 44.24, 34.16, 30.15, 24.53.
f1 (ppm)

1d

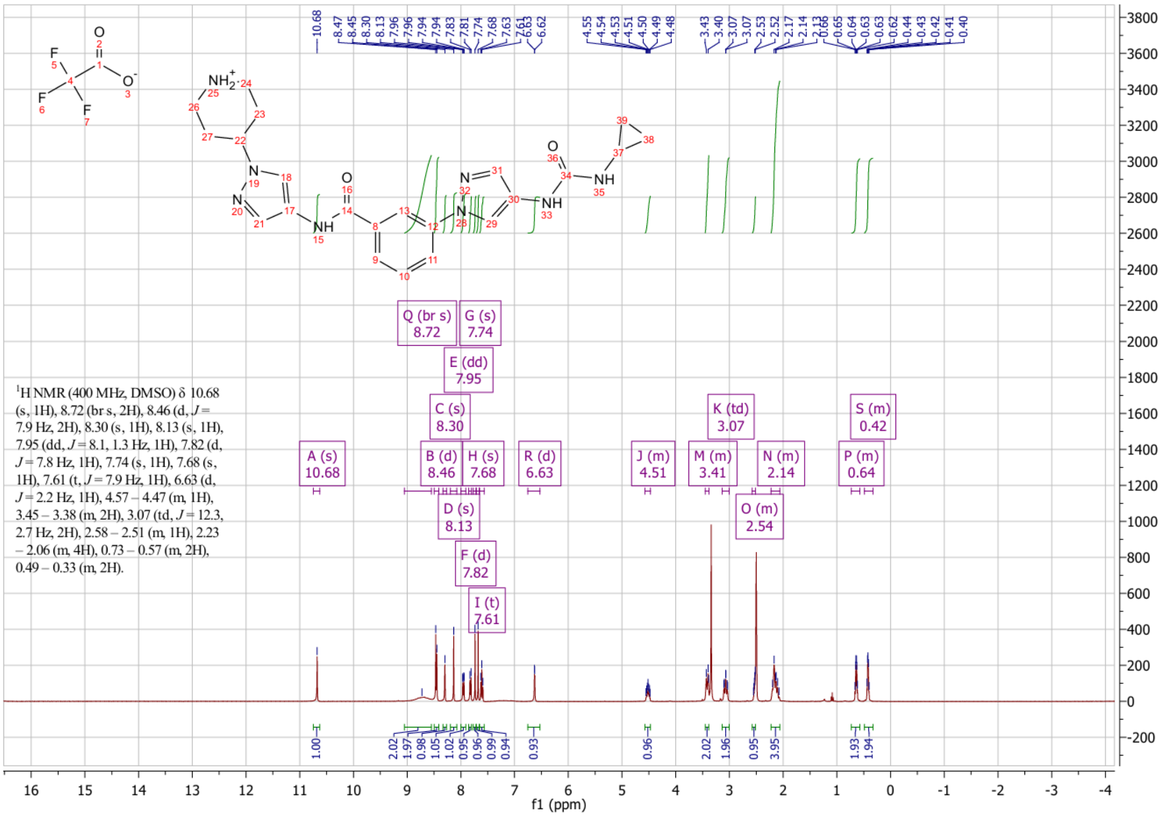
^{1}H NMR (400 MHz, DMSO) δ 10.68 (s, 1H), 8.72 (br s, 2H), 8.46 (d, J = 7.9 Hz, 2H), 8.30 (s, 1H), 8.13 (s, 1H), 7.95 (dd, J = 8.1, 1.3 Hz, 1H), 7.82 (d, J = 7.8 Hz, 1H), 7.74 (s, 1H), 7.68 (s, 1H), 7.61 (t, J = 7.9 Hz, 1H), 6.63 (d, J = 2.2 Hz, 1H), 4.57 – 4.47 (m, 1H), 3.45 – 3.38 (m, 2H), 3.07 (td, J = 12.3, 2.7 Hz, 2H), 2.58 – 2.51 (m, 1H), 2.23 – 2.06 (m, 4H), 0.73 – 0.57 (m, 2H), 0.49 – 0.33 (m, 2H).
f1 (ppm)

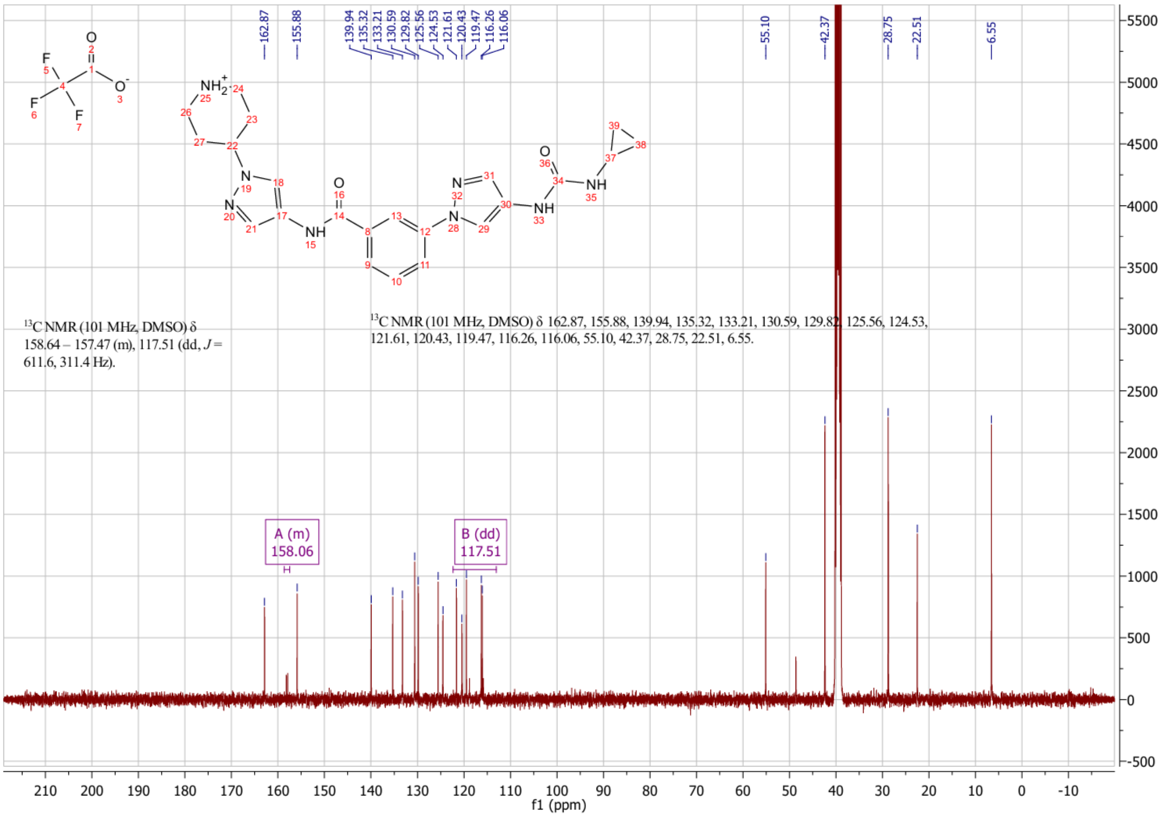
^{13}C NMR (101 MHz, DMSO) δ 158.64 – 157.47 (m), 117.51 (dd, J = 611.6, 311.4 Hz).
^{13}C NMR (101 MHz, DMSO) δ 162.87, 155.88, 139.94, 135.32, 133.21, 130.59, 129.82, 125.56, 124.53, 121.61, 120.43, 119.47, 116.26, 116.06, 55.10, 42.37, 28.75, 22.51, 6.55.
f1 (ppm)

1e

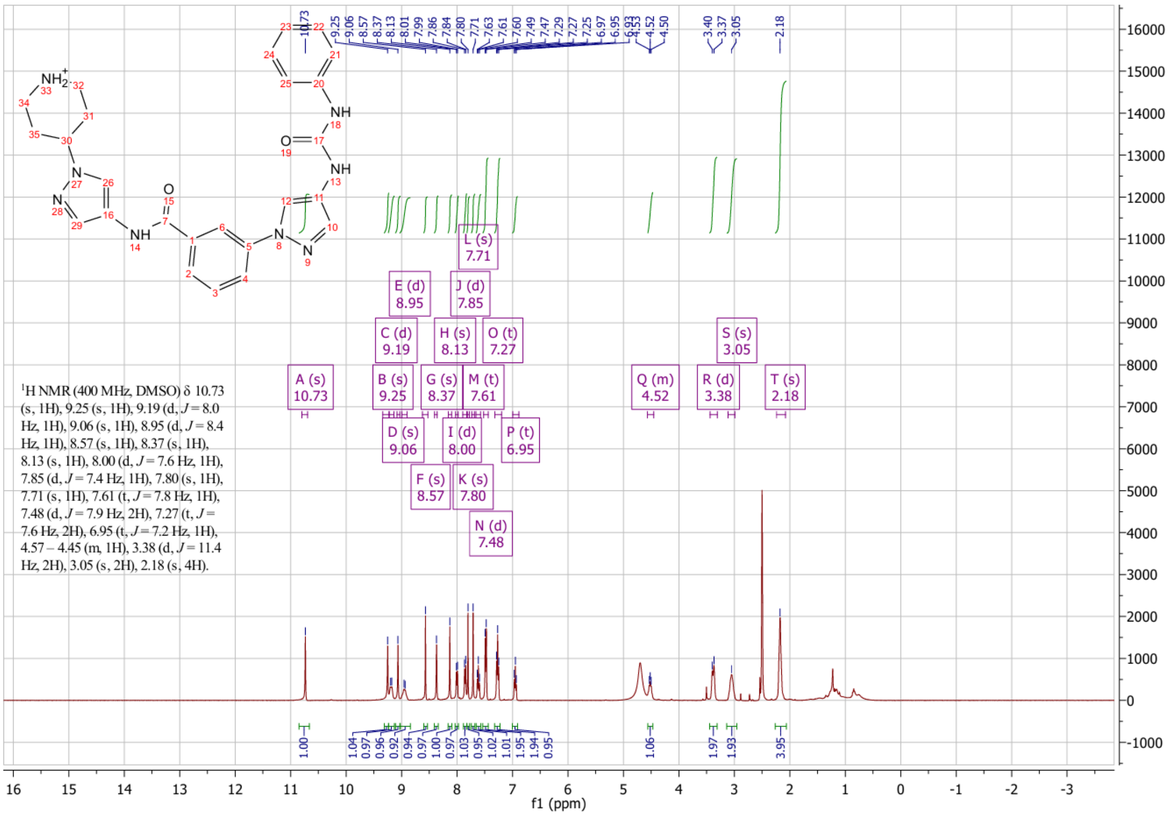

^{1}H NMR (400 MHz, DMSO) δ 10.73 (s, 1H), 9.25 (s, 1H), 9.19 (d, J = 8.0 Hz, 1H), 9.06 (s, 1H), 8.95 (d, J = 8.4 Hz, 1H), 8.57 (s, 1H), 8.37 (s, 1H), 8.13 (s, 1H), 8.00 (d, J = 7.6 Hz, 1H), 7.85 (d, J = 7.4 Hz, 1H), 7.80 (s, 1H), 7.71 (s, 1H), 7.61 (t, J = 7.8 Hz, 1H), 7.48 (d, J = 7.9 Hz, 2H), 7.27 (t, J = 7.6 Hz, 2H), 6.95 (t, J = 7.2 Hz, 1H), 4.57 – 4.45 (m, 1H), 3.38 (d, J = 11.4 Hz, 2H), 3.05 (s, 2H), 2.18 (s, 4H).
f1 (ppm)

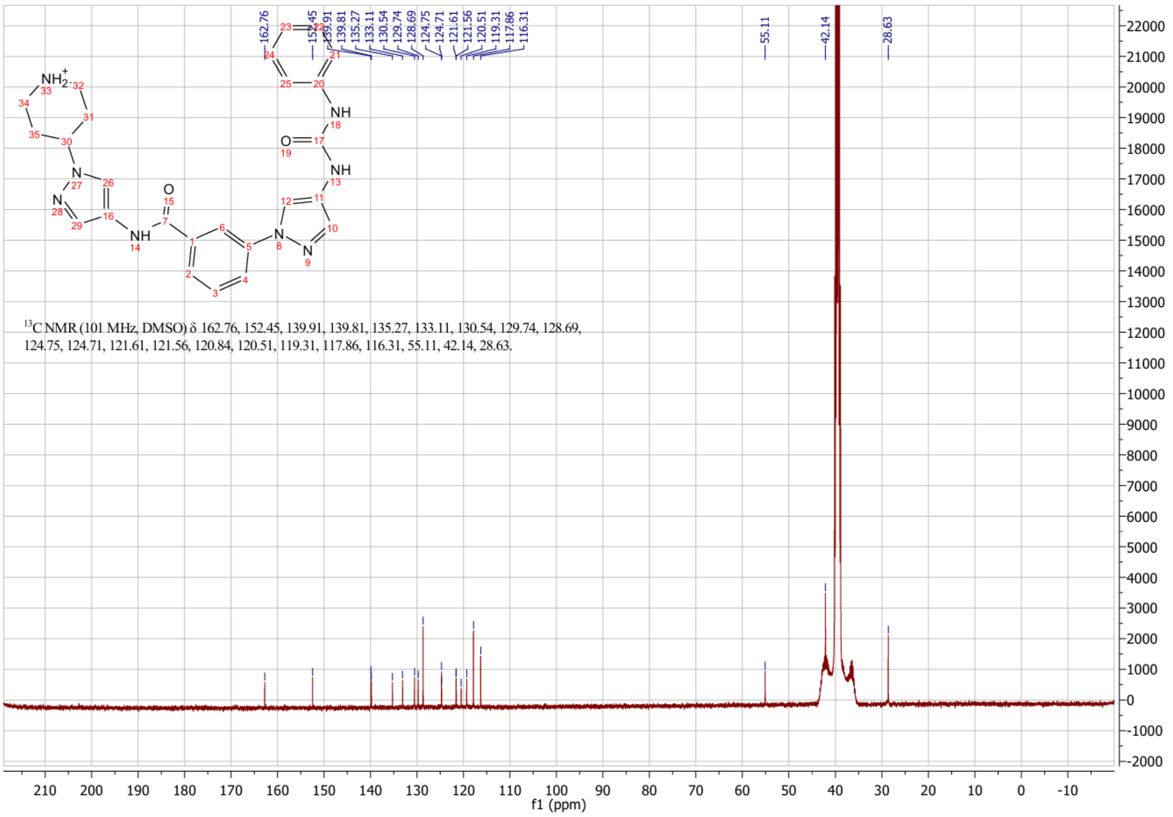

^{13}C NMR (101 MHz, DMSO) δ 162.76, 152.45, 139.91, 139.81, 135.27, 133.11, 130.54, 129.74, 128.69, 124.75, 124.71, 121.61, 121.56, 120.84, 120.51, 119.31, 117.86, 116.31, 55.11, 42.14, 28.63.
f1 (ppm)

1f

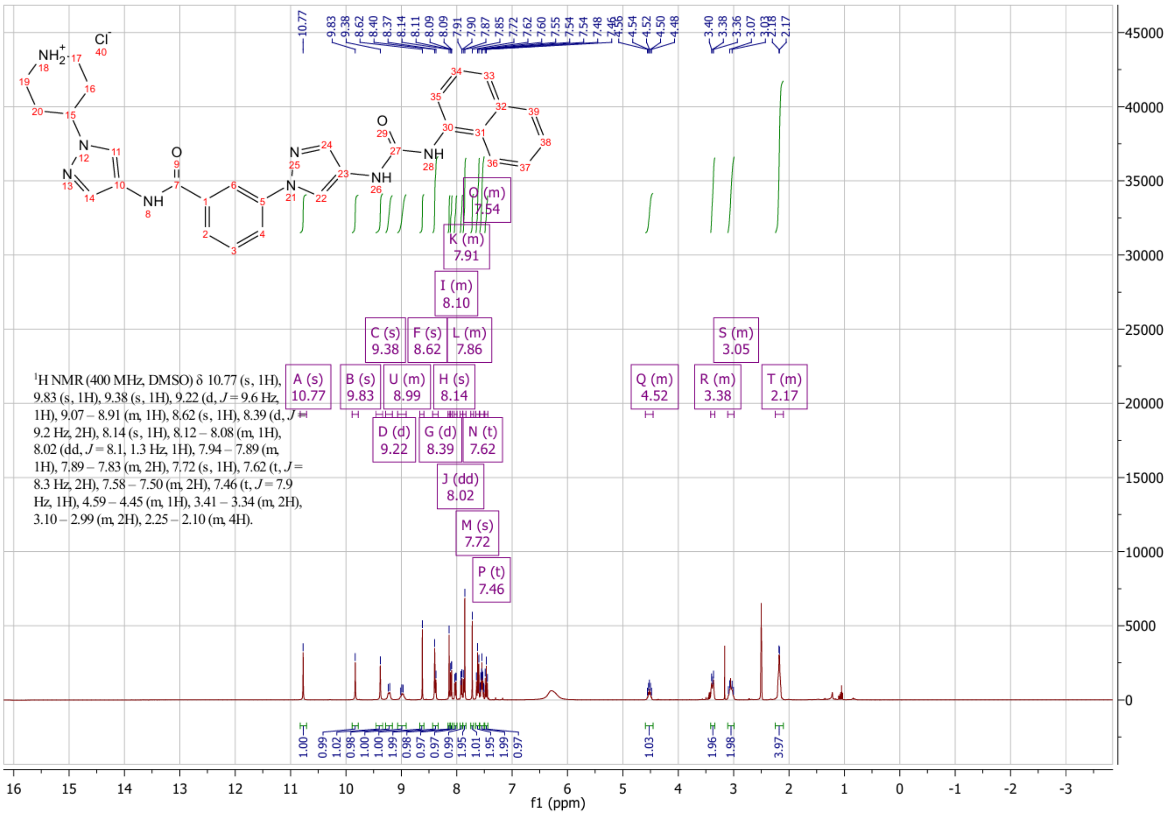
1H NMR (400 MHz, DMSO) δ 10.77 (s, 1H), 9.83 (s, 1H), 9.38 (s, 1H), 9.22 (d, J = 9.6 Hz, 1H), 9.07 – 8.91 (m, 1H), 8.62 (s, 1H), 8.39 (d, J = 9.2 Hz, 2H), 8.14 (s, 1H), 8.12 – 8.08 (m, 1H), 8.02 (dd, J = 8.1, 1.3 Hz, 1H), 7.94 – 7.89 (m, 1H), 7.89 – 7.83 (m, 2H), 7.72 (s, 1H), 7.62 (t, J = 8.3 Hz, 2H), 7.58 – 7.50 (m, 2H), 7.46 (t, J = 7.9 Hz, 1H), 4.59 – 4.45 (m, 1H), 3.41 – 3.34 (m, 2H), 3.10 – 2.99 (m, 2H), 2.25 – 2.10 (m, 4H).
f1 (ppm)

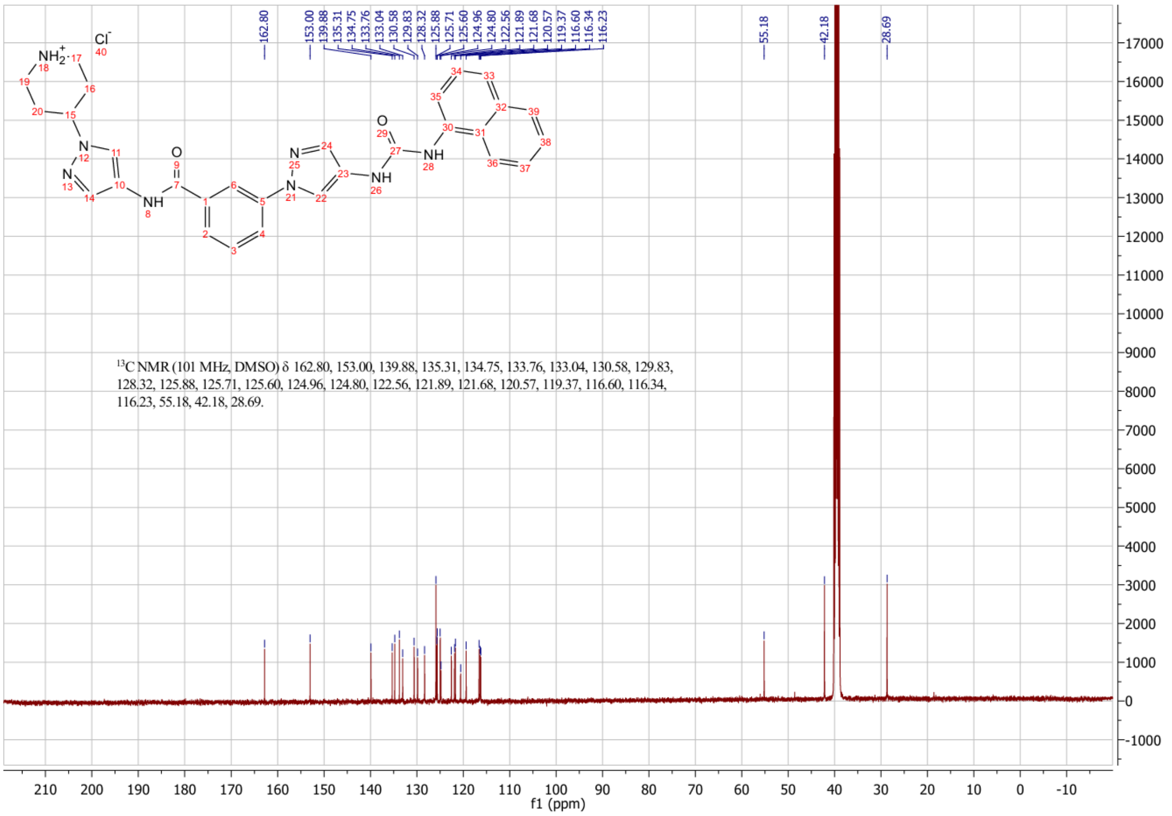
13C NMR (101 MHz, DMSO) δ 162.80, 153.00, 139.88, 135.31, 134.75, 133.76, 133.04, 130.58, 129.83, 128.32, 125.88, 125.71, 125.60, 124.96, 124.80, 122.56, 121.89, 121.68, 120.57, 119.37, 116.60, 116.34, 116.23, 55.18, 42.18, 28.69.
f1 (ppm)

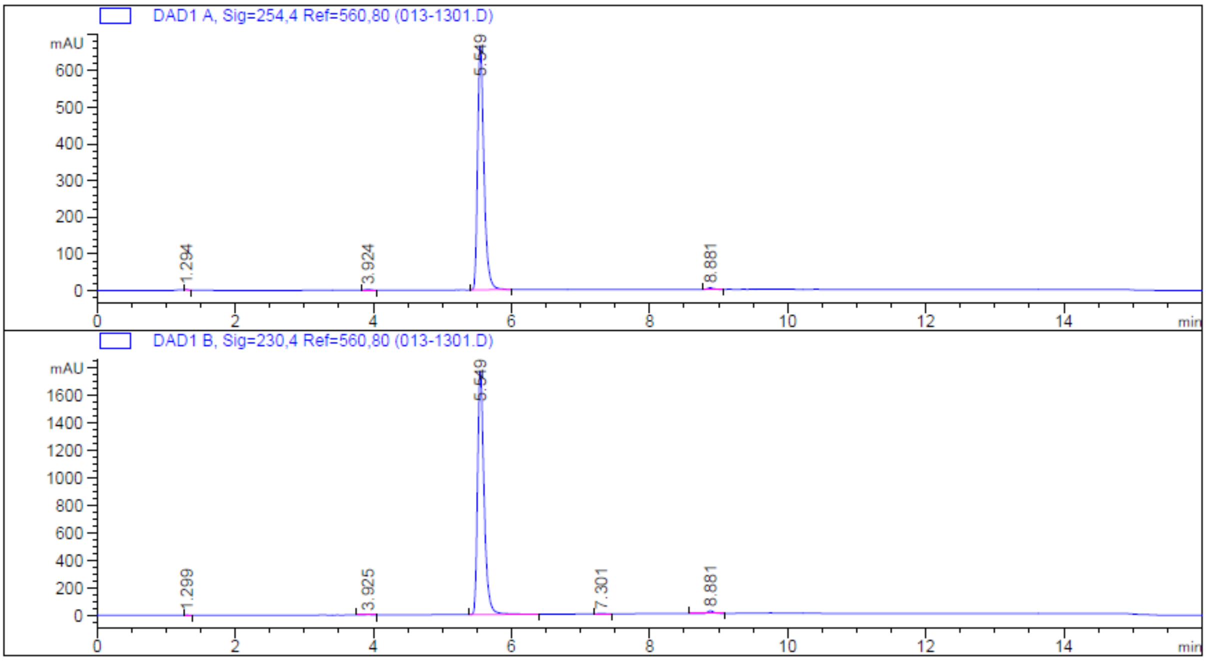

===

Area Percent Report

===

Sorted By : Signal
Multiplier: : 1.0000
Dilution: : 1.0000
Use Multiplier & Dilution Factor with ISTDs

Signal 1: DAD1 A, Sig=254,4 Ref=560,80

Peak #	RetTime [min]	Type	Width [min]	Area [mAU*s]	Height [mAU]	Area %
1	1.294	BB	0.0403	4.17233	1.71951	0.0970
2	3.924	BB	0.0788	8.29754	1.64722	0.1930
3	5.549	BB	0.0964	4262.88574	668.16772	99.1525
4	8.881	BB	0.0885	23.96698	4.20456	0.5575

Totals : 4299.32259 675.73902

Signal 2: DAD1 B, Sig=230,4 Ref=560,80

Peak #	RetTime [min]	Type	Width [min]	Area [mAU*s]	Height [mAU]	Area %
1	1.299	BB	0.0440	5.62630	2.05086	0.0481
2	3.925	BB	0.0865	12.37041	2.17045	0.1057
3	5.549	BB	0.1000	1.15563e4	1773.16809	98.7831
4	7.301	BB	0.0858	18.73091	3.42415	0.1601
5	8.881	BB	0.0902	105.62920	18.07238	0.9029

Totals : 1.16987e4 1798.88593

===

*** End of Report ***

1g

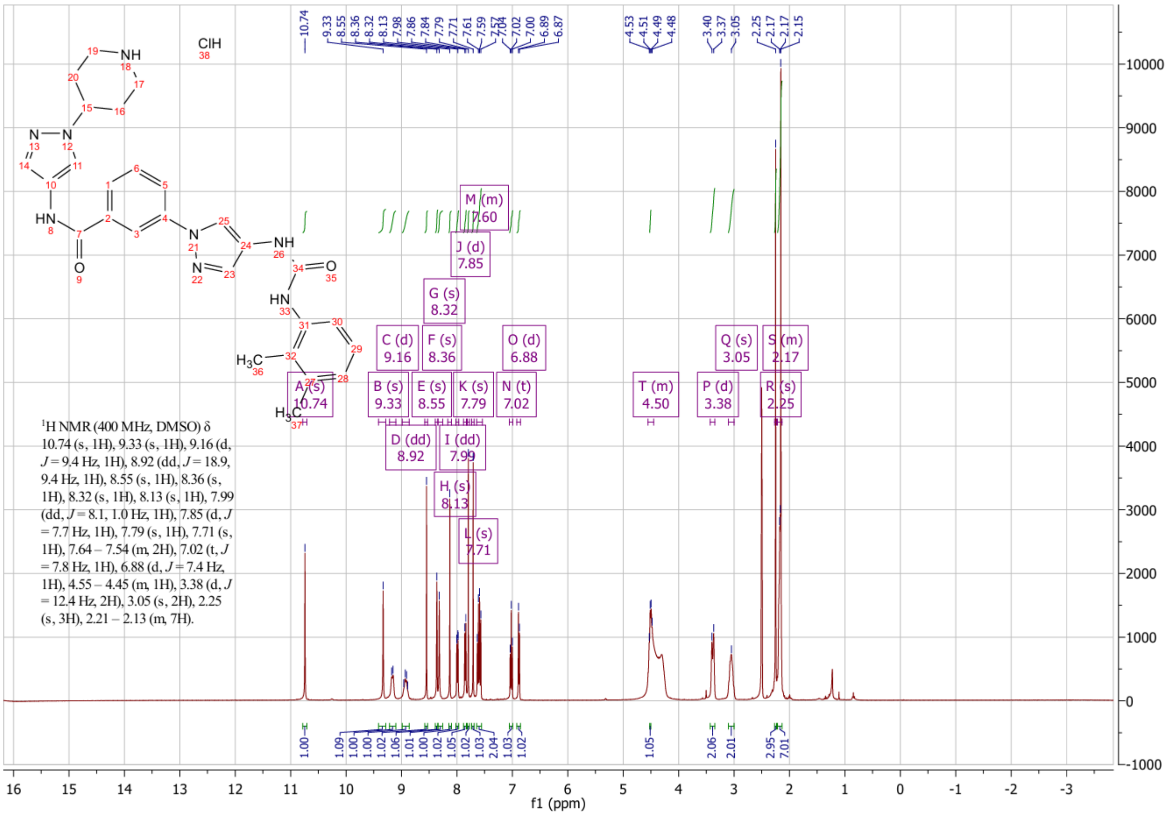

^{1}H NMR (400 MHz, DMSO) δ 10.74 (s, 1H), 9.33 (s, 1H), 9.16 (d, J = 9.4 Hz, 1H), 8.92 (dd, J = 18.9, 9.4 Hz, 1H), 8.55 (s, 1H), 8.36 (s, 1H), 8.32 (s, 1H), 8.13 (s, 1H), 7.99 (dd, J = 8.1, 1.0 Hz, 1H), 7.85 (d, J = 7.7 Hz, 1H), 7.79 (s, 1H), 7.71 (s, 1H), 7.64 – 7.54 (m, 2H), 7.02 (t, J = 7.8 Hz, 1H), 6.88 (d, J = 7.4 Hz, 1H), 4.55 – 4.45 (m, 1H), 3.38 (d, J = 12.4 Hz, 2H), 3.05 (s, 2H), 2.25 (s, 3H), 2.21 – 2.13 (m, 7H).
f1 (ppm)

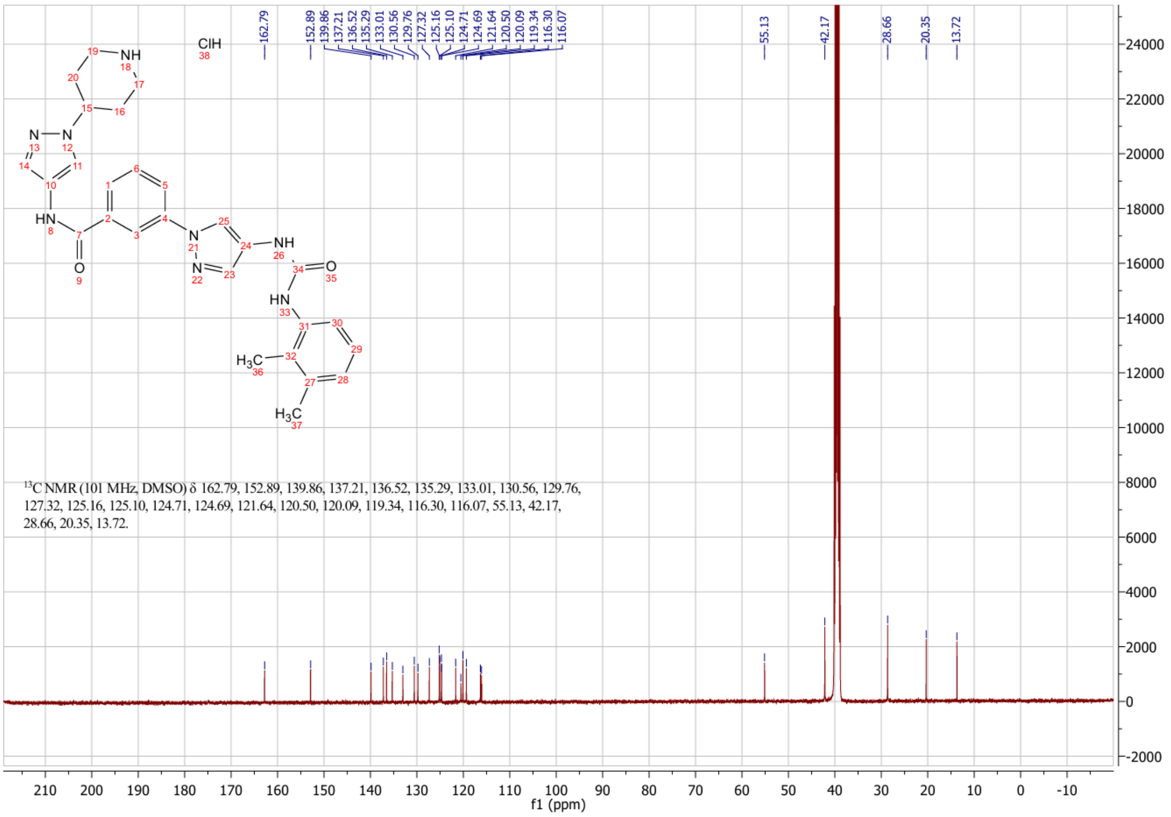

^{13}C NMR (101 MHz, DMSO) δ 162.79, 152.89, 139.86, 137.21, 136.52, 135.29, 133.01, 130.56, 129.76, 127.32, 125.16, 125.10, 124.71, 124.69, 121.64, 120.50, 120.09, 119.34, 116.30, 116.07, 55.13, 42.17, 28.66, 20.35, 13.72.
f1 (ppm)

1h

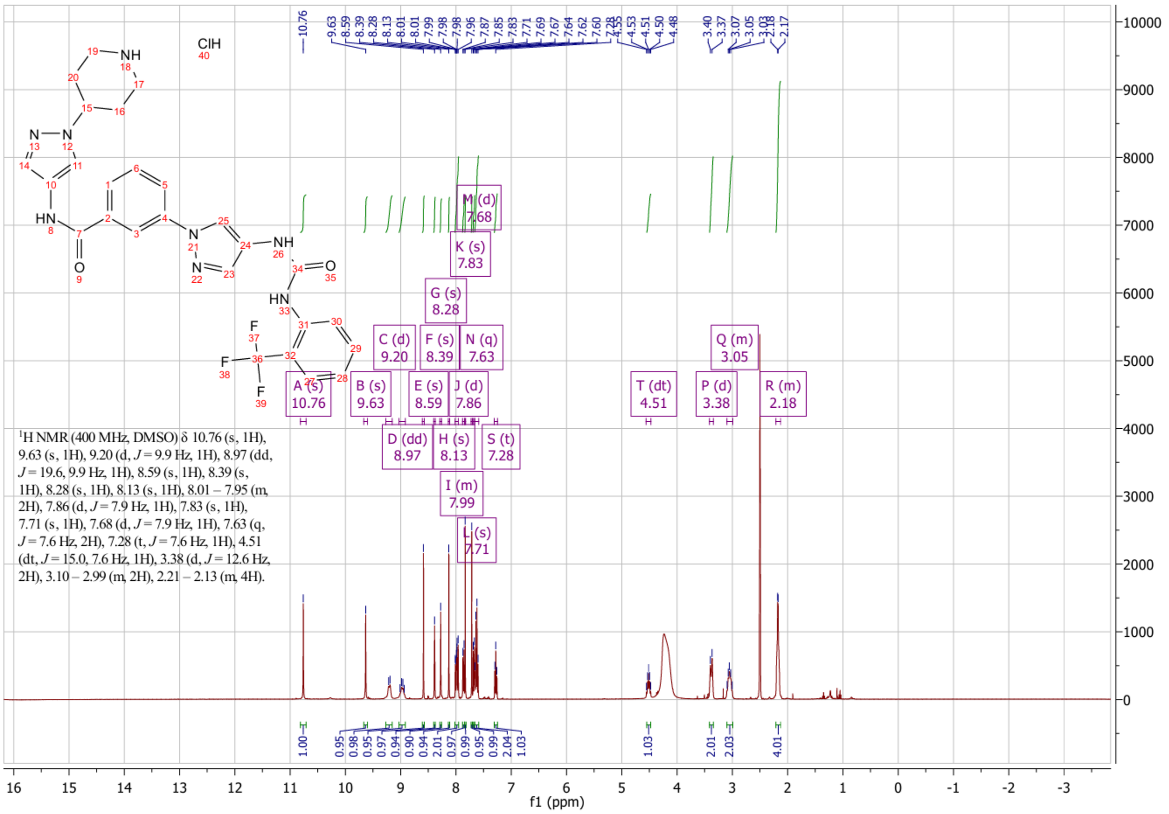
ClH
1H NMR (400 MHz, DMSO) δ 10.76 (s, 1H), 9.63 (s, 1H), 9.20 (d, J = 9.9 Hz, 1H), 8.97 (dd, J = 19.6, 9.9 Hz, 1H), 8.59 (s, 1H), 8.39 (s, 1H), 8.28 (s, 1H), 8.13 (s, 1H), 8.01 – 7.95 (m, 2H), 7.86 (d, J = 7.9 Hz, 1H), 7.83 (s, 1H), 7.71 (s, 1H), 7.68 (d, J = 7.9 Hz, 1H), 7.63 (q, J = 7.6 Hz, 2H), 7.28 (t, J = 7.6 Hz, 1H), 4.51 (dt, J = 15.0, 7.6 Hz, 1H), 3.38 (d, J = 12.6 Hz, 2H), 3.10 – 2.99 (m, 2H), 2.21 – 2.13 (m, 4H).
f1 (ppm)

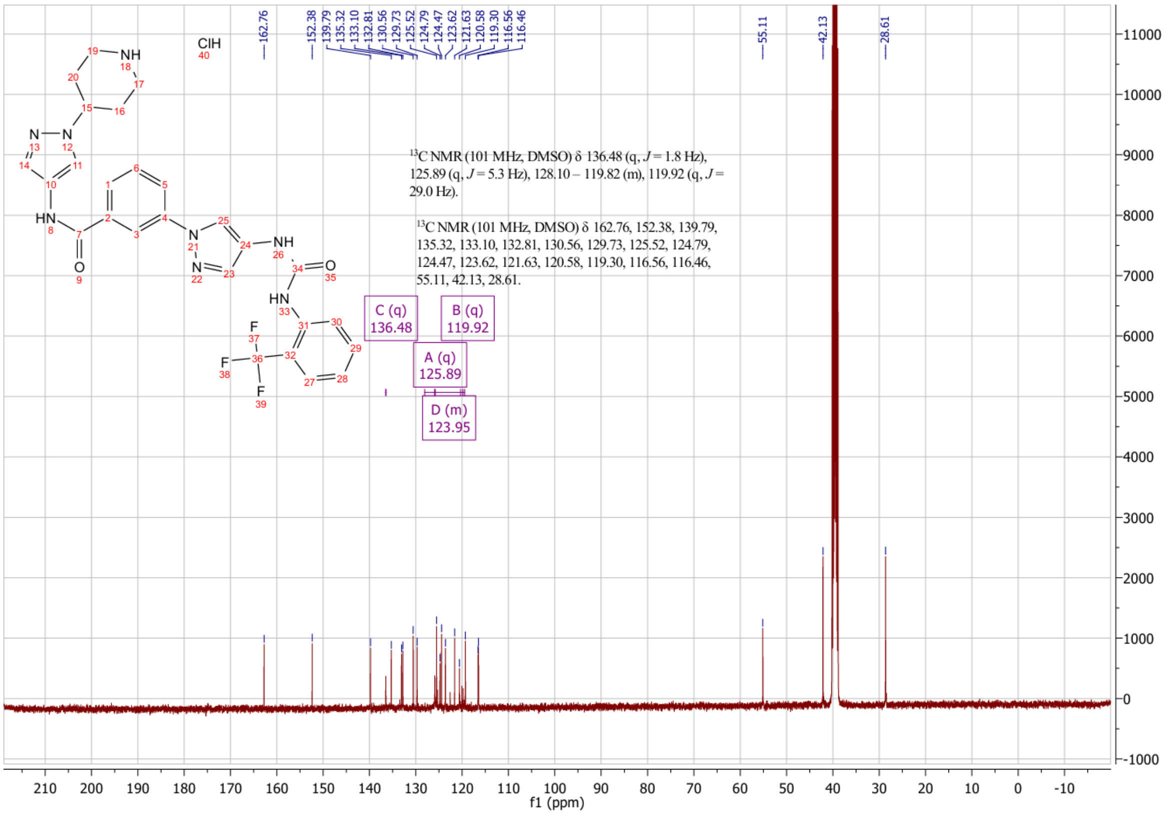
ClH
13C NMR (101 MHz, DMSO) δ 136.48 (q, J = 1.8 Hz), 125.89 (q, J = 5.3 Hz), 128.10 – 119.82 (m), 119.92 (q, J = 29.0 Hz).
13C NMR (101 MHz, DMSO) δ 162.76, 152.38, 139.79, 135.32, 133.10, 132.81, 130.56, 129.73, 125.52, 124.79, 124.47, 123.62, 121.63, 120.58, 119.30, 116.56, 116.46, 55.11, 42.13, 28.61.
f1 (ppm)

1i

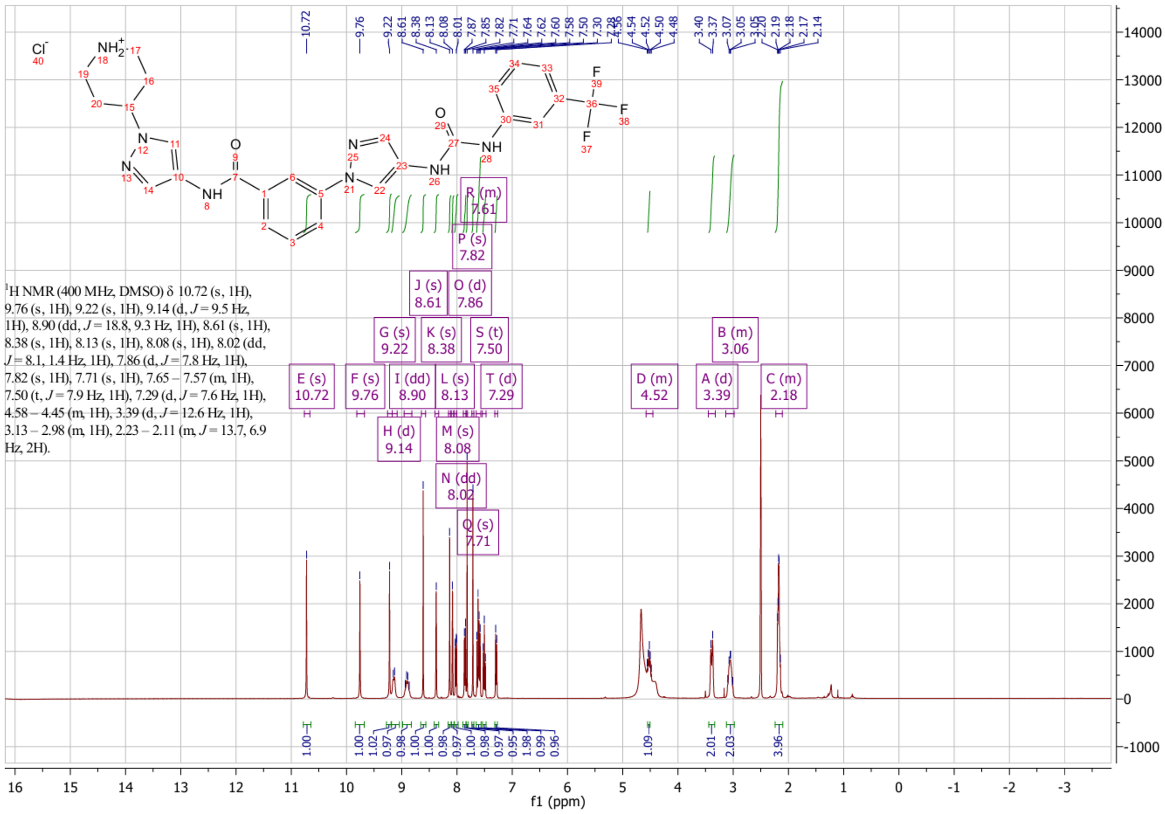

^{1}H NMR (400 MHz, DMSO) δ 10.72 (s, 1H), 9.76 (s, 1H), 9.22 (s, 1H), 9.14 (d, J = 9.5 Hz, 1H), 8.90 (dd, J = 18.8, 9.3 Hz, 1H), 8.61 (s, 1H), 8.38 (s, 1H), 8.13 (s, 1H), 8.08 (s, 1H), 8.02 (dd, J = 8.1, 1.4 Hz, 1H), 7.86 (d, J = 7.8 Hz, 1H), 7.82 (s, 1H), 7.71 (s, 1H), 7.65 – 7.57 (m, 1H), 7.50 (t, J = 7.9 Hz, 1H), 7.29 (d, J = 7.6 Hz, 1H), 4.58 – 4.45 (m, 1H), 3.39 (d, J = 12.6 Hz, 1H), 3.13 – 2.98 (m, 1H), 2.23 – 2.11 (m, J = 13.7, 6.9 Hz, 2H).

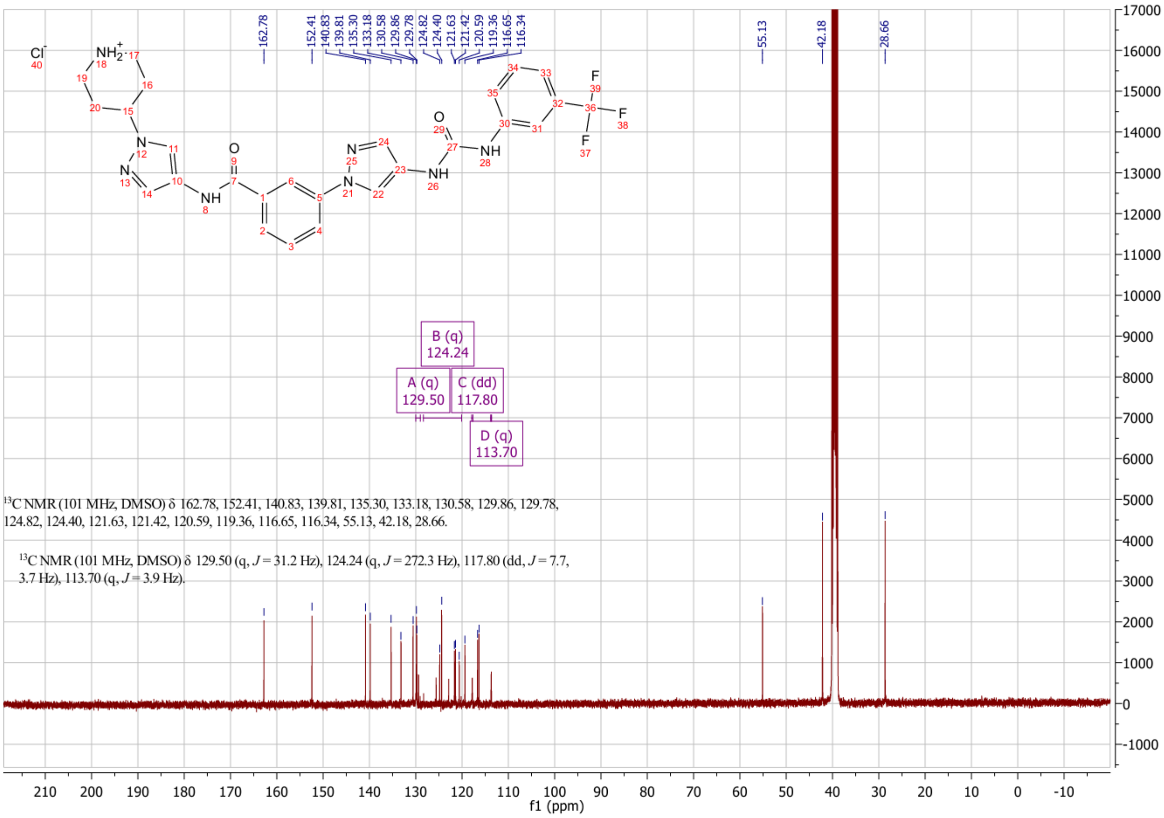

^{13}C NMR (101 MHz, DMSO) δ 162.78, 152.41, 140.83, 139.81, 135.30, 133.18, 130.58, 129.86, 129.78, 124.82, 124.40, 121.63, 121.42, 120.59, 119.36, 116.65, 116.34, 55.13, 42.18, 28.66.
^{13}C NMR (101 MHz, DMSO) δ 129.50 (q, J = 31.2 Hz), 124.24 (q, J = 272.3 Hz), 117.80 (dd, J = 7.7, 3.7 Hz), 113.70 (q, J = 3.9 Hz).

1j

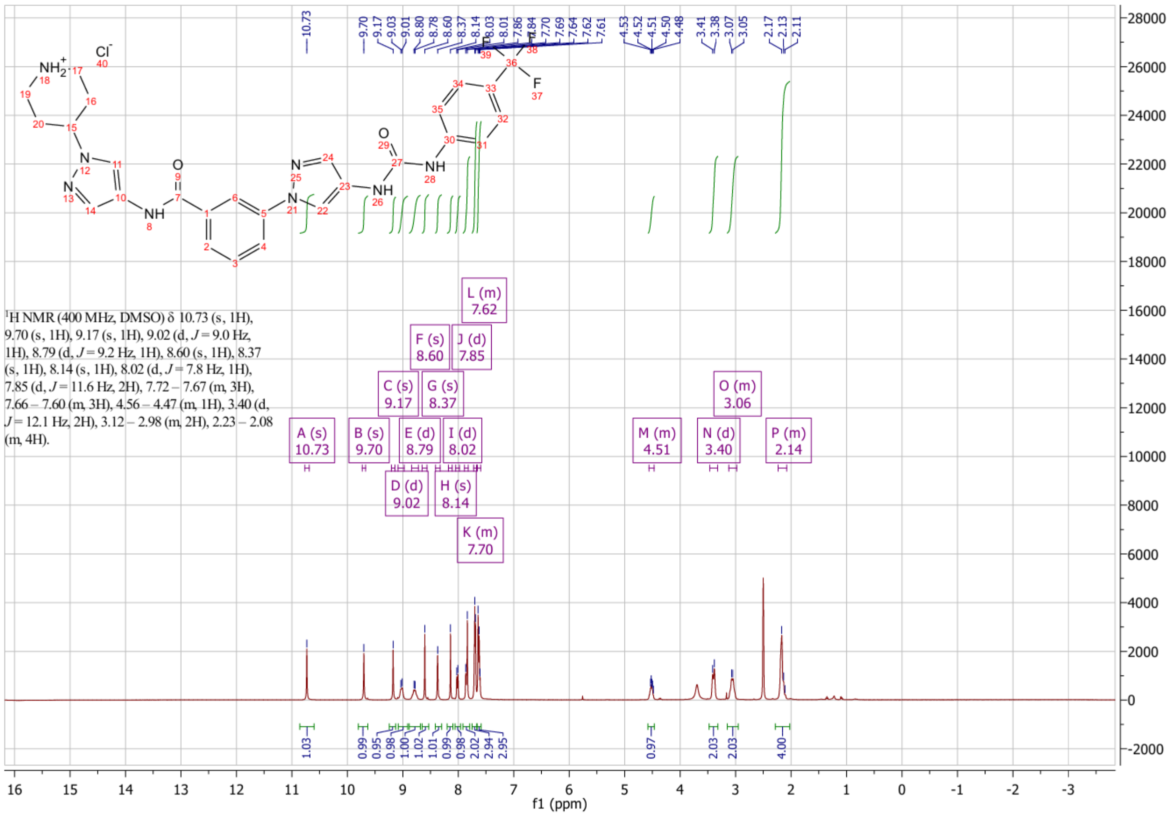

1H NMR (400 MHz, DMSO) δ 10.73 (s, 1H), 9.70 (s, 1H), 9.17 (s, 1H), 9.02 (d, J = 9.0 Hz, 1H), 8.79 (d, J = 9.2 Hz, 1H), 8.60 (s, 1H), 8.37 (s, 1H), 8.14 (s, 1H), 8.02 (d, J = 7.8 Hz, 1H), 7.85 (d, J = 11.6 Hz, 2H), 7.72 – 7.67 (m, 3H), 7.66 – 7.60 (m, 3H), 4.56 – 4.47 (m, 1H), 3.40 (d, J = 12.1 Hz, 2H), 3.12 – 2.98 (m, 2H), 2.23 – 2.08 (m, 4H).
f1 (ppm)

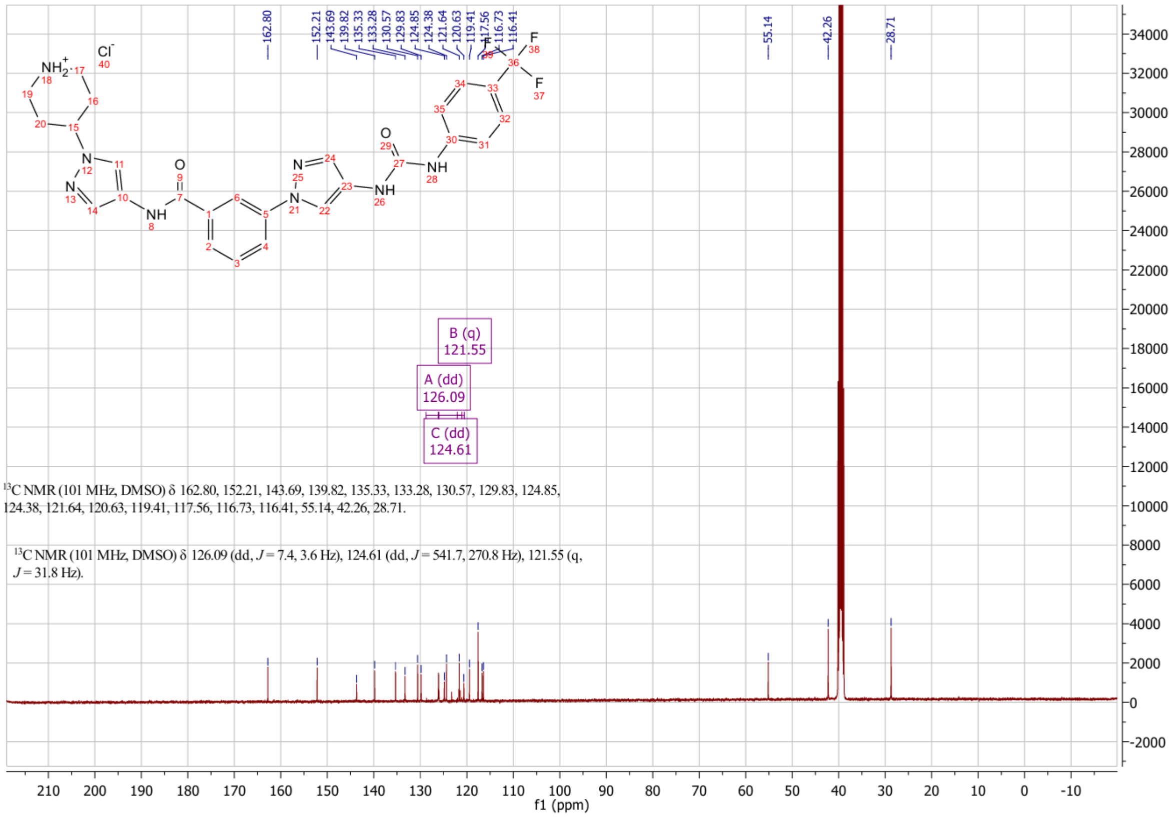

13C NMR (101 MHz, DMSO) δ 162.80, 152.21, 143.69, 139.82, 135.33, 133.28, 130.57, 129.83, 124.85, 124.38, 121.64, 120.63, 119.41, 117.56, 116.73, 116.41, 55.14, 42.26, 28.71.
13C NMR (101 MHz, DMSO) δ 126.09 (dd, J = 7.4, 3.6 Hz), 124.61 (dd, J = 541.7, 270.8 Hz), 121.55 (q, J = 31.8 Hz).
f1 (ppm)

11.2 Table 2

16a

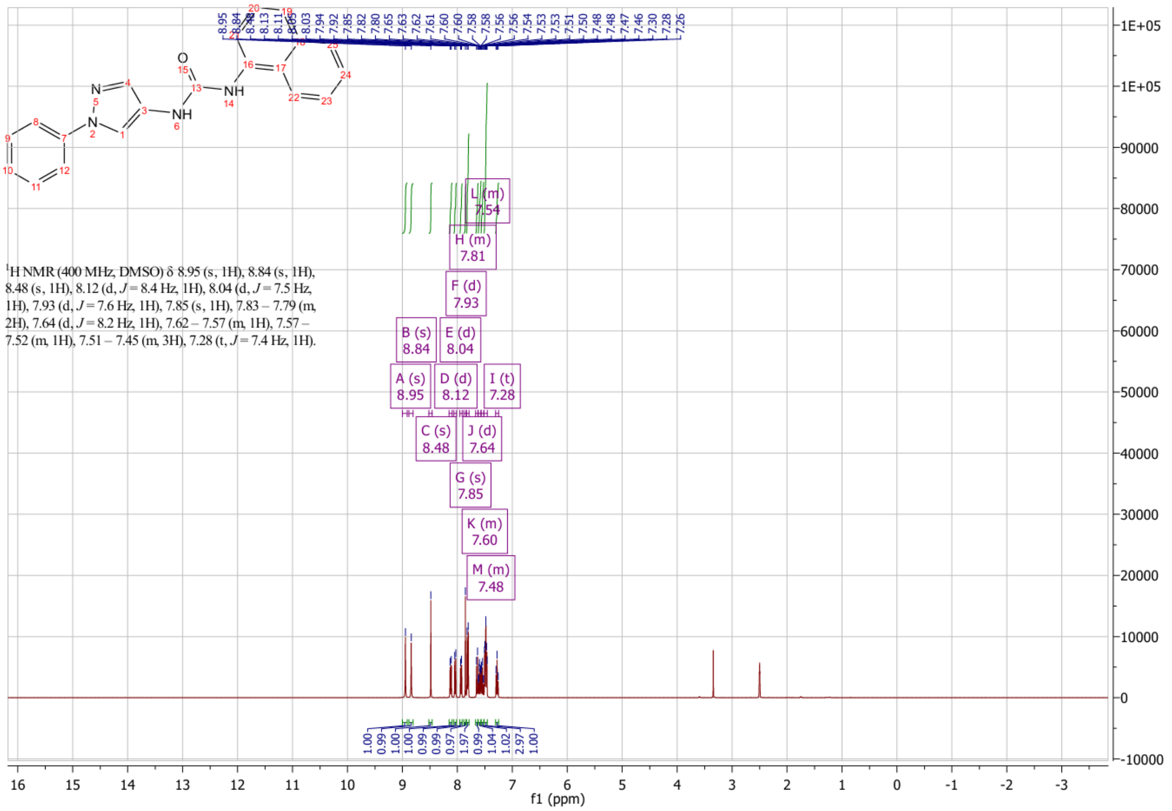

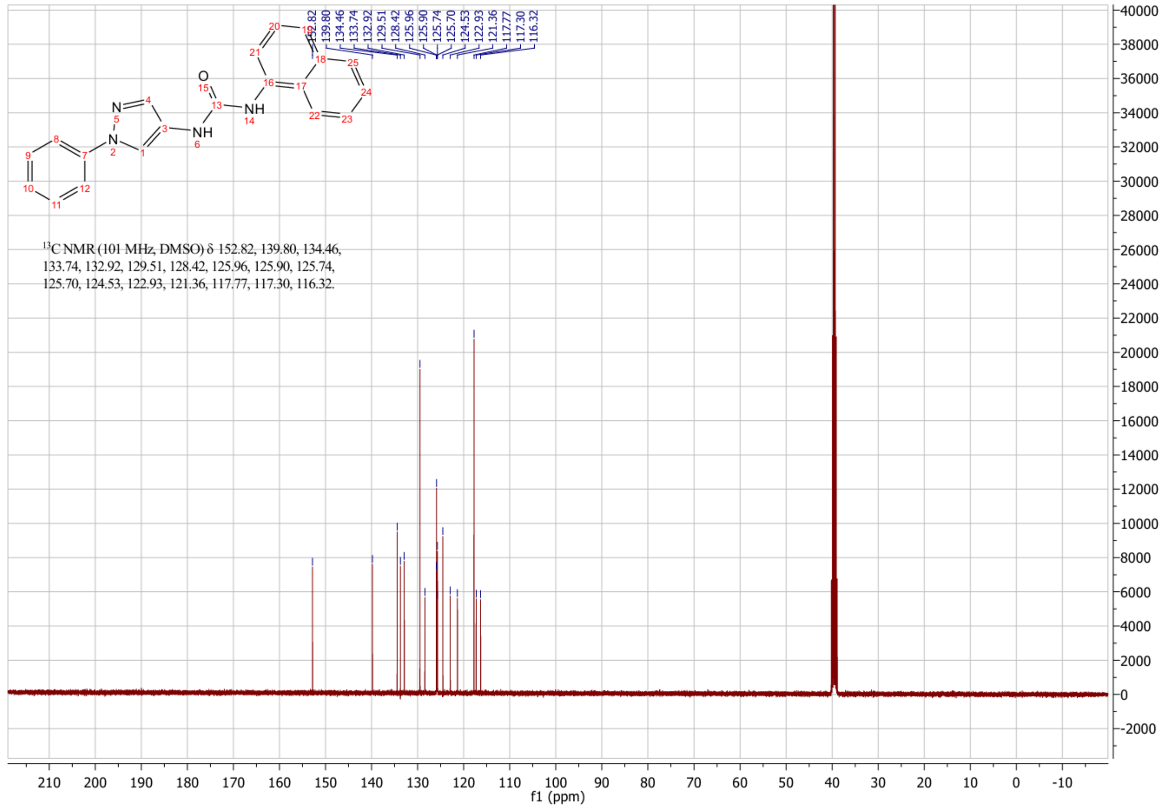

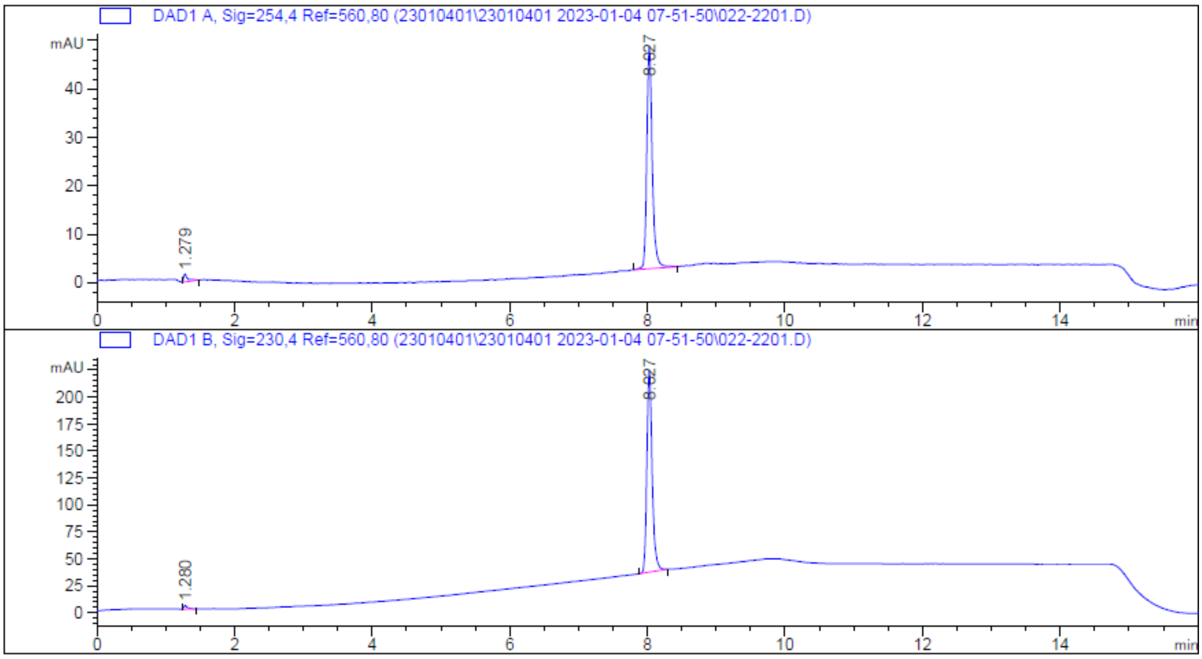

===

Area Percent Report

===

Sorted By : Signal
Multiplier: : 1.0000
Dilution: : 1.0000
Use Multiplier & Dilution Factor with ISTDs

Signal 1: DAD1 A, Sig=254,4 Ref=560,80

Peak #	RetTime [min]	Type	Width [min]	Area [mAU*s]	Height [mAU]	Area %
1	1.279	BB	0.0645	7.47630	1.66076	2.8784
2	8.027	BB	0.0820	252.26077	45.97860	97.1216

Totals : 259.73707 47.63936

Signal 2: DAD1 B, Sig=230,4 Ref=560,80

Peak #	RetTime [min]	Type	Width [min]	Area [mAU*s]	Height [mAU]	Area %
1	1.280	BB	0.0568	12.71957	3.31402	1.2580
2	8.027	BB	0.0801	998.36566	187.49263	98.7420

Totals : 1011.08524 190.80665

===

*** End of Report ***

16b

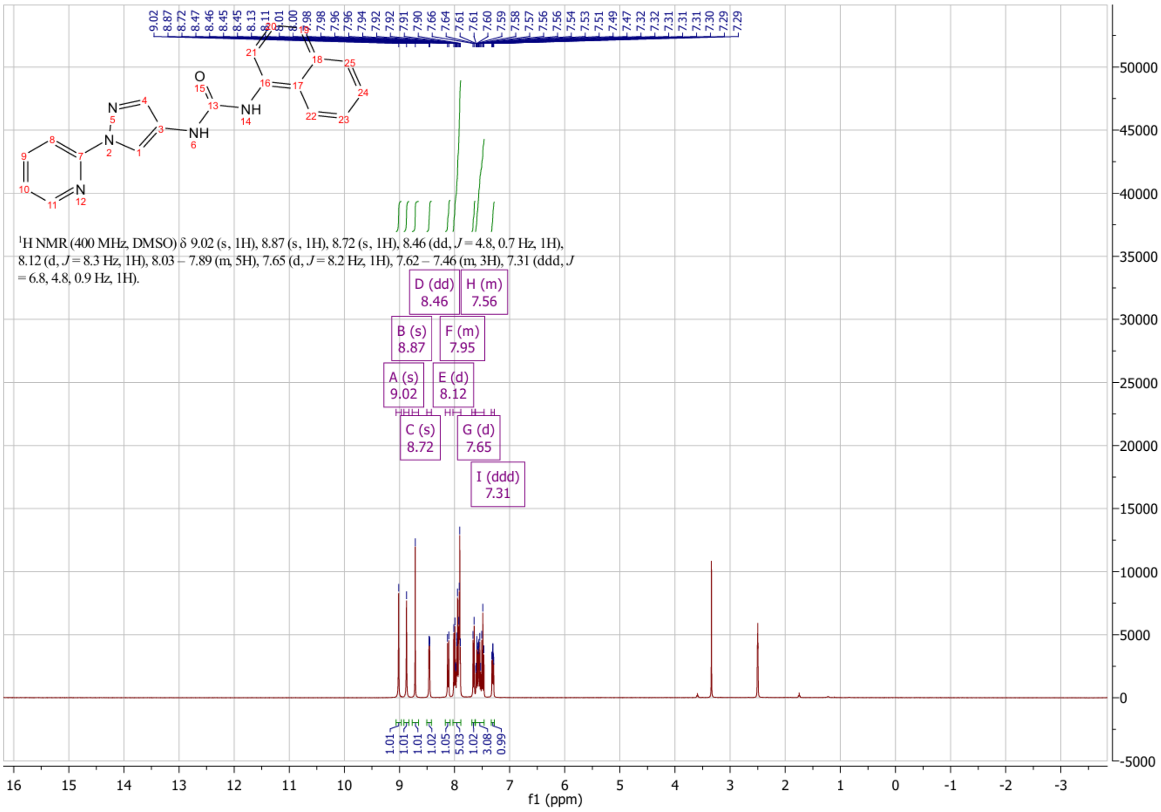

^{1}H NMR (400 MHz, DMSO) δ 9.02 (s, 1H), 8.87 (s, 1H), 8.72 (s, 1H), 8.46 (dd, J = 4.8, 0.7 Hz, 1H), 8.12 (d, J = 8.3 Hz, 1H), 8.03 – 7.89 (m, 5H), 7.65 (d, J = 8.2 Hz, 1H), 7.62 – 7.46 (m, 3H), 7.31 (ddd, J = 6.8, 4.8, 0.9 Hz, 1H).
f1 (ppm)

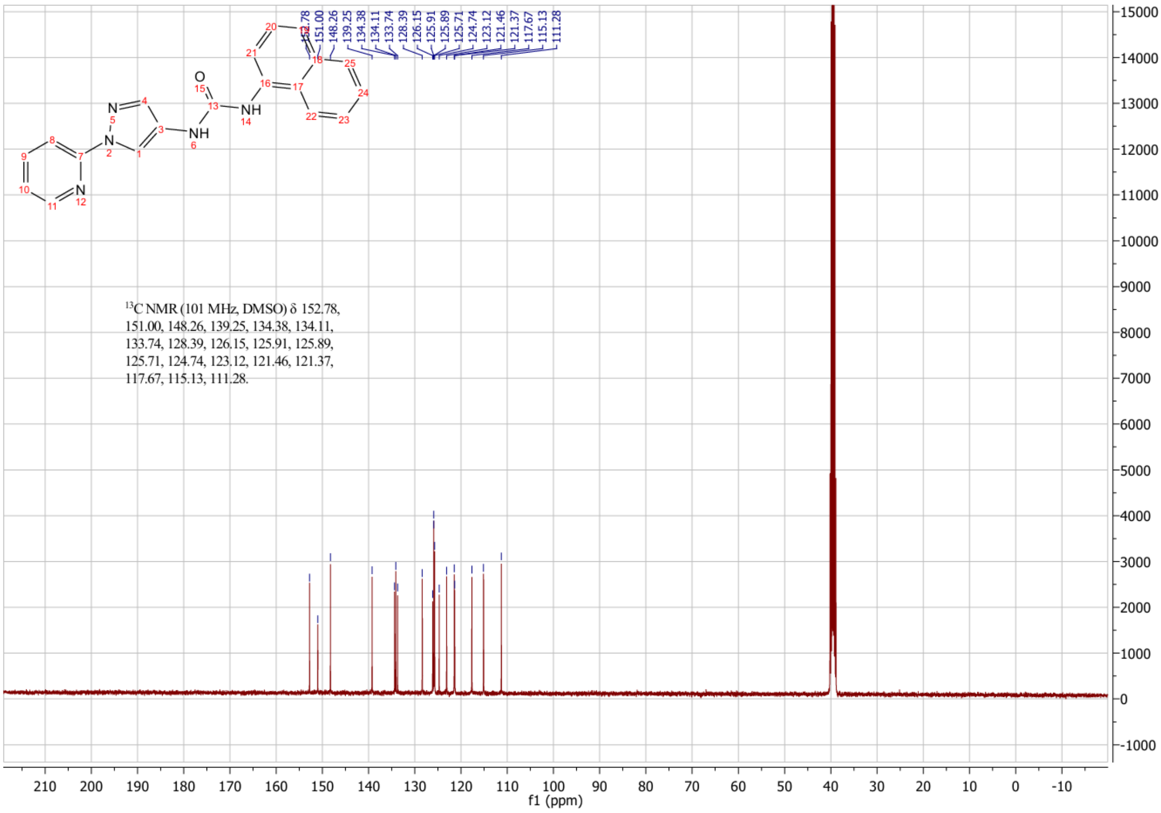

^{13}C NMR (101 MHz, DMSO) δ 152.78, 151.00, 148.26, 139.25, 134.38, 134.11, 133.74, 128.39, 126.15, 125.91, 125.89, 125.71, 124.74, 123.12, 121.46, 121.37, 117.67, 115.13, 111.28.
f1 (ppm)

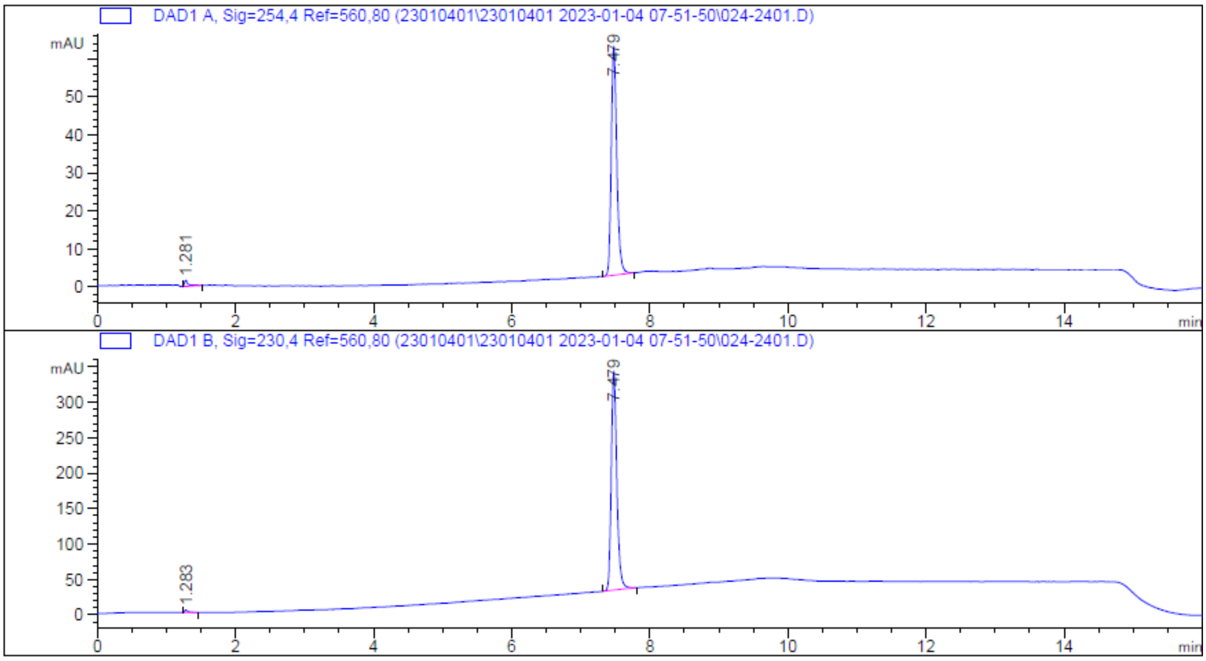

Area Percent Report

Sorted By : Signal
Multiplier: : 1.0000
Dilution: : 1.0000
Use Multiplier & Dilution Factor with ISTDs

Signal 1: DAD1 A, Sig=254,4 Ref=560,80

Peak #	RetTime [min]	Type	Width [min]	Area [mAU*s]	Height [mAU]	Area %
1	1.281	BB	0.0598	6.57704	1.60525	1.9218
2	7.479	BB	0.0848	335.65988	60.39323	98.0782

Totals : 342.23693 61.99848

Signal 2: DAD1 B, Sig=230,4 Ref=560,80

Peak #	RetTime [min]	Type	Width [min]	Area [mAU*s]	Height [mAU]	Area %
1	1.283	BB	0.0555	12.40468	3.32691	0.7154
2	7.479	BB	0.0850	1721.60657	308.98856	99.2846

Totals : 1734.01125 312.31546

*** End of Report ***

16c

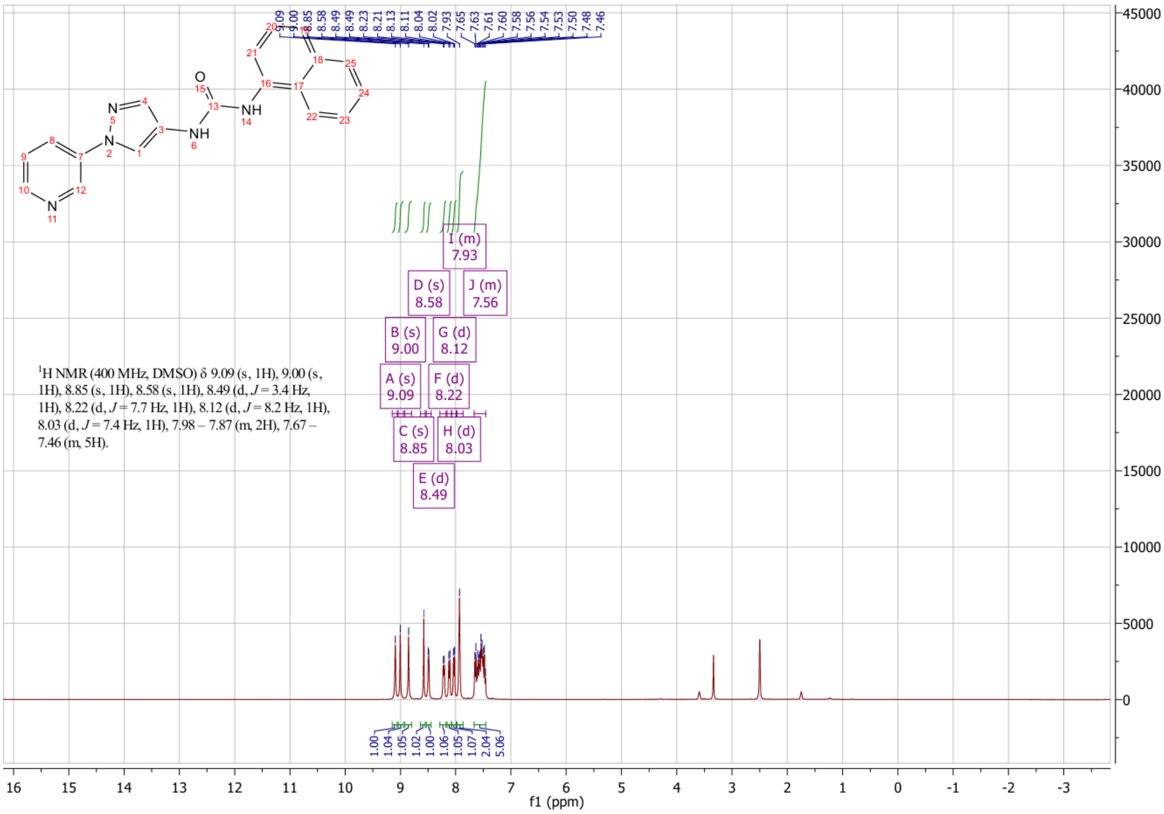
^{1}H NMR (400 MHz, DMSO) δ 9.09 (s, 1H), 9.00 (s, 1H), 8.85 (s, 1H), 8.58 (s, 1H), 8.49 (d, J = 3.4 Hz, 1H), 8.22 (d, J = 7.7 Hz, 1H), 8.12 (d, J = 8.2 Hz, 1H), 8.03 (d, J = 7.4 Hz, 1H), 7.98 – 7.87 (m, 2H), 7.67 – 7.46 (m, 5H).
f1 (ppm)

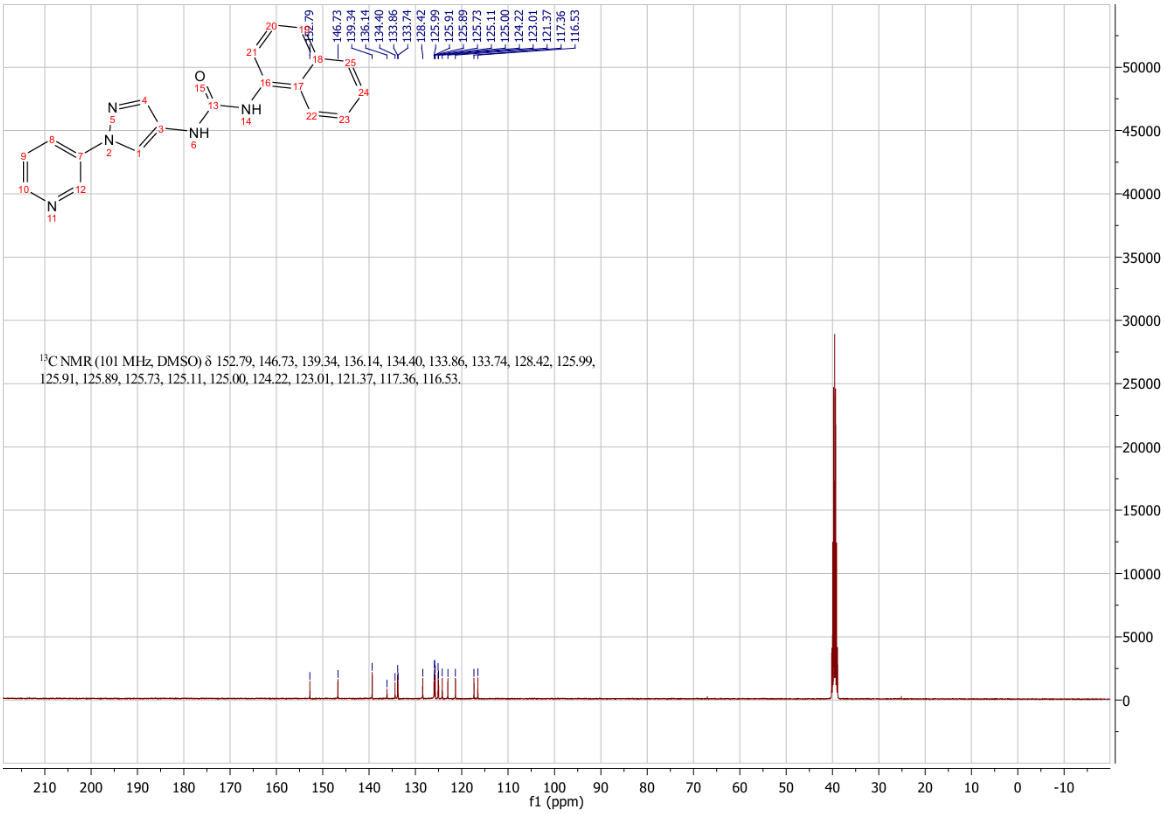
^{13}C NMR (101 MHz, DMSO) δ 152.79, 146.73, 139.34, 136.14, 134.40, 133.86, 133.74, 128.42, 125.99, 125.91, 125.89, 125.73, 125.11, 125.00, 124.22, 123.01, 121.37, 117.36, 116.53.
f1 (ppm)

16d

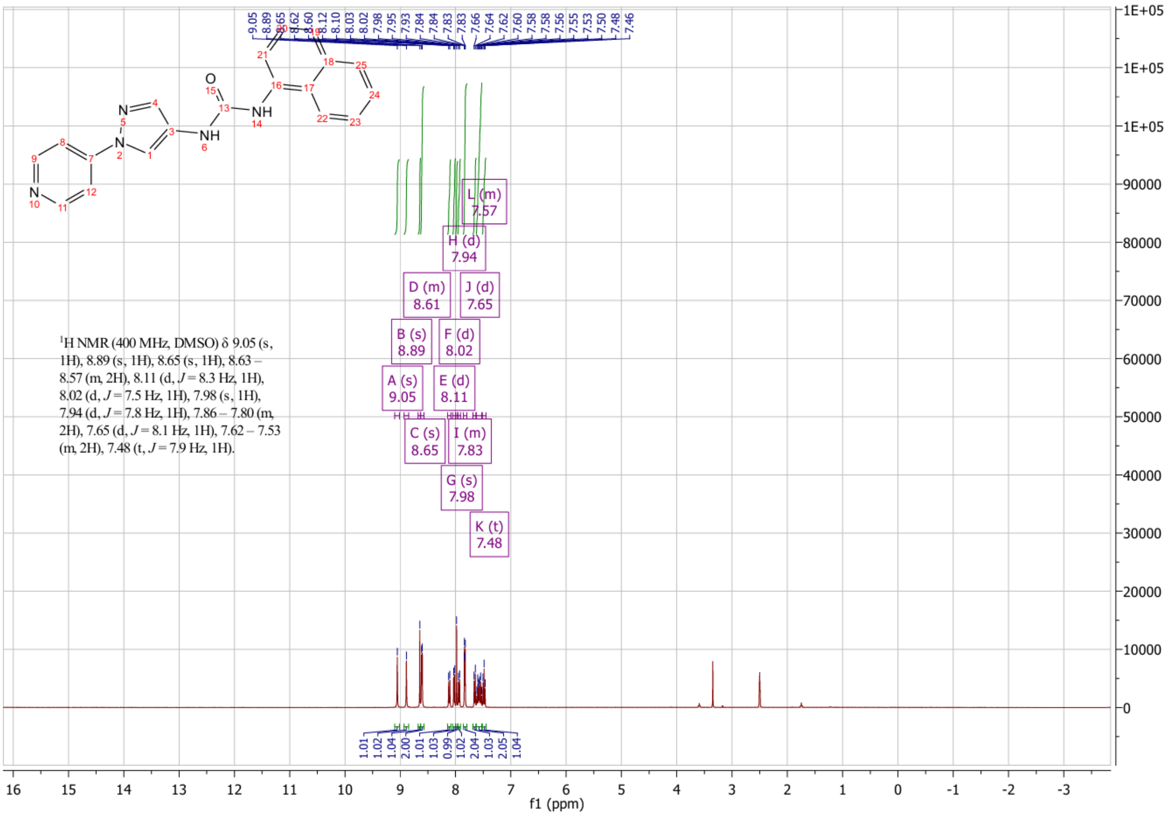
¹H NMR (400 MHz, DMSO) δ 9.05 (s, 1H), 8.89 (s, 1H), 8.65 (s, 1H), 8.63 – 8.57 (m, 2H), 8.11 (d, J = 8.3 Hz, 1H), 8.02 (d, J = 7.5 Hz, 1H), 7.98 (s, 1H), 7.94 (d, J = 7.8 Hz, 1H), 7.86 – 7.80 (m, 2H), 7.65 (d, J = 8.1 Hz, 1H), 7.62 – 7.53 (m, 2H), 7.48 (t, J = 7.9 Hz, 1H).
f1 (ppm)

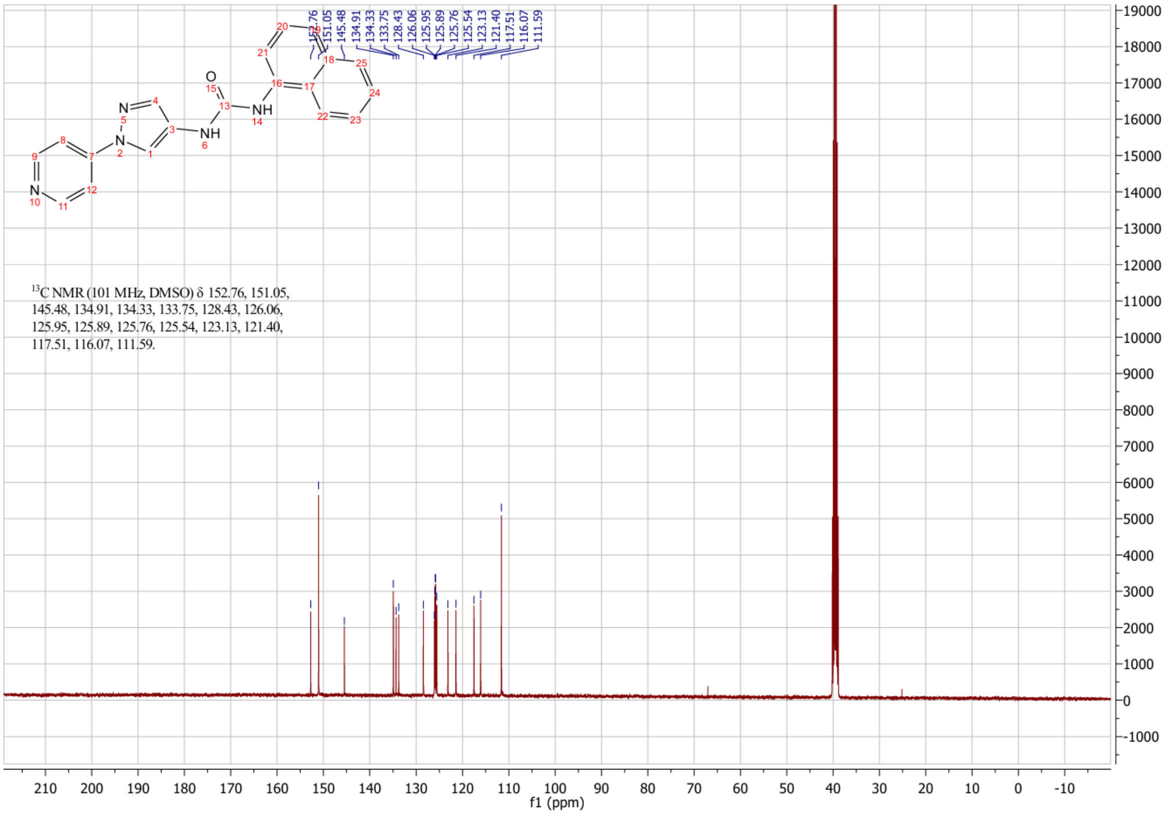
¹³C NMR (101 MHz, DMSO) δ 152.76, 151.05, 145.48, 134.91, 134.33, 133.75, 128.43, 126.06, 125.95, 125.89, 125.76, 125.54, 123.13, 121.40, 117.51, 116.07, 111.59.
f1 (ppm)

11.3 Table 3

20

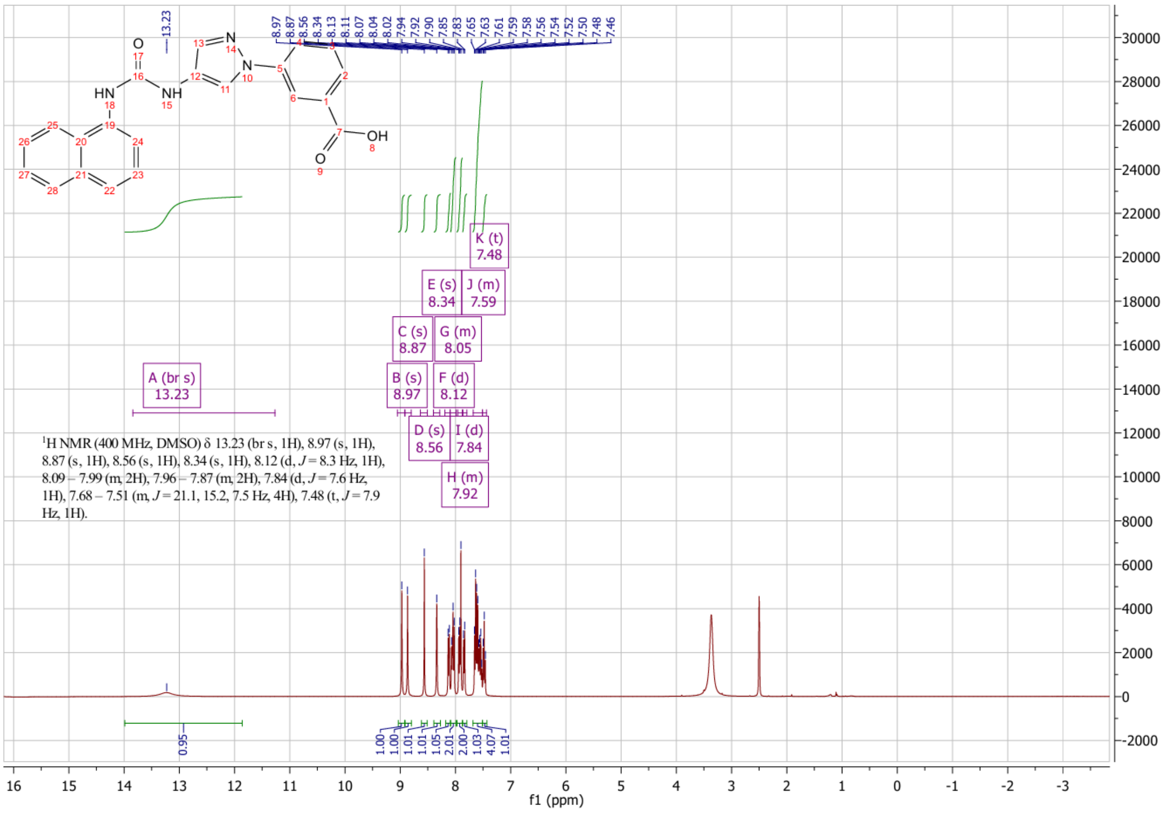

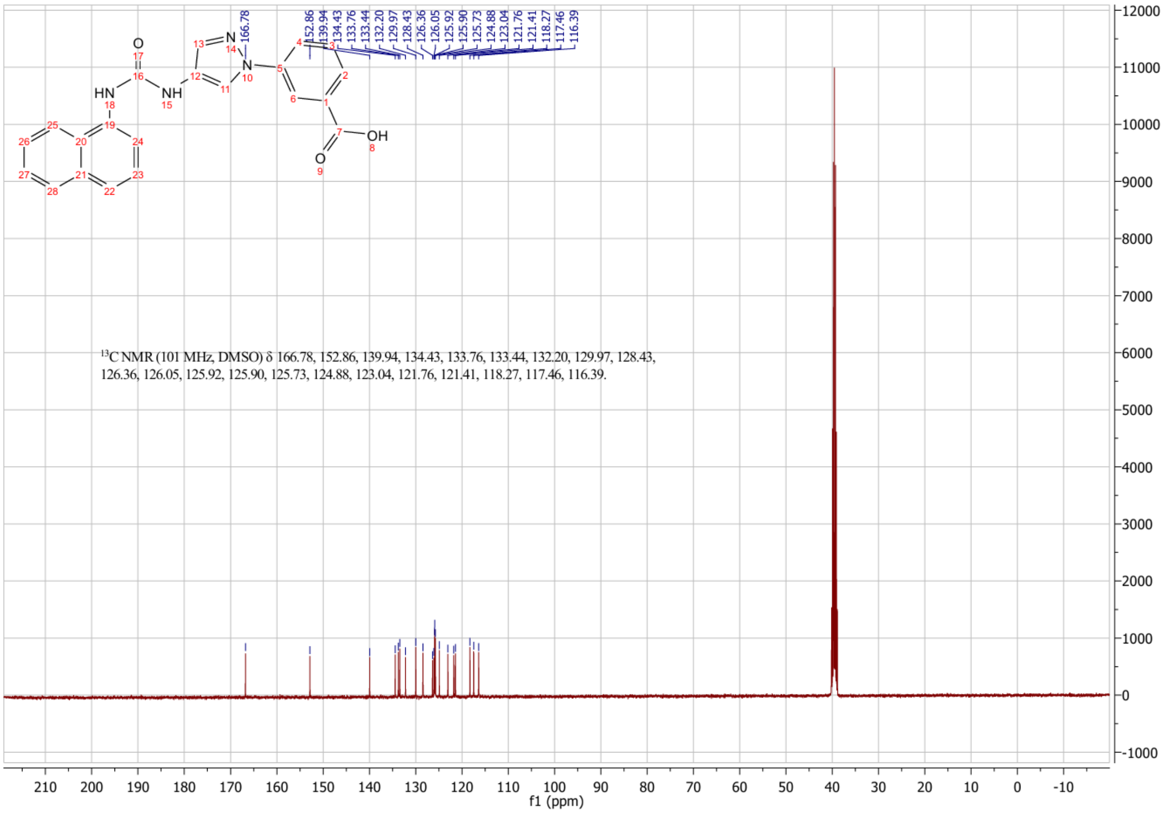

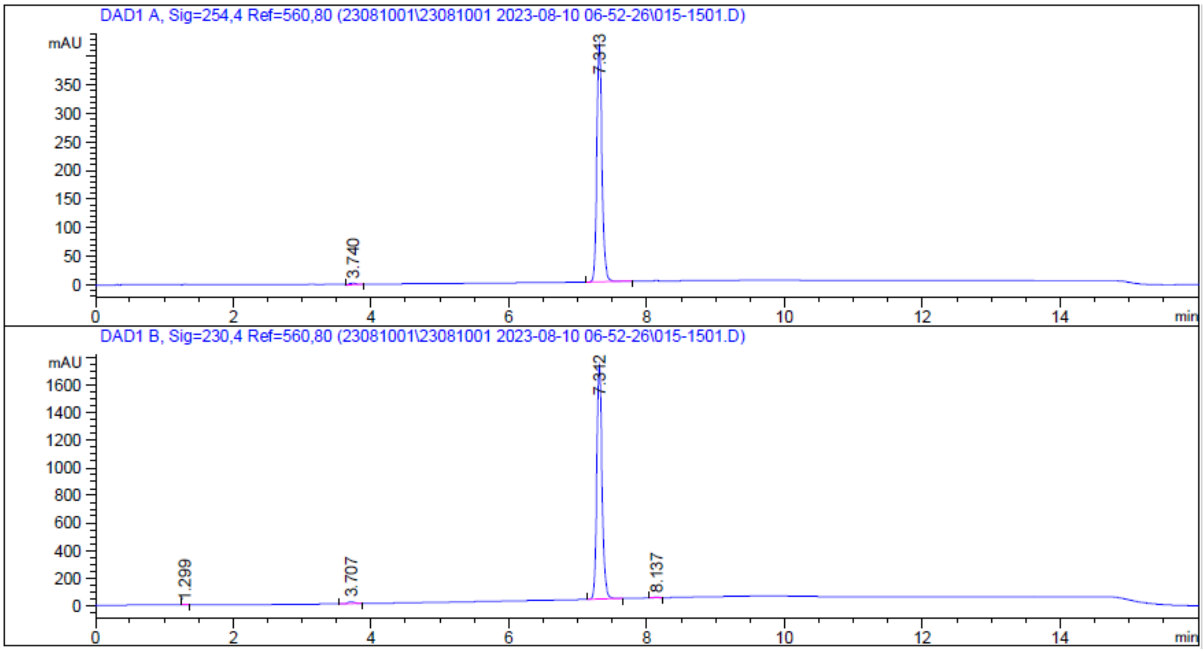

===

Area Percent Report

===

Sorted By : Signal
Multiplier: : 1.0000
Dilution: : 1.0000
Use Multiplier & Dilution Factor with ISTDs

Signal 1: DAD1 A, Sig=254,4 Ref=560,80

Peak #	RetTime [min]	Type	Width [min]	Area [mAU*s]	Height [mAU]	Area %
1	3.740	BB	0.0887	16.78467	2.55402	0.7655
2	7.313	BB	0.0813	2175.96289	414.08026	99.2345

Totals : 2192.74756 416.63429

Signal 2: DAD1 B, Sig=230,4 Ref=560,80

Peak #	RetTime [min]	Type	Width [min]	Area [mAU*s]	Height [mAU]	Area %
1	1.299	BB	0.0473	7.59005	2.66158	0.0835
2	3.707	BB	0.0999	87.31868	13.77715	0.9601
3	7.312	BB	0.0820	8980.97070	1690.32544	98.7495
4	8.137	BB	0.0725	18.82354	4.02916	0.2070

Totals : 9094.70298 1710.79332

===

*** End of Report ***

21a

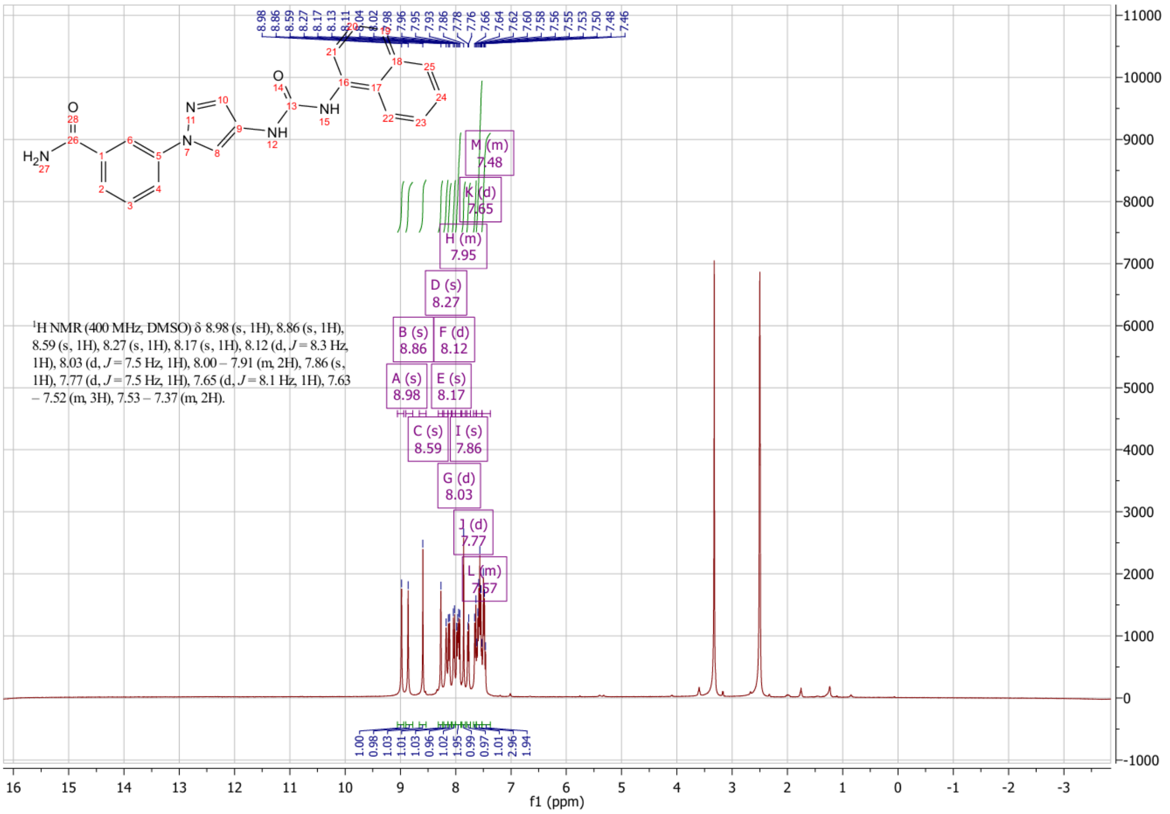

^{1}H NMR (400 MHz, DMSO) δ 8.98 (s, 1H), 8.86 (s, 1H), 8.59 (s, 1H), 8.27 (s, 1H), 8.17 (s, 1H), 8.12 (d, J = 8.3 Hz, 1H), 8.03 (d, J = 7.5 Hz, 1H), 8.00 – 7.91 (m, 2H), 7.86 (s, 1H), 7.77 (d, J = 7.5 Hz, 1H), 7.65 (d, J = 8.1 Hz, 1H), 7.63 – 7.52 (m, 3H), 7.53 – 7.37 (m, 2H).
f1 (ppm)

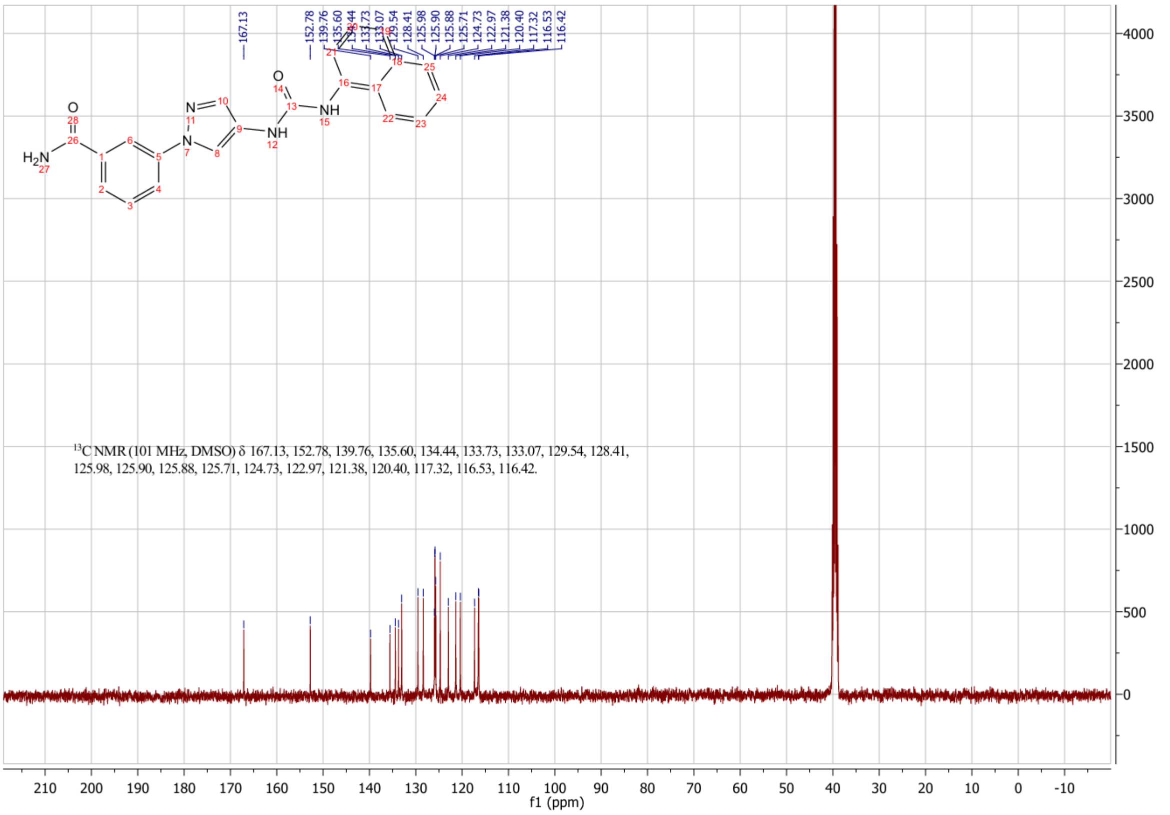

^{13}C NMR (101 MHz, DMSO) δ 167.13, 152.78, 139.76, 135.60, 134.44, 133.73, 133.07, 129.54, 128.41, 125.98, 125.90, 125.88, 125.71, 124.73, 122.97, 121.38, 120.40, 117.32, 116.53, 116.42.
f1 (ppm)

21b

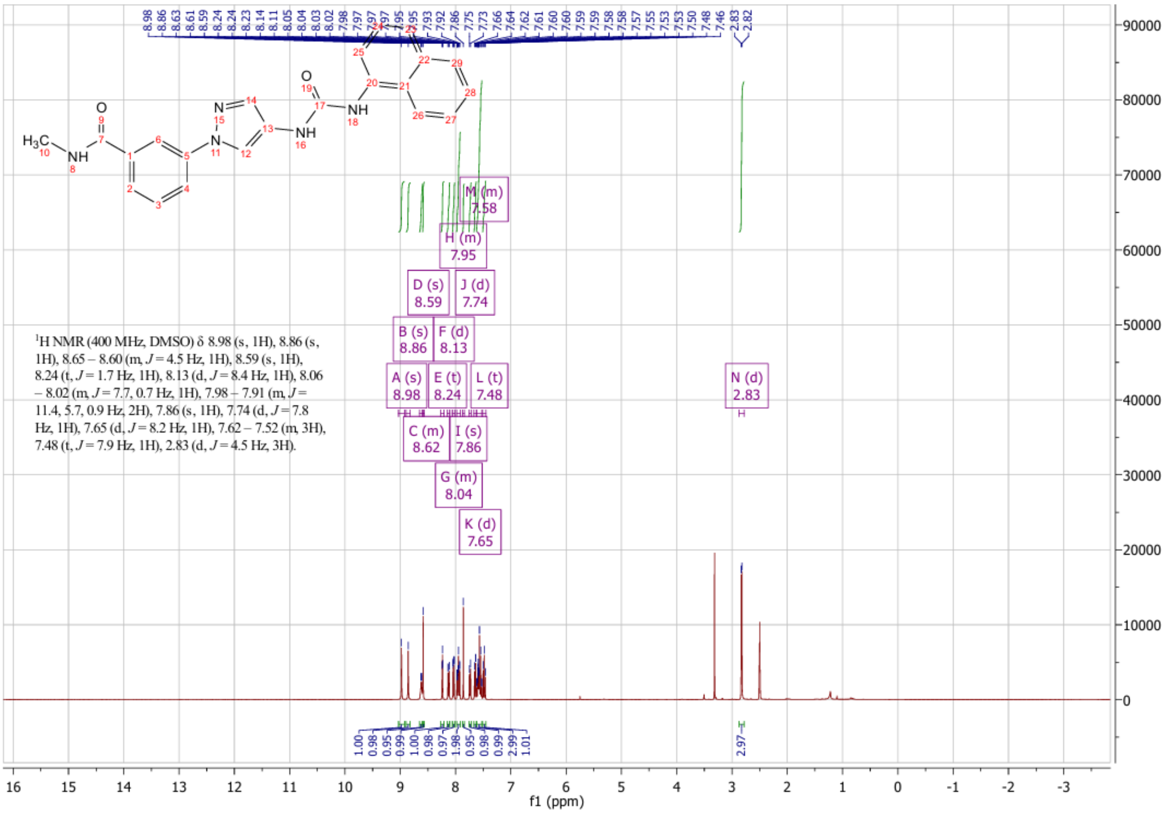

^{1}H NMR (400 MHz, DMSO) δ 8.98 (s, 1H), 8.86 (s, 1H), 8.65 – 8.60 (m, J = 4.5 Hz, 1H), 8.59 (s, 1H), 8.24 (t, J = 1.7 Hz, 1H), 8.13 (d, J = 8.4 Hz, 1H), 8.06 – 8.02 (m, J = 7.7, 0.7 Hz, 1H), 7.98 – 7.91 (m, J = 11.4, 5.7, 0.9 Hz, 2H), 7.86 (s, 1H), 7.74 (d, J = 7.8 Hz, 1H), 7.65 (d, J = 8.2 Hz, 1H), 7.62 – 7.52 (m, 3H), 7.48 (t, J = 7.9 Hz, 1H), 2.83 (d, J = 4.5 Hz, 3H).
f1 (ppm)

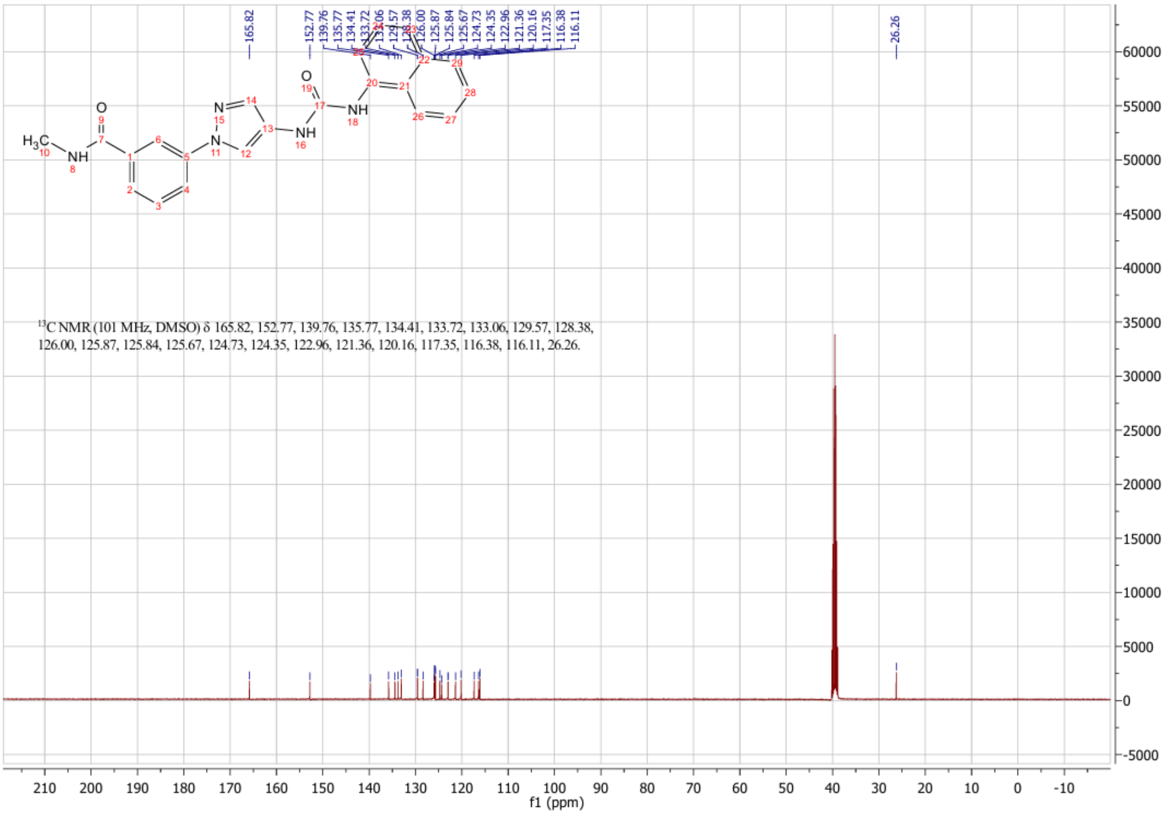

^{13}C NMR (101 MHz, DMSO) δ 165.82, 152.77, 139.76, 135.77, 134.41, 133.72, 133.06, 129.57, 128.38, 126.00, 125.87, 125.84, 125.67, 124.73, 124.35, 122.96, 121.36, 120.16, 117.35, 116.38, 116.11, 26.26.
f1 (ppm)

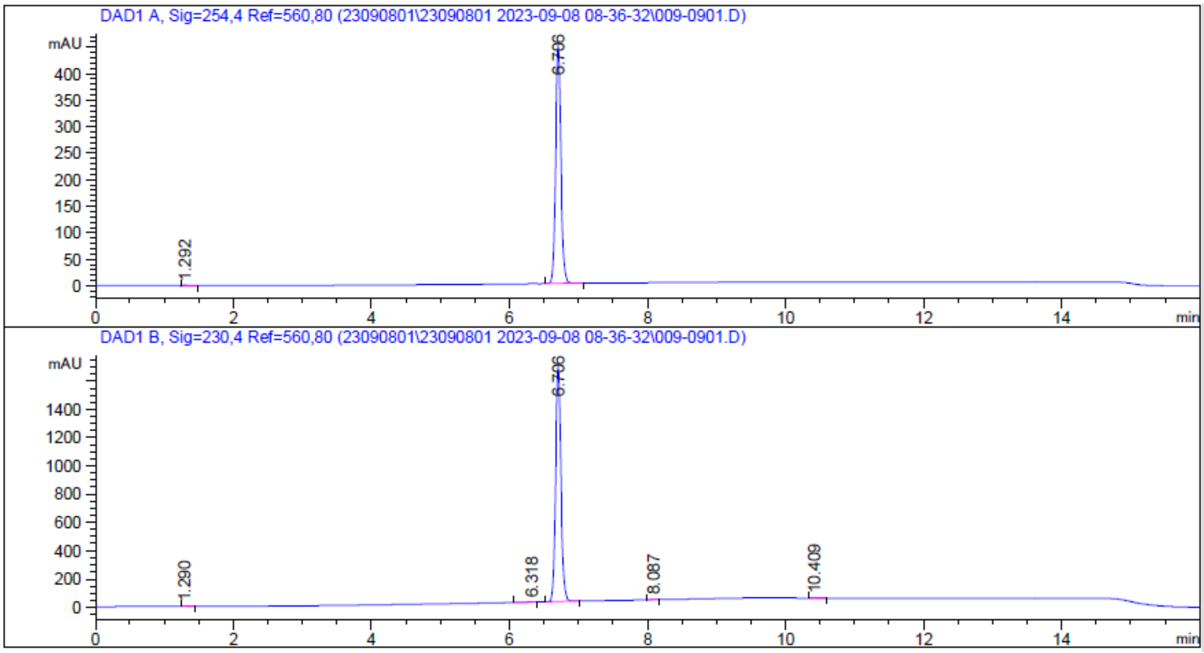

Area Percent Report

Sorted By : Signal
Multiplier: : 1.0000
Dilution: : 1.0000
Use Multiplier & Dilution Factor with ISTDs

Signal 1: DAD1 A, Sig=254,4 Ref=560,80

Peak #	RetTime [min]	Type	Width [min]	Area [mAU*s]	Height [mAU]	Area %
1	1.292	BB	0.0524	6.31084	1.82360	0.2647
2	6.706	BB	0.0819	2377.50024	448.23489	99.7353

Totals : 2383.81109 450.05849

Signal 2: DAD1 B, Sig=230,4 Ref=560,80

Peak #	RetTime [min]	Type	Width [min]	Area [mAU*s]	Height [mAU]	Area %
1	1.290	BB	0.0487	13.61036	4.32276	0.1522
2	6.318	BB	0.0840	12.72316	2.31842	0.1423
3	6.706	BB	0.0826	8894.79883	1658.85437	99.4717
4	8.087	BB	0.0695	11.14312	2.52477	0.1246
5	10.409	BB	0.0870	9.76582	1.70064	0.1092

Totals : 8942.04128 1669.72095

*** End of Report ***

21c

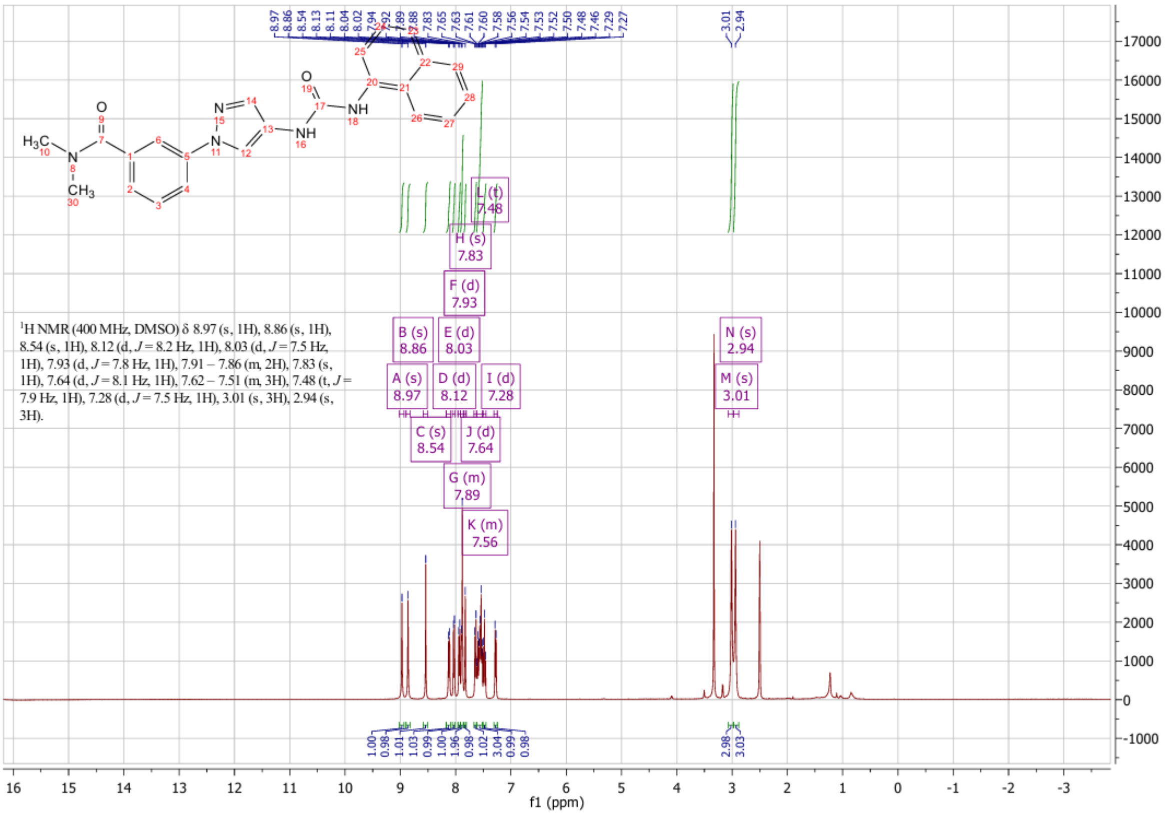

^{1}H NMR (400 MHz, DMSO) δ 8.97 (s, 1H), 8.86 (s, 1H), 8.54 (s, 1H), 8.12 (d, J = 8.2 Hz, 1H), 8.03 (d, J = 7.5 Hz, 1H), 7.93 (d, J = 7.8 Hz, 1H), 7.91 – 7.86 (m, 2H), 7.83 (s, 1H), 7.64 (d, J = 8.1 Hz, 1H), 7.62 – 7.51 (m, 3H), 7.48 (t, J = 7.9 Hz, 1H), 7.28 (d, J = 7.5 Hz, 1H), 3.01 (s, 3H), 2.94 (s, 3H).

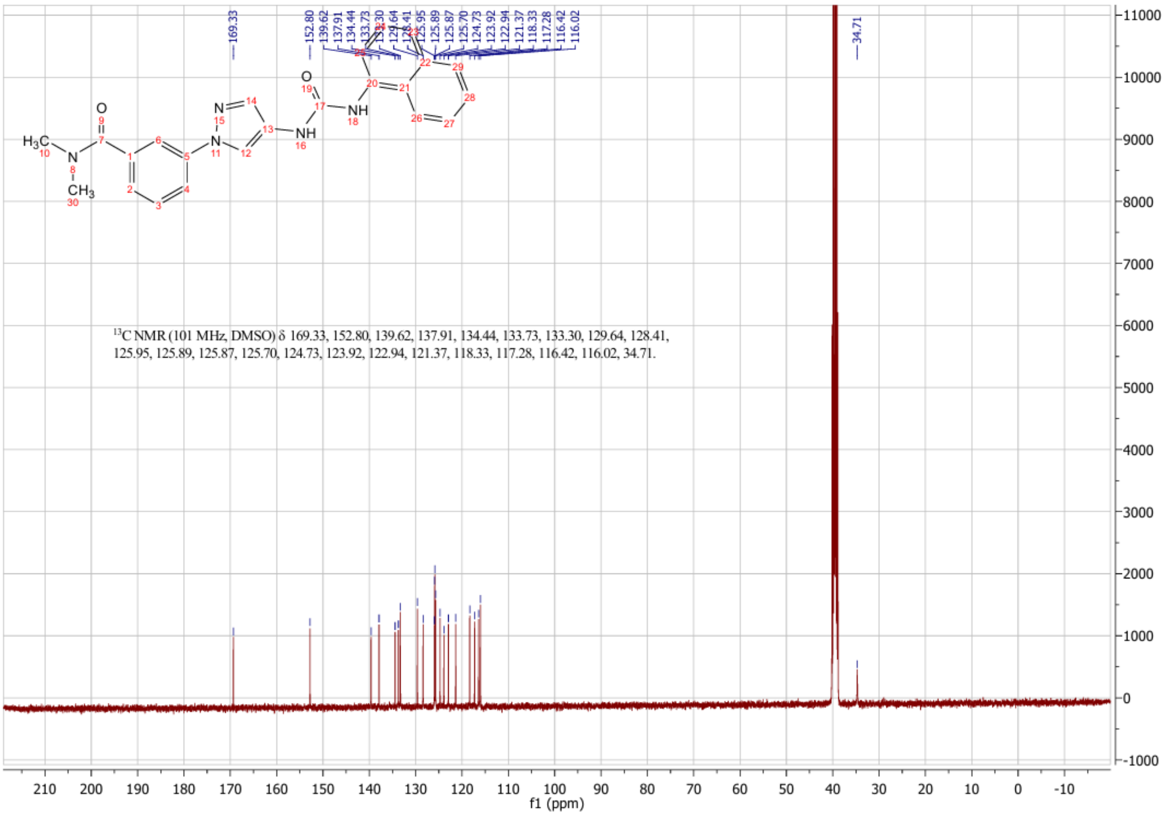

^{13}C NMR (101 MHz, DMSO) δ 169.33, 152.80, 139.62, 137.91, 134.44, 133.73, 133.30, 129.64, 128.41, 125.95, 125.89, 125.87, 125.70, 124.73, 123.92, 122.94, 121.37, 118.33, 117.28, 116.42, 116.02, 34.71.

21d

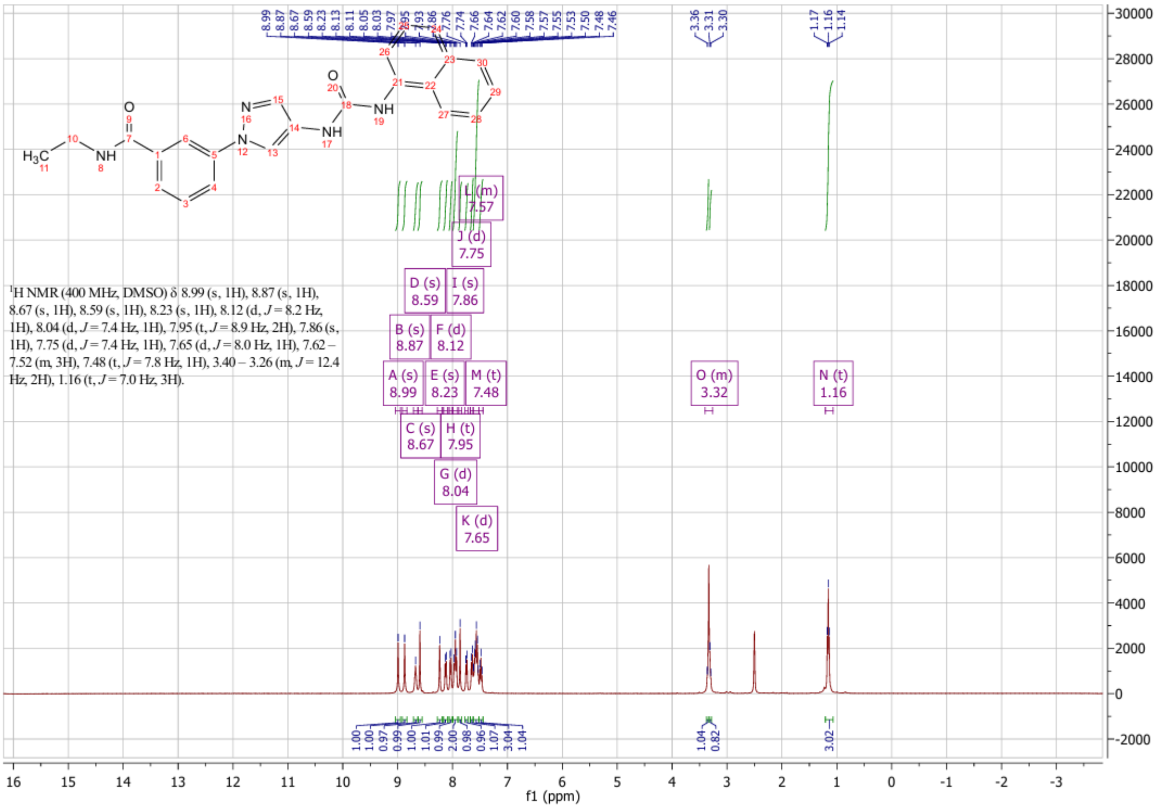

^{1}H NMR (400 MHz, DMSO) δ 8.99 (s, 1H), 8.87 (s, 1H), 8.67 (s, 1H), 8.59 (s, 1H), 8.23 (s, 1H), 8.12 (d, J = 8.2 Hz, 1H), 8.04 (d, J = 7.4 Hz, 1H), 7.95 (t, J = 8.9 Hz, 2H), 7.86 (s, 1H), 7.75 (d, J = 7.4 Hz, 1H), 7.65 (d, J = 8.0 Hz, 1H), 7.62 – 7.52 (m, 3H), 7.48 (t, J = 7.8 Hz, 1H), 3.40 – 3.26 (m, J = 12.4 Hz, 2H), 1.16 (t, J = 7.0 Hz, 3H).
f1 (ppm)

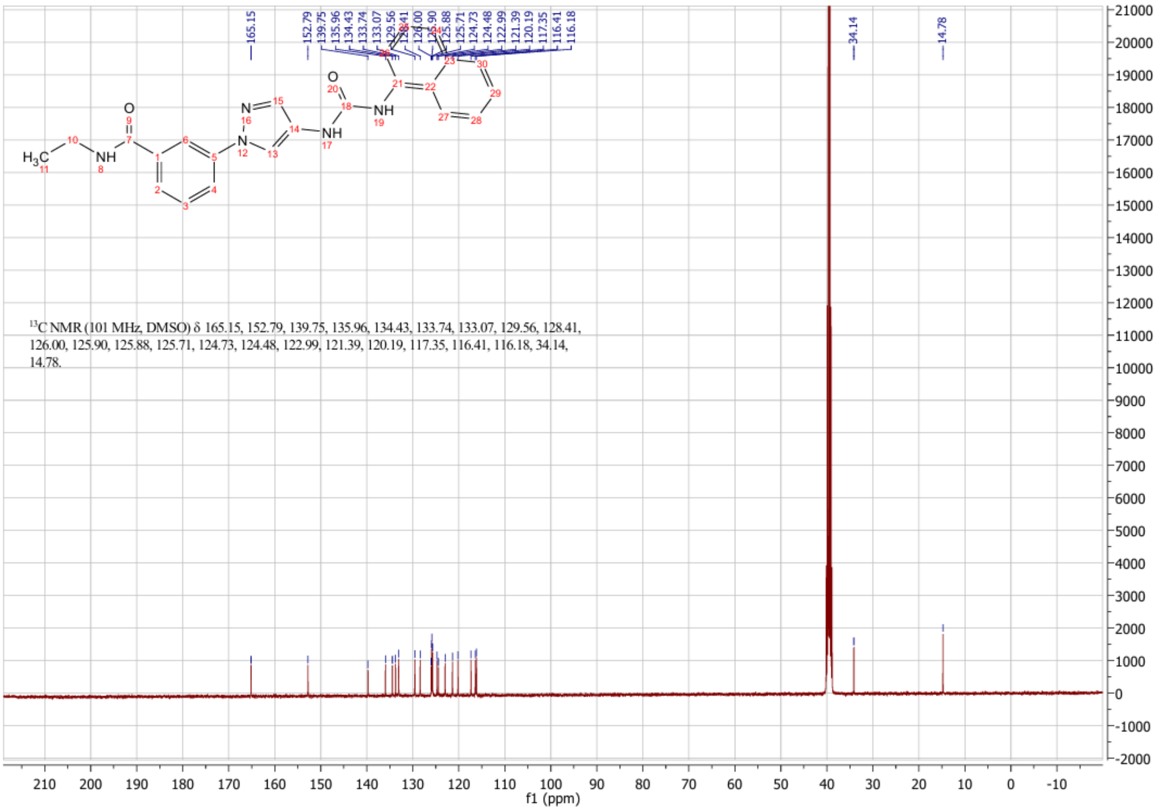

^{13}C NMR (101 MHz, DMSO) δ 165.15, 152.79, 139.75, 135.96, 134.43, 133.74, 133.07, 129.56, 128.41, 126.00, 125.90, 125.88, 125.71, 124.73, 124.48, 122.99, 121.39, 120.19, 117.35, 116.41, 116.18, 34.14, 14.78.
f1 (ppm)

21e

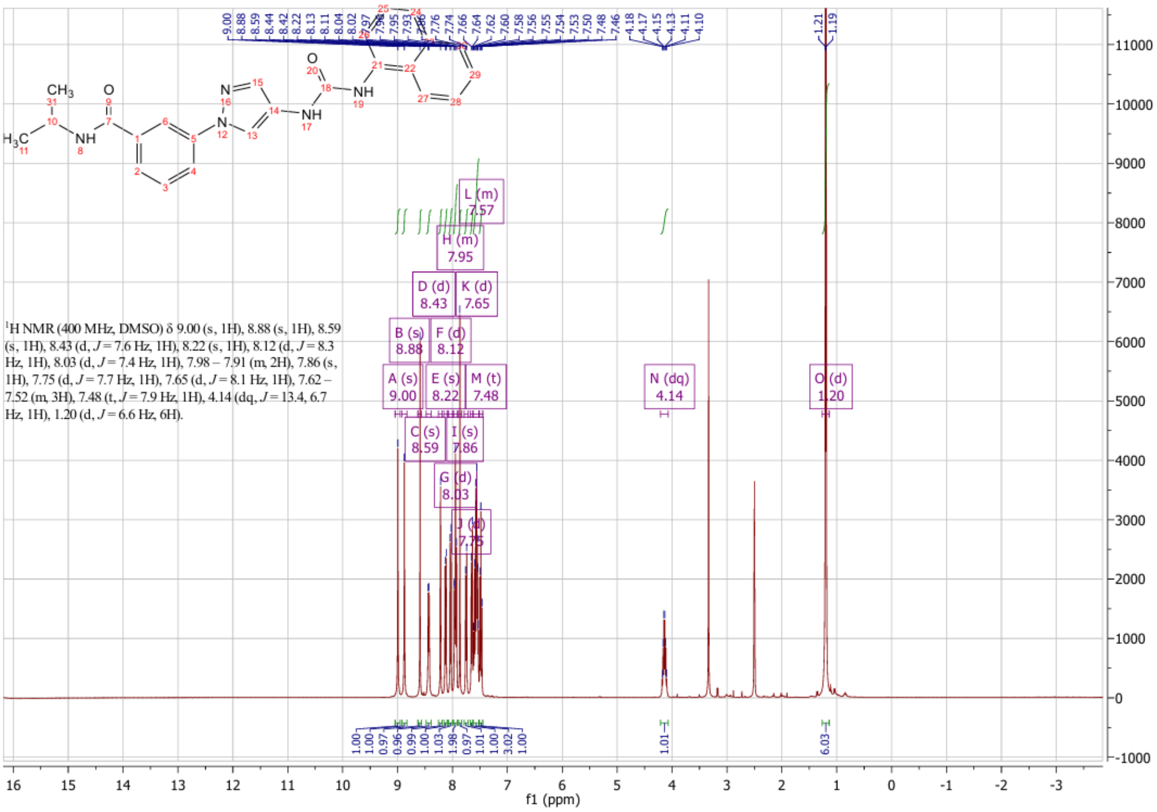
^{1}H NMR (400 MHz, DMSO) δ 9.00 (s, 1H), 8.88 (s, 1H), 8.59 (s, 1H), 8.43 (d, J = 7.6 Hz, 1H), 8.22 (s, 1H), 8.12 (d, J = 8.3 Hz, 1H), 8.03 (d, J = 7.4 Hz, 1H), 7.98 – 7.91 (m, 2H), 7.86 (s, 1H), 7.75 (d, J = 7.7 Hz, 1H), 7.65 (d, J = 8.1 Hz, 1H), 7.62 – 7.52 (m, 3H), 7.48 (t, J = 7.9 Hz, 1H), 4.14 (dq, J = 13.4, 6.7 Hz, 1H), 1.20 (d, J = 6.6 Hz, 6H).
f1 (ppm)

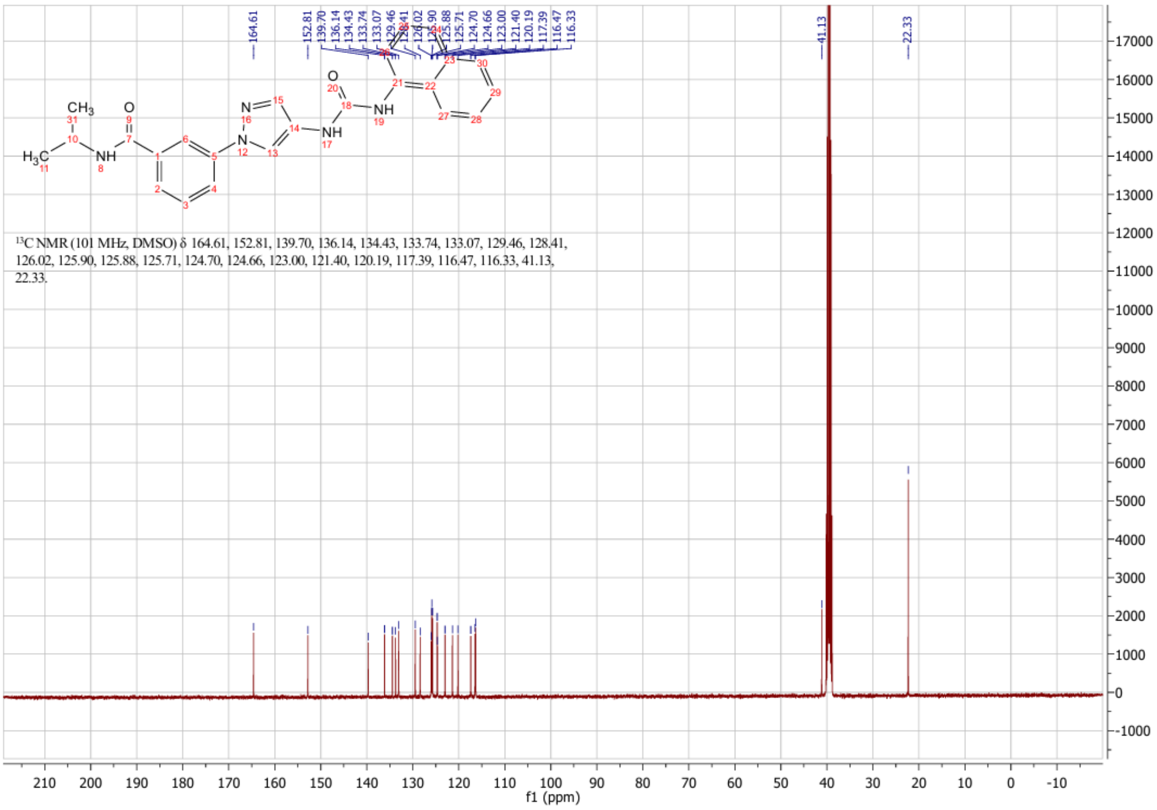
^{13}C NMR (101 MHz, DMSO) δ 164.61, 152.81, 139.70, 136.14, 134.43, 133.74, 133.07, 129.46, 128.41, 126.02, 125.90, 125.88, 125.71, 124.70, 124.66, 123.00, 121.40, 120.19, 117.39, 116.47, 116.33, 41.13, 22.33.
f1 (ppm)

21f

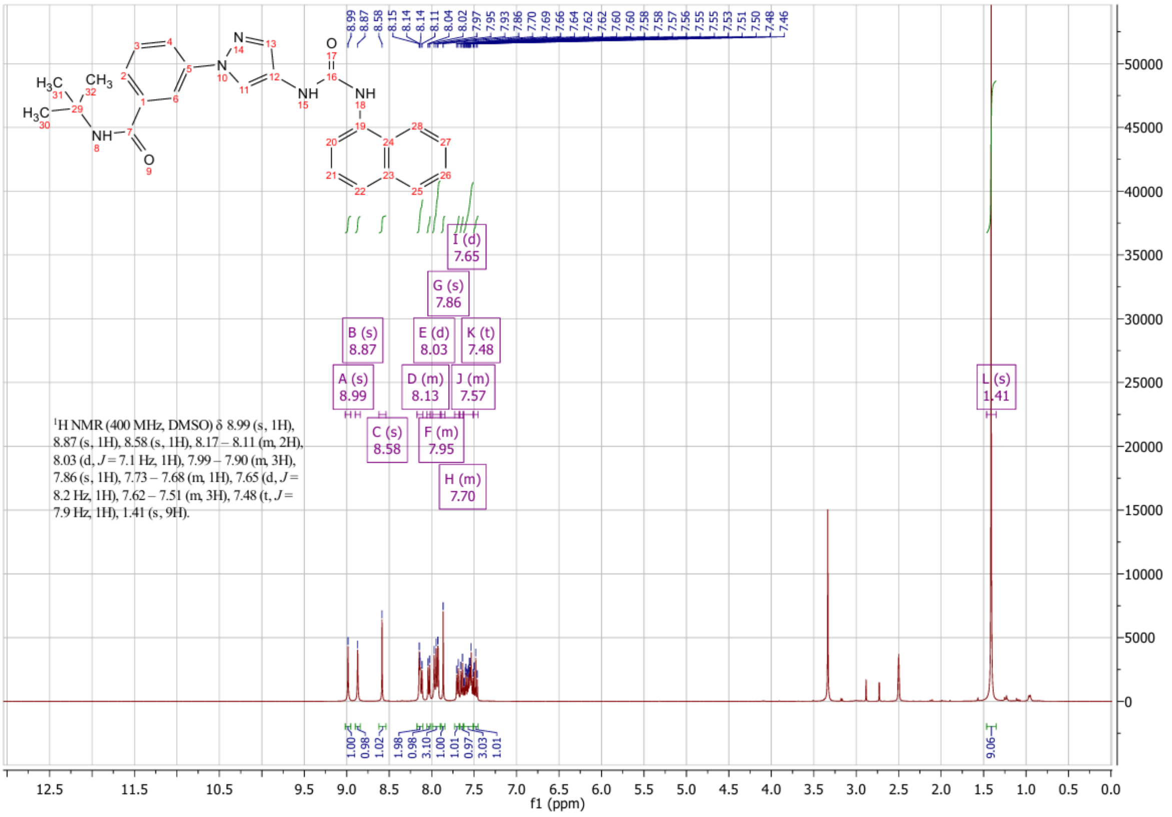

^{1}H NMR (400 MHz, DMSO) δ 8.99 (s, 1H), 8.87 (s, 1H), 8.58 (s, 1H), 8.17 – 8.11 (m, 2H), 8.03 (d, J = 7.1 Hz, 1H), 7.99 – 7.90 (m, 3H), 7.86 (s, 1H), 7.73 – 7.68 (m, 1H), 7.65 (d, J = 8.2 Hz, 1H), 7.62 – 7.51 (m, 3H), 7.48 (t, J = 7.9 Hz, 1H), 1.41 (s, 9H).
f1 (ppm)

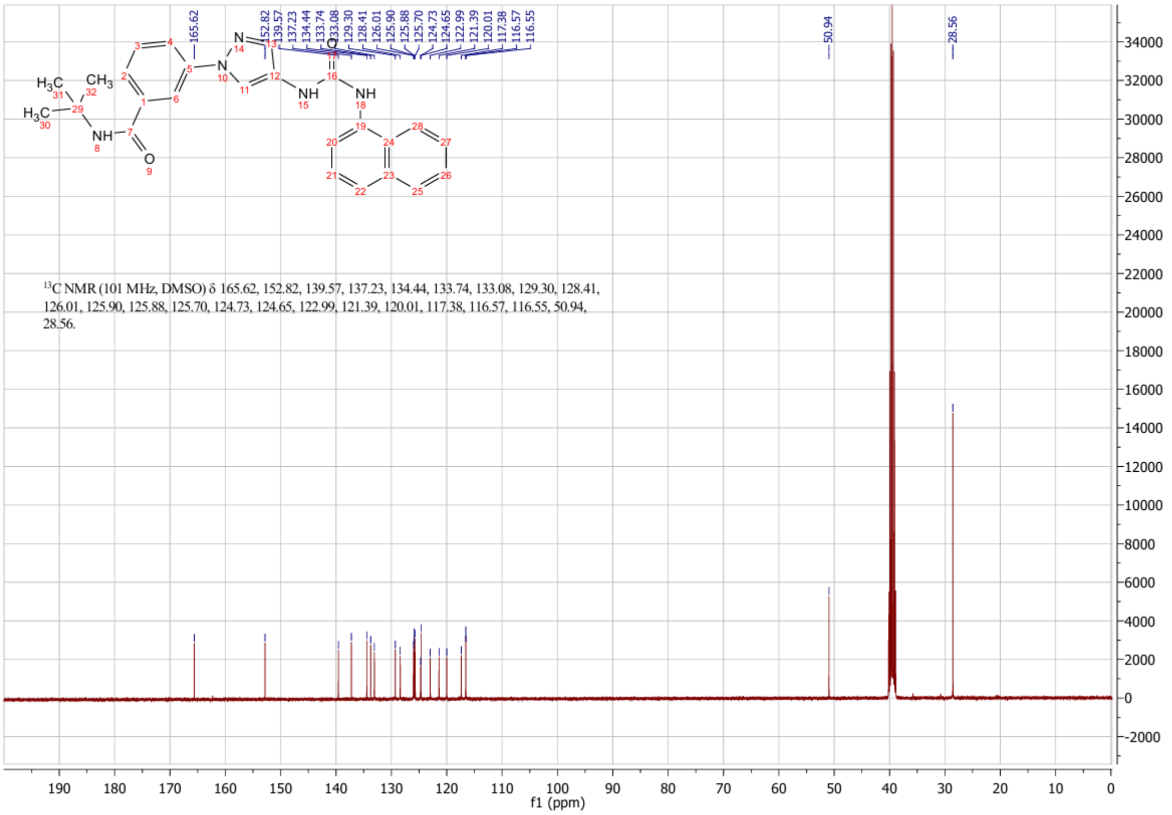

^{13}C NMR (101 MHz, DMSO) δ 165.62, 152.82, 139.57, 137.23, 134.44, 133.74, 133.08, 129.30, 128.41, 126.01, 125.90, 125.88, 125.70, 124.73, 124.65, 122.99, 121.39, 120.01, 117.38, 116.57, 116.55, 50.94, 28.56.
f1 (ppm)

21g

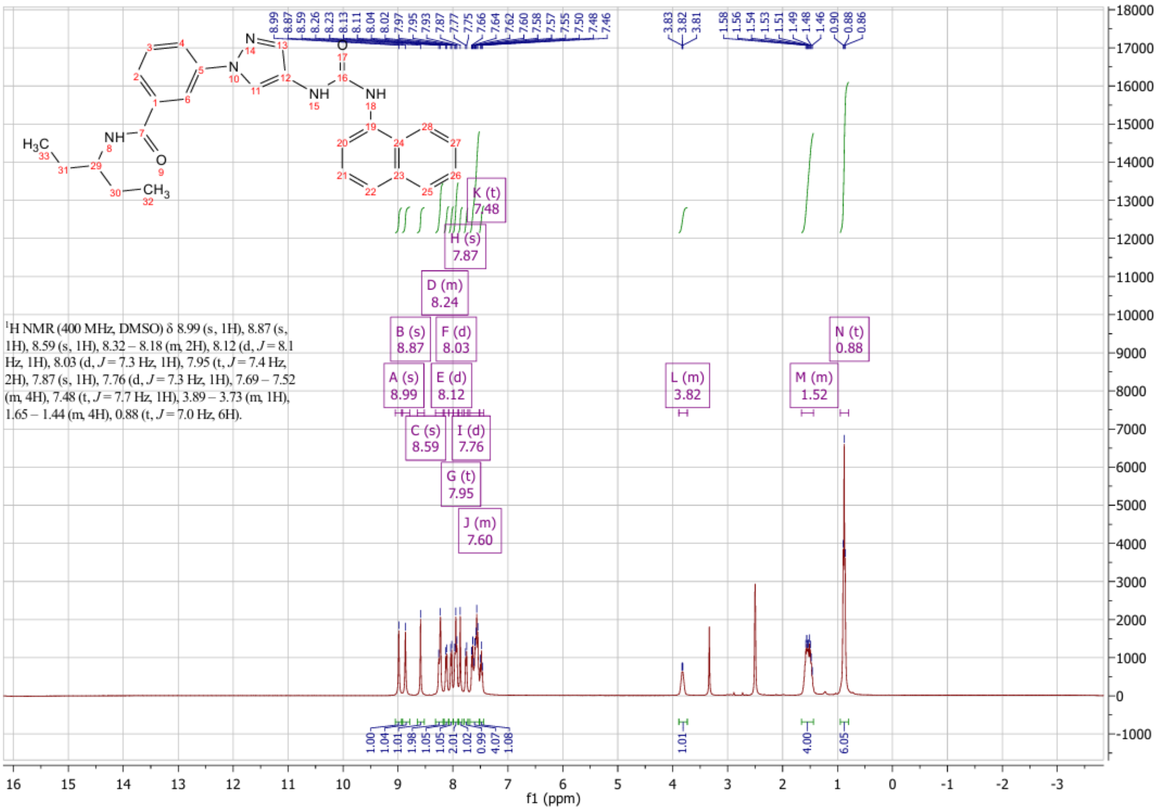

1H NMR (400 MHz, DMSO) δ 8.99 (s, 1H), 8.87 (s, 1H), 8.59 (s, 1H), 8.32 – 8.18 (m, 2H), 8.12 (d, J = 8.1 Hz, 1H), 8.03 (d, J = 7.3 Hz, 1H), 7.95 (t, J = 7.4 Hz, 2H), 7.87 (s, 1H), 7.76 (d, J = 7.3 Hz, 1H), 7.69 – 7.52 (m, 4H), 7.48 (t, J = 7.7 Hz, 1H), 3.89 – 3.73 (m, 1H), 1.65 – 1.44 (m, 4H), 0.88 (t, J = 7.0 Hz, 6H).
f1 (ppm)

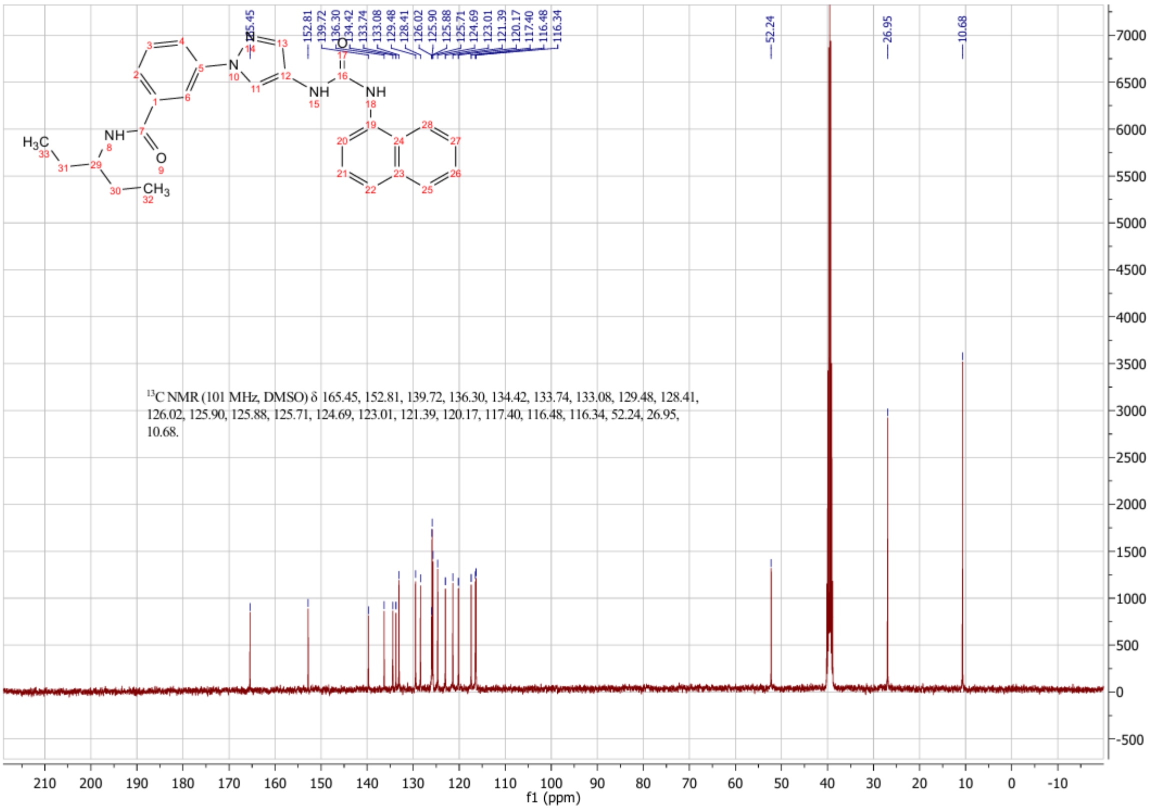

13C NMR (101 MHz, DMSO) δ 165.45, 152.81, 139.72, 136.30, 134.42, 133.74, 133.08, 129.48, 128.41, 126.02, 125.90, 125.88, 125.71, 124.69, 123.01, 121.39, 120.17, 117.40, 116.48, 116.34, 52.24, 26.95, 10.68.
f1 (ppm)

21h

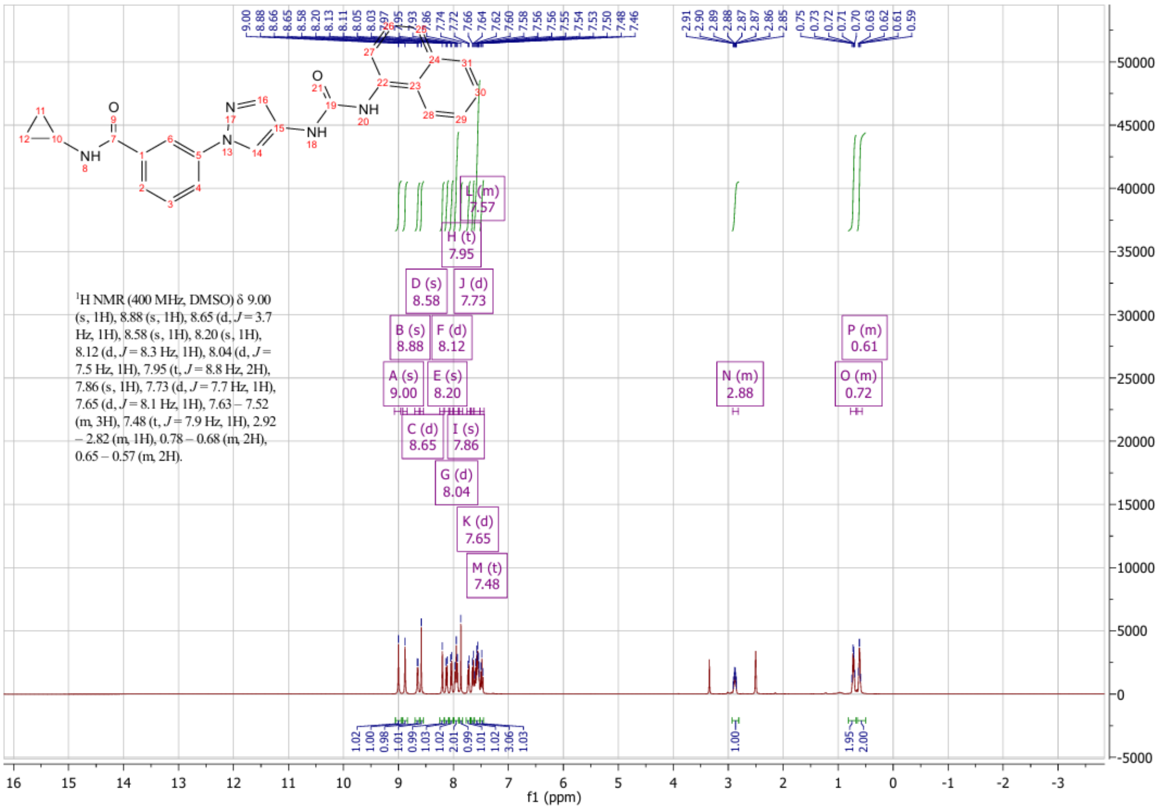

^{1}H NMR (400 MHz, DMSO) δ 9.00 (s, 1H), 8.88 (s, 1H), 8.65 (d, J = 3.7 Hz, 1H), 8.58 (s, 1H), 8.20 (s, 1H), 8.12 (d, J = 8.3 Hz, 1H), 8.04 (d, J = 7.5 Hz, 1H), 7.95 (t, J = 8.8 Hz, 2H), 7.86 (s, 1H), 7.73 (d, J = 7.7 Hz, 1H), 7.65 (d, J = 8.1 Hz, 1H), 7.63 – 7.52 (m, 3H), 7.48 (t, J = 7.9 Hz, 1H), 2.92 – 2.82 (m, 1H), 0.78 – 0.68 (m, 2H), 0.65 – 0.57 (m, 2H).
f1 (ppm)

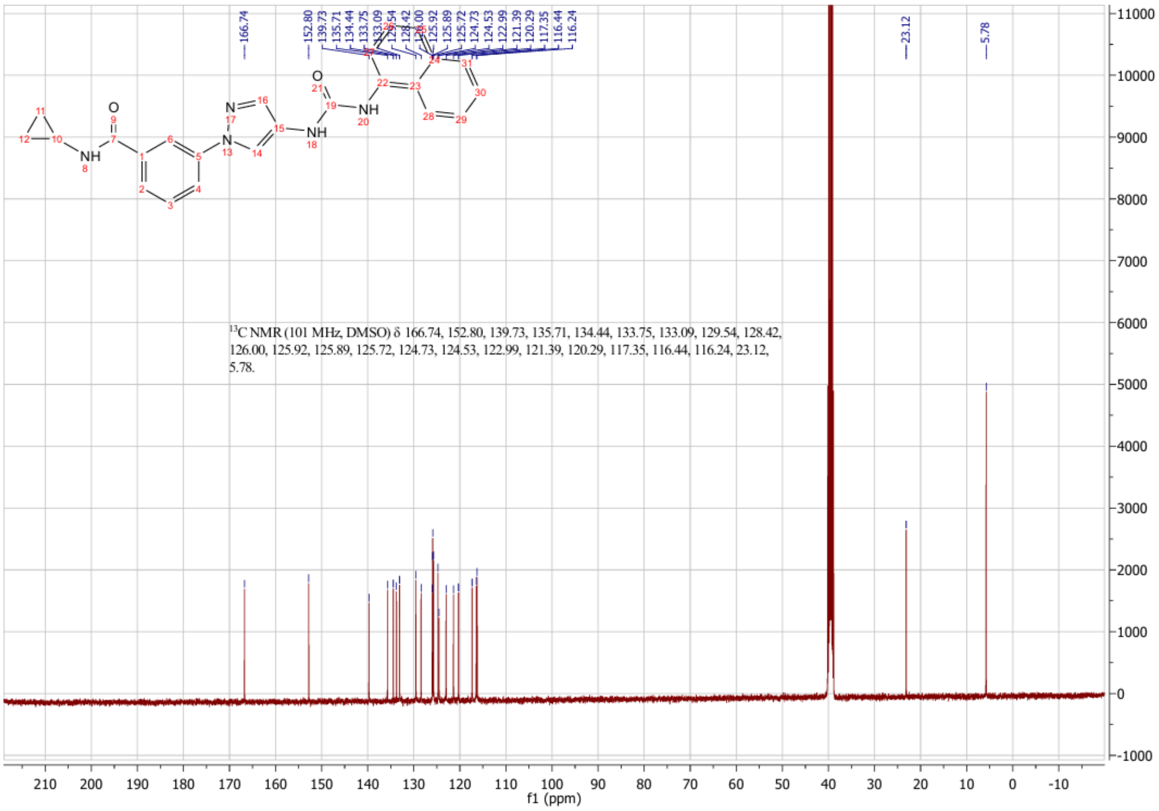

^{13}C NMR (101 MHz, DMSO) δ 166.74, 152.80, 139.73, 135.71, 134.44, 133.75, 133.09, 129.54, 128.42, 126.00, 125.92, 125.89, 125.72, 124.73, 124.53, 122.99, 121.39, 120.29, 117.35, 116.44, 116.24, 23.12, 5.78.
f1 (ppm)

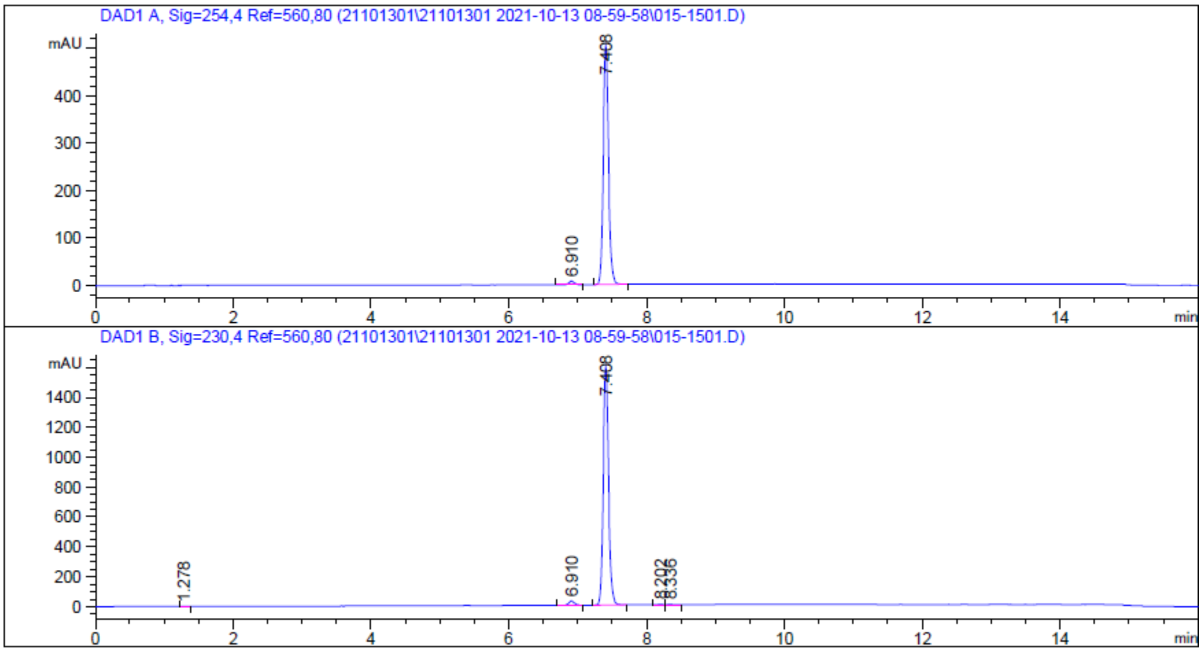

===

Area Percent Report

===

Sorted By : Signal
Multiplier: : 1.0000
Dilution: : 1.0000
Use Multiplier & Dilution Factor with ISTDs

Signal 1: DAD1 A, Sig=254,4 Ref=560,80

Peak #	RetTime [min]	Type	Width [min]	Area [mAU*s]	Height [mAU]	Area %
1	6.910	BB	0.0831	40.29370	7.44996	1.5024
2	7.408	BB	0.0792	2641.59131	503.44617	98.4976

Totals : 2681.88501 510.89613

Signal 2: DAD1 B, Sig=230,4 Ref=560,80

Peak #	RetTime [min]	Type	Width [min]	Area [mAU*s]	Height [mAU]	Area %
1	1.278	BB	0.0507	5.62923	1.69547	0.0651
2	6.910	BB	0.0822	166.68071	31.28740	1.9275
3	7.408	BB	0.0799	8451.96582	1594.02209	97.7402
4	8.202	BV	0.0751	11.67459	2.38444	0.1350
5	8.336	VB	0.0803	11.42939	2.07234	0.1322

Totals : 8647.37974 1631.46174

===

*** End of Report ***

21i

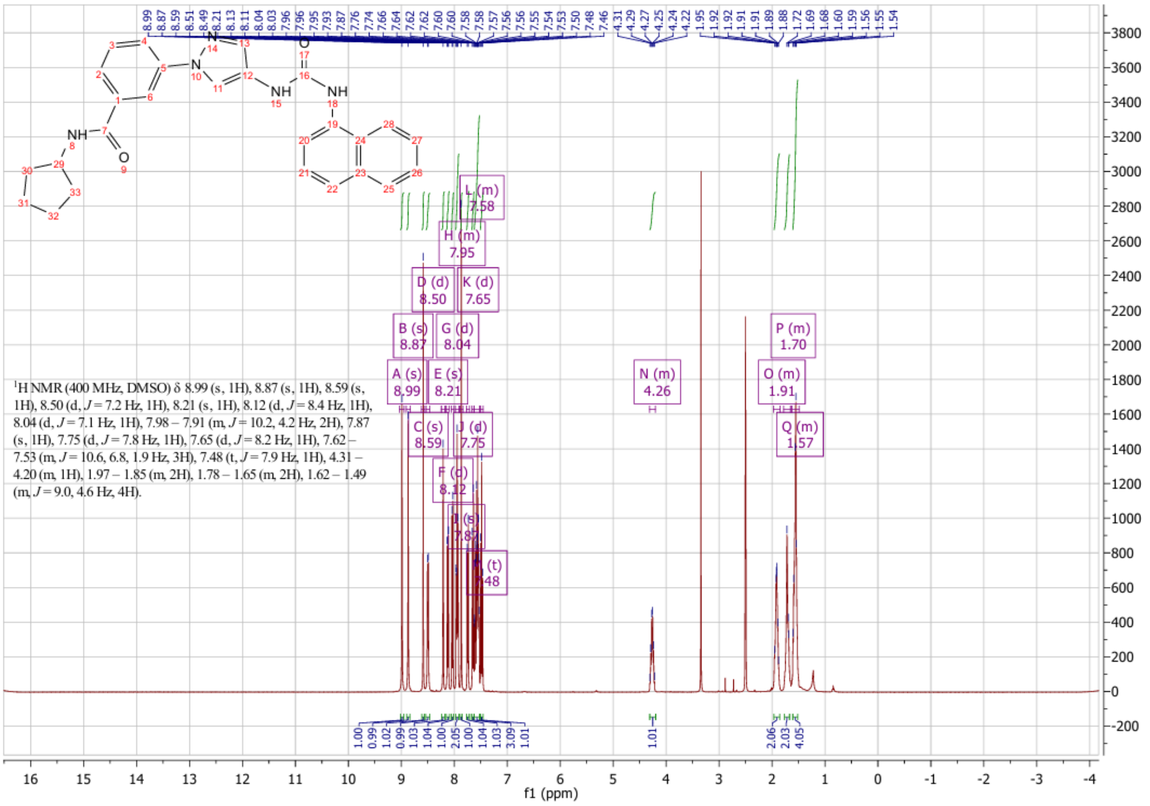

^{1}H NMR (400 MHz, DMSO) δ 8.99 (s, 1H), 8.87 (s, 1H), 8.59 (s, 1H), 8.50 (d, J = 7.2 Hz, 1H), 8.21 (s, 1H), 8.12 (d, J = 8.4 Hz, 1H), 8.04 (d, J = 7.1 Hz, 1H), 7.98 – 7.91 (m, J = 10.2, 4.2 Hz, 2H), 7.87 (s, 1H), 7.75 (d, J = 7.8 Hz, 1H), 7.65 (d, J = 8.2 Hz, 1H), 7.62 – 7.53 (m, J = 10.6, 6.8, 1.9 Hz, 3H), 7.48 (t, J = 7.9 Hz, 1H), 4.31 – 4.20 (m, 1H), 1.97 – 1.85 (m, 2H), 1.78 – 1.65 (m, 2H), 1.62 – 1.49 (m, J = 9.0, 4.6 Hz, 4H).
f1 (ppm)

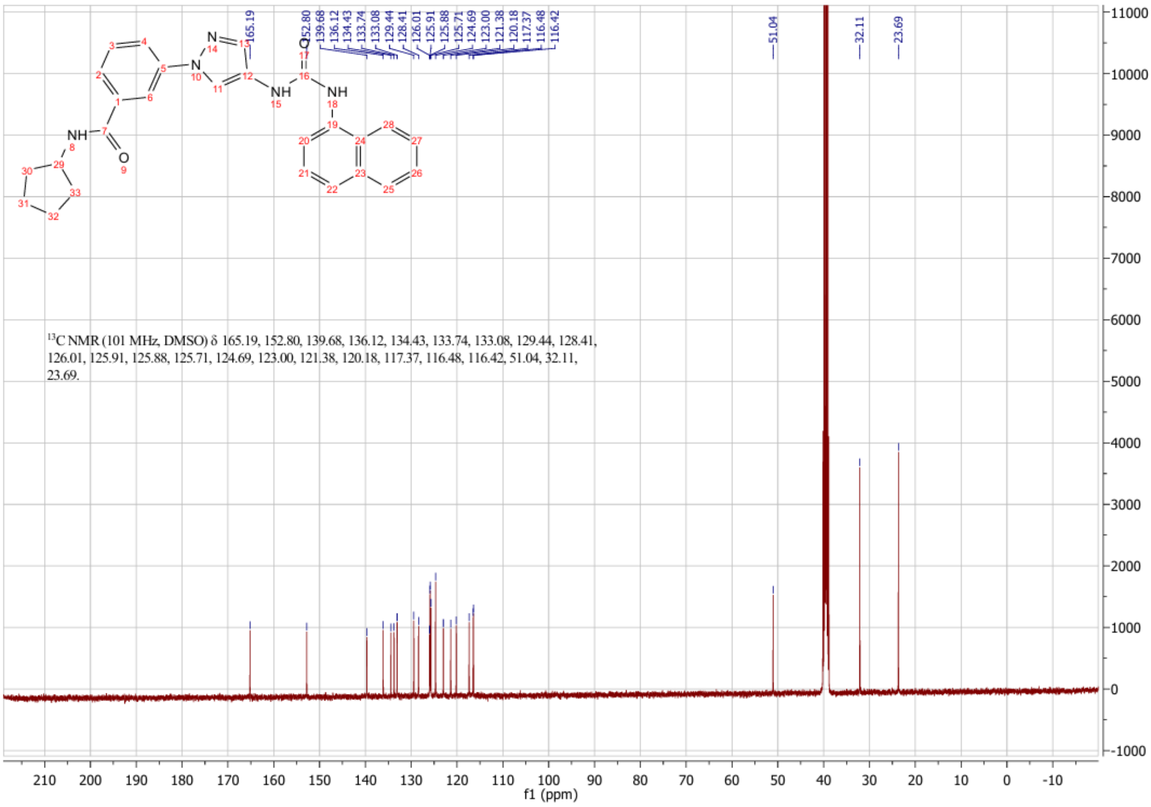

^{13}C NMR (101 MHz, DMSO) δ 165.19, 152.80, 139.68, 136.12, 134.43, 133.74, 133.08, 129.44, 128.41, 126.01, 125.91, 125.88, 125.71, 124.69, 123.00, 121.38, 120.18, 117.37, 116.48, 116.42, 51.04, 32.11, 23.69.
f1 (ppm)

21j

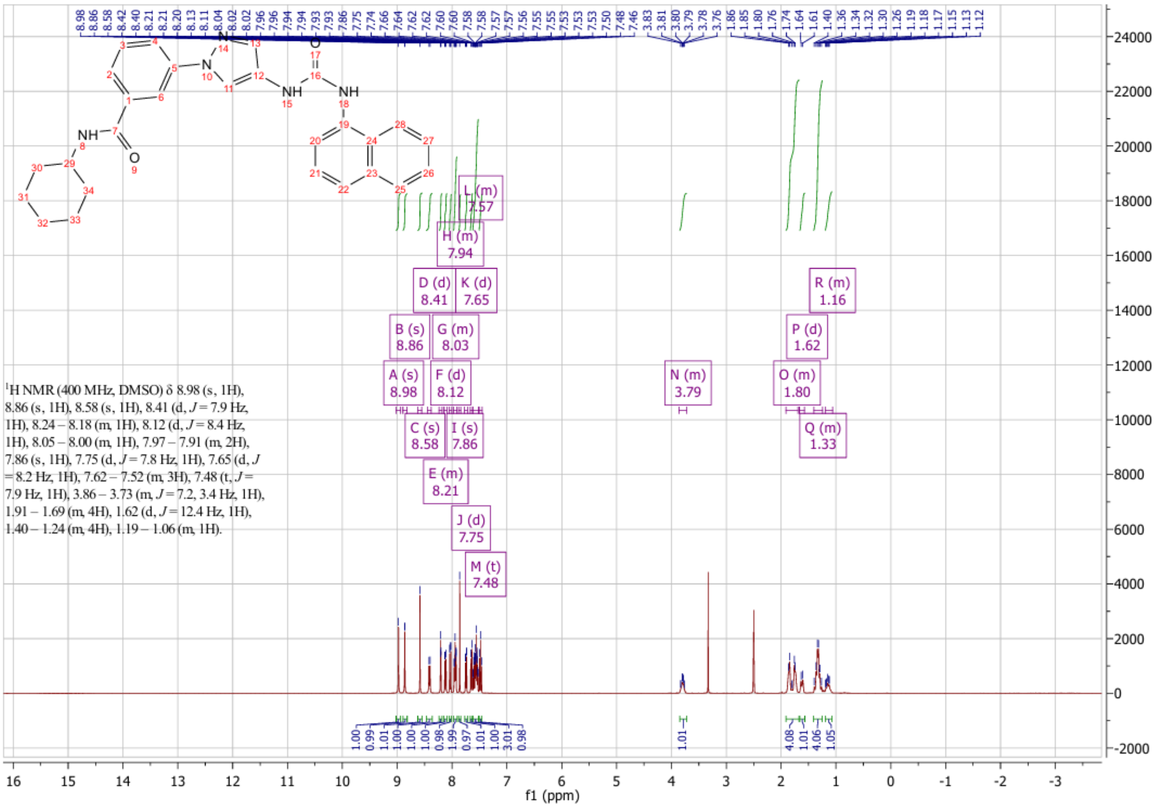
¹H NMR (400 MHz, DMSO) δ 8.98 (s, 1H), 8.86 (s, 1H), 8.58 (s, 1H), 8.41 (d, J = 7.9 Hz, 1H), 8.24 – 8.18 (m, 1H), 8.12 (d, J = 8.4 Hz, 1H), 8.05 – 8.00 (m, 1H), 7.97 – 7.91 (m, 2H), 7.86 (s, 1H), 7.75 (d, J = 7.8 Hz, 1H), 7.65 (d, J = 8.2 Hz, 1H), 7.62 – 7.52 (m, 3H), 7.48 (t, J = 7.9 Hz, 1H), 3.86 – 3.73 (m, J = 7.2, 3.4 Hz, 1H), 1.91 – 1.69 (m, 4H), 1.62 (d, J = 12.4 Hz, 1H), 1.40 – 1.24 (m, 4H), 1.19 – 1.06 (m, 1H).
f1 (ppm)

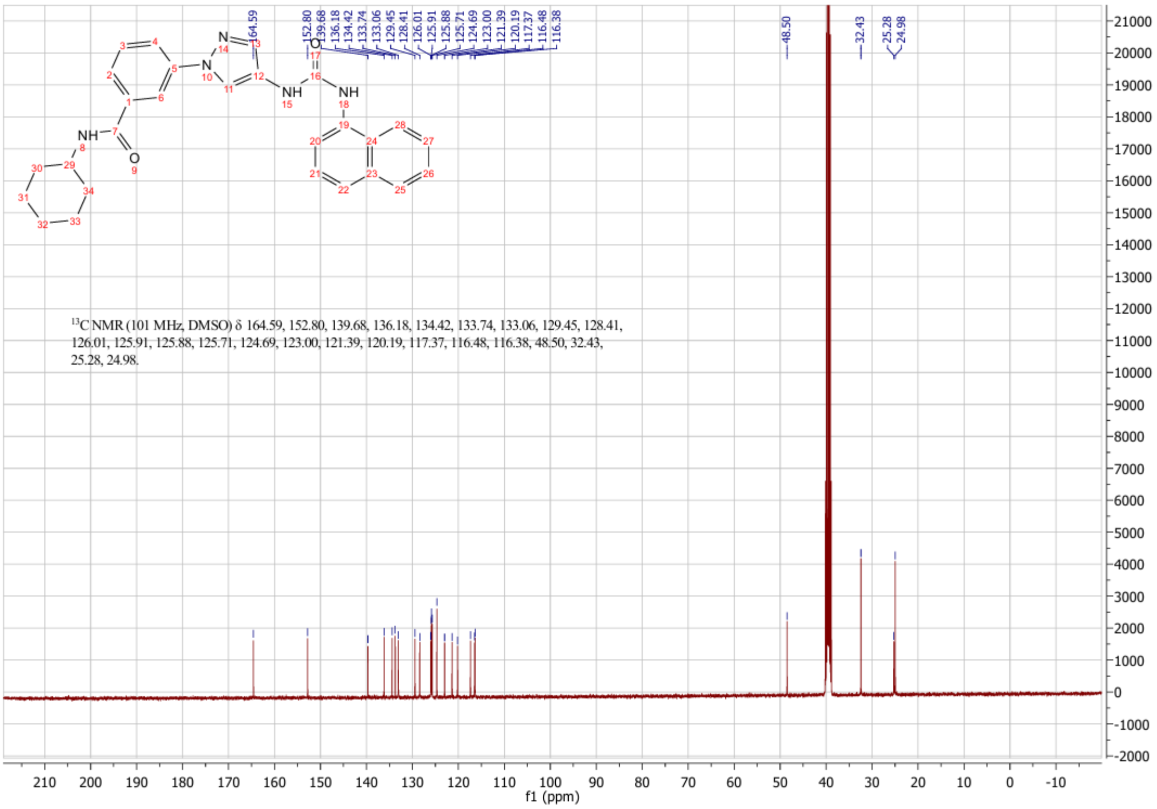
¹³C NMR (101 MHz, DMSO) δ 164.59, 152.80, 139.68, 136.18, 134.42, 133.74, 133.06, 129.45, 128.41, 126.01, 125.91, 125.88, 125.71, 124.69, 123.00, 121.39, 120.19, 117.37, 116.48, 116.38, 48.50, 32.43, 25.28, 24.98.
f1 (ppm)

21k

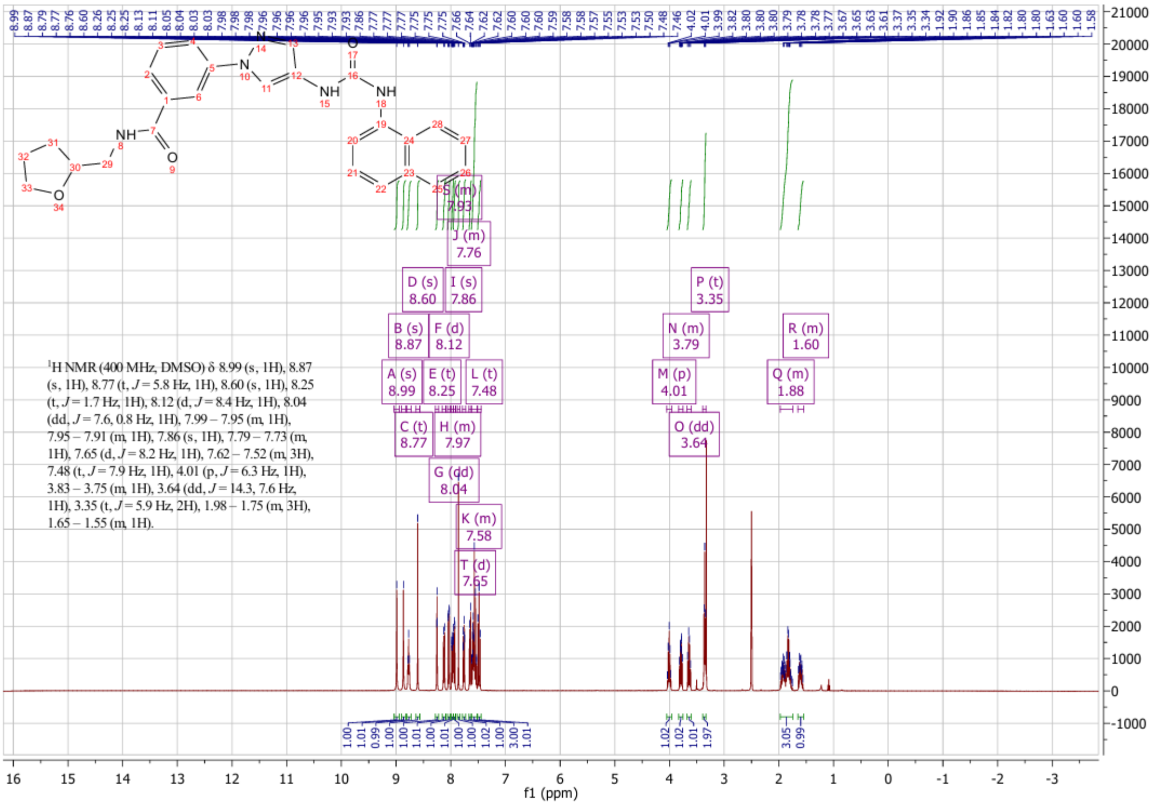
^{1}H NMR (400 MHz, DMSO) δ 8.99 (s, 1H), 8.87 (s, 1H), 8.77 (t, J = 5.8 Hz, 1H), 8.60 (s, 1H), 8.25 (t, J = 1.7 Hz, 1H), 8.12 (d, J = 8.4 Hz, 1H), 8.04 (dd, J = 7.6, 0.8 Hz, 1H), 7.99 – 7.95 (m, 1H), 7.95 – 7.91 (m, 1H), 7.86 (s, 1H), 7.79 – 7.73 (m, 1H), 7.65 (d, J = 8.2 Hz, 1H), 7.62 – 7.52 (m, 3H), 7.48 (t, J = 7.9 Hz, 1H), 4.01 (p, J = 6.3 Hz, 1H), 3.83 – 3.75 (m, 1H), 3.64 (dd, J = 14.3, 7.6 Hz, 1H), 3.35 (t, J = 5.9 Hz, 2H), 1.98 – 1.75 (m, 3H), 1.65 – 1.55 (m, 1H).
f1 (ppm)

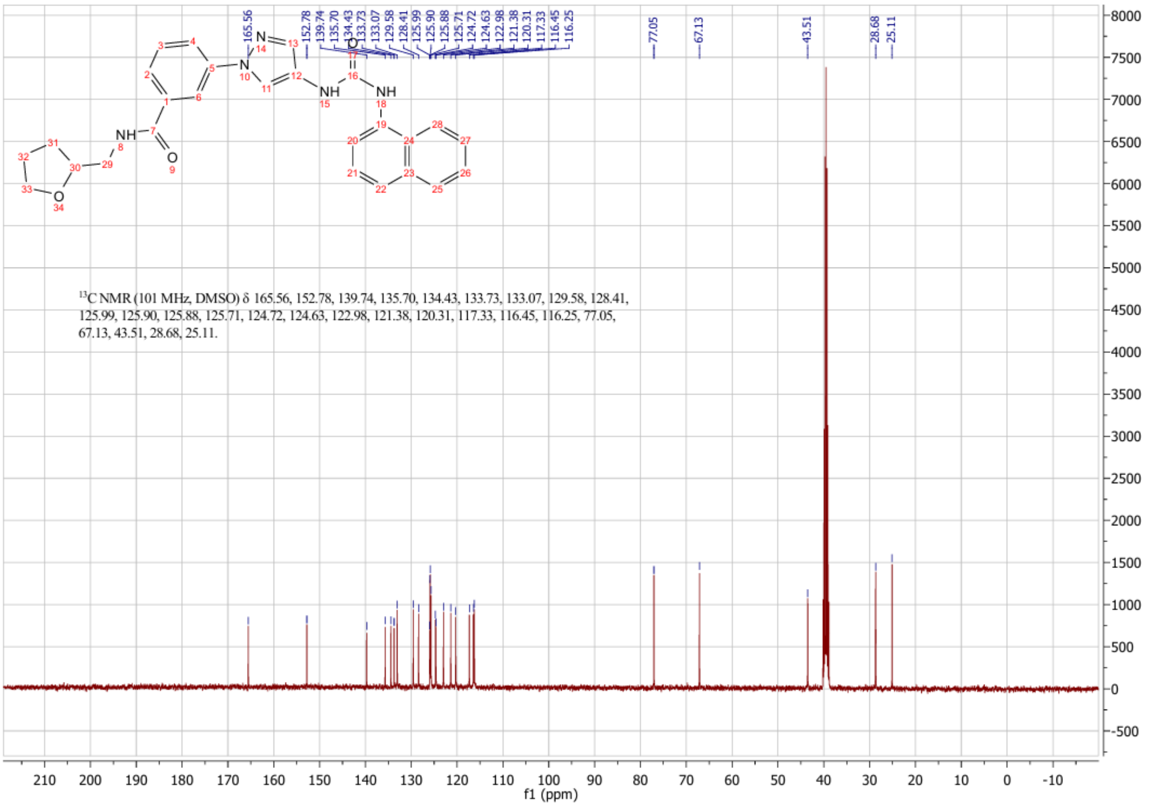
^{13}C NMR (101 MHz, DMSO) δ 165.56, 152.78, 139.74, 135.70, 134.43, 133.73, 133.07, 129.58, 128.41, 125.99, 125.90, 125.88, 125.71, 124.72, 124.63, 122.98, 121.38, 120.31, 117.33, 116.45, 116.25, 77.05, 67.13, 43.51, 28.68, 25.11.
f1 (ppm)

21l

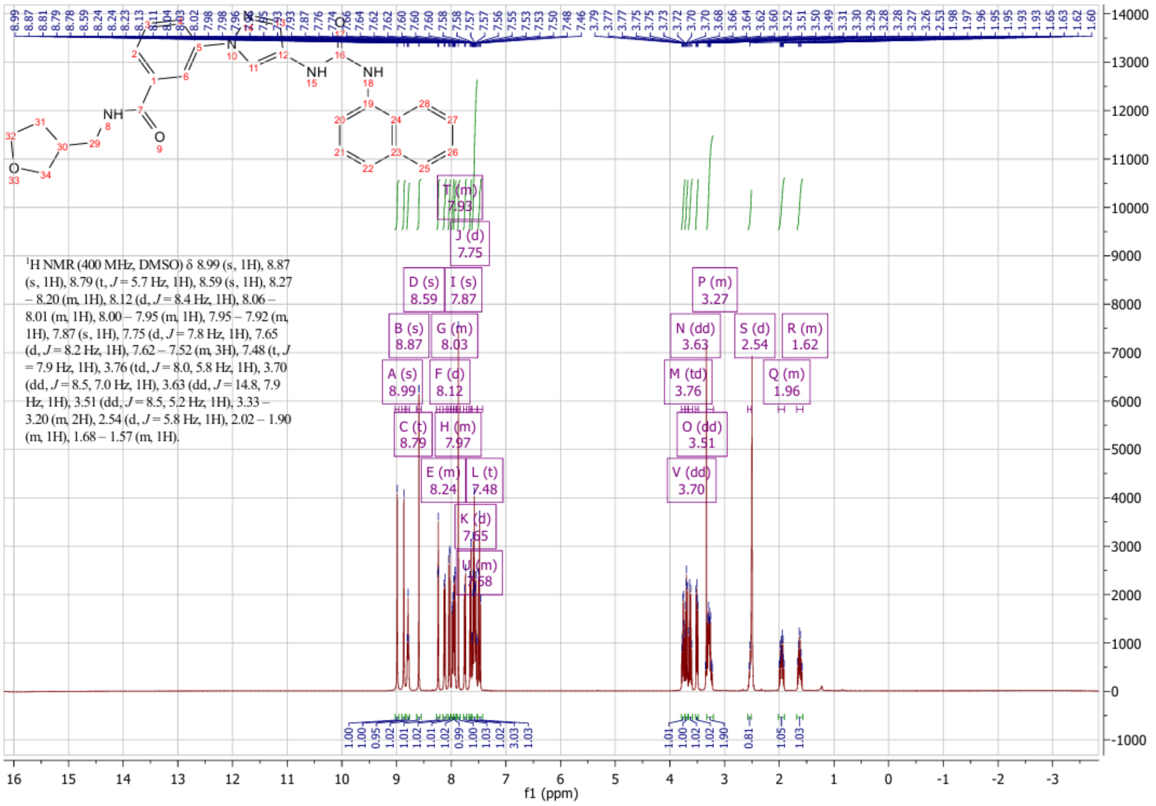
^{1}H NMR (400 MHz, DMSO) δ 8.99 (s, 1H), 8.87 (s, 1H), 8.79 (t, J = 5.7 Hz, 1H), 8.59 (s, 1H), 8.27 – 8.20 (m, 1H), 8.12 (d, J = 8.4 Hz, 1H), 8.06 – 8.01 (m, 1H), 8.00 – 7.95 (m, 1H), 7.95 – 7.92 (m, 1H), 7.87 (s, 1H), 7.75 (d, J = 7.8 Hz, 1H), 7.65 (d, J = 8.2 Hz, 1H), 7.62 – 7.52 (m, 3H), 7.48 (t, J = 7.9 Hz, 1H), 3.76 (td, J = 8.0, 5.8 Hz, 1H), 3.70 (dd, J = 8.5, 7.0 Hz, 1H), 3.63 (dd, J = 14.8, 7.9 Hz, 1H), 3.51 (dd, J = 8.5, 5.2 Hz, 1H), 3.33 – 3.20 (m, 2H), 2.54 (d, J = 5.8 Hz, 1H), 2.02 – 1.90 (m, 1H), 1.68 – 1.57 (m, 1H).
f1 (ppm)

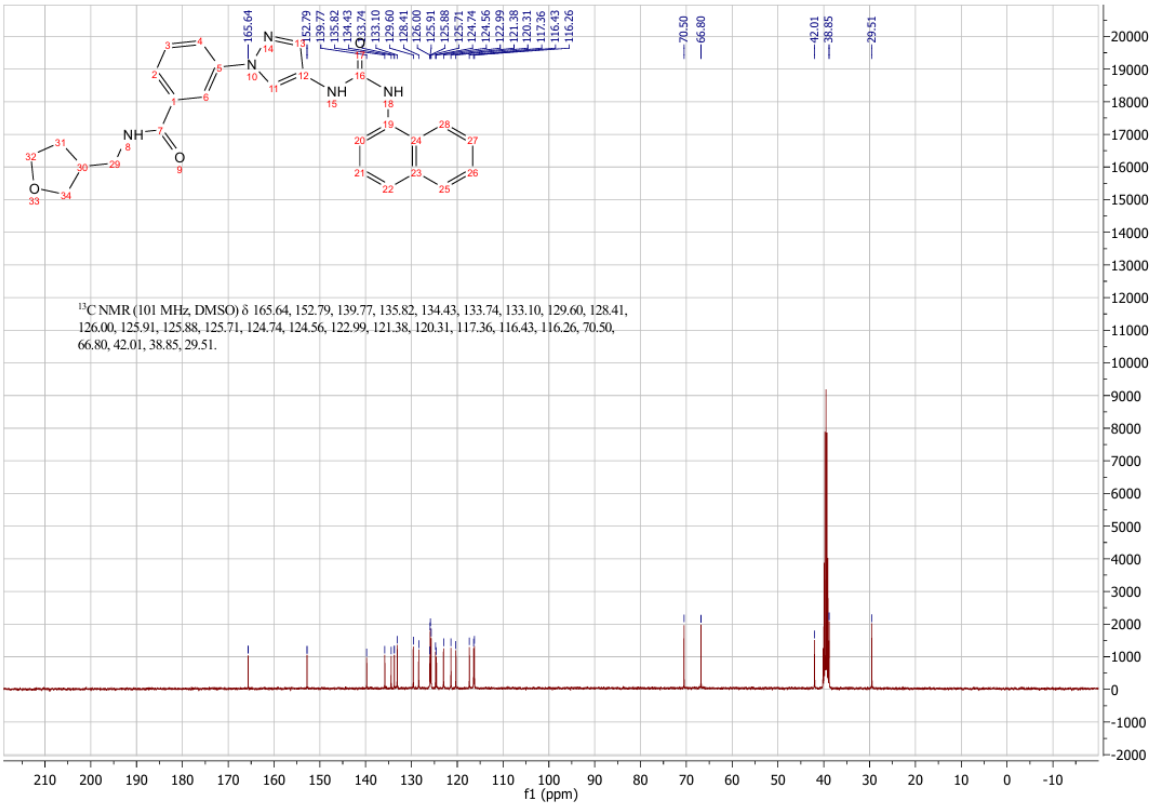
^{13}C NMR (101 MHz, DMSO) δ 165.64, 152.79, 139.77, 135.82, 134.43, 133.74, 133.10, 129.60, 128.41, 126.00, 125.91, 125.88, 125.71, 124.74, 124.56, 122.99, 121.38, 120.31, 117.36, 116.43, 116.26, 70.50, 66.80, 42.01, 38.85, 29.51.
f1 (ppm)

21m

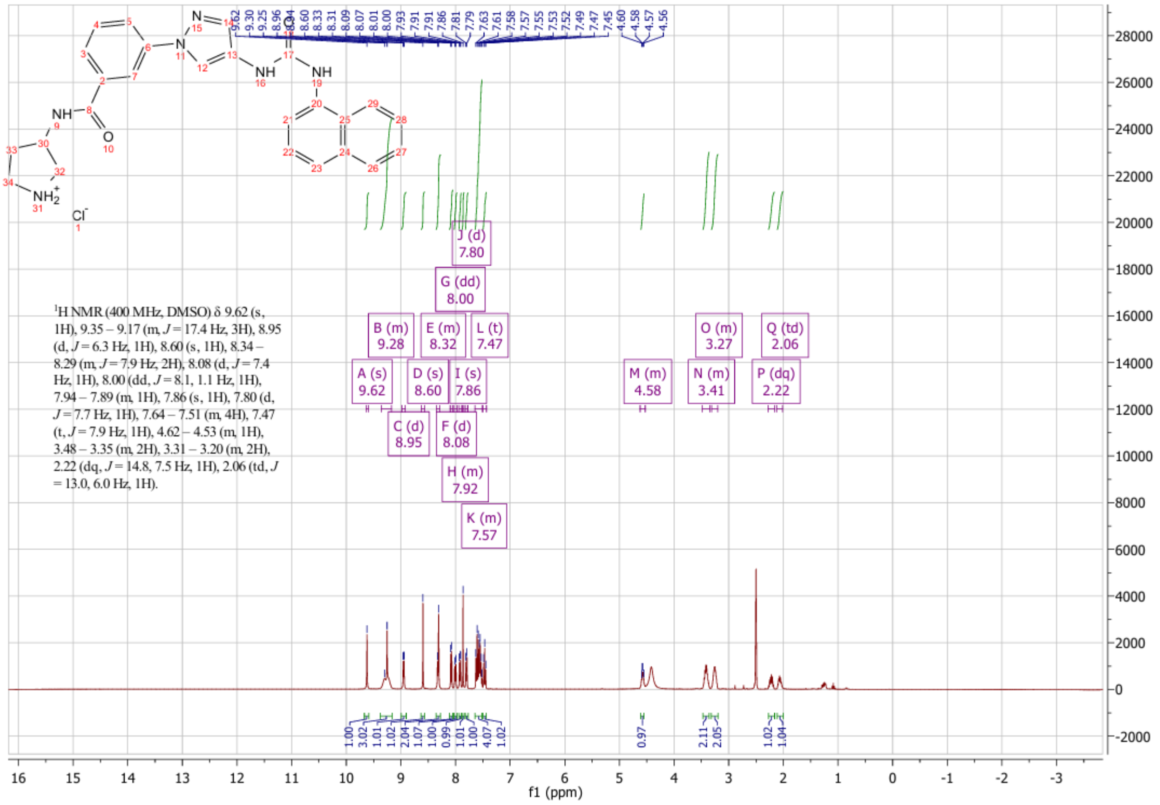
^{1}H NMR (400 MHz, DMSO) δ 9.62 (s, 1H), 9.35 – 9.17 (m, J = 17.4 Hz, 3H), 8.95 (d, J = 6.3 Hz, 1H), 8.60 (s, 1H), 8.34 – 8.29 (m, J = 7.9 Hz, 2H), 8.08 (d, J = 7.4 Hz, 1H), 8.00 (dd, J = 8.1, 1.1 Hz, 1H), 7.94 – 7.89 (m, 1H), 7.86 (s, 1H), 7.80 (d, J = 7.7 Hz, 1H), 7.64 – 7.51 (m, 4H), 7.47 (t, J = 7.9 Hz, 1H), 4.62 – 4.53 (m, 1H), 3.48 – 3.35 (m, 2H), 3.31 – 3.20 (m, 2H), 2.22 (dq, J = 14.8, 7.5 Hz, 1H), 2.06 (td, J = 13.0, 6.0 Hz, 1H).
f1 (ppm)

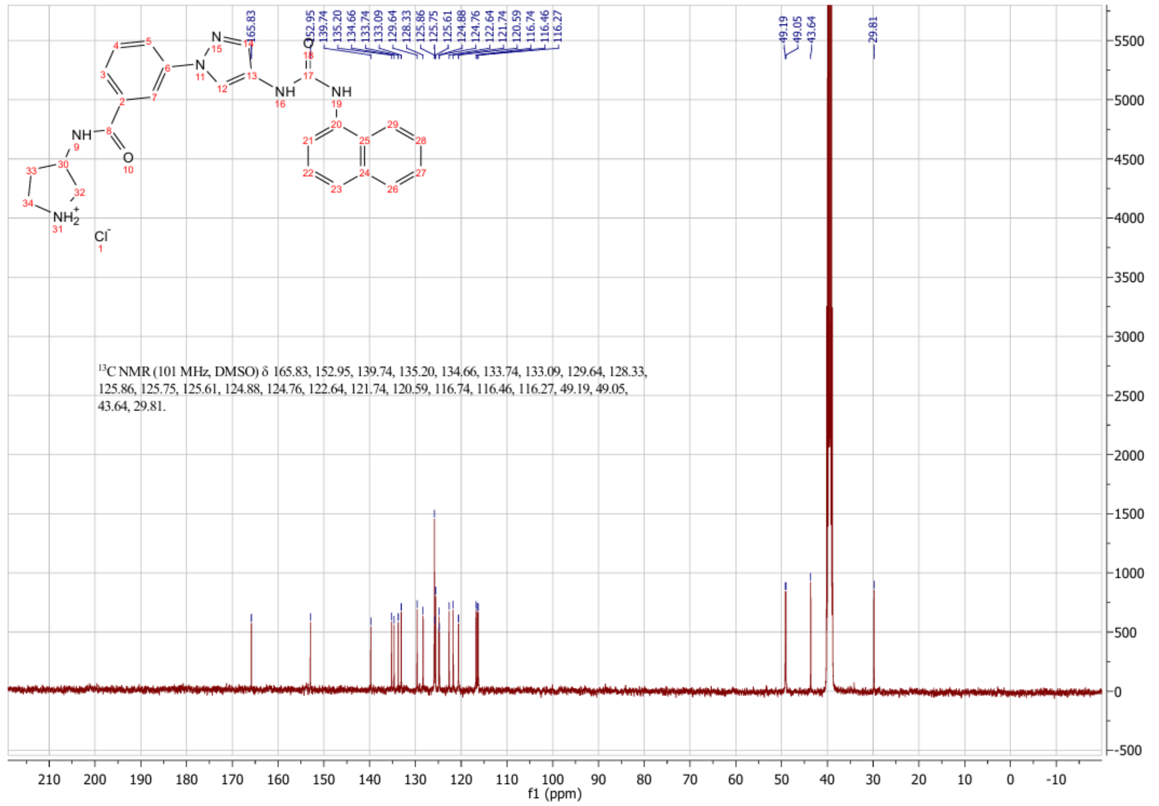
^{13}C NMR (101 MHz, DMSO) δ 165.83, 152.95, 139.74, 135.20, 134.66, 133.74, 133.09, 129.64, 128.33, 125.86, 125.75, 125.61, 124.88, 124.76, 122.64, 121.74, 120.59, 116.74, 116.46, 116.27, 49.19, 49.05, 43.64, 29.81.
f1 (ppm)

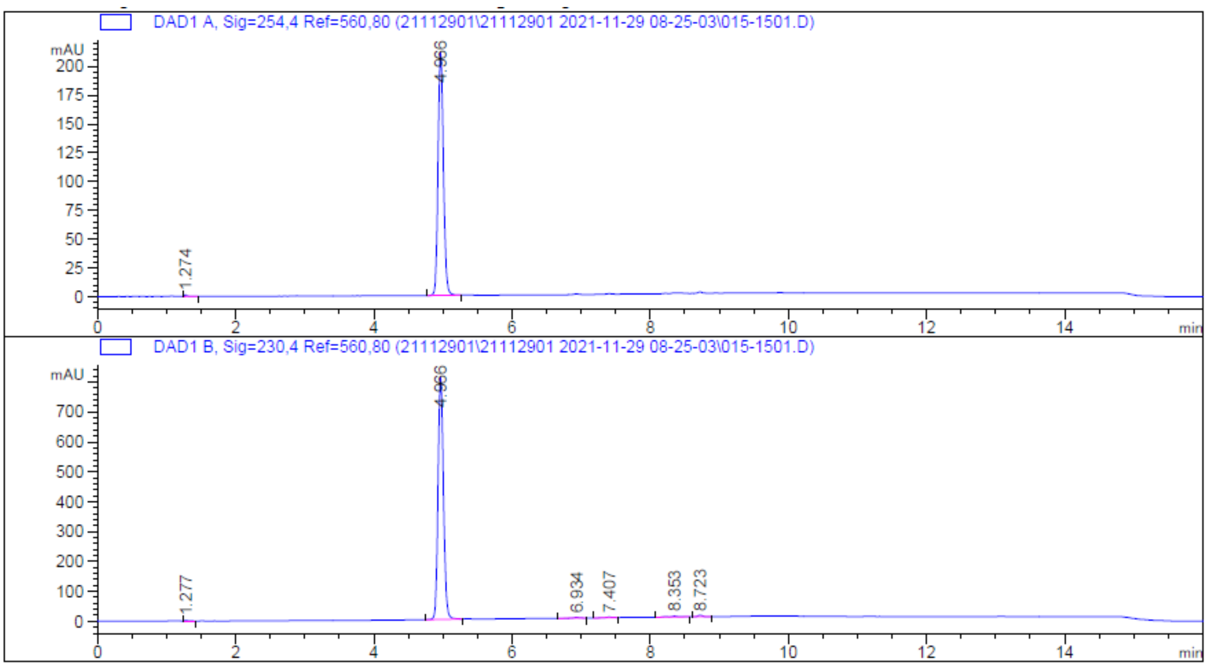

==

Area Percent Report

==

Sorted By : Signal
Multiplier: : 1.0000
Dilution: : 1.0000
Use Multiplier & Dilution Factor with ISTDs

Signal 1: DAD1 A, Sig=254,4 Ref=560,80

Peak #	RetTime [min]	Type	Width [min]	Area [mAU*s]	Height [mAU]	Area %
1	1.274	BB	0.0477	4.45176	1.45533	0.3898
2	4.966	BB	0.0848	1137.65002	211.18155	99.6102

Totals : 1142.10178 212.63688

Signal 2: DAD1 B, Sig=230,4 Ref=560,80

Peak #	RetTime [min]	Type	Width [min]	Area [mAU*s]	Height [mAU]	Area %
1	1.277	BB	0.0496	6.59538	2.04764	0.1475
2	4.966	BB	0.0852	4385.72754	810.17297	98.0628
3	6.934	BB	0.0986	14.46465	2.14830	0.3234
4	7.407	BB	0.0812	12.99227	2.39926	0.2905
5	8.353	BB	0.1257	28.85776	3.06861	0.6452
6	8.723	BB	0.0749	23.72775	4.86448	0.5305

Totals : 4472.36535 824.70128

==

*** End of Report ***

21n

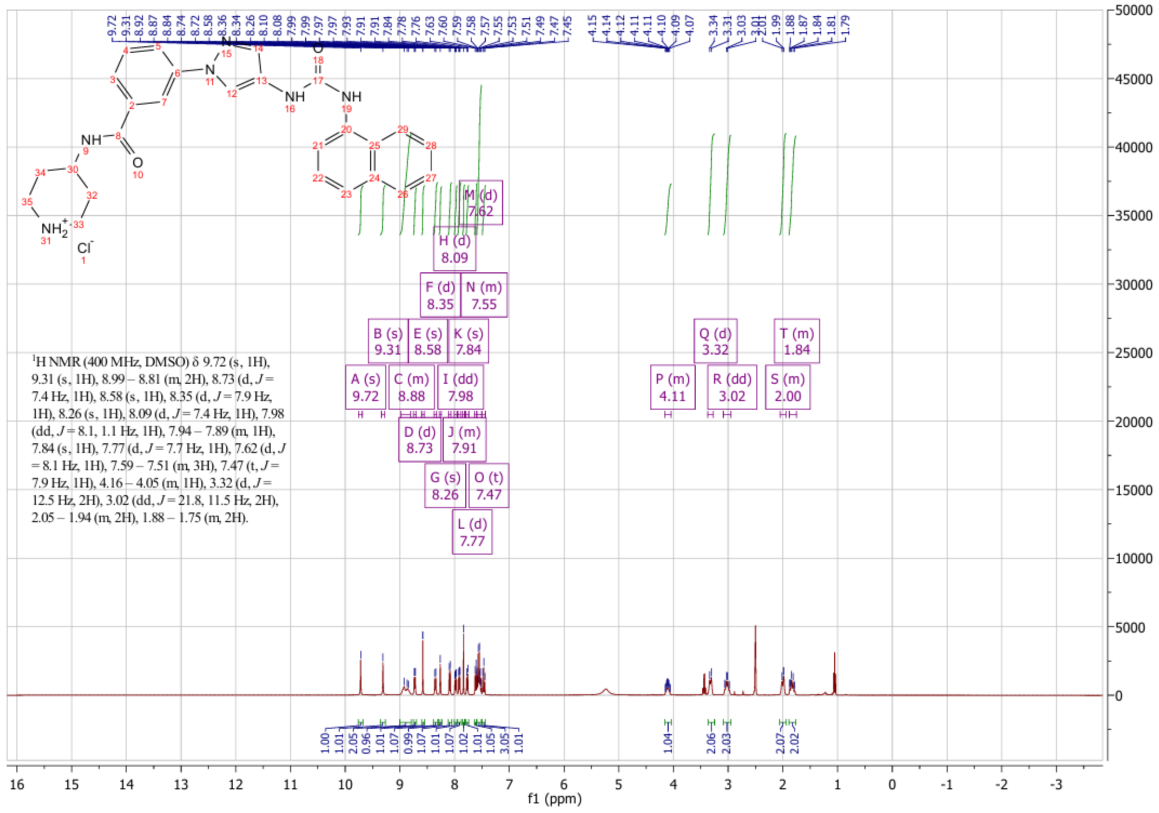

^{1}H NMR (400 MHz, DMSO) δ 9.72 (s, 1H), 9.31 (s, 1H), 8.99 – 8.81 (m, 2H), 8.73 (d, J = 7.4 Hz, 1H), 8.58 (s, 1H), 8.35 (d, J = 7.9 Hz, 1H), 8.26 (s, 1H), 8.09 (d, J = 7.4 Hz, 1H), 7.98 (dd, J = 8.1, 1.1 Hz, 1H), 7.94 – 7.89 (m, 1H), 7.84 (s, 1H), 7.77 (d, J = 7.7 Hz, 1H), 7.62 (d, J = 8.1 Hz, 1H), 7.59 – 7.51 (m, 3H), 7.47 (t, J = 7.9 Hz, 1H), 4.16 – 4.05 (m, 1H), 3.32 (d, J = 12.5 Hz, 2H), 3.02 (dd, J = 21.8, 11.5 Hz, 2H), 2.05 – 1.94 (m, 2H), 1.88 – 1.75 (m, 2H).
f1 (ppm)

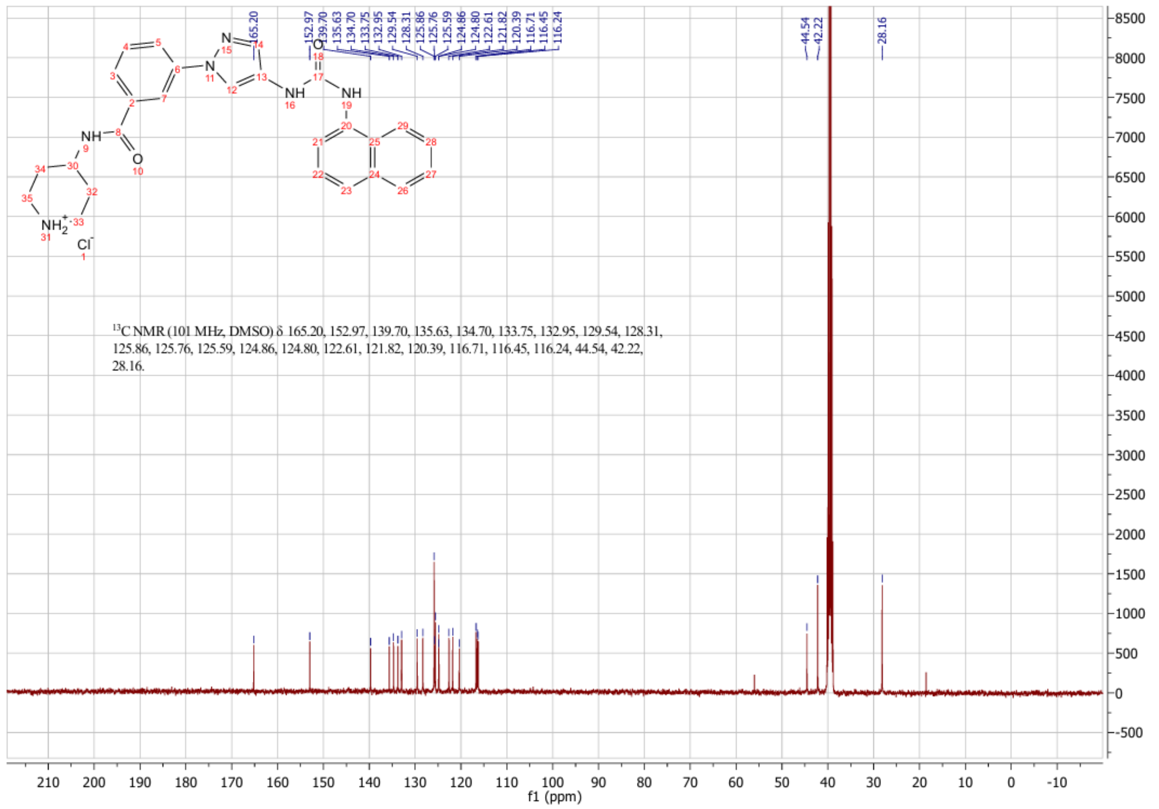

^{13}C NMR (101 MHz, DMSO) δ 165.20, 152.97, 139.70, 135.63, 134.70, 133.75, 132.95, 129.54, 128.31, 125.86, 125.76, 125.59, 124.86, 124.80, 122.61, 121.82, 120.39, 116.71, 116.45, 116.24, 44.54, 42.22, 28.16.
f1 (ppm)

21o

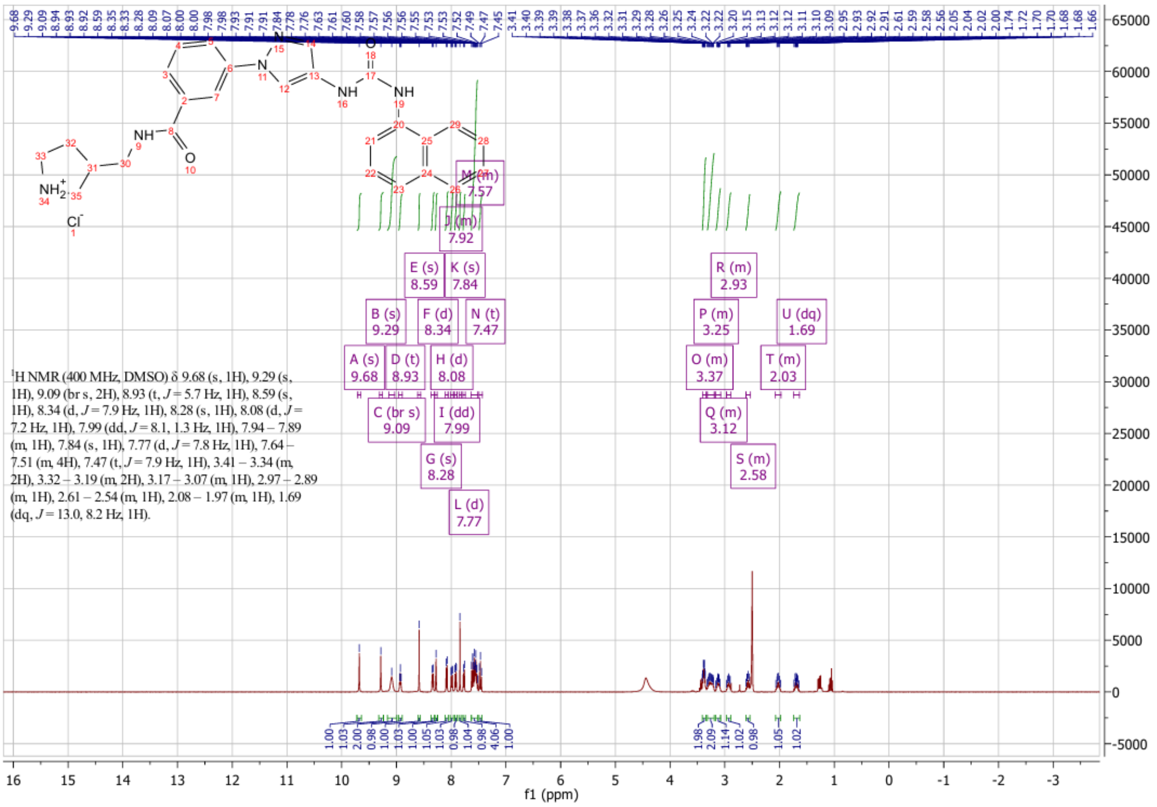

^{1}H NMR (400 MHz, DMSO) δ 9.68 (s, 1H), 9.29 (s, 1H), 9.09 (br s, 2H), 8.93 (t, J = 5.7 Hz, 1H), 8.59 (s, 1H), 8.34 (d, J = 7.9 Hz, 1H), 8.28 (s, 1H), 8.08 (d, J = 7.2 Hz, 1H), 7.99 (dd, J = 8.1, 1.3 Hz, 1H), 7.94 – 7.89 (m, 1H), 7.84 (s, 1H), 7.77 (d, J = 7.8 Hz, 1H), 7.64 – 7.51 (m, 4H), 7.47 (t, J = 7.9 Hz, 1H), 3.41 – 3.34 (m, 2H), 3.32 – 3.19 (m, 2H), 3.17 – 3.07 (m, 1H), 2.97 – 2.89 (m, 1H), 2.61 – 2.54 (m, 1H), 2.08 – 1.97 (m, 1H), 1.69 (dq, J = 13.0, 8.2 Hz, 1H).
f1 (ppm)

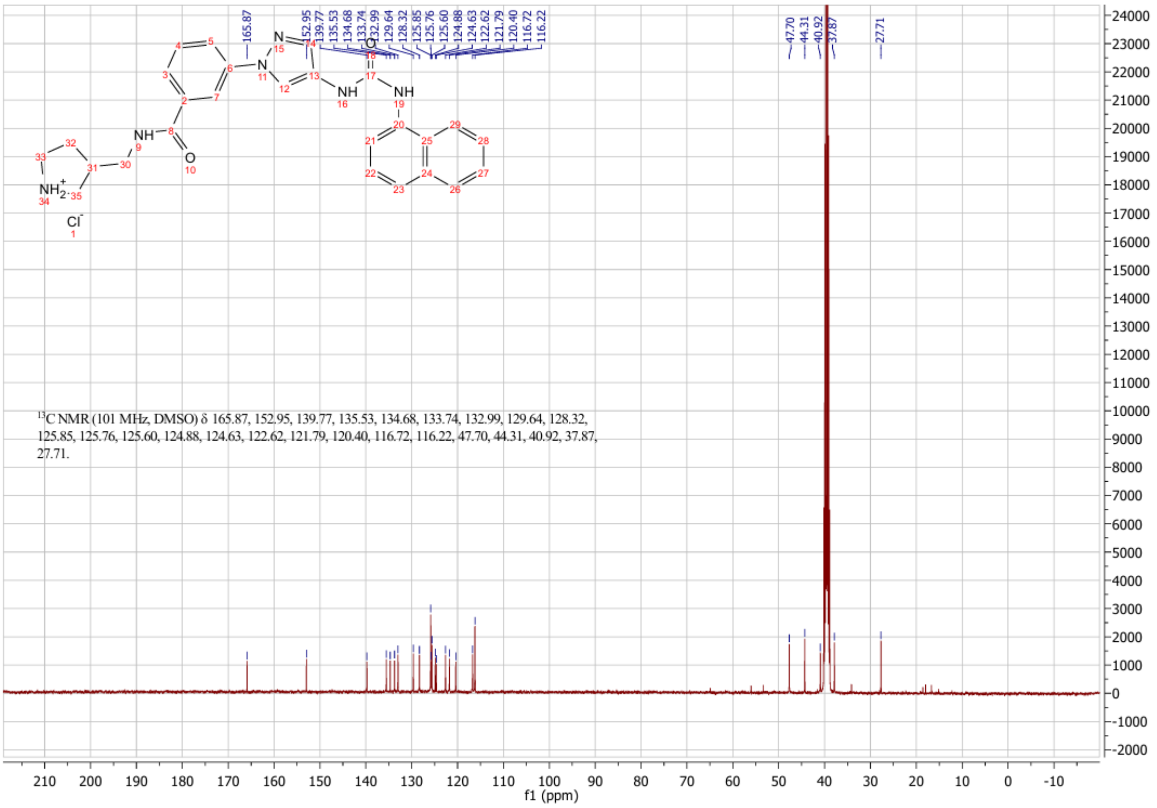

^{13}C NMR (101 MHz, DMSO) δ 165.87, 152.95, 139.77, 135.53, 134.68, 133.74, 132.99, 129.64, 128.32, 125.85, 125.76, 125.60, 124.88, 124.63, 122.62, 121.79, 120.40, 116.72, 116.22, 47.70, 44.31, 40.92, 37.87, 27.71.
f1 (ppm)

21p

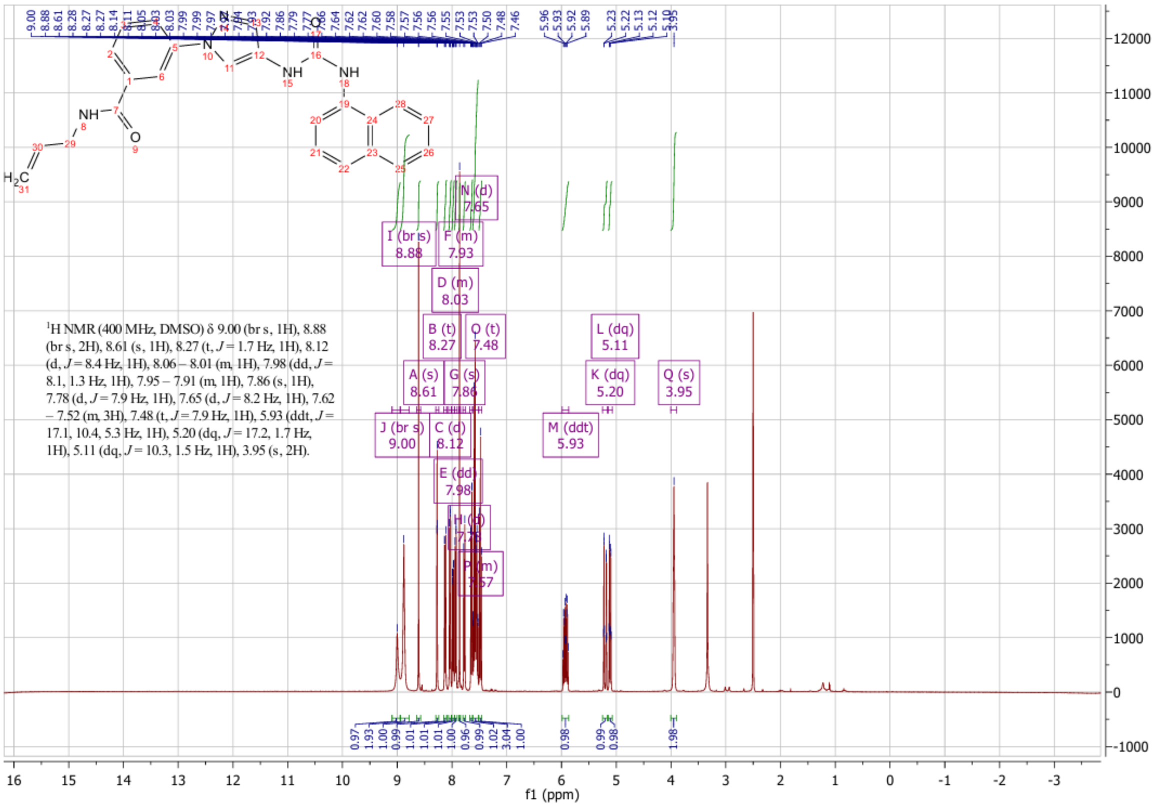

¹H NMR (400 MHz, DMSO) δ 9.00 (br s, 1H), 8.88 (br s, 2H), 8.61 (s, 1H), 8.27 (t, J = 1.7 Hz, 1H), 8.12 (d, J = 8.4 Hz, 1H), 8.06 – 8.01 (m, 1H), 7.98 (dd, J = 8.1, 1.3 Hz, 1H), 7.95 – 7.91 (m, 1H), 7.86 (s, 1H), 7.78 (d, J = 7.9 Hz, 1H), 7.65 (d, J = 8.2 Hz, 1H), 7.62 – 7.52 (m, 3H), 7.48 (t, J = 7.9 Hz, 1H), 5.93 (ddt, J = 17.1, 10.4, 5.3 Hz, 1H), 5.20 (dq, J = 17.2, 1.7 Hz, 1H), 5.11 (dq, J = 10.3, 1.5 Hz, 1H), 3.95 (s, 2H).
f1 (ppm)

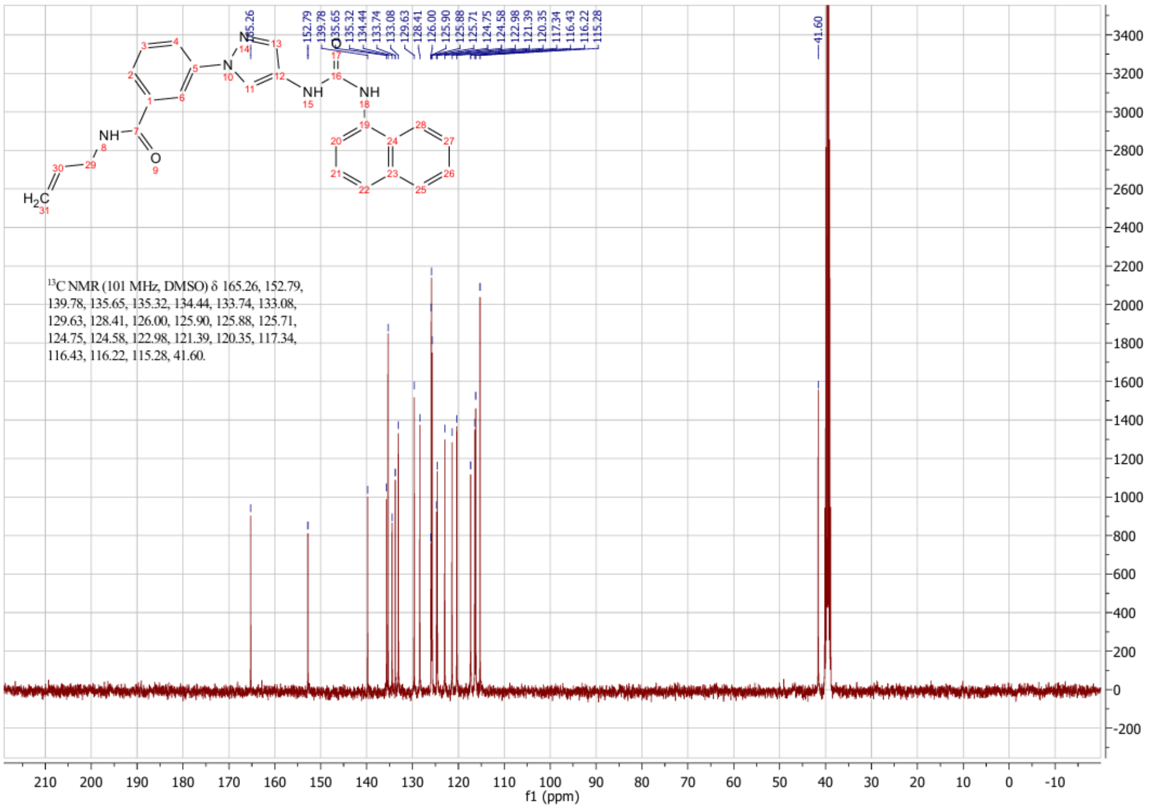

¹³C NMR (101 MHz, DMSO) δ 165.26, 152.79, 139.78, 135.65, 135.32, 134.44, 133.74, 133.08, 129.63, 128.41, 126.00, 125.90, 125.88, 125.71, 124.75, 124.58, 122.98, 121.39, 120.35, 117.34, 116.43, 116.22, 115.28, 41.60.
f1 (ppm)

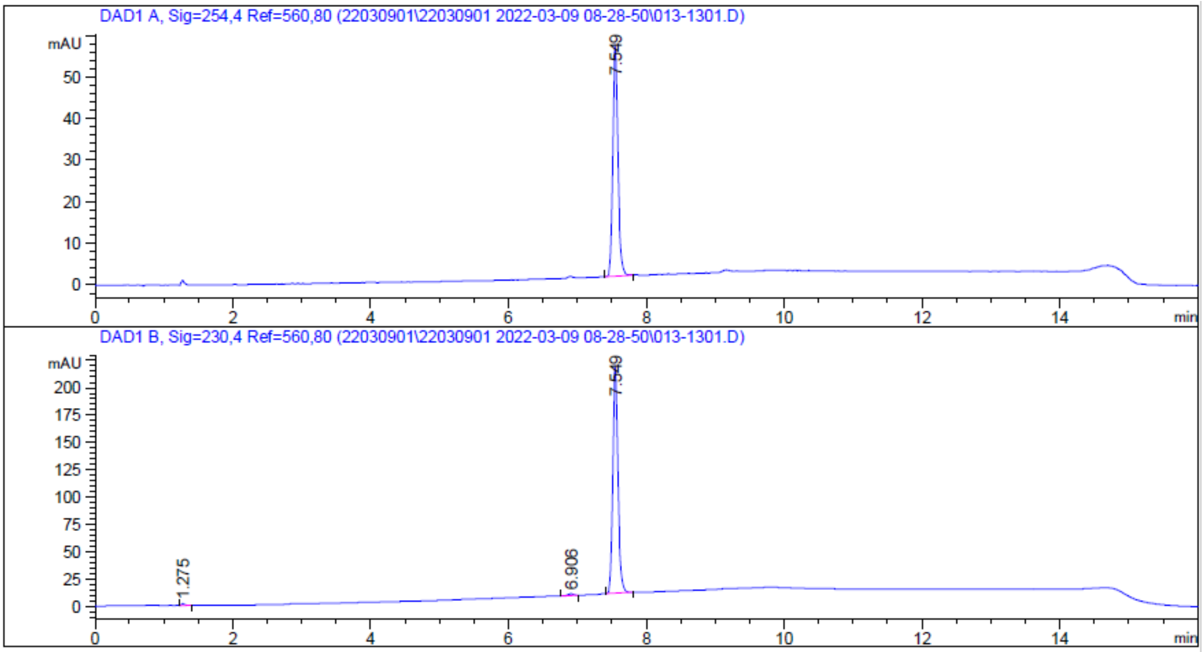

Area Percent Report

Sorted By : Signal
Multiplier: : 1.0000
Dilution: : 1.0000
Use Multiplier & Dilution Factor with ISTDs

Signal 1: DAD1 A, Sig=254,4 Ref=560,80

Peak #	RetTime [min]	Type	Width [min]	Area [mAU*s]	Height [mAU]	Area %
1	7.549	BB	0.0788	288.88608	55.40992	100.0000

Totals : 288.88608 55.40992

Signal 2: DAD1 B, Sig=230,4 Ref=560,80

Peak #	RetTime [min]	Type	Width [min]	Area [mAU*s]	Height [mAU]	Area %
1	1.275	BB	0.0510	5.79895	1.73279	0.5297
2	6.906	BB	0.0828	9.11469	1.69207	0.8326
3	7.549	BB	0.0788	1079.75574	207.21355	98.6376

Totals : 1094.66938 210.63840

*** End of Report ***

21q

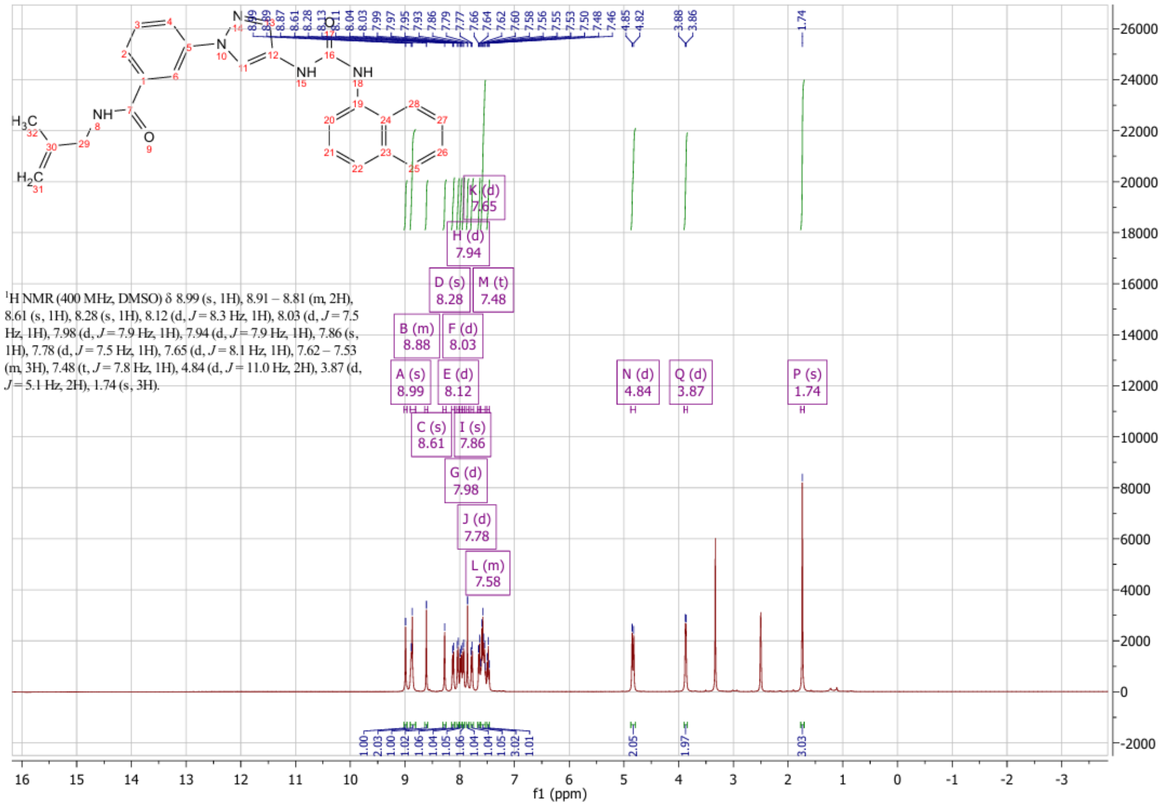

^{1}H NMR (400 MHz, DMSO) δ 8.99 (s, 1H), 8.91 – 8.81 (m, 2H), 8.61 (s, 1H), 8.28 (s, 1H), 8.12 (d, J = 8.3 Hz, 1H), 8.03 (d, J = 7.5 Hz, 1H), 7.98 (d, J = 7.9 Hz, 1H), 7.94 (d, J = 7.9 Hz, 1H), 7.86 (s, 1H), 7.78 (d, J = 7.5 Hz, 1H), 7.65 (d, J = 8.1 Hz, 1H), 7.62 – 7.53 (m, 3H), 7.48 (t, J = 7.8 Hz, 1H), 4.84 (d, J = 11.0 Hz, 2H), 3.87 (d, J = 5.1 Hz, 2H), 1.74 (s, 3H).
f1 (ppm)

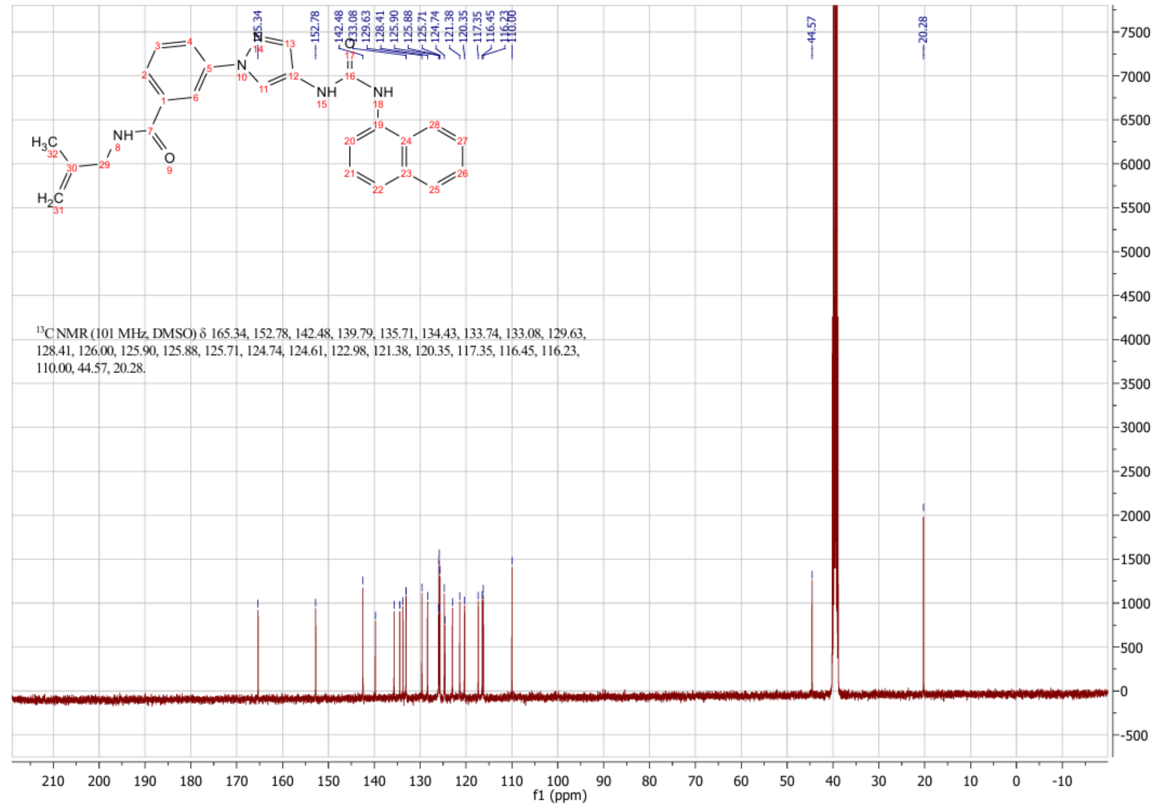

^{13}C NMR (101 MHz, DMSO) δ 165.34, 152.78, 142.48, 139.79, 135.71, 134.43, 133.74, 133.08, 129.63, 128.41, 126.00, 125.90, 125.88, 125.71, 124.74, 124.61, 122.98, 121.38, 120.35, 117.35, 116.45, 116.23, 110.00, 44.57, 20.28.
f1 (ppm)

21r

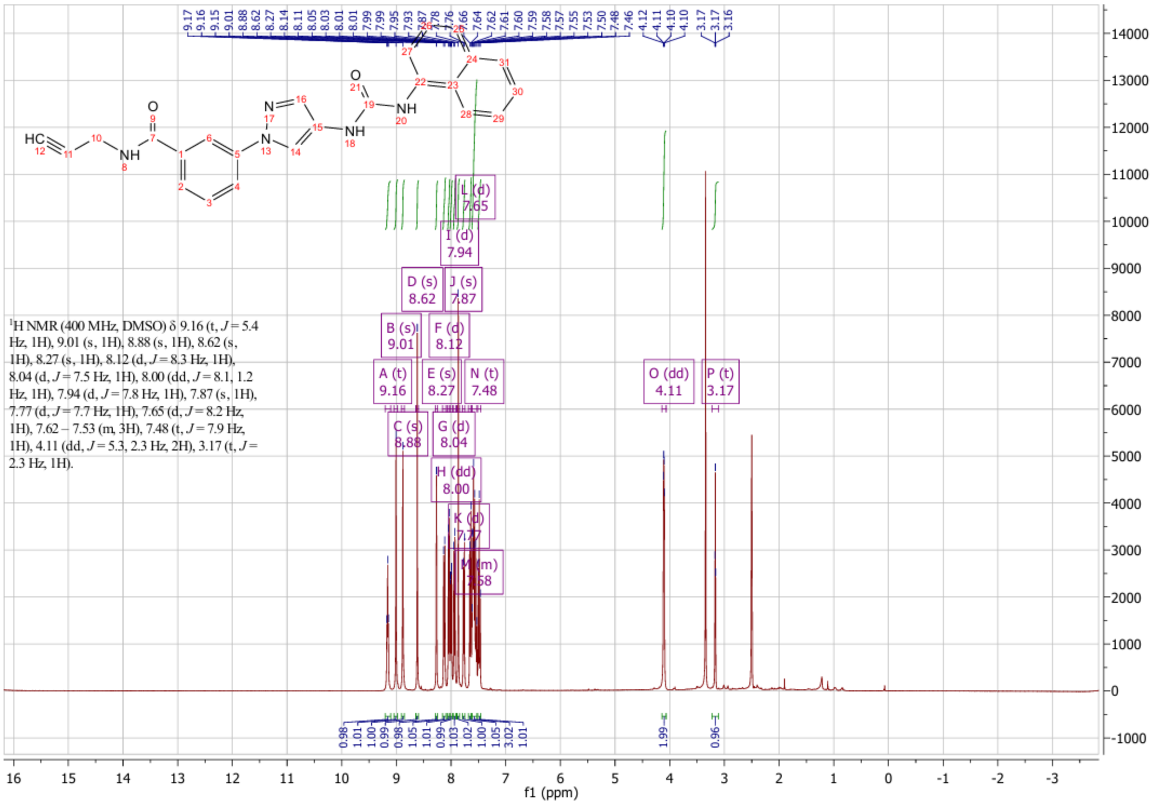

^{1}H NMR (400 MHz, DMSO) δ 9.16 (t, J = 5.4 Hz, 1H), 9.01 (s, 1H), 8.88 (s, 1H), 8.62 (s, 1H), 8.27 (s, 1H), 8.12 (d, J = 8.3 Hz, 1H), 8.04 (d, J = 7.5 Hz, 1H), 8.00 (dd, J = 8.1, 1.2 Hz, 1H), 7.94 (d, J = 7.8 Hz, 1H), 7.87 (s, 1H), 7.77 (d, J = 7.7 Hz, 1H), 7.65 (d, J = 8.2 Hz, 1H), 7.62 – 7.53 (m, 3H), 7.48 (t, J = 7.9 Hz, 1H), 4.11 (dd, J = 5.3, 2.3 Hz, 2H), 3.17 (t, J = 2.3 Hz, 1H).
f1 (ppm)

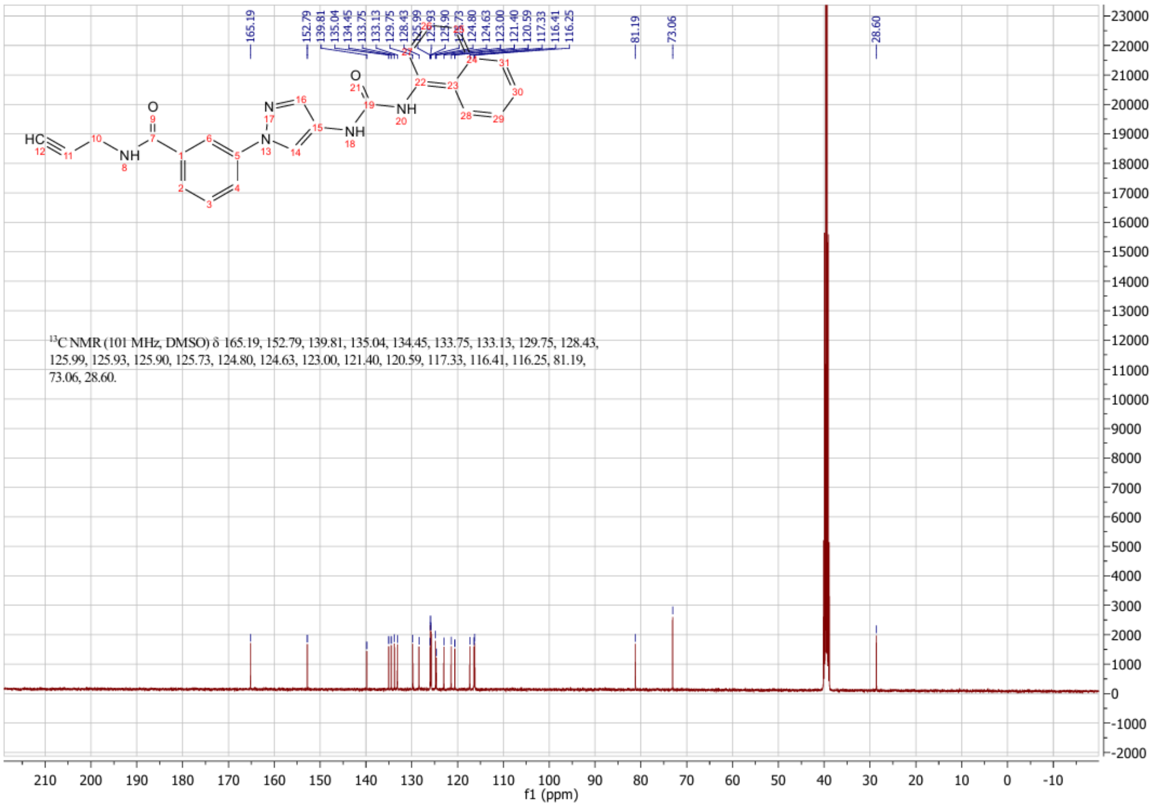

^{13}C NMR (101 MHz, DMSO) δ 165.19, 152.79, 139.81, 135.04, 134.45, 133.75, 133.13, 129.75, 128.43, 125.99, 125.93, 125.90, 125.73, 124.80, 124.63, 123.00, 121.40, 120.59, 117.33, 116.41, 116.25, 81.19, 73.06, 28.60.
f1 (ppm)

11.4 Additional Compounds

SI-I

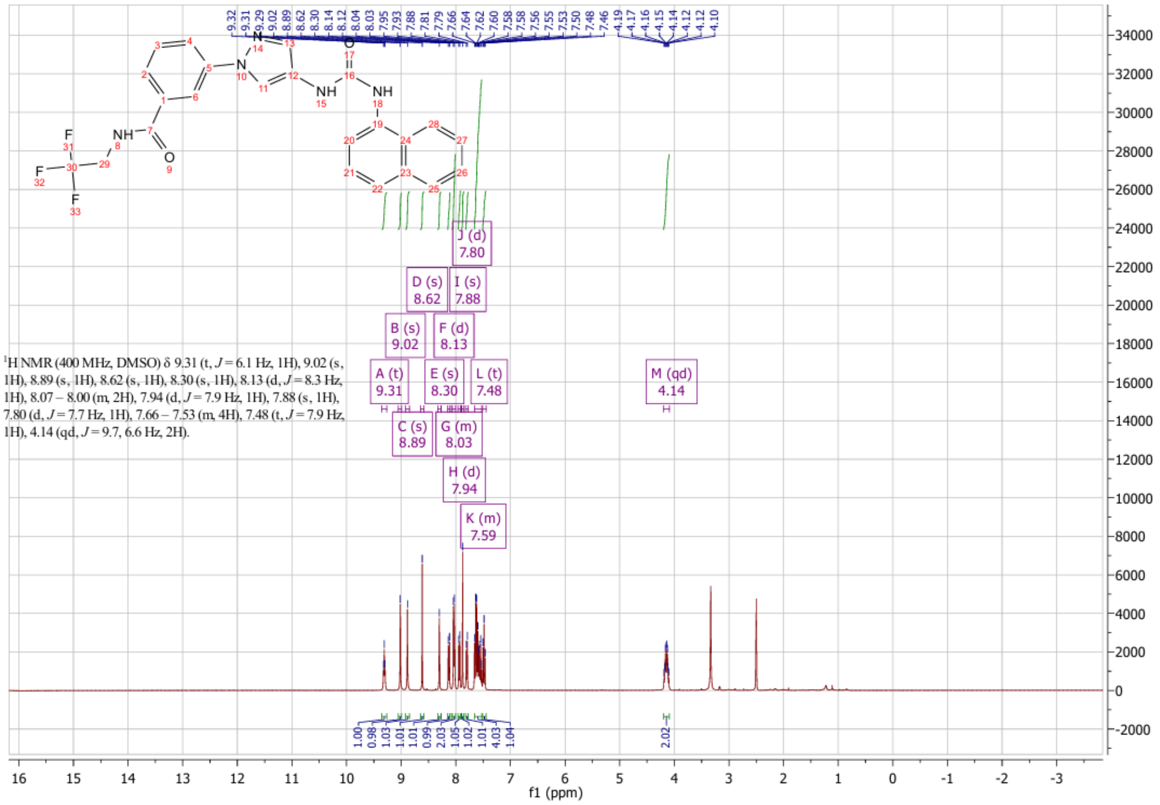

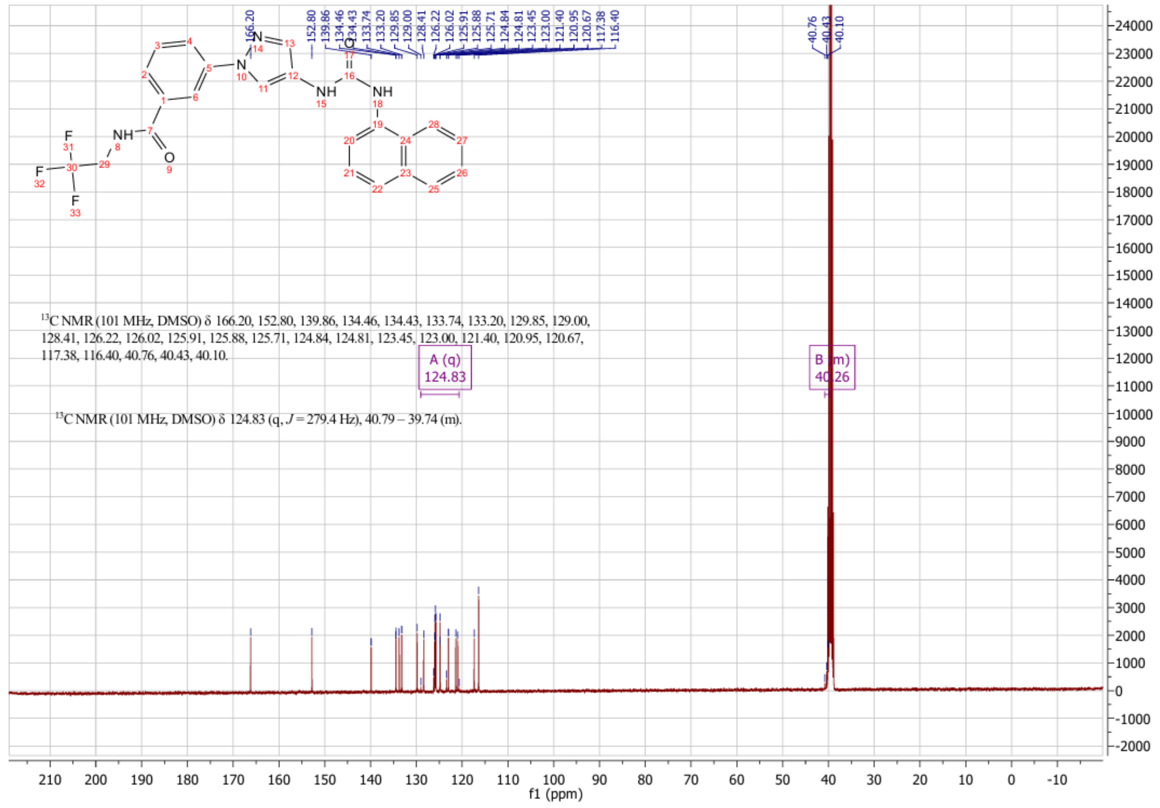

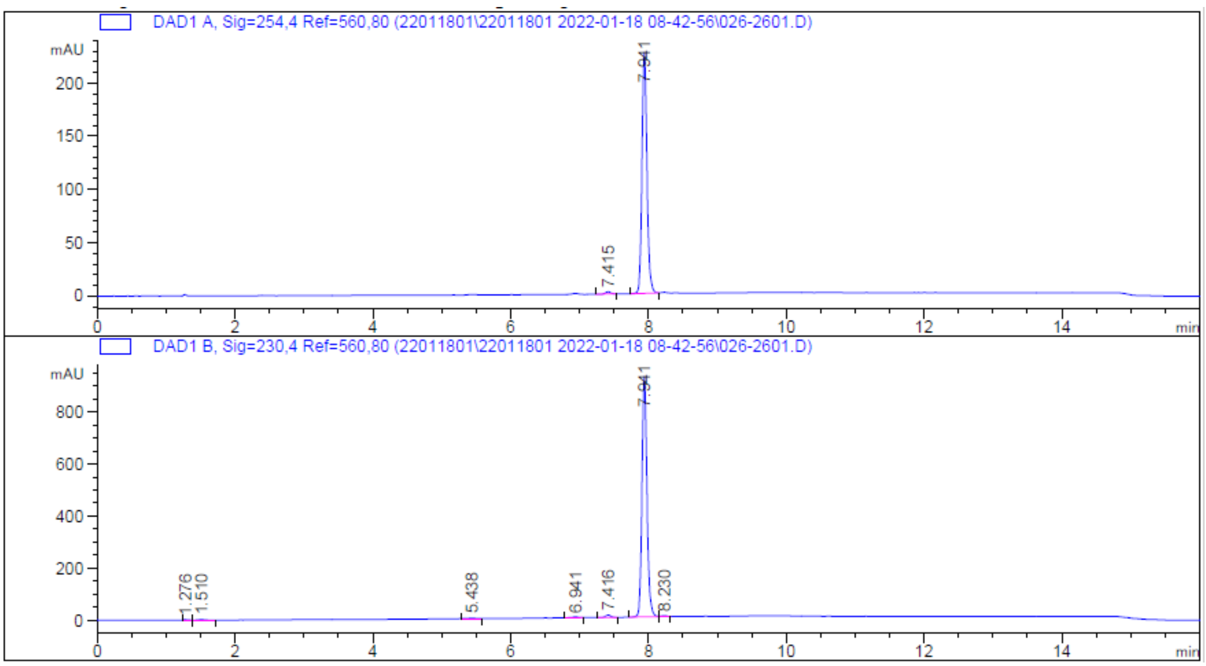

===

Area Percent Report

===

Sorted By : Signal
Multiplier: : 1.0000
Dilution: : 1.0000
Use Multiplier & Dilution Factor with ISTDs

Signal 1: DAD1 A, Sig=254,4 Ref=560,80

Peak #	RetTime [min]	Type	Width [min]	Area [mAU*s]	Height [mAU]	Area %
1	7.415	BB	0.0827	11.55360	2.15062	1.0103
2	7.941	BB	0.0763	1132.05371	226.58446	98.9897

Totals : 1143.60732 228.73508

Signal 2: DAD1 B, Sig=230,4 Ref=560,80

Peak #	RetTime [min]	Type	Width [min]	Area [mAU*s]	Height [mAU]	Area %
1	1.276	BB	0.0498	6.26650	1.93270	0.1316
2	1.510	BB	0.0689	9.89418	2.18404	0.2077
3	5.438	BB	0.1156	15.80409	2.20481	0.3318
4	6.941	BB	0.0846	21.20066	3.71162	0.4451
5	7.416	BB	0.0818	48.67915	9.19412	1.0220
6	7.941	BV	0.0766	4642.66602	923.78247	97.4748
7	8.230	VB	0.0759	18.42763	3.71563	0.3869

Totals : 4762.93822 946.72540

===

*** End of Report ***

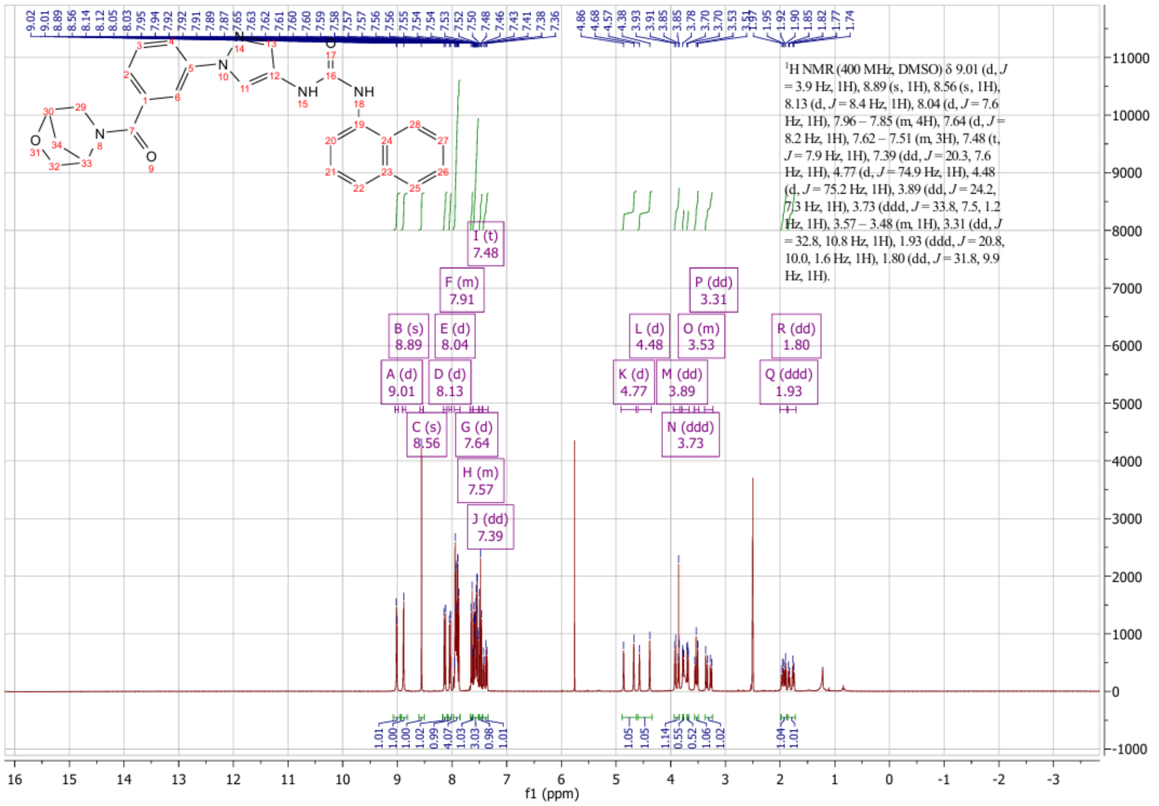
1H NMR (400 MHz, DMSO) δ 9.01 (d, J = 3.9 Hz, 1H), 8.89 (s, 1H), 8.56 (s, 1H), 8.13 (d, J = 8.4 Hz, 1H), 8.04 (d, J = 7.6 Hz, 1H), 7.96 – 7.85 (m, 4H), 7.64 (d, J = 8.2 Hz, 1H), 7.62 – 7.51 (m, 3H), 7.48 (t, J = 7.9 Hz, 1H), 7.39 (dd, J = 20.3, 7.6 Hz, 1H), 4.77 (d, J = 74.9 Hz, 1H), 4.48 (d, J = 75.2 Hz, 1H), 3.89 (dd, J = 24.2, 7.3 Hz, 1H), 3.73 (ddd, J = 33.8, 7.5, 1.2 Hz, 1H), 3.57 – 3.48 (m, 1H), 3.31 (dd, J = 32.8, 10.8 Hz, 1H), 1.93 (ddd, J = 20.8, 10.0, 1.6 Hz, 1H), 1.80 (dd, J = 31.8, 9.9 Hz, 1H).
f1 (ppm)

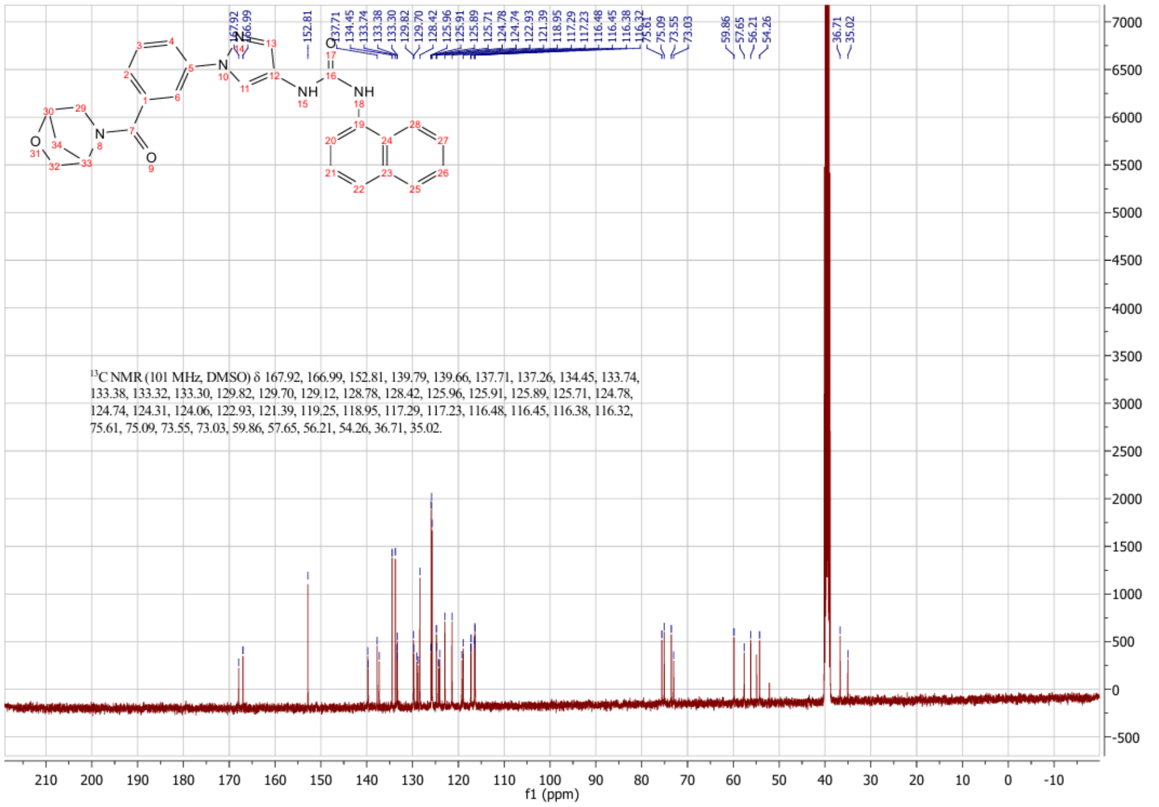
13C NMR (101 MHz, DMSO) δ 167.92, 166.99, 152.81, 139.79, 139.66, 137.71, 137.26, 134.45, 133.74, 133.38, 133.32, 133.30, 129.82, 129.70, 129.12, 128.78, 128.42, 125.96, 125.91, 125.89, 125.71, 124.78, 124.74, 124.31, 124.06, 122.93, 121.39, 119.25, 118.95, 117.29, 117.23, 116.48, 116.45, 116.38, 116.32, 75.61, 75.09, 73.55, 73.03, 59.86, 57.65, 56.21, 54.26, 36.71, 35.02.
f1 (ppm)

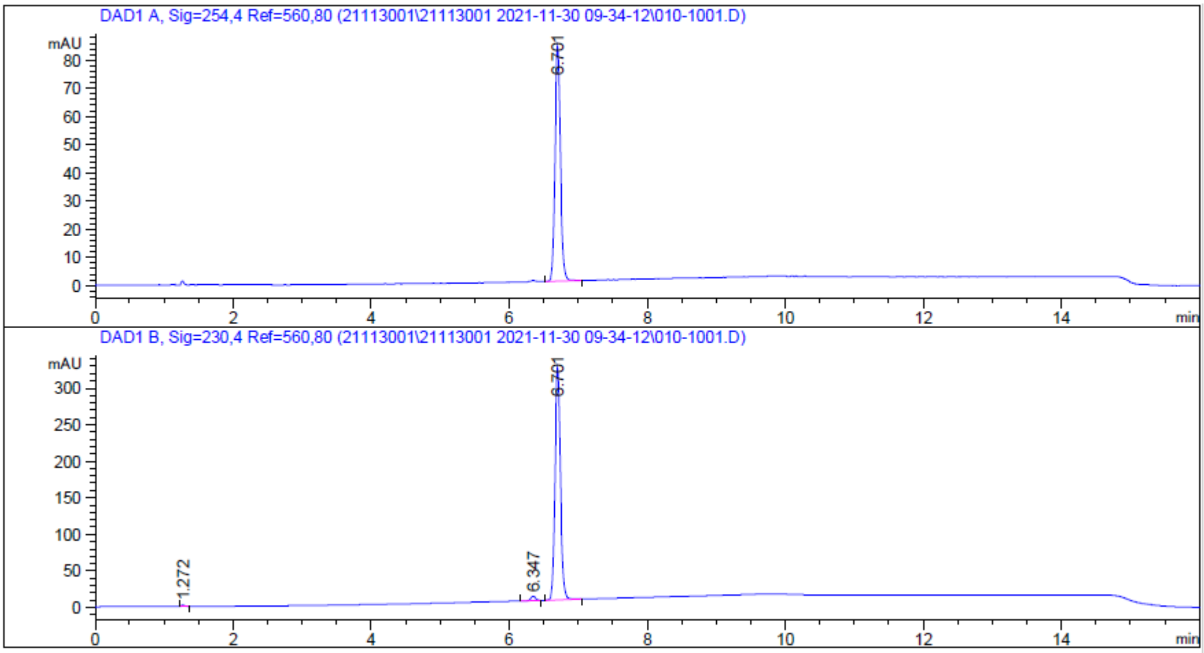

===

Area Percent Report

===

Sorted By : Signal
Multiplier: : 1.0000
Dilution: : 1.0000
Use Multiplier & Dilution Factor with ISTDs

Signal 1: DAD1 A, Sig=254,4 Ref=560,80

Peak #	RetTime [min]	Type	Width [min]	Area [mAU*s]	Height [mAU]	Area %
1	6.701	BB	0.0834	454.50510	83.67661	100.0000

Totals : 454.50510 83.67661

Signal 2: DAD1 B, Sig=230,4 Ref=560,80

Peak #	RetTime [min]	Type	Width [min]	Area [mAU*s]	Height [mAU]	Area %
1	1.272	BB	0.0482	6.47914	2.08602	0.3664
2	6.347	BB	0.0730	28.17687	5.97340	1.5935
3	6.701	BB	0.0835	1733.62195	318.65936	98.0401

Totals : 1768.27796 326.71878

===

*** End of Report ***

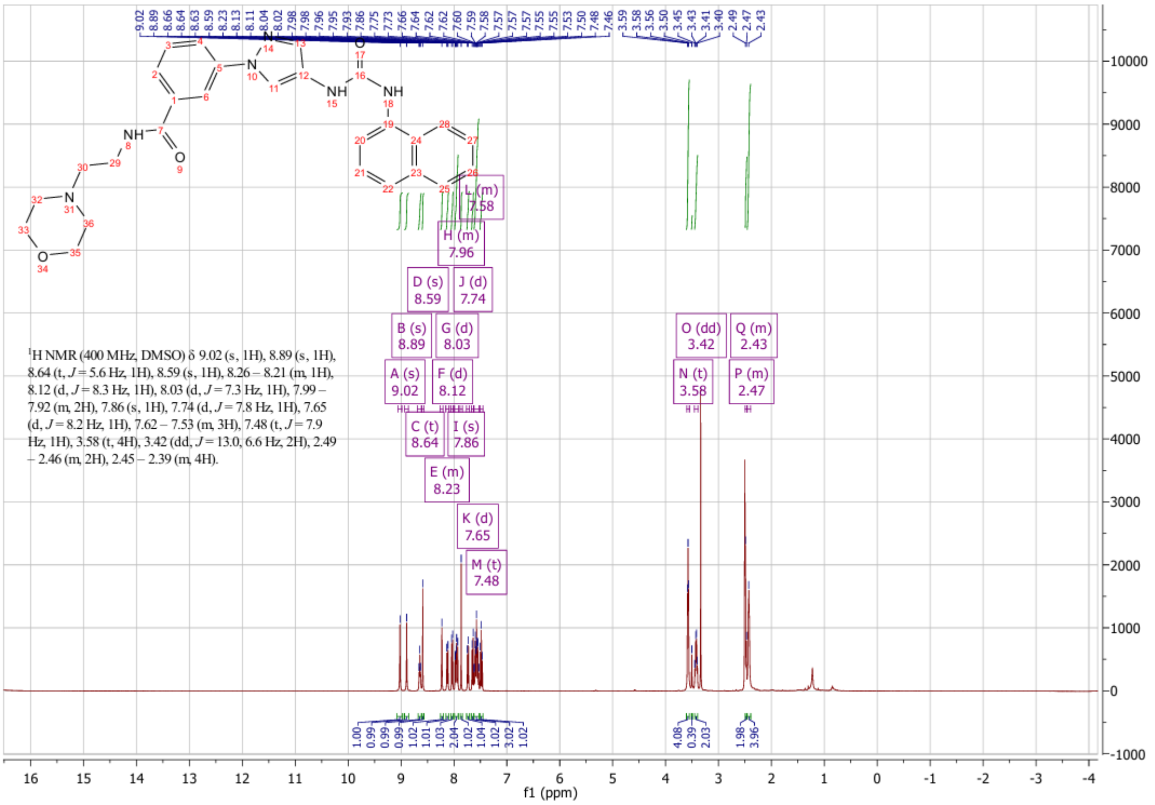
¹H NMR (400 MHz, DMSO) δ 9.02 (s, 1H), 8.89 (s, 1H), 8.64 (t, J = 5.6 Hz, 1H), 8.59 (s, 1H), 8.26 – 8.21 (m, 1H), 8.12 (d, J = 8.3 Hz, 1H), 8.03 (d, J = 7.3 Hz, 1H), 7.99 – 7.92 (m, 2H), 7.86 (s, 1H), 7.74 (d, J = 7.8 Hz, 1H), 7.65 (d, J = 8.2 Hz, 1H), 7.62 – 7.53 (m, 3H), 7.48 (t, J = 7.9 Hz, 1H), 3.58 (t, 4H), 3.42 (dd, J = 13.0, 6.6 Hz, 2H), 2.49 – 2.46 (m, 2H), 2.45 – 2.39 (m, 4H).
f1 (ppm)

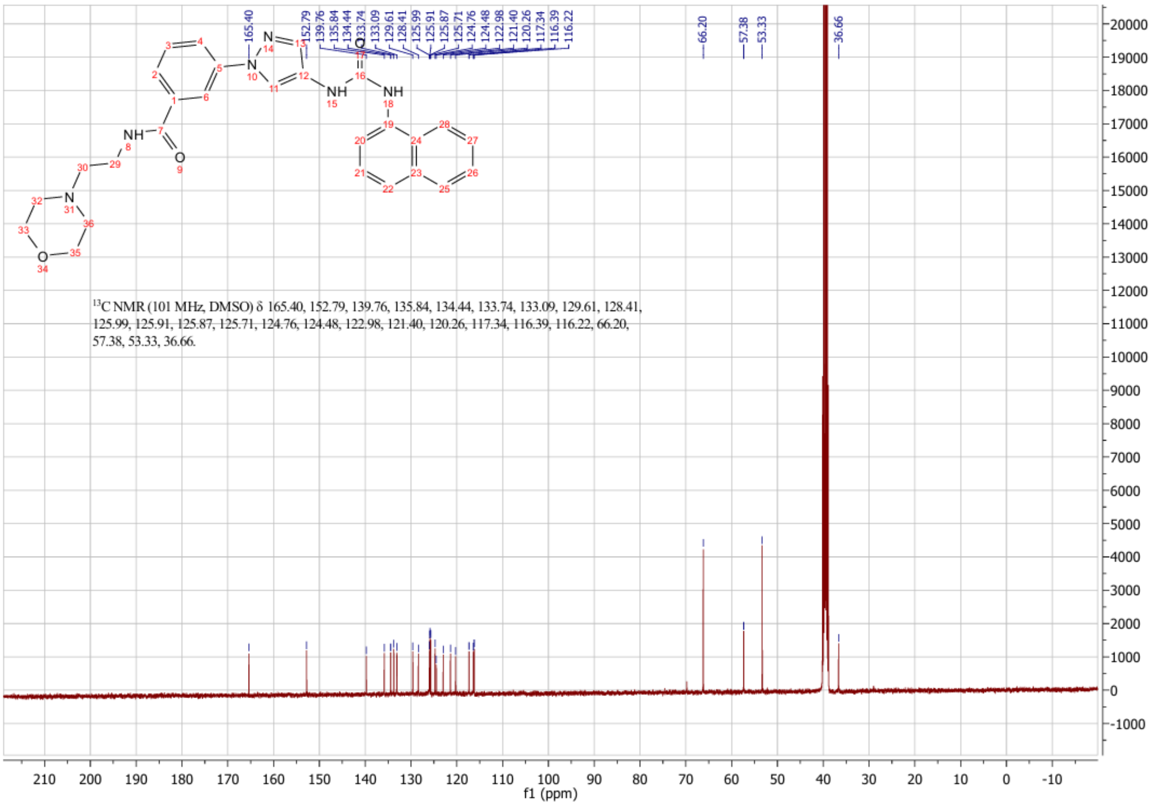
¹³C NMR (101 MHz, DMSO) δ 165.40, 152.79, 139.76, 135.84, 134.44, 133.74, 133.09, 129.61, 128.41, 125.99, 125.91, 125.87, 125.71, 124.76, 124.48, 122.98, 121.40, 120.26, 117.34, 116.39, 116.22, 66.20, 57.38, 53.33, 36.66.
f1 (ppm)

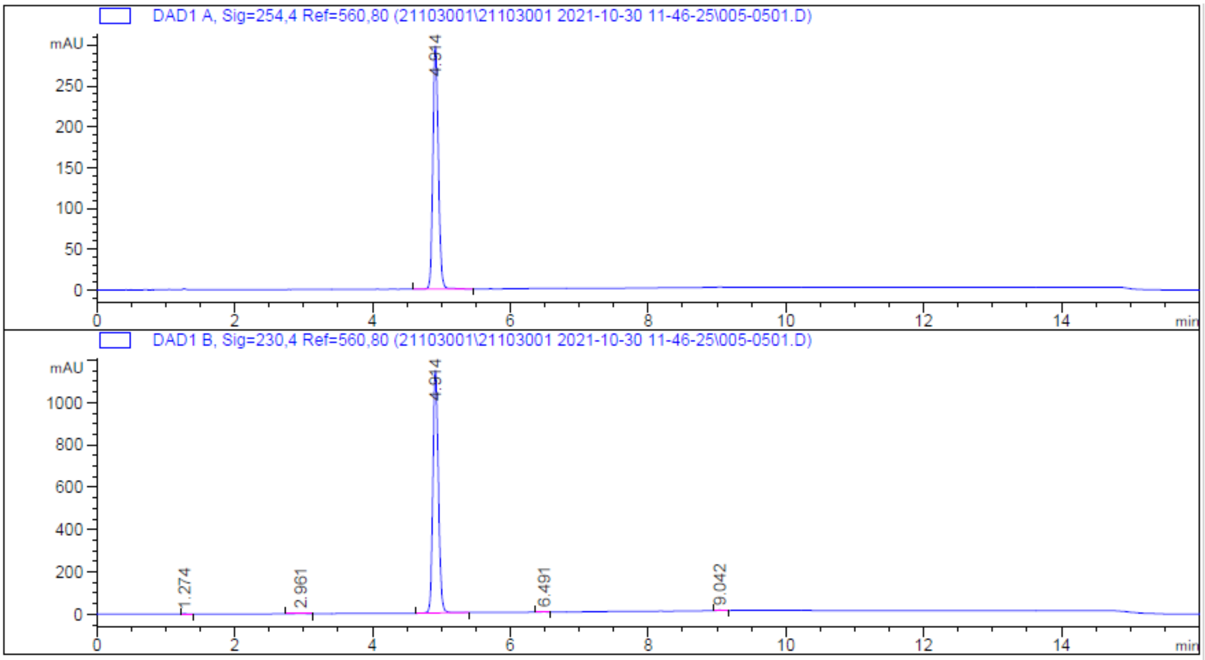

===

Area Percent Report

===

Sorted By : Signal
Multiplier: : 1.0000
Dilution: : 1.0000
Use Multiplier & Dilution Factor with ISTDs

Signal 1: DAD1 A, Sig=254,4 Ref=560,80

Peak #	RetTime [min]	Type	Width [min]	Area [mAU*s]	Height [mAU]	Area %
1	4.914	BB	0.0864	1645.87708	298.00943	100.0000

Totals : 1645.87708 298.00943

Signal 2: DAD1 B, Sig=230,4 Ref=560,80

Peak #	RetTime [min]	Type	Width [min]	Area [mAU*s]	Height [mAU]	Area %
1	1.274	BB	0.0482	6.13461	1.97627	0.0957
2	2.961	BB	0.1056	21.13978	2.94944	0.3299

Peak #	RetTime [min]	Type	Width [min]	Area [mAU*s]	Height [mAU]	Area %
3	4.914	BB	0.0867	6360.03760	1146.00488	99.2409
4	6.491	BB	0.0796	7.52502	1.47297	0.1174
5	9.042	BB	0.0731	13.84840	2.93138	0.2161

Totals : 6408.68541 1155.33494

===

*** End of Report ***

SI-IV

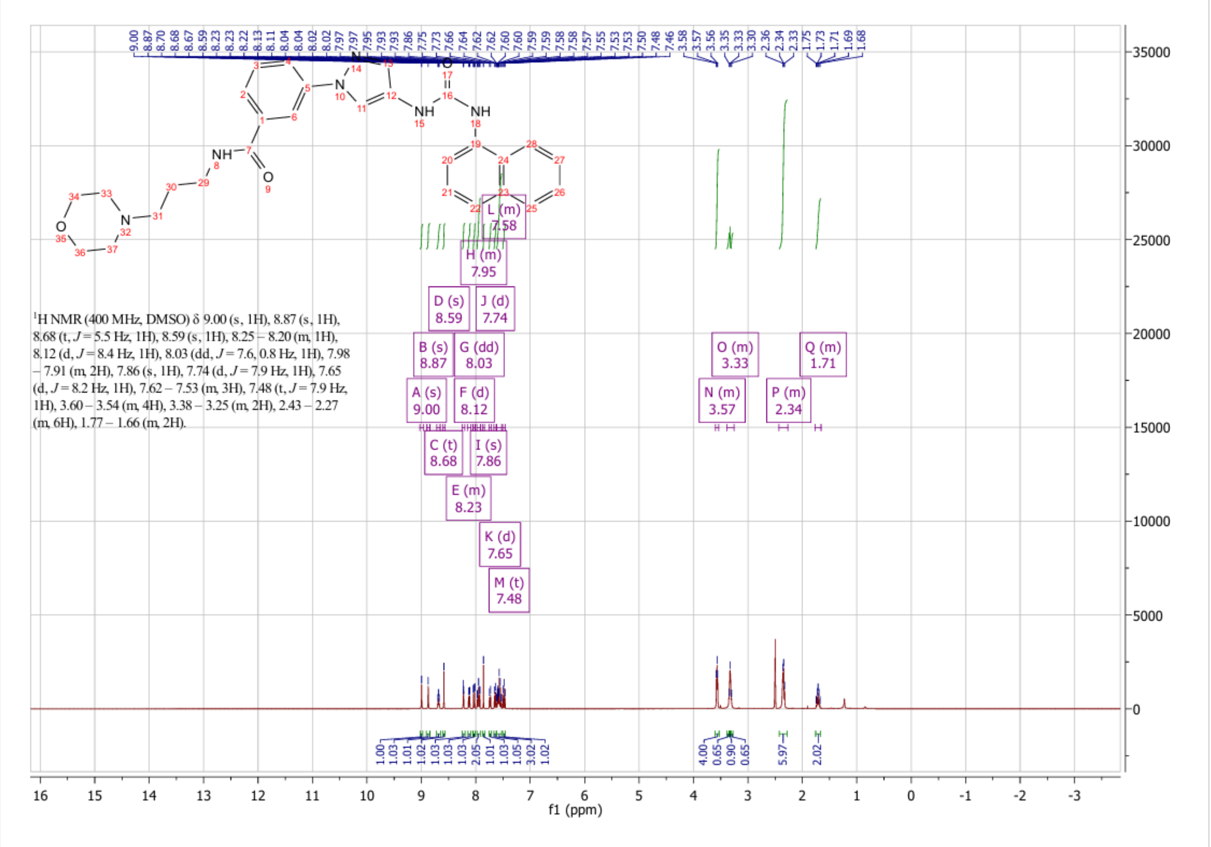

^{1}H NMR (400 MHz, DMSO) δ 9.00 (s, 1H), 8.87 (s, 1H), 8.68 (t, J = 5.5 Hz, 1H), 8.59 (s, 1H), 8.25 – 8.20 (m, 1H), 8.12 (d, J = 8.4 Hz, 1H), 8.03 (dd, J = 7.6, 0.8 Hz, 1H), 7.98 – 7.91 (m, 2H), 7.86 (s, 1H), 7.74 (d, J = 7.9 Hz, 1H), 7.65 (d, J = 8.2 Hz, 1H), 7.62 – 7.53 (m, 3H), 7.48 (t, J = 7.9 Hz, 1H), 3.60 – 3.54 (m, 4H), 3.38 – 3.25 (m, 2H), 2.43 – 2.27 (m, 6H), 1.77 – 1.66 (m, 2H).
f1 (ppm)

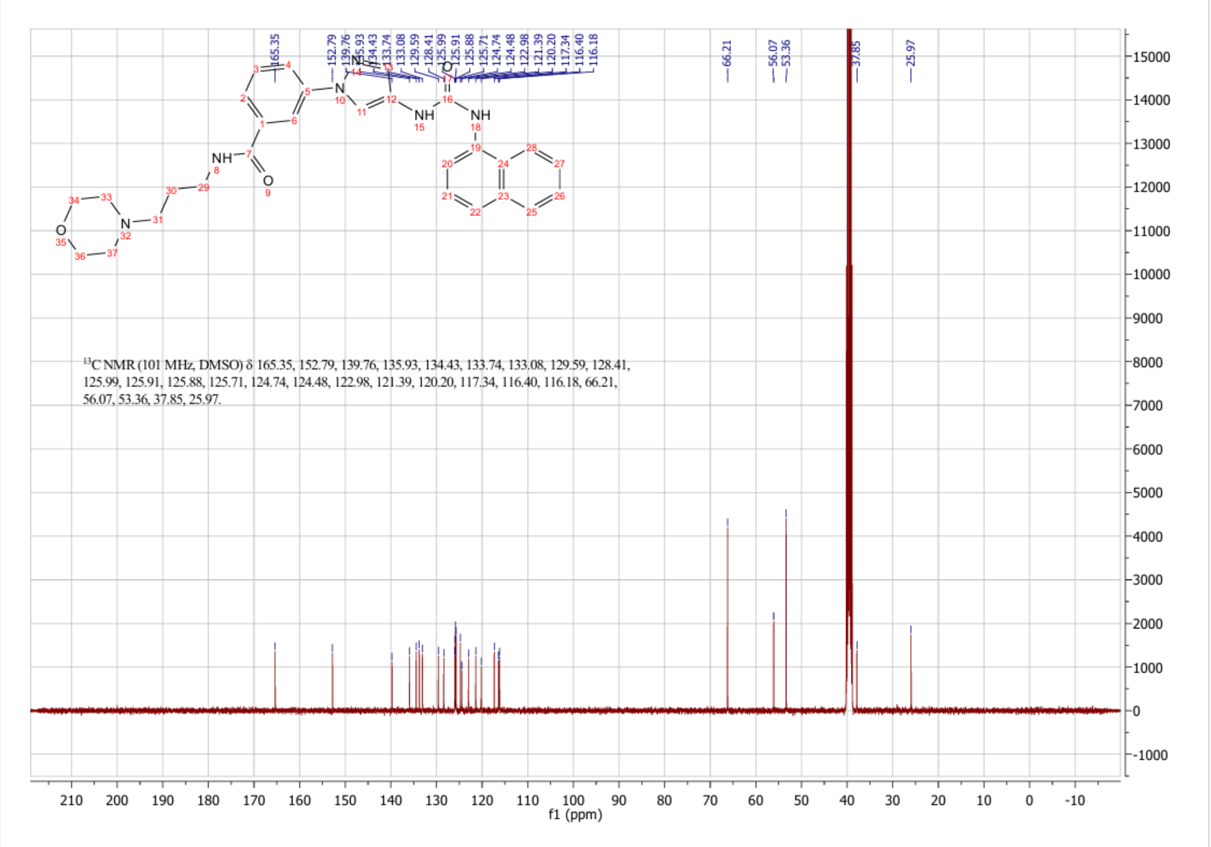

^{13}C NMR (101 MHz, DMSO) δ 165.35, 152.79, 139.76, 135.93, 134.43, 133.74, 133.08, 129.59, 128.41, 125.99, 125.91, 125.88, 125.71, 124.74, 124.48, 122.98, 121.39, 120.20, 117.34, 116.40, 116.18, 66.21, 56.07, 53.36, 37.85, 25.97.
f1 (ppm)

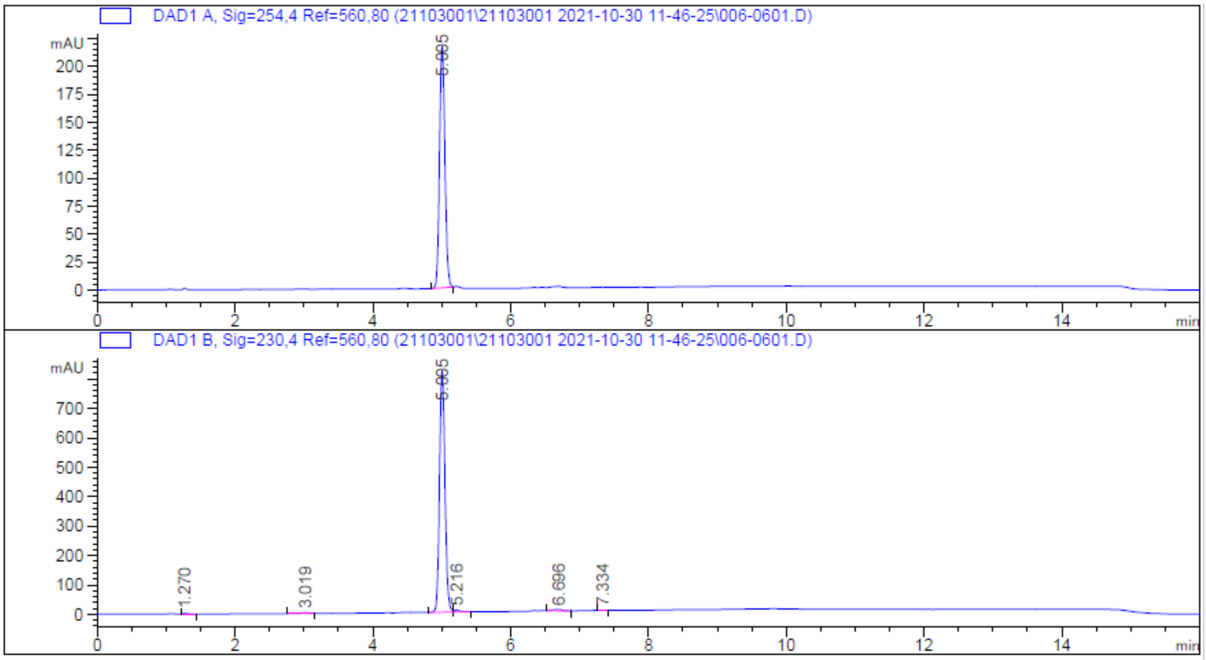


```
=====================================================================
                        Area Percent Report
=====================================================================

Sorted By             :      Signal
Multiplier:                  :        1.0000
Dilution:                    :        1.0000
Use Multiplier & Dilution Factor with ISTDs


Signal 1: DAD1 A, Sig=254,4 Ref=560,80
```

Peak #	RetTime [min]	Type	Width [min]	Area [mAU*s]	Height [mAU]	Area %
1	5.005	BB	0.0847	1164.06897	216.42831	100.0000

Totals : 1164.06897 216.42831

Signal 2: DAD1 B, Sig=230,4 Ref=560,80

Peak #	RetTime [min]	Type	Width [min]	Area [mAU*s]	Height [mAU]	Area %
1	1.270	BB	0.0484	6.67419	2.13950	0.1451
2	3.019	BB	0.1176	20.27158	2.47363	0.4406
3	5.005	BV	0.0856	4487.71826	822.80286	97.5431
4	5.216	VB	0.0866	40.34510	6.85654	0.8769
5	6.696	BB	0.1146	40.87000	4.93597	0.8883
6	7.334	BB	0.0655	4.87372	1.14935	0.1059

Totals : 4600.75285 840.35785

```
=====================================================================
                        *** End of Report ***
```

SI-V

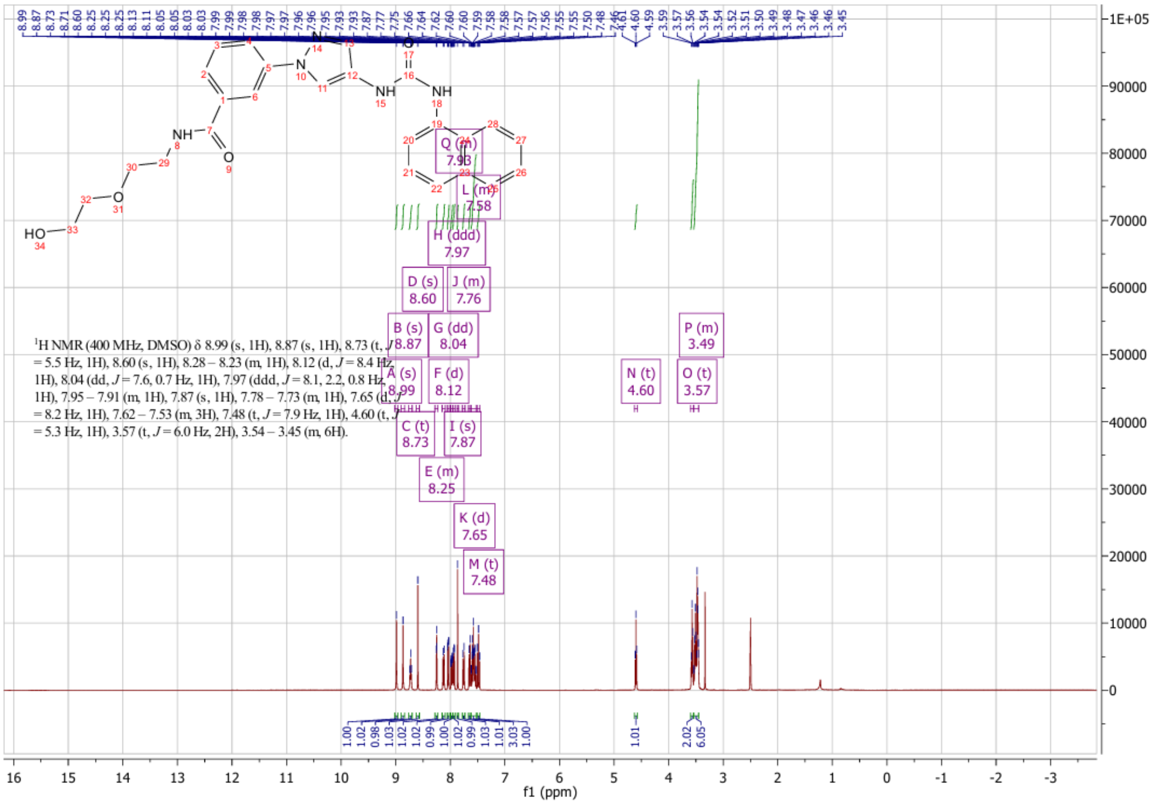

^{1}H NMR (400 MHz, DMSO) δ 8.99 (s, 1H), 8.87 (s, 1H), 8.73 (t, J = 5.5 Hz, 1H), 8.60 (s, 1H), 8.28 – 8.23 (m, 1H), 8.12 (d, J = 8.4 Hz, 1H), 8.04 (dd, J = 7.6, 0.7 Hz, 1H), 7.97 (ddd, J = 8.1, 2.2, 0.8 Hz, 1H), 7.95 – 7.91 (m, 1H), 7.87 (s, 1H), 7.78 – 7.73 (m, 1H), 7.65 (d, J = 8.2 Hz, 1H), 7.62 – 7.53 (m, 3H), 7.48 (t, J = 7.9 Hz, 1H), 4.60 (t, J = 5.3 Hz, 1H), 3.57 (t, J = 6.0 Hz, 2H), 3.54 – 3.45 (m, 6H).
f1 (ppm)

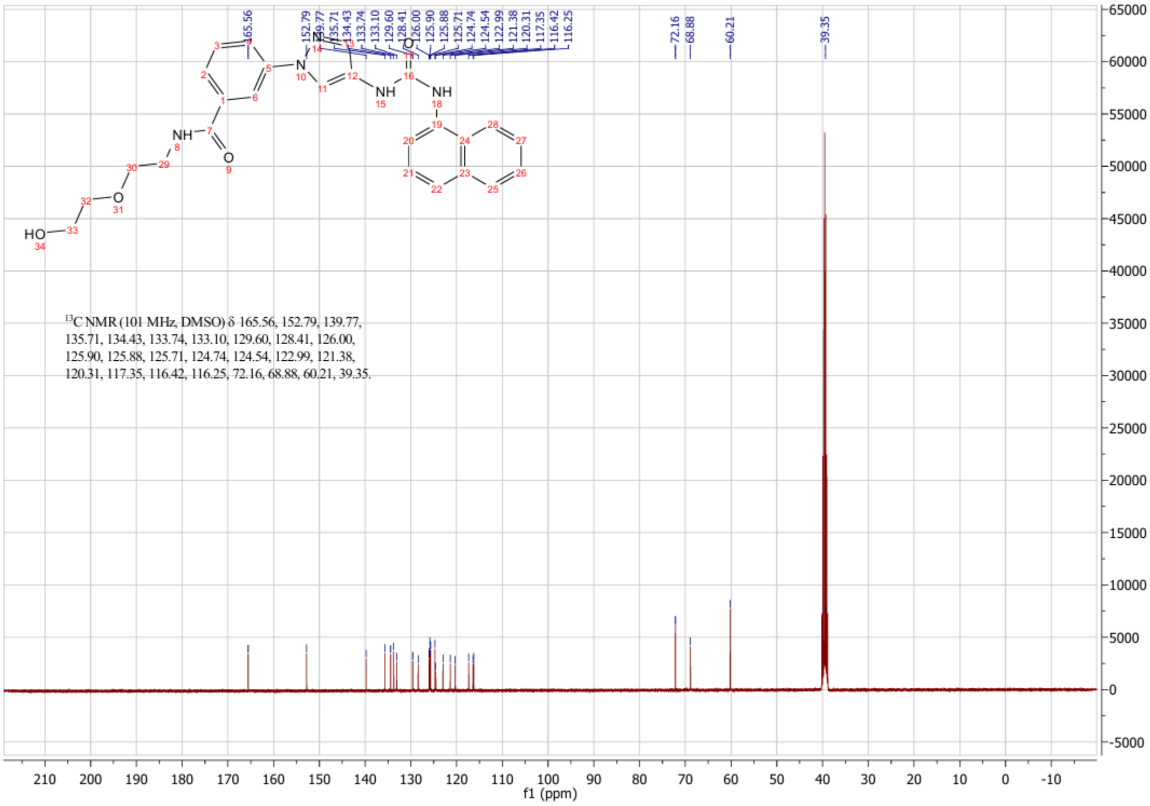

^{13}C NMR (101 MHz, DMSO) δ 165.56, 152.79, 139.77, 135.71, 134.43, 133.74, 133.10, 129.60, 128.41, 126.00, 125.90, 125.88, 125.71, 124.74, 124.54, 122.99, 121.38, 120.31, 117.35, 116.42, 116.25, 72.16, 68.88, 60.21, 39.35.
f1 (ppm)

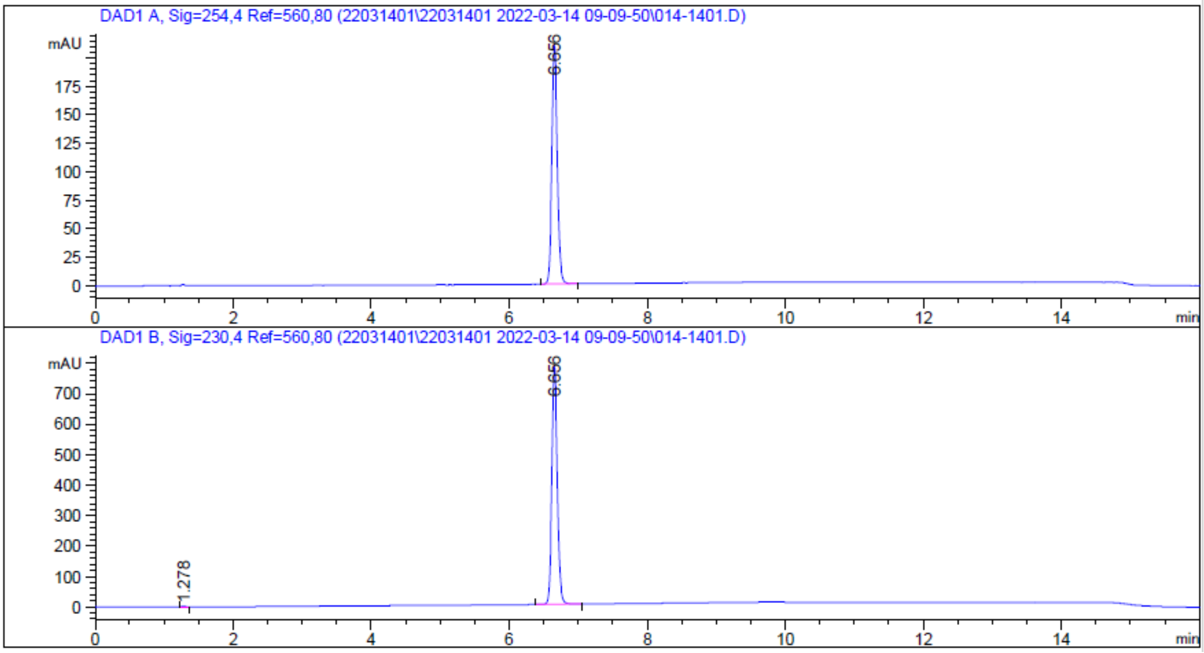

===

Area Percent Report

===

Sorted By : Signal
Multiplier: : 1.0000
Dilution: : 1.0000
Use Multiplier & Dilution Factor with ISTDs

Signal 1: DAD1 A, Sig=254,4 Ref=560,80

Peak #	RetTime [min]	Type	Width [min]	Area [mAU*s]	Height [mAU]	Area %
1	6.656	BB	0.0839	1148.94482	209.91750	100.0000

Totals : 1148.94482 209.91750

Signal 2: DAD1 B, Sig=230,4 Ref=560,80

Peak #	RetTime [min]	Type	Width [min]	Area [mAU*s]	Height [mAU]	Area %
1	1.278	BB	0.0471	5.79157	1.92649	0.1351
2	6.656	BB	0.0840	4281.86670	779.95074	99.8649

Totals : 4287.65827 781.87724

===

*** End of Report ***

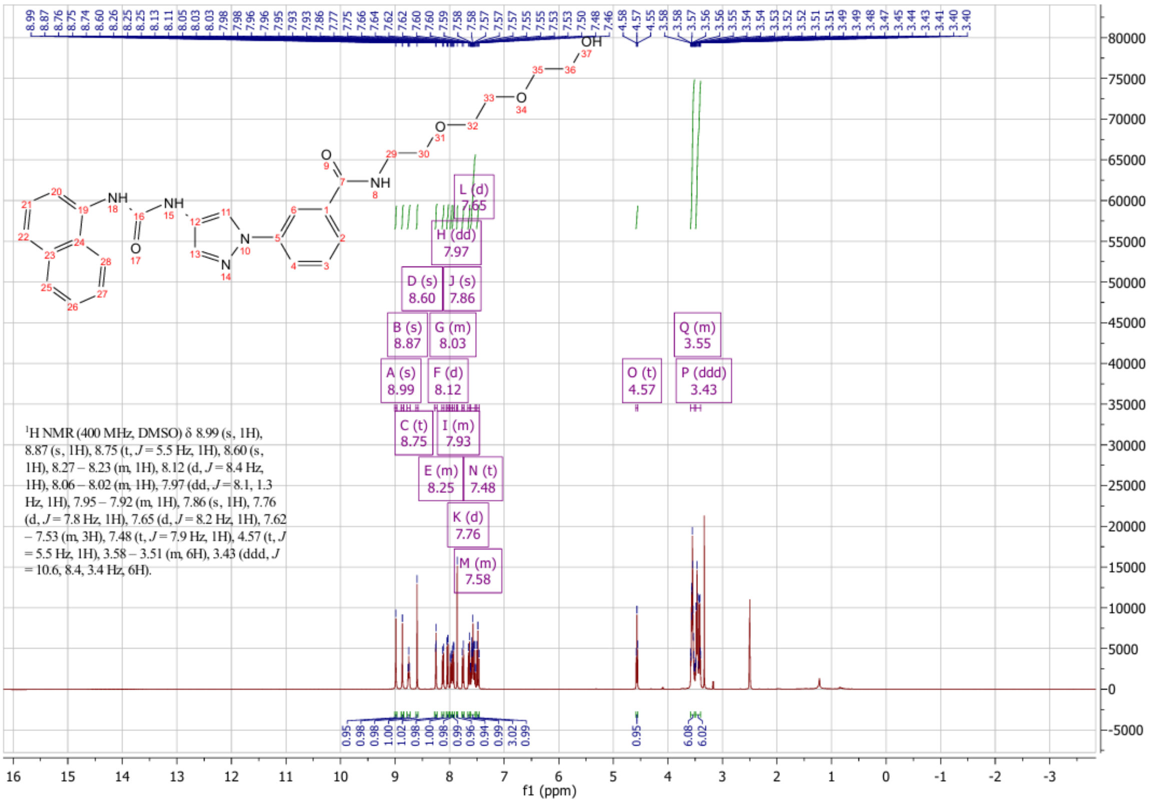

^{1}H NMR (400 MHz, DMSO) δ 8.99 (s, 1H), 8.87 (s, 1H), 8.75 (t, J = 5.5 Hz, 1H), 8.60 (s, 1H), 8.27 – 8.23 (m, 1H), 8.12 (d, J = 8.4 Hz, 1H), 8.06 – 8.02 (m, 1H), 7.97 (dd, J = 8.1, 1.3 Hz, 1H), 7.95 – 7.92 (m, 1H), 7.86 (s, 1H), 7.76 (d, J = 7.8 Hz, 1H), 7.65 (d, J = 8.2 Hz, 1H), 7.62 – 7.53 (m, 3H), 7.48 (t, J = 7.9 Hz, 1H), 4.57 (t, J = 5.5 Hz, 1H), 3.58 – 3.51 (m, 6H), 3.43 (ddd, J = 10.6, 8.4, 3.4 Hz, 6H).
f1 (ppm)

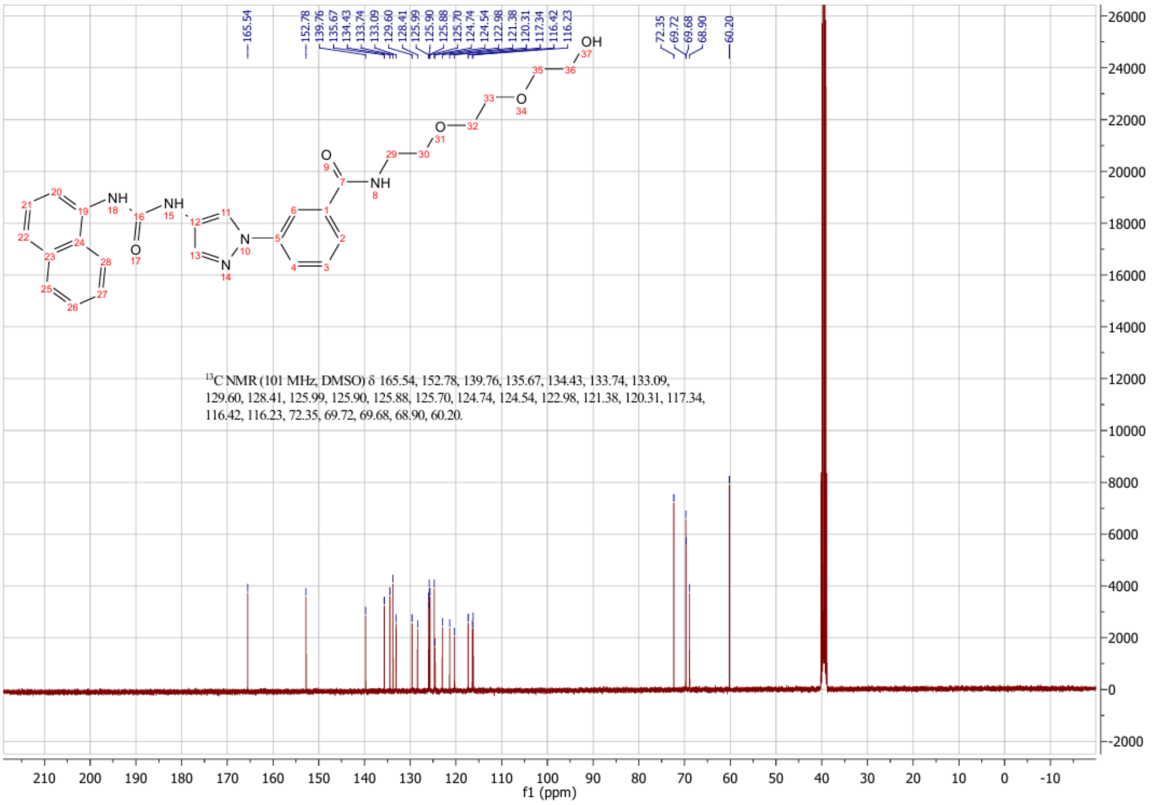

^{13}C NMR (101 MHz, DMSO) δ 165.54, 152.78, 139.76, 135.67, 134.43, 133.74, 133.09, 129.60, 128.41, 125.99, 125.90, 125.88, 125.70, 124.74, 124.54, 122.98, 121.38, 120.31, 117.34, 116.42, 116.23, 72.35, 69.72, 69.68, 68.90, 60.20.
f1 (ppm)

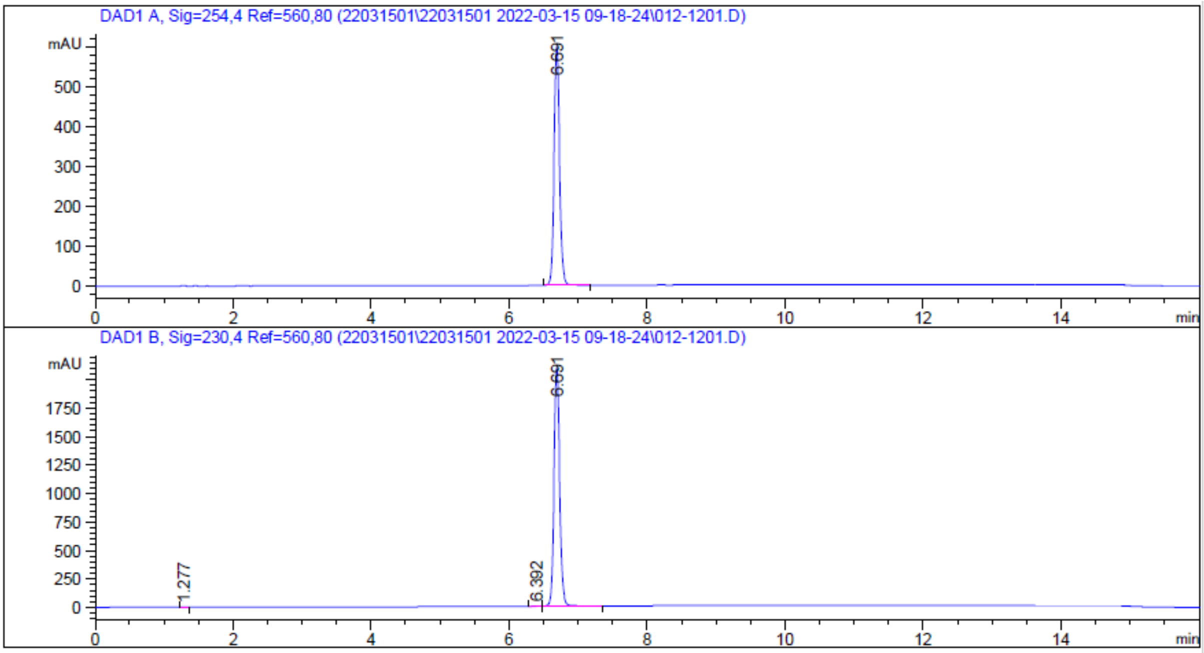

===

Area Percent Report

===

Sorted By : Signal
Multiplier: : 1.0000
Dilution: : 1.0000
Use Multiplier & Dilution Factor with ISTDs

Signal 1: DAD1 A, Sig=254,4 Ref=560,80

Peak #	RetTime [min]	Type	Width [min]	Area [mAU*s]	Height [mAU]	Area %
1	6.691	BB	0.0838	3286.26392	601.20044	100.0000

Totals : 3286.26392 601.20044

Signal 2: DAD1 B, Sig=230,4 Ref=560,80

Peak #	RetTime [min]	Type	Width [min]	Area [mAU*s]	Height [mAU]	Area %
1	1.277	BB	0.0489	5.94160	1.87729	0.0501
2	6.392	BV	0.0909	17.30909	2.93194	0.1460
3	6.691	VB	0.0857	1.18318e4	2101.16699	99.8039

Totals : 1.18551e4 2105.97622

===

*** End of Report ***

11.5 Table 4

28a

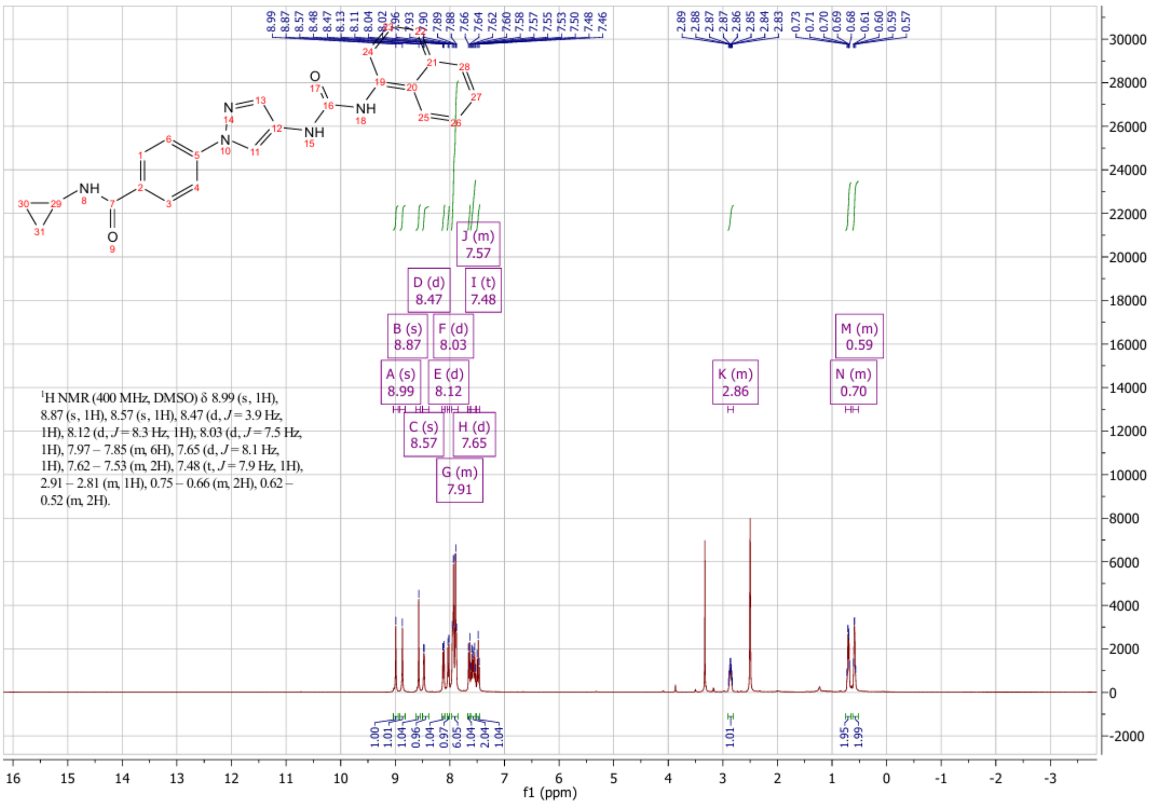

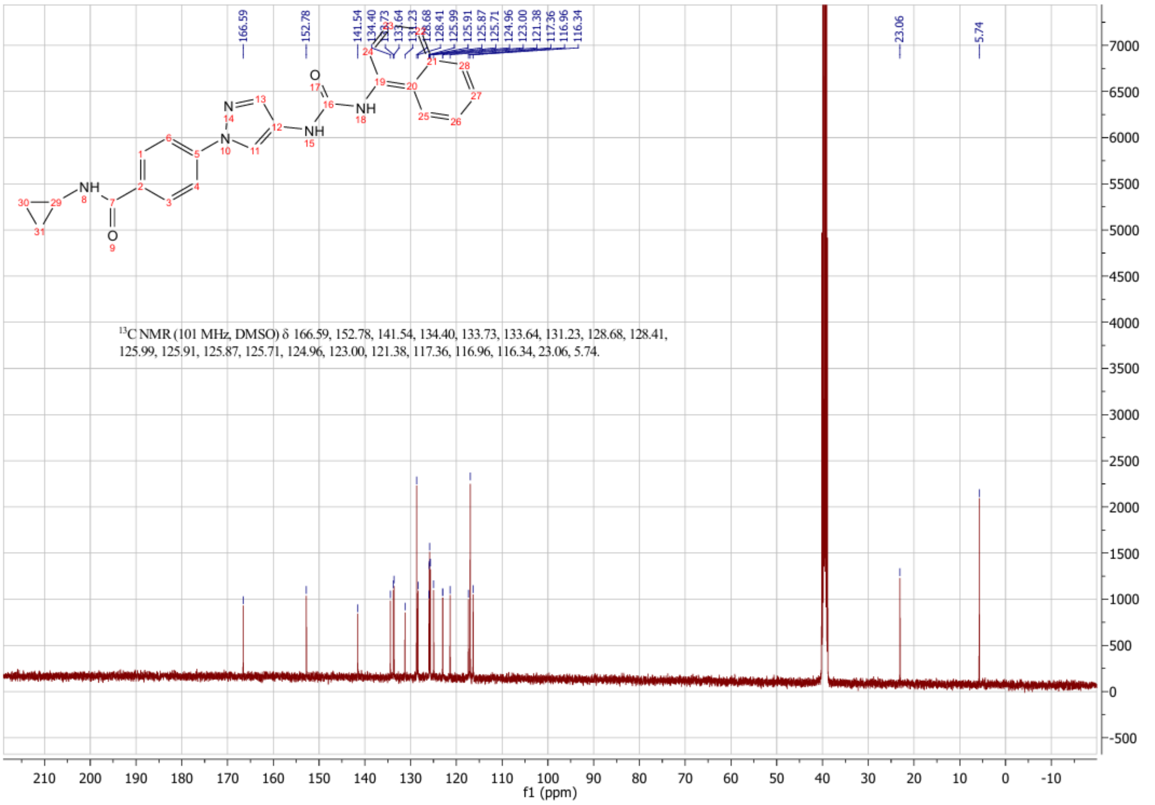

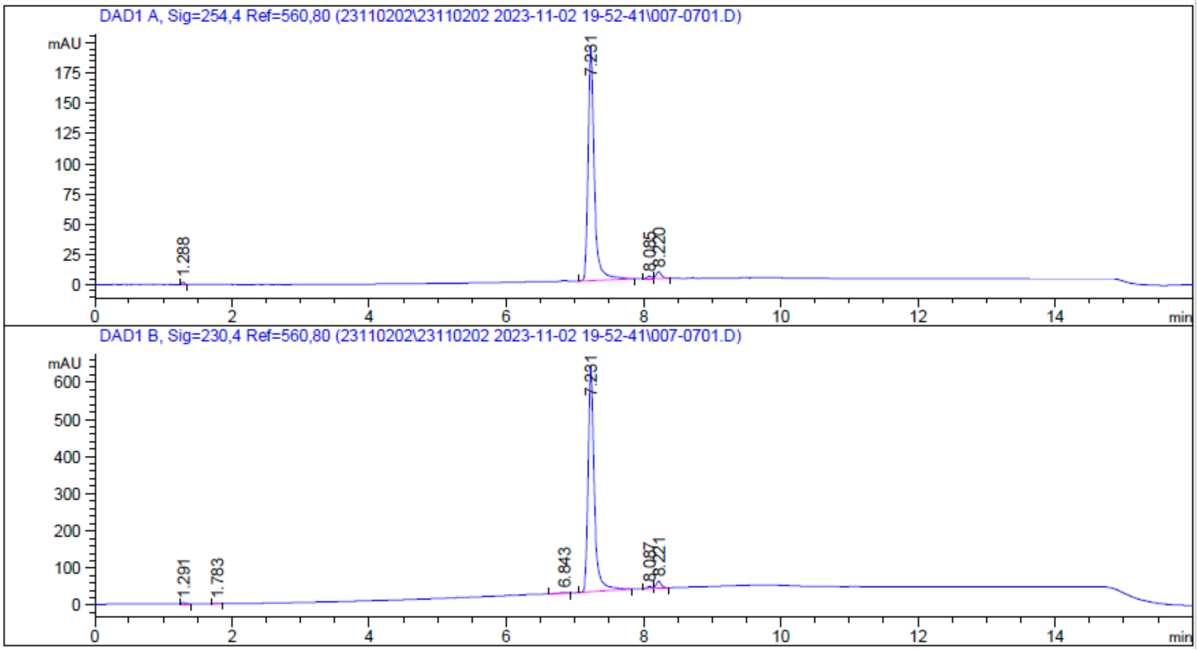

===

Area Percent Report

===

Sorted By : Signal
Multiplier: : 1.0000
Dilution: : 1.0000
Use Multiplier & Dilution Factor with ISTDs

Signal 1: DAD1 A, Sig=254,4 Ref=560,80

Peak #	RetTime [min]	Type	Width [min]	Area [mAU*s]	Height [mAU]	Area %
1	1.288	BB	0.0405	5.31613	2.17494	0.4211
2	7.231	BB	0.0933	1217.30249	193.81703	96.4163
3	8.085	BV	0.0759	9.02533	1.88267	0.7148
4	8.220	VB	0.0803	30.90449	5.78994	2.4478

Totals : 1262.54844 203.66459

Signal 2: DAD1 B, Sig=230,4 Ref=560,80

Peak #	RetTime [min]	Type	Width [min]	Area [mAU*s]	Height [mAU]	Area %
1	1.291	BB	0.0455	11.47279	3.99363	0.2894
2	1.783	BB	0.0562	4.22468	1.16892	0.1066
3	6.843	BB	0.0819	14.10052	2.65878	0.3557
4	7.231	BB	0.0932	3810.57935	607.38202	96.1321
5	8.087	BV	0.0757	24.31619	5.09219	0.6134
6	8.221	VB	0.0794	99.20502	18.86149	2.5027

Totals : 3963.89853 639.15703

===

*** End of Report ***

28b

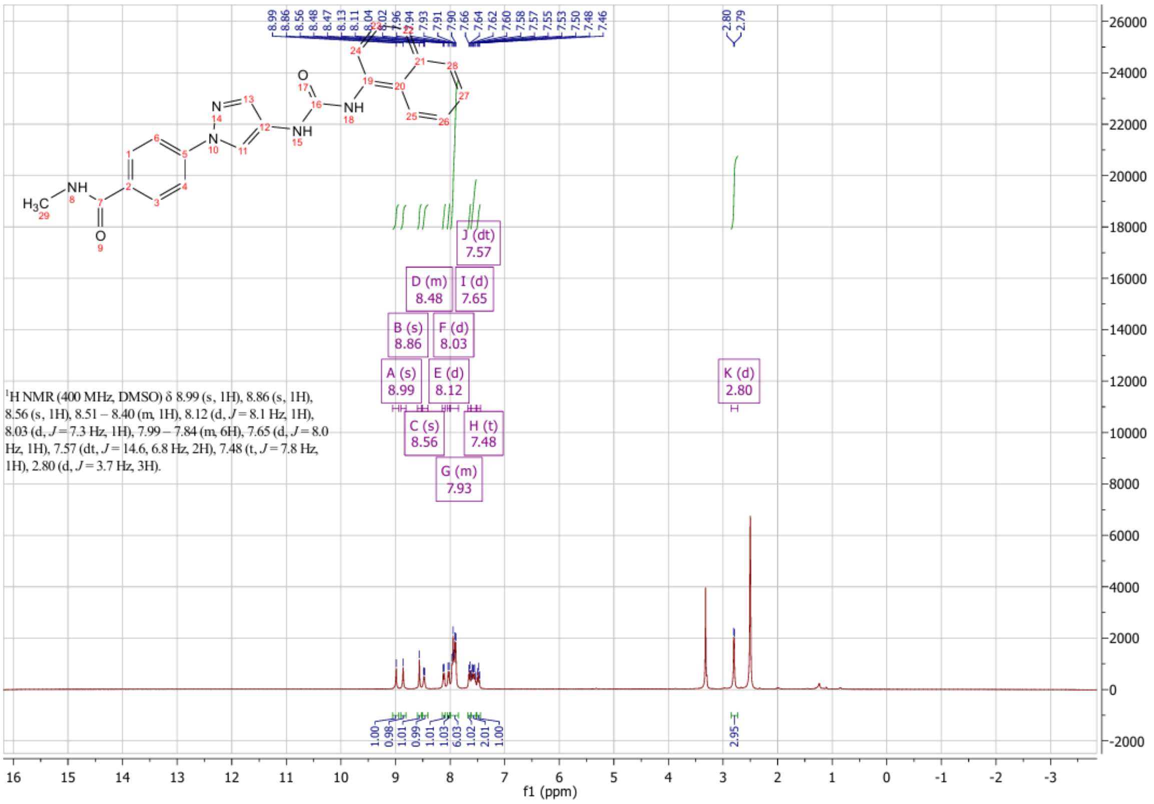

^{1}H NMR (400 MHz, DMSO) δ 8.99 (s, 1H), 8.86 (s, 1H), 8.56 (s, 1H), 8.51 – 8.40 (m, 1H), 8.12 (d, J = 8.1 Hz, 1H), 8.03 (d, J = 7.3 Hz, 1H), 7.99 – 7.84 (m, 6H), 7.65 (d, J = 8.0 Hz, 1H), 7.57 (dt, J = 14.6, 6.8 Hz, 2H), 7.48 (t, J = 7.8 Hz, 1H), 2.80 (d, J = 3.7 Hz, 3H).
f1 (ppm)

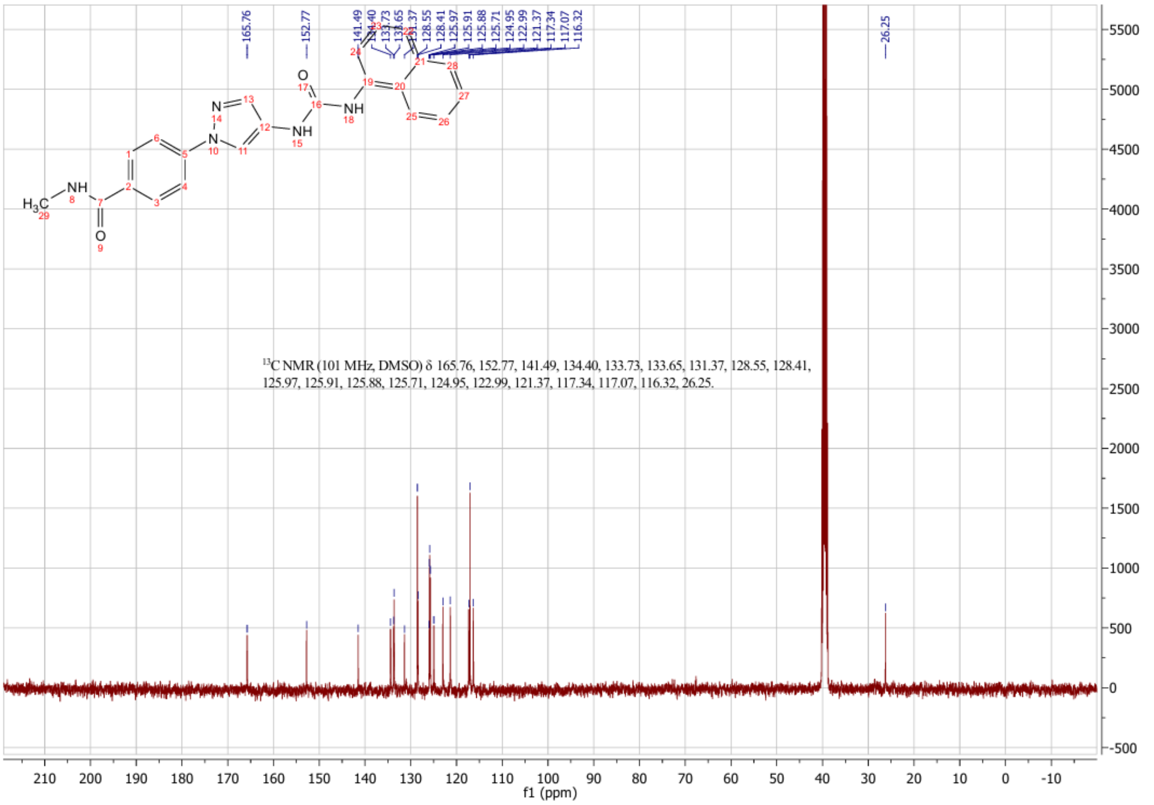

^{13}C NMR (101 MHz, DMSO) δ 165.76, 152.77, 141.49, 134.40, 133.73, 133.65, 131.37, 128.55, 128.41, 125.97, 125.91, 125.88, 125.71, 124.95, 122.99, 121.37, 117.34, 117.07, 116.32, 26.25.
f1 (ppm)

11.6 Table 5

35a

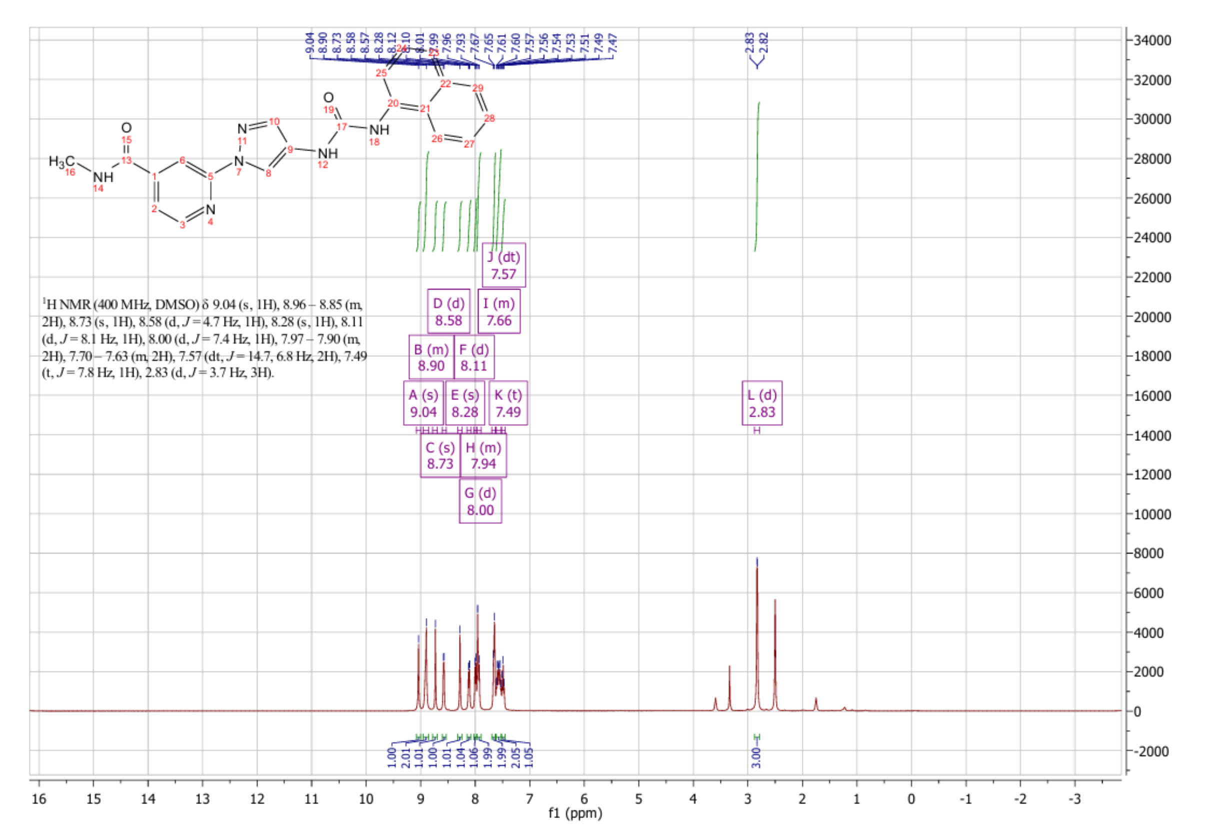

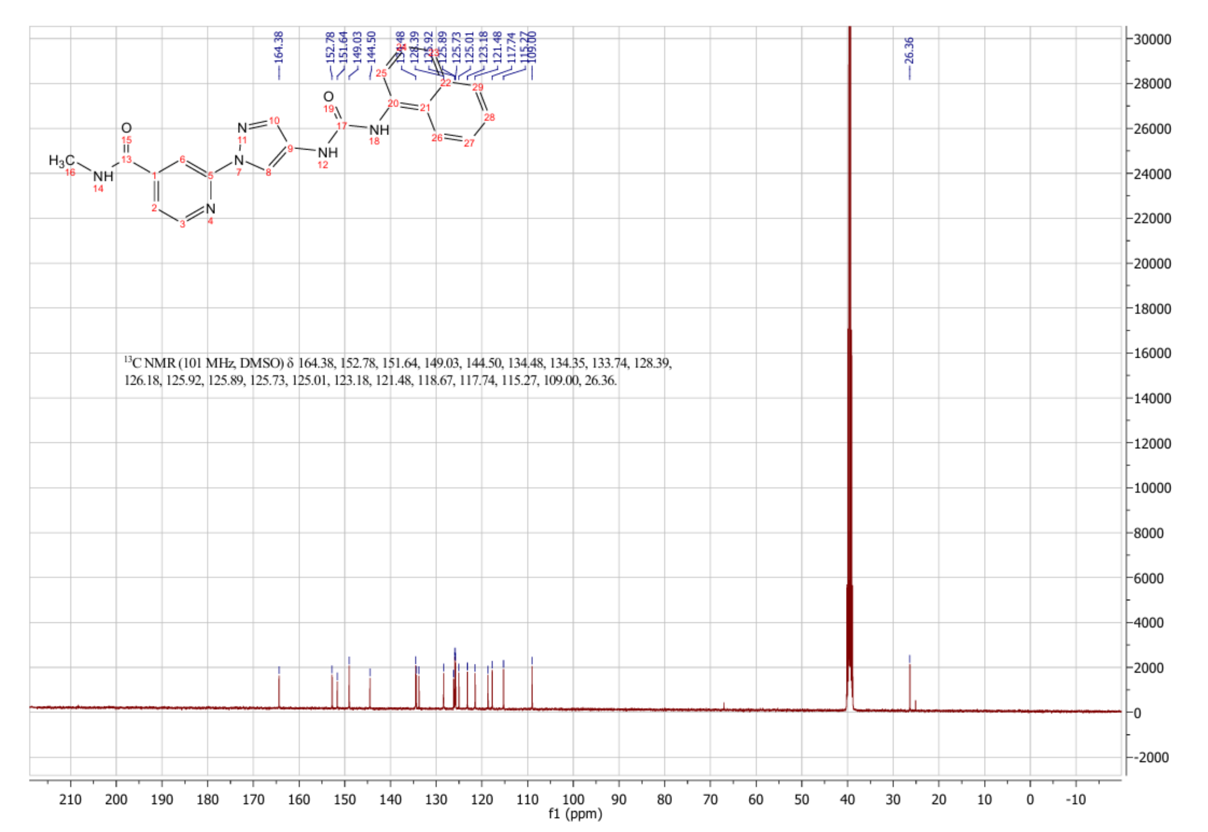

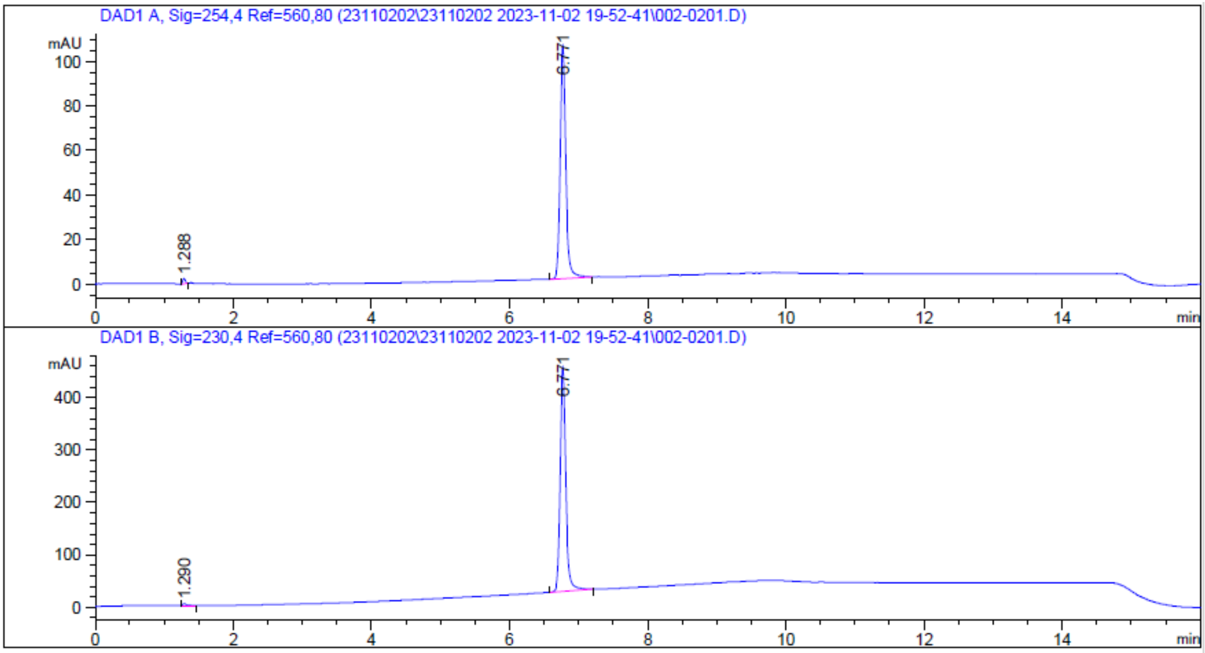

===

Area Percent Report

===

Sorted By : Signal
Multiplier: : 1.0000
Dilution: : 1.0000
Use Multiplier & Dilution Factor with ISTDs

Signal 1: DAD1 A, Sig=254,4 Ref=560,80

Peak #	RetTime [min]	Type	Width [min]	Area [mAU*s]	Height [mAU]	Area %
1	1.288	BB	0.0411	5.84789	2.34508	0.9794
2	6.771	BB	0.0857	591.26178	105.01181	99.0206

Totals : 597.10966 107.35690

Signal 2: DAD1 B, Sig=230,4 Ref=560,80

Peak #	RetTime [min]	Type	Width [min]	Area [mAU*s]	Height [mAU]	Area %
1	1.290	BB	0.0512	14.47720	4.30747	0.5951
2	6.771	BB	0.0858	2418.08472	428.80905	99.4049

Totals : 2432.56192 433.11652

===

*** End of Report ***

36a

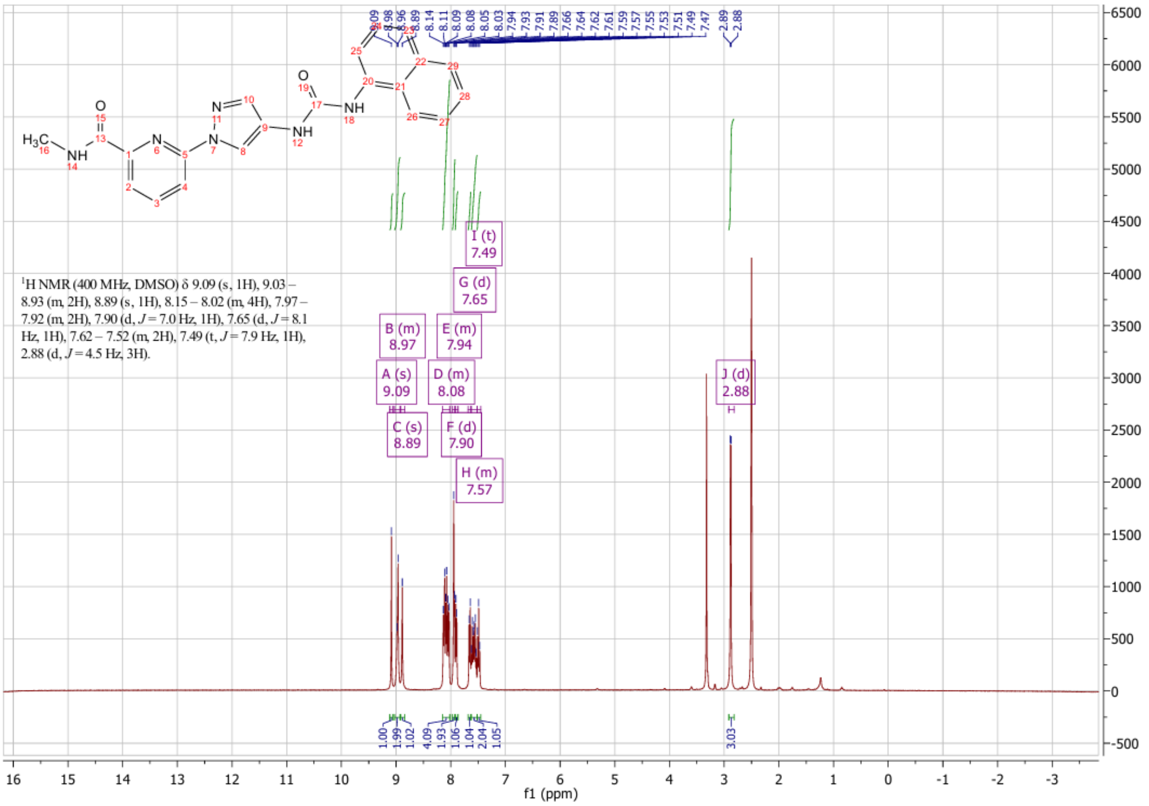

^{1}H NMR (400 MHz, DMSO) δ 9.09 (s, 1H), 9.03 – 8.93 (m, 2H), 8.89 (s, 1H), 8.15 – 8.02 (m, 4H), 7.97 – 7.92 (m, 2H), 7.90 (d, J = 7.0 Hz, 1H), 7.65 (d, J = 8.1 Hz, 1H), 7.62 – 7.52 (m, 2H), 7.49 (t, J = 7.9 Hz, 1H), 2.88 (d, J = 4.5 Hz, 3H).
f1 (ppm)

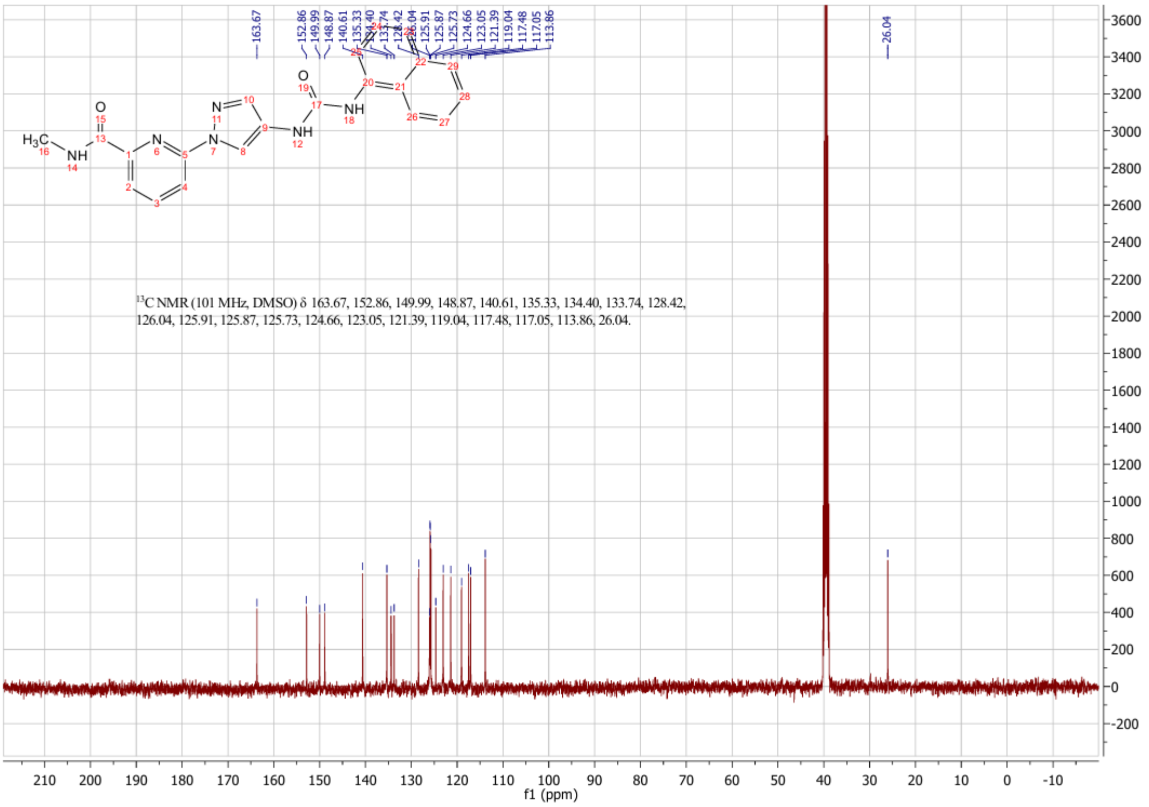

^{13}C NMR (101 MHz, DMSO) δ 163.67, 152.86, 149.99, 148.87, 140.61, 135.33, 134.40, 133.74, 128.42, 126.04, 125.91, 125.87, 125.73, 124.66, 123.05, 121.39, 119.04, 117.48, 117.05, 113.86, 26.04.
f1 (ppm)

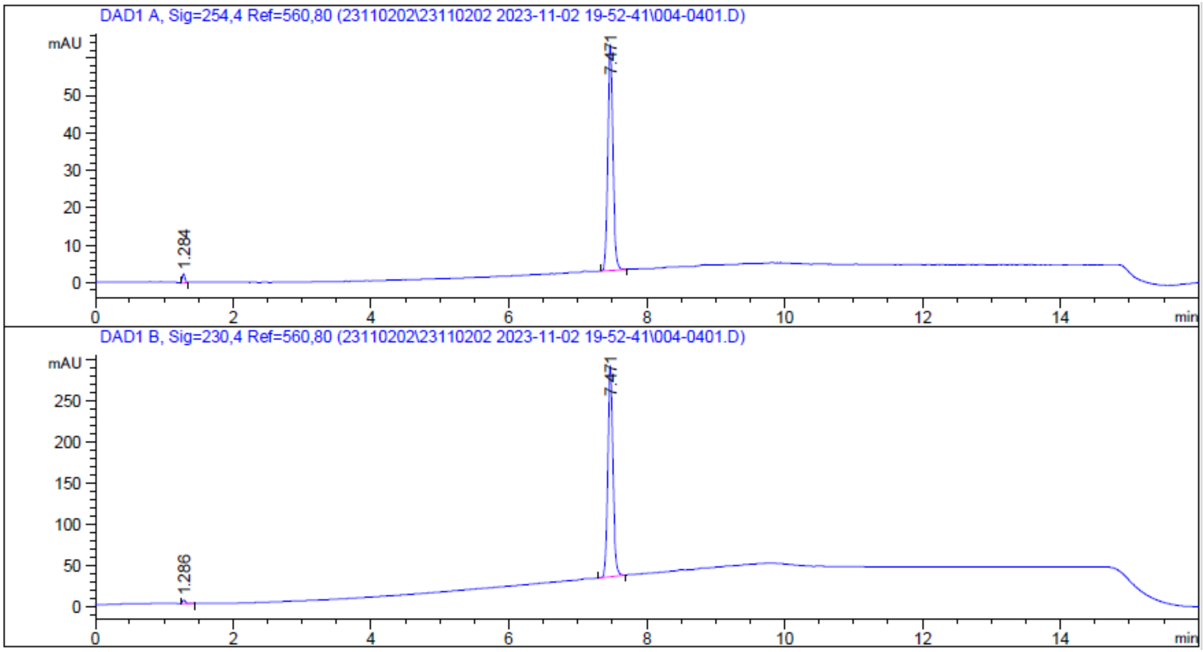

===

Area Percent Report

===

Sorted By : Signal
Multiplier: : 1.0000
Dilution: : 1.0000
Use Multiplier & Dilution Factor with ISTDs

Signal 1: DAD1 A, Sig=254,4 Ref=560,80

Peak #	RetTime [min]	Type	Width [min]	Area [mAU*s]	Height [mAU]	Area %
1	1.284	BB	0.0410	5.71635	2.30032	1.8047
2	7.471	BB	0.0803	311.03464	60.15941	98.1953

Totals : 316.75099 62.45973

Signal 2: DAD1 B, Sig=230,4 Ref=560,80

Peak #	RetTime [min]	Type	Width [min]	Area [mAU*s]	Height [mAU]	Area %
1	1.286	BB	0.0476	13.02698	4.26670	0.9769
2	7.471	BB	0.0802	1320.53479	256.10876	99.0231

Totals : 1333.56177 260.37547

===

*** End of Report ***

11.7 Table 6

45a

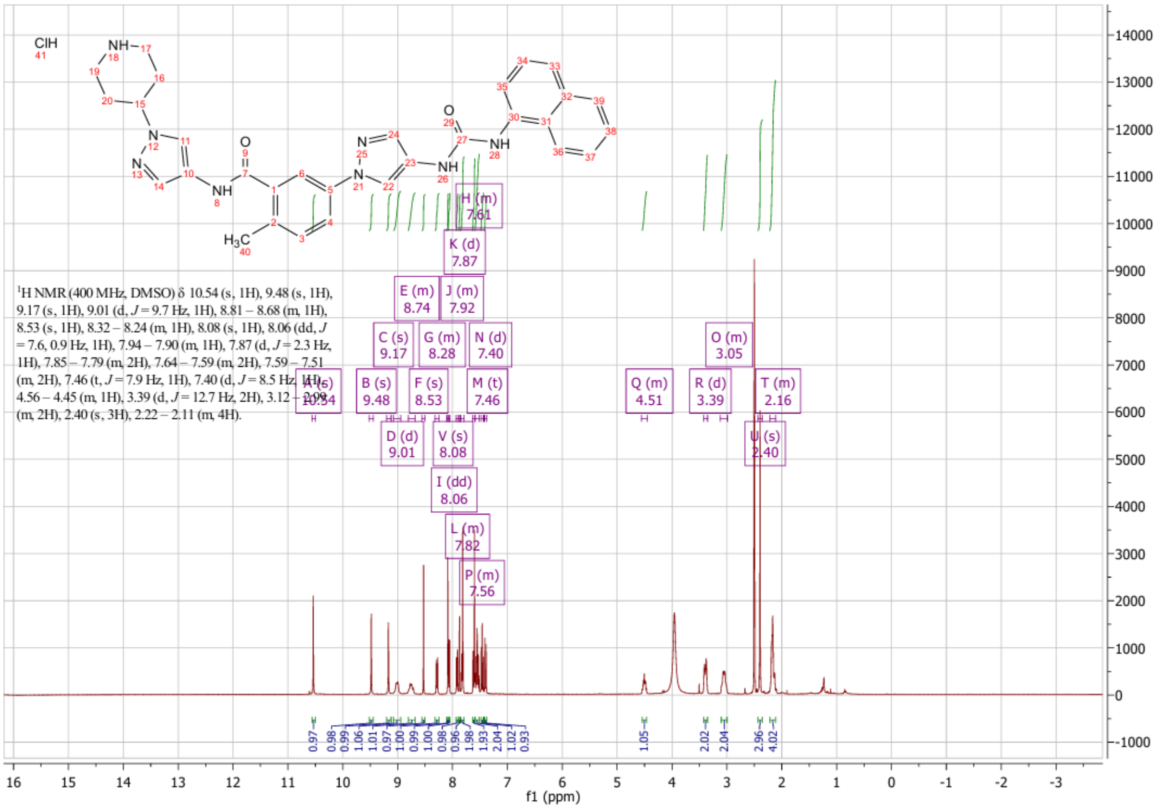

45b

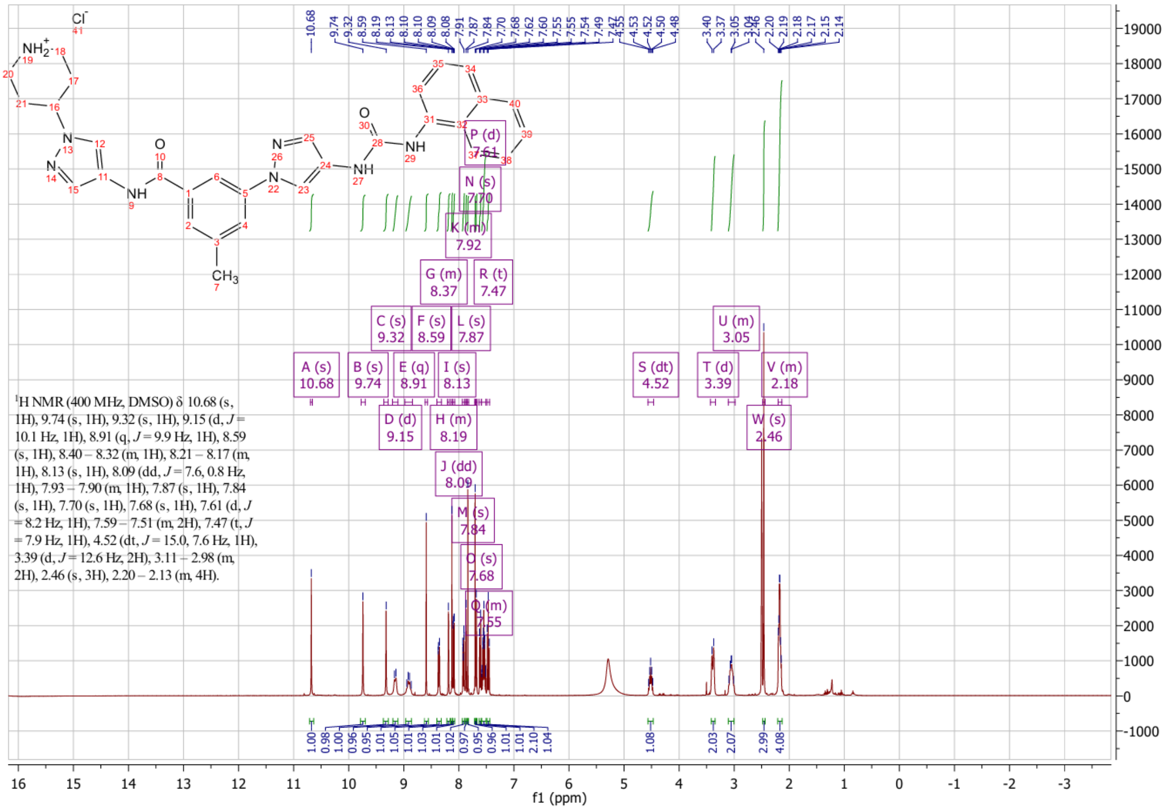
^{1}H NMR (400 MHz, DMSO) δ 10.68 (s, 1H), 9.74 (s, 1H), 9.32 (s, 1H), 9.15 (d, J = 10.1 Hz, 1H), 8.91 (q, J = 9.9 Hz, 1H), 8.59 (s, 1H), 8.40 – 8.32 (m, 1H), 8.21 – 8.17 (m, 1H), 8.13 (s, 1H), 8.09 (dd, J = 7.6, 0.8 Hz, 1H), 7.93 – 7.90 (m, 1H), 7.87 (s, 1H), 7.84 (s, 1H), 7.70 (s, 1H), 7.68 (s, 1H), 7.61 (d, J = 8.2 Hz, 1H), 7.59 – 7.51 (m, 2H), 7.47 (t, J = 7.9 Hz, 1H), 4.52 (dt, J = 15.0, 7.6 Hz, 1H), 3.39 (d, J = 12.6 Hz, 2H), 3.11 – 2.98 (m, 2H), 2.46 (s, 3H), 2.20 – 2.13 (m, 4H).
f1 (ppm)

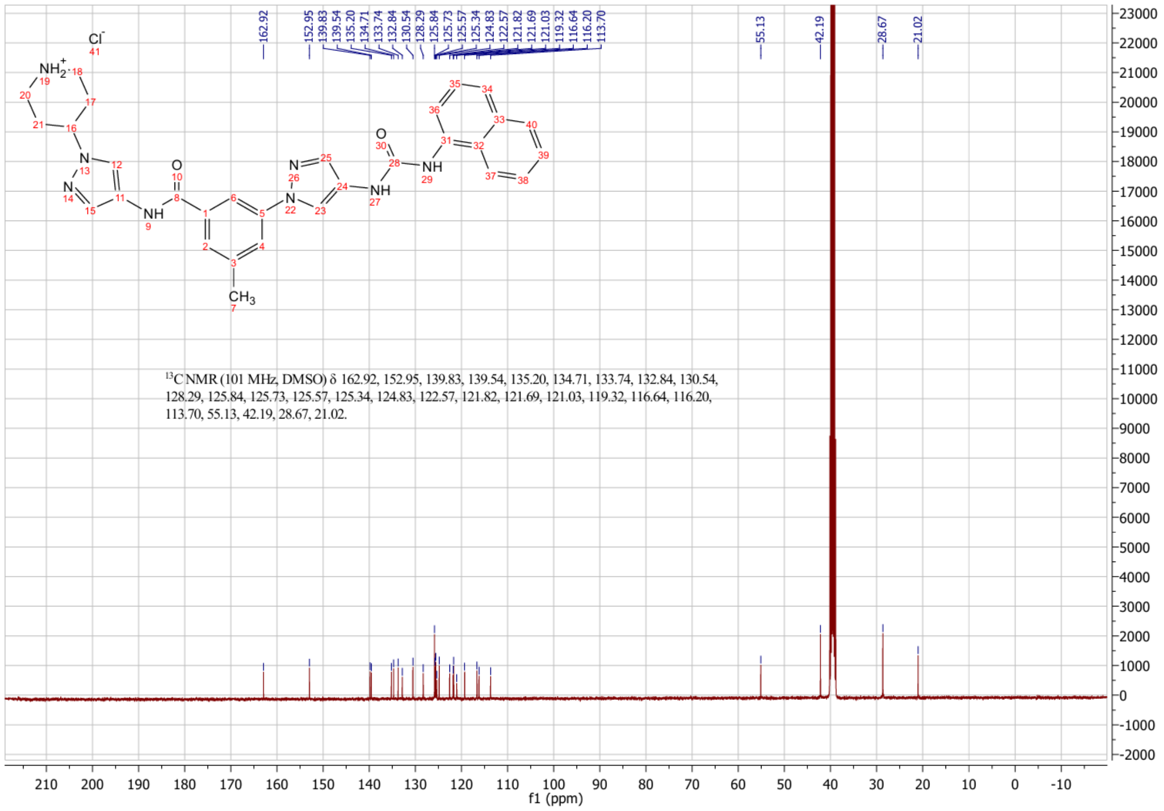
^{13}C NMR (101 MHz, DMSO) δ 162.92, 152.95, 139.83, 139.54, 135.20, 134.71, 133.74, 132.84, 130.54, 128.29, 125.84, 125.73, 125.57, 125.34, 124.83, 122.57, 121.82, 121.69, 121.03, 119.32, 116.64, 116.20, 113.70, 55.13, 42.19, 28.67, 21.02.
f1 (ppm)

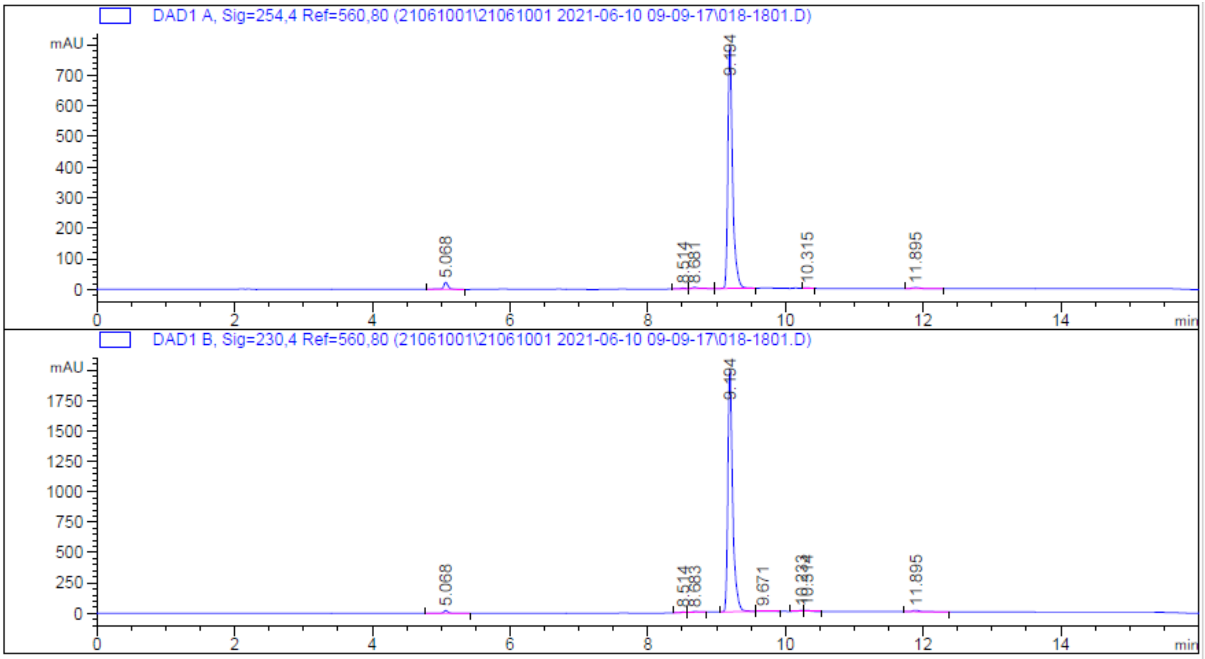

===

Area Percent Report

===

Sorted By : Signal
Multiplier: : 1.0000
Dilution: : 1.0000
Use Multiplier & Dilution Factor with ISTDs

Signal 1: DAD1 A, Sig=254,4 Ref=560,80

Peak #	RetTime [min]	Type	Width [min]	Area [mAU*s]	Height [mAU]	Area %
1	5.068	BB	0.0673	105.37148	23.06610	2.6710
2	8.514	BV	0.1084	19.06667	2.35557	0.4833
3	8.681	VB	0.0977	33.02364	4.60543	0.8371
4	9.194	BB	0.0693	3754.01050	792.63782	95.1570
5	10.315	BB	0.0636	5.53741	1.35754	0.1404
6	11.895	BB	0.1245	28.05929	3.32268	0.7112

Totals : 3945.06899 827.34513

Signal 2: DAD1 B, Sig=230,4 Ref=560,80

Peak #	RetTime [min]	Type	Width [min]	Area [mAU*s]	Height [mAU]	Area %
1	5.068	BB	0.0712	108.81443	22.18633	1.0926
2	8.514	BB	0.0652	13.39932	3.17770	0.1345
3	8.683	BB	0.0770	27.70141	5.13065	0.2781
4	9.194	BB	0.0742	9602.16504	1991.79932	96.4148
5	9.671	BB	0.1506	21.22669	1.84026	0.2131
6	10.233	BV	0.0789	42.26604	7.59442	0.4244
7	10.314	VB	0.0809	47.46747	8.53113	0.4766
8	11.895	BB	0.1225	96.17786	11.62692	0.9657

Totals : 9959.21827 2051.88673

===

*** End of Report ***

45c

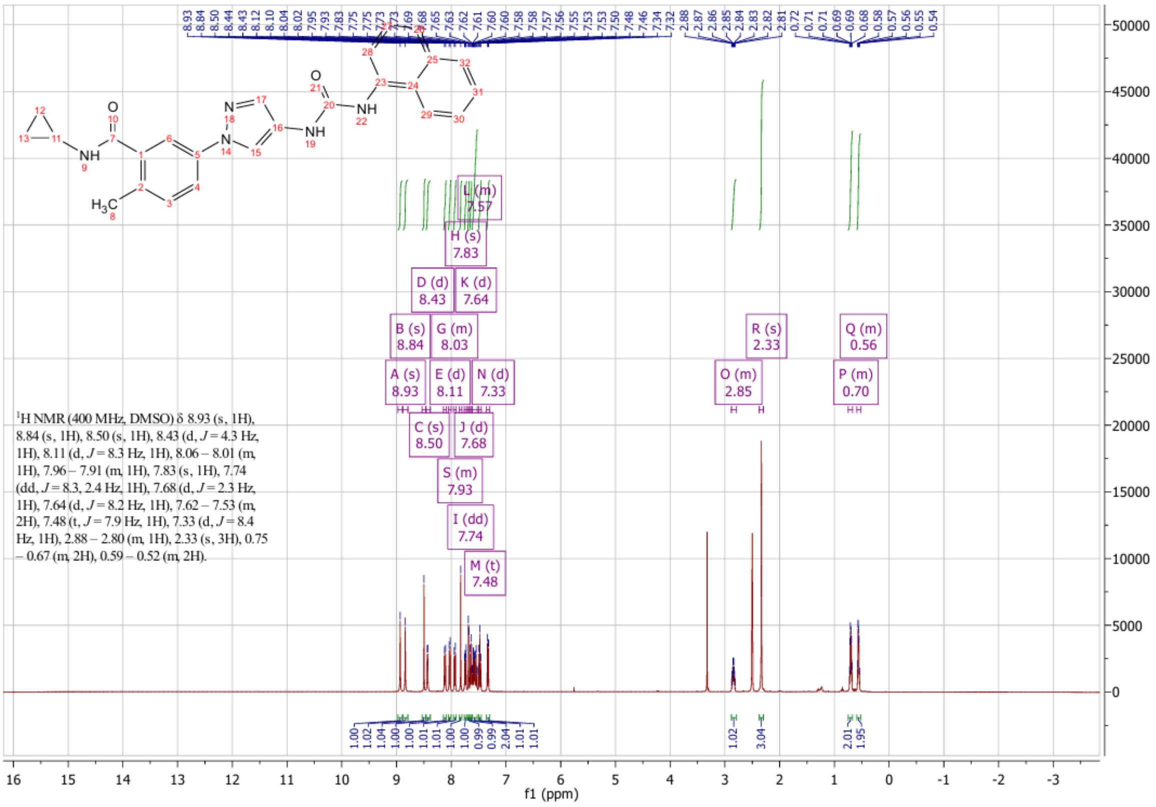
^{1}H NMR (400 MHz, DMSO) δ 8.93 (s, 1H), 8.84 (s, 1H), 8.50 (s, 1H), 8.43 (d, J = 4.3 Hz, 1H), 8.11 (d, J = 8.3 Hz, 1H), 8.06 – 8.01 (m, 1H), 7.96 – 7.91 (m, 1H), 7.83 (s, 1H), 7.74 (dd, J = 8.3, 2.4 Hz, 1H), 7.68 (d, J = 2.3 Hz, 1H), 7.64 (d, J = 8.2 Hz, 1H), 7.62 – 7.53 (m, 2H), 7.48 (t, J = 7.9 Hz, 1H), 7.33 (d, J = 8.4 Hz, 1H), 2.88 – 2.80 (m, 1H), 2.33 (s, 3H), 0.75 – 0.67 (m, 2H), 0.59 – 0.52 (m, 2H).
f1 (ppm)

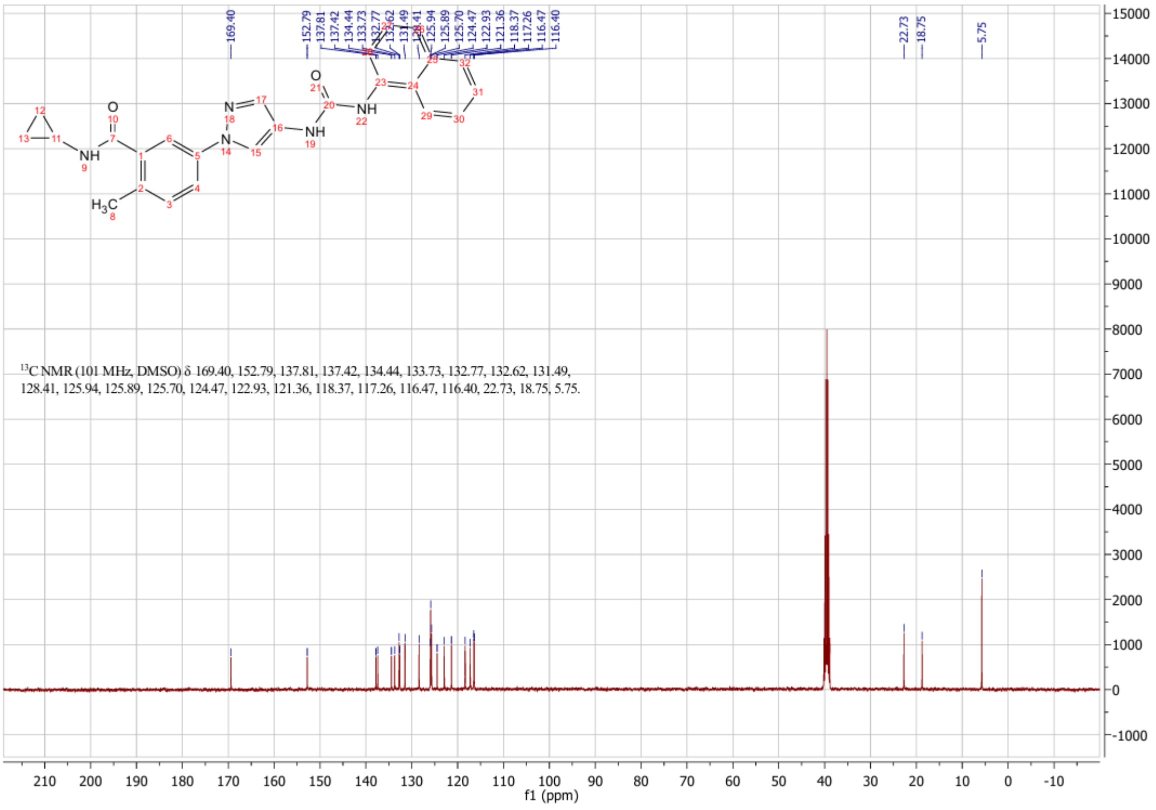
^{13}C NMR (101 MHz, DMSO) δ 169.40, 152.79, 137.81, 137.42, 134.44, 133.73, 132.77, 132.62, 131.49, 128.41, 125.94, 125.89, 125.70, 124.47, 122.93, 121.36, 118.37, 117.26, 116.47, 116.40, 22.73, 18.75, 5.75.
f1 (ppm)

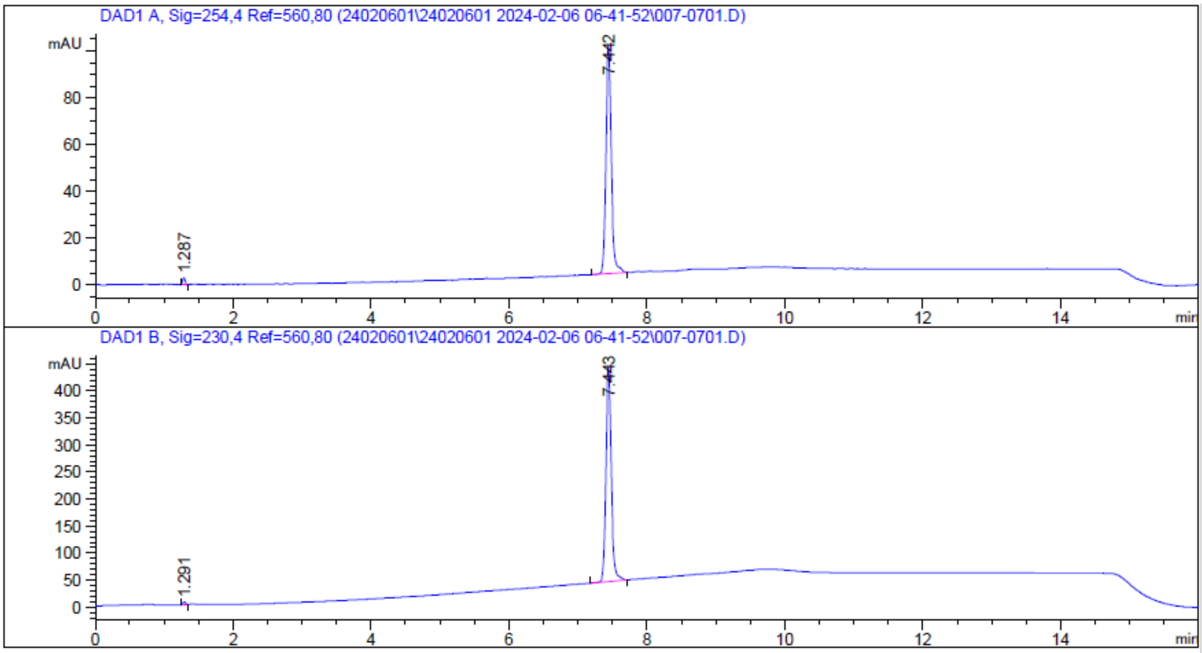

===

Area Percent Report

===

Sorted By : Signal
Multiplier: : 1.0000
Dilution: : 1.0000
Use Multiplier & Dilution Factor with ISTDs

Signal 1: DAD1 A, Sig=254,4 Ref=560,80

Peak #	RetTime [min]	Type	Width [min]	Area [mAU*s]	Height [mAU]	Area %
1	1.287	BB	0.0407	7.42581	3.01896	1.3974
2	7.442	BB	0.0826	523.98212	97.64817	98.6026

Totals : 531.40792 100.66713

Signal 2: DAD1 B, Sig=230,4 Ref=560,80

Peak #	RetTime [min]	Type	Width [min]	Area [mAU*s]	Height [mAU]	Area %
1	1.291	BB	0.0429	13.72757	5.18589	0.6408
2	7.443	BB	0.0825	2128.58276	397.52322	99.3592

Totals : 2142.31033 402.70911

===

*** End of Report ***

11.8 Table 7

51a

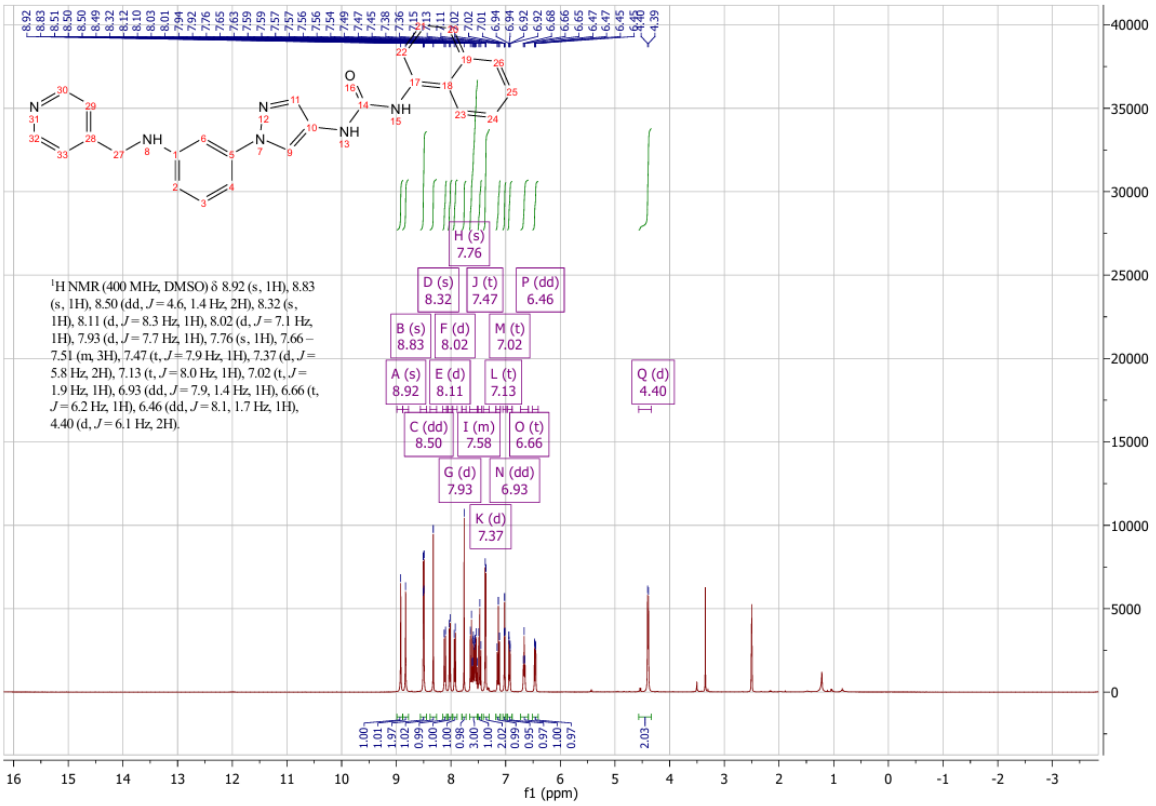

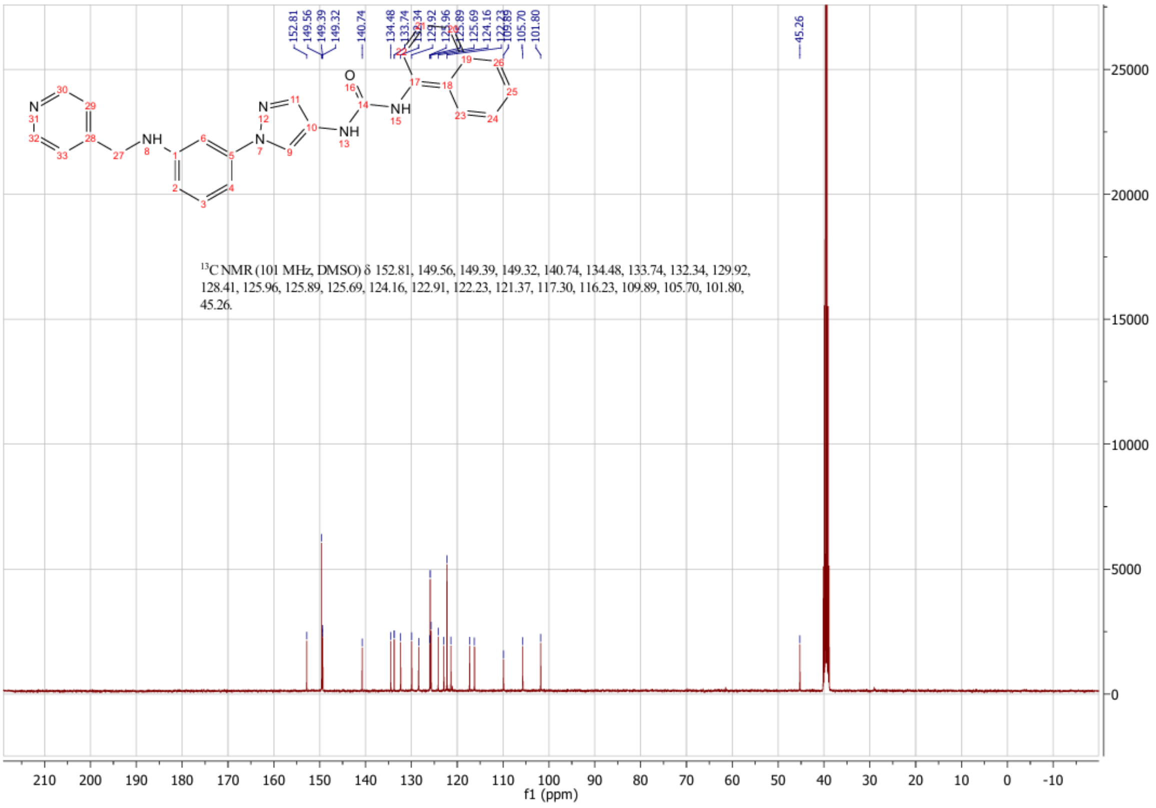

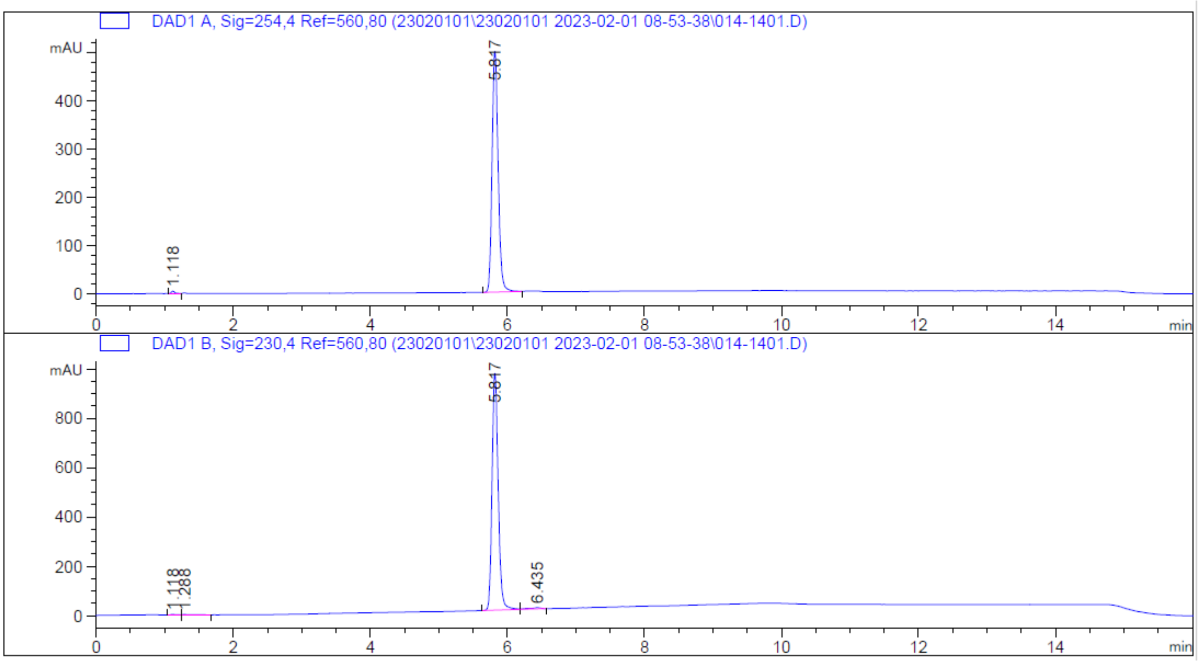

===

Area Percent Report

===

Sorted By : Signal
Multiplier: : 1.0000
Dilution: : 1.0000
Use Multiplier & Dilution Factor with ISTDs

Signal 1: DAD1 A, Sig=254,4 Ref=560,80

Peak #	RetTime [min]	Type	Width [min]	Area [mAU*s]	Height [mAU]	Area %
1	1.118	BV	0.0515	16.61167	4.91216	0.5392
2	5.817	BB	0.0954	3064.22656	500.70663	99.4608

Totals : 3080.83823 505.61880

Signal 2: DAD1 B, Sig=230,4 Ref=560,80

Peak #	RetTime [min]	Type	Width [min]	Area [mAU*s]	Height [mAU]	Area %
1	1.118	BV	0.0516	10.98714	3.23630	0.1816
2	1.288	VB	0.0723	16.17384	3.12686	0.2673
3	5.817	BV	0.0963	5960.82129	961.63599	98.5011
4	6.435	VB	0.1719	63.54267	4.94439	1.0500

Totals : 6051.52494 972.94354

===

*** End of Report ***

51b

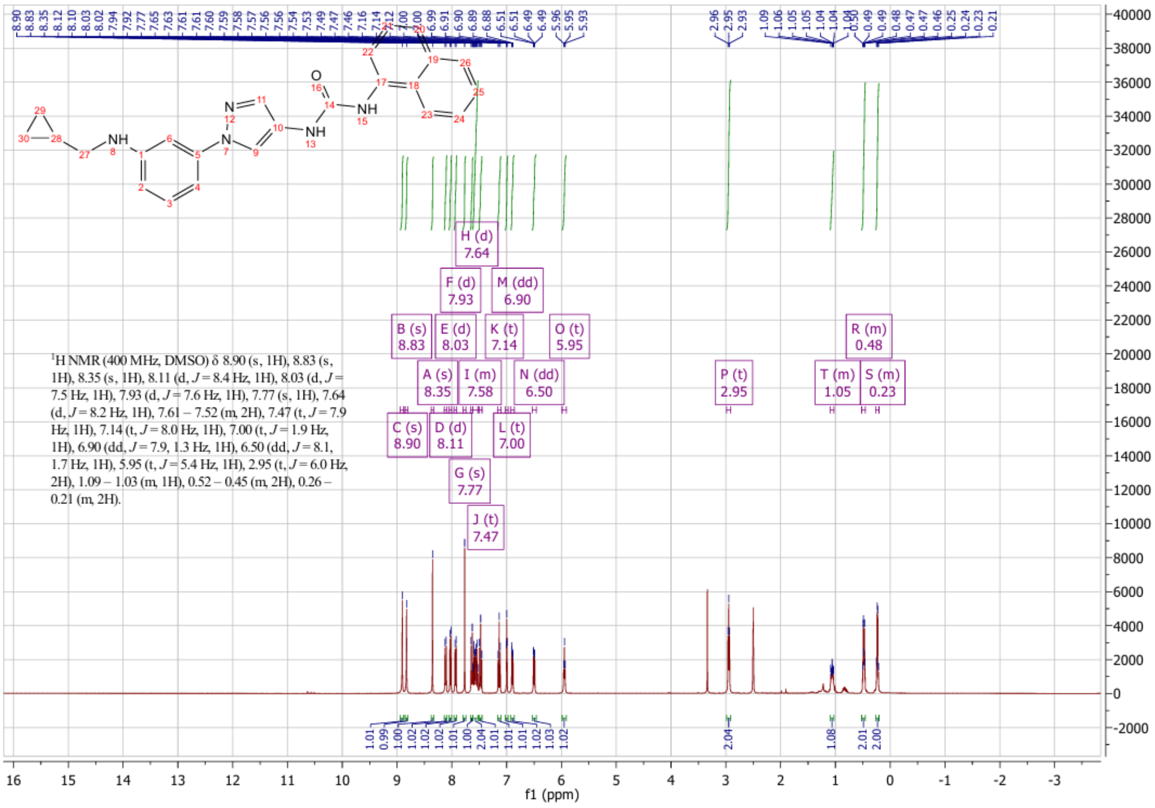
^{1}H NMR (400 MHz, DMSO) δ 8.90 (s, 1H), 8.83 (s, 1H), 8.35 (s, 1H), 8.11 (d, J = 8.4 Hz, 1H), 8.03 (d, J = 7.5 Hz, 1H), 7.93 (d, J = 7.6 Hz, 1H), 7.77 (s, 1H), 7.64 (d, J = 8.2 Hz, 1H), 7.61 – 7.52 (m, 2H), 7.47 (t, J = 7.9 Hz, 1H), 7.14 (t, J = 8.0 Hz, 1H), 7.00 (t, J = 1.9 Hz, 1H), 6.90 (dd, J = 7.9, 1.3 Hz, 1H), 6.50 (dd, J = 8.1, 1.7 Hz, 1H), 5.95 (t, J = 5.4 Hz, 1H), 2.95 (t, J = 6.0 Hz, 2H), 1.09 – 1.03 (m, 1H), 0.52 – 0.45 (m, 2H), 0.26 – 0.21 (m, 2H).
f1 (ppm)

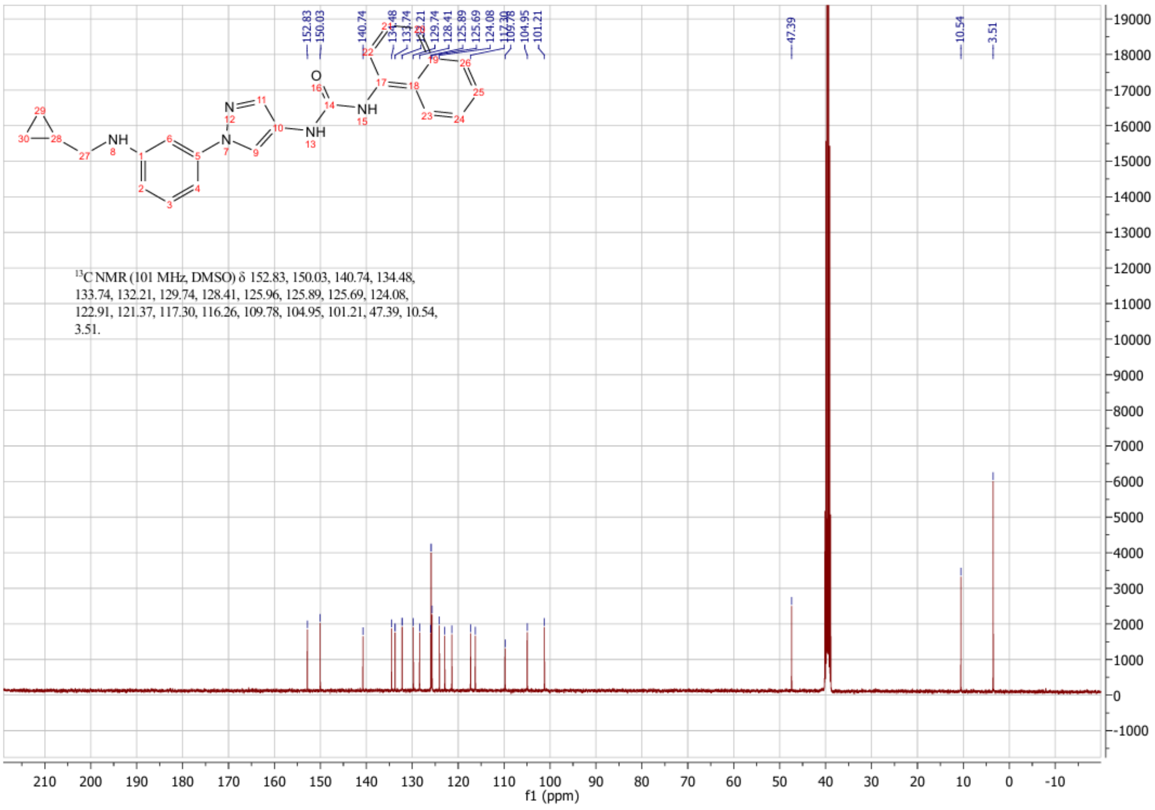
^{13}C NMR (101 MHz, DMSO) δ 152.83, 150.03, 140.74, 134.48, 133.74, 132.21, 129.74, 128.41, 125.96, 125.89, 125.69, 124.08, 122.91, 121.37, 117.30, 116.26, 109.78, 104.95, 101.21, 47.39, 10.54, 3.51.
f1 (ppm)

51c

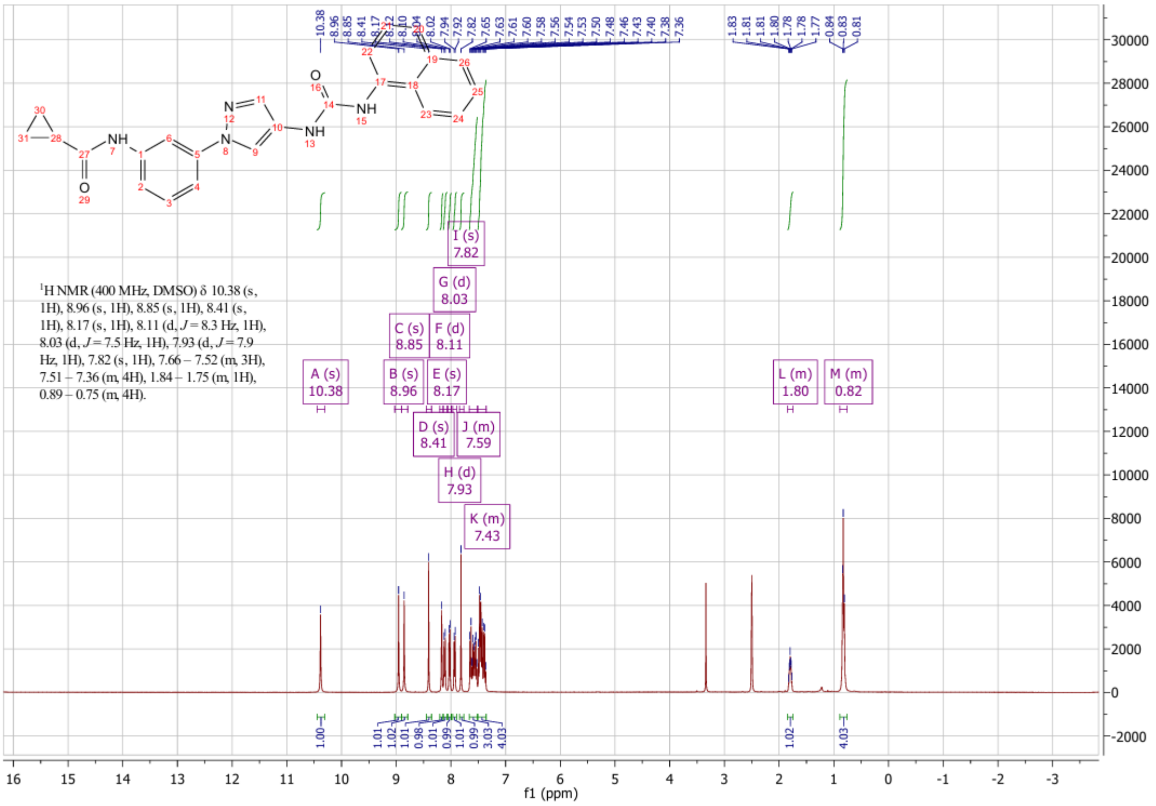
1H NMR (400 MHz, DMSO) δ 10.38 (s, 1H), 8.96 (s, 1H), 8.85 (s, 1H), 8.41 (s, 1H), 8.17 (s, 1H), 8.11 (d, J = 8.3 Hz, 1H), 8.03 (d, J = 7.5 Hz, 1H), 7.93 (d, J = 7.9 Hz, 1H), 7.82 (s, 1H), 7.66 – 7.52 (m, 3H), 7.51 – 7.36 (m, 4H), 1.84 – 1.75 (m, 1H), 0.89 – 0.75 (m, 4H).
f1 (ppm)

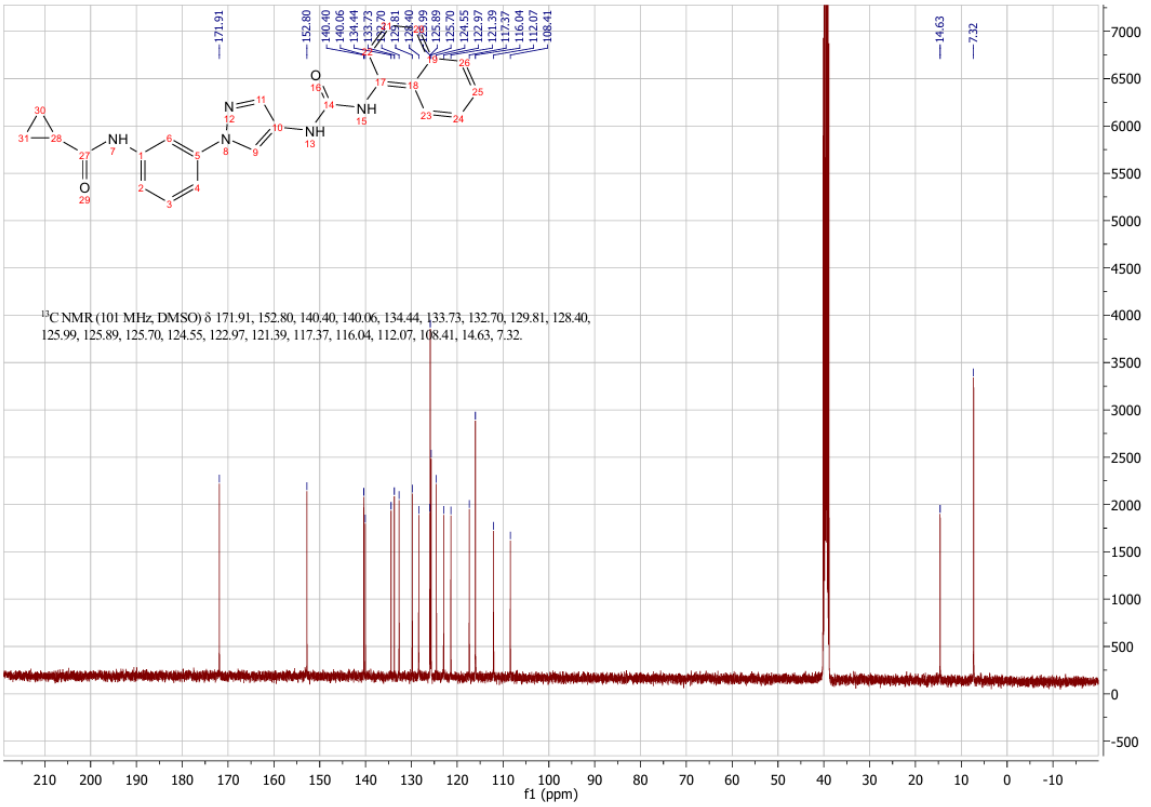
13C NMR (101 MHz, DMSO) δ 171.91, 152.80, 140.40, 140.06, 134.44, 133.73, 132.70, 129.81, 128.40, 125.99, 125.89, 125.70, 124.55, 122.97, 121.39, 117.37, 116.04, 112.07, 108.41, 14.63, 7.32.
f1 (ppm)

51d

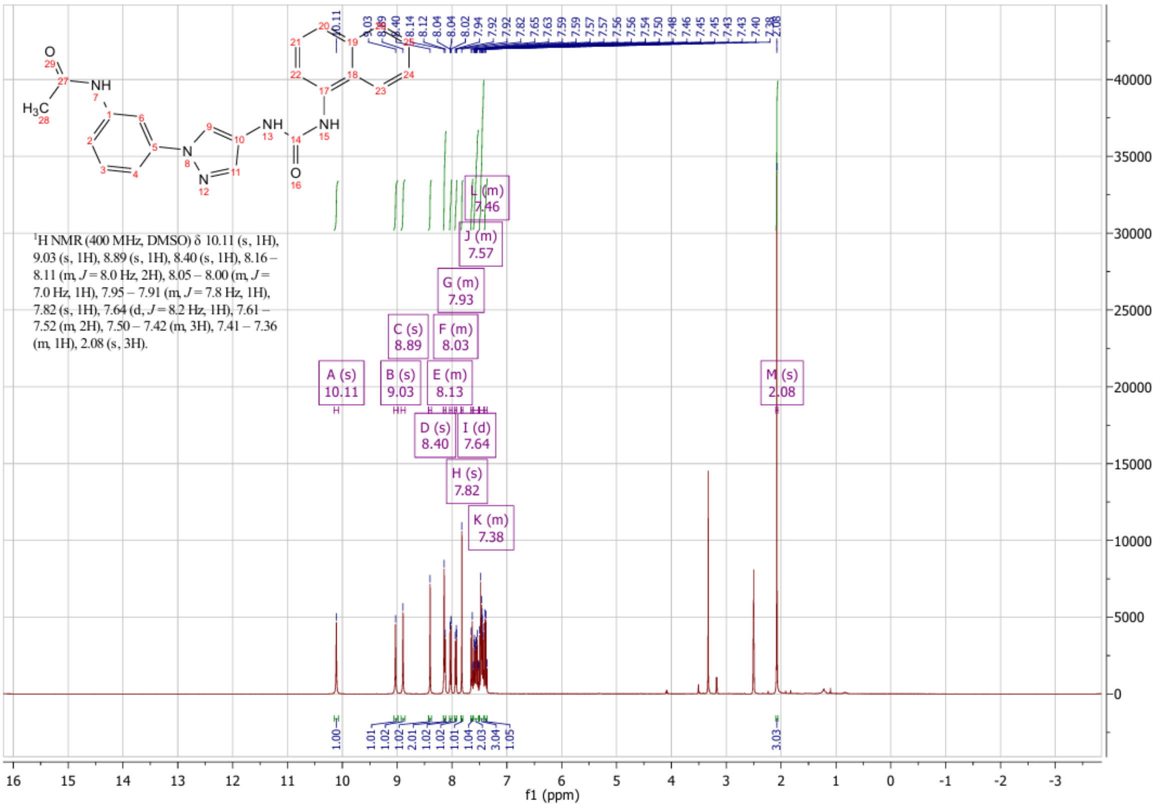

^{1}H NMR (400 MHz, DMSO) δ 10.11 (s, 1H), 9.03 (s, 1H), 8.89 (s, 1H), 8.40 (s, 1H), 8.16 – 8.11 (m, J = 8.0 Hz, 2H), 8.05 – 8.00 (m, J = 7.0 Hz, 1H), 7.95 – 7.91 (m, J = 7.8 Hz, 1H), 7.82 (s, 1H), 7.64 (d, J = 8.2 Hz, 1H), 7.61 – 7.52 (m, 2H), 7.50 – 7.42 (m, 3H), 7.41 – 7.36 (m, 1H), 2.08 (s, 3H).
f1 (ppm)

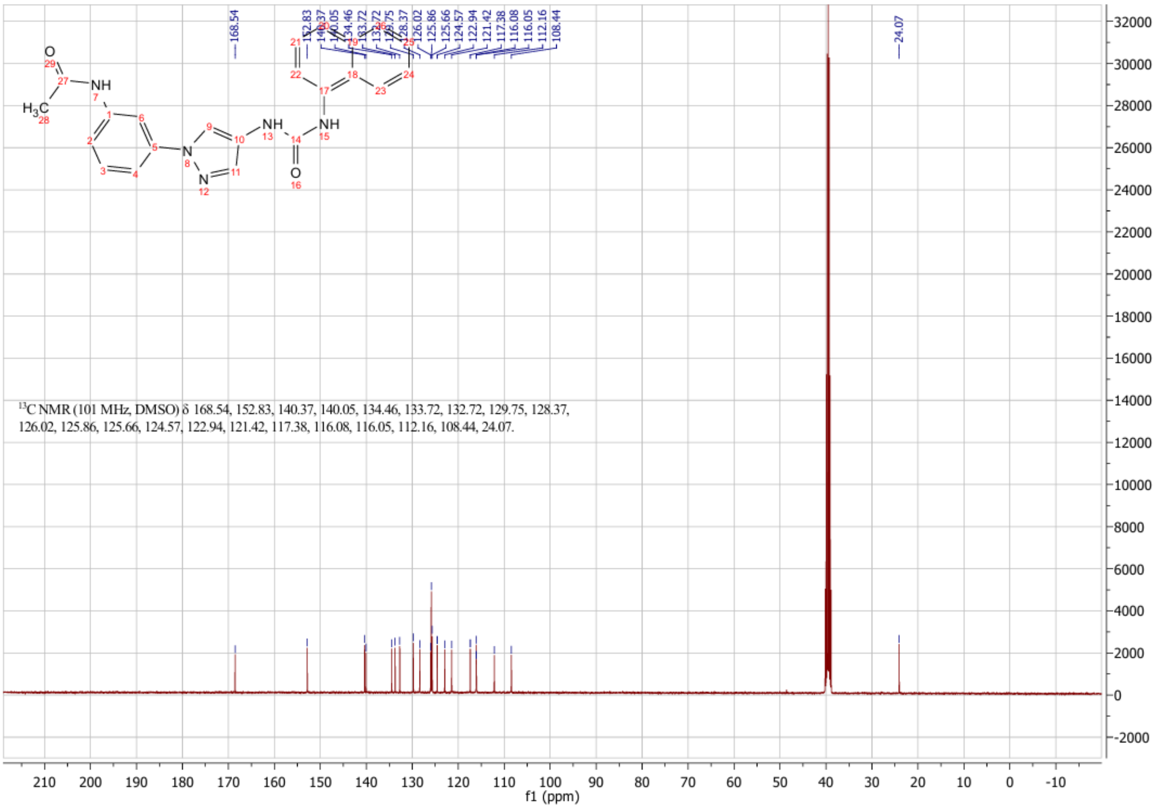

^{13}C NMR (101 MHz, DMSO) δ 168.54, 152.83, 140.37, 140.05, 134.46, 133.72, 132.72, 129.75, 128.37, 126.02, 125.86, 125.66, 124.57, 122.94, 121.42, 117.38, 116.08, 116.05, 112.16, 108.44, 24.07.
f1 (ppm)

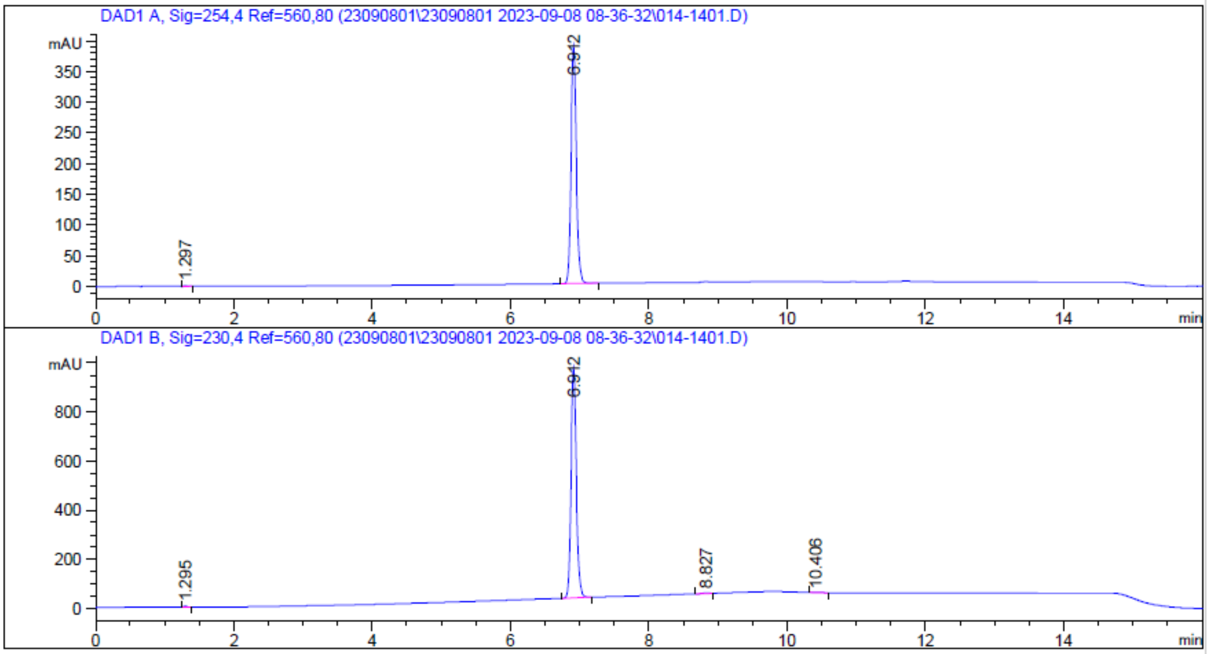

===

Area Percent Report

===

Sorted By : Signal
Multiplier: : 1.0000
Dilution: : 1.0000
Use Multiplier & Dilution Factor with ISTDs

Signal 1: DAD1 A, Sig=254,4 Ref=560,80

Peak #	RetTime [min]	Type	Width [min]	Area [mAU*s]	Height [mAU]	Area %
1	1.297	BB	0.0484	5.32048	1.70368	0.2603
2	6.912	BB	0.0815	2038.52417	386.95898	99.7397

Totals : 2043.84465 388.66266

Signal 2: DAD1 B, Sig=230,4 Ref=560,80

Peak #	RetTime [min]	Type	Width [min]	Area [mAU*s]	Height [mAU]	Area %
1	1.295	BB	0.0475	12.64789	4.15229	0.2541
2	6.912	BB	0.0815	4940.32959	937.41241	99.2469
3	8.827	BB	0.0777	15.31776	3.09934	0.3077
4	10.406	BB	0.0875	9.52053	1.69493	0.1913

Totals : 4977.81577 946.35897

===

*** End of Report ***

51e

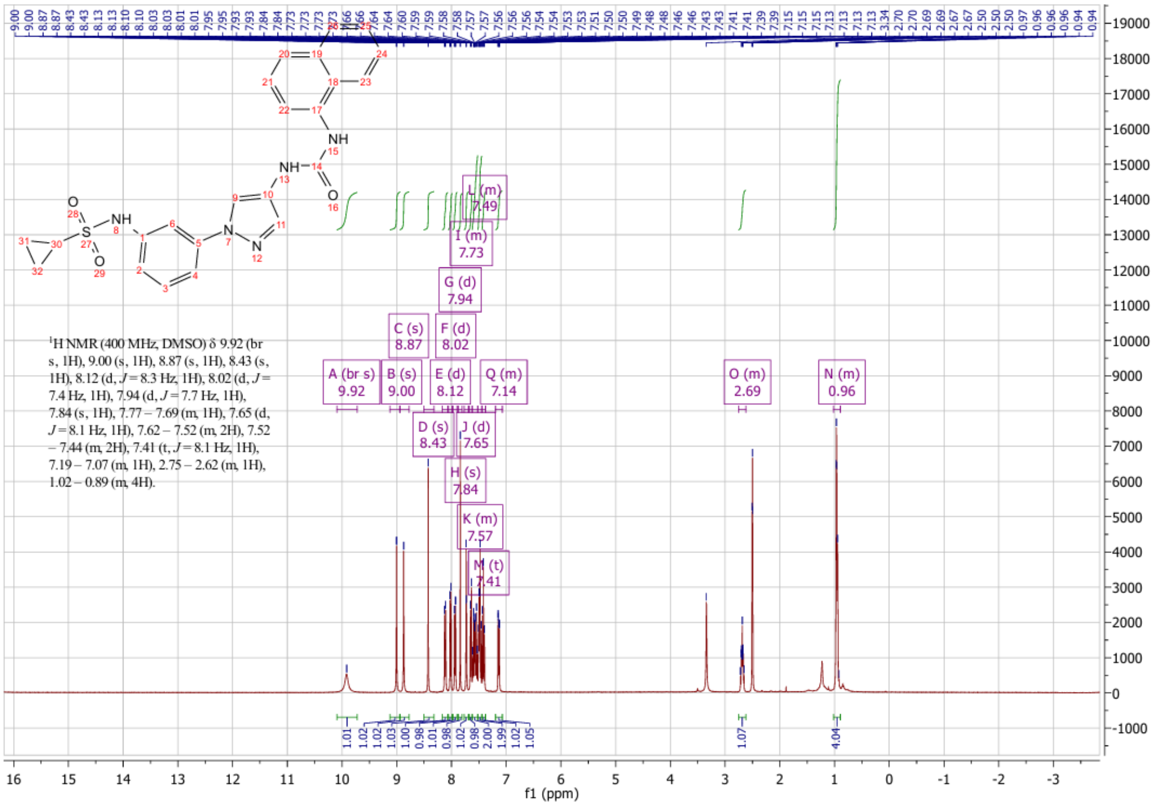
^{1}H NMR (400 MHz, DMSO) δ 9.92 (br s, 1H), 9.00 (s, 1H), 8.87 (s, 1H), 8.43 (s, 1H), 8.12 (d, J = 8.3 Hz, 1H), 8.02 (d, J = 7.4 Hz, 1H), 7.94 (d, J = 7.7 Hz, 1H), 7.84 (s, 1H), 7.77 – 7.69 (m, 1H), 7.65 (d, J = 8.1 Hz, 1H), 7.62 – 7.52 (m, 2H), 7.52 – 7.44 (m, 2H), 7.41 (t, J = 8.1 Hz, 1H), 7.19 – 7.07 (m, 1H), 2.75 – 2.62 (m, 1H), 1.02 – 0.89 (m, 4H).
f1 (ppm)

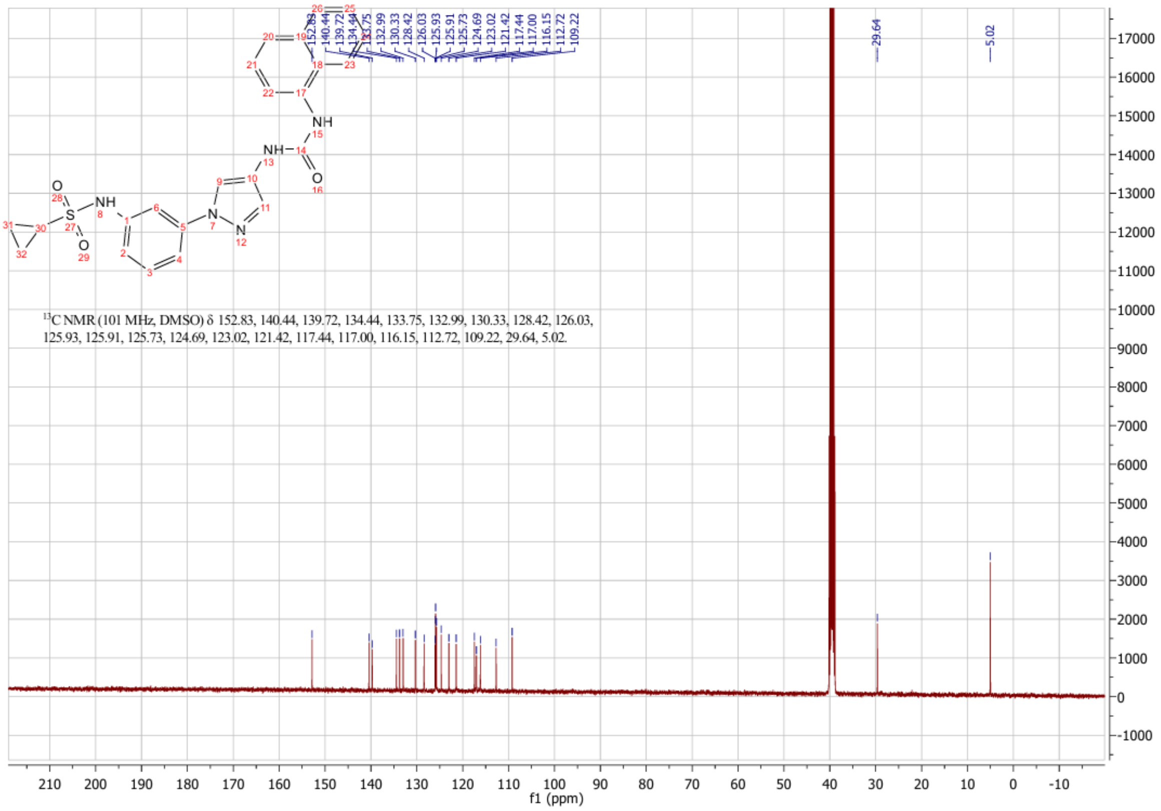
^{13}C NMR (101 MHz, DMSO) δ 152.83, 140.44, 139.72, 134.44, 133.75, 132.99, 130.33, 128.42, 126.03, 125.93, 125.91, 125.73, 124.69, 123.02, 121.42, 117.44, 117.00, 116.15, 112.72, 109.22, 29.64, 5.02.
f1 (ppm)

51f

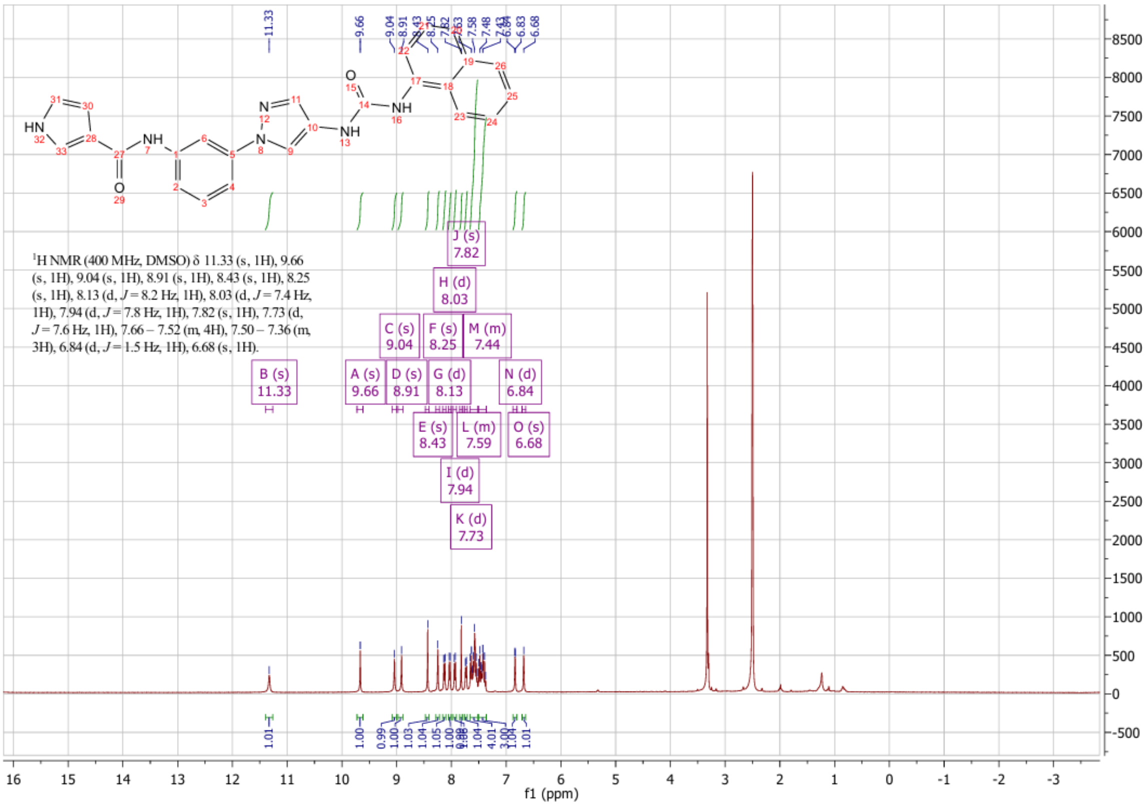

^{1}H NMR (400 MHz, DMSO) δ 11.33 (s, 1H), 9.66 (s, 1H), 9.04 (s, 1H), 8.91 (s, 1H), 8.43 (s, 1H), 8.25 (s, 1H), 8.13 (d, J = 8.2 Hz, 1H), 8.03 (d, J = 7.4 Hz, 1H), 7.94 (d, J = 7.8 Hz, 1H), 7.82 (s, 1H), 7.73 (d, J = 7.6 Hz, 1H), 7.66 – 7.52 (m, 4H), 7.50 – 7.36 (m, 3H), 6.84 (d, J = 1.5 Hz, 1H), 6.68 (s, 1H).
f1 (ppm)

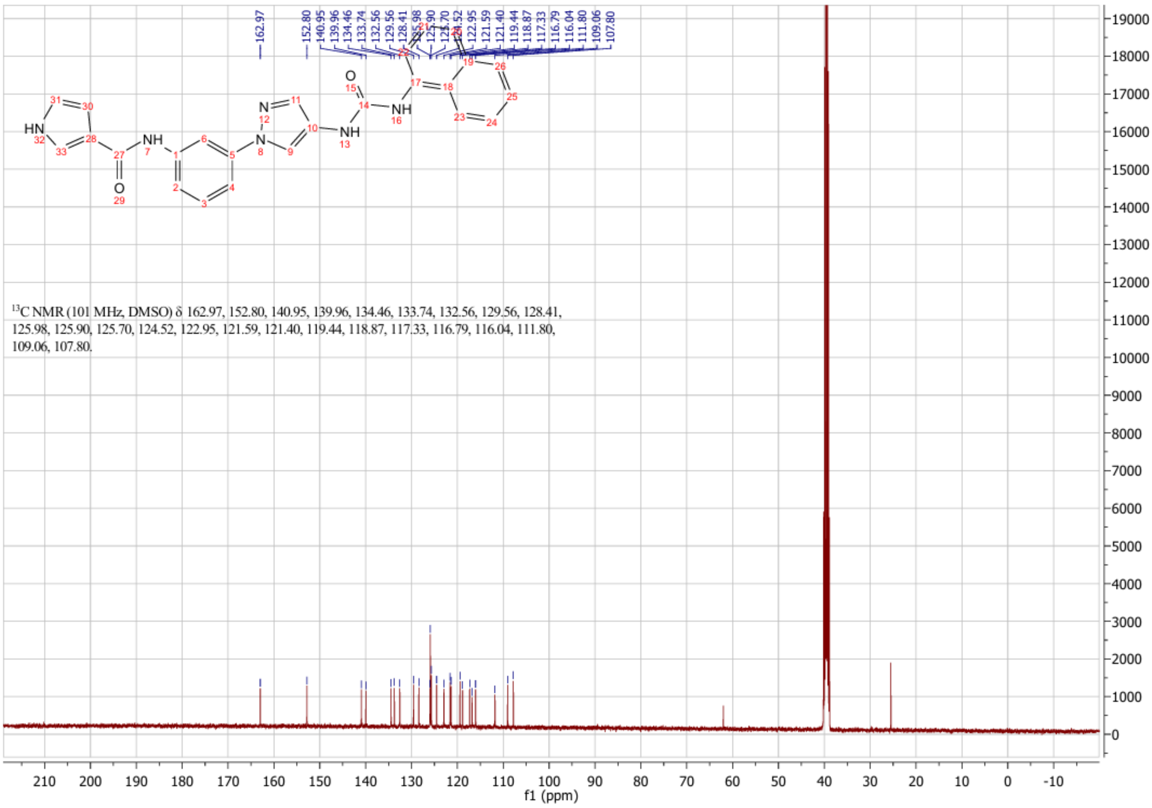

^{13}C NMR (101 MHz, DMSO) δ 162.97, 152.80, 140.95, 139.96, 134.46, 133.74, 132.56, 129.56, 128.41, 125.98, 125.90, 125.70, 124.52, 122.95, 121.59, 121.40, 119.44, 118.87, 117.33, 116.79, 116.04, 111.80, 109.06, 107.80.
f1 (ppm)

51g

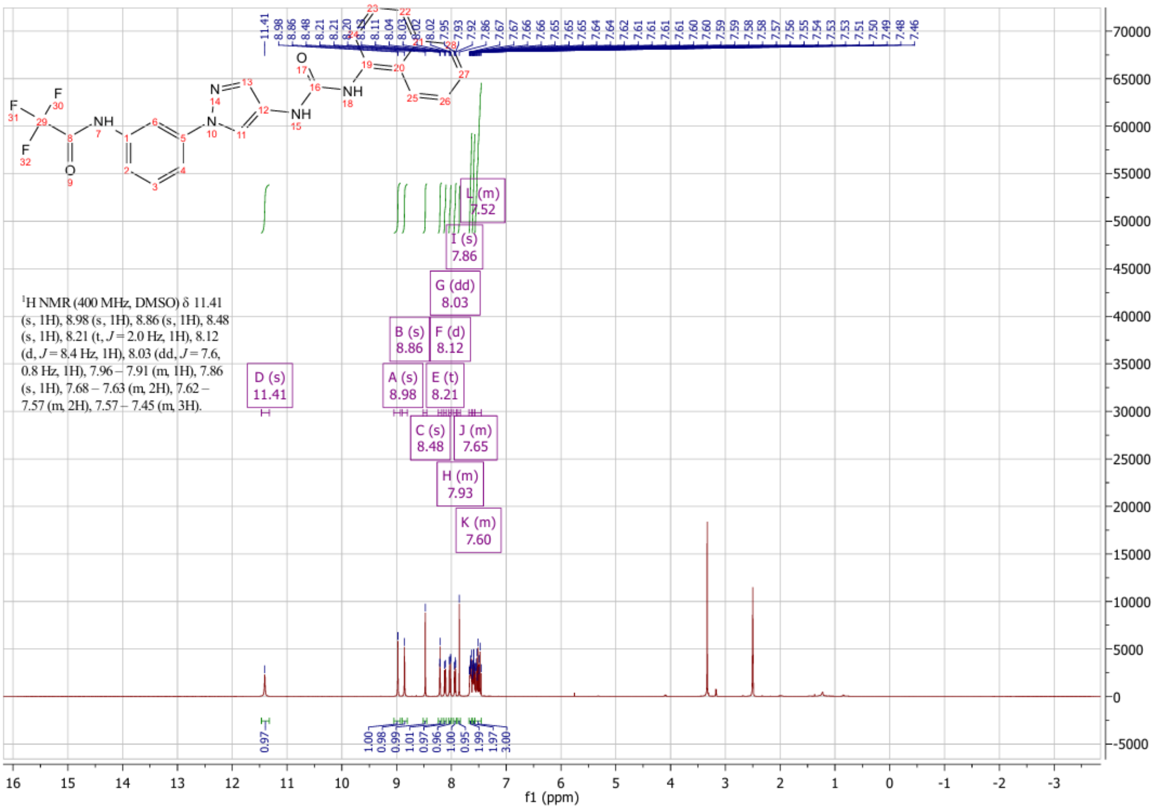

¹H NMR (400 MHz, DMSO) δ 11.41 (s, 1H), 8.98 (s, 1H), 8.86 (s, 1H), 8.48 (s, 1H), 8.21 (t, J = 2.0 Hz, 1H), 8.12 (d, J = 8.4 Hz, 1H), 8.03 (dd, J = 7.6, 0.8 Hz, 1H), 7.96 – 7.91 (m, 1H), 7.86 (s, 1H), 7.68 – 7.63 (m, 2H), 7.62 – 7.57 (m, 2H), 7.57 – 7.45 (m, 3H).
f1 (ppm)

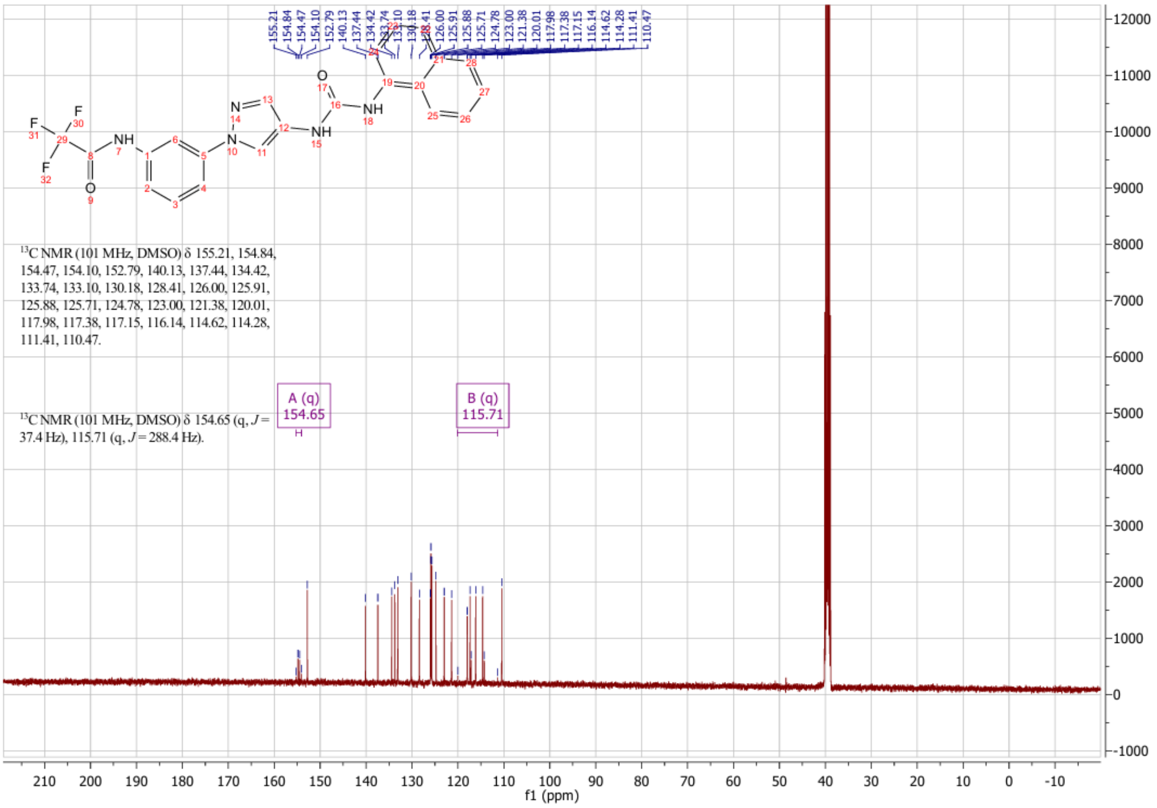

¹³C NMR (101 MHz, DMSO) δ 155.21, 154.84, 154.47, 154.10, 152.79, 140.13, 137.44, 134.42, 133.74, 133.10, 130.18, 128.41, 126.00, 125.91, 125.88, 125.71, 124.78, 123.00, 121.38, 120.01, 117.98, 117.38, 117.15, 116.14, 114.62, 114.28, 111.41, 110.47.
¹³C NMR (101 MHz, DMSO) δ 154.65 (q, J = 37.4 Hz), 115.71 (q, J = 288.4 Hz).
f1 (ppm)

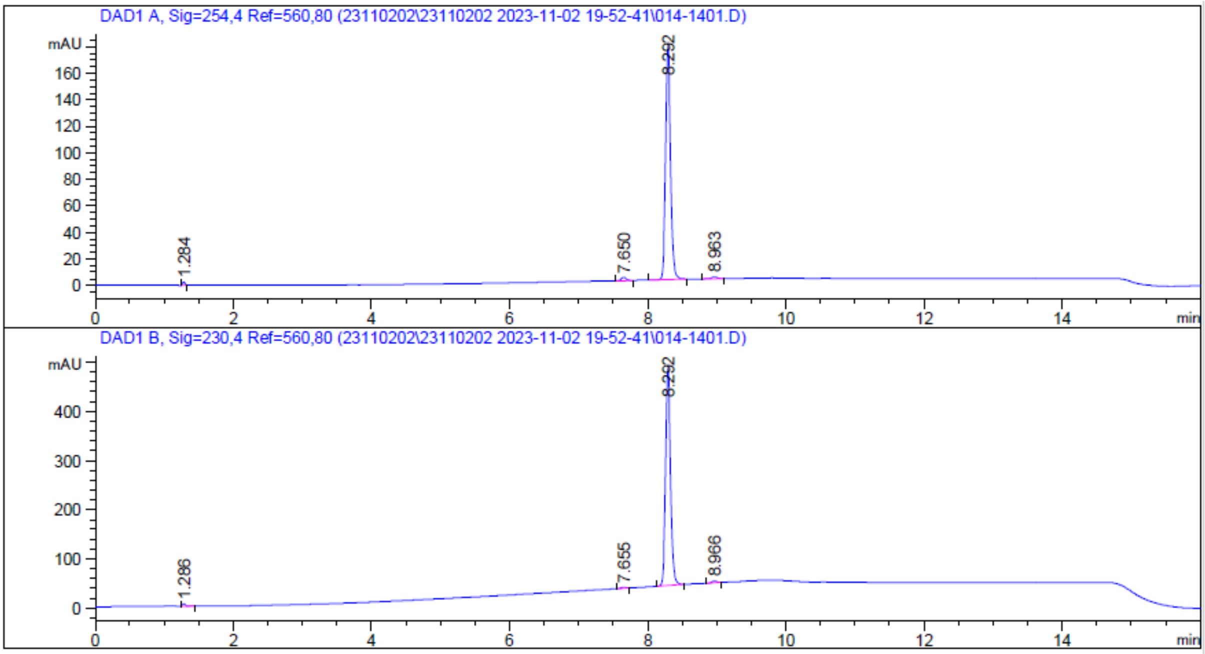

===

Area Percent Report

===

Sorted By : Signal
Multiplier: : 1.0000
Dilution: : 1.0000
Use Multiplier & Dilution Factor with ISTDs

Signal 1: DAD1 A, Sig=254,4 Ref=560,80

Peak #	RetTime [min]	Type	Width [min]	Area [mAU*s]	Height [mAU]	Area %
1	1.284	BB	0.0405	6.15406	2.51617	0.6804
2	7.650	BB	0.0775	12.25677	2.48628	1.3552
3	8.292	BB	0.0762	877.95807	176.07071	97.0741
4	8.963	BB	0.0908	8.05187	1.36511	0.8903

Totals : 904.42077 182.43827

Signal 2: DAD1 B, Sig=230,4 Ref=560,80

Peak #	RetTime [min]	Type	Width [min]	Area [mAU*s]	Height [mAU]	Area %
1	1.286	BB	0.0469	13.76696	4.59679	0.6142
2	7.655	BB	0.0737	9.05878	1.96808	0.4042
3	8.292	BB	0.0761	2200.78516	442.17752	98.1905
4	8.966	BB	0.0801	17.73043	3.44225	0.7911

Totals : 2241.34132 452.18464

===

*** End of Report ***

11.9 Table 8

56a

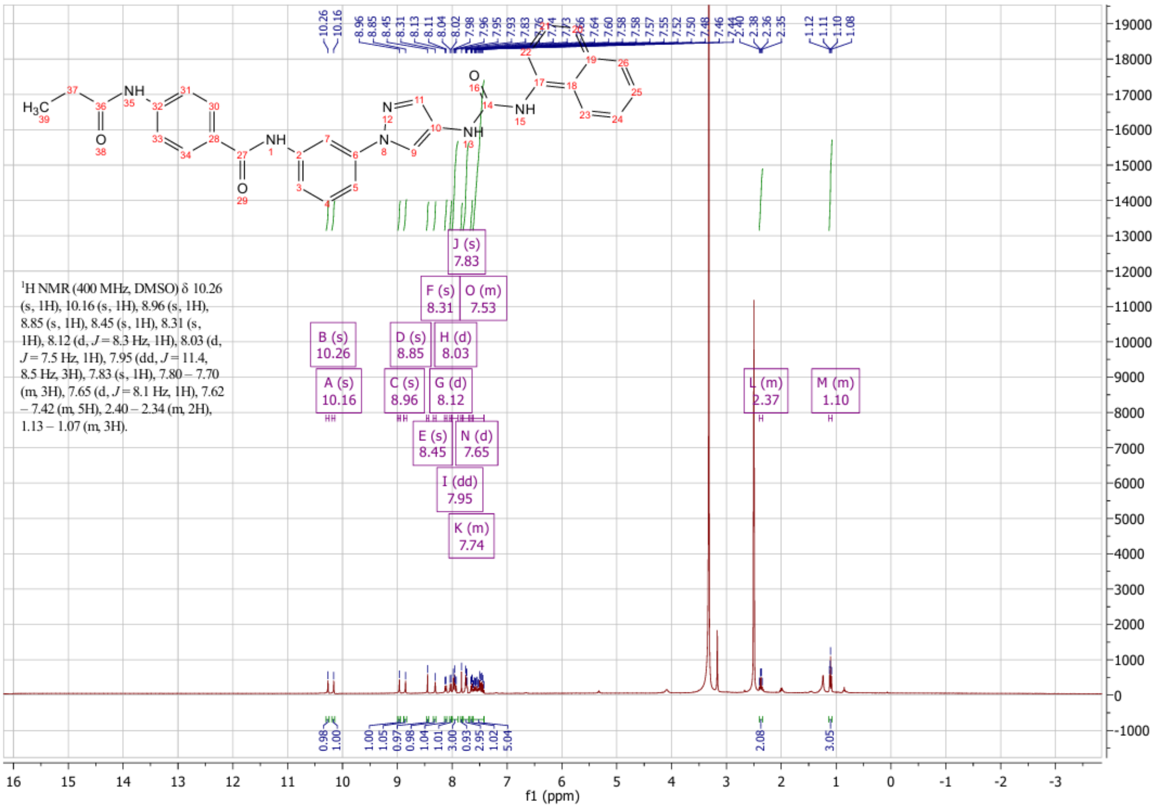

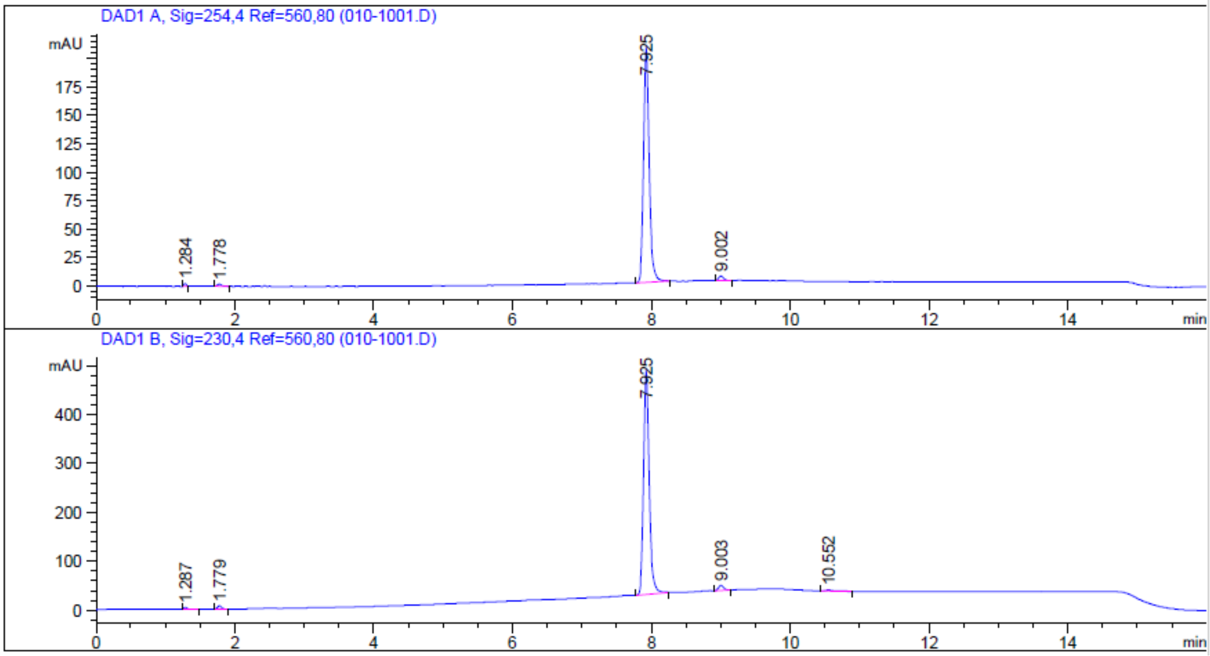

==

Area Percent Report

==

Sorted By : Signal
Multiplier: : 1.0000
Dilution: : 1.0000
Use Multiplier & Dilution Factor with ISTDs

Signal 1: DAD1 A, Sig=254,4 Ref=560,80

Peak #	RetTime [min]	Type	Width [min]	Area [mAU*s]	Height [mAU]	Area %
1	1.284	BB	0.0399	5.83272	2.44171	0.4834
2	1.778	BB	0.0638	8.48761	2.07215	0.7034
3	7.925	BB	0.0858	1171.38672	207.76187	97.0739
4	9.002	BB	0.0769	20.98932	4.15838	1.7394

Totals : 1206.69636 216.43412

Signal 2: DAD1 B, Sig=230,4 Ref=560,80

Peak #	RetTime [min]	Type	Width [min]	Area [mAU*s]	Height [mAU]	Area %
1	1.287	BB	0.0493	11.63523	3.63644	0.4295
2	1.779	BB	0.0566	27.05669	7.41308	0.9989
3	7.925	BB	0.0859	2597.25854	459.68405	95.8855
4	9.003	BB	0.0814	56.51299	10.74681	2.0863
5	10.552	BB	0.1078	16.24565	2.15865	0.5998

Totals : 2708.70911 483.63903

==

*** End of Report ***

56b

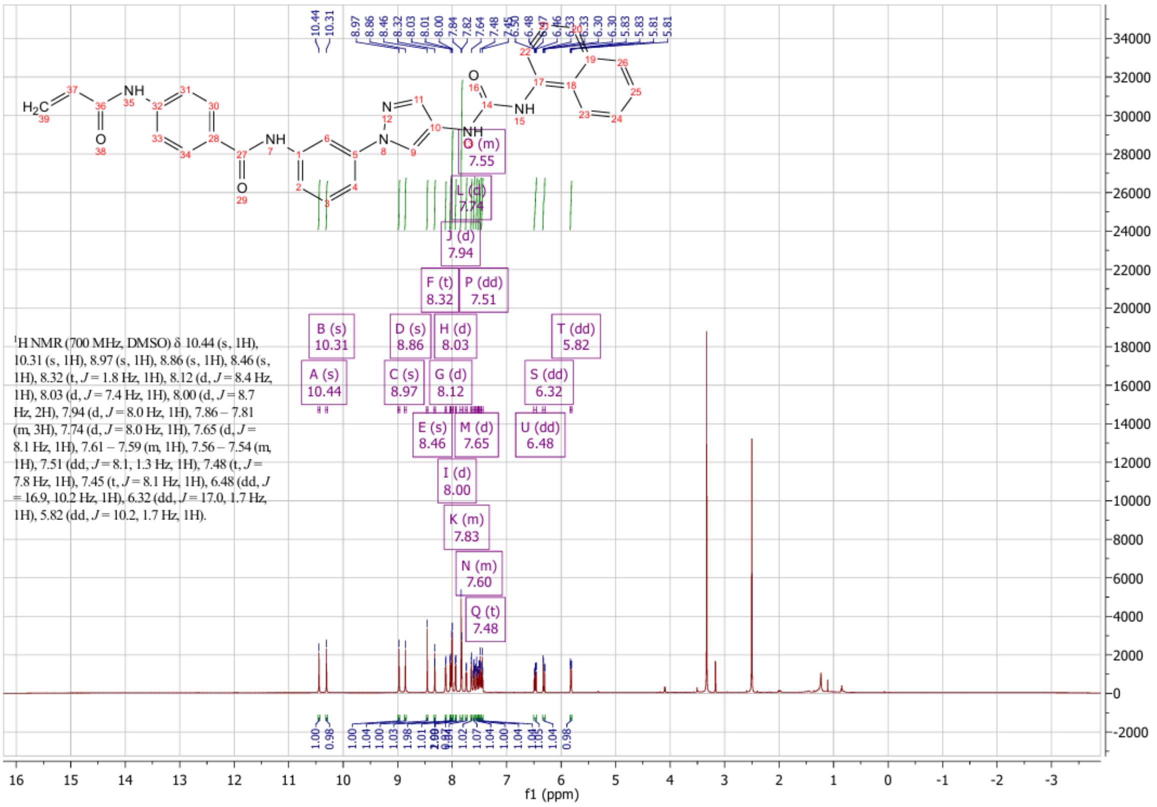

1H NMR (700 MHz, DMSO) δ 10.44 (s, 1H), 10.31 (s, 1H), 8.97 (s, 1H), 8.86 (s, 1H), 8.46 (s, 1H), 8.32 (t, J = 1.8 Hz, 1H), 8.12 (d, J = 8.4 Hz, 1H), 8.03 (d, J = 7.4 Hz, 1H), 8.00 (d, J = 8.7 Hz, 2H), 7.94 (d, J = 8.0 Hz, 1H), 7.86 – 7.81 (m, 3H), 7.74 (d, J = 8.0 Hz, 1H), 7.65 (d, J = 8.1 Hz, 1H), 7.61 – 7.59 (m, 1H), 7.56 – 7.54 (m, 1H), 7.51 (dd, J = 8.1, 1.3 Hz, 1H), 7.48 (t, J = 7.8 Hz, 1H), 7.45 (t, J = 8.1 Hz, 1H), 6.48 (dd, J = 16.9, 10.2 Hz, 1H), 6.32 (dd, J = 17.0, 1.7 Hz, 1H), 5.82 (dd, J = 10.2, 1.7 Hz, 1H).
f1 (ppm)

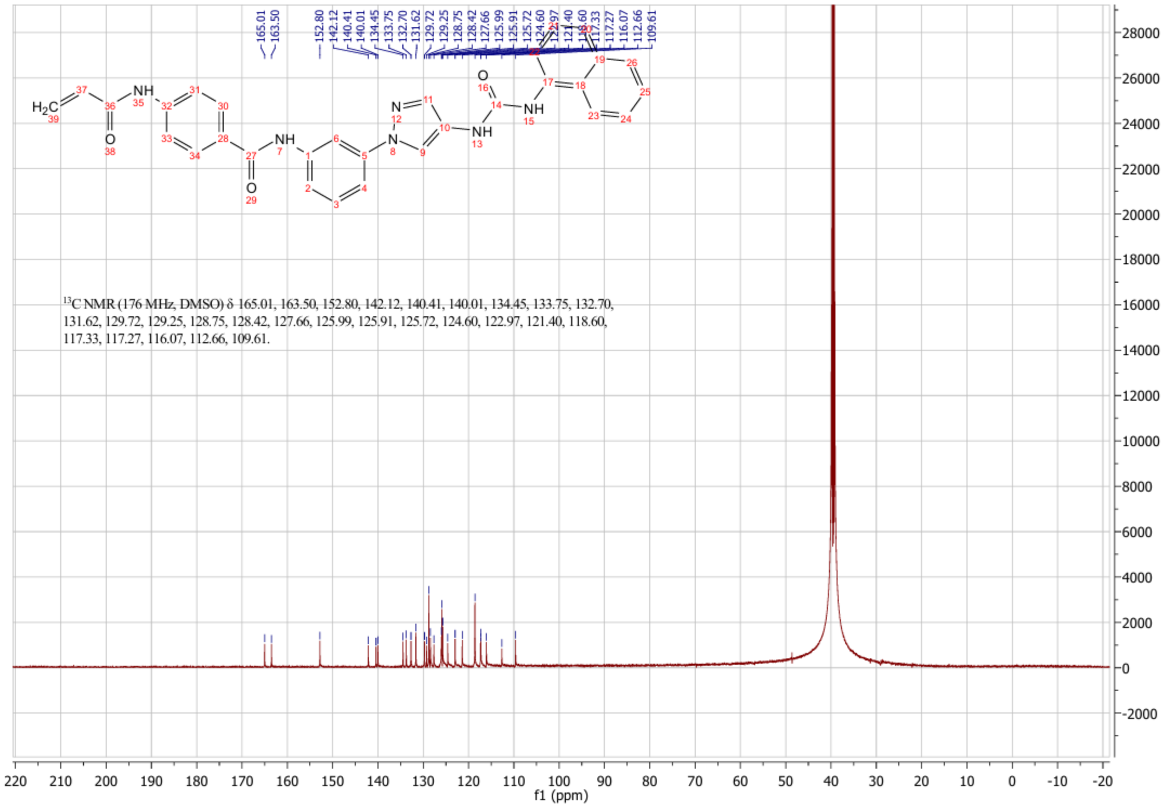

13C NMR (176 MHz, DMSO) δ 165.01, 163.50, 152.80, 142.12, 140.41, 140.01, 134.45, 133.75, 132.70, 131.62, 129.72, 129.25, 128.75, 128.42, 127.66, 125.99, 125.91, 125.72, 124.60, 122.97, 121.40, 118.60, 117.33, 117.27, 116.07, 112.66, 109.61.
f1 (ppm)

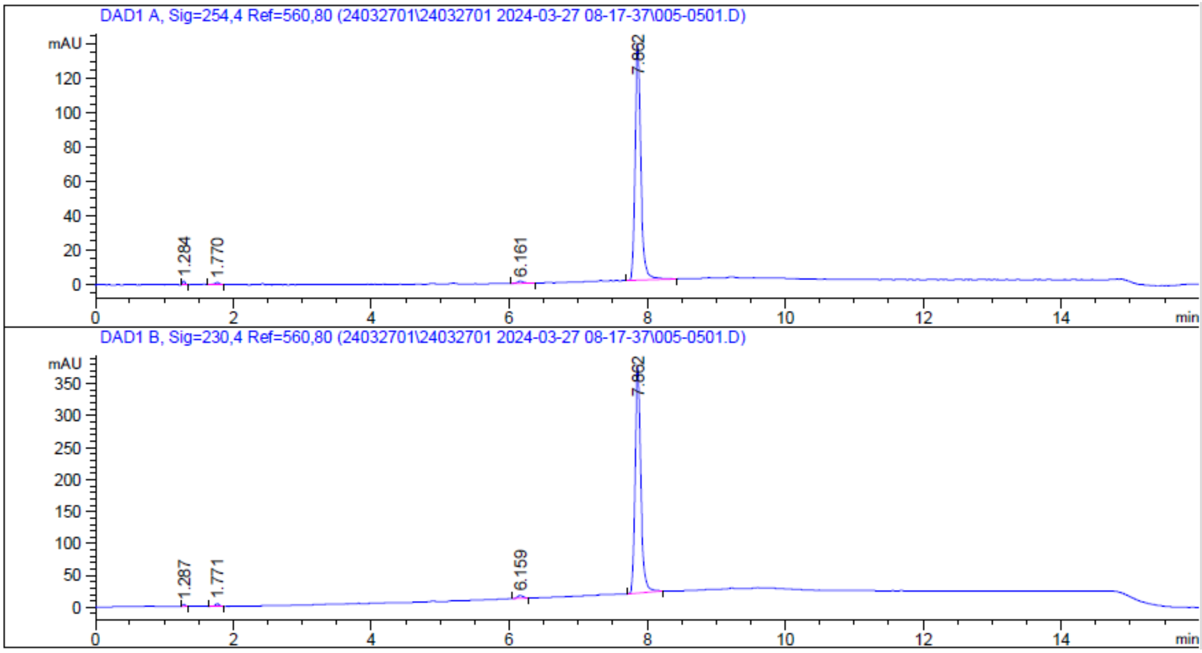

===

Area Percent Report

===

Sorted By : Signal
Multiplier: : 1.0000
Dilution: : 1.0000
Use Multiplier & Dilution Factor with ISTDs

Signal 1: DAD1 A, Sig=254,4 Ref=560,80

Peak #	RetTime [min]	Type	Width [min]	Area [mAU*s]	Height [mAU]	Area %
1	1.284	BB	0.0414	5.62552	2.23223	0.6906
2	1.770	BB	0.0685	5.89263	1.30910	0.7234
3	6.161	BB	0.0930	7.90365	1.26318	0.9703
4	7.862	BB	0.0878	795.15271	136.90475	97.6157

Totals : 814.57451 141.70927

Signal 2: DAD1 B, Sig=230,4 Ref=560,80

Peak #	RetTime [min]	Type	Width [min]	Area [mAU*s]	Height [mAU]	Area %
1	1.287	BB	0.0420	8.08683	3.14014	0.3904
2	1.771	BB	0.0610	16.59250	4.30575	0.8009
3	6.159	BB	0.0858	26.07447	4.62395	1.2587
4	7.862	BB	0.0867	2020.85254	353.40491	97.5500

Totals : 2071.60635 365.47474

===

*** End of Report ***

56c

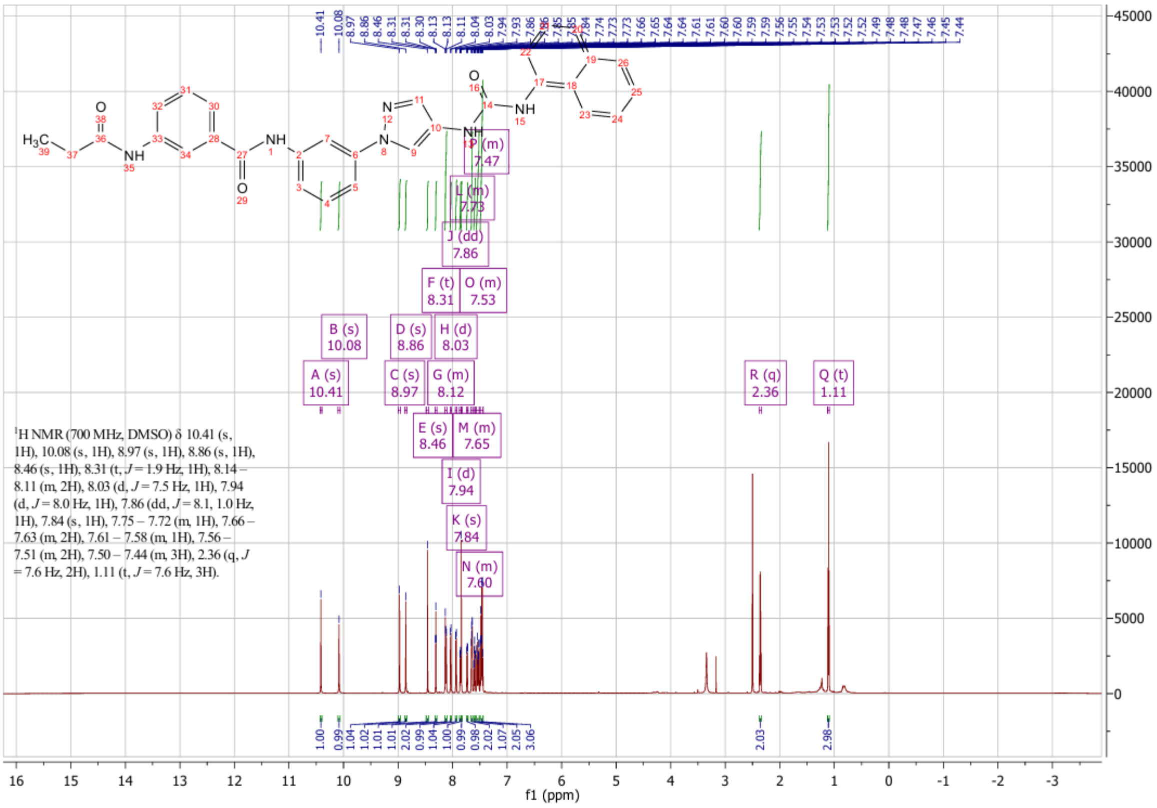
^{1}H NMR (700 MHz, DMSO) δ 10.41 (s, 1H), 10.08 (s, 1H), 8.97 (s, 1H), 8.86 (s, 1H), 8.46 (s, 1H), 8.31 (t, J = 1.9 Hz, 1H), 8.14 – 8.11 (m, 2H), 8.03 (d, J = 7.5 Hz, 1H), 7.94 (d, J = 8.0 Hz, 1H), 7.86 (dd, J = 8.1, 1.0 Hz, 1H), 7.84 (s, 1H), 7.75 – 7.72 (m, 1H), 7.66 – 7.63 (m, 2H), 7.61 – 7.58 (m, 1H), 7.56 – 7.51 (m, 2H), 7.50 – 7.44 (m, 3H), 2.36 (q, J = 7.6 Hz, 2H), 1.11 (t, J = 7.6 Hz, 3H).
f1 (ppm)

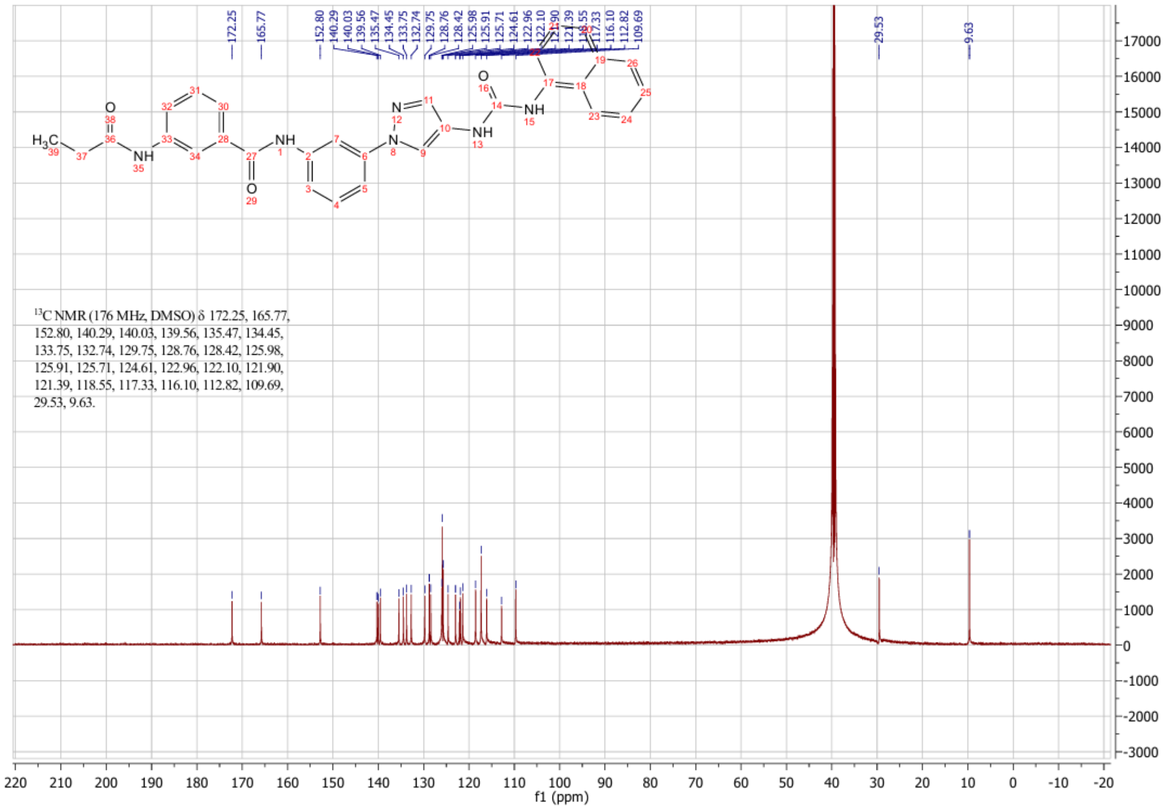
^{13}C NMR (176 MHz, DMSO) δ 172.25, 165.77, 152.80, 140.29, 140.03, 139.56, 135.47, 134.45, 133.75, 132.74, 129.75, 128.76, 128.42, 125.98, 125.91, 125.71, 124.61, 122.96, 122.10, 121.90, 121.39, 118.55, 117.33, 116.10, 112.82, 109.69, 29.53, 9.63.
f1 (ppm)

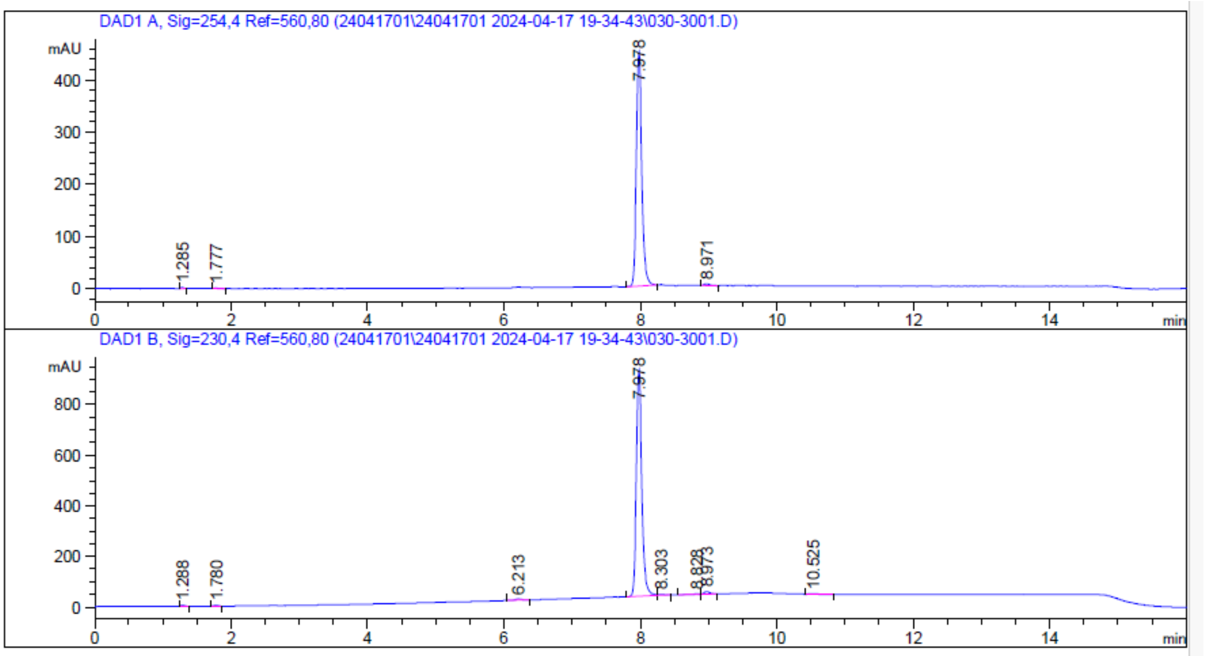

===

Area Percent Report

===

Sorted By : Signal
Multiplier: : 1.0000
Dilution: : 1.0000
Use Multiplier & Dilution Factor with ISTDs

Signal 1: DAD1 A, Sig=254,4 Ref=560,80

Peak #	RetTime [min]	Type	Width [min]	Area [mAU*s]	Height [mAU]	Area %
1	1.285	BB	0.0416	6.35543	2.50095	0.2547
2	1.777	BB	0.0592	4.36659	1.12656	0.1750
3	7.978	BB	0.0839	2464.55176	449.77805	98.7854
4	8.971	BB	0.0859	19.57926	3.57285	0.7848

Totals : 2494.85303 456.97841

Signal 2: DAD1 B, Sig=230,4 Ref=560,80

Peak #	RetTime [min]	Type	Width [min]	Area [mAU*s]	Height [mAU]	Area %
1	1.288	BB	0.0456	12.47554	4.33532	0.2423
2	1.780	BB	0.0560	13.20724	3.66835	0.2565
3	6.213	BB	0.0967	31.38179	4.90422	0.6095
4	7.978	BV	0.0848	4983.63770	897.39404	96.7902
5	8.303	VB	0.0965	32.49044	4.82651	0.6310
6	8.828	BV	0.0708	7.27355	1.49519	0.1413
7	8.973	VB	0.0858	54.70558	9.70673	1.0625
8	10.525	BB	0.1152	13.73291	1.79517	0.2667

Totals : 5148.90475 928.12553

===

*** End of Report ***

56d

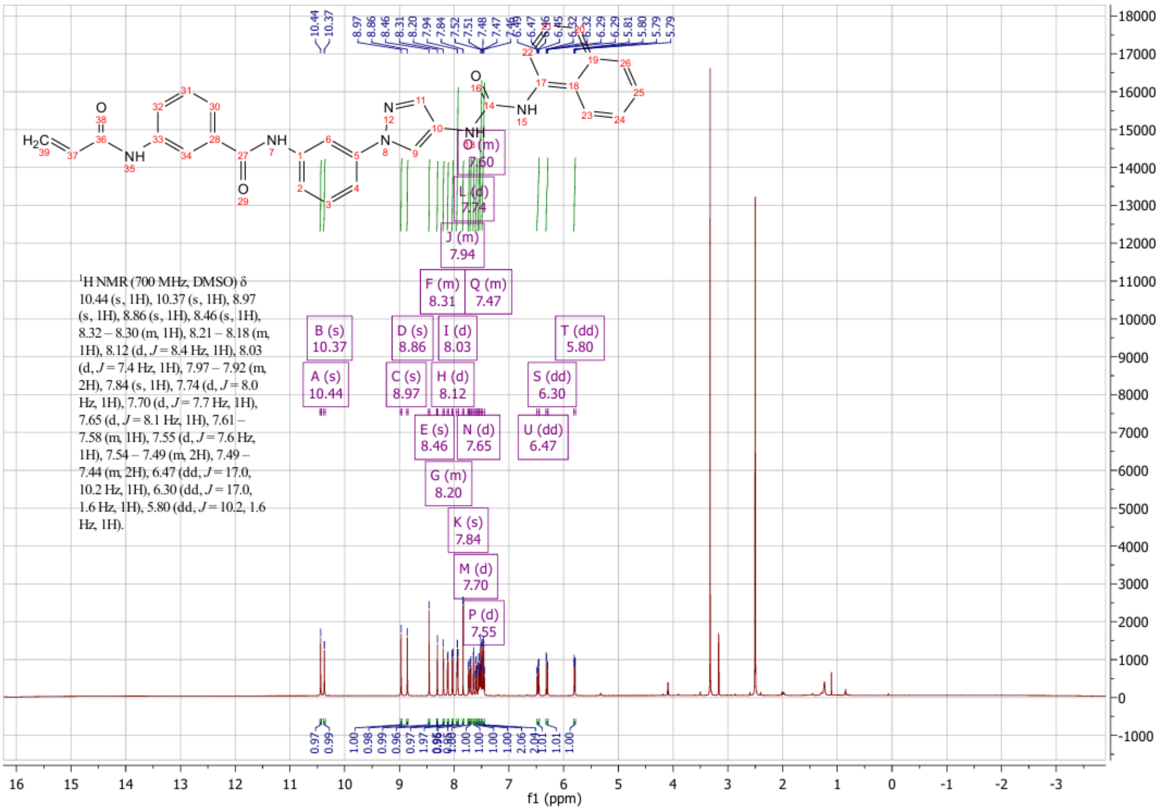

^{1}H NMR (700 MHz, DMSO) δ 10.44 (s, 1H), 10.37 (s, 1H), 8.97 (s, 1H), 8.86 (s, 1H), 8.46 (s, 1H), 8.32 – 8.30 (m, 1H), 8.21 – 8.18 (m, 1H), 8.12 (d, J = 8.4 Hz, 1H), 8.03 (d, J = 7.4 Hz, 1H), 7.97 – 7.92 (m, 2H), 7.84 (s, 1H), 7.74 (d, J = 8.0 Hz, 1H), 7.70 (d, J = 7.7 Hz, 1H), 7.65 (d, J = 8.1 Hz, 1H), 7.61 – 7.58 (m, 1H), 7.55 (d, J = 7.6 Hz, 1H), 7.54 – 7.49 (m, 2H), 7.49 – 7.44 (m, 2H), 6.47 (dd, J = 17.0, 10.2 Hz, 1H), 6.30 (dd, J = 17.0, 1.6 Hz, 1H), 5.80 (dd, J = 10.2, 1.6 Hz, 1H).
f1 (ppm)

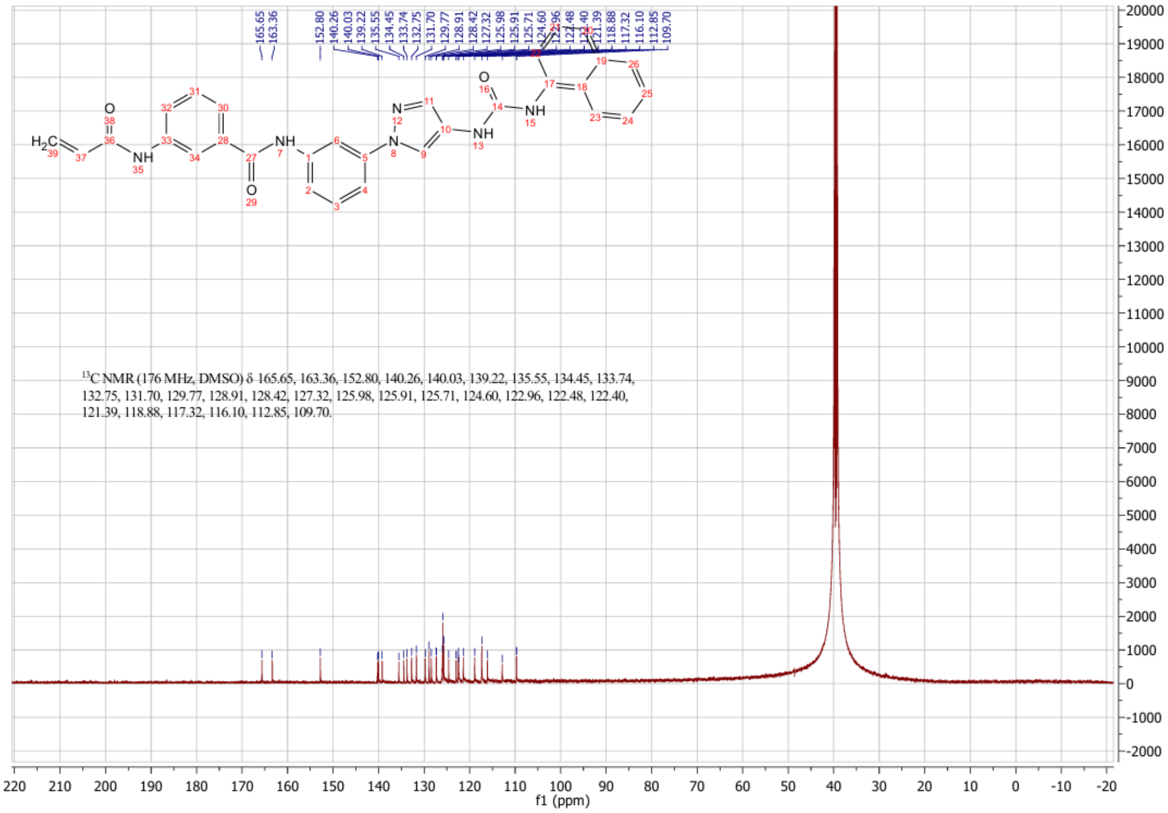

^{13}C NMR (176 MHz, DMSO) δ 165.65, 163.36, 152.80, 140.26, 140.03, 139.22, 135.55, 134.45, 133.74, 132.75, 131.70, 129.77, 128.91, 128.42, 127.32, 125.98, 125.91, 125.71, 124.60, 122.96, 122.48, 122.40, 121.39, 118.88, 117.32, 116.10, 112.85, 109.70.
f1 (ppm)

Compound sample had to be spiked with THF or DMSO to produce a clear solution.

Spiked with THF (THF peak at 1.777 (not integrated)):

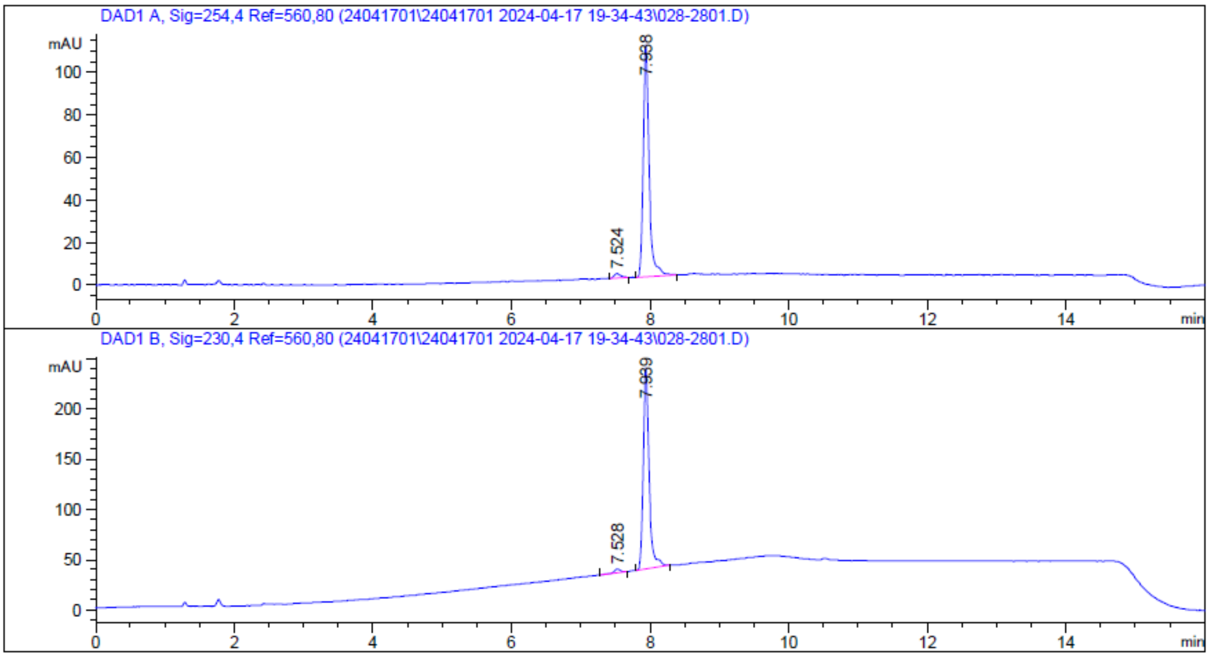

===

Area Percent Report

===

Sorted By : Signal
Multiplier: : 1.0000
Dilution: : 1.0000
Use Multiplier & Dilution Factor with ISTDs

Signal 1: DAD1 A, Sig=254,4 Ref=560,80

Peak #	RetTime [min]	Type	Width [min]	Area [mAU*s]	Height [mAU]	Area %
1	7.524	BB	0.1036	13.99918	2.10531	2.1312
2	7.938	BB	0.0891	642.87567	108.51830	97.8688

Totals : 656.87485 110.62361

Signal 2: DAD1 B, Sig=230,4 Ref=560,80

Peak #	RetTime [min]	Type	Width [min]	Area [mAU*s]	Height [mAU]	Area %
1	7.528	BB	0.0873	23.28967	4.03638	1.9575
2	7.939	BB	0.0884	1166.47644	198.82753	98.0425

Totals : 1189.76611 202.86391

===

*** End of Report ***

Spiked with DMSO (DMSO peak at 1.351 (not integrated)):

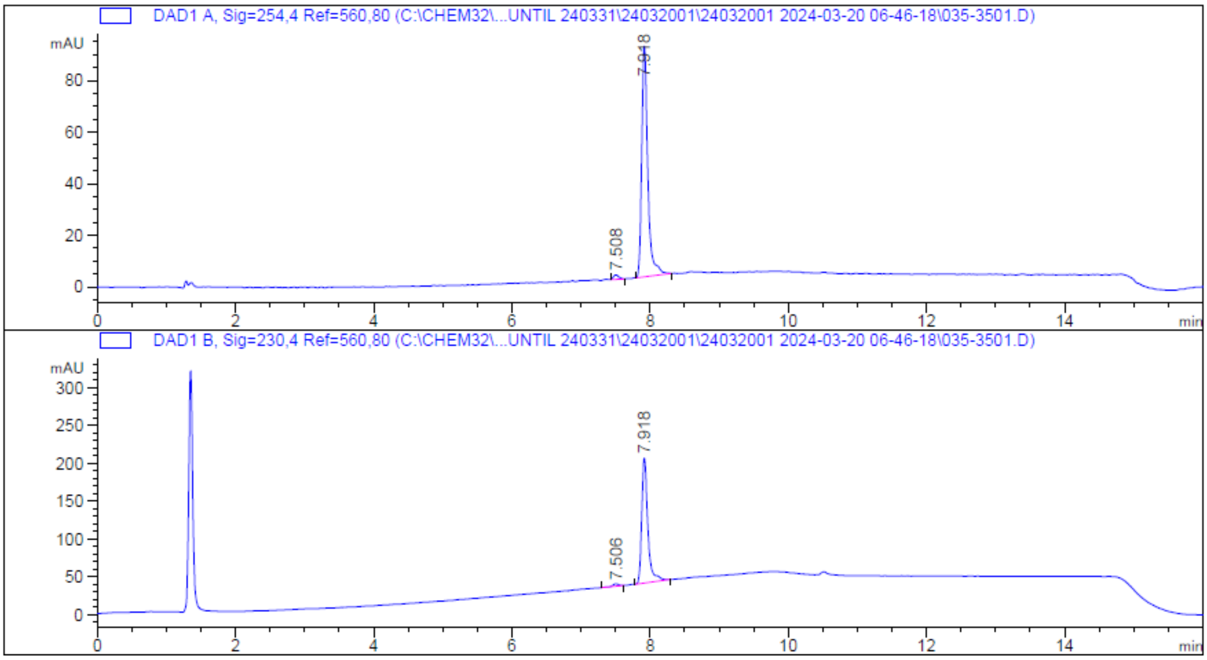

===

Area Percent Report

===

Sorted By : Signal
Multiplier: : 1.0000
Dilution: : 1.0000
Use Multiplier & Dilution Factor with ISTDs

Signal 1: DAD1 A, Sig=254,4 Ref=560,80

Peak #	RetTime [min]	Type	Width [min]	Area [mAU*s]	Height [mAU]	Area %
1	7.508	BB	0.0812	9.16169	1.74538	1.6619
2	7.918	BB	0.0906	542.10254	89.64563	98.3381

Totals : 551.26423 91.39101

Signal 2: DAD1 B, Sig=230,4 Ref=560,80

Peak #	RetTime [min]	Type	Width [min]	Area [mAU*s]	Height [mAU]	Area %
1	7.506	BB	0.0882	18.55192	3.17219	1.8133
2	7.918	BB	0.0908	1004.52502	165.55055	98.1867

Totals : 1023.07694 168.72274

===

*** End of Report ***

12 Supplementary references

(1) Luković, E.; González-Vera, J. A.; Imperiali, B. Recognition-Domain Focused Chemosensors: Versatile and Efficient Reporters of Protein Kinase Activity. *Journal of the American Chemical Society* **2008**, *130* (38), 12821-12827. DOI: 10.1021/ja8046188.
(2) Corporation, R. B. *Protocol HotSpot Kinase Assay*. Reaction Biology Corporation, 2025. https://www.reactionbiology.com/assay-protocol-hotspot/ (accessed 03 May 2025).
(3) Keeley, A.; Ábrányi-Balogh, P.; Keserű, G. M. Design and characterization of a heterocyclic electrophilic fragment library for the discovery of cysteine-targeted covalent inhibitors. *MedChemComm* **2019**, *10* (2), 263-267. DOI: 10.1039/C8MD00327K.
(4) Schwarz, M.; Kurkunov, M.; Wittlinger, F.; Rudalska, R.; Wang, G.; Schwalm, M. P.; Rasch, A.; Wagner, B.; Laufer, S. A.; Knapp, S.; et al. Development of Highly Potent and Selective Covalent FGFR4 Inhibitors Based on SNAr Electrophiles. *Journal of Medicinal Chemistry* **2024**, *67* (8), 6549-6569. DOI: 10.1021/acs.jmedchem.3c02483.
(5) Fabian, M. A.; Biggs, W. H.; Treiber, D. K.; Atteridge, C. E.; Azimioara, M. D.; Benedetti, M. G.; Carter, T. A.; Ciceri, P.; Edeen, P. T.; Floyd, M.; et al. A small molecule–kinase interaction map for clinical kinase inhibitors. *Nature Biotechnology* **2005**, *23* (3), 329-336. DOI: 10.1038/nbt1068.
(6) Zheng, K.; Iqbal, S.; Hernandez, P.; Park, H.; LoGrasso, P. V.; Feng, Y. Design and Synthesis of Highly Potent and Isoform Selective JNK3 Inhibitors: SAR Studies on Aminopyrazole Derivatives. *Journal of Medicinal Chemistry* **2014**, *57* (23), 10013-10030. DOI: 10.1021/jm501256y.
